# Complete sequences of six major histocompatibility complex haplotypes, including all the major MHC class II structures

**DOI:** 10.1111/tan.15020

**Published:** 2023-03-18

**Authors:** Torsten Houwaart, Stephan Scholz, Nicholas R. Pollock, William H. Palmer, Katherine M. Kichula, Daniel Strelow, Duyen B. Le, Dana Belick, Lisanna Hülse, Tobias Lautwein, Thorsten Wachtmeister, Tassilo E. Wollenweber, Birgit Henrich, Karl Köhrer, Peter Parham, Lisbeth A. Guethlein, Paul J. Norman, Alexander T. Dilthey

**Affiliations:** ^1^ Institute of Medical Microbiology and Hospital Hygiene Heinrich Heine University Düsseldorf Düsseldorf Germany; ^2^ Department of Biomedical Informatics Anschutz Medical Campus, University of Colorado Aurora Colorado USA; ^3^ Department of Immunology and Microbiology Anschutz Medical Campus, University of Colorado Aurora Colorado USA; ^4^ Biologisch‐Medizinisches‐Forschungszentrum (BMFZ) Genomics & Transcriptomics Laboratory, Heinrich Heine University Düsseldorf Düsseldorf Germany; ^5^ Department of Structural Biology, and Department of Microbiology and Immunology Stanford University Stanford California USA

**Keywords:** annotation, cell line, HLA, long‐read sequencing, MHC, population, reference graph

## Abstract

Accurate and comprehensive immunogenetic reference panels are key to the successful implementation of population‐scale immunogenomics. The 5Mbp *Major Histocompatibility Complex* (*MHC*) is the most polymorphic region of the human genome and associated with multiple immune‐mediated diseases, transplant matching and therapy responses. Analysis of *MHC* genetic variation is severely complicated by complex patterns of sequence variation, linkage disequilibrium and a lack of fully resolved *MHC* reference haplotypes, increasing the risk of spurious findings on analyzing this medically important region. Integrating Illumina, ultra‐long Nanopore, and PacBio HiFi sequencing as well as bespoke bioinformatics, we completed five of the alternative *MHC* reference haplotypes of the current (GRCh38/hg38) build of the human reference genome and added one other. The six assembled *MHC* haplotypes encompass the DR1 and DR4 haplotype structures in addition to the previously completed DR2 and DR3, as well as six distinct classes of the structurally variable *C4* region. Analysis of the assembled haplotypes showed that *MHC class II* sequence structures, including repeat element positions, are generally conserved within the *DR* haplotype supergroups, and that sequence diversity peaks in three regions around *HLA‐A*, *HLA‐B*+*C*, and the *HLA class II* genes. Demonstrating the potential for improved short‐read analysis, the number of proper read pairs recruited to the *MHC* was found to be increased by 0.06%–0.49% in a 1000 Genomes Project read remapping experiment with seven diverse samples. Furthermore, the assembled haplotypes can serve as references for the community and provide the basis of a structurally accurate genotyping graph of the complete *MHC* region.

## 1 INTRODUCTION

The *Major Histocompatibility Complex* (*MHC*) is the most polymorphic region of the human genome, and is associated with more diseases, than any other region.^1^, ^2^, ^3^, ^4^ The *MHC* spans 5Mbp of human chromosome 6 and encodes ~165 proteins, as well as numerous *cis* and *trans*‐acting factors.^5^, ^6^, ^7^, ^8^ Over 40% of the encoded proteins are directly involved in immunity. Additional to HLA class I and II that control innate and adaptive immunity, are proteins that process the peptide substrates for presentation (e.g., TAP, tapasin, DM, and DO), systemically acting complement components and cytokines (e.g., C4, TNFα), as well as transcription factors that promote or mediate immune responses (e.g., NFκB). Also encoded in the *MHC* are structural and developmental proteins (e.g., CDSN, NOTCH4), and other polymorphic molecules including MICA and HSP that act in stress‐induced responses to infection. Most of the *MHC* region genes exhibit polymorphism and thus a strong potential to impact immune‐mediated disease.^9^ Characterizing the *MHC* are intricate patterns of hyper‐polymorphism, structural diversity and linkage disequilibrium (LD).^10^, ^11^, ^12^ Such complexity arises through a dynamic evolutionary mechanism of natural selection and population demography.^13^, ^14^ The dense sequence and structural diversity hinders attempts to comprehensively genotype *MHC* variation.^15^ The complex and insufficiently characterized patterns of LD, and to some extent the related functions of the encoded proteins, create additional challenges for fine mapping disease associations.^16^, ^17^ For these reasons, it is of paramount importance to generate complete and accurate reference sequences that represent the extent of human genomic diversity in the *MHC*.^18^, ^19^

Within the *MHC*, most notable in their structural diversity and sequence divergence are the *MHC class II* and *C4* regions. The defining components of *MHC class II* include variable presence of the divergent *HLA‐DRB3‐5* genes and their LD with major *DRB1* variants,^20^, ^21^ as well as clearly established hotspots of meiotic recombination.^22^ The defining characteristics of the *C4* region are gene duplication and resulting sequence homology, with up to four copies of the *C4* gene recorded per haplotype, as well as variable presence of a 19 kb HERV insertion that affects expression of some alleles.^23^ Whereas imputation from dense SNP data has helped pinpoint some of the specific alleles associated with disease,^24^ low accuracy due to incompletely characterized sequence and LD patterns across populations, especially in the *C4* and *MHC class II* regions, can reduce the clinical utility of this approach.^25^, ^26^ The most promising solutions for cataloging *MHC* sequence complexity are graph‐based approaches^5^ that can represent all forms of genomic diversity without reference bias. The utility of these existing graph‐based approaches beyond the classical *HLA* genes^27^, ^28^, ^29^, ^30^, ^31^, ^32^, ^33^, ^34^ remains limited because of a lack of fully resolved sequences that could be used to define a structurally accurate genotyping graph for *C4* and *MHC class II* and other genes in the *MHC* region.

Here we present and validate six fully resolved *MHC* sequences from homozygous cell lines as a reference for the community and as the basis for further methods development. We targeted five such cell lines (APD, DBB, MANN, QBL, SSTO) that were partially sequenced from BAC clones by the “MHC Haplotype Project”^35^, ^36^, ^37^, ^38^ and further characterized from targeted short‐read data.^9^ They form part of the current version of the human reference genome, GRCh38, being classified as alternative reference sequences (“alt_ref”) for the *MHC* region.^39^ With our work, we increase the number of completely resolved *MHC* haplotypes of the cell lines present in GRCh38 from 2 to 7. In addition, we included one cell line, KAS116, that lacks *HLA‐DRB3*, *‐DRB4*, or *‐DRB5* genes, thus representing one of two major *MHC class II* sequence structures currently not represented in GRCh38. Due to their importance for human health and difficulty of conventional genotyping, we focused our preliminary assessment of sequence features on the *MHC class II* and *C4* regions.

## 2 METHODS

### 2.1 Culture of six *MHC* ‐homozygous cell lines

Six *MHC*‐homozygous cell lines were cultured for fresh DNA extraction. We targeted five of the six cell lines that were partially sequenced as part of the “MHC Haplotype Project” (the sixth, MCF, was unavailable at the time of study). In addition, we chose one cell line to represent the DR1 haplotype (Table 1). All six cell lines were shown previously to be homozygous through the entire *MHC* region,^40^, ^41^ and their reference *HLA class I* and *II* genotypes are shown in Table 1. The cells investigated here are maintained by the European Collection of Cell Cultures (ECACC) and were purchased from Sigma‐Aldrich, or obtained from the International Histocompatibility Working Group (IHWG) repository (http://www.ihwg.org/). Cells were cultured in RPMI 1640 (1×) media containing 100 mL Foetal calf serum (20%), 5 mL Penicillin/Streptomycin (1%) and 5 mL Glutamine (2 mM). For initial seeding, cells were diluted to 3 × 10^5^ cells/mL from cryopreserved stock. Cells were washed in Phosphate buffered salts, pelleted beforehand by centrifugation (1200 rpm, 5 min.), and redistributed at 1 × 10^6^ cells/mL. Cells were harvested at 1 × 10^7^ cells/mL and pelleted.

**TABLE 1:** Studied cell lines and their HLA genotypes.

Cell line	IHIW ID	*HLA‐A*	*HLA‐B*	*HLA‐C*	*HLA‐DRB345*	*HLA‐DRB1*	*HLA‐DQA1*	*HLA‐DQB1*	*HLA‐DPA1*	*HLA‐DPB1*
APD	IHW09291	A*01:*01*:*01*:*01*	B*40:*01*:*02*:*01*	C*06:*02*:*01*:*01*	DRB3*02:*02*:*01*:*01*	DRB1*13:*01*:*01*:*02*	DQA1*01:*03*:*01*:*02*	DQB1*06:*03*:*01*:*01*	DPA1*01:*03*:*01*:*05*	DPB1*04:*02*:*01*:*01*
COX^a^	IHW09022	A*01:*01*:*01*:*01*	B*08:*01*:*01*:*01*	C*07:*01*:*01*:*01*	DRB3*01:*01*:*02*:*01*	DRB1*03:*01*:*01*:*01*	DQA1*05:*01*:*01*:*02*	DQB1*02:*01*:*01*:*01*	DPA1*01:*03*:*01*:*03*	DPB1*03:*01*:*01*:*01*
DBB	IHW09052	A*02:*01*:*01*:*01*	B*57:*01*:*01*:*01*	C*06:*02*:*01*:*01*	DRB4*01:*03*:*01*:*01N*	DRB1*07:*01*:*01*:*02*	DQA1*02:*01*:*01*:*01*	DQB1*03:*03*:*02*:*01*	DPA1*01:*03*:*01*:*02*	DPB1*04:*01*:*01*:*01* ^b^
KAS116	IHW09003	A*24:*02*:*01*:*01*	B*51:*01*:*01*:*03*	C*12:*03*:*01*:*01*		DRB1*01:*01*:*01*:*01*	DQA1*01:*01*:*01*:*01*	DQB1*05:*01*:*01*:*02*	*Novel* (closest match: DPA1*02:*01*:*01*:*01*)^b^	DPB1*13:*01*:*01*:*01*
MANN	IHW09050	A*29:*02*:*01*:*01*	B*44:*03*:*01*:*01*	C*16:*01*:*01*:*01*	DRB4*01:*01*:*01*:*01*	DRB1*07:*01*:*01*:*01*	DQA1*02:*01*:*01*:*01*	DQB1*02:*02*:*01*:*01*	DPA1*01:*03*:*01*:*01*	DPB1*02:*01*:*02*:*01*
PGF^a^	IHW09318	A*03:*01*:*01*:*01*	B*07:*02*:*01*:*01*	C*07:*02*:*01*:*03*	DRB5*01:*01*:*01*:*01*	DRB1*15:*01*:*01*:*01*	DQA1*01:*02*:*01*:*01*	DQB1*06:*02*:*01*:*01*	DPA1*01:*03*:*01*:*02*	DPB1*04:*01*:*01*:*01*
QBL	IHW09020	A*26:*01*:*01*:*01*	B*18:*01*:*01*:*01*	C*05:*01*:*01*:*01*	DRB3*02:*02*:*01*:*01*	DRB1*03:*01*:*01*:*01*	DQA1*05:*01*:*01*:*01*	DQB1*02:*01*:*01*:*01*	DPA1*01:*03*:*01*:*01*	DPB1*02:*02*:*01*:*01*
SSTO	IHW09302	A*32:*01*:*01*:*01*	B*44:*02*:*01*:*01*	C*05:*01*:*01*:*02*	DRB4*01:*03*:*01*:*03*	*Novel* (closest match: DRB1*04:*03*:*01*:*01*)^b^	DQA1*03:*01*:*01*:*01*	DQB1*03:*05*:*01*	DPA1*01:*03*:*01*:*04*	DPB1*04:*01*:*01*:*01*

*Note*: Shows the *HLA class I* and *II* IPD‐IMGT/HLA reference genotypes of the six MHC homozygous cell lines analyzed here, and two that were completed previously (PGF and COX).

^a^ Complete from hg19 onwards.

^b^ Sequence of assembled haplotype different from IPD‐IMGT/HLA reference HLA type.

### 2.2 Single‐molecule nanopore sequencing of six *MHC* ‐homozygous cell lines

High molecular weight DNA was extracted following the protocol for ultra‐long read nanopore sequencing.^42^ In summary, cells were lysed, digestion was performed using proteinase K, and DNA extraction using phenol/chloroform. Precipitated DNA was spooled onto a glass rod and washed in 70% Ethanol. Sequencing was carried out using the Oxford Nanopore sequencing platform, employing the MinION, GridION and PromethION devices following either the ultra‐long protocol for library preparation^42^ or the regular Oxford Nanopore Ligation Kits (SQK‐LSK108/109). For ligation‐based library preparation, ultra‐high molecular weight DNA was sheared to a size of ~75 kb using the Megaruptor 2 device and library preparation was performed following Oxford Nanopore's protocol. Generated data for each cell line are summarized in Table S1 and full details on the sequencing runs conducted are given in Table S2.

### 2.3 Single molecule, real‐time (SMRT) sequencing

High molecular weight (HMW) DNA for single molecule, real‐time (SMRT) sequencing was extracted using the Nanobind CBB kit. High‐fidelity (HiFi) SMRTbell libraries were prepared using the SMRTbell® prep kit 3.0 (PacBio, CA, USA). In summary, 6 μg of HMW DNA from each sample was sheared with hydropores deriving from the Megaruptor 3 DNAFluid+ and the Megaruptor 3 shearing‐kit (Diagenode, MA, USA) to 16–21 kb. Subsequently, DNA damage and fragment ends were repaired, adapters ligated (in some instances employing barcoding) and fragments cleaned. Incomplete SMRTbell templates were removed by a nuclease treatment and purified. All required reagents were included in the SMRTbell® prep kit 3.0 and barcodes were used from the Barcoded overhang adapter kit 8A. Large‐insert SMRTbell libraries for sequencing were achieved by using a size selection with a 10 kb cut‐off using the BluePippin system (SageScience, MA, USA). SMRT sequencing was carried out using the Sequel II/e systems on SMRT Cells 8 M (PacBio, CA, USA). CCS reads were generated and demultiplexing (if required) was performed using standard settings in SMRTLink v11.0 (PacBio, CA, USA), with min passes = 3 and min read quality = 0.99. Generated data for each cell line are summarized in Table S1 and full details on the sequencing runs conducted are given in Table S2.

### 2.4 DNA sequencing (Illumina)

DNA was extracted for sequencing using the Qiagen Blood and Tissue Kit (Cat. No. 69506). Prior to library preparation 2500 ng of gDNA were sheared with Covaris ME220 (Covaris, Inc.) to a mean fragment size of 550 bp. Library preparation was performed using the VAHTS Universal DNA Library Prep Kit for Illumina (Vazyme Biotech Co.; Ltd) according to the manufacturer's protocol, without any amplification step, but with an additional size selection step after adapter ligation to remove smaller fragments. The library was quantified by qPCR by using KAPA library quantification kit (Roche Diagnostics Corporation) and a QuantStudio 3 (Thermo Fisher Scientific Inc.), and then sequenced using a HiSeq3000 system (Illumina Inc) with a read setup of 2 × 151 bp.

### 2.5 Initial assembly of *MHC* sequences

Nanopore *MHC* reads were collected by alignment against previously assembled draft scaffolds of cell‐line‐specific contigs,^9^ using minimap2 version 2.14‐r892‐dirty.^43^ Draft *MHC* assemblies were created from the Nanopore *MHC* reads using Canu^44^ versions 1.7, 1.8, and 1.9, as well as Flye^45^ version 2.6, empirically exploring the algorithms' parameter spaces until a draft assembly containing a single *MHC* contig from a single algorithm had been obtained, which was then checked for structural consistency by long‐read‐to‐assembly alignment and visual inspection. *MHC* draft assemblies were trimmed to “canonical” *MHC* coordinates by alignment against the GRCh38 *MHC* reference sequence (PGF) using nucmer^46^ version 3.23, and polished in multiple iterations (see next section).

### 2.6 Polishing *MHC* sequences

Polishing was carried out as an iterative process. Each iteration of polishing consisted of the following steps:

1. Nanopore polishing. Alignment of the Nanopore *MHC* reads against the input assembly sequence with minimap2^43^ and polishing using medaka 1.0.1 (https://github.com/nanoporetech/medaka); during the first round of polishing, medaka was used in “consensus” mode; during the second and all subsequent rounds, medaka was used in “variant” mode and the polishing was performed by substituting homozygous variant calls into the assembly sequence according to the Medaka‐generated VCF.
2. Illumina polishing. Whole‐genome Illumina reads were aligned against a version of the primary (contains no “alt_refs” or “decoys”) human reference genome GRCh38, in which the reference *MHC* sequence (PGF) was masked, and in which the output of step 1 was inserted as a separate contig, using BWA‐MEM 0.7.15.^47^ GATK^48^ 4.1.4.1 variant calling was applied to the *MHC* contig, and a polished version of the *MHC* contig was produced by substituting homozygous variant calls into the assembly sequence according to the GATK‐produced VCF.
3. Contig‐based polishing. Cell‐line‐specific contigs^9^ were aligned against the output of Step 2 and “high‐quality” contig alignments were defined as alignments with “query cover” × “alignment identity” ≥ 0.99. The sequences of all high‐quality contig alignments were substituted into the assembly sequence to obtain a polished assembly.

Polishing was carried out iteratively until manual inspection with IGV^49^ and curation of the assemblies was indicative of high quality assembly. The number of polishing rounds per assembled *MHC* haplotype is listed in Table S1.

After the last round of the iterative polishing process described above, two final steps of polishing were carried out. First, polishing using a simple majority‐based assembly improvement process: At each position in the assembled *MHC* haplotypes, the majority allele of the aligned Nanopore *MHC* and Illumina *MHC* reads was determined using samtools 1.6 mpileup.^50^ If the Nanopore and Illumina majority alleles were in agreement and (i) accounted for more than 50% of aligned alleles at the considered position in their respective datasets; (ii) represented no INDEL; and (iii) disagreed with the allele carried by the assembly at the considered position, the assembly allele was replaced by the Nanopore+Illumina majority allele. Second, PacBio HiFi‐based polishing: PacBio sequencing reads were aligned using minimap2^43^ and variants were called using DeepVariant 1.4.0^51^; the variants corresponding to the highest‐frequency alleles observed in the sequencing reads were substituted into the assembly sequences. Spatially clustered variant calls (i.e. entries in the DeepVariant‐generated VCF less than 6 bp apart) were manually inspected.

### 2.7 Comparison to IPD‐IMGT/HLA reference HLA types

Genomic HLA sequences and reference HLA types of the sequenced cell lines for 19 HLA loci (Table S3) were obtained from IPD‐IMGT/HLA.^52^ For each assembled haplotype and each HLA locus, all IPD‐IMGT/HLA‐defined genomic allele sequences were mapped against the assembled haplotype using minimap2,^43^ and the allele identifier of the closest match was extracted and compared with the corresponding reference HLA type. If the closest match was not identical to the reference HLA type, or if the closest match was incomplete (i.e., if it did not span the entirety of the IPD‐IMGT/HLA‐provided multiple sequence alignment of defined alleles at the locus), the sequence of the complete locus was extracted; if the closest match was incomplete, the coordinates used for the extraction process were determined based on the closest complete match. Mismatches between the assembled haplotypes and reference HLA types were adjudicated by manual inspection of the raw sequencing reads in IGV.^49^

### 2.8 Structural analysis

Multiple sequence alignments were computed and visualized using mauve.^53^ Pairwise sequence alignments were computed with nucmer (parameters—maxmatch—nosimplify—mincluster 300) version 3.1^46^ and visualized using mummerplot and gnuplot. For analysis of *MHC class II* sequences, analysis with mauve was found to be sensitive to selection of the “seed weight” parameter. We empirically investigated multiple settings for “seed weight” and settled on a value of 22 for visualization because it identified a reverse‐complemented segment (see the Section 3), the existence of which we verified with minimap 2. Note that, at “seed weight” value 22, mauve generates non‐syntenic alignments between different members of the *HLA‐DRB* family for some haplotypes.

### 2.9 Gene annotation

The generated *MHC* assemblies were annotated by comparison against the IPD‐IMGT/HLA^52^ database, comprising 14 genes and 25 pseudogenes, and the *MHC* components of the RefSeqGene (RSG) and RefSeq (RS)^54^ databases, comprising 87 and 163 genes, respectively. For the genes and pseudogenes represented in IPD‐IMGT/HLA, “genomic” sequences (representing full‐length allelic variants of the included genes) were mapped^43^ against the *MHC* assemblies. Alignments were filtered for complete alignments covering the query sequence in its entirety, and the highest‐scoring alignment was selected to determine the start and end positions of the gene, for annotation purposes. If no complete alignment was found, no annotation was generated. For *MHC* genes not represented in IPD‐IMGT/HLA, genomic reference sequences were extracted from RSG and, for genes not represented in RSG, from RS. Transcript structure was determined by projecting intron‐exon boundaries from the query sequence of the selected highest scoring alignment onto the *MHC* assembly, and the deduced transcript was checked for translational consistency (presence of start and stop codons; absence of nonsense variants in coding regions). Except for transcripts previously shown^9^ to encode incomplete gene products, no annotations were generated for which the implied transcript exhibited any translational inconsistencies. At the last step, annotation coordinates were converted from GFF into SQN format, using NCBI's table2asn_GFF tool. Any transcripts failing the conversion process were removed.

### 2.10 C4 genotyping

C4 polymorphism is characterized by three major factors of functional importance: gene copy number, antigenic determinants of the C4A and C4B isotypes, and presence (L) or absence (S) of a human endogenous retroviruses (HERV) insertion that reduces expression.^23^, ^55^ C4 copy number and genotypes were determined by mapping the C4A sequence of GRCh38,^39^ which contains the HERV element, to the assembled *MHC* sequences using minimap2.^43^ The identified matches were classified with respect to HERV insertion and C4A/B status by (i) determining whether the corresponding alignment contained a deletion relative to the aligned C4A reference sequence between exons 9 and 10 (corresponding to HERV insertion status) and (ii) by determining whether the translated (i.e., amino acid) sequence of exon 26 contained the C4A/B‐defining sequences “PCPVLD” (C4A) or “LSPVIH” (C4B).

### 2.11 Repeat element annotation

Repeat elements were identified using RepeatMasker^56^ version 4.1.2‐p1. To obtain a common coordinate system for cross‐haplotype display of repeat element positions, a multiple sequence alignment of the assembled *MHC* sequences was computed using mafft^57^ version 7.490.

### 2.12 Quantification of polymorphisms

In order to count polymorphisms of a given *MHC* haplotype sequence relative to the reference *MHC* (the PGF haplotype, part of the canonical chromosome 6 sequence of the human reference genome), a pairwise sequence alignment was computed using minimap2.^43^ The alignments corresponding to the mapped regions of the query sequence were projected onto the reference sequence, starting with the longest alignment. Each base of the query sequence was projected only once, even in the presence of overlapping supplementary alignments or secondary alignments. Small variants and INDELs were counted based on the projected pairwise alignments; structural variants (SVs) were defined as insertions or deletions of length ≥ 1000 bp encoded by the CIGAR strings of the projected alignments, or as stretches along the query or reference sequences of more than 1000 bp in length with no projected alignments. Of note, no separate analysis of inversions over and above the mauve‐based analysis described above was performed during this step.

### 2.13 Short‐read mapping experiment

To investigate the effect on short‐read mapping using the complete *MHC* assemblies presented here, (instead of the incomplete versions currently used in GRCh38), we performed a comparative mapping experiment. Seven samples were selected from the 1000 Genomes Project and their read data obtained from the resequencing effort by the New York Genome Center.^58^ Two genome references were created: (i) the 1000 Genomes Project^59^ GRCh38‐based reference genome with the HLA allele sequences removed and the “alt_ref” *MHC* haplotypes retained; and (ii) a modified version in which the incomplete *MHC* haplotypes were substituted with the complete assemblies presented here, including KAS116 (which is not represented in GRCh38). In addition, a short *MHC* region “alt_ref” contig (KI270758; 76,752 bp) that we identified in GRCh38 and which maps to the KAS116 assembly was removed. Whole genome Illumina sequencing reads were aligned independently to each reference using BWA‐MEM.^47^ Sample selection for the comparative mapping experiment was based on previously determined *HLA‐DRB1* and *‐DQB1* genotypes,^60^, ^61^ targeting samples homozygous at these loci that matched the most (PGF) and least (APD) complete *MHC* references in GRCh38 at 2‐field resolution (Table S3).^35^ In addition, as we had sequenced KAS116 because it carries no *HLA‐DRB3*/*4*/*5* genes, we selected HLA‐DRB1*08 and HLA‐DRB1*10 homozygous samples, which also carry no *HLA‐DRB3*/*4*/*5* genes. For evaluating the comparative mapping experiment, we report (i) the number of reads aligning to any location in the utilized reference genome and (ii) the number of reads aligning to the included *MHC* sequences. For the MHC metric, only read alignments with the “proper pair” flag set to 1 are considered, that is, indicating the successful alignment of both reads of a read pair with correct read orientations and a plausible insert size.

## 3 RESULTS

### 3.1 Assembly of six fully resolved finished *MHC* reference haplotypes

**FIGURE 1:**
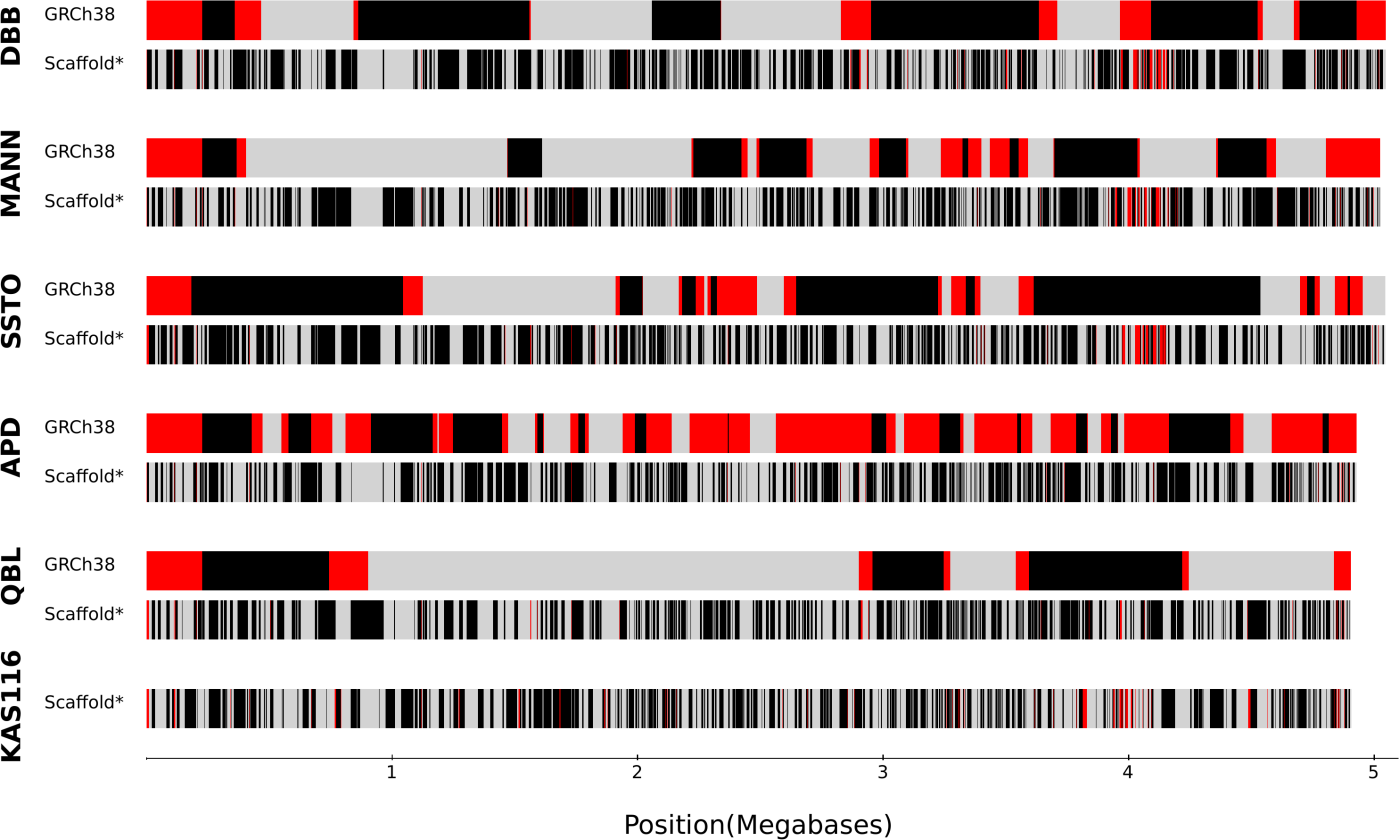
Contiguity and completeness increase over previous *MHC* assemblies. Shown for each of six haplotypes are comparisons to the assembly present in the current genome build (GRCh38: upper) and the short‐read contigs scaffolds from^9^ (SRC: lower). Regions absent from a previous assembly are colored in red. Color changes from black to gray or vice versa indicate distinct contigs that were not physically joined or separated by more than five undefined bases in the previous assemblies.

We integrated sequencing reads generated through ultra‐long Nanopore, PacBio HiFi, whole‐genome Illumina, and targeted Illumina^9^ methods, together with tailored bioinformatics, to obtain high‐quality, fully resolved assemblies of *MHC* haplotypes from five *MHC*‐homozygous cell lines (Table 2). These haplotypes (APD, DBB, MANN, QBL, SSTO) complete five of six unfinished versions present in the current build of the human reference genome (GRCh38) and complement the two completed haplotypes of PGF and COX already present.^35^ We also generated a fully resolved high‐quality assembly of the *MHC* region from the KAS116 cell line using Nanopore, PacBio HiFi, and targeted Illumina sequence data. The newly assembled haplotypes range in length from 4.90 to 5.05 Mbp (Figure 1). Compared with the GRCh38 versions of the same haplotypes, we resolved from 615 kbp (QBL) to 2.6 Mbp (APD) of additional DNA sequence. Compared with previously generated scaffolds,^9^ we resolved from 72 kbp (QBL) to 245 kbp (KAS116) of additional sequence (Table S4). With the addition of KAS116, these four haplotypes represent the major *MHC class II* structural categories, DR1 (*DRB1* only; KAS116), DR2 (*DRB1* + *DRB5*; PGF), DR3 (*DRB1* + *DRB3*; APD, COX, QBL) and DR4 (*DRB1* + *DRB4*; DBB, MANN, SSTO).

**TABLE 2:** Sequenced cell lines and assembled haplotypes—summary of sequencing and assembly statistics

Cell line	MHC class II type	MHC assembly	MHC class II	MHC read depth			GenBank ID
		(bp)	(bp)	Nanopore	Illumina (WG)	PacBio (WG)	
APD	DR3	4.928.029	149.477	31,65	16,26	11,10	OK649231
DBB	DR4	5.048.108	260.711	20,03	12,91	11,50	OK649232
MANN	DR4	5.025.203	259.962	27,62	18,19	11,10	OK649234
SSTO	DR4	5.045.615	258.762	22,43	15,04	9,61	OK649236
KAS116	DR1	4.907.004	155.951	85,87	NA	11,10	OK649233
QBL	DR3	4.904.614	149.416	17,81	12,86	8,95	OK649235
COX	DR3	4.795.265	153.371	NA	NA	NA	GL000251.2
PGF	DR2	4.873.646	169.898	NA	NA	NA	chr6:28510120–33.480.575 (GRCh38)

### 3.2 Comparison to reference HLA types

We compared the HLA gene sequences of the assembled haplotypes to IPD‐IMGT/HLA reference HLA types for the sequenced cell lines (Table S3). In 87% of cases (88 out of 101 comparisons across 18 *HLA* loci), we found that the reference HLA type perfectly matched the sequence of the assembled haplotype; in 6 of these cases, we could complete the definition of an incomplete reference allele based on the assembled complete haplotype. Conversely, we observed a discrepancy in 13% of cases (13 out of 101 comparisons); these were typically small (edit distance 1 or 2 in 8/13 cases) and could be conclusively confirmed by visual inspection in 9/13 cases. Furthermore, the majority of discrepancies could be explained by perfect matches to closely related IPD‐IMGT/HLA reference alleles (9/13 cases), and 3 of these could be completed based on the assembled haplotypes. Visual inspection conclusively confirmed the presence of novel alleles not yet present in IPD‐IMGT/HLA in 2 cases, one closely related to DPA1*02:*01*:*01*:*01* (edit distance 1) and the other to DRB1*04:*03*:*01*:*01* (edit distance 2). Extracted full‐length HLA gene sequences are provided in File S1, and novel allele sequences were submitted to IPD‐IMGT/HLA.

### 3.3 Haplotype annotation and C4 genotypes

Using a semi‐automated annotation approach (see Section 2), we mapped the locations of 160–163 genes and their transcripts, and 7–10 pseudogenes onto the respective haplotypes (Table 3). The annotations are included in the GenBank submissions of the sequences. Independently, we also determined the C4 genotypes (Table 3). The eight completed haplotypes represent six distinct classes of C4 status, C4‐AL (QBL), AL,AL (KAS116), AL,BL (APD, PGF, SSTO) AL,BS (DBB), BL (MANN), and BS (COX) (Figure S1). Of note, we determined the C4 genotype of SSTO to be C4‐AL,BL, in contrast to GRCh38, in which SSTO carries a C4‐BS,BS structure.

**TABLE 3:** Sequenced cell lines and assembled haplotypes—Summary of annotation and genomic variation.

Cell line	Annotation			C4 genotype	Variants relative to PGF		
	Genes	Pseudogenes	Transcripts		SNPs	INDELs	SVs
APD	163	10	162	AL,BL	12.106	2.075	31
DBB	161	10	161	AL,BS	13.355	2.166	48
MANN	161	10	164	BL	13.171	2.179	41
SSTO	163	10	162	AL,BL	13.151	2.361	38
KAS116	160	7	163	AL,AL	13.841	2.415	31
QBL	160	10	163	AL	13.390	2.441	30
COX	NA	NA	NA	BS	13.462	2.306	33

*Note*: Structural variants are defined as insertions or deletions of more than 1000 bp in length. C4 genotypes are specified in a format including the C4 gene (“A” for C4A and “B” for C4B) and whether a HERV element insertion is present (“long”/L) or not (“short”/S).

### 3.4 Large‐scale *MHC* sequence structures and density of genetic variation

**FIGURE 2:**
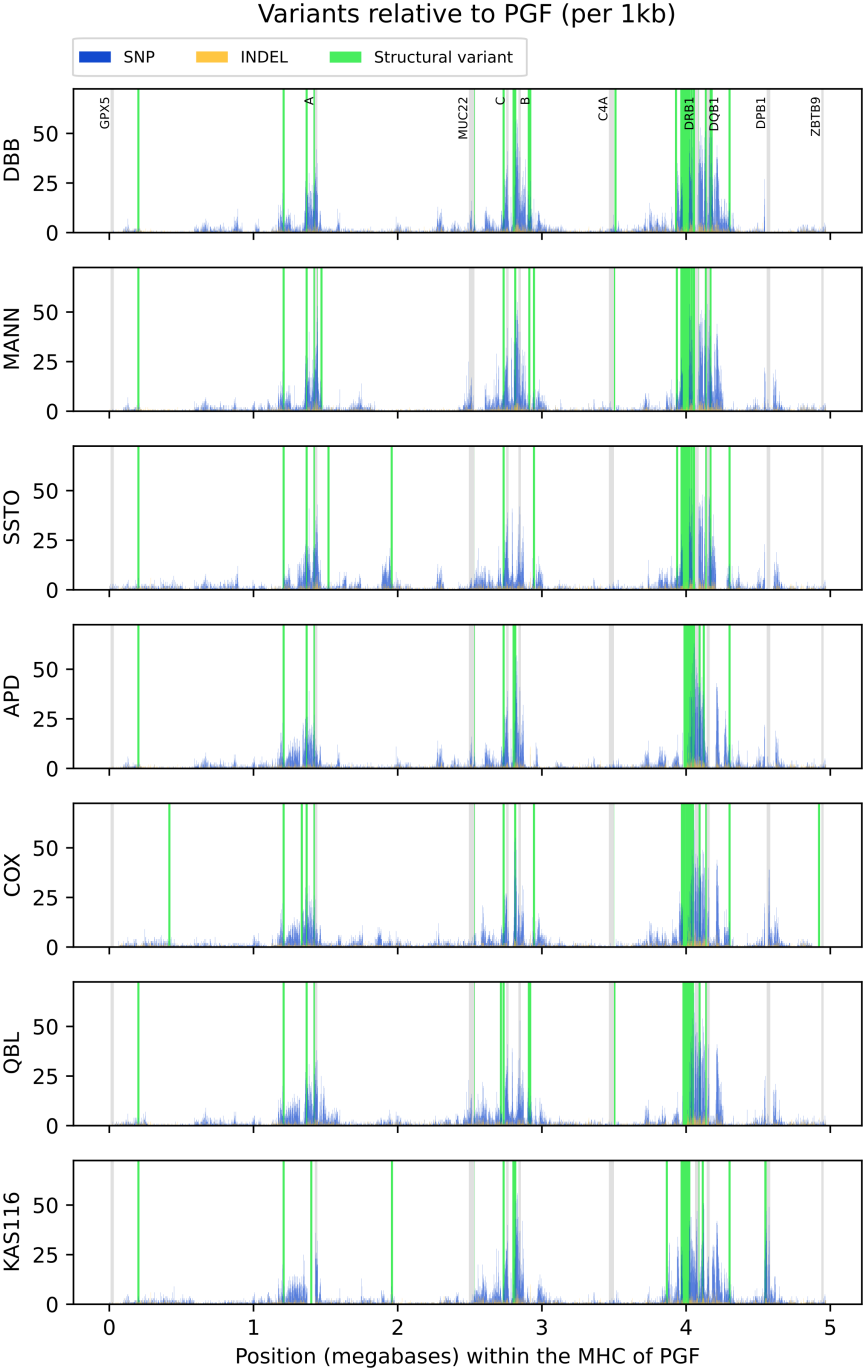
Sequence and structural variation of the completed *MHC* haplotypes. Shown are the genomic variations relative to the GRCh38 reference (PGF) within each completed *MHC* region haplotype. (blue) single‐nucleotide polymorphism (SNP), (gold) insertion–deletion (INDEL), (green) structural variants (indels >1000 bp, or inversions). (gray) selected genes for orientation (names indicated at top). The variants were counted in 1 kbp nonoverlapping windows. See Section 2 for details.

With the exception of the *MHC class II* region (described in the following text), no large‐scale structural differences across the assembled haplotypes were identified, using either multiple sequence alignment (Figure S2) or pairwise sequence alignment (Figure S3) approaches. To assess the density of genetic variation across the *MHC*, we mapped the assemblies against the canonical PGF reference haplotype and quantified them in sliding windows. With the exception of the MHC *class II* region, the positioning (data not shown) and density (Table S5) of repeat elements were generally conserved between the assembled haplotypes, with total interspersed repeats accounting for 51%–52% of sequence content. Consistent with previous analyses,^9^, ^62^ we observed three peaks having up to 50 SNPs/kbp, centred around the *HLA‐A*, *HLA‐B+C*, and *HLA class II* genes respectively, and that structural diversity peaks in the *MHC class II* region (Figure 2). Overall numbers of detected polymorphism were similar across the assemblies, ranging from 12,106 to 13,841 detected SNPs per assembled haplotype and from 2,075 to 2,441 detected INDELs.

### 3.5 Analysis of *MHC* *class II* structures

The *class II* region is the most structurally variable region of the human *MHC*; it is characterized by four major haplotype structures that are defined by the genotype of the *HLA‐DRB1* gene and *HLA‐DRB3*, *−4* and *− 5* carrier status. In accordance with Trowsdale et al.,^20^ we refer to the four major categories of *MHC class II* structure as DR1 (DRB1*01 or *10, with no *DRB3‐5*), DR2 (DRB1*15 or *16, with *DRB5*), DR3 (DRB1*03, *11, *12, *13 or *14 with *DRB3*), DR4 (DRB1*04, *09 or *07 with *DRB4*). DR8 (DRB1*08, with no *DRB3‐*5), which was not targeted here and is likely derived from a DR3 haplotype (most likely DRB1*12), lacks the *DRB*6 pseudogene.

When compared with GRCh38, the haplotypes presented here represent the first complete assemblies of DR1 (*DRB1* only) and DR4 (*DRB1* + *DRB4*) *MHC class II* sequence structures. For the following analysis, the *MHC class II* region was defined as the region from *HLA‐DRA* to 20 kb downstream of *HLA‐DRB1*, and results are reported separately for the DRB3/4/5 carrier status‐defined haplotype classes.

#### 3.5.1 DR3 (*DRB1* + *DRB3*) haplotypes

**FIGURE 3:**
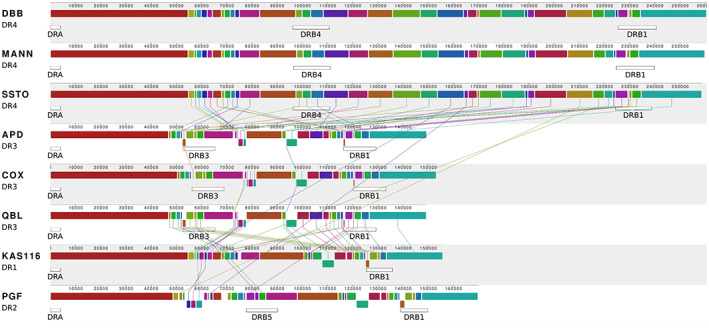
Multiple‐sequence alignment visualization of *MHC class II* haplotype structures. Shown is a comparison of the eight completed *MHC class II* region sequences. Colors represent respective sequence similarity across haplotypes. Segments drawn underneath the respective plots represent inversions. The plot was created using Mauve^53^ with parameter “seed weight” set to 22. Vertical lines connecting horizontally aligned homologous regions were edited for clarity.

**FIGURE 4:**
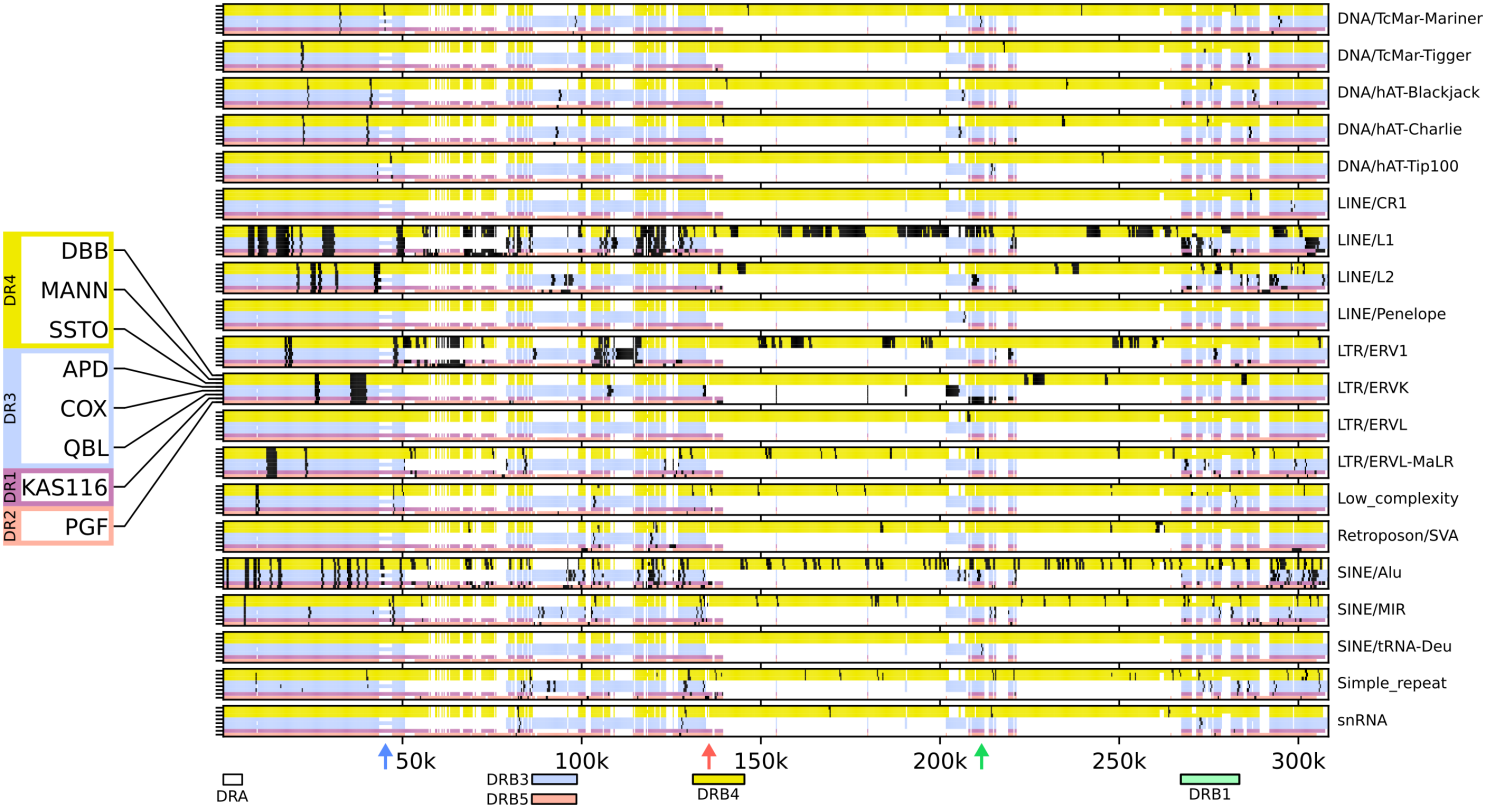
Repeat elements within the *MHC class II* region. Shown are positions of repeat elements identified in the *MHC class II* region of each of the eight completed haplotypes. The haplotypes are colored by their broad grouping: (yellow) DR4, (blue) DR3, (purple) DR1, (red) DR2. White indicates gaps relative to the longest sequence alignment. Black shading indicates locations where the respective repeat element was identified. The multiple sequence alignment was generated using mafft.^57^ Repeat elements were identified using RepeatMasker,^56^ where the sequences were divided into 300 bp non‐overlapping windows and those windows having any overlap with a repeat element were counted as containing a repeat. Red and green arrows indicate the positions of *HLA‐DRB6* and ERV that are components of the segment distinguishing DR1 from DR4; a blue arrow, the position of the identified small structural variant that is not in strict LD with any specific *MHC class II* haplotype.

The DR3 haplotype structure is carried by the APD, COX, and QBL cell lines. The assembled *MHC class II* haplotypes of APD and QBL are 149.5 and 149.4 kbp in length, respectively, compared with the previously characterized 153.4 kbp for the haplotype from COX. Both multiple sequence and pairwise alignments confirmed the structural homology of the assembled *DRB3* containing haplotypes (Figures 3 and S3). Of note, the small difference in *MHC class II* haplotype length within the DR3 group is largely attributable to an ~3.5 kbp sequence segment carried by COX (approximate position: 38 kbp downstream of *HLA‐DRA*; Figure 4, blue arrow) that is shared with the DR4 group and KAS116 (DR1) haplotypes, but absent from the other DR3 haplotypes. We also identified an ~4 kb inversion (relative to the DR4 group and KAS116 haplotypes) carried by the DR3 group and PGF (DR2) haplotypes, located approximately 80 and 60 kbp downstream of *HLA‐DRA* in COX and (in its reverse‐complemented form) in SSTO, respectively. The existence of this inversion was confirmed with minimap2.^43^ Interspersed repeats accounted for approximately 57% of sequence content (Table S5) in the *MHC class II* region of the DR3 group; the positions of repeat elements are generally conserved across the three DR3 haplotypes (Figure 4).

#### 3.5.2 DR4 (*DRB1* + *DRB4*) haplotypes

The DR4 haplotype structure is carried by the DBB, MANN, and SSTO cell lines. The assembled *MHC class II* haplotypes of the DR4 group range in length from 258.8 to 260.7 kbp; of note, GRCh38 does not include a fully resolved *MHC class II* haplotype of the DR4 group. We used both multiple sequence and pairwise alignment to investigate the extent of structural homology within the DR4 group and found no large structural variants (Figures 3 and S3). Repeat element positions are highly conserved across the DR4 haplotypes. The exceptions are retrotransposon/SVA and LINE1 insertions upstream of *HLA‐DRB1* in DBB and SSTO, and LINE1 insertions in the *HLA‐DRB1* of DBB and MANN (Figure 4), which characterize minor structural differences between the haplotypes. At approximately 63%, the interspersed repeat content of the DR4 group is higher than that of any other group (Table S5). Repeat elements also account for a considerable fraction of the sequence that distinguishes the DR4 group from other *MHC class II* sequences (Figure 4).

#### 3.5.3 DR2 (*DRB1* + *DRB5*) haplotypes

The DR2 haplotype structure is carried by PGF, which was used for the canonical chromosome 6 reference *MHC* haplotype of GRCh38. The *MHC class II* region of PGF has a length of 169.9 kb; as PGF is the only representative of the DR2 group, no within‐group structural variation analysis was carried out. Interspersed repeats account for 62.6% of the *MHC class II* sequence content of PGF (Table S5). High density of repeat elements (in particular LINE/L1 and LTR/ERV1) is also found in the *class II* sequence stretches of PGF, and do not align to any of the other assembled haplotypes (Figure 4).

#### 3.5.4 DR1 (*DRB1* only) haplotype

The DR1 haplotype structure is carried by KAS116, whereas GRCh38 contains no representative of this haplotype structure. The *MHC class II* haplotype of KAS116 has a length of 156.0 kbp. As KAS116 is the only representative of the DR1 group, no within‐group structural variation analysis could be carried out. Multiple and pairwise sequence alignments were computed to compare the haplotype of KAS116 to the other *MHC class II* haplotype groups (Figures 3 and S3). The DR1 haplotype is most similar to the DR4 haplotypes, where KAS116 differs by replacement of an approximately 120 kbp segment between *HLA‐DRB4* and *HLA‐DRB1,* with an approximately 20 kbp segment (Figure S3). The segment includes a copy of *HLA‐DRB6* at the 3′ end, followed by a 7.5 kb ERV sequence (Figure 4, red and green arrows). Interspersed repeats account for 61.5% of the *MHC class II* sequence content of KAS116 (Table S5).

### 3.6 Improvement of short‐read mapping experiment with newly assembled *MHC* haplotypes

To investigate the potential benefits for mapping whole‐genome short‐read sequencing data conferred by using the fully resolved *MHC* haplotypes presented here instead of the incomplete versions currently part of GRCh38, we performed a comparative mapping experiment. Whole‐genome Illumina sequencing reads from seven samples (two samples homozygous for a PGF‐like *MHC class II* structure; two samples homozygous for an APD‐like *MHC class II* structure; and three samples homozygous for the DR1 *MHC class II* structure) were aligned to a standard version of the GRCh38 reference genome (containing incompletely resolved *MHC* “alt_ref” contigs) and to an improved version of GRCh38 containing the complete assemblies presented here added (see Section 2). We observed only a small change in the total (whole‐genome) number of mapped reads due to the inclusion of the improved *MHC* reference sequences (Table 4). However, the number of reads recruited to the full‐length *MHC* contigs as part of “proper” read pairs (that is, with correct read orientations and with an insert size deemed plausible by the short‐read mapper BWA) increased by 0.06%–0.49% (Table 4). The largest effects were observed for sample NA10847 (0.49% increase), which carries a DR1 *MHC class II* structure (not represented in GRCh38), and for sample NA20861 (0.31%), which carries an APD‐like *MHC class II* structure (representing the least‐complete GRCh38 *MHC* “alt_ref” contig; 2.5 Mbp missing bases; Table S4).

**TABLE 4:** Comparative short‐read mapping experiment.

Sample ID	Sample MHC class II HLA types (homozygous)	Most similar to reference cell line	Mapping target	Reads aligned (“proper pairs”) to the MHC	Total aligned reads (whole‐genome)
NA20861	DQB1*06:*03*/DRB1*13:*01*	APD (DQB1*06:*03*:*01*:*01*/DRB1*13:*01*:*01*:*02*)	GRCh38	1.228.862	715.427.257
			GRCh38 + complete MHC	1.232.620	715.427.256
			Difference	0,31%	0,00%
HG04206	DQB1*06:*03*/DRB1*13:*01*	APD (DQB1*06:*03*:*01*:*01*/DRB1*13:*01*:*01*:*02*)	GRCh38	1.446.267	854.825.218
			GRCh38 + complete MHC	1.449.587	854.825.265
			Difference	0,23%	0,00%
NA10847	DQB1*05:*01*/DRB1*01:*01*	KAS116 (DQB1*05:*01*:*01*:*02*/DRB1*01:*01*:*01*:*01*)	GRCh38	1.295.561	748.531.837
			GRCh38 + complete MHC	1.301.847	748.531.552
			Difference	0,49%	0,00%
NA19755	DQB1*04:*01*/DRB1*08:*02*	KAS116 (no DRB3/4/5)	GRCh38	1.192.504	702.458.873
			GRCh38 + complete MHC	1.194.262	702.458.922
			Difference	0,15%	0,00%
HG02048	DQB1*05:*01*/DRB1*10:*01*	KAS116 (no DRB3/4/5)	GRCh38	1.264.765	732.313.229
			GRCh38 + complete MHC	1.267.701	732.313.264
			Difference	0,23%	0,00%
HG00135	DQB1*06:*02*/DRB1*15:*01*	PGF (DQB1*06:*02*:*01*:*01*/DRB1*15:*01*:*01*:*01*)	GRCh38	314.505	212.840.164
			GRCh38 + complete MHC	314.835	212.840.151
			Difference	0,10%	0,00%
NA11881	DQB1*06:*02*/DRB1*15:*01*	PGF (DQB1*06:*02*:*01*:*01*/DRB1*15:*01*:*01*:*01*)	GRCh38	1.315.963	753.208.749
			GRCh38 + complete MHC	1.316.719	753.208.789
			Difference	0,06%	0,00%

*Note*: Comparison of whole‐genome short‐read mapping with and without completed MHC haplotypes.

## 4 DISCUSSION

To improve the scope and utility of reference data for population‐scale immunogenomics, we have assembled and resolved the structure and sequence of five *MHC* reference haplotypes. This work completed five targets of the “MHC haplotype Project”^37^ that currently form part of the human reference genome. In targeting the same cell lines present in the GRCh38 human reference genome build, we increased the number of fully resolved *MHC* haplotypes from two to seven. Importantly, we include the first fully resolved *MHC class II* sequences representing the DR4 *MHC* haplotype group, which is 89–91 kbp longer than the PGF sequence that forms the baseline reference for GRCh38. In addition, our analysis of cell line KAS116 provides a structurally resolved reference haplotype for the DR1 *MHC class II* structure that is not yet represented in GRCh38. When combined with the complete PGF (DR2) and COX (DR3) reference haplotypes from GRCh38, the sequences presented here provide a comprehensive reference panel covering the major *MHC class II* haplotype structures.

Despite progress in long‐read sequencing technologies, and their combination with other technologies, fully resolving complex repeat structures of the *MHC*, in for example the *MHC class II* or *C4* regions, remains a challenge.^63^ Here we employed a hybrid strategy, integrating ultra‐long Nanopore sequencing data, PacBio HiFi sequencing, previously assembled, highly accurate short contigs from targeted sequencing, and Illumina whole‐genome sequencing data. For assembly we devised custom bioinformatics pipelines, where the long‐read data provided structural integrity, the contigs provided sequence accuracy, and the whole‐genome reads were vital for haplotype polishing. The accuracy of the assembled sequences is supported by multiple lines of evidence, including (i) structural consistency between available GRCh38 assembly fragments and the assemblies we produced; (ii) internal consistency within the different *MHC class II* haplotype groups; and (iii) comparable rates of genetic variation relative to the canonical PGF haplotype between the haplotypes assembled here and the other complete *MHC* reference haplotype part of GRCh38, COX (Table  3). Future goals include targeting representative DR8 haplotypes, which also lack the *DRB3*, *4* or *5* genes, to determine the ancestral relationship with DR3.

The availability of a comprehensive set of haplotype sequences enabled us to assess variation across the major *MHC class II* structures. Specifically, we ruled out the presence of any previously uncharacterized large‐scale structural variants, and showed that the DR1 *MHC class II* haplotype structure is most closely related to that of the DR4 haplotype. Interestingly, we also identified a small structural variant in the *MHC class II* region that is not in strict LD with any specific *MHC class II* haplotype class (Figure 4, blue arrow). Although we did not carry out a full analysis of the assembled *MHC class II* sequences, we have demonstrated that the density of repeat elements increases in the *MHC class II* region compared with the rest of the *MHC* (Table S5), that it varies between *MHC class II* haplotype groups, and that repeat elements are often found in the group‐exclusive sequence regions that differentiate between the *MHC class II* haplotype groups. The latter observation suggests a role for the repeat elements in the divergence and/or maintenance of separation between the different haplotypes.^21^ Our findings confirm and expand recent detailed analyses of the *MHC* haplotypes.^21^, ^64^

As a first step towards measuring how improved *MHC* reference assemblies can contribute to improved read mapping, and sequence and structure variant determination throughout the *MHC* genomic region, we performed a comparative short‐read mapping experiment. This experiment showed that our improved assemblies enable improvements in the recruitment of “proper pair” read alignments to full‐length *MHC* reference contigs. As expected, the largest effects were observed for the samples with *MHC class II* structures that were unfinished in GRCh38. Further improvements are expected by increasing the representation of *MHC* haplotypes of non‐European ancestry, which could complement ongoing pan‐genomic assembly efforts,^65^, ^66^ and by the further development of graph‐based approaches integrating information across reference sequences and haplotypes.^27^, ^33^, ^34^, ^67^, ^68^, ^69^, ^70^ Efforts along both directions are currently under way and we predict significant improvements to accessibility of immunogenetic variation and its phenotypic impacts in the near future.

## AUTHOR CONTRIBUTIONS

Alexander T. Dilthey and Paul J. Norman conceptualized and designed the study. Torsten Houwaart, Stephan Scholz, Nicholas R. Pollock, Birgit Henrich, Karl Köhrer, Peter Parham, Lisbeth A. Guethlein gave input into the study design. Torsten Houwaart, Stephan Scholz, Nicholas R. Pollock, William H. Palmer, Duyen B. Le performed data analysis. Torsten Houwaart and Stephan Scholz developed software for the analysis of sequencing data. Peter Parham provided samples. William H. Palmer, Katherine M. Kichula, Daniel Strelow, Dana Belick, Lisanna Hülse, Tobias Lautwein, Tassilo E. Wollenweber, Thorsten Wachtmeister performed experiments and generated data. Torsten Houwaart, Stephan Scholz, Nicholas R. Pollock, Peter Parham, Lisbeth A. Guethlein, Paul J. Norman and Alexander T. Dilthey wrote the first draft of the manuscript. All authors reviewed and approved the final manuscript.

## CONFLICT OF INTEREST STATEMENT

Alexander T. Dilthey is a co‐founder of Peptide Groove, LLP, a company that commercializes statistical HLA typing approaches. The other authors declare no conflicting interests.

## Supporting information


**Supplementary Figure S1. C4 genotypes of assembled *MHC* sequences and the complete GF and COX *MHC* reference haplotypes from GRCh38.** HERV = human endogenous retrovirus. Visual display adapted from Sekar et al^26^

**Supplementary Figure S2. Mauve multiple‐sequence alignment of the assembled *MHC* sequences and the complete PGF and COX *MHC* reference haplotypes from GRCh38**. The plot is based on an alignment generated with the ‘seed weight’ parameter set to 22.

**Supplementary Figure S3. Dot plots visualizing the haplotype structures of the assembled *MHC* sequences and the complete PGF and COX *MHC* reference haplotypes from GRCh38.** Each plot consists of two triangular panels, showing visualizations of whole‐*MHC* (upper‐left half) and *MHC class II* (lower‐right half) structures of the corresponding haplotypes (see labels on the X and Y axes), also showing the lengths of the visualized sequences. Panel (a) shows a comparison between one representative of each of the four major *MHC class II* haplotype structures; panel (b) shows a comparison between the sequence structures of the DR3 group of *MHC* haplotypes; panel (c), a comparison between the sequence structures of the DR4 *MHC* haplotype group. Shaded bars in the *MHC class II*‐specific panels indicate the positions of the *HLA‐DRB3*, *‐DRB4*, and *‐DRB5* genes. The plots were generated with the nucmer (parameters ‐‐maxmatch ‐‐nosimplify ‐‐mincluster 300), mummerplot^46^ and gnuplot programs.

**Supplementary File S1.** Full‐length HLA gene sequences of the assembled haplotypes for 18 analyzed HLA loci (Supplementary Table S3).

**Supplementary Table S1. Data and assembly generation summary.** Illumina and Nanopore sequencing data summary for the sequenced cell lines, number of polishing rounds performed during the assembly process (see Methods), and BioSample and GenBank IDs.
**Supplementary Table S2. Data generation details.** Illumina and Nanopore sequencing runs performed on the different cell lines, including details on the utilized DNA extraction, library preparation and sequencing protocols.
**Supplementary Table S3. Comparison to reference HLA types.** Comparison on a per‐locus basis between the assembled haplotype sequences and reference HLA types from IPD‐IMGT/HLA. “Edit distance assembly ↔ IMGT/HLA ref.” specifies the edit distance between the assembled haplotype and the reference HLA type (“IMGT/HLA reference”); “IMGT/HLA min. edit distance” specifies the IPD‐IMGT/HLA reference allele that most closely matches the assembled haplotype, if not identical to the reference HLA type. For incompletely defined alleles, “Added bases” specifies the number of bases that could be completed based on the assembled haplotype.
**Supplementary Table S4. Assembly comparison to GRCh38 and Norman et al. (2017).** Comparison between the haplotype assemblies generated here and earlier assemblies of the same haplotypes from GRCh38 and the scaffold dataset released by.^9^ “Undetermined nucleotides” were quantified by counting the number of ‘N' characters in the corresponding assembly version; “missing bases” were quantified by mapping the earlier assemblies of the considered haplotypes against the versions presented here, retaining only unique alignments, and counting the number of bases with 0 coverage from the earlier‐assembly alignments.
**Supplementary Table S5. Interspersed repeats content.** Proportion of assembled *MHC* sequences marked as interspersed repeats by the RepeatMasker^56^ algorithm, reported separately for the complete *MHC* and the *MHC class II* subregion.

## Data Availability

Whole‐genome Nanopore, Illumina and PacBio HiFi sequencing data generated as part of this study and the generated *MHC* assemblies were submitted to NCBI BioProject PRJNA764575. The whole‐genome sequencing data were filtered to contain only reads mapping to the assembled *MHC* sequences prior to submission. The generated assemblies were also submitted to GenBank; GenBank accessions are listed in Table 1. Data generated by^9^ that were used as part of the assembly process described here are also publicly available; the corresponding BioSample IDs are listed in Table S2. The Python source code used for annotation of the assembled *MHC* sequences is available via PyPI (https://pypi.org/project/MHC-Annotation/). A separate publication describing the annotation method is currently under preparation.
