# Supplementary figures and images for: Complete sequences of six major histocompatibility complex haplotypes, including all the major MHC class II structures
Source: HLA. 2023 Mar 18;102(1):28–43. doi: 10.1111/tan.15020 (PMC10986641; doi:10.1111/tan.15020)

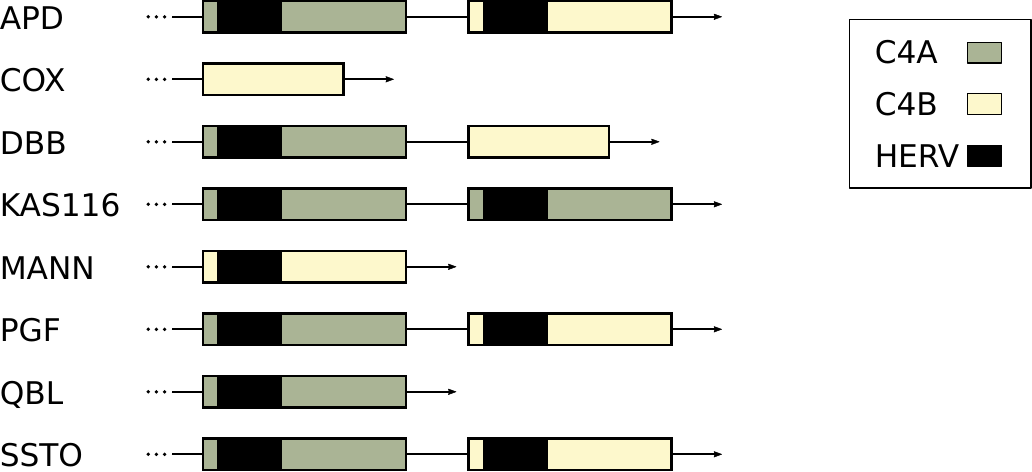

Supplement: Supplementary file 1 — Supplementary Figure S1. C4 genotypes of assembled MHC sequences and the complete GF and COX MHC reference haplotypes from GRCh38. HERV = human endogenous retrovirus. Visual display adapted from Sekar et al 26 [file TAN-102-28-s001.tif]

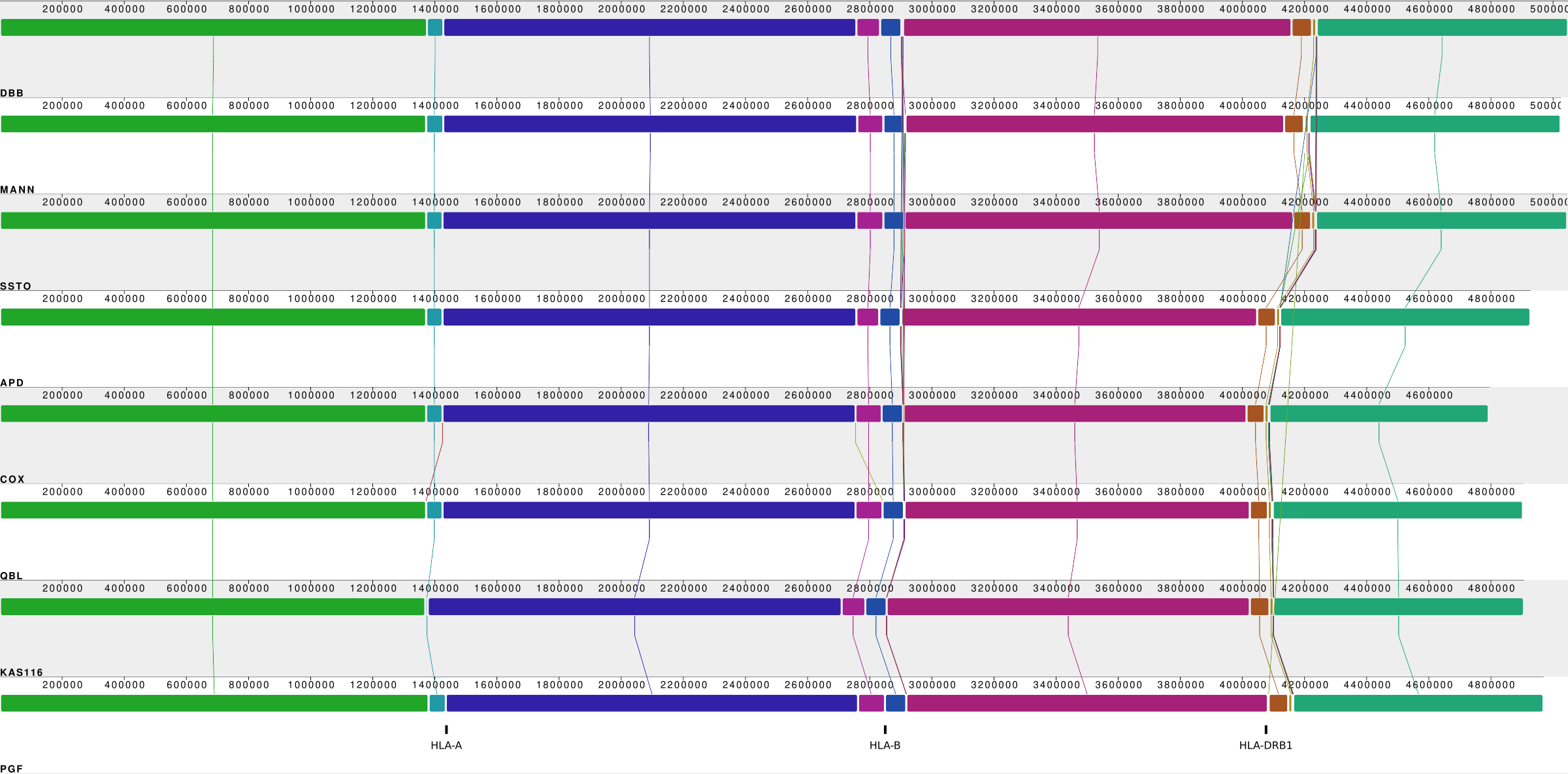

Supplement: Supplementary file 2 — Supplementary Figure S2. Mauve multiple‐sequence alignment of the assembled MHC sequences and the complete PGF and COX MHC reference haplotypes from GRCh38. The plot is based on an alignment generated with the ‘seed weight’ parameter set to 22. [file TAN-102-28-s003.tiff]

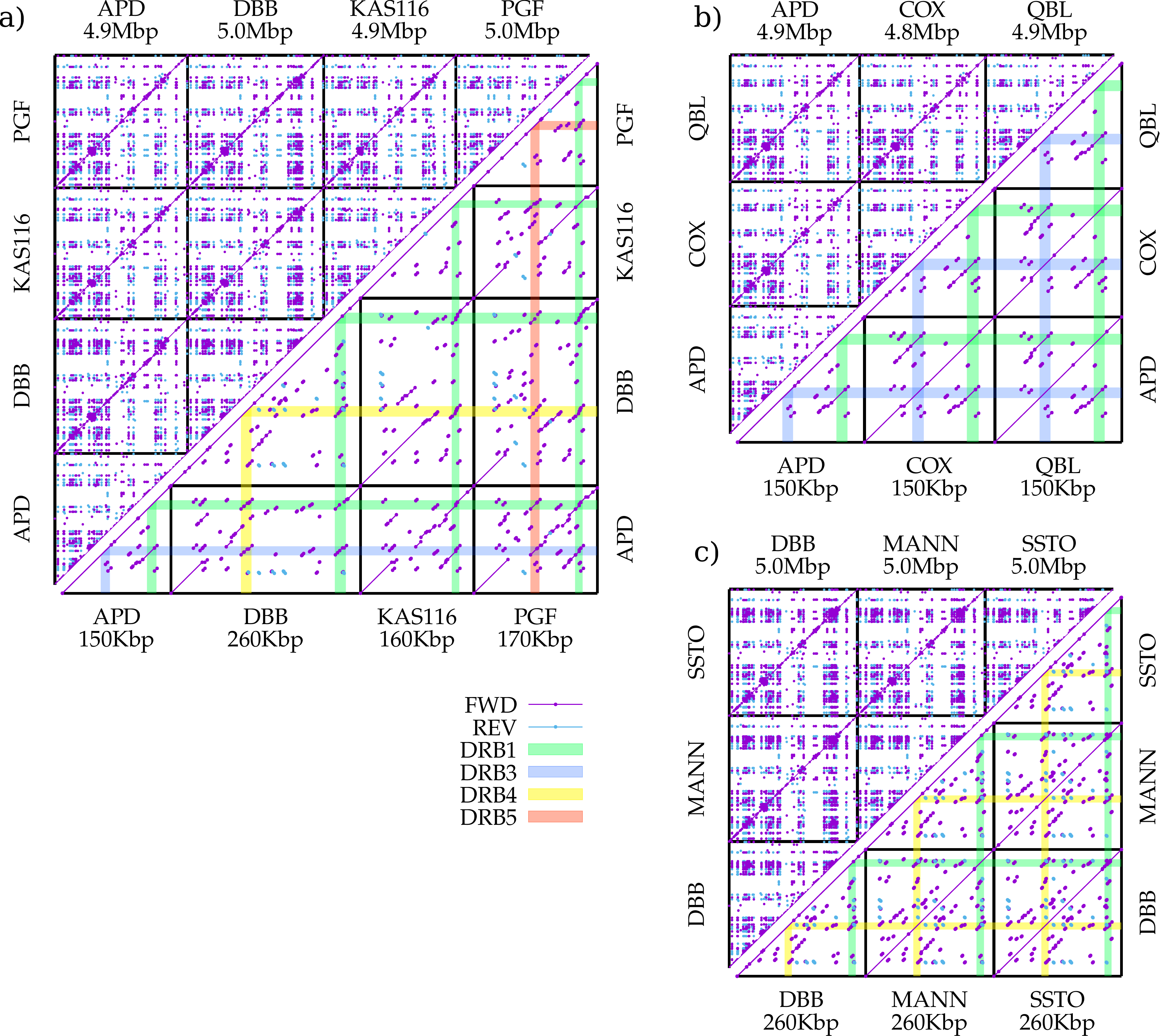

Supplement: Supplementary file 3 — Supplementary Figure S3. Dot plots visualizing the haplotype structures of the assembled MHC sequences and the complete PGF and COX MHC reference haplotypes from GRCh38. Each plot consists of two triangular panels, showing visualizations of whole‐MHC (upper‐left half) and MHC class II (lower‐right half) structures of the corresponding haplotypes (see labels on the X and Y axes), also showing the lengths of the visualized sequences. Panel (a) shows a comparison between one representative of each of the four major MHC class II haplotype structures; panel (b) shows a comparison between the sequence structures of the DR3 group of MHC haplotypes; panel (c), a comparison between the sequence structures of the DR4 MHC haplotype group. Shaded bars in the MHC class II‐specific panels indicate the positions of the HLA‐DRB3, ‐DRB4, and ‐DRB5 genes. The plots were generated with the nucmer (parameters ‐‐maxmatch ‐‐nosimplify ‐‐mincluster 300), mummerplot 46 and gnuplot programs. [file TAN-102-28-s004.tif]
