# Supplementary material for: Complete sequences of six major histocompatibility complex haplotypes, including all the major MHC class II structures
Source: HLA. 2023 Mar 18;102(1):28–43. doi: 10.1111/tan.15020 (PMC10986641; doi:10.1111/tan.15020)
Supplement: Supplementary file 4 — Supplementary File S1. Full‐length HLA gene sequences of the assembled haplotypes for 18 analyzed HLA loci (Supplementary Table S3). [file TAN-102-28-s002.docx]

>HLA:HLA00001 A*01:01:01:01

CAGGAGCAGAGGGGTCAGGGCGAAGTCCCAGGGCCCCAGGCGTGGCTCTCAGGGTCTCAG

GCCCCGAAGGCGGTGTATGGATTGGGGAGTCCCAGCCTTGGGGATTCCCCAACTCCGCAG

TTTCTTTTCTCCCTCTCCCAACCTACGTAGGGTCCTTCATCCTGGATACTCACGACGCGG

ACCCAGTTCTCACTCCCATTGGGTGTCGGGTTTCCAGAGAAGCCAATCAGTGTCGTCGCG

GTCGCTGTTCTAAAGTCCGCACGCACCCACCGGGACTCAGATTCTCCCCAGACGCCGAGG

ATGGCCGTCATGGCGCCCCGAACCCTCCTCCTGCTACTCTCGGGGGCCCTGGCCCTGACC

CAGACCTGGGCGGGTGAGTGCGGGGTCGGGAGGGAAACCGCCTCTGCGGGGAGAAGCAAG

GGGCCCTCCTGGCGGGGGCGCAGGACCGGGGGAGCCGCGCCGGGAGGAGGGTCGGGCAGG

TCTCAGCCACTGCTCGCCCCCAGGCTCCCACTCCATGAGGTATTTCTTCACATCCGTGTC

CCGGCCCGGCCGCGGGGAGCCCCGCTTCATCGCCGTGGGCTACGTGGACGACACGCAGTT

CGTGCGGTTCGACAGCGACGCCGCGAGCCAGAAGATGGAGCCGCGGGCGCCGTGGATAGA

GCAGGAGGGGCCGGAGTATTGGGACCAGGAGACACGGAATATGAAGGCCCACTCACAGAC

TGACCGAGCGAACCTGGGGACCCTGCGCGGCTACTACAACCAGAGCGAGGACGGTGAGTG

ACCCCGGCCCGGGGCGCAGGTCACGACCCCTCATCCCCCACGGACGGGCCAGGTCGCCCA

CAGTCTCCGGGTCCGAGATCCACCCCGAAGCCGCGGGACTCCGAGACCCTTGTCCCGGGA

GAGGCCCAGGCGCCTTTACCCGGTTTCATTTTCAGTTTAGGCCAAAAATCCCCCCGGGTT

GGTCGGGGCGGGGCGGGGCTCGGGGGACTGGGCTGACCGCGGGGTCGGGGCCAGGTTCTC

ACACCATCCAGATAATGTATGGCTGCGACGTGGGGCCGGACGGGCGCTTCCTCCGCGGGT

ACCGGCAGGACGCCTACGACGGCAAGGATTACATCGCCCTGAACGAGGACCTGCGCTCTT

GGACCGCGGCGGACATGGCAGCTCAGATCACCAAGCGCAAGTGGGAGGCGGTCCATGCGG

CGGAGCAGCGGAGAGTCTACCTGGAGGGCCGGTGCGTGGACGGGCTCCGCAGATACCTGG

AGAACGGGAAGGAGACGCTGCAGCGCACGGGTACCAGGGGCCACGGGGCGCCTCCCTGAT

CGCCTATAGATCTCCCGGGCTGGCCTCCCACAAGGAGGGGAGACAATTGGGACCAACACT

AGAATATCACCCTCCCTCTGGTCCTGAGGGAGAGGAATCCTCCTGGGTTTCCAGATCCTG

TACCAGAGAGTGACTCTGAGGTTCCGCCCTGCTCTCTGACACAATTAAGGGATAAAATCT

CTGAAGGAGTGACGGGAAGACGATCCCTCGAATACTGATGAGTGGTTCCCTTTGACACCG

GCAGCAGCCTTGGGCCCGTGACTTTTCCTCTCAGGCCTTGTTCTCTGCTTCACACTCAAT

GTGTGTGGGGGTCTGAGTCCAGCACTTCTGAGTCTCTCAGCCTCCACTCAGGTCAGGACC

AGAAGTCGCTGTTCCCTTCTCAGGGAATAGAAGATTATCCCAGGTGCCTGTGTCCAGGCT

GGTGTCTGGGTTCTGTGCTCTCTTCCCCATCCCGGGTGTCCTGTCCATTCTCAAGATGGC

CACATGCGTGCTGGTGGAGTGTCCCATGACAGATGCAAAATGCCTGAATTTTCTGACTCT

TCCCGTCAGACCCCCCCAAGACACATATGACCCACCACCCCATCTCTGACCATGAGGCCA

CCCTGAGGTGCTGGGCCCTGGGCTTCTACCCTGCGGAGATCACACTGACCTGGCAGCGGG

ATGGGGAGGACCAGACCCAGGACACGGAGCTCGTGGAGACCAGGCCTGCAGGGGATGGAA

CCTTCCAGAAGTGGGCGGCTGTGGTGGTGCCTTCTGGAGAGGAGCAGAGATACACCTGCC

ATGTGCAGCATGAGGGTCTGCCCAAGCCCCTCACCCTGAGATGGGGTAAGGAGGGAGATG

GGGGTGTCATGTCTCTTAGGGAAAGCAGGAGCCTCTCTGGAGACCTTTAGCAGGGTCAGG

GCCCCTCACCTTCCCCTCTTTTCCCAGAGCTGTCTTCCCAGCCCACCATCCCCATCGTGG

GCATCATTGCTGGCCTGGTTCTCCTTGGAGCTGTGATCACTGGAGCTGTGGTCGCTGCCG

TGATGTGGAGGAGGAAGAGCTCAGGTGGAGAAGGGGTGAAGGGTGGGGTCTGAGATTTCT

TGTCTCACTGAGGGTTCCAAGCCCCAGCTAGAAATGTGCCCTGTCTCATTACTGGGAAGC

ACCTTCCACAATCATGGGCCGACCCAGCCTGGGCCCTGTGTGCCAGCACTTACTCTTTTG

TAAAGCACCTGTTAAAATGAAGGACAGATTTATCACCTTGATTACGGCGGTGATGGGACC

TGATCCCAGCAGTCACAAGTCACAGGGGAAGGTCCCTGAGGACAGACCTCAGGAGGGCTA

TTGGTCCAGGACCCACACCTGCTTTCTTCATGTTTCCTGATCCCGCCCTGGGTCTGCAGT

CACACATTTCTGGAAACTTCTCTGGGGTCCAAGACTAGGAGGTTCCTCTAGGACCTTAAG

GCCCTGGCTCCTTTCTGGTATCTCACAGGACATTTTCTTCCCACAGATAGAAAAGGAGGG

AGTTACACTCAGGCTGCAAGTAAGTATGAAGGAGGCTGATGCCTGAGGTCCTTGGGATAT

TGTGTTTGGGAGCCCATGGGGGAGCTCACCCACCCCACAATTCCTCCTCTAGCCACATCT

TCTGTGGGATCTGACCAGGTTCTGTTTTTGTTCTACCCCAGGCAGTGACAGTGCCCAGGG

CTCTGATGTGTCTCTCACAGCTTGTAAAGGTGAGAGCTTGGAGGGCCTGATGTGTGTTGG

GTGTTGGGTGGAACAGTGGACACAGCTGTGCTATGGGGTTTCTTTGCGTTGGATGTATTG

AGCATGCGATGGGCTGTTTAAGGTGTGACCCCTCACTGTGATGGATATGAATTTGTTCAT

GAATATTTTTTTCTATAGTGTGAGACAGCTGCCTTGTGTGGGACTGAGAGGCAAGAGTTG

TTCCTGCCCTTCCCTTTGTGACTTGAAGAACCCTGACTTTGTTTCTGCAAAGGCACCTGC

ATGTGTCTGTGTTCGTGTAGGCATAATGTGAGGAGGTGGGGAGAGCACCCCACCCCCATG

TCCACCATGACCCTCTTCCCACGCTGACCTGTGCTCCCTCTCCAATCATCTTTCCTGTTC

CAGAGAGGTGGGGCTGAGGTGTCTCCATCTCTGTCTCAACTTCATGGTGCACTGAGCTGT

AACTTCTTCCTTCCCTATTAAAA

>HLA:HLA00005 A*02:01:01:01

CAGAAGCAGAGGGGTCAGGGCGAAGTCCCAGGGCCCCAGGCGTGGCTCTCAGGGTCTCAG

GCCCCGAAGGCGGTGTATGGATTGGGGAGTCCCAGCCTTGGGGATTCCCCAACTCCGCAG

TTTCTTTTCTCCCTCTCCCAACCTATGTAGGGTCCTTCTTCCTGGATACTCACGACGCGG

ACCCAGTTCTCACTCCCATTGGGTGTCGGGTTTCCAGAGAAGCCAATCAGTGTCGTCGCG

GTCGCGGTTCTAAAGTCCGCACGCACCCACCGGGACTCAGATTCTCCCCAGACGCCGAGG

ATGGCCGTCATGGCGCCCCGAACCCTCGTCCTGCTACTCTCGGGGGCTCTGGCCCTGACC

CAGACCTGGGCGGGTGAGTGCGGGGTCGGGAGGGAAACGGCCTCTGTGGGGAGAAGCAAC

GGGCCCGCCTGGCGGGGGCGCAGGACCCGGGAAGCCGCGCCGGGAGGAGGGTCGGGCGGG

TCTCAGCCACTCCTCGTCCCCAGGCTCTCACTCCATGAGGTATTTCTTCACATCCGTGTC

CCGGCCCGGCCGCGGGGAGCCCCGCTTCATCGCAGTGGGCTACGTGGACGACACGCAGTT

CGTGCGGTTCGACAGCGACGCCGCGAGCCAGAGGATGGAGCCGCGGGCGCCGTGGATAGA

GCAGGAGGGTCCGGAGTATTGGGACGGGGAGACACGGAAAGTGAAGGCCCACTCACAGAC

TCACCGAGTGGACCTGGGGACCCTGCGCGGCTACTACAACCAGAGCGAGGCCGGTGAGTG

ACCCCGGCCCGGGGCGCAGGTCACGACCTCTCATCCCCCACGGACGGGCCAGGTCGCCCA

CAGTCTCCGGGTCCGAGATCCGCCCCGAAGCCGCGGGACCCCGAGACCCTTGCCCCGGGA

GAGGCCCAGGCGCCTTTACCCGGTTTCATTTTCAGTTTAGGCCAAAAATCCCCCCAGGTT

GGTCGGGGCGGGGCGGGGCTCGGGGGACCGGGCTGACCGCGGGGTCCGGGCCAGGTTCTC

ACACCGTCCAGAGGATGTATGGCTGCGACGTGGGGTCGGACTGGCGCTTCCTCCGCGGGT

ACCACCAGTACGCCTACGACGGCAAGGATTACATCGCCCTGAAAGAGGACCTGCGCTCTT

GGACCGCGGCGGACATGGCAGCTCAGACCACCAAGCACAAGTGGGAGGCGGCCCATGTGG

CGGAGCAGTTGAGAGCCTACCTGGAGGGCACGTGCGTGGAGTGGCTCCGCAGATACCTGG

AGAACGGGAAGGAGACGCTGCAGCGCACGGGTACCAGGGGCCACGGGGCGCCTCCCTGAT

CGCCTGTAGATCTCCCGGGCTGGCCTCCCACAAGGAGGGGAGACAATTGGGACCAACACT

AGAATATCGCCCTCCCTCTGGTCCTGAGGGAGAGGAATCCTCCTGGGTTTCCAGATCCTG

TACCAGAGAGTGACTCTGAGGTTCCGCCCTGCTCTCTGACACAATTAAGGGATAAAATCT

CTGAAGGAATGACGGGAAGACGATCCCTCGAATACTGATGAGTGGTTCCCTTTGACACAC

ACAGGCAGCAGCCTTGGGCCCGTGACTTTTCCTCTCAGGCCTTGTTCTCTGCTTCACACT

CAATGTGTGTGGGGGTCTGAGTCCAGCACTTCTGAGTCCTTCAGCCTCCACTCAGGTCAG

GACCAGAAGTCGCTGTTCCCTCTTCAGGGACTAGAATTTTCCACGGAATAGGAGATTATC

CCAGGTGCCTGTGTCCAGGCTGGTGTCTGGGTTCTGTGCTCCCTTCCCCATCCCAGGTGT

CCTGTCCATTCTCAAGATAGCCACATGTGTGCTGGAGGAGTGTCCCATGACAGATGCAAA

ATGCCTGAATGATCTGACTCTTCCTGACAGACGCCCCCAAAACGCATATGACTCACCACG

CTGTCTCTGACCATGAAGCCACCCTGAGGTGCTGGGCCCTGAGCTTCTACCCTGCGGAGA

TCACACTGACCTGGCAGCGGGATGGGGAGGACCAGACCCAGGACACGGAGCTCGTGGAGA

CCAGGCCTGCAGGGGATGGAACCTTCCAGAAGTGGGCGGCTGTGGTGGTGCCTTCTGGAC

AGGAGCAGAGATACACCTGCCATGTGCAGCATGAGGGTTTGCCCAAGCCCCTCACCCTGA

GATGGGGTAAGGAGGGAGACGGGGGTGTCATGTCTTTTAGGGAAAGCAGGAGCCTCTCTG

ACCTTTAGCAGGGTCAGGGCCCCTCACCTTCCCCTCTTTTCCCAGAGCCGTCTTCCCAGC

CCACCATCCCCATCGTGGGCATCATTGCTGGCCTGGTTCTCTTTGGAGCTGTGATCACTG

GAGCTGTGGTCGCTGCTGTGATGTGGAGGAGGAAGAGCTCAGGTGGGGAAGGGGTGAAGG

GTGGGTCTGAGATTTCTTGTCTCACTGAGGGTTCCAAGACCCAGGTAGAAGTGTGCCCTG

CCTCGTTACTGGGAAGCACCACCCACAATTATGGGCCTACCCAGCCTGGGCCCTGTGTGC

CAGCACTTACTCTTTTGTAAAGCACCTGTTAAAATGAAGGACAGATTTATCACCTTGATT

ACAGCGGTGATGGGACCTGATCCCAGCAGTCACAAGTCACAGGGGAAGGTCCCTGAGGAC

CTTCAGGAGGGCGGTTGGTCCAGGACCCACACCTGCTTTCTTCATGTTTCCTGATCCCGC

CCTGGGTCTGCAGTCACACATTTCTGGAAACTTCTCTGAGGTCCAAGACTTGGAGGTTCC

TCTAGGACCTTAAGGCCCTGACTCCTTTCTGGTATCTCACAGGACATTTTCTTCCCACAG

ATAGAAAAGGAGGGAGCTACTCTCAGGCTGCAAGTAAGTATGAAGGAGGCTGATGCCTGA

GGTCCTTGGGATATTGTGTTTGGGAGCCCATGGGGGAGCTCACCCACCCCACAATTCCTC

CTCTAGCCACATCTTCTGTGGGATCTGACCAGGTTCTGTTTTTGTTCTACCCCAGGCAGT

GACAGTGCCCAGGGCTCTGATGTGTCTCTCACAGCTTGTAAAGGTGAGAGCCTGGAGGGC

CTGATGTGTGTTGGGTGTTGGGCGGAACAGTGGACACAGCTGTGCTATGGGGTTTCTTTC

CATTGGATGTATTGAGCATGCGATGGGCTGTTTAAAGTGTGACCCCTCACTGTGACAGAT

ACGAATTTGTTCATGAATATTTTTTTCTATAGTGTGAGACAGCTGCCTTGTGTGGGACTG

AGAGGCAAGAGTTGTTCCTGCCCTTCCCTTTGTGACTTGAAGAACCCTGACTTTGTTTCT

GCAAAGGCACCTGCATGTGTCTGTGTTCGTGTAGGCATAATGTGAGGAGGTGGGGAGACC

ACCCCACCCCCATGTCCACCATGACCCTCTTCCCACGCTGACCTGTGCTCCCTCCCCAAT

CATCTTTCCTGTTCCAGAGAGGTGGGGCTGAGGTGTCTCCATCTCTGTCTCAACTTCATG

GTGCACTGAGCTGTAACTTCTTCCTTCCCTATTAAAA

>HLA:HLA00050 A*24:02:01:01

CAGAAGCAGAGGGGTCAGGGCGAAGTCCCAGGGCCCCAGGCGTGGCTCTCAGGGTCTCAG

GCCCCGAAGGCGGTGTATGGATTGGGGAGTCCCAGCCTTGGGGATTCCCCAACTCCGCAG

TTTCTTTTCTCCCTCTCCCAACCTATGTAGGGTCCTTCTTCCTGGATACTCACGACGCGG

ACCCAGTTCTCACTCCCATTGGGTGTCGGGTTTCCAGAGAAGCCAATCAGTGTCGTCGCG

GTCGCTGTTCTAAAGTCCGCACGCACCCACCGGGACTCAGATTCTCCCCAGACGCCGAGG

ATGGCCGTCATGGCGCCCCGAACCCTCGTCCTGCTACTCTCGGGGGCCCTGGCCCTGACC

CAGACCTGGGCAGGTGAGTGCGGGGTCGGGAGGGAAACGGCCTCTGCGGGGAGAAGCAAG

GGGCCCGCCTGGCGGGGGCGCAAGACCCGGGAAGCCGCGCCGGGAGGAGGGTCGGGCGGG

TCTCAGCCACTCCTCGTCCCCAGGCTCCCACTCCATGAGGTATTTCTCCACATCCGTGTC

CCGGCCCGGCCGCGGGGAGCCCCGCTTCATCGCCGTGGGCTACGTGGACGACACGCAGTT

CGTGCGGTTCGACAGCGACGCCGCGAGCCAGAGGATGGAGCCGCGGGCGCCGTGGATAGA

GCAGGAGGGGCCGGAGTATTGGGACGAGGAGACAGGGAAAGTGAAGGCCCACTCACAGAC

TGACCGAGAGAACCTGCGGATCGCGCTCCGCTACTACAACCAGAGCGAGGCCGGTGAGTG

ACCCCGGCCCGGGGCGCAGGTCACGACCCCTCATCCCCCACGGACGGGCCGGGTCGCCCA

CAGTCTCCGGGTCCGAGATCCACCCCGAAGCCGCGGGACCCCGAGACCCTTGCCCCGGGA

GAGGCCCAGGCGCCTTAACCCGGTTTCATTTTCAGTTTAGGCCAAAAATCCCCCCGGGTT

GGTCGGGGCCGGGCGGGGCTCGGGGGACTGGGCTGACCGCGGGGTCGGGGCCAGGTTCTC

ACACCCTCCAGATGATGTTTGGCTGCGACGTGGGGTCGGACGGGCGCTTCCTCCGCGGGT

ACCACCAGTACGCCTACGACGGCAAGGATTACATCGCCCTGAAAGAGGACCTGCGCTCTT

GGACCGCGGCGGACATGGCGGCTCAGATCACCAAGCGCAAGTGGGAGGCGGCCCATGTGG

CGGAGCAGCAGAGAGCCTACCTGGAGGGCACGTGCGTGGACGGGCTCCGCAGATACCTGG

AGAACGGGAAGGAGACGCTGCAGCGCACGGGTACCAGGGGCCACGGGGCGCCTACCTGAT

CGCCTGTAGGTCTCCCGGGCTGGCCTCCCACAAGGAGGGGAGACAATTGGGACCAACACT

AGAATATCGCCCTCCCTCTGGTCCTGAGGGAGAGGAATCCTCCTGGGTTTCCAGATCCTG

TACCAGAGAGTGACTCTGAGGTTCCGCCCTGCTCTCTGACACAATTAAGGGATAAAATCT

CTGACGGAATGACGGAAAGACGATCCCTCGAATACTGATGACTGGTTCCCTTTGACACCG

GCAGCAGCCTTGGGACCGTGACTTTTCCTCTCAGGCCTTGTTCTCTGCTTCACACTCAAT

GTGTGTGGGGGTCTGAGTCCAGCACTTCTGAGTCCCTCAGCCTCCACTCAGGTCAGGACC

AGAAGTCGCTGTTCCCTCCTCAGGGAATAGAAGATTATCCCAGGTGCCTGTGTCCAGGCT

GGTGTCTGGGTTCTGTGCTCTCTTCCCCATCCCGGGTGTCCTGTCCATTCTCAAGATGGC

CACATGCATGCTGGTGGAGTGTCCCATGACAGATGCAAAATGCCTGAATTTTCTGACTCT

TCCCGTCAGACCCCCCCAAGACACATATGACCCACCACCCCATCTCTGACCATGAGGCCA

CTCTGAGATGCTGGGCCCTGGGCTTCTACCCTGCGGAGATCACACTGACCTGGCAGCGGG

ATGGGGAGGACCAGACCCAGGACACGGAGCTTGTGGAGACCAGGCCTGCAGGGGATGGAA

CCTTCCAGAAGTGGGCAGCTGTGGTGGTACCTTCTGGAGAGGAGCAGAGATACACCTGCC

ATGTGCAGCATGAGGGTCTGCCCAAGCCCCTCACCCTGAGATGGGGTAAGGAGGGAGATG

GGGGTGTCATGTCTCTTAGGGAAAGCAGGAGCCTCTCTGGAGACCTTTAGCAGGGTCAGG

GCCCCTCACCTTCCCCTCTTTTCCCAGAGCCATCTTCCCAGCCCACCGTCCCCATCGTGG

GCATCATTGCTGGCCTGGTTCTCCTTGGAGCTGTGATCACTGGAGCTGTGGTCGCTGCTG

TGATGTGGAGGAGGAACAGCTCAGGTGGAGAAGGGGTGAAGGGTGGGGTCTGAGATTTCT

TGTCTCACTGAGGGTTCCAAGCCCCAGCTAGAAATGTGCCCTGTCTCATTACTGGGAAGC

ACCATCCACAATCATGGGCCGACCCAGCCTGGGCCCTGTGTGCCAGCACTTACTCTTTTG

TAAAGCACCTGTGACAATGAAGGACAGATTTATCACCTTGATTATGGCGGTGATGGGACC

TGATCCCAGCAGTCACAAGTCACAGGGGAAGGTCCCTGACGACAGATCTCAGGAGGGCGA

TTGGTCCAGGGCCCACATCTGCTTTCTTCATGTTTCCTGATCCTGCCCTGGGTCTGCAGT

CACACATTTCTGGAAACTTCTCTGGGGTCCAAGACTAGGAGGTTCCTCTAGGACCTTAAG

GCCCTGGCTCCTTTCTGGTATCTCACAGGACATTTTCTTCCCACAGATAGAAAAGGAGGG

AGCTACTCTCAGGCTGCAAGTAAGTATGAAGGAGGCTGATGCCTGAGGTCCTTGGGATAT

TGTGTTTGGGAGCCCATGGGGGAGCTCAACCACCCCACAATTCCTCCTCTAGCCACATCT

TCTGTGGGATCTGACCAGGTTCTGTTTTTGTTCTACCCCAGGCAGTGACAGTGCCCAGGG

CTCTGATGTGTCTCTCACAGCTTGTAAAGGTGAGAGCTTGGAGGGCCTGATGTGTGTTGG

GTGTTGGGCGGAACAGTGGACACAGCTGTGCTATGGGGTTTCTTTGCATTGGATGTATTG

AGCATGCGATGGGCTGTTTAAAGTGTGACCCCTCACTGTGACAGATATGAAGTTGTTCAT

GAATTTTTTTTCTATAGTGTGAGACAGCTGCCTTGTGTGGGACTGAGAGGCAAGAGTTGT

TCCTGCCCTTCCCTTTGTGACTTGAAGAACCCTGACTTTGTTTCTGCAAAGGCACCTGCA

TGTGTCTGTGTTCATGTAGGCATAATGTGAGGAGGTGGGGAGACCACCCCACCCCCATGT

CCACCATGACCCTCTTCCCACGCTGACCTGTGCTCCCTCCCCAATCATCTTTCCTGTTGC

AGAGAGGTGGGGCTGAGGTGTCTCCATCTCTGTCTCAACTTCATGGTGCACTGAGCTGTA

ACTTCTTCCTTCCCTATTAAAA

>HLA:HLA00073 A*26:01:01:01

CAGGAGCAGAGGGGTCAGGGCGAAGTCCCAGGGCCCCAGGCGTGGCTCTCAGGGTCTCAG

GCCCCGAAGGCGGTATATGGATTGGGGAGTCCCAGCCTTGGGGATTCCCCAACTCCGCAG

TTTCTTTTCTCCCTCTCCCAACCTATGTAGGGTCCTTCTTCCTGGATACTCACGACGCGG

ACCCAGTTCTCACTCCCATTGGGTGTCGGGTTTCCAGAGAAGCCAATCAGTGTCGTCGCG

GTCGCGGTTCTAAAGTCCGCACGCACCCACCGGGACTCAGATTCTCCCCAGACGCCGAGG

ATGGCCGTCATGGCGCCCCGAACCCTCGTCCTGCTACTCTCGGGGGCCCTGGCCCTGACC

CAGACCTGGGCGGGTGAGTGCGGGGTCGGGAGGGAAACGGCCTCTGTGGGGAGAAGCAAG

GGGCCCGCCCGGCGGGGGCGCAGGACCCGGGAAGCCGCGCCTGGAGGAGGGTCGGGCGGG

TCTCAGCCACTCCTCGCCCCCAGGCTCCCACTCCATGAGGTATTTCTACACCTCCGTGTC

CCGGCCCGGCCGCGGGGAGCCCCGCTTCATCGCCGTGGGCTACGTGGACGACACGCAGTT

CGTGCGGTTCGACAGCGACGCCGCGAGCCAGAGGATGGAGCCGCGGGCGCCGTGGATAGA

GCAGGAGGGGCCGGAGTATTGGGACCGGAACACACGGAATGTGAAGGCCCACTCACAGAC

TGACCGAGCGAACCTGGGGACCCTGCGCGGCTACTACAACCAGAGCGAGGACGGTGAGTG

ACCCCGGCCCGGGGCGCAGGTCACGACCCCTCATCCCCCACGGACGGGCCAGGTCGCCCA

CAGTCTCCGGGTCCGAGATCCGCCCCGAAGCCGCGGGACCCCGAGACCCTTGCCCCGGGA

GAGGCCCAGGCGCCTTTACCCGGTTTCATTTTCAGTTTAGGCCAAAAATCCCCCCGGGTT

GGTCGGGGCGGGGCGGGGCTCGGGGGACCGGGCTGACCTCGGGGTCCGGGCCAGGTTCTC

ACACCATCCAGAGGATGTATGGCTGCGACGTGGGGCCGGACGGGCGCTTCCTCCGCGGGT

ACCAGCAGGACGCTTACGACGGCAAGGATTACATCGCCCTGAACGAGGACCTGCGCTCTT

GGACCGCGGCGGACATGGCGGCTCAGATCACCCAGCGCAAGTGGGAGACGGCCCATGAGG

CGGAGCAGTGGAGAGCCTACCTGGAGGGCCGGTGCGTGGAGTGGCTCCGCAGATACCTGG

AGAACGGGAAGGAGACGCTGCAGCGCACGGGTACCAGGGGCCACGGGGCGCCTCCCTGAT

CGCCTGTAGATCTCCCGGGCTGGCCTCCCACAAGGAGGGGAGACAATTGGGACCAACACT

AGAATATCGCCCTCCCTCTGGTCCTGAGGGAGAGGAATCCTCCTGGGTTTCCAGATCCTG

TACCAGAGAGTGACTCTGAGGTTCCGCCCTGCTCTCTGACACAATTAAGGGATAAAATCT

CTGAAGGAATGACGGGAAGACGATCCCTCGAATACTGATGAGTGGTTCCCTTTGACACAC

ACCGGCAGCAGCCTTGGGCCCGTGACTTTTCCTCTCAGGCCTTGTTCTCTGCTTCACACT

CAATGTGTGTGGGGGTCTGAGTCCAGCACTTCTGAGTCCCTCAGCCTCCACTCAGGTCAG

GACCAGAAGTCGCTGTTCCCTCTTCAGGGACTAGAATTTTCCACGGAATAGGAGATTATC

CCAGGTGCCTGTGTCCAGGCTGGTGTCTGGGTTCTGTGCTCCCTTCCCCATCCCAGGTGT

CCTGTCCATTCTCAAGATAGCCACATGTGTGCTGGAGGAGTGTCCCATGACAGATGCAAA

ATGCCTGAATGTTCTGACTCTTCCTGACAGACGCCCCCAAGACGCATATGACTCACCACG

CTGTCTCTGACCATGAGGCCACCCTGAGGTGCTGGGCCCTGAGCTTCTACCCTGCGGAGA

TCACACTGACCTGGCAGCGGGATGGGGAGGACCAGACCCAGGACACGGAGCTCGTGGAGA

CCAGGCCTGCAGGGGATGGGACCTTCCAGAAGTGGGCGTCTGTGGTGGTGCCTTCTGGAC

AGGAGCAGAGATACACCTGCCATGTGCAGCATGAGGGTCTGCCCAAGCCCCTCACCCTGA

GATGGGGTAAGGAGGGAGACGGGGGTGTCATGTCTTTTAGGGAAAGCAGGAGCCTCTCTG

ACCTTTAGCAGGGTCAGGGCCCCTCACCTTCCCCTCTTTTCCCAGAGCCGTCTTCCCAGC

CCACCATCCCCATCGTGGGCATCATTGCTGGCCTGGTTCTCTTTGGAGCTGTGATCGCTG

GAGCTGTGGTCGCTGCTGTGATGTGGAGGAGGAAGAGCTCAGGTGGGGAAGGGATGAAGG

GTGGGTCTGAGATTTCTTGTCTCACTGAGGGTTCCAAGACCCAGGTAGAAGTGTGCCCTG

CCTCGTTACTGGGAAGCACCATCCACAATTATGAGCCTACCCAGCCTGGGCCCTGTGTGC

CAGCACTTACTCTTTTGTAAAGCACCTGTTAAAATGAAGGACAGATTTATCACCTTGATT

ACGGCGGTGATGGGACCTGATCCCAGCAGTCACAAGTCACAGGGGAAGGTCCCTGAGGAC

CTTCAGGAGGGCGGTTGGTCCAGGACCCACACCTGCTTTCTTCATGTTTCCTGATCCCGC

CCTGGGTCTGCAGTCACACATTTCTGGAAACTTCTCTGAGGTCCAAGACTTGGAGGTTCC

TCTAGGACCTTAAGGCCCTGGCTCCTTTCTGGTATCTCACAGGACATTTTCTTCCCACAG

ATAGAAAAGGAGGGAGCTACTCTCAGGCTGCAAGTAAGTATGAAGGAGGCTGATGCCTGA

GGTCCTTGGGATATTGTGTTTGGGAGCCCGTGGGGGAGCTCACCCACCCCACAATTCCTC

CTCTAGCCACATGTTCTGTGGGATCTGACCAGGTTCTGTTTTTGTCCTACCCCAGGCAGT

GACAGTGCCCAGGGCTCTGATATGTCTCTCACAGCTTGTAAAGGTGAGAGCCTGGAGGGC

CTGATGTGTGTTGGGTGTTGGGCGGAACAGTGGACGCAGCTGTGCTATGGGGTTTCTTTG

CATTGGATGTATTGAGCATGCGATGGGCTGTTTAAAGTGTGACTCCTCACTGTGACAGAT

ACGAATTTGTTCATGAATATTTTTTTCTATAGTGTGAGACAGCTGCCTTGTGTGGGACTG

AGAGGCAAGATTTGTTCCTGCCCTTCCCTTTGTGACTTGAAGAACCCTGACTTTGTTTCT

GCAAAGGCACCTGCATGTGTCTGTGTTCTTGTAGGCATAATGTGAGGAGGTGGGGAGACC

ACCCCACCCCCATGTCCACCATGACCCTCTTCCCACGCTGACCTGTGCTCCCTCCCCAAT

CATCTTTCCTGTTCCAGAGAGGTGGGGCTGAGGTGTCTCCATCTCTGCCTCAACTTCATG

GTGCACTGAGCTGTAACTTTTTCCTTCCCTATTAAAA

>HLA:HLA00086 A*29:02:01:01

CAGGAGCAGAGGGGTCAGGGCGAAGTCCCAGGGCCCCAGGCGTGGCTCTCAGGGTCTCAG

GCCCCGAAGGCGGTGTATGGATTGGGGAGTCCCAGCCTTGGGGATTCCCCAACTCCGCAG

TTTCTTTTCTCCCTCTGCCAACCTATGTAGGGTCCTTCTTCCTGGATACTCACGACGCGG

ACCCAGTTCTCACTCCCATTGGGTGTCGGGTTTCCAGAGAAGCCAATCAGTGTCGTCGCG

GTCGCGGTTCTAAAGTCCGCACGCACCCACCGGGACTCAGATTCTCCCCAGACGCCGAGG

ATGGCCGTCATGGCGCCCCGAACCCTCCTCCTGCTACTCTTGGGGGCCCTGGCCCTGACC

CAGACCTGGGCGGGTGAGTGCGGGGTCGTGGGGAAACCGCCTCTGCGGGGAGAAGCAAGG

GGCCCGCCCGGCGGGGACGCAGGACCCGGGTAGCCGCGCCGGGAGGAGGGTCGGGTGGGT

CTCAGCCACTCCTCGCCCCCAGGCTCCCACTCCATGAGGTATTTCACCACATCCGTGTCC

CGGCCCGGCCGCGGGGAGCCCCGCTTCATCGCCGTGGGCTACGTGGACGACACGCAGTTC

GTGCGGTTTGACAGCGACGCCGCGAGCCAGAGGATGGAGCCGCGGGCACCGTGGATAGAG

CAGGAGGGGCCGGAGTATTGGGACCTGCAGACACGGAATGTGAAGGCCCAGTCACAGACT

GACCGAGCGAACCTGGGGACCCTGCGCGGCTACTACAACCAGAGCGAGGCCGGTGAGTGA

CCCCGGCCCGGGGCGCAGGTCACGACCTCTCATCCCCCACGGACGGGCCGGGTCGCCCAC

AGTCTCCGGGTCCGAGATCCACCCCGAAGCCGCGGGACCCCGAGACCCTTGCCCCGGGAG

AGGCCCAGGCGCCTTTACCCGGTTTCATTTTCAGTTTAGGCCAAAAATCCCCCCGGGTTG

GTCGGGGCCGGACGGGGCTCGGGGGACTGGGCTGACCGTGGGGTCGGGGCCAGGTTCTCA

CACCATCCAGATGATGTATGGCTGCGACGTGGGGTCGGACGGGCGCTTCCTCCGCGGGTA

CCGGCAGGACGCCTACGACGGCAAGGATTACATCGCCTTGAACGAGGACCTGCGCTCTTG

GACCGCGGCGGACATGGCGGCTCAGATCACCCAGCGCAAGTGGGAGGCGGCCCGTGTGGC

GGAGCAGTTGAGAGCCTACCTGGAGGGCACGTGCGTGGAGTGGCTCCGCAGATACCTGGA

GAACGGGAAGGAGACGCTGCAGCGCACGGGTACCGGGGGCCACGGGGCGCCTCCCTGATC

GCCTGTAGATCTCCCGGGCTGGCCTCCCACAAGGAGGGGAGACAATTGGGACCAACACTA

GAATATCGCCCTCCCTCTGGTCCTGAGGGAGAGGAATCCTCCTGGGTTTCCAGATCCTGT

ACCAGAGAGTGACTCTGAGGTTCCGCCCTGCTCTCTGACACAATTAAGGGATAAAATCTC

TGAAGGAATGACGGGAAGACGATCCCTCGAATACTGATGAGTGGTTCCCTTTGACACACA

CCGGCAGCAGCCTTGGGCCCGTGACTTTTCCTCTCAGGCCTTGTTCTCTGCTTCACACTC

AATGTGTGTGGGGGTCTGAGTCCAGCACTTCTGAGTCCCTCAGCCTCCACTCAGGTCAGG

ACCAGAAGTCGCTGTTCCCTCTTCAGGGACTAGAATTTTCCACGGAATAGGAGATTATCC

CAGGTGCCTGTGTCCAGGCTGGTGTCTGGGTTCTGTGCTCCCTTCCCCATCCCAGGTGTC

CTGTCCATTCTCAAGATAGCCACATGTGTGCTGGAGGAGTGTCCCATGACAGATGCAAAA

TGCCTGAATGTTCTGACTCTTCCTGACAGACGCCCCCAAGACGCATATGACTCACCACGC

TGTCTCTGACCATGAGGCCACCCTGAGGTGCTGGGCCCTGAGCTTCTACCCTGCGGAGAT

CACACTGACCTGGCAGCGGGATGGGGAGGACCAGACCCAGGACACGGAGCTTGTGGAGAC

CAGGCCTGCAGGGGATGGAACCTTCCAGAAGTGGGCGTCTGTGGTGGTGCCTTCTGGACA

GGAGCAGAGATACACCTGCCATGTGCAGCATGAGGGTCTGCCCAAGCCCCTCACCCTGAG

ATGGGGTAAGGAGGGAGATGGGGGTGTCATGTCTTTTAGGGAAAGCAGGAGCCTCTCTGA

CCTTTAGCAGGGTCAGGGCCCCTCACCTTCCCCTCTTTTCCCAGAGCCGTCTTCCCAGCC

CACCATCCCCATCGTGGGCATCATTGCTGGCCTAGTTCTCTTTGGAGCTGTGTTCGCTGG

AGCTGTGGTCGCTGCTGTGAGGTGGAGGAGGAAGAGCTCAGGTGGGGTGAAGGGATGAAG

GGTGGGTCTGAGATTTCTTGTCTCACTGAGGGTTCCAAGACCCAGGTAGAAGTGTGCCCT

GCCTCGTTACTGGGAAGCACCATCCACAATTATGGGCCTACCCAGCCTGGGCCCTGTGTG

CCAGCACTTACTCTTTTGTAAAGCACCTGTTAAAATGAAGGACAGATTTATCACCTTGAT

TACGGCGGTGATGGGACCTGATCCCAGCAGTCACAAGTCACAGGGGAAGGTCCCTGAGGA

CCTTCAGGAGGGCGGTTGGTCCAGGACCCACACCTGCTTTCTTCATGTTTCCTGATCCCG

CCCTGGGTCTGCAGTCACACATTTCTGGAAACTTCTCTGAGGTCCAAGACTTGGAGGTTC

CTCTAGGACCTTAAGGCCCTGGCTCCTTTCTGGTATCTCACAGGACATTTTCTTCCCACA

GATAGAAAAGGAGGGAGCTACTCTCAGGCTGCAAGTAAGTATGAAGGAGGCTGATGCCTG

AGGTCCTTGGGATATTGTGTTTGGGAGCCCGTGGGGGAGCTCACCCACCCCACAATTCCT

CCTCTAGCCACATCTTCTGTGGGATCTGACCAGGTTCTGTTTTTGTCCTACCCCAGGCAG

TGACAGTGCCCAGGGCTCTGATATGTCTCTCACAGCTTGTAAAGGTGAGAGCCTGGAGGG

CCTGATGTGTGTTGGGTGTTGGGCGGAACAGTGGACGCAGCTGTGCTATGGGGTTTCTTT

GCATTGGATGTATTGAGCATGCGATGGGCTGTTTAAAGTGTGACTCCTCACTGTGACAGA

TACGAATTTGTTCATGAATATTTTTTTCTATAGTGTGAGACAGCTGCCTTGTGTGGGACT

GAGAGGCAAGATTTGTTCCTGCCCTTCCCTTTGTGACTTGAAGAACCCTGACTTTGTTTC

TGCAAAGGCACCTGCATGTGTCTGTGTTCTTGTAGGCATAATGTGAGGAGGTGGGGAGAC

CACCCCACCCCCATGTCCACCATGACCCTCTTCCCACGCTGACCTGTGCTCCCTCCCCAA

TCATCTTTCCTGTTCCAGAGAGGTGGGGCTGAGGTGTCTCCATCTCTGCCTCAACTTCAT

GGTGCACTGAGCTGTAACTTCTTCCTTCCCTATTAAAA

>HLA:HLA00101 A*32:01:01:01

CAGGAGCAGAGGGGTCAGGGCGAAGTCCCAGGGCCCCAGGCGTGGCTCTCAGGGTCTCAG

GCCCCGAAGGCGGTGTATGGATTGGGGAGTCCCAGCCTTGGGGATTCCCCAACTCCGCAG

TTTCTTTTCTCCCTCTGCCAACCTATGTAGGGTCCTTCTTCCTGGATACTCACGACGCGG

ACCCAGTTCTCACTCCCATTGGGTGTCGGGTTTCCAGAGAAGCCAATCAGTGTCGTCGCG

GTCGCGGTTCTAAAGTCCGCACGCACCCACCGGGACTCAGATTCTCCCCAGACGCCGAGG

ATGGCCGTCATGGCGCCCCGAACCCTCCTCCTGCTACTCTTGGGGGCCCTGGCCCTGACC

CAGACCTGGGCGGGTGAGTGCGGGGTCGTGGGGAAACCGCCTCTGCGGGGAGAAGCAAGG

GGCCCGCCCGGCGGGGGCGCAGGACCCGGGTAGCCGCGCCGGGAGGAGGGTCGGGCGGGT

CTCAGCCACTCCTCGCCCCCAGGCTCCCACTCCATGAGGTATTTCTTCACATCCGTGTCC

CGGCCCGGCCGCGGGGAGCCCCGCTTCATCGCCGTGGGCTACGTGGACGACACGCAGTTC

GTGCGGTTTGACAGCGACGCCGCGAGCCAGAGGATGGAGCCGCGGGCGCCGTGGATAGAG

CAGGAGGGGCCGGAGTATTGGGACCAGGAGACACGGAATGTGAAGGCCCACTCACAGACT

GACCGAGAGAGCCTGCGGATCGCGCTCCGCTACTACAACCAGAGCGAGGCCGGTGAGTGA

CCCCGGCCCGGGGCGCAGGTCACGACCTCTCATCCCCCACGGACGGGCCAGGTCGCCCAC

AGTCTCCGGGTCCGAGATCCACCCCGAAGCCGCGGGACCCCGAGACCCTTGCCCCGGGAG

AGGCCCAGGCGCCTTTACCCGGTTTCATTTTCAGTTTAGGCCAAAAATCCCCCCGGGTTG

GTCGGGGCCGGACGGGGCTCGGGGGACTGGGCTGACCGTGGGGTCGGGGCCAGGTTCTCA

CACCATCCAGATGATGTATGGCTGCGACGTGGGGCCGGACGGGCGCCTCCTCCGCGGGTA

CCAGCAGGACGCCTACGACGGCAAGGATTACATCGCCTTGAACGAGGACCTGCGCTCTTG

GACCGCGGCGGACATGGCGGCTCAGATCACCCAGCGCAAGTGGGAGGCGGCCCGTGTGGC

GGAGCAGTTGAGAGCCTACCTGGAGGGCACGTGCGTGGAGTGGCTCCGCAGATACCTGGA

GAACGGGAAGGAGACGCTGCAGCGCACGGGTACCAGGGGCCACGGGGCGCCTCCCTGATC

GCCTGTAGATCTCCCGGGCTGGCCTCCCACAAGGAGGGGAGACAATTGGGACCAACACTA

GAATATCGCCCTCCCTCTGGTCCTGAGGGAGAGGAATCCTCCTGGGTTTCCAGATCCTGT

ACCAGAGAGTGACTCTGAGGTTCCGCCCTGCTCTCTGACACAATTAAGGGATAAAATCTC

TGAAGGAATGACGGGAAGACGATCCCTCGAATACTGATGAGTGGTTCCCTTTGACACACA

CCGGCAGCAGCCTTGGGCCCGTGACTTTTCCTCTCAGGCCTTGTTCTCTGCTTCACACTC

AATGTGTGTGGGGGTCTGAGTCCAGCACTTCTGAGTCCCTCAGCCTCCACTCAGGTCAGG

ACCAGAAGTCGCTGTTCCCTCTTCAGGGACTAGAATTTTCCACGGAATAGGAGATTATCC

CAGGTGCCTGTGTCCAGGCTGGTGTCTGGGTTCTGTGCTCCCTTCCCCATCCCAGGTGTC

CTGTCCATTCTCAAGATAGCCACATGTGTGCTGGAGGAGTGTCCCATGACAGATGCAAAA

TGCCTGAATGTTCTGACTCTTCCTGACAGACGCCCCCAAGACGCATATGACTCACCACGC

TGTCTCTGACCATGAGGCCACCCTGAGGTGCTGGGCCCTGAGCTTCTACCCTGCGGAGAT

CACACTGACCTGGCAGCGGGATGGGGAGGACCAGACCCAGGACACGGAGCTTGTGGAGAC

CAGGCCTGCAGGGGATGGAACCTTCCAGAAGTGGGCGTCTGTGGTGGTGCCTTCTGGACA

GGAGCAGAGATACACCTGCCATGTGCAGCATGAGGGTCTGCCCAAGCCCCTCACCCTGAG

ATGGGGTAAGGAGGGAGATGGGGGTGTCATGTCTTTTACGGAAAGCAGGAGCCTCTCTGA

CCTTTAGCAGGGTCAGGGCCCCTCACCTTCCCCTCTTTTCCCAGAGCCGTCTTCCCAGCC

CACCATCCCCATCGTGGGCATCATTGCTGGCCTAGTTCTCTTTGGAGCTATGTTCGCTGG

AGCTGTGGTCGCTGCTGTGAGGTGGAGGAGGAAGAGCTCAGGTGGGGTGAAGGGATGAAG

GGTGGGTCTGAGATTTCTTGTCTCACTGAGGGTTCCAAGACCCAGGTAGAAGTGTGCCCT

GCCTCGTTACTGGGAAGCACCATCCACAATTATGGGCCTACCCAGCCTGGGCCCTGTGTG

CCAGCACTTACTCTTTTGTAAAGCACCTGTTAAAATGAAGGACAGATTTATCACCTTGAT

TACGGCGGTGATGGGACCTGATCCCAGCAGTCACAAGTCACAGGGGAAGGTCCCTGAGGA

CCTTCAGGAGGGCGGTTGGTCCAGGACCCACACCTGCTTTCTTCATGTTTCCTGATCCCG

CCCTGGGTCTGCAGTCACACATTTCTGGAAACTTCTCTGAGGTCCAAGACTTGGAGGTTC

CTCTAGGACCTTAAGGCCCTGGCTCCTTTCTGGTATCTCACAGGACATTTTCTTCCCACA

GATAGAAAAGGAGGGAGCTACTCTCAGGCTGCAAGTAAGTATGAAGGAGGCTGATGCCTG

AGGTCCTTGGGATATTGTGTTTGGGAGCCCGTGGGGGAGCTCACCCACCCCACAATTCCT

CCTCTAGCCACATCTTCTGTGGGATCTGACCAGGTTCTGTTTTTGTCCTACCCCAGGCAG

TGACAGTGCCCAGGGCTCTGATATGTCTCTCACAGCTTGTAAAGGTGAGAGCCTGGAGGG

CCTGATGTGTGTTGGGTGTTGGGCGGAACAGTGGACGCAGCTGTGCTATGGGGTTTCTTT

GCATTGGATGTATTGAGCATGCGATGGGCTGTTTAAAGTGTGACTCCTCACTGTGACAGA

TACGAATTTGTTCATGAATATTTTTTTCTATAGTGTGAGACAGCTGCCTTGTGTGGGACT

GAGAGGCAAGATTTGTTCCTGCCCTTCCCTTTGTGACTTGAAGAACCCTGACTTTGTTTC

TGCAAAGGCACCTGCATGTGTCTGTGTTCTTGTAGGCATAATGTGAGGAGGTGGGGAGAC

CACCCCACCCCCATGTCCACCATGACCCTCTTCCCACGCTGACCTGTGCTCCCTCCCCAA

TCATCTTTCCTGTTCCAGAGAGGTGGGGCTGAGGTGTCTCCATCTCTGCCTCAACTTCAT

GGTGCACTGAGCTGTAACTTCTTCCTTCCCTATTAAAA

>HLA:HLA00213 B*18:01:01:01

GATCAGGACGAAGTCCCAGGCCCCGGGCGGGGCTCTCAGGGTCTCAGGCTCCGAGAGCCT

TGTCTGCATTGGGGAGGCGCAGCATTGGGGATTCCCCACTCCCACGAGTTTCACTTCTTC

TCCCAACCTATGTCGGGTCCTTCTTCCAGGATACTCGTGACGCGTCCCCATTTCCCACTC

CCATTGGGTGTCGGGTGTCTAGAGAAGCCAATCAGTGTCGCCGGGGTCCCAGTTCTAAAG

TCCCCACGCACCCACCCGGACTCAGAATCTCCTCAGACGCCGAGATGCGGGTCACGGCGC

CCCGAACCCTCCTCCTGCTGCTCTGGGGGGCAGTGGCCCTGACCGAGACCTGGGCTGGTG

AGTGCGGGGTCGGCAGGGAAATGGCCTCTGTGGGGAGGAGCGAGGGGACCGCAGGCGGGG

GCGCAGGACCCGGGGAGCCGCGCCGGGAGGAGGGTCGGGCGGGTCTCAGCCCCTCCTTGC

CCCAGGCTCCCACTCCATGAGGTATTTCCACACCTCCGTGTCCCGGCCCGGCCGCGGGGA

GCCCCGCTTCATCTCAGTGGGCTACGTGGACGGCACCCAGTTCGTGAGGTTCGACAGCGA

CGCCGCGAGTCCGAGGACGGAGCCCCGGGCGCCGTGGATAGAGCAAGAGGGGCCGGAGTA

TTGGGACCGGAACACACAGATCTCCAAGACCAACACACAGACTTACCGAGAGAGCCTGCG

GAACCTGCGCGGCTACTACAACCAGAGCGAGGCCGGTGAGTGACCCCGGCCCGGGGCGCA

GGTCACGACTCCCCATCCCCCACGTACGGCCCGGGTCGCCCCGAGTCTCCGGGTCCGAGA

TCCGCCCCCCTGAGGCCGCGGGACCCGCCCAGACCCTCGACCGGCGAGAGCCCCAGGCGC

GTTTACCCGGTTTCATTTTCAGTTGAGGCCAAAATCCCCGCGGGTTGGTCGGGGCGGGGC

GGGGCTCGGGGGGACGGGGCTGACCGCGGGGCCGGGGCCAGGGTCTCACACCCTCCAGAG

GATGTACGGCTGCGACGTGGGGCCGGACGGGCGCCTCCTCCGCGGGCATGACCAGTCCGC

CTACGACGGCAAGGATTACATCGCCCTGAACGAGGACCTGAGCTCCTGGACCGCGGCGGA

CACCGCGGCTCAGATCACCCAGCGCAAGTGGGAGGCGGCCCGTGTGGCGGAGCAGCTGAG

AGCCTACCTGGAGGGCACGTGCGTGGAGTGGCTCCGCAGACACCTGGAGAACGGGAAGGA

GACGCTGCAGCGCGCGGGTACCAGGGGCAGTGGGGAGCCTTCCCCATCTCCTATAGGTCG

CCGGGGATGGCCTCCCACGAGAAGAGGAGGAAAATGGGATCAGCGCTAGAATGTCGCCCT

CCCTTGAATGGAGAATGGCATGAGTTTTCCTGAGTTTCCTCTGAGGGCCCCCTCTTCTCT

CTAGGACAATTAAGGGATGACGTCTCTGAGGAAATGGAGGGGAAGACAGTCCCTAGAATA

CTGATCAGGGGTCCCCTTTGACCCCTGCAGCAGCCTTGGGAACCATGACTTTTCCTCTCA

GGCCTTGTTCTCTGCCTCACACTCAGTGTGTTTGGGGCTCTGATTCCAGCACTTCTGAGT

CACTTTACCTCCACTCAGATCAGGAGCAGAAGTCTCTGTTCCCCGCTCAGAGACTCGAAC

TTTCCAATGAATAGATTATCCCAGGTGCCTGCGTCCAGGCTGGTGTCTGGGTTCTGTGCC

CCTTCCCCACCCCAGGTGTCCTGTCCATTCTCAGGCTGGTCACATGGGTGGTCCTAGGGT

GTCCCATGAGAGATGCAAAGCGCCTGAATTTTCTGACTCTTCCCATCAGACCCCCCAAAG

ACACATGTGACCCACCACCCCATCTCTGACCATGAGGCCACCCTGAGGTGCTGGGCCCTG

GGCTTCTACCCTGCGGAGATCACACTGACCTGGCAGCGGGATGGCGAGGACCAAACTCAG

GACACCGAGCTTGTGGAGACCAGACCAGCAGGAGATAGAACCTTCCAGAAGTGGGCAGCT

GTGGTGGTGCCTTCTGGAGAAGAGCAGAGATACACATGCCATGTACAGCATGAGGGGCTG

CCGAAGCCCCTCACCCTGAGATGGGGTAAGGAGGGGGATGAGGGGTCATATCTCTTCTCA

GGGAAAGCAGGAGCCCTTCTGGAGCCCTTCAGCAGGGTCAGGGCCCCTCATCTTCCCCTC

CTTTCCCAGAGCCATCTTCCCAGTCCACCATCCCCATCGTGGGCATTGTTGCTGGCCTGG

CTGTCCTAGCAGTTGTGGTCATCGGAGCTGTGGTCGCTACTGTGATGTGTAGGAGGAAGA

GCTCAGGTAGGGAAGGGGTGAGGGGTGGGGTCTGGGTTTTCTTGTCCCACTGGGGGTTTC

AAGCCCCAGGTAGAAGTGTTCCCTGCCTCATTACTGGGAAGCAGCATCCACACAGGGGCT

AACACAGCCTGGGACCCTGTGTGCCAGCACTTACTCTTTTGTGCAGCACATGTGACAATG

AAGGACGGATGTATCACCTTGATGGTTGTGGTGTTGGGGTCCTGATTCCAGCATTCATGA

GTCAGGGGAAGGTCCCTGCTAAGGACAGACCTTAGGAGGGCAGTTGGTCCAGGACCCACA

CTTGCTTTCCTCGTGTTTCCTGATCCTGCCTTGGGTCTGTAGTCATACTTCTGGAAATTC

CTTTTGGTTCCAAGACGAGGAGGTTCCTCTAAGATCTCATGGCCCTGCTTCCTCCCAGTC

CCCTCACAGGACATTTTCTTCCCACAGGTGGAAAAGGAGGGAGCTACTCTCAGGCTGCGT

GTAAGTGGTGGGGGTGGGAGTGTGGAGGAGCTCACCCACCCCATAATTCCTCCTGTCCCA

CGTCTCCTGCGGGCTCTGACCAGGTCCTGTTTTTGTTCTACTCCAGCCAGCGACAGTGCC

CAGGGCTCTGATGTGTCTCTCACAGCTTGAAAAGGTGAGATTCTTGGGGTCTAGAGTGGG

CGGGGGGGCGGGGAGGGGGCAGAGGGGAAAGGCCTGGGTAATGGAGATTCTTTGATTGGG

ATGTTTCGCGTGTGTGGTGGGCTGTTCAGAGTGTCATCACTTACCATGACTAACCAGAAT

TTGTTCATGACTGTTGTTTTCTGTAGCCTGAGACAGCTGTCTTGTGAGGGACCGAGATGC

AGGATTTCTTCACGCCTCCCCTTTGTGACTTCAAGAGCCTCTGGCATCTCTTTCTGCAAA

GGCACCTGAATGTGTCTGCGTCCCTGTTAGCATAATGTGAGGAGGTGGAGAGACAGCTCA

CCCCCGTGTCCACCGTGACCCCTGTTCCCATGCTGACCTGTGTTTCCTCCCCAGTCATCT

TTCTTGTTCCAGAGAGGTGGGGCTGGATGTCTCCATCTCTGTCTCAACTTTATGTGCACT

GAGCTGCAACTTCTTACTTCCCTACTGAAAATAAGAATCTGAATATAAATTTGTTTTCTC

AAATATTTGCTATGAGAGGTTGATGGATTAATTAAATAAGTCAATTCCTGGAATTTGAGA

GAGCAAATAAAGACCTGAGAACCTTCCAGAATCTGCATGTTCGCTGTGCTGAGTCTGTTG

CAGGTGGGGTGTGGAGAAGGCTGTGGGGGGCCGAGTGTGGACGGGGCCTGTGCCCATTTG

GTGTTGAGTCCATCATGGGCTTTATGTGGTTAGTCCTCAGCTGGGTCACCTTCACTGCTC

CATTGTCCTTGTCCCTTCAGTGGAAACTTGTCCAGCGGGAGCTGTGACCACAGAGGCTCA

CACATCGCCCAGGGCGGCCCCTGCACACGGGGGTCTCTGTGCATTCTGAGACAAATTTTC

AGAGCCATTCACCTCCTGCCCTGCTTCTAGAGCTCCTTTTCTGCTCTGCTCTCCTGCCCT

CTCTCCCTGCCCTGGTTCTAGTGATCTTGGTGCTGAATCCAATCCCAACTCCTGAATCTG

TAAAGCAGAGTCTAATTTAGACTTACATTTGTCTGTGAAATTGGACCCGTCATCAAGGAC

TGTTCTTTCCTGAAGAGAGAACCTGATTGTGTGCTGCAGTGTGCTGGGGCAGGGGGTGCG

G

>HLA:HLA00292 B*40:01:02:01

GATCAGGACGAAGTCCCAGGCCCCGGGCGGGGCTCTCAGGGTCTCAGGCTCCGAGGGCCG

CGTCTGCAATGGGGAGGCGCAGCGTTGGGGATTCCCCACTCCCACGAGTTTCACTTCTTC

TCCCAACCTATGTCGGGTCCTTCTTCCAGGATACTCGTGACGCGTCCCCATTTCCCACTC

CCATTGGGTGTCGGGTGTCTAGAGAAGCCAATCAGCGTCGCCGTGGTCCCAGTTCTAAAG

TCCCCACGCACCCACCCGGACTCAGAATCTCCTCAGACGCCGAGATGCGGGTCACGGCAC

CCCGAACCGTCCTCCTGCTGCTCTCGGCGGCCCTGGCCCTGACCGAGACCTGGGCCGGTG

AGTGCGGGTCGGCAGGGAAATGGCCTCTGTGGGGAGGAGCGAGGGGACCGCAGGCGGGGG

CGCAGGACCCGGGGAGCCGCGCCGGGAGGAGGGTCGGGCGGGTCTCAGCTCCTCCTCGCC

CCCAGGCTCCCACTCCATGAGGTATTTCCACACCGCCATGTCCCGGCCCGGCCGCGGGGA

GCCCCGCTTCATCACCGTGGGCTACGTGGACGACACGCTGTTCGTGAGGTTCGACAGCGA

CGCCACGAGTCCGAGGAAGGAGCCGCGGGCGCCATGGATAGAGCAGGAGGGGCCGGAGTA

TTGGGACCGGGAGACACAGATCTCCAAGACCAACACACAGACTTACCGAGAGAGCCTGCG

GAACCTGCGCGGCTACTACAACCAGAGCGAGGCCGGTGAGTGACCCCGGCCCGGGGCGCA

GGTCACGACTCCCCATCCCCCACGTACGGCCCGGGTCGCCCCGAGTCTCCGGGTCCGAGA

TCCGACCCCCTGAGGCCGCGGGACCCGCCCAGACCCTCGACCGGCGAGAGCCCCAGGCGC

GTTTACCCGGTTTCATTTTCAGTTGAGGCCAAAATCCCCGCGGGTTGGTCGGGGCGGGGC

GGGGCTCGGGGGACTGGGCTGACCGCGGGGCCGGGGCCAGGGTCTCACACCCTCCAGAGG

ATGTACGGCTGCGACGTGGGGCCGGACGGGCGCCTCCTCCGCGGGCATAACCAGTACGCC

TACGACGGCAAGGATTACATCGCCCTGAACGAGGACCTGCGCTCCTGGACCGCCGCGGAC

ACGGCGGCTCAGATCTCCCAGCGCAAGTTGGAGGCGGCCCGTGTGGCGGAGCAGCTGAGA

GCCTACCTGGAGGGCGAGTGCGTGGAGTGGCTCCGCAGATACCTGGAGAACGGGAAGGAC

AAGCTGGAGCGCGCTGGTACCAGGGGCAGTGGGGAGCCTTCCCCATCTCCTATAGGTCGC

CGGGGATGGCCTCCCACGAGAAGAGGAGGAAAATGGGATCAGCGCTAGAATGTCGCCCTC

CGTTGAATGGAGAATGGCATGAGTTTTCCTGAGTTTCCTCTGAGGGCCCCCTCTTCTCTC

TAGACAATTAAGGAATGACGTCTCTGAGGAAATGGAGGGGAAGACAGTCCCTAGAATACT

GATCAGGGGTCCCCTTTGACCCCTGCAGCAGCCTTGGGAACCGTGACTTTTCCTCTCAGG

CCTTGTTCTCTGCCTCACACTCAGTGTGTTTGGGGCTCTGATTCCAGCACTTCTGAGTCA

CTTTACCTCCACTCAGATCAGGAGCAGAAGTCCCTGTTCCCCGCTCAGAGACTCGAACTT

TCCAATGAATAGGAGATTATCCCAGGTGCCTGCGTCCAGGCTGGTGTCTGGGTTCTGTGC

CCCTTCCCCACCCCAGGTGTCCTGTCCATTCTCAGGCTGGTCACATGGGTGGTCCTAGGG

TGTCCCATGAAAGATGCAAAGCGCCTGAATTTTCTGACTCTTCCCATCAGACCCCCCAAA

GACACACGTGACCCACCACCCCATCTCTGACCATGAGGCCACCCTGAGGTGCTGGGCCCT

GGGTTTCTACCCTGCGGAGATCACACTGACCTGGCAGCGGGATGGCGAGGACCAAACTCA

GGACACTGAGCTTGTGGAGACCAGACCAGCAGGAGATAGAACCTTCCAGAAGTGGGCAGC

TGTGGTGGTGCCTTCTGGAGAAGAGCAGAGATACACATGCCATGTACAGCATGAGGGGCT

GCCGAAGCCCCTCACCCTGAGATGGGGTAAGGAGGGGGATGAGGGGTCATATCTCTTCTC

AGGGAAAGCAGGAGCCCTTCAGCAGGGTCAGGGCCCCTCATCTTCCCCTCCTTTCCCAGA

GCCGTCTTCCCAGTCCACCGTCCCCATCGTGGGCATTGTTGCTGGCCTGGCTGTCCTAGC

AGTTGTGGTCATCGGAGCTGTGGTCGCTGCTGTGATGTGTAGGAGGAAGAGTTCAGGTAG

GGAAGGGGTGAGGGGTGGGGTCTGGGTTTTCTTGTCCCACTGGGGGTTTCAAGCCCCAGG

TAGAAGTGTTCCCTGCCTCATTACTGGGAAGCAGCATGCACACAGGGGCTAACGCAGCCT

GGGACCCTGTGTGCCAGCACTTACTCTTTTGTGCAGCACATGTGACAATGAAGGATGGAT

GTATCACCTTGATGGTTGTGGTGTTGGGGTCCTGATTCCAGCATTCATGAGTCAGGGGAA

GGTCCCTGCTAAGGACAGACCTTAGGAGGGCAGTTGGTCCAGGACCCACACTTGCTTTCC

TCGTGTTTCCTGATCCTGCCCTGGGTCTGTAGTCATACTTCTGGAAATTCCTTTTGGGTC

CAAGACTAGGAGGTTCCTCTAAGATCTCATGGCCCTGCTTCCTCCCAGTGCCCTCACAGG

ACATTTTCTTCCCACAGGTGGAAAAGGAGGGAGCTACTCTCAGGCTGCGTGTAAGTGGTG

GGGGTGGGAGTGTGGAGGAGCTCACCCACCCCATAATTCCTCCTGTCCCACGTCTCCTGC

GGGCTCTGACCAGGTCCTGTTTTTGTTCTACTCCAGGCAGCGACAGTGCCCAGGGCTCTG

ATGTGTCTCTCACAGCTTGAAAAGGTGAGATTCTTGGGGTCTAGAGTGGGTGGGGTGGCG

GGTCTGGGGGTGGGTGGGGCAGAGGGGAAAGGCCTGGGTAATGGGGATTCTTTGATTGGG

ATGTTTCGCGTGTGTGGTGGGCTGTTTAGAGTGTCATCGCTTACCATGACTAACCAGAAT

TTGTTCATGACTGTTGTTTTCTGTAGCCTGAGACAGCTGTCTTGTGAGGGACTGAGATGC

AGGATTTCTTCACGCCTCCCCTTTGTGACTTCAAGAGCCTCTGGCATCTCTTTCTGCAAA

GGCACCTGAATGTGTCTGCGTCCCTGTTAGCATAATGTGAGGAGGTGGAGAGACAGCCCA

CCCTTGTGTCCACTGTGACCCCTGTTCCCATGCTGACCTGTGTTTCCTCCCCAGTCATCT

TTCTTGTTCCAGAGAGGTGGGGCTGGATGTCTCCATCTCTGTCTCAACTTTACGTGCACT

GAGCTGCAACTTCTTACTTCCCTACTGAAAATAAGAATCTGAATATAAATTTGTTTTCTC

AAATATTTGCTATGAGAGGTTGATGGATTAATTAAATAAGTCAATTCCTGGAATTTGAAA

GAGCAAATAAAGACCTGAGAACCTTCCAGAATCTGCATGTTCGCTGTGCTGAGTCTGTTG

CAGGTGGGGTGTGGAGAAGGCTGTGGGGGGCCGAGTGTGGATGGGGCCTGTGCCCATTTG

GTGTTGAGTCCATCATGGGCTTTATGTGGTTAGTCCTCAGCTGGGTCACCTTCACTGCTC

CATTGTCCTTGTCCCTTCAGTGGAAACTTGTCCAGCGGGAGCTGTGACCACAGAGGCTCA

CACATCGCCCAGGGCGGCCCCTGCACACGGGGATCTCTGTGCATTCTGAGACAAATTTTC

AGAGCCATTCACCTCCTGCCCTGCTTCTAGAGCTCCTTTTCTGCTCTGCTCTTCTGCCCT

CTCTCCCTGCCCTGGTTCTAGTGATCTTGGTGCTGAATCCAATCCCAACTCATGAATCTG

TAAAGCAGAGTCTAATTTAGACTTACATTTGTCTGTGAAATTGGACCCATCATCAAGGAC

TGTTCTTTCCTGAAGAGAGAACCTGATTGTGTGCTGCAGTGTGCTGGGGCAGGGGGTGCG

G

>HLA:HLA00318 B*44:02:01:01

GATCAGGACGAAGTCCCAGGTCCCGGACGGGGCTCTCAGGGTCTCAGGCTCCGAGGGCCG

CGTCTGCAATGGGGAGGCGCAGCGTTGGGGATTCCCCACTCCCACGAGTTTCACTTCTTC

TCCCAACCTATGTCGGGTCCTTCTTCCAGGATACTCGTGACGCGTCCCCATTTCCCACTC

CCATTGGGTGTCGGGTGTCTAGAGAAGCCAATCAGTGTCGCCGGGGTCCCAGTTCTAAAG

TCCCCACGCACCCACCCGGACTCAGAATCTCCTCAGACGCCGAGATGCGGGTCACGGCGC

CCCGAACCCTCCTCCTGCTGCTCTGGGGGGCAGTGGCCCTGACCGAGACCTGGGCCGGTG

AGTGCGGGGTCGGGAGGGAAATGGCCTCTGTGGGGAGGAGAGAGGGGACCGCAGGCGGGG

GCGCAGGACCCGGGGAGCCGCGCCGGGAGGAGGGTCGGGCGGGTCTCAGCCCCTCCTCGC

CCCCAGGCTCCCACTCCATGAGGTATTTCTACACCGCCATGTCCCGGCCCGGCCGCGGGG

AGCCCCGCTTCATCACCGTGGGCTACGTGGACGACACGCTGTTCGTGAGGTTCGACAGCG

ACGCCACGAGTCCGAGGAAGGAGCCGCGGGCGCCATGGATAGAGCAGGAGGGGCCGGAGT

ATTGGGACCGGGAGACACAGATCTCCAAGACCAACACACAGACTTACCGAGAGAACCTGC

GCACCGCGCTCCGCTACTACAACCAGAGCGAGGCCGGTGAGTGACCCCGGCCCGGGGCGC

AGGTCACGACTCCCCATCCCCCACGTACGGCCCGGGTCGCCCCGAGTCTCCGGGTCCGAG

ATCCGCCCCCGAGGCCGCGGGACCCGCCCAGACCCTCGACCGGCGAGAGCCCCAGGCGCG

TTTACCCGGTTTCATTTTCAGTTGAGGCCAAAATCCCCGCGGGTTGGTCGGGGCGGGGCG

GGGCTCGGGGGACGGGGCTGACCGCGGGGCCGGGGCCAGGGTCTCACATCATCCAGAGGA

TGTACGGCTGCGACGTGGGGCCGGACGGGCGCCTCCTCCGCGGGTATGACCAGGACGCCT

ACGACGGCAAGGATTACATCGCCCTGAACGAGGACCTGAGCTCCTGGACCGCGGCGGACA

CCGCGGCTCAGATCACCCAGCGCAAGTGGGAGGCGGCCCGTGTGGCGGAGCAGGACAGAG

CCTACCTGGAGGGCCTGTGCGTGGAGTCGCTCCGCAGATACCTGGAGAACGGGAAGGAGA

CGCTGCAGCGCGCGGGTACCAGGGGCAGTGGGGAGCCTTCCCCATCTCCTATAGGTCGCC

GGGGATGGCCTCCCACGAGAAGAGGAGGAAAATGGGATCAGCGCTAGAATGTCGCCCTCC

CTTGAATGGAGAATGGCATGAGTTTTCCTGAGTTTCCTCTGAGGGCCCCCTCTTCTCTCT

AGGACAATTAAGGGATGACGTCTCTGAGGAAATGGAGGGGAAGACAGTCCCTAGAATACT

GATCAGGGGTCCCCTTTGACCCCTGCAGCAGCCTTGGGAACCGTGACTTTTCCTCTCAGG

CCTTGTTCTCTGCCTCACACTCAGTGTGTTTGGGGCTCTGATTCCAGCACTTCTGAGTCA

CTTTACCTCCACTCAGATCAGGAGCAGAAGTCCCTGTTCCCCGCTCAGAGACTCGAACTT

TCCAATGAATAGGAGATTATCCCAGGTGCCTGCGTCCAGGCTGGTGTCTGGGTTCTGTGC

CCCTTCCCCACCCCAGGTGTCCTGTCCATTCTCAGGCTGGTCACATGGGTGGTCCTAGGG

TGTCCCATGAGAGATGCAAAGCGCCTGAATTTTCTGACTCTTCCCATCAGACCCCCCAAA

GACACATGTGACCCACCACCCCATCTCTGACCATGAGGTCACCCTGAGGTGCTGGGCCCT

GGGCTTCTACCCTGCGGAGATCACACTGACCTGGCAGCGGGATGGCGAGGACCAAACTCA

GGACACCGAGCTTGTGGAGACCAGACCAGCAGGAGATAGAACCTTCCAGAAGTGGGCAGC

TGTGGTGGTGCCTTCTGGAGAAGAGCAGAGATACACATGCCATGTACAGCATGAGGGGCT

GCCGAAGCCCCTCACCCTGAGATGGGGTAAGGAGGGGGATGAGGGGTCATATCTCTTCTC

AGGGAAAGCAGGAGCCCTTCAGCAGGGTCAGGGCCCCTCATCTTCCCTTCCTTTCCCAGA

GCCGTCTTCCCAGTCCACCGTCCCCATCGTGGGCATTGTTGCTGGCCTGGCTGTCCTAGC

AGTTGTGGTCATCGGAGCTGTGGTCGCTGCTGTGATGTGTAGGAGGAAGAGCTCAGGTAG

GGAAGGGGTGAGGGGTGGGGTCTGGGTTTTCTTGTCCCACTGGGGGTTTCAAGCCCCAGG

TAGAAGTGTTCCCTGCCTCATTACTGGGAAGCAGCATCCACACAGGGGCTAACGCAGCCT

GGGACCCTGTGTGCCAGCACTTACTCTTTTGTGCAGCACATGTGACAATGAAGGACGGAT

GTATCACCTTGGTGGTTGTGGTGTTGGGGTCCTGATTCCAGCATTCATGAGTCAGGGGAA

GGTCCCTGCTAAGGACAGACCTTAGGAGGGCAGTTGGTCCAGGACCCACACTTGCTTTCC

TCGTGTTTCCTGATCCTGCCTTGGGTCTGTAGTCATACTTCTGGAAATTCCTTTTGGGTC

CAAGACGAGGAGGTTCCTCTAAGATCTCATGGCCCTGCTTCCTCCCAGTCCCCTCACAGG

GCATTTTCTTCCCACAGGTGGAAAAGGAGGGAGCTACTCTCAGGCTGCGTGTAAGTGATG

GGGGCGGGAGTGTGGAGGAGCTCACCCACCCCATAATTCCTCCTGTCCCACGTCTCCTGC

GGGCTCTGACCAGGTCCTGTTTTTGTTCTACTCCAGGCAGCGACAGTGCCCAGGGCTCTG

ATGTGTCTCTCACAGCTTGAAAAGGTGAGATTCTTGGGGTCTAGAGTGGGTGGGGTGGCA

GGTCTGGGGGTGGGTGGGGCAGTGGGGAAAGGCCTGGGTAATGGAGATTCTTTGATTGGG

ATGTTTCGCGTGTGTGGTGGGCTGTTTAGAGTGTCATCACTTACCATGACTAACCAGAAT

TTGTTCATGACTGTTGTTTTCTGTAGCCTGAGACAGCTGTCTTGTGAGGGACTGAGATGC

AGGATTTCTTCACGCCTCCCCTTTGTGACTTCAAGAGCCTCTGGCATCTCTTTCTGCAAA

GGCACCTGAATGTGTCTGCGTCCCTGTTAGCATAATGTGAGGAGGTGGAGAGACAGCCCA

CCCCCGTGTCCACCGTGACCCCTGTTCCCATGCTGACTTGTGTTTCCTCCCCAGTCATCT

TTCCTGTTCCAGAGAGGTGGGGCTGGATGTCTCCATCTCTGTCTCAACTTTATGTGCACT

GAGCTGCAACTTCTTACTTCCCTACTGAAAATAAGAATCTGAATATAAATTTGTTTTCTC

AAATATTTGCTATGAGAGGTTGATGGATTAATTAAATAAGTCAATTCCTGGAATTTGAGA

GAGCAAATAAAGACCTGAGAACCTTCCAGAATCTGCATGTTCGCTGTGCTGAGTCTGTTG

CAGGTGGGGTGTGGAGAAGGCTGTGGGGGGCCGAGTGTGGACGGGGCCTGTGCCCATTTG

GTGTTGAGTCCATCATGGGCTTTATGTGGTTAGTCTTCAGCTGGGTCACCTTCACTGCTC

CATTGTCCTTGTCCCTTCAGTGGAAACTTGTCCAGTGGGAGCTGTGACCACAGAGGCTCA

CACATCGCCCTGGGCGGCCCCTGCACGCGGGGGTCTCTGTGCATTCTGAGACAAATTTTC

AGAGCCATTCACCTCCTGCCCTGCTTCTAGAGCTCCTTTTCTGCTCTGCTCTTCTGCCCT

CTCTCCCTGCCCTGGTTCTAGTGATCTTGGTGCTGAATCCAATCCCAACTCATGAATCTG

TAAAGCAGAGTCTAATTTAGACTTACATTTGTCTGTGAAATTGGACCCATCATCAAGGAC

TGTTCTTTCCTGAAGAGAGAACCTGATTGTGTGCTGCAGTGTGCTGGGGCAGGGGGTGCG

G

>HLA:HLA00319 B*44:03:01:01

GATCAGGACGAAGTCCCAGGTCCCGGACGGGGCTCTCAGGGTCTCAGGCTCCGAGGGCCG

CGTCTGCAATGGGGAGGCGCAGCGTTGGGGATTCCCCACTCCCACGAGTTTCACTTCTTC

TCCCAACCTATGTCGGGTCCTTCTTCCAGGATACTCGTGACGCGTCCCCATTTCCCACTC

CCATTGGGTGTCGGGTGTCTAGAGAAGCCAATCAGTGTCGCCGGGGTCCCAGTTCTAAAG

TCCCCACGCACCCACCCGGACTCAGAATCTCCTCAGACGCCGAGATGCGGGTCACGGCGC

CCCGAACCCTCCTCCTGCTGCTCTGGGGGGCAGTGGCCCTGACCGAGACCTGGGCCGGTG

AGTGCGGGGTCGGGAGGGAAATGGCCTCTGTGGGGAGGAGAGAGGGGACCGCAGGCGGGG

GCGCAGGACCCGGGGAGCCGCGCCGGGAGGAGGGTCGGGCGGGTCTCAGCCCCTCCTCGC

CCCCAGGCTCCCACTCCATGAGGTATTTCTACACCGCCATGTCCCGGCCCGGCCGCGGGG

AGCCCCGCTTCATCACCGTGGGCTACGTGGACGACACGCTGTTCGTGAGGTTCGACAGCG

ACGCCACGAGTCCGAGGAAGGAGCCGCGGGCGCCATGGATAGAGCAGGAGGGGCCGGAGT

ATTGGGACCGGGAGACACAGATCTCCAAGACCAACACACAGACTTACCGAGAGAACCTGC

GCACCGCGCTCCGCTACTACAACCAGAGCGAGGCCGGTGAGTGACCCCGGCCCGGGGCGC

AGGTCACGACTCCCCATCCCCCACGTACGGCCCGGGTCGCCCCGAGTCTCCGGGTCCGAG

ATCCGCCCCCGAGGCCGCGGGACCCGCCCAGACCCTCGACCGGCGAGAGCCCCAGGCGCG

TTTACCCGGTTTCATTTTCAGTTGAGGCCAAAATCCCCGCGGGTTGGTCGGGGCGGGGCG

GGGCTCGGGGGACGGGGCTGACCGCGGGGCCGGGGCCAGGGTCTCACATCATCCAGAGGA

TGTACGGCTGCGACGTGGGGCCGGACGGGCGCCTCCTCCGCGGGTATGACCAGGACGCCT

ACGACGGCAAGGATTACATCGCCCTGAACGAGGACCTGAGCTCCTGGACCGCGGCGGACA

CCGCGGCTCAGATCACCCAGCGCAAGTGGGAGGCGGCCCGTGTGGCGGAGCAGCTGAGAG

CCTACCTGGAGGGCCTGTGCGTGGAGTCGCTCCGCAGATACCTGGAGAACGGGAAGGAGA

CGCTGCAGCGCGCGGGTACCAGGGGCAGTGGGGAGCCTTCCCCATCTCCTATAGGTCGCC

GGGGATGGCCTCCCACGAGAAGAGGAGGAAAATGGGATCAGCGCTAGAATGTCGCCCTCC

CTTGAATGGAGAATGGCATGAGTTTTCCTGAGTTTCCTCTGAGGGCCCCCTCTTCTCTCT

AGGACAATTAAGGGATGACGTCTCTGAGGAAATGGAGGGGAAGACAGTCCCTAGAATACT

GATCAGGGGTCCCCTTTGACCCCTGCAGCAGCCTTGGGAACCGTGACTTTTCCTCTCAGG

CCTTGTTCTCTGCCTCACACTCAGTGTGTTTGGGGCTCTGATTCCAGCACTTCTGAGTCA

CTTTACCTCCACTCAGATCAGGAGCAGAAGTCCCTGTTCCCCGCTCAGAGACTCGAACTT

TCCAATGAATAGGAGATTATCCCAGGTGCCTGCGTCCAGGCTGGTGTCTGGGTTCTGTGC

CCCTTCCCCACCCCAGGTGTCCTGTCCATTCTCAGGCTGGTCACATGGGTGGTCCTAGGG

TGTCCCATGAGAGATGCAAAGCGCCTGAATTTTCTGACTCTTCCCATCAGACCCCCCAAA

GACACATGTGACCCACCACCCCATCTCTGACCATGAGGTCACCCTGAGGTGCTGGGCCCT

GGGCTTCTACCCTGCGGAGATCACACTGACCTGGCAGCGGGATGGCGAGGACCAAACTCA

GGACACCGAGCTTGTGGAGACCAGACCAGCAGGAGATAGAACCTTCCAGAAGTGGGCAGC

TGTGGTGGTGCCTTCTGGAGAAGAGCAGAGATACACATGCCATGTACAGCATGAGGGGCT

GCCGAAGCCCCTCACCCTGAGATGGGGTAAGGAGGGGGATGAGGGGTCATATCTCTTCTC

AGGGAAAGCAGGAGCCCTTCAGCAGGGTCAGGGCCCCTCATCTTCCCTTCCTTTCCCAGA

GCCGTCTTCCCAGTCCACCGTCCCCATCGTGGGCATTGTTGCTGGCCTGGCTGTCCTAGC

AGTTGTGGTCATCGGAGCTGTGGTCGCTGCTGTGATGTGTAGGAGGAAGAGCTCAGGTAG

GGAAGGGGTGAGGGGTGGGGTCTGGGTTTTCTTGTCCCACTGGGGGTTTCAAGCCCCAGG

TAGAAGTGTTCCCTGCCTCATTACTGGGAAGCAGCATCCACACAGGGGCTAACGCAGCCT

GGGACCCTGTGTGCCAGCACTTACTCTTTTGTGCAGCACATGTGACAATGAAGGACGGAT

GTATCACCTTGGTGGTTGTGGTGTTGGGGTCCTGATTCCAGCATTCATGAGTCAGGGGAA

GGTCCCTGCTAAGGACAGACCTTAGGAGGGCAGTTGGTCCAGGACCCACACTTGCTTTCC

TCGTGTTTCCTGATCCTGCCTTGGGTCTGTAGTCATACTTCTGGAAATTCCTTTTGGGTC

CAAGACGAGGAGGTTCCTCTAAGATCTCATGGCCCTGCTTCCTCCCAGTCCCCTCACAGG

GCATTTTCTTCCCACAGGTGGAAAAGGAGGGAGCTACTCTCAGGCTGCGTGTAAGTGATG

GGGGCGGGAGTGTGGAGGAGCTCACCCACCCCATAATTCCTCCTGTCCCACGTCTCCTGC

GGGCTCTGACCAGGTCCTGTTTTTGTTCTACTCCAGGCAGCGACAGTGCCCAGGGCTCTG

ATGTGTCTCTCACAGCTTGAAAAGGTGAGATTCTTGGGGTCTAGAGTGGGTGGGGTGGCA

GGTCTGGGGGTGGGTGGGGCAGTGGGGAAAGGCCTGGGTAATGGAGATTCTTTGATTGGG

ATGTTTCGCGTGTGTGGTGGGCTGTTTAGAGTGTCATCACTTACCATGACTAACCAGAAT

TTGTTCATGACTGTTGTTTTCTGTAGCCTGAGACAGCTGTCTTGTGAGGGACTGAGATGC

AGGATTTCTTCACGCCTCCCCTTTGTGACTTCAAGAGCCTCTGGCATCTCTTTCTGCAAA

GGCACCTGAATGTGTCTGCGTCCCTGTTAGCATAATGTGAGGAGGTGGAGAGACAGCCCA

CCCCCGTGTCCACCGTGACCCCTGTTCCCATGCTGACTTGTGTTTCCTCCCCAGTCATCT

TTCCTGTTCCAGAGAGGTGGGGCTGGATGTCTCCATCTCTGTCTCAACTTTATGTGCACT

GAGCTGCAACTTCTTACTTCCCTACTGAAAATAAGAATCTGAATATAAATTTGTTTTCTC

AAATATTTGCTATGAGAGGTTGATGGATTAATTAAATAAGTCAATTCCTGGAATTTGAGA

GAGCAAATAAAGACCTGAGAACCTTCCAGAATCTGCATGTTCGCTGTGCTGAGTCTGTTG

CAGGTGGGGTGTGGAGAAGGCTGTGGGGGGCCGAGTGTGGACGGGGCCTGTGCCCATTTG

GTGTTGAGTCCATCATGGGCTTTATGTGGTTAGTCGTCAGCTGGGTCACCTTCACTGCTC

CATTGTCCTTGTCCCTTCAGTGGAAACTTGTCCAGCGGGAGCTGTGACCACAGAGGCTCA

CACATCGCCCAGGGCGGCCCCTGCACACGGGGGTCTCTGTGCATTCTGAGACAAATTTTC

AGAGCCATTCACCTCCTGCCCTGCTTCTAGAGCTCCTTTTCTGCTCTGCTCTTCTGCCCT

CTCTCCCTGCCCTGGTTCTAGTGATCTTGGTGCTGAATCCAATCCCAACTCATGAATCTG

TAAAGCAGAGTCTAATTTAGACTTACATTTGTCTGTGAAATTGGACCCATCATCAAGGAC

TGTTCTTTCCTGAAGAGAGAACCTGATTGTGTGCTGCAGTGTGCTGGGGCAGGGGGTGCG

G

>HLA:HLA16059 B*51:01:01:03

GATCAGGACGAAGTCCCAGGCCCCGGGCGGGGCTCTCAGGGTCTCAGGCTCCGAGAGCCT

TGTCTGCATTGGGGAGGCGCAGCGTTGGGGATTCCCCACTCCCACGAGTTTCACTTCTTC

TCCCAACCTATGTCGGGTCCTTCTTCCAGGATACTCGTGACGCGTCCCCATTTCCCACTC

CCATTGGGTGTCGGATATCTAGAGAAGCCAATCAGTGTCGCCGGGGTCCCAGTTCTAAAG

TCCCCACGCACCCACCCGGACTCAGAATCTCCTCAGACGCCGAGATGCGGGTCACGGCGC

CCCGAACCGTCCTCCTGCTGCTCTGGGGGGCAGTGGCCCTGACCGAGACCTGGGCCGGTG

AGTGCGGGGTCGGGAGGGAAATGGCCTCTGTGGGGAGGAGCGAGGGGACCGCAGGCGGGG

GCGCAGGACCTGAGGAGCCGCGCCGGGAGGAGGGTCGGGCGGGTCTCAGCCCCTCCTCGC

CCCCAGGCTCCCACTCCATGAGGTATTTCTACACCGCCATGTCCCGGCCCGGCCGCGGGG

AGCCCCGCTTCATTGCAGTGGGCTACGTGGACGACACCCAGTTCGTGAGGTTCGACAGCG

ACGCCGCGAGTCCGAGGACGGAGCCCCGGGCGCCATGGATAGAGCAGGAGGGGCCGGAGT

ATTGGGACCGGAACACACAGATCTTCAAGACCAACACACAGACTTACCGAGAGAACCTGC

GGATCGCGCTCCGCTACTACAACCAGAGCGAGGCCGGTGAGTGACCCCGGCCCGGGGCGC

AGGTCACGACTCCCCATCCCCCACGTACGGCCCGGGTCGCCCCGAGTCTCCGGGTCCGAG

ATCCGCCTCCCTGAGGCCGCGGGACCCGCCCAGACCCTCGACCGGCGAGAGCCCCAGGCG

CGTTTACCCGGTTTCATTTTCAGTTGAGGCCAAAATCCCCGCGGGTTGGTCGGGGCGGGG

CGGGGCTCGGGGGACGGTGCTGACCGCGGGGCCGGGGCCAGGGTCTCACACTTGGCAGAC

GATGTATGGCTGCGACGTGGGGCCGGACGGGCGCCTCCTCCGCGGGCATAACCAGTACGC

CTACGACGGCAAAGATTACATCGCCCTGAACGAGGACCTGAGCTCCTGGACCGCGGCGGA

CACCGCGGCTCAGATCACCCAGCGCAAGTGGGAGGCGGCCCGTGAGGCGGAGCAGCTGAG

AGCCTACCTGGAGGGCCTGTGCGTGGAGTGGCTCCGCAGACACCTGGAGAACGGGAAGGA

GACGCTGCAGCGCGCGGGTACCAGGGGCAGTGGGGAGCCTTCCCCATCTCCTATAGGTCG

CCGGGGATGGCCTCCCACGAGAAGAGGAGGAAAATGGGATCAGCGCTAGAATGTCGCCCT

CCCTTGAATGGAGAATGGCATGAGTTTTCCTGAGTTTCCTCTGAGGGCCCCCTCTTCTCT

CTAGGACAATTAAGGGATGACGTCTCTGAGGAAATGGAGGGGAAGACAGTCCCTAGAATA

CTGATCAGGGGTCCCCTTTGACCCCTGCAGCAGCCTTGGGAACCGTGACTTTTCCTCTCA

GGCCTTGTTCTCTGCCTCACACTCAGTGTGTTTGGGGCTCTGATTCCAGCACTTCTGAGT

CACTTTACCTCCACTCAGATCAGGAGCAGAAGTCCCTGTTCCCCGCTCAGAGACTCGAAC

TTTCCAATGAATAGGAGATTATCCCAGGTGCCTGCGTCCAGGCTGGTGTCTGGGTTCTGT

GCCCCTTCCCCACACCAGGTGTCCTGTCCATTCTCAGGCTGGTCACATGGGTGGTCCTAG

GGTGTCCCATGAGAGATGCAAAGCGCCTGAATTTTCTGACTCTTCCCATCAGACCCCCCA

AAGACACACGTGACCCACCACCCCGTCTCTGACCATGAGGCCACCCTGAGGTGCTGGGCC

CTGGGCTTCTACCCTGCGGAGATCACACTGACCTGGCAGCGGGATGGCGAGGACCAAACT

CAGGACACTGAGCTTGTGGAGACCAGACCAGCAGGAGATAGAACCTTCCAGAAGTGGGCA

GCTGTGGTGGTGCCTTCTGGAGAAGAGCAGAGATACACATGCCATGTACAGCATGAGGGG

CTGCCGAAGCCCCTCACCCTGAGATGGGGTAAGGAGGGGGATGAGGGGTCATATCTCTTC

TCAGGGAAAGCAGGAGCCCTTCTGGAGCCCTTCAGCAGGGTCAGGGCCCCTCGTCTTCCC

CTCCTTTCCCAGAGCCATCTTCCCAGTCCACCATCCCCATCGTGGGCATTGTTGCTGGCC

TGGCTGTCCTAGCAGTTGTGGTCATCGGAGCTGTGGTCGCTACTGTGATGTGTAGGAGGA

AGAGCTCAGGTAGGGAAGGGGTGAGGGGTGGGGTCTGGGTTTTCTTGTCCCACTGGGGGT

TTCAAGCCCCAGGTAGAAGTGTTCCCTGCCTCATTACTGGGAAGCAGCATCCACACAGGG

GCTAACGCAGCCTGGGACCCTGTGTGCCAGCACTTACTCTTTTGTGCAGCACATGTGACA

ATGAAGGACGGATGTATCACCTTGATGGTTGTGGTGTTGGGGTCCTGATTTCAGCATTCA

TGAGTCAGGGGAAGGTCCCTGCTAAGGACAGACCTTAGGAGGGCAGTTGGTCCAGGACCC

ACACTTGCTTTCCTCGTGTTTCCTGATCCTGCCTTGGGTCTGTAGTCATACTTCTGGAAA

TTCCTTTTGGGTCCAAGACGAGGAGGTTCCTCTAAGATCTCATGGCCCTGCTTCCTCCCA

GTCCCCTCACAGGACATTTTCTTCCCACAGGTGGAAAAGGAGGGAGCTACTCTCAGGCTG

CGTGTAAGTGGTGGGGGTGGGAGTGTGGAGGAGCTCACCCACCCCATAATTCCTCCTGTC

CCACGTCTCCTGCGGGCTCTGACCAGGTCCTGTTTTTGTTCTACTCCAGCCAGCGACAGT

GCCCAGGGCTCTGATGTGTCTCTCACAGCTTGAAAAGGTGAGATTCTTGGGGTCTAGAGT

GGGCGGGGGGGGCGGGGAGGGGGCAGAGGGGAAAGGCCTGGGTAATGGAGATTCTTTGAT

TGGGATGTTTCGCGTGTGTCGTGGGCTGTTCAGAGTGTCATCACTTACCATGACTAACCA

GAATTTGTTCATGACTGTTGTTTTCTGTAGCCTGAGACAGCTGTCTTGTGAGGGACTGAG

ATGCAGGATTTCTTCACTCCTCCCCTTTGTGACTTCAAGAGCCTCTGGCATCTCTTTCTG

CAAAGGCACCTGAATGTGTCTGCGTCCCTGTTAGCATAATGTGAGGAGGTGGAGAGACAG

CCCACCCTTGTGTCCACTGTGACCCCTGTTCCCATGCTGACCTGTGTTTCCTCCCCAGTC

ATCTTTCTTGTTCCAGAGAGGTGGGGCTGGATGTCTCCATCTCTGTCTCAACTTTATGTG

CACTGAGCTGCAACTTCTTACTTCCCTACTGAAAATAAGAATCTGAATATACATTTGTTT

TCTCAAATATTTGCTATGAGAGGTTGATGGATTAATTAAATAAGTCAATTCCTGGAATGT

GAGAGAGCAAATAAAGACCTGAGAACCTTCCAGAATCTGCATGTTCGCTGTGCTGAGTCT

ATTGCAGGTGGGGTGTGGAGAAGGCTGTGGGGGGCCGAGTGTGGACAGGGCCTGTGCCCA

GTTGTTGTTGAGCCCATCATGGGCTTTATGTGGTTAGTCCTCAGCTGGGTCACCTTCACT

GCCCCATTGTCCTTGTCCCTTCAGCGGAAACTTGTCCAGTGGGAGCTGTGACCACAGAGG

CTCACACATCGCCCAGGGTGGCCCCTGCACACGGGGGTCTCTGTGCATTCTGAGACAAAT

TTTCAGAGCCATTCACCTCCTGCCCTGCTTCTAGAGCTCCTTTTCTGCTCTGCTCTCCTG

CCCTCTCTCCCTGCCCTGGTTCTAGTGATCTTGGTGCTGAATCCAATCCCAACTCATGAA

TCTGTAAAGCAGAGTCTAATTTAGAGTTACATTTGTCTGTGAAATTGGACCCATCATCAA

GGACTGTTCTTTCCTGAAGAGAGAACCTGATTGTGTGCTGCAGTGTGCTGGGGCAGGGGG

TGCGG

>HLA:HLA00381 B*57:01:01:01

GATCAGGACGAAGTCCCAGGTCCCGGACGGGGCTCTCAGGGTCTCAGGCTCCGAGAGCCT

TGTCTGCATTGGGGAGGCGCAGCGTTGGGGATTCCCCACTCCCACGAGTTTCACTTCTTC

TCCCAACCTGTGTCGGGTCCTTCTTCCAGGATACTCGTGACGCGTCCCCATTTCCCACTC

CCATTGGGTGTCGGGTGTCTAGAGAAGCCAATCAGCGTCGCCGCGGTCCCAGTTCTAAAG

TCCCCACGCACCCACCCGGACTCAGAATCTCCTCAGACGCCGAGATGCGGGTCACGGCAC

CCCGAACCGTCCTCCTGCTGCTCTGGGGGGCAGTGGCCCTGACCGAGACCTGGGCCGGTG

AGTGCGGGTCGGCAGGGAAATGGCCTCTGTAGGGAGGAGCAAGGGGACCGCAGGCGGGGG

CGCAGGACCCGGGGAGCCGCGCCGGGAGGAGGGTCGGGCGGGTCTCAGCCCCTCCTCGCC

CCCAGGCTCCCACTCCATGAGGTATTTCTACACCGCCATGTCCCGGCCCGGCCGCGGGGA

GCCCCGCTTCATCGCAGTGGGCTACGTGGACGACACCCAGTTCGTGAGGTTCGACAGCGA

CGCCGCGAGTCCGAGGATGGCGCCCCGGGCGCCATGGATAGAGCAGGAGGGGCCGGAGTA

TTGGGACGGGGAGACACGGAACATGAAGGCCTCCGCGCAGACTTACCGAGAGAACCTGCG

GATCGCGCTCCGCTACTACAACCAGAGCGAGGCCGGTGAGTGACCCCGGCCCGGGGCGCA

GGTCACGACTCCCCATCCCCCACGTACGGCCCGGGTCGCCCCGAGTCTCCGGGTCCGAGA

TCCACCCCCCTGAGGCCGCGGGACCCGCCCAGACCCTCGACCGGCGAGAGCCCCAGGCGC

GTTTACCCGGTTTCATTTTCAGTTGAGGCCAAAATCCCCGCGGGTTGGTCAGGGCGGGGC

GGGGCTCGGGGGGACGGGGCTGACCGCGGGGCCGGGGCCAGGGTCTCACATCATCCAGGT

GATGTATGGCTGCGACGTGGGGCCGGACGGGCGCCTCCTCCGCGGGCATGACCAGTCCGC

CTACGACGGCAAGGATTACATCGCCCTGAACGAGGACCTGAGCTCCTGGACCGCGGCGGA

CACGGCGGCTCAGATCACCCAGCGCAAGTGGGAGGCGGCCCGTGTGGCGGAGCAGCTGAG

AGCCTACCTGGAGGGCCTGTGCGTGGAGTGGCTCCGCAGATACCTGGAGAACGGGAAGGA

GACGCTGCAGCGCGCGGGTACCAGGGGCAGTGGGGAGCCTTCCCCATCTCCTATAGGTCG

CCGGGGATGGCCTCCCACGAGAAGAGGAGGAAAATGGGATCAGCGCTAGAATGTCGCCCT

CCCTTGAATGGAGAATGGCATGAGTTTTCCTGAGTTTCCTCTGAGGGCCCCCTCTTCTCT

CTAGGACAATTAAGGGATGACGTCTCTGAGGAAATGGAGGGGAAGACAGTCCCTAGAATA

CTGATCAGGGGTCCCCTTTGACCCCTGCAGCAGCCTTGGGAACCGTGACTTTTCCTCTCA

GGCCTTGTTCTCTGCCTCACACTCAGTGTGTTTGGGGCTCTGATTCCAGCACTTCTGAGT

CACTTTACCTCCACTCAGATCAGGAGCAGAAGTCCCTGTTCCCCGCTCAGAGACTCGAAC

TTTCCAATGAATAGGAGATTATCCCAGGTGCCTGCGTCCAGGCTGGTGTCTGGGTTCTGT

GCCCCTTCCCCACCCCAGGTGTCCTGCCCATTCTCAGGCTGGTCACATGGGTGGTCCTAG

GGTGTCCCATGAGAGATGCAAAGCGCCTGAATTTTCTGACTCTTCCCATCAGACCCCCCA

AAGACACATGTGACCCACCACCCCATCTCTGACCATGAGGCCACCCTGAGGTGCTGGGCC

CTGGGCTTCTACCCTGCGGAGATCACACTGACCTGGCAGCGGGATGGCGAGGACCAAACT

CAGGACACCGAGCTTGTGGAGACCAGACCAGCAGGAGATAGAACCTTCCAGAAGTGGGCA

GCTGTGGTGGTGCCTTCTGGAGAAGAGCAGAGATACACATGCCATGTACAGCATGAGGGG

CTGCCAAAGCCCCTCACCCTGAGATGGGGTAAGGAGGGGGATGAGGGGTCATATCTCTTC

TCAGGGAAAGCAGGAGCCCTTCTGGAGCCCTTCAGCAGGGTCAGGGCCCCTCATCTTCCC

CTCCTTTCCCAGAGCCATCTTCCCAATCCACCGTCCCCATCGTGGGCATTGTTGCTGGCC

TGGCTGTCCTAGCAGTTGTGGTCATCGGAGCTGTGGTCGCTGCTGTGATGTGTAGGAGGA

AGAGCTCAGGTAGGGAAGGGGTGAGGGGTGGGGTCTGGGTTTTCTTGTCCCACTGGGGGT

TTCAAGCCCCAGGTAGAAGTGTTCCCTGCCTCATTACTGGGAAGCAGCATGCACACAGGG

GCTAACGCAGCCTGGGACCCTGTGTGCCAGCACTTACTCTTTTGTGCAGCACATGTGACA

ATGAAGGACGGATGTATCACCTCGATGGTTGTGGTGTTGGGGTCCTGATTCCAGCATTCA

TGAGTCAGGGGAAGGTCCCTGCTAAGGACAGACCTTAGGAGAGCAGTTGGTCCAGGACCC

ACACTTGCTTTCCTCGTGTTTCCTGATCCTGCCCTGGGTCTGTAGTCATACTTCTGGAAA

TTCCTTTTGGGTCCAAGACGAGGAGGTTCCTCTAAGATCTCATGGCCCTGCTTCCTCCCA

GTCCCCTCACAGGACATTTTCTTCCCACAGGTGGAAAAGGAGGGAGCTACTCTCAGGCTG

CGTGTAAGTGGTGGGGGTGGGAGTGTGGAGGAGCTCACCCACCCCATAATTCCTCCTGTC

CCACGTCTCCTGCGGGCTCTGACCAGGTCCTGTTTTTGTTCTACTCCAGGCAGCGACAGT

GCCCAGGGCTCTGATGTGTCTCTCACAGCTTGAAAAGGTGAGATTCTTGGGGTCTAGAGT

GGGTGGGGGTGGCGGGTCTGGGGCGGGGTGGGGCAGAGGGGAAAGGCCTGGGTAATGGAG

ATTCTTTGATTGGGATGTTTCGCGTGTGTGGTGGGCTGTTCAGAGTGTCATCACTTACCA

TGACTCACCAGAATTTGTTCATGACTGTTGTTTTCTGTAGCCTGAGACAGCTGTCTTGTG

AGGGACTGAGATGCAGGATTTCTTCACGCCTCCCCTTTGTGACTTCAAGAGCCTCTGGCA

TCTCTTTCTGCAAAGGCACCTGAATGTGTCTGCGTCCCTGTTAGCCTAATGTGAGGAGGT

GGAGAGACAGCCCAACCTTGTGTCCACTGTGACCCCTGTTCCCATGCTGACCTGTGTTTC

CTCCCCAGTCATCTTTCTTGTTCCAGAGAGGTGGGGCTGGATGTCTCCATCTCTGTCTCA

ACTTTATGTGCACTGAGCTGCAACTTCTTACTTCCCTGCTGAAAATAAGAATCTGAATAT

CAATTTGTTTTCTCAAATATTTGCTATGAGAGGTTGATGGATTAATTAAATAAGTCAATT

CCTGGAATTTGAGAGAGCAAATAAAGACCTGAGAACCTTCCAGAATCTGCATGTTCGCTG

TGCTGAGTCTGTTGCAGGTGGGGTGTGGAGAAGGCTGTGGGGGGCCGAGTGTGGACGGGG

CCTGTGCCCATTTGGTGTTGAGTCCATCATGGGCTTTATGTGGTTAGTCCTCAGCTGGGT

CACCTTCACTGCTCCATTGTCCTTGTCCCTTCAGTGGAAACTTGTCCAGCGGGAGCTGTG

ACCACAGAGGCTCACACATCGCCCAGGGCGGCCCCTGCACACGGGGGTCTCTGTGCATTC

TGAGACAAATTTTCAGAGCCATTCACCTCCTGCCCTGCTTCTAGAGCTCCTTTTCTGCTC

TGCTCTTCTGCCCTCTCTCCCTGCCCTGGTTCTAGTGATCTTGGTGCTGAATCCAATCCC

AACTCATGAATCTGTAAAGCAGAGTCTAATTTAGAGTTACATTTGTCTGTGAAATTGGAC

CCGTCATCAAGGACTGTTCTTTCCTGAAGAGAGAACCTGATTGTGTGCTGCAGTGTGCTG

GGGCAGGGGGTGCGG

>HLA:HLA00427 C*05:01:01:01

TTATTTTGCTGGATGTAGTTTAATATTACCTGAGGTAAGGTAAGGCAAAGAGTGGGAGGC

AGGGAGTCCAGTTCAGGGACGGGGATTCCAGGAGAAGTGAAGGGGAAGGGGCTGGGCGCA

GCCTGGGGGTCTCTCCCTGGTTTCCACAGACAGATCCTTGGCCAGGACTCAGGCACACAG

TGTGACAAAGATGCTTGGTGTAGGAGAAGAGGGATCAGGACGAAGTCCCAGGTCCCGGGC

GGGGCTCTCAGGGTCTCAGGCTCCAAGGGCCGTGTCTGCACTGGGGAGGCGCCGCGTTGA

GGATTCTCCACTCCCCTGAGTTTCACTTCTTCTTCCAACCTGCGTCGGGTCCTTCTTCCT

GAATACTCATGACGCGTCCCCAATTCCCACTCCCATTGGGTGTCGGGTTCTAGAGAAGCC

AATCAGCGTCTCCGCAGTCCCGGTTCTAAAGTCCCCAGTCACCCACCCGGACTCGGATTC

TCCCCAGACGCCGAGATGCGGGTCATGGCGCCCCGAACCCTCATCCTGCTGCTCTCGGGA

GCCCTGGCCCTGACCGAGACCTGGGCCTGTGAGTGCGAGGTTGGGAGGGAAACGGCCTCT

GCGGAGAGGAGCGAGGGGCCCGCCCGGCGAGGGCGCAGGACCCGGGGAGCCGCGCAGGGA

GGAGGGTCGGGCGGGTCTCAGCCCCTCCTCGCCCCCAGGCTCCCACTCCATGAGGTATTT

CTACACCGCCGTGTCCCGGCCCGGCCGCGGAGAGCCCCGCTTCATCGCAGTGGGCTACGT

GGACGACACGCAGTTCGTGCAGTTCGACAGCGACGCCGCGAGTCCAAGAGGGGAGCCGCG

GGCGCCGTGGGTGGAGCAGGAGGGGCCGGAGTATTGGGACCGGGAGACACAGAAGTACAA

GCGCCAGGCACAGACTGACCGAGTGAACCTGCGGAAACTGCGCGGCTACTACAACCAGAG

CGAGGCCGGTGAGTGACCCCGGCCCGGGGCGCAGGTCACGACCCCTCCCCATCCCCCACG

GACGGCCCGGGTCGCCCCGAGTCTCCCGGTCTGAGATCCACCCCGAGGCTGCGGAACCCG

CCCAGACCCTCGACCGGAGAGAGCCCCAGTCACCTTTACCCGGTTTCATTTTCAGTTTAG

GCCAAAATCCCCGCGGGTTGGTCGGGGCTGGGGCGGGGCTCGGGGGACGGGGCTGACCAC

GGGGGCGGGGCCAGGGTCTCACACCCTCCAGAGGATGTATGGCTGCGACCTGGGGCCCGA

CGGGCGCCTCCTCCGCGGGTATAACCAGTTCGCCTACGACGGCAAGGATTACATCGCCCT

GAATGAGGACCTGCGCTCCTGGACCGCCGCGGACAAGGCGGCTCAGATCACCCAGCGCAA

GTGGGAGGCGGCCCGTGAGGCGGAGCAGCGGAGAGCCTACCTGGAGGGCACGTGCGTGGA

GTGGCTCCGCAGATACCTGGAGAACGGGAAGAAGACGCTGCAGCGCGCGGGTACCAGGGG

CAGTGGGGAGCCTTCCCCATCTCCTGTAGATCTCCCGGGATGGCCTCCCACGAGGAGGGG

AGGAAAATGGGATCAGCGCTGGAATATCGCCCTCCCTTGAATGGAGAATGGGATGAGTTT

TCCTGAGTTTCCTCTGAGGGCCCCCTCTGCTCTCTAGGACAATTAAGGGATGAAGTCCTT

GAGGAAATGGAGGGGAAGACAGTCCCTGGAATACTGATCAGGGGTCCCCTTTGACCACTT

TGACCACTGCAGCAGCTGTGGTCAGGCTGCTGACCTTTCTCTCAGGCCTTGTTCTCTGCC

TCACGCTCAATGTGTTTAAAGGTTTGATTCCAGCTTTTCTGAGTCCTTCGGCCTCCACTC

AGGTCAGGACCAGAAGTCGCTGTTCCTCCCTCAGAGACTAGAACTTTCCAATGAATAGGA

GATTATCCCAGGTGCCTGTGTCCAGGCTGGCGTCTGGGTTCTGTGCCCCCTTCCCCACCC

CAGGTGTCCTGTCCATTCTCAGGATGGTCACATGGGCACTGTTGGAGTGTCGCAAGAGAG

ATACAAAGTGTCTGAATTTTCTGACTCTTCCCGTCAGAACACCCAAAGACACACGTGACC

CACCATCCCGTCTCTGACCATGAGGCCACCCTGAGGTGCTGGGCCCTGGGCTTCTACCCT

GCGGAGATCACACTGACCTGGCAGCGGGATGGCGAGGACCAAACTCAGGACACCGAGCTT

GTGGAGACCAGGCCAGCAGGAGATGGAACCTTCCAGAAGTGGGCAGCTGTGGTGGTGCCT

TCTGGAGAAGAGCAGAGATACACGTGCCATGTGCAGCACGAGGGGCTGCCAGAGCCCCTC

ACCCTGAGATGGGGTAAGGAGGGGGATGAGGGGTCATGTGTCTTCTCAGGGAAAGCAGAA

GTCCTGGAGCCCTTCAGCCGGGTCAGGGCTGAGGCTTGGGGGTCAGGGCCCCTCACCTTC

CCCTCCTTTCCCAGGGCCATCTTCCCAGCCCACCATCCCCATCGTGGGCATCGTTGCTGG

CCTGGCTGTCCTGGCTGTCCTAGCTGTCCTAGGAGCTGTGATGGCTGTTGTGATGTGTAG

GAGGAAGAGCTCAGGTAGGGAAGGGGTGAGGAGTGGGGTCTGGGTTTTCTTGTCCCACTG

GGAGTTTCAAGCCCCAGGTAGAAGTGTGCCCCACCTCGTTACTGGAAGCACCATCCACAC

ATGGGCCATCCCAGCCTGGGACCCTGTGTGCTAGCACTTACTCTGTTGTGAAGCACATGA

CAATGAAGGACAGATGTATCACCTTGATGATTATGGTGTTGGGGTCCTTGATTCCAGCAT

TCATGAGTCAGGGGAAGGTCCCTGCTAAGGACAGACCTTAGGAGGGCAGTTGCTCCAGAA

CCCACAGCTGCTTTCCCCGTGTTTCCTGATCCTGCCCTGGGTCTGCAGTCATAGTTCTGG

AAACTTCTCTTGGGTCCAAGACTAGGAGGTTCCCCTAAGATCGCATGGCCCTGCCTCCTC

CCTGTCCCCTCACAGGGCATTTTCTTCCCACAGGTGGAAAAGGAGGGAGCTGCTCTCAGG

CTGCGTGTAAGTGATGGCGGTGGGCGTGTGGAGGAGCTCACCCACCCCATAATTCCTCTT

GTCCCACATCTCCTGCGGGCTCTGACCAGGTCTTTTTTTTTGTTCTACCCCAGCCAGCAA

CAGTGCCCAGGGCTCTGATGAGTCTCTCATCGCTTGTAAAGGTGAGATTCTGGGGAGCTG

AAGTGGTCGGGGGTGGGGCAGAGGGAAAAGGCCTAGGTAATGGGGATCCTTTGATTGGGA

CGTTTCGAATGTGTGGTGAGCTGTTCAGAGTGTCATCACTTACCATGACTGACCTGAATT

TGTTCATGACTATTGTGTTCTGTAGCCTGAGACAGCTGCCTGTGTGGGACTGAGATGCAG

GATTTCTTCACACCTTTCCTTTGTGACTTCAAGAGCCTCTGGCATCTCTTTCTGCAAAGG

CATCTGAATGTGTCTGCGTTCCTGTTAGCATAATGTGAGGAGGTGGAGAGACAGCCCACC

CCCGTGTCCACCGTGACCCCTGTCCCCACACTGACCTGTGTTCCCTCCCCGATCATCTTT

CCTGTTCCAGAGAAGTGGGCTGGATGTCTCCATCTCTGTCTCAACTTTACGTGTACTGAG

CTGCAACTTCTTACTTCCCTACTGAAAATAAGAATCTGAATATAAATTTGTTTTCTCAAA

TATTTGCTATGAGAGGTTGATGGATTAATTAAATAAGTCAATTCCTGGAAGTTGAGAGAG

CAAATAAAGACCTGAGAACCTTCCAGAATCCGCATGTTCGCTGTGCTGAGTCTGTTGCAG

GTGGGGGTGGGGAAGGCTGTGAGGAGACGAGTGTGGACGGGGCCTGTGCCTAGTTGCTGT

TCAGTTCTTCATGGGCTTTATGTGGTCAGTCCTCAGCTGGGTCACCTTCACTGCTCCATT

GTCCTTGTCCCTTCAGTGGAAACTTGTCCAGCGGGAGCTGTGACCACAGAGGCTCACACA

TCGCCCAGGGCAGCCCCTGCACACGGGAGTCCCTGTGCTTTCTGAGACAAATTTTCAGAC

CCATTCAGCTCCTGCCCTCCTTCTAGGGCTCCTCTTCTGCTTTGGTCTCCTGCCCTCTCT

CCCTTCCCTGATTCCAGTAATCTTCGTGCTGACTCCAATCCCAACTCATGAATCTAAAGC

AGAGCCTAATTTAGATTTATATTTGTTTGTAAAATTGGGTCCATAGTCTAGAATTGTTCC

TTCCTGAAGAGAGAAACCTGATTGTGTGCTGCAGTGTGCGGGG

>HLA:HLA03586 C*05:01:01:02

TTATTTTGCTGGATGTAGTTTAATATTACCTGAGGTAAGGTAAGGCAAAGAGTGGGAGGC

AGGGAGTCCAGTTCAGGGACGGGGATTCCAGGAGAAGTGAAGGGGAAGGGGCTGGGCGCA

GCCTGGGGGTCTCTCCCTGGTTTCCACAGACAGATCCTTGGCCAGGACTCAGGCACACAG

TGTGACAAAGATGCTTGGTGTAGGAGAAGAGGGATCAGGACGAAGTCCCAGGTCCCGGGC

GGGGCTCTCAGGGTCTCAGGCTCCAAGGGCCGTGTCTGCACTGGGGAGGCGCCGCGTTGA

GGATTCTCCACTCCCCTGAGTTTCACTTCTTCTTCCAACCTGCGTCGGGTCCTTCTTCCT

GAATACTCATGACGCGTCCCCAATTCCCACTCCCATTGGGTGTCGGGTTCTAGAGAAGCC

AATCAGCGTCTCCGCAGTCCCGGTTCTAAAGTCCCCAGTCACCCACCCGGACTCGGATTC

TCCCCAGACGCCGAGATGCGGGTCATGGCGCCCCGAACCCTCATCCTGCTGCTCTCGGGA

GCCCTGGCCCTGACCGAGACCTGGGCCTGTGAGTGCGAGGTTGGGAGGGAAACGGCCTCT

GCGGAGAGGAGCGAGGGGCCCGCCCGGCGAGGGCGCAGGACCCGGGGAGCCGCGCAGGGA

GGAGGGTCGGGCGGGTCTCAGCCCCTCCTCGCCCCCAGGCTCCCACTCCATGAGGTATTT

CTACACCGCCGTGTCCCGGCCCGGCCGCGGAGAGCCCCGCTTCATCGCAGTGGGCTACGT

GGACGACACGCAGTTCGTGCAGTTCGACAGCGACGCCGCGAGTCCAAGAGGGGAGCCGCG

GGCGCCGTGGGTGGAGCAGGAGGGGCCGGAGTATTGGGACCGGGAGACACAGAAGTACAA

GCGCCAGGCACAGACTGACCGAGTGAACCTGCGGAAACTGCGCGGCTACTACAACCAGAG

CGAGGCCGGTGAGTGACCCCGGCCCGGGGCGCAGGTCACGACCCCTCCCCATCCCCCACG

GACGGCCCGGGTCGCCCCGAGTCTCCCGGTCTGAGATCCACCCCGAGGCTGCGGAACCCG

CCCAGACCCTCGACCGGAGAGAGCCCCAGTCACCTTTACCCGGTTTCATTTTCAGTTTAG

GCCAAAATCCCCGCGGGTTGGTCGGGGCTGGGGCGGGGCTCGGGGGACGGGGCTGACCAC

GGGGGCGGGGCCAGGGTCTCACACCCTCCAGAGGATGTATGGCTGCGACCTGGGGCCCGA

CGGGCGCCTCCTCCGCGGGTATAACCAGTTCGCCTACGACGGCAAGGATTACATCGCCCT

GAATGAGGACCTGCGCTCCTGGACCGCCGCGGACAAGGCGGCTCAGATCACCCAGCGCAA

GTGGGAGGCGGCCCGTGAGGCGGAGCAGCGGAGAGCCTACCTGGAGGGCACGTGCGTGGA

GTGGCTCCGCAGATACCTGGAGAACGGGAAGAAGACGCTGCAGCGCGCGGGTACCAGGGG

CAGTGGGGAGCCTTCCCCATCTCCTGTAGATCTCCCGGGATGGCCTCCCACGAGGAGGGG

AGGAAAATGGGATCAGCGCTGGAATATCGCCCTCCCTTGAATGGAGAATGGGATGAGTTT

TCCTGAGTTTCTTCTGAGGGCCCCCTCTGCTCTCTAGGACAATTAAGGGATGAAGTCCTT

GAGGAAATGGAGGGGAAGACAGTCCCTGGAATACTGATCAGGGGTCCCCTTTGACCACTT

TGACCACTGCAGCAGCTGTGGTCAGGCTGCTGACCTTTCTCTCAGGCCTTGTTCTCTGCC

TCACGCTCAATGTGTTTAAAGGTTTGATTCCAGCTTTTCTGAGTCCTTCGGCCTCCACTC

AGGTCAGGACCAGAAGTCGCTGTTCCTCCCTCAGAGACTAGAACTTTCCAATGAATAGGA

GATTATCCCAGGTGCCTGTGTCCAGGCTGGCGTCTGGGTTCTGTGCCCCCTTCCCCACCC

CAGGTGTCCTGTCCATTCTCAGGATGGTCACATGGGCACTGTTGGAGTGTCGCAAGAGAG

ATACAAAGTGTCTGAATTTTCTGACTCTTCCCGTCAGAACACCCAAAGACACACGTGACC

CACCATCCCGTCTCTGACCATGAGGCCACCCTGAGGTGCTGGGCCCTGGGCTTCTACCCT

GCGGAGATCACACTGACCTGGCAGCGGGATGGCGAGGACCAAACTCAGGACACCGAGCTT

GTGGAGACCAGGCCAGCAGGAGATGGAACCTTCCAGAAGTGGGCAGCTGTGGTGGTGCCT

TCTGGAGAAGAGCAGAGATACACGTGCCATGTGCAGCACGAGGGGCTGCCAGAGCCCCTC

ACCCTGAGATGGGGTAAGGAGGGGGATGAGGGGTCATGTGTCTTCTCAGGGAAAGCAGAA

GTCCTGGAGCCCTTCAGCCGGGTCAGGGCTGAGGCTTGGGGGTCAGGGCCCCTCACCTTC

CCCTCCTTTCCCAGGGCCATCTTCCCAGCCCACCATCCCCATCGTGGGCATCGTTGCTGG

CCTGGCTGTCCTGGCTGTCCTAGCTGTCCTAGGAGCTGTGATGGCTGTTGTGATGTGTAG

GAGGAAGAGCTCAGGTAGGGAAGGGGTGAGGAGTGGGGTCTGGGTTTTCTTGTCCCACTG

GGAGTTTCAAGCCCCAGGTAGAAGTGTGCCCCACCTCGTTACTGGAAGCACCATCCACAC

ATGGGCCATCCCAGCCTGGGACCCTGTGTGCTAGCACTTACTCTGTTGTGAAGCACATGA

CAATGAAGGACAGATGTATCACCTTGATGATTATGGTGTTGGGGTCCTTGATTCCAGCAT

TCATGAGTCAGGGGAAGGTCCCTGCTAAGGACAGACCTTAGGAGGGCAGTTGCTCCAGAA

CCCACAGCTGCTTTCCCCGTGTTTCCTGATCCTGCCCTGGGTCTGCAGTCATAGTTCTGG

AAACTTCTCTTGGGTCCAAGACTAGGAGGTTCCCCTAAGATCGCATGGCCCTGCCTCCTC

CCTGTCCCCTCACAGGGCATTTTCTTCCCACAGGTGGAAAAGGAGGGAGCTGCTCTCAGG

CTGCGTGTAAGTGATGGCGGTGGGCGTGTGGAGGAGCTCACCCACCCCATAATTCCTCTT

GTCCCACATCTCCTGCGGGCTCTGACCAGGTCTTTTTTTTTGTTCTACCCCAGCCAGCAA

CAGTGCCCAGGGCTCTGATGAGTCTCTCATCGCTTGTAAAGGTGAGATTCTGGGGAGCTG

AAGTGGTCGGGGGTGGGGCAGAGGGAAAAGGCCTAGGTAATGGGGATCCTTTGATTGGGA

CGTTTCGAATGTGTGGTGAGCTGTTCAGAGTGTCATCACTTACCATGACTGACCTGAATT

TGTTCATGACTATTGTGTTCTGTAGCCTGAGACAGCTGCCTGTGTGGGACTGAGATGCAG

GATTTCTTCACACCTTTCCTTTGTGACTTCAAGAGCCTCTGGCATCTCTTTCTGCAAAGG

CATCTGAATGTGTCTGCGTTCCTGTTAGCATAATGTGAGGAGGTGGAGAGACAGCCCACC

CCCGTGTCCACCGTGACCCCTGTCCCCACACTGACCTGTGTTCCCTCCCCGATCATCTTT

CCTGTTCCAGAGAAGTGGGCTGGATGTCTCCATCTCTGTCTCAACTTTACGTGTACTGAG

CTGCAACTTCTTACTTCCCTACTGAAAATAAGAATCTGAATATAAATTTGTTTTCTCAAA

TATTTGCTATGAGAGGTTGATGGATTAATTAAATAAGTCAATTCCTGGAAGTTGAGAGAG

CAAATAAAGACCTGAGAACCTTCCAGAATCCGCATGTTCGCTGTGCTGAGTCTGTTGCAG

GTGGGGGTGGGGAAGGCTGTGAGGAGACGAGTGTGGACGGGGCCTGTGCCTAGTTGCTGT

TCAGTTCTTCATGGGCTTTATGTGGTCAGTCCTCAGCTGGGTCACCTTCACTGCTCCATT

GTCCTTGTCCCTTCAGTGGAAACTTGTCCAGCGGGAGCTGTGACCACAGAGGCTCACACA

TCGCCCAGGGCAGCCCCTGCACACGGGAGTCCCTGTGCTTTCTGAGACAAATTTTCAGAC

CCATTCAGCTCCTGCCCTCCTTCTAGGGCTCCTCTTCTGCTTTGGTCTCCTGCCCTCTCT

CCCTTCCCTGATTCCAGTAATCTTCGTGCTGACTCCAATCCCAACTCATGAATCTAAAGC

AGAGCCTAATTTAGATTTATATTTGTTTGTAAAATTGGGTCCATAGTCTAGAATTGTTCC

TTCCTGAAGAGAGAAACCTGATTGTGTGCTGCAGTGTGCGGGG

>HLA:HLA00430 C*06:02:01:01

TTATTTTGCTGGATGTAGTTTAATATTACCTGAGGTAAGGTAAGGCAAAGAGTGGGAGGC

AGGGAGTCCAGTTCAGGGACGGGGATTCCAGGAGAAGTGAAGGGGAAGGGGCTGGGCGCA

GCCTGGGGGTCTCTCCCTGGTTTCCACAGACAGATCCTTGGCCAGGACTCAGGCACACAG

TGTGACAAAGATGCTTGGTGTAGGAGAAGAGGGATCAGGACGAAGTCCCAGGTCCCGGGC

GGGGCTCTCAGGGTCTCAGGCTCCAAGGGCCGTGTCTGCACTGGGGAGGCGCCGCGTTGA

GGATTCTCCACTCCCCTGAGTTTCACTTCTTCTCCCAACCTGCGTCGGGTCCTTCTTCCT

GAATACTCATGACGCGTCCCCAATTCCCACTCCCATTGGGTGTCGGGTTCTAGAGAAGCC

AATCAGCGTCTCCGCAGTCCCGGTTCTAAAGTCCCCAGTCACCCACCCGGACTCGGATTC

TCCCCAGACGCCGAGATGCGGGTCATGGCGCCCCGAACCCTCATCCTGCTGCTCTCGGGA

GCCCTGGCCCTGACCGAGACCTGGGCCTGTGAGTGCGGGGTTGGGAGGGAAACGGCCTCT

GCGGAGAGGAGCGAGGGGCCCGCCCGGCGAGGGCGCAGGACCCGGGGAGCCGCGCAGGGA

GGTGGGTCGGGCGGGTCTCAGCCCCTCCTCGCCCCCAGGCTCCCACTCCATGAGGTATTT

CGACACCGCCGTGTCCCGGCCCGGCCGCGGAGAGCCCCGCTTCATCTCAGTGGGCTACGT

GGACGACACGCAGTTCGTGCGGTTCGACAGCGACGCCGCGAGTCCGAGAGGGGAGCCCCG

GGCGCCGTGGGTGGAGCAGGAGGGGCCGGAGTATTGGGACCGGGAGACACAGAAGTACAA

GCGCCAGGCACAGGCTGACCGAGTGAACCTGCGGAAACTGCGCGGCTACTACAACCAGAG

CGAGGACGGTGAGTGACCCCGGCCCGGGGCGCAGGTCACGACCCCTCCCCATCCCCCACG

GACGGCCCGGGTCGCCCCGAGTCTCCCGGTCTGAGATCCACCCCGAGGCTGCGGAACCCG

CCCAGACCCTCGACCGGAGAGAGCCCCAGTCACCTTTACCCGGTTTCATTTTCAGTTTAG

GCCAAAATCCCCGCGGGTTGGTCGGGGCTGGGGCGGGGCTCGGGGGACGGGGCTGACCAC

GGGGGCGGGGCCAGGGTCTCACACCCTCCAGTGGATGTATGGCTGCGACCTGGGGCCCGA

CGGGCGCCTCCTCCGCGGGTATGACCAGTCCGCCTACGACGGCAAGGATTACATCGCCCT

GAACGAGGACCTGCGCTCCTGGACCGCCGCGGACACGGCGGCTCAGATCACCCAGCGCAA

GTGGGAGGCGGCCCGTGAGGCGGAGCAGTGGAGAGCCTACCTGGAGGGCACGTGCGTGGA

GTGGCTCCGCAGATACCTGGAGAACGGGAAGGAGACGCTGCAGCGCGCGGGTACCAGGGG

CAGTGGGGAGCCTTCCCCATCTCCTGTAGATCTCCCGGGATGGCCTCCCACGAGGAGGGG

AGGAAAATGGGATCAGCGCTAGAATATCGCCCTCCCTTGAATGGAGAATGGGATGAGTTT

TCCTGAGTTTCCTCTGAGGGCCCCCTCTGCTCTCTAGGACAATTAAGGGATGAAGTCCTT

GAGGAAATGGAGGGGAAGACAGTCCCTGGAATACTGATCAGGGGTCCCCTTTGACCACTT

TGACCACTGCAGCAGCTGTGGTCAGGCTGCTGACCTTTCTCTCAGGCCTTGTTCTCTGCC

TCACGCTCAATGTGTTTAAAGGTTTGATTCCAGCTTTTCTGAGTCCTTCGGCCTCCACTC

AGGTCAGGACCAGAAGTCGCTGTTCCTCCCTCAGAGACTAGAACTTTCCAATGAATAGGA

GATTATCCCAGGTGCCTGTGTCCAGGCTGGCGTCTGGGTTCTGTGCCCCCTTCCCCACCC

CAGGTGTCCTGTCCATTCTCAGGATGGTCACATGGGCGCTGTTGGAGTGTCGCAAGAGAG

ATACAAAGTGTCTGAATTTTCTGACTCTTCCCGTCAGAACACCCAAAGACACACGTGACC

CACCATCCCGTCTCTGACCATGAGGCCACCCTGAGGTGCTGGGCCCTGGGCTTCTACCCT

GCGGAGATCACACTGACCTGGCAGCGGGATGGCGAGGACCAAACTCAGGACACCGAGCTT

GTGGAGACCAGGCCAGCAGGAGATGGAACCTTCCAGAAGTGGGCAGCTGTGGTGGTGCCT

TCTGGAGAAGAGCAGAGATACACGTGCCATGTGCAGCACGAGGGGCTGCCAGAGCCCCTC

ACCCTGAGATGGGGTAAGGAGGGGGATGAGGGGTCATGTGTCTTCTCAGGGAAAGCAGAA

GTCCTGGAGCCCTTCAGCCGGGTCAGGGCTGAGGCTTGGGGGTCAGGGCCCCTCACCTTC

CCCTCCTTTCCCAGAGCCATCTTCCCAGCCCACCATCCCCATCGTGGGCATCGTTGCTGG

CCTGGCTGTCCTGGCTGTCCTAGCTGTCCTAGGAGCTGTGATGGCTGTTGTGATGTGTAG

GAGGAAGAGCTCAGGTAGGGAAGGGGTGAGGAGTGGGGTCTGGGTTTTCTTGTCCCACTG

GGAGTTTCAAGCCCCAGGTAGAAGTGTGCCCCACCTCGTTACTGGAAGCACCATCCACAC

ATGGGCCATCCCAGCCTGGGACCCTGTGTGCTAGCACTTACTCTGTTGTGAAGCACATGA

CAATGAAGGACAGATGTATCACCTTGATGATTATGGTGTTGGGGTCCTTGATTCCAGCAT

TCATGAGTCAGGGGAAGGTCCCTGCTAAGGACAGACCTTAGGAGGGCAGTTGCTCCAGAA

CCCACAGCTGCTTTCCCCGTGTTTCCTGATCCTGCCCTGGGTCTGCAGTCATAGTTCTGG

AAACTTCTCTTGGGTCCAAGACTAGGAGGTTCCCCTAAGATCGCATGGCCCTGCCTCCTC

CCTGTCCCCTCACAGGGCATTTTCTTCCCACAGGTGGAAAAGGAGGGAGCTGCTCTCAGG

CTGCGTGTAAGTGATGGCGGTGGGCGTGTGGAGGAGCTCACCCACCCCATAATTCCTCTT

GTCCCACATCTCCTGCGGGCTCTGACCAGGTCTTTTTTTTTGTTCTACCCCAGCCAGCAA

CAGTGCCCAGGGCTCTGATGAGTCTCTCATCGCTTGTAAAGGTGAGATTCTGGGGAGCTG

AAGTGGTCTGGGGTGGGGCAGAGGGAAAAGGCCTAGGTAATGGGGATCCTTTGATTGGGA

CGTTTCGAATGTGTGGTGAGCTGTTCAGAGTGTCATCACTTACCATGACTGACCTGAATT

TGTTCATGACTATTGTGTTCTGTAGCCTGAGACAGCTGCCTGTGTGGGACTGAGATGCAG

GATTTCTTCACACCTCTCCTTTGTGACTTCAAGAGCCTCTGGCATCTCTTTCTGCAAAGG

CATCTGAATGTGTCTGCGTTCCTGTTAGCATAATGTGAGGAGGTGGAGAGACAGCCCACC

CCCGTGTCCACCGTGACCCCTGTCCCCACACTGACCTGTGTTCCCTCCCCGATCATCTTT

CCTGTTCCAGAGAAGTGGGCTGGATGTCTCCATCTCTGTCTCAACTTTACGTGTACTGAG

CTGCAACTTCTTACTTCCCTACTGAAAATAAGAATCTGAATATAAATTTGTTTTCTCAAA

TATTTGCTATGAGAGGTTGATGGATTAATTAAATAAGTCAATTCCTGGAAGTTGAGAGAG

CAAATAAAGACCTGAGAACCTTCCAGAATCCGCATGTTCGCTGTGCTGAGTCTGTTGCAG

GTGGGGGTGGGGAAGGCTGTGAGGAGACGAGTGTGGACGGGGCCTGTGCCTAGTTGCTGT

TCAGTTCTTCATGGGCTTTATGTGGTCAGTCCTCAGCTGGGTCACCTTCACTGCTCCATT

GTCCTTGTCCCTTCAGTGGAAACTTGTCCAGCGGGAGCTGTGACCACAGAGGCTCACACA

TCGCCCAGGGCAGCCCCTGCACACGGGAGTCCCTGTGCTTTCTGAGACAAATTTTCAGAC

CCATTCAGCTCCTGCCCTCCTTCTAGGGCTCCTCTTCTGCTTTGGTCTCCTGTCCTCTCT

CCCTTCCCTGATTCCAGTAATCTTCGTGCTGACTCCAATCCCAACTCATGAATCTAAAGC

AGAGCCTAATTTAGATTTATATTTGTTTGTAAAATTGGGTCCATAGTCTAGAATTGTTCC

TTCCTGAAGAGAGAAACCTGATCGTGTGCTGCAGTGTGCGGGG

>HLA:HLA00455 C*12:03:01:01

TTATTTTGCTGGATGTAGTTTAATATTACCTGAGGTAAGGTAAGGCAAAGAGTGGGAGGC

AGGGAGTCCAGTTCAGGGACGGGGATTCCAGGAGAAGTGAAGGGGAAGGGGCTGGGCGCA

GCCTGGGGGTCTCTCCCTGGTTTCCACAGACAGATCCTTGGCCAGGACTCAGGCACACAG

TGTGACAAAGATGCTTGGTGTAGGAGAAGAGGGATCAGGACGAAGTCCCAGGTCCCGGGC

GGGGCTCTCAGGGTCTCAGGCTCCAAGGGCCGTGTCTGCACTGGGGAGGCGCCGCGTTGA

GGATTCTCCACTCCCCTGAGTTTCACTTCTTCTCCCAACCTGCGTCGGGTCCTTCTTCCT

GAATACTCATGACGCGTCCCCAATTCCCACTCCCATTGGGTGTCGGGTTCTAGAGAAGCC

AATCAGCGTCTCCGCAGTCCCGGTTCTAAAGTCCCCAGTCACCCACCCGGACTCGGATTC

TCCCCAGACGCCGAGATGCGGGTCATGGCGCCCCGAACCCTCATCCTGCTGCTCTCGGGA

GCCCTGGCCCTGACCGAGACCTGGGCCTGTGAGTGCGGGGTTGGGAGGGAAACGGCCTCT

GCGGAGAGGAGCGAGGGGCCCGCCCGGCGAGGGCGCAGGACCCGGGGAGCCGCGCAGGGA

GGAGGGTCGGGCGGGTCTCAGCCCCTCCTCGCCCCCAGGCTCCCACTCCATGAGGTATTT

CTACACCGCCGTGTCCCGGCCCGGCCGCGGAGAGCCCCGCTTCATCGCAGTGGGCTACGT

GGACGACACGCAGTTCGTGCGGTTCGACAGCGACGCCGCGAGTCCAAGAGGGGAGCCGCG

GGCGCCGTGGGTGGAGCAGGAGGGGCCGGAGTATTGGGACCGGGAGACACAGAAGTACAA

GCGCCAGGCACAGGCTGACCGAGTGAGCCTGCGGAACCTGCGCGGCTACTACAACCAGAG

CGAGGCCGGTGAGTGACCCCGGCCCGGGGCGCAGGTCACGACCCCTCCCCATCCCCCACG

GACGGCCCGGGTCGCCCCGAGTCTCCCGGTCTGAGATCCACCCCGAGGCTGCGGAACCCG

CCCAGACCCTCGACCGGAGAGAGCCCCAGTCACCTTTACCCGGTTTCATTTTCAGTTTAG

GCCAAAATCCCCGCGGGTTGGTCGGGGCTGGGGCGGGGCTCGGGGGACGGGGCTGACCAC

GGGGGCGGGGCCAGGGTCTCACACCCTCCAGTGGATGTATGGCTGCGACCTGGGGCCCGA

CGGGCGCCTCCTCCGCGGGTATGACCAGTCCGCCTACGACGGCAAGGATTACATCGCCCT

GAACGAGGACCTGCGCTCCTGGACTGCCGCGGACACGGCGGCTCAGATCACCCAGCGCAA

GTGGGAGGCGGCCCGTGAGGCGGAGCAGTGGAGAGCCTACCTGGAGGGCACGTGCGTGGA

GTGGCTCCGCAGATACCTGGAGAACGGGAAGGAGACGCTGCAGCGCGCGGGTACCAGGGG

CAGTGGGGAGCCTTCCCCATCTCCTGTAGATCTCCCGGGATGGCCTCCCACGAGGAGGGG

AGGAAAATGGGATCAGCGCTAGAATATCGCCCTCCCTTGAATGGAGAATGGGATGAGTTT

TCCTGAGTTTCCTCTGAGGGCCCCCTCTGCTCTCTAGGACAATTAAGGGATGAAGTCCTT

GAGGAAATGGAGGGGAAGACAGTCCCTGGAATACTGATCAGGGGTCCCCTTTGACCACTT

TGACCACTGCAGCAGCTGTGGTCAGGCTGCTGACCTTTCTCTCAGGCCTTGTTCTCTGCC

TCACGCTCAATGTGTTTAAAGGTTTGATTCCAGCTTTTCTGAGTCCTTCGGCCTCCACTC

AGGTCAGGACCAGAAGTCGCTGTTCCTCCCTCAGAGACTAGAACTTTCCAATGAATAGGA

GATTATCCCAGGTGCCTGTGTCCAGGCTGGCGTCTGGGTTCTGTGCCCCCTTCCCCACCC

CAGGTGTCCTGTCCATTCTCAGGATGGTCACATGGGCGCTGTTGGAGTGTCGCAAGAGAG

ATACAAAGTGTCTGAATTTTCTGACTCTTCCCGTCAGAACACCCAAAGACACACGTGACC

CACCATCCCGTCTCTGACCATGAGGCCACCCTGAGGTGCTGGGCCCTGGGCTTCTACCCT

GCGGAGATCACACTGACCTGGCAGCGGGATGGCGAGGACCAAACTCAGGACACCGAGCTT

GTGGAGACCAGGCCAGCAGGAGATGGAACCTTCCAGAAGTGGGCAGCTGTGGTGGTGCCT

TCTGGAGAAGAGCAGAGATACACGTGCCATGTGCAGCACGAGGGGCTGCCAGAGCCCCTC

ACCCTGAGATGGGGTAAGGAGGGGGATGAGGGGTCATGTGTCTTCTCAGGGAAAGCAGAA

GTCCTGGAGCCCTTCAGCCGGGTCAGGGCTGAGGCTTGGGGGTCAGGGCCCCTCACCTTC

CCCTCCTTTCCCAGAGCCATCTTCCCAGCCCACCATCCCCATCGTGGGCATCGTTGCTGG

CCTGGCTGTCCTGGCTGTCCTAGCTGTCCTAGGAGCTGTGATGGCTGTTGTGATGTGTAG

GAGGAAGAGCTCAGGTAGGGAAGGGGTGAGGAGTGGGGTCTGGGTTTTCTTGTCCCACTG

GGAGTTTCAAGCCCCAGGTAGAAGTGTGCCCCACCTCGTTACTGGAAGCACCATCCACAC

ATGGGCCATCCCAGCCTGGGACCCTGTGTGCTAGCACTTACTCTGTTGTGAAGCACATGA

CAATGAAGGACAGATGTATCACCTTGATGATTATGGTGTTGGGGTCCTTGATTCCAGCAT

TCATGAGTCAGGGGAAGGTCCCTGCTAAGGACAGACCTTAGGAGGGCAGTTGCTCCAGAA

CCCACAGCTGCTTTCCCCGTGTTTCCTGATCCTGCCCTGGGTCTGCAGTCATAGTTCTGG

AAACTTCTCTTGGGTCCAAGACTAGGAGGTTCCCCTAAGATCGCATGGCCCTGCCTCCTC

CCTGTCCCCTCACAGGGCATTTTCTTCCCACAGGTGGAAAAGGAGGGAGCTGCTCTCAGG

CTGCGTGTAAGTGATGGCGGTGGGCGTGTGGAGGAGCTCACCCACCCCATAATTCCTCTT

GTCCCACATCTCCTGCGGGCTCTGACCAGGTCTTTTTTTTTGTTCTACCCCAGCCAGCAA

CAGTGCCCAGGGCTCTGATGAGTCTCTCATCGCTTGTAAAGGTGAGATTCTGGGGAGCTG

AAGTGGTCTGGGGTGGGGCAGAGGGAAAAGGCCTAGGTAATGGGGATCCTTTGATTGGGA

CGTTTCGAATGTGTGGTGAGCTGTTCAGAGTGTCATCACTTACCATGACTGACCTGAATT

TGTTCATGACTATTGTGTTCTGTAGCCTGAGACAGCTGCCTGTGTGGGACTGAGATGCAG

GATTTCTTCACACCTCTCCTTTGTGACTTCAAGAGCCTCTGGCATCTCTTTCTGCAAAGG

CATCTGAATGTGTCTGCGTTCCTGTTAGCATAATGTGAGGAGGTGGAGAGACAGCCCACC

CCCGTGTCCACCGTGACCCCTGTCCCCACACTGACCTGTGTTCCCTCCCCGATCATCTTT

CCTGTTCCAGAGAAGTGGGCTGGATGTCTCCATCTCTGTCTCAACTTTACGTGTACTGAG

CTGCAACTTCTTACTTCCCTACTGAAAATAAGAATCTGAATATAAATTTGTTTTCTCAAA

TATTTGCTATGAGAGGTTGATGGATTAATTAAATAAGTCAATTCCTGGAAGTTGAGAGAG

CAAATAAAGACCTGAGAACCTTCCAGAATCCGCATGTTCGCTGTGCTGAGTCTGTTGCAG

GTGGGGGTGGGGAAGGCTGTGAGGAGACGAGTGTGGACGGGGCCTGTGCCTAGTTGCTGT

TCAGTTCTTCATGGGCTTTATGTGGTCAGTCCTCAGCTGGGTCACCTTCACTGCTCCATT

GTCCTTGTCCCTTCAGTGGAAACTTGTCCAGCGGGAGCTGTGACCACAGAGGCTCACACA

TCGCCCAGGGCAGCCCCTGCACACGGGAGTCCCTGTGCTTTCTGAGACAAATTTTCAGAC

CCATTCAGCTCCTGCCCTCCTTCTAGGGCTCCTCTTCTGCTTTGGTCTCCTGTCCTCTCT

CCCTTCCCTGATTCCAGTAATCTTCGTGCTGACTCCAATCCCAACTCATGAATCTAAAGC

AGAGCCTAATTTAGATTTATATTTGTTTGTAAAATTGGGTCCATAGTCTAGAATTGTTCC

TTCCTGAAGAGAGAAACCTGATTGTGTGCTGCAGTGTGTGGGG

>HLA:HLA00475 C*16:01:01:01

TTATTTTGCTGGATGTAGTTTAATATTACCTGAGGTAAGGTAAGGCAAAGAGTGGGAGGC

AGGGAGTCCAGTTCAGGGACGGGGATTCCAGGAGAAGTGAAGGGGAAGGGGCTGGGCGCA

GCCTGGGGGTCTCTCCCTGGTTTCCACAGACAGATCCTTGGCCAGGACTCAGGCACACAG

TGTGACAAAGATGCTTGGTGTAGGAGAAGAGGGATCAGGACGAAGTCCCAGGTCCCGGGC

GGGGCTCTCAGGGTCTCAGGCTCCAAGGGCCGTGTCTGCACTGGGGAGGCGCCGCGTTGA

GGATTCTCCACTCCCCTGAGTTTCACTTCTTCTCCCAACCTGCGTCGGGTCCTTCTTCCT

GAATACTCATGACGCGTCCCCAATTCCCACTCCCATTGGGTGTCGGGTTCTAGAGAAGCC

AATCAGCGTCTCCGCAGTCCCGGTTCTAAAGTCCCCAGTCACCCACCCGGACTCGGATTC

TCCCCAGACGCCGAGATGCGGGTCATGGCGCCCCGAACCCTCATCCTGCTGCTCTCGGGA

GCCCTGGCCCTGACCGAGACCTGGGCCTGTGAGTGCGGGGTTGGGAGGGAAACGGCCTCT

GCGGAGAGGAGCGAGGGGCCCGCCCGGCGAGGGCGCAGGACCCGGGGAGCCGCGCAGGGA

GGAGGGTCGGGCGGGTCTCAGCCCCTCCTCGCCCCCAGGCTCCCACTCCATGAGGTATTT

CTACACCGCCGTGTCCCGGCCCGGCCGCGGAGAGCCCCGCTTCATCGCAGTGGGCTACGT

GGACGACACGCAGTTCGTGCGGTTCGACAGCGACGCCGCGAGTCCAAGAGGGGAGCCGCG

GGCGCCGTGGGTGGAGCAGGAGGGGCCGGAGTATTGGGACCGGGAGACACAGAAGTACAA

GCGCCAGGCACAGACTGACCGAGTGAGCCTGCGGAACCTGCGCGGCTACTACAACCAGAG

CGAGGCCGGTGAGTGACCCCGGCCCGGGGCGCAGGTCACGACCCCTCCCCATCCCCCACG

GACGGCCCGGGTCGCCCCGAGTCTCCCGGTCTGAGATCCACCCCGAGGCTGCGGAACCCG

CCCAGACCCTCGGCCGGAGAGAGCCCCAGTCACCTTTACCCGGTTTCATTTTCAGTTTAG

GCCAAAATCCCCGCGGGTTGGTCGGGGCTGGGGCGGGGCTCGCGGGACGGGGCTGACCAC

GGGGGCGGGGCCAGGGTCTCACACCCTCCAGTGGATGTATGGCTGCGACCTGGGGCCCGA

CGGGCGCCTCCTCCGCGGGTATGACCAGTCCGCCTACGACGGCAAGGATTACATCGCCCT

GAACGAGGACCTGCGCTCCTGGACCGCCGCGGACACGGCGGCTCAGATCACCCAGCGCAA

GTGGGAGGCGGCCCGTGCGGCGGAGCAGCAGAGAGCCTACCTGGAGGGCACGTGCGTGGA

GTGGCTCCGCAGATACCTGGAGAACGGGAAGGAGACGCTGCAGCGCGCGGGTACCAGGGG

CAGTGGGGAGCCTTCCCCATCTCCTGTAGATCTCCCGGGATGGCCTCCCACGAGGAGGGG

AGGAAAATGGGATCAGCGCTAGAATATCGCCCTCCCTTGAATGGAGAATGGGATGAGTTT

TCCTGAGTTTCCTCTGAGGGCCCCCTCTGCTCTCTAGGACAATTAAGGGATGAAGTCCTT

GAGGAAATGGAGGGGAAGACAGTCCCTGGAATACTGATCAGGGGTCCCCTTTGACCACTT

TGACCACTGCAGCAGCTGTGGTCAGGCTGCTGACCTTTCTCTCAGGCCTTGTTCTCTGCC

TCATGCTCAATGTGTTTGAAGGTTTGATTCCAGCTTTTCTGAGTTCTTCAGCCTCCACTC

AGGTCAGGACCAGAAGTCGCTGTTCCTCCCTCAGAGACTAGAACTTTCCAATGAATAGGA

GATTATCCCAGGTGCCTGTGTCCAGGCTGGCGTCTGGGTTCTGTGCCCCCTTCCCCACCC

CAGGTGTCCTGTCCATTCTCAGGATGGTCACATGGGCGCTGTTGGAGTGTCGCAAGAGAG

ATACAAAGTGTCTGAATTTTCTGACTCTTCCCGTCAGAACACCCAAAGACACACGTGACC

CACCATCTCGTCTCTGACCATGAGGCCACCCTGAGGTGCTGGGCCCTGGGCTTCTACCCT

GCGGAGATCACACTGACCTGGCAGCGGGATGGCGAGGACCAAACTCAGGACACCGAGCTT

GTGGAGACCAGGCCAGCAGGAGATGGAACCTTCCAGAAGTGGGCAGCTGTGGTGGTGCCT

TCTGGAGAAGAGCAGAGATACACGTGCCATGTGCAGCACGAGGGGCTGCCGGAGCCCCTC

ACCCTGAGATGGGGTAAGGAGGGGGATGAGGGGTCATGTGTCTTCTCAGGGAAAGCAGAA

GTCCTGGAGCCCTTCAGCCGGGTCAGGGCTGAGGCTTGGGGGTCAGGGCCCCTCACCTTC

CCCTCCTTTCCCAGAGCCATCTTCCCAGCCCACCATCCCCATCGTGGGCATCGTTGCTGG

CCTGGCTGTCCTGGCTGTCCTAGCTGTCCTAGGAGCTGTGGTGGCTGTTGTTATGTGTAG

GAGGAAGAGCTCAGGTAGGGAAGGGGTGAGGAGTGGGGTCTGGGTTTTCTTGTCCCACTG

GGAGTTTCAAGCCCCAGGTAGAAGTGTGCCCCACCTCGTTACTGGAAGCACCATCCACAC

ATGGGCCATCCCAGCCTGGGACCCTGTGTGCCAGCACTTACTCTGTTGTGAAGCACATGA

CAATGAAGGACAGATGTATCACCTTGATGATTATGGTGTTGGGGTCCTTGATTCCAGCAT

TCATGAGTCAGGGGAAGGTCCCTGCTAAGGACAGACCTTAGGAGGGCAGTTGCTCCAGAA

CCCACAGCTGCTTTCCCTGTGTTTCCTGATCCTGCCCTGGGTCTGCAGTCATAGTTCTGG

AAACTTCTCTTGGGTCCAAGACTAGGAGGTTCCCCTAAGATCGCATGGCCCTGCCTCCTC

CCTGTCCCCTCACAGGGCATTTTCTTCCCACAGGTGGAAAAGGAGGGAGCTGCTCTCAGG

CTGCGTGTAAGTGATGGCAGTGGGCGTGTGGAGGAGCTCACCCACCCCATAATTCCTCTT

GTCCCACATCTCCTGCGGGCTCTGACCAGGTCTTTTTTTTTGTTCTACCCCAGCCAGCAA

CAGTGCCCAGGGCTCTGATGAGTCTCTCATCGCTTGTAAAGGTGAGATTCTGGGGAGCTG

AAGTGGTCGGGGGTGGGGCAGAGGGAAAAGGCCTAGGTAATGGGGATCCTTTGATTGGGA

CGTTTCGAATGTGTGGTGAGCTGTTCAGAGTGTCATCACTTACCATGACTGACCTGAATT

TGTTCATGACTATTGTGTTCTGTAGCCTGAGACAGCTGCCTGTGTGGGACTGAGATGCAG

GATTTCTTCACACCTCTCCTTTGTGACTTCAAGAGCCTCTGGCATCTCTTTCTGCAAAGG

CATCTGAATGCGTCTGCGTTCCTGTTAGCATAATGTGAGGAGGTGGAGAGACAGCCCACC

CCCGTGTCCACCGTGACCCCTGTCCCCACACTGACCTGTGTTCCCTCCCCGATCATCTTT

CCTGTTCCAGAGAAGTGGGCTGGATGTCTCCATCTCTGTCTCAACTTTACGTGTACTGAG

CTGCAACTTCTTACTTCCCTACTGAAAATAAGAATCTGAATATAAATTTGTTTTCTCAAA

TATTTGCTATGAGAGGTTGATGGATTAATTAAATAAGTCAATCCCTGGAAGTTGAGAGAG

CAAATAAAGACCTGAGAACCTTCCAGAATCCGCATGTTCGCTGTGCTGAGTCTGTTGCAG

GTGGGGGTGGGGAAGGCTGTGAGGAGACGAGTGTGGACGGGGCCTGTGCCTAGTTGCTGT

TCAGTTCTTCATGGGCTTTATGTGGTCAGTCCTCAGCTGGGTCACCTTCACTGCTCCATT

GTCCTTGTCCCTTCAGTGGAAACTTGTCCAGCGGGAGCTGTGACCACAGAGGCTCACACA

TCGCCCAGGGCAGCCGCTGCACACTGGAGTCCCTGTGCTTTCTGAGACAAATTTTCAGAC

CCATTCAGCTCCTGCCCTCCTTCTAGGGCTCCTCTTCTGCTTTGGTCTCCTGCCCTCTCT

CCCTTCCCTGATTCCAGTAATCTTCGTGCTGACTCCAATCCCAACTCATGAATCTAAAGC

AGAGCCTAATTTAGATTTATATTTGTTTGTAGAATTGGGTCCATAGTCTAGAATTGTTCC

TTCCTGAAGAGAGAAACCTGATTGTGTGCTGCAGTGTGCGGGG

>HLA:HLA00485 DMA*01:01:01:01

GCTGGTCGCTTACAGACTGAGGCTGACAGCTTGAAACCAAAGAGGGAACTCCACCAGGAA

GCAACATTCCTCCACTGGGCTTCCCAGCCCAGTTACATGCCATACTCTGCCCTGGTCAAA

CAGCCAAGTCTTCAGGAGGTTACTGGCCCCAGGCGTCTCCCCAGTGACTGATGATGTTAA

ACCCTACGCTTCTCTGATTGGTTTAGACAAAATGACAAGGGCACCTATTGGAAATGATCT

GGCAAAACATGATCTAAGGCCACCCTCTCGGGGAGGGAGTTGGGGAAGCTGGGTTGGCTG

GGTTGGTAGCTCCTACCTACTGTGTGGCAAGAAGGTATGGGTCATGAACAGAACCAAGGA

GCTGCGCTGCTACAGATGTTACCACTTCTGTGGCTGCTACCCCACTCCTGGGCCGTCCCT

GAAGGTAAGATGGTACTCCTATTTACTTCCATCCTGAACCTAGGGAGCCCACTCAGCTTT

GTGAGGAAAAGCGCTGTGCTTTGTGAGTGGTGGGAAGTCTTATGAGGCAGTGGAAACAAC

AGGGGAGTGGGGAAAGACAGCTGCTATGTGTGGTTGGTAAACGATATCAGTGATGCTTTG

CATGTTCCATTTAAGACAATCTCTTGAAGCGGAGATTGTTCTCTCCATTTTACAAATGAA

GAAAATGAGACTCAGAGTTATTTGTGCAAGTTCACACCATTGGAAAGTGGTAGAGCTGGG

ATTTGAACAAAGTGAATGTTATTTTCACTCCTCCACTCAAGATTCTACTCTGCTTTCTAT

CATTAACTTATCTTGTGATTCTTGAAAAGTGTCTTAGTTTCCTTCTCTGTAGAGGATGGG

GCAGAATGGAAGAATAATCTGGAAGATCCCTCTTCCTCCAAATGTCTGTTTTTCTGGGTA

TTGGGTTAAAGGTTTCCTGCTGGCTGAGCGATTCCTGCCAAGAGCTTCAAAGAAGAGGGA

TTATTTTATGCAGTTATGGGGTCACTTCTCCCATTGCTGTCTTTGGGAGGCCTGGTAGGT

TAAGGAACAGAAGACATTTTGAGAGACGCTTGGTGCCAATGAAGTGGCTGAAGAAATGTC

TGGATACAGGTTTTCCAATTTACCATTTGTGTCTGGGGATAGTGGGAGGCAGTCCCATCT

ATGGCTTGGGGAGGCTGTAGCCATTCCTCCTCTTTCCCTTCTAAGAGTGAGAAACCCATC

CATGGCCTTCTCTAGTCAATACAGTGGCCTGGTATGAATTAGACAAAGGAATCCCCTTTG

GGAGGATACAGCCCTATGCCAGGATGTATGGCATGGTGAGCAGAATGCAGGCTTTCACTT

GCTTCTTTGGCTAGGTGTCTTTGTGCAGCCCCCAAACACACAATGAACAGTGACTCTGAA

TCTGTCACTTTATTTTATATATATACATTTTTAAATTTTTTATTTGATTTAATTTATTTT

TTTTGACACGGCATCTCGCTCTGTCACCCAGGCTAGAGTGCAGTGACGTGATCTCGGCTC

CCTGCAACCCCTGCCTCTGGGGTTCAAGAGACTCTCCTGCCTCAGCCTCCCAAGTAGCTG

GGATTACAGGCGCATGCCACCACACCCAGCTGATTTTTGTATTTTTAGTAGAGATGGGGT

TTCATTATGTTTGCCAGGCTGGTCTCAAACTCCTGACCTCAGGTGATCCACCCGCCTTGG

CCTCCCAAAGTGCTGGGATTACAAGCTTGAGCCACCGCGCCCGGCCTTTATATATATATT

TTTTGAGACGGGCTCATTCTGTCACCTGGGCTGGAGTCCAGTGGCACAATCATGGCTCAC

TGCAGCCCCAACTTCCTAGGCACAAGCAATCCTCCTGCCTCAGCCTCCAGAGTAGCTGAG

ACCACAGGTGTTTGCCACCACACCTGGCTAAGTTTTTGTATTTTTTGTAGAGACGGTCTC

GCTGTGTTGCCCAGGTTGGTCTTGAACTCCTGGGCTCAAGCAATCTGCCTGCCTCAGCCT

CCCAAAGTGCTGGGATTACAGGCATGAGCCACCATGCCTGGCCTGAATCTGTCACTTTAG

AAAGTAGAACTCTTTACTTTTGCCAACTGGCCTTTTTATAGGCAGGGGAAGCTTGGAGAG

ATGCCATAATTTGTTCTTCACCCCCTTTGGGAGGGGGAGTCTAAATAACAGACACAGGAA

AGGGTCTCACCTCAGCCCACGCTCCTTAAGCAGGCCTTGCTTGTTTTTGTTGTTTTGTTT

TGAGACAGAGTCTCACTCTGTCACCCAGGCTGGAGTGCAGTGGCTTAATCTTAGCTCCCG

GCAACCTCTGCCTCCCTGGTTCAAGCAATTCTCCTCCCTCAGCCTCCCGAGTAGCTACAG

GCGCATGCCAACATGCCCAGCTACTTTTTTGTATTTTTAGTAGAGATGGAGTTTCACCAT

GCTGGCCAGGCTGGTCTCAAACTCCTGACCTCATGATCCACCCACCTTGGCCTCCCAAAG

TGCTGGGATTACAGGCATGAGCCACTGCGCCTAGCCCCACCTTGCTGTTTTTTAAGTTGG

AGGAGAAGGCTCCCACCTCCCTGAACCTCACCCTGTGTCCTTTGCAGCTCCTACTCCAAT

GTGGCCAGATGACCTGCAAAACCACACATTCCTGCACACAGTGTACTGCCAGGATGGGAG

TCCCAGTGTGGGACTCTCTGAGGCCTACGACGAGGACCAGCTTTTCTTCTTCGACTTTTC

CCAGAACACTCGGGTGCCTCGCCTGCCCGAATTTGCTGACTGGGCTCAGGAACAGGGAGA

TGCTCCTGCCATTTTATTTGACAAAGAGTTCTGCGAGTGGATGATCCAGCAAATAGGGCC

AAAACTTGATGGGAAAATCCCGGTGTCCAGAGGTCAGGAGTTTTCTGGGGAGTGAAGGGA

GGAGGGCTGCATTAACCTCATTGATCTGTACACTGAATAATTCCCCTTGATACCAGCTCC

CCATCTCAAATACTTTCTGGTTCTCTTCATCACCTTAATTTTTCCACCAGCCTTGGTCTG

CACCCTGTGTTCTTTTGGTGGGCCGAAGTACCTAGCATGTAGTAGGCATTCAGAACTATG

TATTGAATGTGATGAATTCAACAGGTACCAACTGATACCTACTGAATATTAACACTTGTG

CTACTATGCCCAGCAAGATGGATGGGAAGAGTGGAAATATCTGATGACGTGACTATGTCT

TAGTGAGAAGACAGTGCATGGTTAGACATCAATGTGAGCTCTAGACAGGAAGTGCTGAAG

GAAGTCATGGTGGAGGGGTCCAGAGTAGCCTGGATCTGGCTCTGCTTCTACGTCTAGCTG

CAGTCCTTGCCTGGAAGAAGACCTCCCTTCAGAGCCCAGCTCCTCGCTCATCTCTGTCTC

CCAAAGCCTGACCCACTGTGTTCTTCTCTGCCCTCCCCTCCATATGCCATGGCCCCTCCA

AACACAGAGATACCATCTAAACTAGTCTCTTTTTCCCCCTACACTTCAATCCCCCCACCA

GGGTTTCCTATCGCTGAAGTGTTCACGCTGAAGCCCCTGGAGTTTGGCAAGCCCAACACT

TTGGTCTGTTTTGTCAGTAATCTCTTCCCACCCATGCTGACAGTGAACTGGCAGCATCAT

TCCGTCCCTGTGGAAGGATTTGGGCCTACTTTTGTCTCAGCTGTCGATGGACTCAGCTTC

CAGGCCTTTTCTTACTTAAACTTCACACCAGAACCTTCTGACATTTTCTCCTGCATTGTG

ACTCACGAAATTGACCGCTACACAGCAATTGCCTATTGGGGTGAGGCTTTCTCCCTGGAA

TTCTGGTCCTTTTGGGGGCAAAAAGGGATAGATCCATGGGAGGAGGCTTCTTTCTCCACT

GGTACCTTGTTTAGTCCATTCCTACCCTAAGCCCATCCCAGTCTCCCATGTCATCCCAGA

CACCCACGTCATTTCCCTGGGTGGGAGGCTCCCTAACTAGGTCCCCAGGCTGAGCCACTC

ATTTCCTCCAGTACCCCGGAACGCACTGCCCTCAGATCTGCTGGAGAATGTGCTGTGTGG

CGTGGCCTTTGGCCTGGGTGTGCTGGGCATCATCGTGGGCATTGTTCTCATCATCTACTT

CCGGAAGCCTTGCTCAGGTGGTATGTCATCTGGAGGGGGCGGGTGAGCCTGTGGGAGCCA

GATACAGTGGTGCATGCGTGCATGTGTCAGGATTATTTTGTGGCATGGGGGGACATATAG

CGATCCTCAGGCCCTTGGGTGTGGGGGCCTGTATCCAGCACCATGGGGGCACATCTTCCC

AGTTGGGGACCCAGTTACACACACACAGTTATGGGTCACAAGAATTGCTTTGAGTGAAAA

AAGGAATCATGGGGTGCTGTAGGAAGGGTGCTTGGAGATGATTTGGGAACAAGGAGAGAT

CAACTTCTGCAGGGTGTGGTGCAGGCAGGGCGAGCAAGGCCTTTGTGGGGGAGGAGGAGG

AGGAGAAGGAGGAGGAGGATGGATCCCCTGATGCCTTTCCTCCATCCCTGTCTCTCCCCC

AGACTGATTCTTCCAGACCAGAGTTTGATGCCAGCAGCTTCGGCCATCCAAACAGAGGAT

GCTCAGATTTCTCACATCCTGCCCAGGATCTCCTCTTAGGGTAGAAGAAGTCTCTGGGAC

ATCCCTGGGGTGTGTGTGTAGATTTCCCACCTGGGGACTCTGCTGTCCCTGGGCTTGCAT

CCCAGGGATCCCAGAGTGGCCTGCCTATCACAACCACATCCCTTCCCCCCACAAGGCAAT

AAATCTCATTTCTTTATATCAGTGTGGCTTCTTTCTTAACTCATGGTATTTGTTTCTGGA

TATCTCAACTTGAGTGGGTTGTCGTTTCAAATTCAGCATGCCTTAACCTGAACACAGCTT

GACCTCGTTAGGGAGGGAAATAGGGAAAACCCCTAATTTGCCAGCTGAGCTCTTATTCCC

TGGTCTTGGCGGTACATGATGTTTTTCCATCTATCGGTTTGTGCAAAATATGTGAGAAAC

GAAGGCAGAGTTATTTTCTAATAATCTGCTT

>HLA:HLA06456 DMA*01:01:01:02

GCTGGTCGCTTACAGACTGAGGCTGACAGCTTGAAACCAAAGAGGGAACTCCACCAGGAA

GCAACATTCCTCCACTGGGCTTCCCAGCCCAGTTACATGCCATACTCTGCCCTGGTCAAA

CAGCCAAGTCTTCAGGAGGTTACTGGCCCCAGGCGTCTCCCCAGTGACTGATGATGTTAA

ACCCTACGCTTCTCTGATTGGTTTAGACAAAATGACAAGGGCACCTATTGGAAATGATCT

GGCAAAACATGATCTAAGGCCACCCTCTCGGGGAGGGAGTTGGGGAAGCTGGGTTGGCTG

GGTTGGTAGCTCCTACCTACTGTGTGGCAAGAAGGTATGGGTCATGAACAGAACCAAGGA

GCTGCGCTGCTACAGATGTTACCACTTCTGTGGCTGCTACCCCACTCCTGGGCCGTCCCT

GAAGGTAAGATGGTACTCCTATTTACTTCCATCCTGAACCTAGGGAGCCCACTCAGCTTT

GTGAGGAAAAGCGCTGTGCTTTGTGAGTGGTGGGAAGTCTTATGAGGCAGTGGAAACAAC

AGGGGAGTGGGGAAAGACAGCTGCTATGTGTGGTTGGTAAACGATATCAGTGATGCTTTG

CATGTTCCATTTAAGACAATCTCTTGAAGCGGAGATTGTTCTCTCCATTTTACAAATGAA

GAAAATGAGACTCAGAGTTATTTGTGCAAGTTCACACCATTGGAAAGTGGTAGAGCTGGG

ATTTGAACAAAGTGAATGTTATTTTCACTCCTCCACTCAAGATTCTACTCTGCTTTCTAT

CATTAACTTATCTTGTGATTCTTGAAAAGTGTCTTAGTTTCCTTCTCTGTAGAGGATGGG

GCAGAATGGAAGAATAATCTGGAAGATCCCTCTTCCTCCAAATGTCTGTTTTTCTGGGTA

TTGGGTTAAAGGTTTCCTGCTGGCTGAGCGATTCCTGCCAAGAGCTTCAAAGAAGAGGGA

TTATTTTATGCAGTTATGGGGTCACTTCTCCCATTGCTGTCTTTGGGAGGCCTGGTAGGT

TAAGGAACAGAAGACATTTTGAGAGACGCTTGGTGCCAATGAAGTGGCTGAAGAAATGTC

TGGATACAGGTTTTCCAATTTACCATTTGTGTCTGGGGATAGTGGGAGGCAGTCCCATCT

ATGGCTTGGGGAGGCTGTAGCCATTCCTCCTCTTTCCCTTCTAAGAGTGAGAAACCCATC

CATGGCCTTCTCTAGTCAATACAGTGGCCTGGTATGAATTAGACAAAGGAATCCCCTTTG

GGAGGATACAGCCCTATGCCAGGATGTATGGCATGGTGAGCAGAATGCAGGCTTTCACTT

GCTTCTTTGGCTAGGTGTCTTTGTGCAGCCCCCAAACACACAATGAACAGTGACTCTGAA

TCTGTCACTTTATTTTATATATATACATTTTTAAATTTTTTATTTGATTTAATTTATTTT

TTTTGACACGGCATCTCGCTCTGTCACCCAGGCTAGAGTGCAGTGACGTGATCTCGGCTC

CCTGCAACCCCTGCCTCTGGGGTTCAAGAGACTCTCCTGCCTCAGCCTCCCAAGTAGCTG

GGATTACAGGCGCATGCCACCACACCCAGCTGATTTTTGTATTTTTAGTAGAGATGGGGT

TTCATCATGTTTGCCAGGCTGGTCTCAAACTCCTGACCTCAGGTGATCCACCCGCCTTGG

CCTCCCAAAGTGCTGGGATTACAAGCTTGAGCCACCGCGCCCGGCCTTTATATATATATT

TTTTGAGACGGGCTCATTCTGTCACCTGGGCTGGAGTCCAGTGGCACAATCATGGCTCAC

TGCAGCCCCAACTTCCTAGGCACAAGCAATCCTCCTGCCTCAGCCTCCAGAGTAGCTGAG

ACCACAGGTGTTTGCCACCACACCTGGCTAAGTTTTTGTATTTTTTGTAGAGACAGGGTC

TCGCTGTGTTGCCCAGGTTGGTCTTGAACTCCTGGGCTCAAGCAATCTGCCTGCCTCAGC

CTCCCAAAGTGCTGGGATTACAGGCATGAGCCACCATGCCTGGCCTGAATCTGTCACTTT

AGAAAGTAGAACTCTTTACTTTTGCCAACTGGCCTTTTTATAGGCAGGGGAAGCTTGGAG

AGATGCCATAATTTGTTCTTCACCCCCTTTGGGAGGGGGAGTCTAAATAACAGACACAGG

AAAGGGTCTCACCTCAGCCCACGCTCCTTAAGCAGGCCTTGCTTGTTTTTGTTGTTTTGT

TTTGAGACAGAGTCTCACTCTGTCACCCAGGCTGGAGTGCAGTGGCTTAATCTTAGCTCC

CGGCAACCTCTGCCTCCCTGGTTCAAGCAATTCTCCTCCCTCAGCCTCCCGAGTAGCTAC

AGGCGCATGCCAACATGCCCAGCTACTTTTTTGTATTTTTAGTAGAGATGGAGTTTCACC

ATGCTGGCCAGGCTGGTCTCAAACTCCTGACCTCATGATCCACCCACCTTGGCCTCCCAA

AGTGCTGGGATTACAGGCATGAGCCACTGCGCCTAGCCCCACCTTGCTGTTTTTTAAGTT

GGAGGAGAAGGCTCCCACCTCCCTGAACCTCACCCTGTGTCCTTTGCAGCTCCTACTCCA

ATGTGGCCAGATGACCTGCAAAACCACACATTCCTGCACACAGTGTACTGCCAGGATGGG

AGTCCCAGTGTGGGACTCTCTGAGGCCTACGACGAGGACCAGCTTTTCTTCTTCGACTTT

TCCCAGAACACTCGGGTGCCTCGCCTGCCCGAATTTGCTGACTGGGCTCAGGAACAGGGA

GATGCTCCTGCCATTTTATTTGACAAAGAGTTCTGCGAGTGGATGATCCAGCAAATAGGG

CCAAAACTTGATGGGAAAATCCCGGTGTCCAGAGGTCAGGAGTTTTCTGGGGAGTGAAGG

GAGGAGGGCTGCATTAACCTCATTGATCTGTACACTGAATAATTCCCCTTGATACCAGCT

CCCCATCTCAAATACTTTCTGGTTCTCTTCATCACCTTAATTTTTCCACCAGCCTTGGTC

TGCACCCTGTGTTCTTTTGGTGGGCCGAAGTACCTAGCATGTAGTAGGCATTCAGAACTA

TGTATTGAATGTGATGAATTCAACAGGTACCAACTGATACCTACTGAATATTAACACTTG

TGCTACTATGCCCAGCAAGATGGATGGGAAGAGTGGAAATATCTGATGACGTGACTATGT

CTTAGTGAGAAGACAGTGCATGGTTAGACATCAATGTGAGCTCTAGACAGGAAGTGCTGA

AGGAAGTCATGGTGGAGGGGTCCAGAGTAGCCTGGATCTGGCTCTGCTTCTACGTCTAGC

TGCAGTCCTTGCCTGGAAGAAGACCTCCCTTCAGAGCCCAGCTCCTCGCTCATCTCTGTC

TCCCAAAGCCTGACCCACTGTGTTCTTCTCTGCCCTCCCCTCCATATGCCATGGCCCCTC

CAAACACAGAGATACCATCTAAACTAGTCTCTTTTTCCCCCTACACTTCAATCCCCCCAC

CAGGGTTTCCTATCGCTGAAGTGTTCACGCTGAAGCCCCTGGAGTTTGGCAAGCCCAACA

CTTTGGTCTGTTTTGTCAGTAATCTCTTCCCACCCATGCTGACAGTGAACTGGCAGCATC

ATTCCGTCCCTGTGGAAGGATTTGGGCCTACTTTTGTCTCAGCTGTCGATGGACTCAGCT

TCCAGGCCTTTTCTTACTTAAACTTCACACCAGAACCTTCTGACATTTTCTCCTGCATTG

TGACTCACGAAATTGACCGCTACACAGCAATTGCCTATTGGGGTGAGGCTTTCTCCCTGG

AATTCTGGTCCTTTTGGGGGCAAAAAGGGATAGATCCATGGGAGGAGGCTTCTTTCTCCA

CTGGTACCTTGTTTAGTCCATTCCTACCCTAAGCCCATCCCAGTCTCCCATGTCATCCCA

GACACCCACGTCATTTCCCTGGGTGGGAGGCTCCCTAACTAGGTCCCCAGGCTGAGCCAC

TCATTTCCTCCAGTACCCCGGAACGCACTGCCCTCAGATCTGCTGGAGAATGTGCTGTGT

GGCGTGGCCTTTGGCCTGGGTGTGCTGGGCATCATCGTGGGCATTGTTCTCATCATCTAC

TTCCGGAAGCCTTGCTCAGGTGGTATGTCATCTGGAGGGGGCGGGTGAGCCTGTGGGAGC

CAGATACAGTGGTGCATGCGTGCATGTGTCAGGATTATTTTGTGGCATGGGGGGACATAT

AGCGATCCTCAGGCCCTTGGGTGTGGGGGCCTGTATCCAGCACCATGGGGGCACATCTTC

CCAGTTGGGGACCCAGTTACACACACACAGTTATGGGTCACAAGAATTGCTTTGAGTGAA

AAAAGGAATCATGGGGTGCTGTAGGAAGGGTGCTTGGAGATGATTTGGGAACAAGGAGAG

ATCAACTTCTGCAGGGTGTGGTGCAGGCAGGGCGAGCAAGGCCTTTGTGGGGGAGGAGGA

GGAGGAGAAGGAGGAGGAGGATGGATCCCCTGATGCCTTTCCTCCATCCCTGTCTCTCCC

CCAGACTGATTCTTCCAGACCAGAGTTTGATGCCAGCAGCTTCGGCCATCCAAACAGAGG

ATGCTCAGATTTCTCACATCCTGCCCAGGATCTCCTCTTAGGGTAGAAGAAGTCTCTGGG

ACATCCCTGGGGTGTGTGTGTAGATTTCCCACCTGGGGACTCTGCTGTCCCTGGGCTTGC

ATCCCAGGGATCCCAGAGTGGCCTGCCTATCACAACCACATCCCTTCCCCCCACAAGGCA

ATAAATCTCATTTCTTTATATCAGTGTGGCTTCTTTCTTAACTCATGGTATTTGTTTCTG

GATATCTCAACTTGAGTGGGTTGTCGTTTCAAATTCAGCATGCCTTAACCTGAACACAGC

TTGACCTCGTTAGGGAGGGAAATAGGGAAAACCCCTAATTTGCCAGCTGAGCTCTTATTC

CCTGGTCTTGGCGGTACATGATGTTTTTCCATCTATCGGTTTGTGCAAAATATGTGAGAA

ACGAAGGCAGAGTTATTTTCTAATAATCTGCTT

>HLA:HLA06464 DMA*01:01:01:04

GCTGGTCGCTTACAGACTGAGGCTGACAGCTTGAAACCAAAGAGGGAACTCCACCAGGAA

GCAACATTCCTCCACTGGGCTTCCCAGCCCAGTTACATGCCATACTCTGCCCTGGTCAAA

CAGCCAAGTCTTCAGGAGGTTACTGGCCCCAGGCGTCTCCCCAGTGACTGATGATGTTAA

ACCCTACGCTTCTCTGATTGGTTTAGACAAAATGACAAGGGCACCTATTGGAAATGATCT

GGCAAAACATGATCTAAGGCCACCCTCTCGGGGAGGGAGTTGGGGAAGCTGGGTTGGCTG

GGTTGGTAGCTCCTACCTACTGTGTGGCAAGAAGGTATGGGTCATGAACAGAACCAAGGA

GCTGCGCTGCTACAGATGTTACCACTTCTGTGGCTGCTACCCCACTCCTGGGCCGTCCCT

GAAGGTAAGATGGTACTCCTATTTACTTCCATCCTGAACCTAGGGAGCCCACTCAGCTTT

GTGAGGAAAAGCGCTGTGCTTTGTGAGTGGTGGGAAGTCTTATGAGGCAGTGGAAACAAC

AGGGGAGTGGGGAAAGACAGCTGCTATGTGTGGTTGGTAAACGATATCAGTGATGCTTTG

CATGTTCCATTTAAGACAATCTCTTGAAGCGGAGATTGTTCTCTCCATTTTACAAATGAA

GAAAATGAGACTCAGAGTTATTTGTGCAAGTTCACACCATTGGAAAGTGGTAGAGCTGGG

ATTTGAACAAAGTGAATGTTATTTTCACTCCTCCACTCAAGATTCTACTCTGCTTTCTAT

CATTAACTTATCTTGTGATTCTTGAAAAGTGTCTTAGTTTCCTTCTCTGTAGAGGATGGG

GCAGAATGGAAGAATAATCTGGAAGATCCCTCTTCCTCCAAATGTCTGTTTTTCTGGGTA

TTGGGTTAAAGGTTTCCTGCTGGCTGAGCGATTCCTGCCAAGAGCTTCAAAGAAGAGGGA

TTATTTTATGCAGTTATGGGGTCACTTCTCCCATTGCTGTCTTTGGGAGGCCTGGTAGGT

TAAGGAACAGAAGACATTTTGAGAGACGCTTGGTGCCAATGAAGTGGCTGAAGAAATGTC

TGGATACAGGTTTTCCAATTTACCATTTGTGTCTGGGGATAGTGGGAGGCAGTCCCATCT

ATGGCTTGGGGAGGCTGTAGCCATTCCTCCTCTTTCCCTTCTAAGAGTGAGAAACCCATC

CATGGCCTTCTCTAGTCAATACAGTGGCCTGGTATGAATTAGACAAAGGAATCCCCTTTG

GGAGGATACAGCCCTATGCCAGGATGTATGGCATGGTGAGCAGAATGCAGGCTTTCACTT

GCTTCTTTGGCTAGGTGTCTTTGTGCAGCCCCCAAACACACAATGAACAGTGACTCTGAA

TCTGTCACTTTATTTTATATATATACATTTTTAAATTTTTTATTTGATTTAATTTATTTT

TTTTGACACGGCATCTCGCTCTGTCACCCAGGCTAGAGTGCAGTGACGTGATCTCGGCTC

CCTGCAACCCCTGCCTCTGGGGTTCAAGAGACTCTCCTGCCTCAGCCTCCCAAGTAGCTG

GGATTACAGGCGCATGCCACCACACCCAGCTGATTTTTGTATTTTTAGTAGAGATGGGGT

TTCATCATGTTTGCCAGGCTGGTCTCAAACTCCTGACCTCAGGTGATCCACCCGCCTTGG

CCTCCCAAAGTGCTGGGATTACAAGCTTGAGCCACCGCGCCCGGCCTTTATATATATATT

TTTTGAGACGGGCTCATTCTGTCACCTGGGCTGGAGTCCAGTGGCACAATCATGGCTCAC

TGCAGCCCCAACTTCCTAGGCACAAGCAATCCTCCTGCCTCAGCCTCCAGAGTAGCTGAG

ACCACAGGTGTTTGCCACCACACCTGGCTAAGTTTTTGTATTTTTTGTAGAGACAGGGTC

TCGCTGTGTTGCCCAGGTTGGTCTTGAACTCCTGGGCTCAAGCAATCTGCCTGCCTCAGC

CTCCCAAAGTGCTGGGATTACAGGCATGAGCCACCATGCCTGGCCTGAATCTGTCACTTT

AGAAAGTAGAACTCTTTACTTTTGCCAACTGGCCTTTTTATAGGCAGGGGAAGCTTGGAG

AGATGCCATAATTTGTTCTTCACCCCCTTTGGGAGGGGGAGTCTAAATAACAGACACAGG

AAAGGGTCTCACCTCAGCCCACGCTCCTTAAGCAGGCCTTGCTTGTTTTTGTTGTTTTGT

TTTGAGACAGAGTCTCACTCTGTCACCCAGGCTGGAGTGCAGTGGCTTAATCTTAGCTCC

CGGCAACCTCTGCCTCCCTGGTTCAAGCAATTCTCCTCCCTCAGCCTCCCGAGTAGCTAC

AGGCGCATGCCAACATGCCCAGCTACTTTTTTGTATTTTTAGTAGAGATGGAGTTTCACC

ATGCTGGCCAGGCTGGTCTCAAACTCCTGACCTCATGATCCACCCACCTTGGCCTCCCAA

AGTGCTGGGATTACAGGCATGAGCCACTGCGCCTAGCCCCACCTTGCTGTTTTTTAAGTT

GGAGGAGAAGGCTCCCACCTCCCTGAACCTCACCCTGTGTCCTTTGCAGCTCCTACTCCA

ATGTGGCCAGATGACCTGCAAAACCACACATTCCTGCACACAGTGTACTGCCAGGATGGG

AGTCCCAGTGTGGGACTCTCTGAGGCCTACGACGAGGACCAGCTTTTCTTCTTCGACTTT

TCCCAGAACACTCGGGTGCCTCGCCTGCCCGAATTTGCTGACTGGGCTCAGGAACAGGGA

GATGCTCCTGCCATTTTATTTGACAAAGAGTTCTGCGAGTGGATGATCCAGCAAATAGGG

CCAAAACTTGATGGGAAAATCCCGGTGTCCAGAGGTCAGGAGTTTTCTGGGGAGTGAAGG

GAGGAGGGCTGCATTAACCTCATTGATCTGTACACTGAATAATTCCCCTTGATACCAGCT

CCCCATCTCAAATACTTTCTGGTTCTCTTCATCACCTTAATTTTTCCACCAGCCTTGGTC

TGCACCCTGTGTTCTTTTGGTGGGCCGAAGTACCTAGCATGTAGTAGGCATTCAGAACTA

TGTATTGAATGTGATGAATTCAACAGGTACCAACTGATACCTACTGAATATTAACACTTG

TGCTACTATGCCCAGCAAGATGGATGGGAAGAGTGGAAATATCTGATGACGTGACTATGT

CTTAGTGAGAAGACAGTGCATGGTTAGACATCAATGTGAGCTCTAGACAGGAAGTGCTGA

AGGAAGTCATGGTGGAGGGGTCCAGAGTAGCCTGGATCTGGCTCTGCTTCTACGTCTAGC

TGCAGTCCTTGCCTGGAAGAAGACCTCCCTTCAGAGCCCAGCTCCTCGCTCATCTCTGTC

TCCCAAAGCCTGACCCACTGTGTTCTTCTCTGCCCTCCCCTCCATATGCCATGGCCCCTC

CAAACACAGAGATACCATCTAAACTAGTCTCTTTTTCCCCCTACACTTCAATCCCCCCAC

CAGGGTTTCCTATCGCTGAAGTGTTCACGCTGAAGCCCCTGGAGTTTGGCAAGCCCAACA

CTTTGGTCTGTTTTGTCAGTAATCTCTTCCCACCCATGCTGACAGTGAACTGGCAGCATC

ATTCCGTCCCTGTGGAAGGATTTGGGCCTACTTTTGTCTCAGCTGTCGATGGACTCAGCT

TCCAGGCCTTTTCTTACTTAAACTTCACACCAGAACCTTCTGACATTTTCTCCTGCATTG

TGACTCACGAAATTGACCGCTACACAGCAATTGCCTATTGGGGTGAGGCTTTCTCCCTGG

AATTCTGGTCCTTTTGGGGGCAAAAAGGGATAGATCCATGGGAGGAGGCTTCTTTCTCCA

CTGGTACCTTGTTTAGTCCATTCCTACCCTAAGCCCATCCCAGTCTCCCATGTCATCCCA

GACACCCACGTCATTTCCCTGGGTGGGAGGCTCCCTAACTAGGTCCCCAGGCTGAGCCAC

TCATTTCCTCCAGTACCCCGGAACGCACTGCCCTCAGATCTGCTGGAGAATGTGCTGTGT

GGCGTGGCCTTTGGCCTGGGTGTGCTGGGCATCATCGTGGGCATTGTTCTCATCATCTAC

TTCCGGAAGCCTTGCTCAGGTGGTATGTCATCTGGAGGGGGCGGGTGAGCCTGTGGGAGC

CAGATACAGTGGTGCATGCGTGCATGTGTCAGGATTATTTTGTGGCATGGGGGGACATAT

AGCGATCCTCAGGCCCTTGGGTGTGGGGGCCTGTATCCAGCACCATGGGGGCACATCTTC

CCAGTTGGGGACCCAGTTACACACACACAGTTATGGGTCACAAGAATTGCTTTGAGTGAA

AAAAGGAATCATGGGGTGCTGTAGGAAGGGTGCTTGGAGATGATTTGGGAACAAGGAGAG

ATCAACTTCTGCAGGGTGTGGTGCAGGCAGGGCGAGCAAGGCCTTTGTGGGGGAGGAGGA

GGAGGAGAAGAAGGAGGAGGATGGATCCCCTGATGCCTTTCCTCCATCCCTGTCTCTCCC

CCAGACTGATTCTTCCAGACCAGAGTTTGATGCCAGCAGCTTCGGCCATCCAAACAGAGG

ATGCTCAGATTTCTCACATCCTGCCCAGGATCTCCTCTTAGGGTAGAAGAAGTCTCTGGG

ACATCCCTGGGGTGTGTGTGTAGATTTCCCACCTGGGGACTCTGCTGTCCCTGGGCTTGC

ATCCCAGGGATCCCAGAGTGGCCTGCCTATCACAACCACATCCCTTCCCCCCACAAGGCA

ATAAATCTCATTTCTTTATATCAGTGTGGCTTCTTTCTTAACTCATGGTATTTGTTTCTG

GATATCTCAACTTGAGTGGGTTGTCGTTTCAAATTCAGCATGCCTTAACCTGAACACAGC

TTGACCTCGTTAGGGAGGGAAATAGGGAAAACCCCTAATTTGCCAGCTGAGCTCTTATTC

CCTGGTCTTGGCGGTACATGATGTTTTTCCATCTATCGGTTTGTGCAAAATATGTGAGAA

ACGAAGGCAGAGTTATTTTCTAATAATCTGCTT

>HLA:HLA00486 DMA*01:02:01:01

GCTGGTCGCTTACAGACTGAGGCTGACAGCTTGAAACCAAAGAGGGAACTCCACCAGGAA

GCAACATTCCTCCACTGGGCTTCCCAGCCCAGTTACATGCCATACTCTGCCCTGGTCAAA

CAGCCAAGTCTTCAGGAGGTTACTGGCCCCAGGCGTCTCCCCAGTGACTGATGATGTTAA

ACCCTACGCTTCTCTGATTGGTTTAGACAAAATGACAAGGGCACCTATTGGAAATGATCT

GGCAAAACATGATCTAAGGCCACCCTCTCGGGGAGGGAGTTGGGGAAGCTGGGTTGGCTG

GGTTGGTAGCTCCTACCTACTGTGTGGCAAGAAGGTATGGGTCATGAACAGAACCAAGGA

GCTGCGCTGCTACAGATGTTACCACTTCTGTGGCTGCTACCCCACTCCTGGGCCGTCCCT

GAAGGTAAGATGGTACTCCTATTTACTTCCATCCTGAACCTAGGGAGCCCACTCAGCTTT

GTGAGGAAAAGCGCTGTGCTTTGTGAGTGGTGGGAAGTCTTATGAGGCAGTGGAAACAAC

AGGGGAGTGGGGAAAGACAGCTGCTATGTGTGGTTGGTAAACGATATCAGTGATGCTTTG

CATGTTCCATTTAAGACAATCTCTTGAAGCGGAGATTGTTCTCTCCATTTTACAAATGAA

GAAAATGAGACTCAGAGTTATTTGTGCAAGTTCACACCATTGGAAAGTGGTAGAGCTGGG

ATTTGAACAAAGTGAATGTTATTTTCACTCCTCCACTCAAGATTCTACTCTGCTTTCTAT

CATTAACTTATCTTGTGATTCTTGAAAAGTGTCTTAGTTTCCTTCTCTGTAGAGGATGGG

GCAGAATGGAAGAATAATCTGGAAGATCCCTCTTCCTCCAAATGTCTGTTTTTCTGGGTA

TTGGGTTAAAGGTTTCCTGCTGGCTGAGCGATTCCTGCCAAGAGCTTCAAAGAAGAGGGA

TTATTTTATGCAGTTATGGGGTCACTTCTCCCATTGCTGTCTTTGGGAGGCCTGGTAGGT

TAAGGAACAGAAGACATTTTGAGAGACGCTTGGTGCCAATGAAGTGGCTGAAGAAATGTC

TGGATACAGGTTTTCCAATTTACCATTTGTGTCTGGGGATAGTGGGAGGCAGTCCCATCT

ATGGCTTGGGGAGGCTGTAGCCATTCCTCCTCTTTCCCTTCTAAGAGTGAGAAACCCATC

CATGGCCTTCTCTAGTCAATACAGTGGCCTGGTATGAATTAGACAAAGGAATCCCCTTTG

GGAGGATACAGCCCTATGCCAGGATGTATGGCATGGTGAGCAGAATGCAGGCTTTCACTT

GCTTCTTTGGCTAGGTGTCTTTGTGCAGCCCCCAAACACACAATGAACAGTGACTCTGAA

TCTGTCACTTTATTTTATATATATACATTTTTAAATTTTTTATTTGATTTAATTTATTTT

TTTTGACACGGCATCTCGCTCTGTCACCCAGGCTAGAGTGCAGTGACGTGATCTCGGCTC

CCTGCAACCCCTGCCTCTGGGGTTCAAGAGACTCTCCTGCCTCAGCCTCCCAAGTAGCTG

GGATTACAGGCGCATGCCACCACACCCAGCTGATTTTTGTATTTTTAGTAGAGATGGGGT

TTCATCATGTTTGCCAGGCTGGTCTCAAACTCCTGACCTCAGGTGATCCACCCGCCTTGG

CCTCCCAAAGTGCTGGGATTACAAGCTTGAGCCACCGCGCCCGGCCTTTATATATATATT

TTTTGAGACGGGCTCATTCTGTCACCTGGGCTGGAGTCCAGTGGCACAATCATGGCTCAC

TGCAGCCCCAACTTCCTAGGCACAAGCAATCCTCCTGCCTCAGCCTCCAGAGTAGCTGAG

ACCACAGGTGTTTGCCACCACACCTGGCTAAGTTTTTGTATTTTTTGTAGAGACAGGGTC

TCGCTGTGTTGCCCAGGTTGGTCTTGAACTCCTGGGCTCAAGCAATCTGCCTGCCTCAGC

CTCCCAAAGTGCTGGGATTACAGGCATGAGCCACCATGCCTGGCCTGAATCTGTCACTTT

AGAAAGTAGAACTCTTTACTTTTGCCAACTGGCCTTTTTATAGGCAGGGGAAGCTTGGAG

AGATGCCATAATTTGTTCTTCACCCCCTTTGGGAGGGGGAGTCTAAATAACAGACACAGG

AAAGGGTCTCACCTCAGCCCACGCTCCTTAAGCAGGCCTTGCTTGTTTTTGTTGTTTTGT

TTTGAGACAGAGTCTCACTCTGTCACCCAGGCTGGAGTGCAGTGGCTTAATCTTAGCTCC

CGGCAACCTCTGCCTCCCTGGTTCAAGCAATTCTCCTCCCTCAGCCTCCCGAGTAGCTAC

AGGCGCATGCCAACATGCCCAGCTACTTTTTTGTATTTTTAGTAGAGATGGAGTTTCACC

ATGCTGGCCAGGCTGGTCTCAAACTCCTGACCTCATGATCCACCCACCTTGGCCTCCCAA

AGTGCTGGGATTACAGGCATGAGCCACTGCGCCTAGCCCCACCTTGCTGTTTTTTAAGTT

GGAGGAGAAGGCTCCCACCTCCCTGAACCTCACCCTGTGTCCTTTGCAGCTCCTACTCCA

ATGTGGCCAGATGACCTGCAAAACCACACATTCCTGCACACAGTGTACTGCCAGGATGGG

AGTCCCAGTGTGGGACTCTCTGAGGCCTACGACGAGGACCAGCTTTTCTTCTTCGACTTT

TCCCAGAACACTCGGGTGCCTCGCCTGCCCGAATTTGCTGACTGGGCTCAGGAACAGGGA

GATGCTCCTGCCATTTTATTTGACAAAGAGTTCTGCGAGTGGATGATCCAGCAAATAGGG

CCAAAACTTGATGGGAAAATCCCGGTGTCCAGAGGTCAGGAGTTTTCTGGGGAGTGAAGG

GAGGAGGGCTGCATTAACCTCATTGATCTGTACACTGAATAATTCCCCTTGATACCAGCT

CCCCATCTCAAATACTTTCTGGTTCTCTTCATCACCTTAATTTTTCCACCAGCCTTGGTC

TGCACCCTGTGTTCTTTTGGTGGGCCGAAGTACCTAGCATGTAGTAGGCATTCAGAACTA

TGTATTGAATGTGATGAATTCAACAGGTACCAACTGATACCTACTGAATATTAACACTTG

TGCTACTATGCCCAGCAAGATGGATGGGAAGAGTGGAAATATCTGATGACGTGACTATGT

CTTAGTGAGAAGACAGTGCATGGTTAGACATCAATGTGAGCTCTAGACAGGAAGTGCTGA

AGGAAGTCATGGTGGAGGGGTCCAGAGTAGCCTGGATCTGGCTCTGCTTCTACGTCTAGC

TGCAGTCCTTGCCTGGAAGAAGACCTCCCTTCAGAGCCCAGCTCCTCGCTCATCTCTGTC

TCCCAAAGCCTGACCCACTGTGTTCTTCTCTGCCCTCCCCTCCATATGCCATGGCCCCTC

CAAACACAGAGATACCATCTAAACTAGTCTCTTTTTCCCCCTACACTTCAATCCCCCCAC

CAGGGTTTCCTATCGCTGAAGTGTTCACGCTGAAGCCCCTGGAGTTTGGCAAGCCCAACA

CTTTGGTCTGTTTTGTCAGTAATCTCTTCCCACCCATGCTGACAGTGAACTGGCAGCATC

ATTCCATCCCTGTGGAAGGATTTGGGCCTACTTTTGTCTCAGCTGTCGATGGACTCAGCT

TCCAGGCCTTTTCTTACTTAAACTTCACACCAGAACCTTCTGACATTTTCTCCTGCATTG

TGACTCACGAAATTGACCGCTACACAGCAATTGCCTATTGGGGTGAGGCTTTCTCCCTGG

AATTCTGGTCCTTTTGGGGGCAAAAAGGGATAGATCCATGGGAGGAGGCTTCTTTCTCCA

CTGGTACCTTGTTTAGTCCATTCCTACCCTAAGCCCATCCCAGTCTCCCATGTCATCCCA

GACACCCACGTCATTTCCCTGGGTGGGAGGCTCCCTAACTAGGTCCCCAGGCTGAGCCAC

TCATTTCCTCCAGTACCCCGGAACGCACTGCCCTCAGATCTGCTGGAGAATGTGCTGTGT

GGCGTGGCCTTTGGCCTGGGTGTGCTGGGCATCATCGTGGGCATTGTTCTCATCATCTAC

TTCCGGAAGCCTTGCTCAGGTGGTATGTCATCTGGAGGGGGCGGGTGAGCCTGTGGGAGC

CAGATACAGTGGTGCATGCGTGCATGTGTCAGGATTATTTTGTGGCATGGGGGGACATAT

AGCGATCCTCAGGCCCTTGGGTGTGGGGGCCTGTATCCAGCACCATGGGGGCACATCTTC

CCAGTTGGGGACCCAGTTACACACACACAGTTATGGGTCACAAGAATTGCTTTGAGTGAA

AAAAGGAATCATGGGGTGCTGTAGGAAGGGTGCTTGGAGATGATTTGGGAACAAGGAGAG

ATCAACTTCTGCAGGGTGTGGTGCAGGCAGGGCGAGCAAGGCCTTTGTGGGGGAGGAGGA

GGAGGAGAAGAAGGAGGAGGATGGATCCCCTGATGCCTTTCCTCCATCCCTGTCTCTCCC

CCAGACTGATTCTTCCAGACCAGAGTTTGATGCCAGCAGCTTCGGCCATCCAAACAGAGG

ATGCTCAGATTTCTCACATCCTGCCCAGGATCTCCTCTTAGGGTAGAAGAAGTCTCTGGG

ACATCCCTGGGGTGTGTGTGTAGATTTCCCACCTGGGGACTCTGCTGTCCCTGGGCTTGC

ATCCCAGGGATCCCAGAGTGGCCTGCCTATCACAACCACATCCCTTCCCCCCACAAGGCA

ATAAATCTCATTTCTTTATATCAGTGTGGCTTCTTTCTTAACTCATGGTATTTGTTTCTG

GATATCTCAACTTGAGTGGGTTGTCGTTTCAAATTCAGCATGCCTTAACCTGAACACAGC

TTGACCTCGTTAGGGAGGGAAATAGGGAAAACCCCTAATTTGCCAGCTGAGCTCTTATTC

CCTGGTCTTGGCGGTACATGATGTTTTTCCATCTATCGGTTTGTGCAAAATATGTGAGAA

ACGAAGGCAGAGTTATTTTCTAATAATCTGCTT

>HLA:HLA00489 DMB*01:01:01:01

CTAGTAACTGATGATGCTAGGCTGAGGCACTCAGTGATTTGTCTCTACATTTGTCCCTGC

CTACCTAGCCAATCTGTCCCTGTTTGGGACACTGGACTCCCGTGAGCTGGAAGGAACAGA

TTTAATATCTAGGGGCTGGGTATCCCCACATCACTCATTTGGGGGGTCAAGGGACCCGGG

CAATATAGTATTCTGCTCAGTGTCTGGAGATCATCTACCCAGGCTGGGGCTTCTGGGACA

GGCGAGGACCCACGGACCCTGGAAGAGCTGGTCCAGGGGACTGAACTCCCGGCATCTTTA

CAGAGCAGAGCATGATCACATTCCTGCCGCTGCTGCTGGGGCTCAGCCTGGGCTGCACAG

GAGCAGGTAAGGACACTTCTTCTGGGGACTCTCCCTTCCCCTGCTCCTGTTTCAGGGTAA

GGGTGTTCCGTTTTGTAATTGCATTTGACACCCCAGATAGTTTGTCTCCCTGGTATACAT

TCCTATAGCACTTTGTACTTTGTAGCAATTTTAATGTAATTAATCTGTATAATTATCTGT

GCAGTGTATATTCCCTGCTGGAATATAGGCAAGGACAATGTTCATCTTATTTATTGCTGC

CTCCTCAGCTCCTAGCACAGTGCCTTGCATGCAGCAAGTGCTTCATAAATATGTGCGAAG

TGAATATTTAATATTTCCAGCACAATACAAGGCTGACTCTTTCTCTTGACCCTTTTTCTC

TCTCAATAATTTGCCTTACTGAAGGTCTGTGTTCTGGGCAAATTGTCATGTTTAAACATG

CAAATAATCTCGGGGGGCTACTCCTATCCCTGTGCTTAGTCTTGCATAAAGAGGAGACTG

GATCTAAAAACTTATCTACTACTTCTACTGACTCCCTCAAATCAGACTTTCAGAAACTTC

AGTGTATGAGCTTGGTCAGTAGATGTTCCCTGAGCAGGAAATCTGTGCCAGACTAGCTGG

ATGTCACCAAGGCTTAGGTTCTGAGCTGAATATAGGAAAAATCAACTTTTTTTCTTCTAT

ATGCTCACACTCAACACTTCTTTGACCAACTGTGTGAGGTTTTTTTTTTTTTTTTACTCA

TACCAACCAATTCTCCTATATTAGCTGGATATCCTATAATTCAATTCCATTGTGACATTA

ACTAGAGTTAACATAGACACCAAAGGTTAAAGACTCAGTCCCATAAGACTGCCTCCATTT

CAGACACCAATCACAAGTAGTAGGTTCCCAAATTACCCACATCTTCTGTCCAACTTGCCT

ACAAATCAGAGGTTCCCATGACCCCCTCCTTGGGGTTGGTAATTTGCTAAAGTGGCTTAT

GGAACTCAGGAAAAGTTTACTTATTATTGTAGATTTTTTACAAAGGATATTTTAATTGAT

AATAATAATTATAAATATTCATGGGGTGGATAATGATGTTTTAGTACATGTAATGTACAG

TGATCAGATCAGATCATCATCTCATTTATCATTTCTTTGTGTTGGAAACATTCCATATCC

TTCTTCTAGCGATTTGAAATGATATAAAGTATTATTGTTAATGACAGTCATCCCACAGTG

GTATAGAACACTAGATCATAATTTTGCATCCTTTAACAAAGGATATTTTAAAGCATACAA

AGGAACATCCAGATGAAGAGATACCAGATCTGGAAGGGTCCCAATCACAGGAGCTCTGTC

CCCACAGAATTGGGGTACTTCACCTCCTGGCATGTGGATGTGTTTACCAACTGAGAAGTT

CTCTGAACTCCATAGTTCCGGGATTTTTATGGAGGCTTCATCATGTAGGCATGACTGATT

ATTAACTCAATCTCCAGCCCCTTCCCCTTCAGGGAGTATGGGGGATGGGACTAAAAGTTC

CAGACTTCTAATCATGACTTGGTCTTTCTGGTGACCAGCCCCTCCTGCAGGAGCCCACCA

AGAGTACCTCATTAGAACAAAAGACACTCCTGTTATCTAGGAAATTCTAAGCGATTAGGC

ACTCTATGTCAGGAACCAGGGTCAAAGACAAAGCTCTGTGCAGAGCTCCTAAATATACGT

CTGTATGTTTTATATATTTATTATTTGTGTATATTTATTATTTATATATTTATTTATTTA

TTGCCAGGCCAGTAGAAGACATTGACCTGTTCTCCCTTCCCTGGCTCCTCTAGGTGGCTT

CGTGGCCCATGTGGAAAGCACCTGTCTGTTGGATGATGCTGGGACTCCAAAGGATTTCAC

ATACTGCATCTCCTTCAACAAGGATCTGCTGACCTGCTGGGATCCAGAGGAGAATAAGAT

GGCCCCTTGCGAATTTGGGGTGCTGAATAGCTTGGCGAATGTCCTCTCACAGCACCTCAA

CCAAAAAGACACCCTGATGCAGCGCTTGCGCAATGGGCTTCAGAATTGTGCCACACACAC

CCAGCCCTTCTGGGGATCACTGACCAACAGGACACGTGAGGAGAGAGGGGTGCAGAGGGG

CTACCAGGAAGTGCAGTTAGGAGGGCAGGCCAGGGAGGATCCCACAGTGGCCCAGGGGTT

TGAGATTTGAGCAGCAAATAAGAGAAAATGTGTGGATCTGAAATGTAGAAAGACGGAGGA

TTGAACCTCAAGGGGAACAAGGTGGCTGACGTGAGTGGAACAGGAGTAAAGAAGGGGAGG

TGAGGCTTGAACCGCGAGGTGCCATGTGGGGAGCTTATGCAGAGGCTGGGGCATCTCAGG

ATGCATACCCAAGATGTTCTTGCCTTGTTATCCCAGATTTTGATGTTCCAGATCTGATGT

GGGCCCAGGCATGGGAATATTTGGAATCCCAGGGGATTCTGACACATGCTTTTTCTCACC

CTTAAACTCTTGCATTGACAATGGCTTGAAGTTTGTGAAAGTAAACTTGAAGATCTCCAC

AGTACAGAACAGTGTGTCTCAAGGGTGGTTTCTGAACCACCTTTCTCAGAATTGCCTGGA

GGAGGTGCTTGTTAAAGATGCAAGCCCCTTAGTACCACTCCAGATCTGTTGAAAGAAAGT

ATCTGGGGATACAGCCTAGGAAATCTGCATTTTAACATAATTCCTTGGATTTTTATTTAA

GATTGTGTTTGAAAAATGTCAAGATAGAGGCAAGCAAGAGGATCACCTAGGAGAGTAAAT

TAGTAAAAGATGGCAGTATTAGCAATCTCTTTAGTTTGACTACATTCATTCCAAATTTAA

GAGTGAGTCCTAAGTTAGGCTTGTTTCCTTGAACTATGTGAGGAGAAAAAGCTTTAACTA

GCAAAAGAACGTATTAAACAAGATTTGGAGAAAAATTCCTTTTCCACCTTAAAAAAACCC

AATGTACAACTCTGGATTACTCTTAGCTTCCTTATTTCAAATACTTTCCAGTTTATGTAC

TTGAAATAAATACAACAACTTCTAGAACAGCTTGCAGTTCAGATCTGGCTTTTACTAATT

GTAATCAAACATAATTCTGGAGGAGGAAAGAAAGAAAGGGGCAATGAAGGAATGGGAGAG

AAGAAAGAGTAATGCAGGAATACATTCTAACGGTTCCCCTTCAAGGGGCAGCATGGCAGA

GGGGGCTGGGGTGGAAAGTGGGTTGCAAAATCTACGAAGAGTTGCGATAGGGAAGAAACC

AGGTTGAGGAAGCAGCCAGAATGTCACCCTCCTTCCTAAACATGTTTTTTTCTCCTATGC

AGGGCCACCATCTGTGCAAGTAGCCAAAACCACTCCTTTTAACACGAGGGAGCCTGTGAT

GCTGGCCTGCTATGTGTGGGGCTTCTATCCAGCAGAAGTGACTATCACGTGGAGGAAGAA

CGGGAAGCTTGTCATGCCTCACAGCAGTGCGCACAAGACTGCCCAGCCCAATGGAGACTG

GACATACCAGACCCTCTCCCATTTAGCCTTAACCCCCTCTTACGGGGACACTTACACCTG

TGTGGTAGAGCACATTGGGGCTCCTGAGCCCATCCTTCGGGACTGGAGTAAGTGTATGGC

AGATGGATGGAATTAGGGTCAAAGCAGAGAAAATGAGATGTGGATCGATACATGGTACAT

GGTAGACAGCGAAGTGCTGAAAATGGGGACTGAGTCTGGAGGAACTTACGGGGGGCTTAG

GACCAGAATGGGGAAATGGGATAAAGAAATGGAAATATTTAGGTTGGTGCAAAAGTAATT

GCAGTTTTTGCCATTACTTTCAGTGGCAAAAACCGCAATTACTTTTGCACCAGTTTAATA

TTTAGTCTGTGCTATTGCTGCTCTGGTGGTGTGGTTGATGTTGCTGCGTCTATGTTTGAG

GGTGAGAGGGGAGCGTGCTTGCTTTGAAATGAGGATGTAAATTTGGCAATCATATTTTCA

GAACCCCAAATTGTAATACACTATTCTAGCCTCCTTAGATTTCAACTATTCTGGTGCCAG

AAGCAGATGGGAGCTGAAGGAATGATGAAGGTTGAAGAAGGGGGGCTTTTCTTGGTGTGG

GGCAGTACTGCATTTGGCCTGCTCTACCAAGCATACGGGAGTAGTAAAGCCACGGCTGGC

AGACCATTTGGCATGCATGCTCAGGGGCCAGTGGATAAAGAATTACTTACAGTTCAAACA

CTGTTTGAACTCAGTGTCGGGAGTAGTTAAAGGTATCGTGAGAAGTTGCACACAGCTTTG

GGGACTCTTGGAAAAGAAAGAGGAAGAAATGAGGAAGAGGAAGGGTGTCTACAAAGGGCC

AGAGAACAGGATCTCAGATCAGCTGCTGTAACCAGGTTTCCCCTTGTGGGAAGTGTTGTT

TCTTGCTGGGCAGTTGGGAAGGGAATGGAGAACAGAGAAGAGAGTGGAAATCACATGCTC

ACTTGAACTTTCCTGGGGAACGTCTCCTCACAGCGTGCACAAGAGCCTCCCTTTAGAAAT

GGAGTGTTCATTTTATCATGGGAAAAGAATCTGAGTGGGACATGATTCAGAACAGGACCG

GCCCAAGGAAGTGCAGGGGCTGTGGAGTGGGATGGAGACAAGCTCTGAAAGGACACATGG

GAGATCTAGATGTAGAAGGTACACAAGTAGTAGGATAACTCACAGGATGGATCCACTGGA

GGTTAAGACATGTGGTAAGACAGTGTAATAGGAAGCTGCTCAGTTGGAGAAAGTAAGGAA

GCAAACATTGTTACCGTGGGGGCAATGGAGAGGACAGTGAGGAGCCCTTTATCCTGATAA

GGGTGGCTTTGAGGTAAAGGAAGGAAAGAGGATGCCTTGAGAGGCCCCACTGTATTAGAG

AGGACCTGGAAGCCAGGATGCTAATTCTGGGGAGATGGATTCCCCAGGCTTACTCTAGGA

GTAGAGGTCCATGGGACGAGGGTTTGATTTGAGAAAGATCATTTTCTTGGGAGTGGGTGG

TGTGAGCTAGACCCTTGGAGCTGGGATAAAGGACCTTTTAACCCACTGAGAGGTGGCTGC

AATAAATGGAATTGCCCTGGGGGTGAGCAACAGAAACTGGGTCAAGTAAGTTTCTATTTT

TTGCAGCACCTGGGCTGTCCCCCATGCAGACCCTGAAGGTTTCTGTGTCTGCAGTGACTC

TGGGCCTGGGCCTCATCATCTTCTCTCTTGGTGTGATCAGCTGGCGGAGAGCTGGCCACT

CTAGTGAGTGACTCGCTGAACTCCCATCCCCACTCTTGGTCCCACTCTCTGCTTACTTTC

TGTTTGTGATTAACTCTCTCCTTCCTACTGCATTTGCTATGAATACTGCTAGATATTTTC

ATCCACAAAGACTGGTATAATCAAGTATCTTCCTCTCTTAGGTTACACTCCTCTTCCTGG

GTCCAATTATTCAGAAGGTAACATCTCTGTTGGTCTGTTTCCCTACTTGCCCTTTGGTAG

GGGTGCGGGTTAGAGGGGTCAGTGTTGGGTTCAACTAATCTTGATTATTATATGGGTGAG

CTTCCATGAGGATCTAGGCAAGGGCATGATTTAAGCTGCCATTGCTAGGATTAAGAGCAG

GAAGGAGCATCCTCCTCTTCTACCAAGTGGGATGTCTGTGGAGAGGAGGCTGAAGGTGCT

TCCTTTGTATTAGTTGTTGGTGCCCTGGAGTTTTCAGTATCACTGTATTAAGGCATGGGA

TGGTTACAGTGACAAACGATGGGGGCAAGTTGGGTTGAAGCCTCATTATCTCCCTTTTAT

TTATTCTGTAGGATGGCACATTTCCTAGAGGCAGAATCCTACAACTTCCACTCCAAGTGA

GAAGGAGATTCAAACTCAATGATGCTACCATGCCTCTCCAACATCTTCAACCCCCTGACA

TTATCTTGGATCCTATGGTTTCTCCATCCAATTCTTTGAATTTCCCAGTCTCCCCTATGT

AAAACTTAGCAACTTGGGGGACCTCATTCCTGGGACTATGCTGTAACCAAATTATTGTCC

AAGGCTATATTTCTGGGATGAATATAATCTGAGGAAGGGAGTTAAAGACCCTCCTGGGGC

TCTCAGTGTGCCATAGAGGACAGCAACTGGTGATTGTTTCAGAGAAATAAACTTTGGTGG

AAATATTGTTTTTCCATGTCTTCTTCCTGGGGCCCTGGGGAAGGAATATGGGCAAAGCAG

GGACTGAGGTTAATTCTCTTCTGCTTGAGTAGGGGAGAAATCAATGCCTTCTTCCATTTT

CCCACTTAGACATGACAGAATTTGGGGCCGTTTTCTGATTTATAATTCATAAGGAGAAAT

TCAACTGTGGTGGGTTGGAGTCACAGAGTATGGGCAAGGAAGGGAATTAACAGCTTACTC

ACCTCATACAGGATCTTATG

>HLA:HLA06461 DMB*01:01:01:02

CTAGTAACTGATGATGCTAGGCTGAGGCACTCAGTGATTTGTCTCTACATTTGTCCCTGC

CTACCTAGCCAATCTGTCCCTGTTTGGGACACTGGACTCCCGTGAGCTGGAAGGAACAGA

TTTAATATCTAGGGGCTGGGTATCCCCACATCACTCATTTGGGGGGTCAAGGGACCCGGG

CAATATAGTATTCTGCTCAGTGTCTGGAGATCATCTACCCAGGCTGGGGCTTCTGGGACA

GGCGAGGACCCACGGACCCTGGAAGAGCTGGTCCAGGGGACTGAACTCCCGGCATCTTTA

CAGAGCAGAGCATGATCACATTCCTGCCGCTGCTGCTGGGGCTCAGCCTGGGCTGCACAG

GAGCAGGTAAGGACACTTCTTCTGGGGACTCTCCCTTCCCCTGCTCCTGTTTCAGGGTAA

GGGTGTTCCGTTTTGTAATTGCATTTGACACCCCAGATAGTTTGTCTCCCTGGTATACAT

TCCTATAGCACTTTGTACTTTGTAGCAATTTTAATGTAATTAATCTGTATAATTATCTGT

GCAGTGTATATTCCCTGCTGGAATATAGGCAAGGACAATGTTCATCTTATTTATTGCTGC

CTCCTCAGCTCCTAGCACAGTGCCTTGCATGCAGCAAGTGCTTCATAAATATGTGCGAAG

TGAATATTTAATATTTCCAGCACAATACAAGGCTGACTCTTTCTCTTGACCCTTTTTCTC

TCTCAATAATTTGCCTTACTGAAGGTCTGTGTTCTGGGCAAATTGTCATGTTTAAACATG

CAAATAATCTCGGGGGGCTACTCCTATCCCTGTGCTTAGTCTTGCATAAAGAGGAGACTG

GATCTAAAAACTTATCTACTACTTCTACTGACTCCCTCAAATCAGACTTTCAGAAACTTC

AGTGTATGAGCTTGGTCAGTAGATGTTCCCTGAGCAGGAAATCTGTGCCAGACTAGCTGG

ATGTCACCAAGGCTTAGGTTCTGAGCTGAATATAGGAAAAATCAACTTTTTTTCTTCTAT

ATGCTCACACTCAACACTTCTTTGACCAACTGTGTGAGGTTTTTTTTTTTTTTTTACTCA

TACCAACCAATTCTCCTATATTAGCTGGATATCCTATAATTCAATTCCATTGTGACATTA

ACTAGAGTTAACATAGACACCAAAGGTTAAAGACTCAGTCCCATAAGACTGCCTCCATTT

CAGACACCAATCACAAGTAGTAGGTTCCCAAATTACCCACATCTTCTGTCCAACTTGCCT

ACAAATCAGAGGTTCCCATGACCCCCTCCTTGGGGTTGGTAATTTGCTAAAGTGGCTTAT

GGAACTCAGGAAAAGTTTACTTATTATTGTAGATTTTTTACAAAGGATATTTTAATTGAT

AATAATAATTATAAATATTCATGGGGTGGATAATGATGTTTTAGTACATGTAATGTACAG

TGATCAGATCAGATCATCATCTCATTTATCATTTCTTTGTGTTGGAAACATTCCATATCC

TTCTTCTAGCGATTTGAAATGATATAAAGTATTATTGTTAATGACAGTCATCCCACAGTG

GTATAGAACACTAGATCATAATTTTGCATCCTTTAACAAAGGATATTTTAAAGCATACAA

AGGAACATCCAGATGAAGAGATACCAGATCTGGAAGGGTCCCAATCACAGGAGCTCTGTC

CCCACAGAATTGGGGTACTTCACCTCCTGGCATGTGGATGTGTTTACCAACTGAGAAGTT

CTCTGAACTCCATAGTTCCGGGATTTTTATGGAGGCTTCATCATGTAGGCATGACTGATT

ATTAACTCAATCTCCAGCCCCTTCCCCTTCAGGGAGTATGGGGGATGGGACTAAAAGTTC

CAGACTTCTAATCATGACTTGGTCTTTCTGGTGACCAGCCCCTCCTGCAGGAGCCCACCA

AGAGTACCTCATTAGAACAAAAGACACTCCTGTTATCTAGGAAATTCTAAGCGATTAGGC

ACTCTATGTCAGGAACCAGGGTCAAAGACAAAGCTCTGTGCAGAGCTCCTAAATATACGT

CTGTATGTTTTATATATTTATTATTTGTGTATATTTATTATTTATATATTTATTTATTTA

TTGCCAGGCCAGTAGAAGACATTGACCTGTTCTCCCTTCCCTGGCTCCTCTAGGTGGCTT

CGTGGCCCATGTGGAAAGCACCTGTCTGTTGGATGATGCTGGGACTCCAAAGGATTTCAC

ATACTGCATCTCCTTCAACAAGGATCTGCTGACCTGCTGGGATCCAGAGGAGAATAAGAT

GGCCCCTTGCGAATTTGGGGTGCTGAATAGCTTGGCGAATGTCCTCTCACAGCACCTCAA

CCAAAAAGACACCCTGATGCAGCGCTTGCGCAATGGGCTTCAGAATTGTGCCACACACAC

CCAGCCCTTCTGGGGATCACTGACCAACAGGACACGTGAGGAGAGAGGGGTGCAGAGGGG

CTACCAGGAAGTGCAGTTAGGAGGGCAGGCCAGGGAGGATCCCACAGTGGCCCAGGGGTT

TGAGATTTGAGCAGCAAATAAGAGAAAATGTGTGGATCTGAAATGTAGAAAGACGGAGGA

TTGAACCTCAAGGGGAACAAGGTGGCTGACGTGAGTGGAACAGGAGTAAAGAAGGGGAGG

TGAGGCTTGAACCGCGAGGTGCCATGTGGGGAGCTTATGCAGAGGCTGGGGCATCTCAGG

ATGCATACCCAAGATGTTCTTGCCTTGTTATCCCAGATTTTGATGTTCCAGATCTGATGT

GGGCCCAGGCATGGGAATATTTGGAATCCCAGGGGATTCTGACACATGCTTTTTCTCACC

CTTAAACTCTTGCATTGACAATGGCTTGAAGTTTGTGAAAGTAAACTTGAAGATCTCCAC

AGTACAGAACAGTGTGTCTCAAGGGTGGTTTCTGAACCACCTTTCTCAGAATTGCCTGGA

GGAGGTGCTTGTTAAAGATGCAAGCCCCTTAGTACCACTCCAGATCTGTTGAAAGAAAGT

ATCTGGGGATACAGCCTAGGAAATCTGCATTTTAACATAATTCCTTGGATTTTTATTTAA

GATTGTGTTTGAAAAATGTCAAGATAGAGGCAAGCAAGAGGATCACCTAGGAGAGTAAAT

TAGTAAAAGATGGCAGTATTAGCAATCTCATTAGTTTGACTACATTCATTCCAAATTTAA

GAGTGAGTCCTAAGTTAGGCTTGTTTCCTTGAACTATGTGAGGAGAAAAAGCTTTAACTA

GCAAAAGAACGTATTAAACAAGATTTGGAGAAAAATTCCTTTTCCACCTTAAAAAAACCC

AATGTACAACTCTGGATTACTCTTAGCTTCCTTATTTCAAATACTTTCCAGTTTATGTAC

TTGAAATAAATACAACAACTTCTAGAACAGCTTGCAGTTCAGATCTGGCTTTTACTAATT

GTAATCAAACATAATTCTGGAGGAGGAAAGAAAGAAAGGGGCAATGAAGGAATGGGAGAG

AAGAAAGAGTAATGCAGGAATACATTCTAACGGTTCCCCTTCAAGGGGCAGCATGGCAGA

GGGGGCTGGGGTGGAAAGTGGGTTGCAAAATCTACGAAGAGTTGCGATAGGGAAGAAACC

AGGTTGAGGAAGCAGCCAGAATGTCACCCTCCTTCCTAAACATGTTTTTTTCTCCTATGC

AGGGCCACCATCTGTGCAAGTAGCCAAAACCACTCCTTTTAACACGAGGGAGCCTGTGAT

GCTGGCCTGCTATGTGTGGGGCTTCTATCCAGCAGAAGTGACTATCACGTGGAGGAAGAA

CGGGAAGCTTGTCATGCCTCACAGCAGTGCGCACAAGACTGCCCAGCCCAATGGAGACTG

GACATACCAGACCCTCTCCCATTTAGCCTTAACCCCCTCTTACGGGGACACTTACACCTG

TGTGGTAGAGCACATTGGGGCTCCTGAGCCCATCCTTCGGGACTGGAGTAAGTGTATGGC

AGATGGATGGAATTAGGGTCAAAGCAGAGAAAATGAGATGTGGATCGATACATGGTACAT

GGTAGACAGCGAAGTGCTGAAAATGGGGACTGAGTCTGGAGGAACTTACGGGGGGCTTAG

GACCAGAATGGGGAAATGGGATAAAGAAATGGAAATATTTAGGTTGGTGCAAAAGTAATT

GCAGTTTTTGCCATTACTTTCAGTGGCAAAAACCGCAATTACTTTTGCACCAGTTTAATA

TTTAGTCTGTGCTATTGCTGCTCTGGTGGTGTGGCTGATGTTGCTGCGTCTATGTTTGAG

GGTGAGAGGGGAGCGTGCTTGCTTTGAAATGAGGCTGTAAATTTGGCAATCATATTTTCA

GAACCCCAAATTGTAATACACTATTCTAGCCTCCTTAGATTTCAACTATTCTGGTGCCAG

AAGCAGATGGGAGCTGAAGGAATGATGAAGGTTGAAGAAGGGGGGCTTTTCTTGGTGTGG

GGCAGTACTGCATTTGGCCTGCTCTACCAAGCATACGGGAGTAGTAAAGCCACGGCTGGC

AGACCATTTGGCATGCATGCTCAGGGGCCAGTGGATAAAGAATTACTTACAGTTCAAACA

CTGTTTGAACTCAGTGTCGGGAGTAGTTAAAGGTATCGTGAGAAGTTGCACACAGCTTTG

GGGACTCTTGGAAAAGAAAGAGGAAGAAATGAGGAAGAGGAAGGGTGTCTACAAAGGGCC

AGAGAACAGGATCTCAGATCAGCTGCTGTAACCAGGTTTCCCCTTGTGGGAAGTGTTGTT

TCTTGCTGGGCAGTTGGGAAGGGAATGGAGAACAGAGAAGAGAGTGGAAATCACATGCTC

ACTTGAACTTTCCTGGGGAACGTCTCCTCACAGCGTGCACAAGAGCCTCCCTTTAGAAAT

GGAGTGTTCATTTTATCATGGGAAAAGAATCTGAGTGGGACATGATTCAGAACAGGACCG

GCCCAAGGAAGTGCAGGGGCTGTGGAGTGGGATGGAGACAAGCTCTGAAAGGACACATGG

GAGATCTAGATGTAGAAGGTACACAAGTAGTAGGATAACTCACAGGATGGATCCACTGGA

GGTTAAGACATGTGGTAAGACAGTGTAATAGGAAGCTGCTCAGTTGGAGAAAGTAAGGAA

GCAAACATTGTTACCGTGGGGGCAATGGAGAGGACAGTGAGGAGCCCTTTATCCTGATAA

GGGTGGCTTTGGGGTAAAGGAAGGAAAGAGGATGCCTTGAGAGGCCCCACTGTATTAGAG

AGGACCTGGAAGCCAGGATGCTAATTCTGGGGAGATGGGTTCCCCAGGCTTACTCTAGGA

GTAGAGGTCCATGGGACGAGGGTTTGATTTGAGAAAGATCATTTTCTTGGGAGTGGGTGG

TGTGAGCTAGACCCTTGGAGCTGGGATAAAGGACCTTTTAACCCACTGAGAGGTGGCTGC

AATAAATGGAATTGCCCTGGGGGTGAGCAACAGAAACTGGGTCAAGTAAGTTTCTATTTT

TTGCAGCACCTGGGCTGTCCCCCATGCAGACCCTGAAGGTTTCTGTGTCTGCAGTGACTC

TGGGCCTGGGCCTCATCATCTTCTCTCTTGGTGTGATCAGCTGGCGGAGAGCTGGCCACT

CTAGTGAGTGACTCGCTGAACTCCCATCCCCACTCTTGGTCCCACTCTCTGCTTACTTTC

TGTTTGTGATTAACTCTCTCCTTCCTACTGCATTTGCTATGAATACTGCTAGATATTTTC

ATCCACAAAGACTGGTATAATCAAGTATCTTCCTCTCTTAGGTTACACTCCTCTTCCTGG

GTCCAATTATTCAGAAGGTAACATCTCTGTTGGTCTGTTTCCCTACTTGCCCTTTGGTAG

GGGTGCGGGTTAGAGGGGTCAGTGTTGGGTTCAACTAATCTTGATTATTATATGGGTGAG

CTTCCATGAGGATCTAGGCAAGGGCATGATTTAAGCTGCCATTGCTAGGATTAAGAGCAG

GAAGGAGCATCCTCCTCTTCTACCAAGTGGGATGTCTGTGGAGAGGAGGCTGAAGGTGCT

TCCTTTGTATTAGTTGTTGGTGCCCTGGAGTTTTCAGTATCACTGTATTAAGGCATGGGA

TGGTTACAGTGACAAACGATGGGGGCAAGTTGGGTTGAAGCCTCATTATCTCCCTTTTAT

TTATTCTGTAGGATGGCACATTTCCTAGAGGCAGAATCCTACAACTTCCACTCCAAGTGA

GAAGGAGATTCAAACTCAATGATGCTACCATGCCTCTCCAACATCTTCAACCCCCTGACA

TTATCTTGGATCCTATGGTTTCTCCATCCAATTCTTTGAATTTCCCAGTCTCCCCTATGT

AAAACTTAGCAACTTGGGGGACCTCATTCCTGGGACTATGCTGTAACCAAATTATTGTCC

AAGGCTATATTTCTGGGATGAATATAATCTGAGGAAGGGAGTTAAAGACCCTCCTGGGGC

TCTCAGTGTGCCATAGAGGACAGCAACTGGTGATTGTTTCAGAGAAATAAACTTTGGTGG

AAATATTGTTTTTCCATGTCTTCTTCCTGGGGCCCTGGGGAAGGAATATGGGCAAAGCAG

GGACTGAGGTTAATTCTCTTCTGCTTGAGTAGGGGAGAAATCAATGCCTTCTTCCATTTT

CCCACTTAGACATGACAGAATTTGGGGCCGTTTTCTGATTTATAATTCATAAGGAGAAAT

TCAACTGTGGTGGGTTGGAGTCACAGAGTATGGGCAAGGAAGGGAATTAACAGCTTACTC

ACCTCATACAGGATCTTATG

>HLA:HLA06462 DMB*01:01:01:03

CTAGTAACTGATGATGCTAGGCTGAGGCACTCAGTGATTTGTCTCTACATTTGTCCCTGC

CTACCTAGCCAATCTGTCCCTGTTTGGGACACTGGACTCCCGTGAGCTGGAAGGAACAGA

TTTAATATCTAGGGGCTGGGTATCCCCACATCACTCATTTGGGGGGTCAAGGGACCCGGG

CAATATAGTATTCTGCTCAGTGTCTGGAGATCATCTACCCAGGCTGGGGCTTCTGGGACA

GGCGAGGACCCACGGACCCTGGAAGAGCTGGTCCAGGGGACTGAACTCCCGGCATCTTTA

CAGAGCAGAGCATGATCACATTCCTGCCGCTGCTGCTGGGGCTCAGCCTGGGCTGCACAG

GAGCAGGTAAGGACACTTCTTCTGGGGACTCTCCCTTCCCCTGCTCCTGTTTCAGGGTAA

GGGTGTTCCGTTTTGTAATTGCATTTGACACCCCAGATAGTTTGTCTCCCTGGTATACAT

TCCTATAGCACTTTGTACTTTGTAGCAATTTTAATGTAATTAATCTGTATAATTATCTGT

GCAGTGTATATTCCCTGCTGGAATATAGGCAAGGACAATGTTCATCTTATTTATTGCTGC

CTCCTCAGCTCCTAGCACAGTGCCTTGCATGCAGCAAGTGCTTCATAAATATGTGCGAAG

TGAATATTTAATATTTCCAGCACAATACAAGGCTGACTCTTTCTCTTGACCCTTTTTCTC

TCTCAATAATTTGCCTTACTGAAGGTCTGTGTTCTGGGCAAATTGTCATGTTTAAACATG

CAAATAATCTCGGGGGGCTACTCCTATCCCTGTGCTTAGTCTTGCATAAAGAGGAGACTG

GATCTAAAAACTTATCTACTACTTCTACTGACTCCCTCAAATCAGACTTTCAGAAACTTC

AGTGTATGAGCTTGGTCAGTAGATGTTCCCTGAGCAGGAAATCTGTGCCAGACTAGCTGG

ATGTCACCAAGGCTTAGGTTCTGAGCTGAATATAGGAAAAATCAACTTTTTTTCTTCTAT

ATGCTCACACTCAACACTTCTTTGACCAACTGTGTGAGGTTTTTTTTTTTTTTTTACTCA

TACCAACCAATTCTCCTATATTAGCTGGATATCCTATAATTCAATTCCATTGTGACATTA

ACTAGAGTTAACATAGACACCAAAGGTTAAAGACTCAGTCCCATAAGACTGCCTCCATTT

CAGACACCAATCACAAGTAGTAGGTTCCCAAATTACCCACATCTTCTGTCCAACTTGCCT

ACAAATCAGAGGTTCCCATGACCCCCTCCTTGGGGTTGGTAATTTGCTAAAGTGGCTTAT

GGAACTCAGGAAAAGTTTACTTATTATTGTAGATTTTTTACAAAGGATATTTTAATTGAT

AATAATAATTATAAATATTCATGGGGTGGATAATGATGTTTTAGTACATGTAATGTACAG

TGATCAGATCAGATCATCATCTCATTTATCATTTCTTTGTGTTGGAAACATTCCATATCC

TTCTTCTAGCGATTTGAAATGATATAAAGTATTATTGTTAATGACAGTCATCCCACAGTG

GTATAGAACACTAGATCATAATTTTGCATCCTTTAACAAAGGATATTTTAAAGCATACAA

AGGAACATCCAGATGAAGAGATACCAGATCTGGAAGGGTCCCAATCACAGGAGCTCTGTC

CCCACAGAATTGGGGTACTTCACCTCCTGGCATGTGGATGTGTTTACCAACTGAGAAGTT

CTCTGAACTCCATAGTTCCGGGATTTTTATGGAGGCTTCATCATGTAGGCATGACTGATT

ATTAACTCAATCTCCAGCCCCTTCCCCTTCAGGGAGTATGGGGGATGGGACTAAAAGTTC

CAGACTTCTAATCATGACTTGGTCTTTCTGGTGACCAGCCCCTCCTGCAGGAGCCCACCA

AGAGTACCTCATTAGAACAAAAGACACTCCTGTTATCTAGGAAATTCTAAGCGATTAGGC

ACTCTATGTCAGGAACCAGGGTCAAAGACAAAGCTCTGTGCAGAGCTCCTAAATATACGT

CTGTATGTTTTATATATTTATTATTTGTGTATATTTATTATTTATATATTTATTTATTTA

TTGCCAGGCCAGTAGAAGACATTGACCTGTTCTCCCTTCCCTGGCTCCTCTAGGTGGCTT

CGTGGCCCATGTGGAAAGCACCTGTCTGTTGGATGATGCTGGGACTCCAAAGGATTTCAC

ATACTGCATCTCCTTCAACAAGGATCTGCTGACCTGCTGGGATCCAGAGGAGAATAAGAT

GGCCCCTTGCGAATTTGGGGTGCTGAATAGCTTGGCGAATGTCCTCTCACAGCACCTCAA

CCAAAAAGACACCCTGATGCAGCGCTTGCGCAATGGGCTTCAGAATTGTGCCACACACAC

CCAGCCCTTCTGGGGATCACTGACCAACAGGACACGTGAGGAGAGAGGGGTGCAGAGGGG

CTACCAGGAAGTGCAGTTAGGAGGGCAGGCCAGGGAGGATCCCACAGTGGCCCAGGGGTT

TGAGATTTGAGCAGCAAATAAGAGAAAATGTGTGGATCTGAAATGTAGAAAGACGGAGGA

TTGAACCTCAAGGGGAACAAGGTGGCTGACGTGAGTGGAACAGGAGTAAAGAAGGGGAGG

TGAGGCTTGAACCGCGAGGTGCCATGTGGGGAGCTTATGCAGAGGCTGGGGCATCTCAGG

ATGCATACCCAAGATGTTCTTGCCTTGTTATCCCAGATTTTGATGTTCCAGATCTGATGT

GGGCCCAGGCATGGGAATATTTGGAATCCCAGGGGATTCTGACACATGCTTTTTCTCACC

CTTAAACTCTTGCATTGACAATGGCTTGAAGTTTGTGAAAGTAAACTTGAAGATCTCCAC

AGTACAGAACAGTGTGTCTCAAGGGTGGTTTCTGAACCACCTTTCTCAGAATTGCCTGGA

GGAGGTGCTTGTTAAAGATGCAAGCCCCTTAGTACCACTCCAGATCTGTTGAAAGAAAGT

ATCTGGGGATACAGCCTAGGAAATCTGCATTTTAACATAATTCCTTGGATTTTTATTTAA

GATTGTGTTTGAAAAATGTCAAGATAGAGGCAAGCAAGAGGATCACCTAGGAGAGTAAAT

TAGTAAAAGATGGCAGTATTAGCAATCTCTTTAGTTTGACTACATTCATTCCAAATTTAA

GAGTGAGTCCTAAGTTAGGCTTGTTTCCTTGAACTATGTGAGGAGAAAAAGCTTTAACTA

GCAAAAGAACGTATTAAACAAGATTTGGAGAAAAATTCCTTTTCCACCTTAAAAAAACCC

AATGTACAACTCTGGATTACTCTTAGCTTCCTTATTTCAAATACTTTCCAGTTTATGTAC

TTGAAATAAATACAACAACTTCTAGAACAGCTTGCAGTTCAGATCTGGCTTTTACTAATT

GTAATCAAACATAATTCTGGAGGAGGAAAGAAAGAAAGGGGCAATGAAGGAATGGGAGAG

AAGAAAGAGTAATGCAGGAATACATTCTAACGGTTCCCCTTCAAGGGGCAGCATGGCAGA

GGGGGCTGGGGTGGAAAGTGGGTTGCAAAATCTACGAAGAGTTGCGATAGGGAAGAAACC

AGGTTGAGGAAGCAGCCAGAATGTCACCCTCCTTCCTAAACATGTTTTTTTCTCCTATGC

AGGGCCACCATCTGTGCAAGTAGCCAAAACCACTCCTTTTAACACGAGGGAGCCTGTGAT

GCTGGCCTGCTATGTGTGGGGCTTCTATCCAGCAGAAGTGACTATCACGTGGAGGAAGAA

CGGGAAGCTTGTCATGCCTCACAGCAGTGCGCACAAGACTGCCCAGCCCAATGGAGACTG

GACATACCAGACCCTCTCCCATTTAGCCTTAACCCCCTCTTACGGGGACACTTACACCTG

TGTGGTAGAGCACATTGGGGCTCCTGAGCCCATCCTTCGGGACTGGAGTAAGTGTATGGC

AGATGGATGGAATTAGGGTCAAAGCAGAGAAAATGAGATGTGGATCGATACATGGTACAT

GGTAGACAGCGAAGTGCTGAAAATGGGGACTGAGTCTGGAGGAACTTACGGGGGGCTTAG

GACCAGAATGGGGAAATGGGATAAAGAAATGGAAATATTTAGGTTGGTGCAAAAGTAATT

GCAGTTTTTGCCATTACTTTCAGTGGCAAAAACCGCAATTACTTTTGCACCAGTTTAATA

TTTAGTCTGTGCTATTGCTGCTCTGGTGGTGTGGTTGATGTTGCTGCGTCTATGTTTGAG

GGTGAGAGGGGAGCGTGCTTGCTTTGAAATGAGGATGTAAATTTGGCAATCATATTTTCA

GAACCCCAAATTGTAATACACTATTCTAGCCTCCTTAGATTTCAACTATTCTGGTGCCAG

AAGCAGATGGGAGCTGAAGGAATGATGAAGGTTGAAGAAGGGGGGCTTTTCTTGGTGTGG

GGCAGTACTGCATTTGGCCTGCTCTACCAAGCATACGGGAGTAGTAAAGCCACGGCTGGC

AGACCATTTGGCATGCATGCTCAGGGGCCAGTGGATAAAGAATTACTTACAGTTCAAACA

CTGTTTGAACTCAGTGTCGGGAGTAGTTAAAGGTATCGTGAGAAGTTGCACACAGCTTTG

GGGACTCTTGGAAAAGAAAGAGGAAGAAATGAGGAAGAGGAAGGGTGTCTACAAAGGGCC

AGAGAACAGGATCTCAGATCAGCTGCTGTAACCAGGTTTCCCCTTGTGGGAAGTGTTGTT

TCTTGCTGGGCAGTTGGGAAGGGAATGGAGAACAGAGAAGAGAGTGGAAATCACATGCTC

ACTTGAACTTTCCTGGGGAACGTCTCCTCACAGCGTGCACAAGAGCCTCCCTTTAGAAAT

GGAGTGTTCATTTTATCATGGGAAAAGAATCTGAGTGGGACATGATTCAGAACAGGACCG

GCCCAAGGAAGTGCAGGGGCTGTGGAGTGGGATGGAGACAAGCTCTGAAAGGACACATGG

GAGATCTAGATGTAGAAGGTACACAAGTAGTAGGATAACTCACAGGATGGATCCACTGGA

GGTTAAGACATGTGGTAAGACAGTGTAATAGGAAGCTGCTCAGTTGGAGAAAGTAAGGAA

GCAAACATTGTTACCGTGGGGGCAATGGAGAGGACAGTGAGGAGCCCTTTATCCTGATAA

GGGTGGCTTTGAGGTAAAGGAAGGAAAGAGGATGCCTTGAGAGGCCCCACTGTATTAGAG

AGGACCTGGAAGCCAGGATGCTAATTCTGGGGAGATGGATTCCCCAGGCTTACTCTAGGA

GTAGAGGTCCATGGGACGAGGGTTTGATTTGAGAAAGATCATTTTCTTGGGAGTGGGTGG

TGTGAGCTAGACCCTTGGAGCTGGGATAAAGGACCTTTTAACCCACTGAGAGGTGGCTGC

AATAAATGGAATTGCCCTGGGGGTGAGCAACAGAAACTGGGTCAAGTAAGTTTCTATTTT

TTGCAGCACCTGGGCTGTCCCCCATGCAGACCCTGAAGGTTTCTGTGTCTGCAGTGACTC

TGGGCCTGGGCCTCATCATCTTCTCTCTTGGTGTGATCAGCTGGCGGAGAGCTGGCCACT

CTAGTGAGTGACTCGCTGAACTCCCATCCCCACTCTTGGTCCCACTCTCTGCTTACTTTC

TGTTTGTGATTAACTCTCTCCTTCCTACTGCATTTGCTATGAATACTGCTAGATATTTTC

ATCCACAAAGACTGGTATAATCAAGTATCTTCCTCTCTTAGGTTACACTCCTCTTCCTGG

GTCCAATTATTCAGAAGGTAACATCTCTGTTGGTCTGTTTCCCTACTTGCCCTTTGGTAG

GGGTGCGGGTTAGAGGGGTCAGTGTTGGGTTCAACTAATCTTGATTATTATATGGGTGAG

CTTCCATGAGGATCTAGGCAAGGGCATGATTTAAGCTGCCATTGCTAGGATTAAGAGCAG

GAAGGAGCATCCTCCTCTTCTACCAAGTGGGATGTCTGTGGAGAGGAGGCTGAAGGTGCT

TCCTTTGTATTAGTTGTTGGTGCCCTGGAGTTTTCAGTATCACTGTATTAAGGCATGGGA

TGGTTACAGTGACAAACGATGGGGGCAAGTTGGGTTGAAGCCTCATTATCTCCCTTTTAT

TTATTCTGTAGGATGGCACATTTCCTAGAGGCAGAATCCTACAACTTCCACTCCAAGTGA

GAAGGAGATTCAAACTCAATGATGCTACCATGCCTCTCCAACATCTTCAACCCCCTGACA

TTATCTTGGATCCTATGGTTTCTCCATCCAATTCTTTGAATTTCCCAGTCTCCCCTATGT

AAAACTTAGCAATTTGGGGGACCTCATTCCTGGGACTATGCTGTAACCAAATTATTGTCC

AAGGCTATATTTCTGGGATGAATATAATCTGAGGAAGGGAGTTAAAGACCCTCCTGGGGC

TCTCAGTGTGCCATAGAGGACAGCAACTGGTGATTGTTTCAGAGAAATAAACTTTGGTGG

AAATATTGTTTTTCCATGTCTTCTTCCTGGGGCCCTGGGGAAGGAATATGGGCAAAGCAG

GGACTGAGGTTAATTCTCTTCTGCTTGAGTAGGGGAGAAATCAATGCCTTCTTCCATTTT

CCCACTTAGACATGACAGAATTTGGGGCCGTTTTCTGATTTATAATTCATAAGGAGAAAT

TCAACTGTGGTGGGTTGGAGTCACAGAGTATGGGCAAGGAAGGGAATTAACAGCTTACTC

ACCTCATACAGGATCTTATG

>HLA:HLA06466 DMB*01:01:01:04

CTAGTAACTGATGATGCTAGGCTGAGGCACTCAGTGATTTGTCTCTACATTTGTCCCTGC

CTACCTAGCCAATCTGTCCCTGTTTGGGACACTGGACTCCCGTGAGCTGGAAGGAACAGA

TTTAATATCTAGGGGCTGGGTATCCCCACATCACTCATTTGGGGGGTCAAGGGACCCGGG

CAATATAGTATTCTGCTCAGTGTCTGGAGATCATCTACCCAGGCTGGGGCTTCTGGGACA

GGCGAGGACCCACGGACCCTGGAAGAGCTGGTCCAGGGGACTGAACTCCCGGCATCTTTA

CAGAGCAGAGCATGATCACATTCCTGCCGCTGCTGCTGGGGCTCAGCCTGGGCTGCACAG

GAGCAGGTAAGGACACTTCTTCTGGGGACTCTCCCTTCCCCTGCTCCTGTTTCAGGGTAA

GGGTGTTCCGTTTTGTAATTGCATTTGACACCCCAGATAGTTTGTCTCCCTGGTATACAT

TCCTATAGCACTTTGTACTTTGTAGCAATTTTAATGTAATTAATCTGTATAATTATCTGT

GCAGTGTATATTCCCTGCTGGAATATAGGCAAGGACAATGTTCATCTTATTTATTGCTGC

CTCCTCAGCTCCTAGCACAGTGCCTTGCATGCAGCAAGTGCTTCATAAATATGTGCGAAG

TGAATATTTAATATTTCCAGCACAATACAAGGCTGACTCTTTCTCTTGACCCTTTTTCTC

TCTCAATAATTTGCCTTACTGAAGGTCTGTGTTCTGGGCAAATTGTCATGTTTAAACATG

CAAATAATCTCGGGGGGCTACTCCTATCCCTGTGCTTAGTCTTGCATAAAGAGGAGACTG

GATCTAAAAACTTATCTACTACTTCTACTGACTCCCTCAAATCAGACTTTCAGAAACTTC

AGTGTATGAGCTTGGTCAGTAGATGTTCCCTGAGCAGGAAATCTGTGCCAGACTAGCTGG

ATGTCACCAAGGCTTAGGTTCTGAGCTGAATATAGGAAAAATCAACTTTTTTTCTTCTAT

ATGCTCACACTCAACACTTCTTTGACCAACTGTGTGAGGTTTTTTTTTTTTTTTACTCAT

ACCAACCAATTCTCCTATATTAGCTGGATATCCTATAATTCAATTCCATTGTGACATTAA

CTAGAGTTAACATAGACACCAAAGGTTAAAGACTCAGTCCCATAAGACTGCCTCCATTTC

AGACACCAATCACAAGTAGTAGGTTCCCAAATTACCCACATCTTCTGTCCAACTTGCCTA

CAAATCAGAGGTTCCCATGACCCCCTCCTTGGGGTTGGTAATTTGCTAAAGTGGCTTATG

GAACTCAGGAAAAGTTTACTTATTATTGTAGATTTTTTACAAAGGATATTTTAATTGATA

ATAATAATTATAAATATTCATGGGGTGGATAATGATGTTTTAGTACATGTAATGTACAGT

GATCAGATCAGATCATCATCTCATTTATCATTTCTTTGTGTTGGAAACATTCCATATCCT

TCTTCTAGCGATTTGAAATGATATAAAGTATTATTGTTAATGACAGTCATCCCACAGTGG

TATAGAACACTAGATCATAATTTTGCATCCTTTAACAAAGGATATTTTAAAGCATAGAAA

GGAACATCCAGATGAAGAGATACCAGATCTGGAAGGGTCCCAATCACAGGAGCTCTGTCC

CCACAGAATTGGGGTACTTCACCTCCTGGCATGTGGATGTGTTTACCAACTGAGAAGTTC

TCTGAACTCCATAGTTCCGGGATTTTTATGGAGGCTTCATCATGTAGGCATGACTGATTA

TTAACTCAATCTCCAGCCCCTTCCCCTTCAGGGAGTATGGGGGATGGGACTAAAAGTTCC

AGACTTCTAATCATGACTTGGTCTTTCTGGTGACCAGCCCCTCCTGCAGGAGCCCACCAA

GAGTACCTCATTAGAACAAAAGACACTCCTGTTATCTAGGAAATTCTAAGCGATTAGGCA

CTCTATGTCAGGAACCAGGGTCAAAGACAAAGCTCTGTGCAGAGCTCCTAAATATACGTC

TGTATGTTTTATATATTTATTATTTGTGTATATTTATTATTTATATATTTATTTATTTAT

TGCCAGGCCAGTAGAAGACATTGACCTGTTCTCCCTTCCCTGGCTCCTCTAGGTGGCTTC

GTGGCCCATGTGGAAAGCACCTGTCTGTTGGATGATGCTGGGACTCCAAAGGATTTCACA

TACTGCATCTCCTTCAACAAGGATCTGCTGACCTGCTGGGATCCAGAGGAGAATAAGATG

GCCCCTTGCGAATTTGGGGTGCTGAATAGCTTGGCGAATGTCCTCTCACAGCACCTCAAC

CAAAAAGACACCCTGATGCAGCGCTTGCGCAATGGGCTTCAGAATTGTGCCACACACACC

CAGCCCTTCTGGGGATCACTGACCAACAGGACACGTGAGGAGAGAGGGGTGCAGAGGGGC

TACCAGGAAGTGCAGTTAGGAGGGCAGGCCAGGGAGGATCCCACAGTGGCCCAGGGGTTT

GAGATTTGAGCAGCAAATAAGAGAAAATGTGTGGATCTGAAATGTAGAAAGACGGAGGAT

TGAACCTCAAGGGGAACAAGGTGGCTGACGTGAGTGGAACAGGAGTAAAGAAGGGGAGGT

GAGGCTTGAACCGCGAGGTGCCATGTGGGGAGCTTATGCAGAGGCTGGGGCATCTCAGGA

TGCATACCCAAGATGTTCTTGCCTTGTTATCCCAGATTTTGATGTTCCAGATCTGATGTG

GGCCCAGGCATGGGAATATTTGGAATCCCAGGGGATTCTGACACATGCTTTTTCTCACCC

TTAAACTCTTGCATTGACAATGGCTTGAAGTTTGTGAAAGTAAACTTGAAGATCTCCACA

GTACAGAACAGTGTGTCTCAAGGGTGGTTTCTGAACCACCTTTCTCAGAATTGCCTGGAG

GAGGTGCTTGTTAAAGATGCAAGCCCCTTAGTACCACTCCAGATCTGTTGAAAGAAAGTA

TCTGGGGATACAGCCTAGGAAATCTGCATTTTAACATAATTCCTTGGATTTTTATTTAAG

ATTGTGTTTGAAAAATGTCAAGATAGAGGCAAGCAAGAGGATCACCTAGGAGAGTAAATT

AGTAAAAGATGGCAGTATTAGCAATCTCATTAGTTTGACTACATTCATTCCAAATTTAAG

AGTGAGTCCTAAGTTAGGCTTGTTTCCTTGAACTATGTGAGGAGAAAAAGCTTTAACTAG

CAAAAGAACGTATTAAACAAGATTTGGAGAAAAATTCCTTTTCCACCTTAAAAAAACCCA

ATGTACAACTCTGGATTACTCTTAGCTTCCTTATTTCAAATACTTTCCAGTTTATGTACT

TGAAATAAATACAACAACTTCTAGAACAGCTTGCAGTTCAGATCTGGCTTTTACTAATTG

TAATCAAACATAATTCTGGAGGAGGAAAGAAAGAAAGGGGCAATGAAGGAATGGGAGAGA

AGAAAGAGTAATGCAGGAATACATTCTAACGGTTCCCCTTCAAGGGGCAGCATGGCAGAG

GGGGCTGGGGTGGAAAGTGGGTTGCAAAATCTACGAAGAGTTGCGATAGGGAAGAAACCA

GGTTGAGGAAGCAGCCAGAATGTCACCCTCCTTCCTAAACATGTTTTTTTCTCCTATGCA

GGGCCACCATCTGTGCAAGTAGCCAAAACCACTCCTTTTAACACGAGGGAGCCTGTGATG

CTGGCCTGCTATGTGTGGGGCTTCTATCCAGCAGAAGTGACTATCACGTGGAGGAAGAAC

GGGAAGCTTGTCATGCCTCACAGCAGTGCGCACAAGACTGCCCAGCCCAATGGAGACTGG

ACATACCAGACCCTCTCCCATTTAGCCTTAACCCCCTCTTACGGGGACACTTACACCTGT

GTGGTAGAGCACATTGGGGCTCCTGAGCCCATCCTTCGGGACTGGAGTAAGTGTATGGCA

GATGGATGGAATTAGGGTCAAAGCAGAGAAAATGAGATGTGGATCGATACATGGTACATG

GTAGACAGCGAAGTGCTGAAAATGGGGACTGAGTCTGGAGGAACTTACGGGGGGCTTAGG

ACCAGAATGGGGAAATGGGATAAAGAAATGGAAATATTTAGGTTGGTGCAAAAGTAATTG

CAGTTTTTGCCATTACTTTCAGTGGCAAAAACCGCAATTACTTTTGCACCAGTTTAATAT

TTAGTCTGTGCTATTGCTGCTCTGGTGGTGTGGCTGATGTTGCTGCGTCTATGTTTGAGG

GTGAGAGGGGAGCGTGCTTGCTTTGAAATGAGGATGTAAATTTGGCAATCATATTTTCAG

AACCCCAAATTGTAATACACTATTCTAGCCTCCTTAGATTTCAACTATTCTGGTGCCAGA

AGCAGATGGGAGCTGAAGGAATGATGAAGGTTGAAGAAGGGGGGCTTTTCTTGGTGTGGG

GCAGTACTGCATTTGGCCTGCTCTACCAAGCATACGGGAGTAGTAAAGCCACGGCTGGCA

GACCATTTGGCATGCATGCTCAGGGGCCAGTGGATAAAGAATTACTTACAGTTCAAACAC

TGTTTGAACTCAGTGTCGGGAGTAGTTAAAGGTATCGTGAGAAGTTGCACACAGCTTTGG

GGACTCTTGGAAAAGAAAGAGGAAGAAATGAGGAAGAGGAAGGGTGTCTACAAAGGGCCA

GAGAACAGGATCTCAGATCAGCTGCTGTAACCAGGTTTCCCCTTGTGGGAAGTGTTGTTT

CTTGCTGGGCAGTTGGGAAGGGAATGGAGAACAGAGAAGAGAGTGGAAATCACATGCTCA

CTTGAACTTTCCTGGGGAACGTCTCCTCACAGCGTGCACAAGAGCCTCCCTTTAGAAATG

GAGTGTTCATTTTATCATGGGAAAAGAATCTGAGTGGGACATGATTCAGAACAGGACCGG

CCCAAGGAAGTGCAGGGGCTGTGGAGTGGGATGGAGACAAGCTCTGAAAGGACACATGGG

AGATCTAGATGTAGAAGGTACACAAGTAGTAGGATAACTCACAGGATGGATCCACTGGAG

GTTAAGACATGTGGTAAGACAGTGTAATAGGAAGCTGCTCAGTTGGAGAAAGTAAGGAAG

CAAACATTGTTACCGTGGGGGCAATGGAGAGGACAGTGAGGAGCCCTTTATCCTGATAAG

GGTGGCTTTGGGGTAAAGGAAGGAAAGAGGATGCCTTGAGAGGCCCCACTGTATTAGAGA

GGACCTGGAAGCCAGGATGCTAATTCTGGGGAGATGGGTTCCCCAGGCTTACTCTAGGAG

TAGAGGTCCATGGGACGAGGGTTTGATTTGAGAAAGATCATTTTCTTGGGAGTGGGTGGT

GTGAGCTAGACCCTTGGAGCTGGGATAAAGGACCTTTTAACCCACTGAGAGGTGGCTGCA

ATAAATGGAATTGCCCTGGGGGTGAGCAACAGAAACTGGGTCAAGTAAGTTTCTATTTTT

TGCAGCACCTGGGCTGTCCCCCATGCAGACCCTGAAGGTTTCTGTGTCTGCAGTGACTCT

GGGCCTGGGCCTCATCATCTTCTCTCTTGGTGTGATCAGCTGGCGGAGAGCTGGCCACTC

TAGTGAGTGACTCGCTGAACTCCCATCCCCACTCTTGGTCCCACTCTCTGCTTACTTTCT

GTTTGTGATTAACTCTCTCCTTCCTACTGCATTTGCTATGAATACTGCTAGATATTTTCA

TCCACAAAGACTGGTATAATCAAGTATCTTCCTCTCTTAGGTTACACTCCTCTTCCTGGG

TCCAATTATTCAGAAGGTAACATCTCTGTTGGTCTGTTTCCCTACTTGCCCTTTGGTAGG

GGTGCGGGTTAGAGGGGTCAGTGTTGGGTTCAACTAATCTTGATTATTATATGGGTGAGC

TTCCATGAGGATCTAGGCAAGGGCATGATTTAAGCTGCCATTGCTAGGATTAAGAGCAGG

AAGGAGCATCCTCCTCTTCTACCAAGTGGGATGTCTGTGGAGAGGAGGCTGAAGGTGCTT

CCTTTGTATTAGTTGTTGGTGCCCTGGAGTTTTCAGTATCACTGTATTAAGGCATGGGAT

GGTTACAGTGACAAACGATGGGGGCAAGTTGGGTTGAAGCCTCATTATCTCCCTTTTATT

TATTCTGTAGGATGGCACATTTCCTAGAGGCAGAATCCTACAACTTCCACTCCAAGTGAG

AAGGAGATTCAAACTCAATGATGCTACCATGCCTCTCCAACATCTTCAACCCCCTGACAT

TATCTTGGATCCTATGGTTTCTCCATCCAATTCTTTGAATTTCCCAGTCTCCCCTATGTA

AAACTTAGCAACTTGGGGGACCTCATTCCTGGGACTATGCTGTAACCAAATTATTGTCCA

AGGCTATATTTCTGGGATGAATATAATCTGAGGAAGGGAGTTAAAGACCCTCCTGGGGCT

CTCAGTGTGCCATAGAGGACAGCAACTGGTGATTGTTTCAGAGAAATAAACTTTGGTGGA

AATATTGTTTTTCCATGTCTTCTTCCTGGGGCCCTGGGGAAGGAATATGGGCAAAGCAGG

GACTGAGGTTAATTCTCTTCTGCTTGAGTAGGGGAGAAATCAATGCCTTCTTCCATTTTC

CCACTTAGACATGACAGAATTTGGGGCCGTTTTCTGATTTATAATTCATAAGGAGAAATT

CAACTGTGGTGGGTTGGAGTCACAGAGTATGGGCAAGGAAGGGAATTAACAGCTTACTCA

CCTCATACAGGATCTTATG

>HLA00490 DMB*01:02:01:01

CATAAGATCCTGTATGAGGTGAGTAAGCTGTTAATTCCCTTCCTTGCCCATACTCTGTGA

CTCCAACCCACCACAGTTGAATTTCTCCTTATGAATTATAAATCAGAAAACGGCCCCAAA

TTCTGTCATGTCTAAGTGGGAAAATGGAAGAAGGCATTGATTTCTCCCCTACTCAAGCAG

AAGAGAATTAACCTCAGTCCCTGCTTTGCCCATATTCCTTCCCCAGGGCCCCAGGAAGAA

GACATGGAAAAACAATATTTCCACCAAAGTTTATTTCTCTGAAACAATCACCAGTTGCTG

TCCTCTATGGCACACTGAGAGCCCCAGGAGGGTCTTTAACTCCCTTCCTCAGATTATATT

CATCCCAGAAATATAGCCTTGGACAATAATTTGGTTACAGCATAGTCCCAGGAATGAGGT

CCCCCAAGTTGCTAAGTTTTACATAGGGGAGACTGGGAAATTCAAAGAATTGGATGGAGA

AACCATAGGATCCAAGATAATGTCAGGGGGTTGAAGATGTTGGAGAGGCATGGTAGCATC

ATTGAGTTTGAATCTCCTTCTCACTTGGAGTGGAAGTTGTAGGATTCTGCCTCTAGGAAA

TGTGCCATCCTACAGAATAAATAAAAGGGAGATAATGAGGCTTCAACCCAACTTGCCCCC

ATCGTTTGTCACTGTAACCATCCCATGCCTTAATACAGTGATACTGAAAACTCCAGGGCA

CCAACAACTAATACAAAGGAAGCACCTTCAGCCTCCTCTCCACAGACATCCCACTTGGTA

GAAGAGGAGGATGCTCCTTCCTGCTCTTAATCCTAGCAATGGCAGCTTAAATCATGCCCT

TGCCTAGATCCTCATGGAAGCTCACCCATATAATAATCAAGATTAGTTGAACCCAACACT

GACCCCTCTAACCCGCACCCCTACCAAAGGGCAAGTAGGGAAACAGACCAACAGAGATGT

TACCTTCTGAATAATTGGACCCAGGAAGAGGAGTGTAACCTAAGAGAGGAAGATACTTGA

TTATACCAGTCTTTGTGGATGAAAATATCTAGCAGTATTCATAGCAAATGCAGTAGGAAG

GAGAGAGTTAATCACAAACAGAAAGTAAGCAGAGAGTGGGACCAAGAGTGGGGATGGGAG

TTCAGCGAGTCACTCACTAGAGTGGCCAGCTCTCCGCCAGCTGATCACACCAAGAGAGAA

GATGATGAGGCCCAGGCCCAGAGTCACTGCAGACACAGAAACCTTCAGGGTCTGCATGGG

GGACAGCCCAGGTGCTGCAAAAAATAGAAACTTACTTGACCCAGTTTCTGTTGCTCACCC

CCAGGGCAATTCCATTTATTGCAGCCACCTCTCAGTGGGTTAAAAGGTCCTTTATCCCAG

CTCCAAGGGTCTAGCTCACACCACCCACTCCCAAGAAAATGATCTTTCTCAAATCAAACC

CTCGTCCCATGGACCTCTACTCCTAGAGTAAGCCTGGGGAATCCATCTCCCCAGAATTAG

CATCCTGGCTTCCAGGTCCTCTCTAATACAGTGGGGCCTCTCAAGGCATCCTCTTTCCTT

CCTTTACCTCAAAGCCACCCTTATCAGGATAAAGGGCTCCTCACTGTCCTCTCCATTGCC

CCCACGGTAACAATGTTTGCTTCCTTACTTTCTCCAACTGAGCAGCTTCCTATTACACTG

TCTTACCACATGTCTTAACCTCCAGTGGATCCATCCTGTGAGTTATCCTACTACTTGTGT

ACCTTCTACATCTAGATCTCCCATGTGTCCTTTCAGAGCTTGTCTCCATCCCACTCCACA

GCCCCTGCACTTCCTTGGGCCGGTCCTGTTCTGAATCATGTCCCACTCAGATTCTTTTCC

CATGATAAAATGAACACTCCATTTCTAAAGGGAGGCTCTTGTGCACGCTGTGAGGAGACG

TTCCCCAGGAAAGTTCAAGTGAGCATGTGATTTCCACTCTCTTCTCTGTTCTCCATTCCC

TTCCCAACTGCCCAGCAAGAAACAACACTTCCCACAAGGGGAAACCTGGTTACAGCAGCT

GATCTGAGATCCTGTTCTCTGGCCCTTTGTAGACACCCTTCCTCTTCCTCATTTCTTCCT

CTTTCTTTTCCAAGAGTCCCCAAAGCTGTGTGCAACTTCTCACGATACCTTTAACTACTC

CCGACACTGAGTTCAAACAGTGTTTGAACTGTAAGTAATTCTTTATCCACTGGCCCCTGA

GCATGCATGCCAAATGGTCTGCCAGCCGTGGCTTTACTACTCCCGTATGCTTGGTAGAGC

AGGCCAAATGCAGTACTGCCCCACACCAAGAAAAGCCCCCCTTCTTCAACCTTCATCATT

CCTTCAGCTCCCATCTGCTTCTGGCACCAGAATAGTTGAAATCTAAGGAGGCTAGAATAG

TGTATTACAATTTGGGGTTCTGAAAATATGATTGCCAAATTTACATCCTCATTTCAAAGC

AAGCACGCTCCCCTCTCACCCTCAAACATAGACGCAGCAACATCAACCACACCACCAGAG

CAGCAATAGCACAGACTAAATATTAAACTGGTGCAAAAGTAATTGCGGTTTTTGCCACTG

AAAGTAATGGCAAAAACTGCAATTACTTTTGCACCAACCTAAATATTTCCATTTCTTTAT

CCCATTTCCCCATTCTGGTCCTAAGCCCCCCGTAAGTTCCTCCAGACTCAGTCCCCATTT

TCAGCACTTCGCTGTCTACCATGTACCATGTATCGATCCACATCTCATTTTCTCTGCTTT

GACCCTAATTCCATCCATCTGCCATACACTTACTCCAGTCCCGAAGGATGGGCTCAGGAG

CCCCAATGTGCTCTACCACACAGGTGTAAGTGTCCCCGTAAGAGGGGGTTAAGGCTAAAT

GGGAGAGGGTCTGGTATGTCCAGTCTCCATTGGGCTGGGCAGTCTTGTGCTCACTGCTGT

GAGGCATGACAAGCTTCCCGTTCTTCCTCCACGTGATAGTCACTTCTGCTGGATAGAAGC

CCCACACATAGCAGGCCAGCATCACAGGCTCCCTCGTGTTAAAAGGAGTGGTTTTGGCTA

CTTGCACAGATGGTGGCCCTGCATAGGAGAAAAAAACATGTTTAGGAAGGAGGGTGACAT

TCTGGCTGCTTCCTCAACCTGGTTTCTTCCCTATCGCAACTCTTCGTAGATTTTGCAACC

CACTTTCCACCCCAGCCCCCTCTGCCATGCTGCCCCTTGAAGGGGAACCGTTAGAATGTA

TTCCTGCATTACTCTTTCTTCTCTCCCATTCCTTCATTGCCCCTTTCTTTCTTTCCTCCT

CCAGAATTATGTTTGATTACAATTAGTAAAAGCCAGATCTGAACTGCAAGCTGTTCTAGA

AGTTGTTGTATTTATTTCAAGTACATAAACTGGAAAGTATTTGAAATAAGGAAGCTAAGA

GTAATCCAGAGTTGTACATTGGGTTTTTTTAAGGTGGAAAAGGAATTTTTCTCCAAATCT

TGTTTAATACGTTCTTTTGCTAGTTAAAGCTTTTTCTCCTCACATAGTTCAAGGAAACAA

GCCTAACTTAGGACTCACTCTTAAATTTGGAATGAATGTAGTCAAACTAAAGAGATTGCT

AATACTGCCATCTTTTACTAATTTACTCTCCTAGGTGATCCTCTTGCTTGCCTCTATCTT

GACATTTTTCAAACACAATCTTAAATAAAAATCCAAGGAATTATGTTAAAATGCAGATTT

CCTAGGCTGTATCCCCAGATACTTTCTTTCAACAGATCTGGAGTGGTACTAAGGGGCTTG

CATCTTTAACAAGCACCTCCTCCAGGCAATTCTGAGAAAGGTGGTTCAGAAACCACCCTT

GAGACACACTGTTCTGTACTGTGGAGATCTTCAAGTTTACTTTCACAAACTTCAAGCCAT

TGTCAATGCAAGAGTTTAAGGGTGAGAAAAAGCATGTGTCAGAATCCCCTGGGATTCCAA

ATATTCCCATGCCTGGGCCCACATCAGATCTGGAACATCAAAATCTGGGATAACAAGGCA

AGAACATCTTGGGTATGCATCCTGAGATGCCCCAGCCTCTGCATAAGCTCCCCACATGGC

ACCTCGCGGTTCAAGCCTCACCTCCCCTTCTTTACTCCTGTTCCACTCACGTCAGCCACC

TTGTTCCCCTTGAGGTTCAATCCTCCGTCTTTCTACATTTCAGATCCACACATTTTCTCT

TATTTGCTGCTCAAATCTCAAACCCCTGGGCCACTGTGGGATCCTCCCTGGCCTGCCCTC

CTAACTGCACTTCCTGGTAGCCCCTCTGCACCCCTCTCTCCTCACGTGTCCTGTTGGTCA

GTGATCCCCAGAAGGGCTGGGTGTGTGTGGCACAATTCTGAAGCCCATTGCGCAAGCGCT

GCATCAGGGTGTCTTTTTGGTTGAGGTGCTGTGAGAGGACATTCGCCAAGCTATTCAGCA

CCCCAAATTCGCAAGGGGCCATCTTATTCTCCTCTGGATCCCAGCAGGTCAGCAGATCCT

TGTTGAAGGAGATGCAGTATGTGAAATCCTTTGGAGTCCCAGCATCATCCAACAGACAGG

TGCTTTCCACATGGGCCACGAAGCCACCTAGAGGAGCCAGGGAAGGGAGAACAGGTCAAT

GTCTTCTACTGGCCTGGCAATAAATAAATAAATATATAAATAATAAATATACACAAATAA

TAAATATATAAAACATACAGACGTATATTTAGGAGCTCTGCACAGAGCTTTGTCTTTGAC

CCTGGTTCCTGACATAGAGTGCCTAATCGCTTAGAATTTCCTAGATAACAGGAGTGTCTT

TTGTTCTAATGAGGTACTCTTGGTGGGCTCCTGCAGGAGGGGCTGGTCACCAGAAAGACC

AAGTCATGATTAGAAGTCTGGAACTTTTAGTCCCATCCCCCATACTCCCTGAAGGGGAAG

GGGCTGGAGATTGAGTTAATAATCAGTCATGCCTACATGATGAAGCCTCCATAAAAATCC

CGGAACTATGGAGTTCAGAGAACTTCTCAGTTGGTAAACACATCCACATGCCAGGAGGTG

AAGTACCCCAATTCTGTGGGGACAGAGCTCCTGTGATTGGGACCCTTCCAGATCTGGTAT

CTCTTCATCTGGATGTTCCTTTGTATGCTTTAAAATATCCTTTGTTAAAGGATGCAAAAT

TATGATCTAGTGTTCTATACCACTGTGGGATGACTGTCATTAACAATAATACTTTATATC

ATTTCAAATCGCTAGAAGAAGGATATGGAATGTTTCCAACACAAAGAAATGATAAATGAG

ATGATGATCTGATCTGATCACTGTACATTACATGTACTAAAACATCATTATCCACCCCAT

GAATATTTATAATTATTATTATCAATTAAAATATCCTTTGTAAAAAATCTACAATAATAA

GTAAACTTTTCCTGAGTTCCATAAGCCACTTTAGCAAATTACCAACCCCAAGGAGGGGGT

CATGGGAACCTCTGATTTGTAGGCAAGTTGGACAGAAGATGTGGGTAATTTGGGAACCTA

CTACTTGTGATTGGTGTCTGAAATGGAGGCAGTCTTATGGGACTGAGTCTTTAACCTTTG

GTGTCTATGTTAACTCTAGTTAATGTCACAATGGAATTGAATTATAGGATATCCAGCTAA

TATAGGAGAATTGGTTGGTATGAGTAAAAAAAAAAAAAAAACCTCACACAGTTGGTCAAA

GAAGTGTTGAGTGTGAGCATATAGAAGAAAAAAAGTTGATTTTTCCTATATTCAGCTCAG

AACCTAAGCCTTGGTGACATCCAGCTAGTCTGGCACAGATTTCCTGCTCAGGGAACATCT

ACTGACCAAGCTCATACACTGAAGTTTCTGAAAGTCTGATTTGAGGGAGTCAGTAGAAGT

AGTAGATAAGTTTTTAGATCCAGTCTCCTCTTTATGCAAGACTAAGCACAGGGATAGGAG

TAGCCCCCCGAGATTATTTGCATGTTTAAACATGACAATTTGCCCAGAACACAGACCTTC

AGTAAGGCAAATTATTGAGAGAGAAAAAGGGTCAAGAGAAAGAGTCAGCCTTGTATTGTG

CTGGAAATATTAAATATTCACTTCGCACATATTTATGAAGCACTTGCTGCATGCAAGGCA

CTGTGCTAGGAGCTGAGGAGGCAGCAATAAATAAGATGAACATTGTCCTTGCCTATATTC

CAGCAGGGAATATACACTGCACAGATAATTATACAGATTAATTACATTAAAATTGCTACA

AAGTACAAAGTGCTATAGGAATGTATACCAGGGAGACAAACTATCTGGGGTGTCAAATGC

AATTACAAAACGGAACACCCTTACCCTGAAACAGGAGCAGGGGAAGGGAGAGTCCCCAGA

AGAAGTGTCCTTACCTGCTCCTGTGCAGCCCAGGCTGAGCCCCAGCAGCAGCGGCAGGAA

TGTGATCATGCTCTGCTCTGTAAAGATGCCGGGAGTTCAGTCCCCTGGACCAGCTCTTCC

AGGGTCCGTGGGTCCTCGCCTGTCCCAGAAGCCCCAGCCTGGGTAGATGATCTCCAGACA

CTGAGCAGAATACTATATTGCCCGGGTCCCTTGACCCCCCAAATGAGTGATGTGGGGATA

CCCAGCCCCTAGATATTAAATCTGTTCCTTCCAGCTCACGGGAGTCCAGTGTCCCAAACA

GGGACAGATTGGCTAGGTAGGCAGGGACAAATGTAGAGACAAATCACTGAGTGCCTCAGC

CTAGCATCATCAGTTACTAG

>HLA:HLA00491 DMB*01:03:01:01

CTAGTAACTGATGATGCTAGGCTGAGGCACTCAGTGATTTGTCTCTACATTTGTCCCTGC

CTACCTAGCCAATCTGTCCCTGTTTGGGACACTGGACTCCCGTGAGCTGGAAGGAACAGA

TTTAATATCTAGGGGCTGGGTATCCCCACATCACTCATTTGGGGGGTCAAGGGACCCGGG

CAATATAGTATTCTGCTCAGTGTCTGGAGATCATCTACCCAGGCTGGGGCTTCTGGGACA

GGCGAGGACCCACGGACCCTGGAAGAGCTGGTCCAGGGGACTGAACTCCCGGCATCTTTA

CAGAGCAGAGCATGATCACATTCCTGCCGCTGCTGCTGGGGCTCAGCCTGGGCTGCACAG

GAGCAGGTAAGGACACTTCTTCTGGGGACTCTCCCTTCCCCTGCTCCTGTTTCAGGGTAA

GGGTGTTCCGTTTTGTAATTGCATTTGACACCCCAGATAGTTTGTCTCCCTGGTATACAT

TCCTATAGCACTTTGTACTTTGTAGCAATTTTAATGTAATTAATCTGTATAATTATCTGT

GCAGTGTATATTCCCTGCTGGAATATAGGCAAGGACAATGTTCATCTTATTTATTGCTGC

CTCCTCAGCTCCTAGCACAGTGCCTTGCATGCAGCAAGTGCTTCATAAATATGTGCGAAG

TGAATATTTAATATTTCCAGCACAATACAAGGCTGACTCTTTCTCTTGACCCTTTTTCTC

TCTCAATAATTTGCCTTACTGAAGGTCTGTGTTCTGGGCAAATTGTCATGTTTAAACATG

CAAATAATCTCGGGGGGCTACTCCTATCCCTGTGCTTAGTCTTGCATAAAGAGGAGACTG

GATCTAAAAACTTATCTACTACTTCTACTGACTCCCTCAAATCAGACTTTCAGAAACTTC

AGTGTATGAGCTTGGTCAGTAGATGTTCCCTGAGCAGGAAATCTGTGCCAGACTAGCTGG

ATGTCACCAAGGCTTAGGTTCTGAGCTGAATATAGGAAAAATCAACTTTTTTTCTTCTAT

ATGCTCACACTCAACACTTCTTTGACCAACTGTGTGAGGTTTTTTTTTTTTTTTTACTCA

TACCAACCAATTCTCCTATATTAGCTGGATATCCTATAATTCAATTCCATTGTGACATTA

ACTAGAGTTAACATAGACACCAAAGGTTAAAGACTCAGTCCCATAAGACTGCCTCCATTT

CAGACACCAATCACAAGTAGTAGGTTCCCAAATTACCCACATCTTCTGTCCAACTTGCCT

ACAAATCAGAGGTTCCCATGACCCCCTCCTTGGGGTTGGTAATTTGCTAAAGTGGCTTAT

GGAACTCAGGAAAAGTTTACTTATTATTGTAGATTTTTTACAAAGGATATTTTAATTGAT

AATAATAATTATAAATATTCATGGGGTGGATAATGATGTTTTAGTACATGTAATGTACAG

TGATCAGATCAGATCATCATCTCATTTATCATTTCTTTGTGTTGGAAACATTCCATATCC

TTCTTCTAGCGATTTGAAATGATATAAAGTATTATTGTTAATGACAGTCATCCCACAGTG

GTATAGAACACTAGATCATAATTTTGCATCCTTTAACAAAGGATATTTTAAAGCATACAA

AGGAACATCCAGATGAAGAGATACCAGATCTGGAAGGGTCCCAATCACAGGAGCTCTGTC

CCCACAGAATTGGGGTACTTCACCTCCTGGCATGTGGATGTGTTTACCAACTGAGAAGTT

CTCTGAACTCCATAGTTCCGGGATTTTTATGGAGGCTTCATCATGTAGGCATGACTGATT

ATTAACTCAATCTCCAGCCCCTTCCCCTTCAGGGAGTATGGGGGATGGGACTAAAAGTTC

CAGACTTCTAATCATGACTTGGTCTTTCTGGTGACCAGCCCCTCCTGCAGGAGCCCACCA

AGAGTACCTCATTAGAACAAAAGACACTCCTGTTATCTAGGAAATTCTAAGCGATTAGGC

ACTCTATGTCAGGAACCAGGGTCAAAGACAAAGCTCTGTGCAGAGCTCCTAAATATACGT

CTGTATGTTTTATATATTTATTATTTGTGTATATTTATTATTTATATATTTATTTATTTA

TTGCCAGGCCAGTAGAAGACATTGACCTGTTCTCCCTTCCCTGGCTCCTCTAGGTGGCTT

CGTGGCCCATGTGGAAAGCACCTGTCTGTTGGATGATGCTGGGACTCCAAAGGATTTCAC

ATACTGCATCTCCTTCAACAAGGATCTGCTGACCTGCTGGGATCCAGAGGAGAATAAGAT

GGCCCCTTGCGAATTTGGGGTGCTGAATAGCTTGGCGAATGTCCTCTCACAGCACCTCAA

CCAAAAAGACACCCTGATGCAGCGCTTGCGCAATGGGCTTCAGAATTGTGCCACACACAC

CCAGCCCTTCTGGGGATCACTGACCAACAGGACACGTGAGGAGAGAGGGGTGCAGAGGGG

CTACCAGGAAGTGCAGTTAGGAGGGCAGGCCAGGGAGGATCCCACAGTGGCCCAGGGGTT

TGAGATTTGAGCAGCAAATAAGAGAAAATGTGTGGATCTGAAATGTAGAAAGACGGAGGA

TTGAACCTCAAGGGGAACAAGGTGGCTGACGTGAGTGGAACAGGAGTAAAGAAGGGGAGG

TGAGGCTTGAACCGCGAGGTGCCATGTGGGGAGCTTATGCAGAGGCTGGGGCATCTCAGG

ATGCATACCCAAGATGTTCTTGCCTTGTTATCCCAGATTTTGATGTTCCAGATCTGATGT

GGGCCCAGGCATGGGAATATTTGGAATCCCAGGGGATTCTGACACATGCTTTTTCTCACC

CTTAAACTCTTGCATTGACAATGGCTTGAAGTTTGTGAAAGTAAACTTGAAGATCTCCAC

AGTACAGAACAGTGTGTCTCAAGGGTGGTTTCTGAACCACCTTTCTCAGAATTGCCTGGA

GGAGGTGCTTGTTAAAGATGCAAGCCCCTTAGTACCACTCCAGATCTGTTGAAAGAAAGT

ATCTGGGGATACAGCCTAGGAAATCTGCATTTTAACATAATTCCTTGGATTTTTATTTAA

GATTGTGTTTGAAAAATGTCAAGATAGAGGCAAGCAAGAGGATCACCTAGGAGAGTAAAT

TAGTAAAAGATGGCAGTATTAGCAATCTCATTAGTTTGACTACATTCATTCCAAATTTAA

GAGTGAGTCCTAAGTTAGGCTTGTTTCCTTGAACTATGTGAGGAGAAAAAGCTTTAACTA

GCAAAAGAACGTATTAAACAAGATTTGGAGAAAAATTCCTTTTCCACCTTAAAAAAACCC

AATGTACAACTCTGGATTACTCTTAGCTTCCTTATTTCAAATACTTTCCAGTTTATGTAC

TTGAAATAAATACAACAACTTCTAGAACAGCTTGCAGTTCAGATCTGGCTTTTACTAATT

GTAATCAAACATAATTCTGGAGGAGGAAAGAAAGAAAGGGGCAATGAAGGAATGGGAGAG

AAGAAAGAGTAATGCAGGAATACATTCTAACGGTTCCCCTTCAAGGGGCAGCATGGCAGA

GGGGGCTGGGGTGGAAAGTGGGTTGCAAAATCTACGAAGAGTTGCGATAGGGAAGAAACC

AGGTTGAGGAAGCAGCCAGAATGTCACCCTCCTTCCTAAACATGTTTTTTTCTCCTATGC

AGGGCCACCATCTGTGCAAGTAGCCAAAACCACTCCTTTTAACACGAGGGAGCCTGTGAT

GCTGGCCTGCTATGTGTGGGGCTTCTATCCAGCAGAAGTGACTATCACGTGGAGGAAGAA

CGGGAAGCTTGTCATGCCTCACAGCAGTGCGCACAAGACTGCCCAGCCCAATGGAGACTG

GACATACCAGACCCTCTCCCATTTAGCCTTAACCCCCTCTTACGGGGACACTTACACCTG

TGTGGTAGAGCACACTGGGGCTCCTGAGCCCATCCTTCGGGACTGGAGTAAGTGTATGGC

AGATGGATGGAATTAGGGTCAAAGCAGAGAAAATGAGATGTGGATCGATACATGGTACAT

GGTAGACAGCGAAGTGCTGAAAATGGGGACTGAGTCTGGAGGAACTTACGGGGGGCTTAG

GACCAGAATGGGGAAATGGGATAAAGAAATGGAAATATTTAGGTTGGTGCAAAAGTAATT

GCAGTTTTTGCCATTACTTTCAGTGGCAAAAACCGCAATTACTTTTGCACCAGTTTAATA

TTTAGTCTGTGCTATTGCTGCTCTGGTGGTGTGGCTGATGTTGCTGCGTCTATGTTTGAG

GGTGAGAGGGGAGCGTGCTTGCTTTGAAATGAGGCTGTAAATTTGGCAATCATATTTTCA

GAACCCCAAATTGTAATACACTATTCTAGCCTCCTTAGATTTCAACTATTCTGGTGCCAG

AAGCAGATGGGAGCTGAAGGAATGATGAAGGTTGAAGAAGGGGGGCTTTTCTTGGTGTGG

GGCAGTACTGCATTTGGCCTGCTCTACCAAGCATACGGGAGTAGTAAAGCCACGGCTGGC

AGACCATTTGGCATGCATGCTCAGGGGCCAGTGGATAAAGAATTACTTACAGTTCAAACA

CTGTTTGAACTCAGTGTCGGGAGTAGTTAAAGGTATCGTGAGAAGTTGCACACAGCTTTG

GGGACTCTTGGAAAAGAAAGAGGAAGAAATGAGGAAGAGGAAGGGTGTCTACAAAGGGCC

AGAGAACAGGATCTCAGATCAGCTGCTGTAACCAGGTTTCCCCTTGTGGGAAGTGTTGTT

TCTTGCTGGGCAGTTGGGAAGGGAATGGAGAACAGAGAAGAGAGTGGAAATCACATGCTC

ACTTGAACTTTCCTGGGGAACGTCTCCTCACAGCGTGCACAAGAGCCTCCCTTTAGAAAT

GGAGTGTTCATTTTATCATGGGAAAAGAATCTGAGTGGGACATGATTCAGAACAGGACCG

GCCCAAGGAAGTGCAGGGGCTGTGGAGTGGGATGGAGACAAGCTCTGAAAGGACACATGG

GAGATCTAGATGTAGAAGGTACACAAGTAGTAGGATAACTCACAGGATGGATCCACTGGA

GGTTAAGACATGTGGTAAGACAGTGTAATAGGAAGCTGCTCAGTTGGAGAAAGTAAGGAA

GCAAACATTGTTACCGTGGGGGCAATGGAGAGGACAGTGAGGAGCCCTTTATCCTGATAA

GGGTGGCTTTGAGGTAAAGGAAGGAAAGAGGATGCCTTGAGAGGCCCCACTGTATTAGAG

AGGACCTGGAAGCCAGGATGCTAATTCTGGGGAGATGGATTCCCCAGGCTTACTCTAGGA

GTAGAGGTCCATGGGACGAGGGTTTGATTTGAGAAAGATCATTTTCTTGGGAGTGGGTGG

TGTGAGCTAGACCCTTGGAGCTGGGATAAAGGACCTTTTAACCCACTGAGAGGTGGCTGC

AATAAATGGAATTGCCCTGGGGGTGAGCAACAGAAACTGGGTCAAGTAAGTTTCTATTTT

TTGCAGCACCTGGGCTGTCCCCCATGCAGACCCTGAAGGTTTCTGTGTCTGCAGTGACTC

TGGGCCTGGGCCTCATCATCTTCTCTCTTGGTGTGATCAGCTGGCGGAGAGCTGGCCACT

CTAGTGAGTGACTCGCTGAACTCCCATCCCCACTCTTGGTCCCACTCTCTGCTTACTTTC

TGTTTGTGATTAACTCTCTCCTTCCTACTGCATTTGCTATGAATACTGCTAGATATTTTC

ATCCACAAAGACTGGTATAATCAAGTATCTTCCTCTCTTAGGTTACACTCCTCTTCCTGG

GTCCAATTATTCAGAAGGTAACATCTCTGTTGGTCTGTTTCCCTACTTGCCCTTTGGTAG

GGGTGCGGGTTAGAGGGGTCAGTGTTGGGTTCAACTAATCTTGATTATTATATGGGTGAG

CTTCCATGAGGATCTAGGCAAGGGCATGATTTAAGCTGCCATTGCTAGGATTAAGAGCAG

GAAGGAGCATCCTCCTCTTCTACCAAGTGGGATGTCTGTGGAGAGGAGGCTGAAGGTGCT

TCCTTTGTATTAGTTGTTGGTGCCCTGGAGTTTTCAGTATCACTGTATTAAGGCATGGGA

TGGTTACAGTGACAAACGATGGGGGCAAGTTGGGTTGAAGCCTCATTATCTCCCTTTTAT

TTATTCTGTAGGATGGCACATTTCCTAGAGGCAGAATCCTACAACTTCCACTCCAAGTGA

GAAGGAGATTCAAACTCAATGATGCTACCATGCCTCTCCAACATCTTCAACCCCCTGACA

TTATCTTGGATCCTATGGTTTCTCCATCCAATTCTTTGAATTTCCCAGTCTCCCCTATGT

AAAACTTAGCAACTTGGGGGACCTCATTCCTGGGACTATGCTGTAACCAAATTATTGTCC

AAGGCTATATTTCTGGGATGAATATAATCTGAGGAAGGGAGTTAAAGACCCTCCTGGGGC

TCTCAGTGTGCCATAGAGGACAGCAACTGGTGATTGTTTCAGAGAAATAAACTTTGGTGG

AAATATTGTTTTTCCATGTCTTCTTCCTGGGGCCCTGGGGAAGGAATATGGGCAAAGCAG

GGACTGAGGTTAATTCTCTTCTGCTTGAGTAGGGGAGAAATCAATGCCTTCTTCCATTTT

CCCACTTAGACATGACAGAATTTGGGGCCGTTTTCTGATTTATAATTCATAAGGAGAAAT

TCAACTGTGGTGGGTTGGAGTCACAGAGTATGGGCAAGGAAGGGAATTAACAGCTTACTC

ACCTCATACAGGATCTTATG

>HLA:HLA00494 DOA*01:01:01:01

AGCTGCGCGGTGCTCTGACGGCCTTTTCTCTCTCTTCAGGGGGTCCCTTCTTGGAGGACG

TGGTAATAGTTGGTCCAAGCCCCTCCCATCCTCCAAAACTTCCTTTGGCCTCTCCTGGGG

GCCCAGTGAATCCTTCCACCTTCTCACCCCGGCTCTTTCCCTCTTTACCCAGCAACAGAT

ACATTCACTCAGAGAATTTCTGTGATTGGCTGAAGACAGAAGGGGTCGCCCCCATCCTCG

AATCTGTTTTCTTCTTCTTTACCTCCGCCTTGTTCCTGTCCTCACCACACGGACTGAGAC

TGATTTGATTAAAGCACCAGAGTGTAATGGCCCTCAGAGCAGGGCTGGTCCTGGGGTTCC

ACACCCTGATGACCCTCCTGAGCCCGCAGGAGGCAGGGGCCACCAAGGGTGAGTGCGAGG

GCGAGGAGGGTGCGGCGGGGAGCAGAGATTTACGGAGTTGGGTTACATGAGGAGGTGGCA

TGGAGGATGCTTGTTCCTCTCGCTCTCTGGTTTATGGGCAACTTCCTTCACCAAGAGACA

CCCAATCCCCCTCATCTCTGTCACATCCACTCTGGACCTAAATGAAGATGCAGCTCGGTC

AGCTGCGCAGGTGCCCCAGTCAGCCTTTGCTGACGTTCAGATTTCTCCTCATTCCTTCCT

CCTTCCTGAGACCCAAACCTCCACCCAACAGATGCCAGCAAGCACCCTGATTTCTCTACC

ACCCCTGGCCTGGAATGTGCCCGATCAAGTCCAGTTCTGTTGCAGTATTTATGCCCATGC

CGGTAGTTAACTATTTACCTGTCTTTGTTCTTCGGGAGACATGAGCTGGGGTGCGGGTCT

ACAGATGGTTCATCTTTTTTTTCTTTTATTTCCCTGGCCCCCCATTGTGCTGGGTGCATG

CTAGTTCCTCAATAACTGTTGCTCAAACAACTTCATAGAGTTCTACAAGAATTAAAACTT

AATCCCTAACTTCCAGAAAACTAGACAACAGTTATGGAAGAGCCACACTCAGTCATAATG

CTCTGAGATGGAGGAATTGGGACATGAACCTTGACTTCTGACTCCTCGTCCAGTGCTCTT

TGTAATGCCTTGAGTTGCCTCTCCCTATCCCCTTGGTCTCTGGGTCACTAACCTTAATTC

TTACCCCTGCCAGCCGTGGCCTGTTTCCCCTCACACCCACCTGCACTGCATCTCTGTGCA

AAGCTCCATACTCTCCTGTCCTTAATCATTCCTCCTCATCCCACCCCCACAGTCCCACCA

GCTCACAACACAGGGCCTGACAACACAGCCAGGGCAGATGACCACAGCCAAGATTTAAAT

TCTGGAACCCCAAGCATGATTTTAGGCAGTCCCTCCTCTTCCCATCCTGATATGCCAGAC

TGCACTGTCTCTGTGCCGACTGCAGTGTGCTGGGATGAGCCTCTTTCTTCCTGTTCTCTC

TCCCTTTCTCTCTCCCAGGGCCAGCCCAGTGTCAGAACAGGACTCTGTCCCCACACAGAA

CCCAGGACGGGGCCCAGGCTCAGGGACTCAACAATCACATATTGTGGATGAGACAGACAC

ATTTTTTTCTCTCTCCTTGACCCTGAACTCGCCAAACACAGCTGACCACATGGGCTCCTA

CGGACCCGCCTTCTACCAGTCTTACGGCGCCTCGGGCCAGTTCACCCATGAATTTGATGA

GGAACAGCTGTTCTCTGTGGACCTGAAGAAAAGCGAGGCCGTGTGGCGTCTGCCTGAGTT

TGGTGACTTTGCCCGCTTTGACCCGCAGGGTGGGCTGGCCGGCATCGCCGCAATCAAAGC

CCATCTGGACATCCTGGTGGAGCGCTCCAACCGCAGCAGAGCCATCAACGGTACCGGCCC

TCCCTCTGCCCACCCAGTCAGGCGGGAAGGTCCAGAGAAACTTCCTCCCAGTTCCTAGGT

TCCCATCACTCTGGGGCGCGCTCTCAGCGCCCGCGCCTGTCATGCCCTGTTCCTTTCTTT

CCCAGGAGGCTCCAGGTCTTCCCAGACCCCTTTGGCACCCCTCTCCTTGAGGAATGACAC

CTCTCACCCGGACTCCCGCCCAGGGACCAGTCAAATATAGGAGCTCCTGGCGTCCCCACT

CCCTCCCCAGTCTCCTCTCCCTCTGTTTCCCTCCTCTCCTGCCCCAGTGGATACCCCAGA

GCATCCCCTGCCCACAGATGGCTACAAAGGGGGAACGTCCCTTAATCCCAGTCCTAGTAA

GGCCCTGGGGTGAGGGATGAGCCTGTGGACTCAGGGCCCGTTCCTCCTAGTGCCTCCACG

GGTGACCGTGCTCCCCAAGTCTCGGGTGGAGCTGGGCCAGCCCAACATCCTCATCTGCAT

CGTGGACAACATCTTCCCCCCTGTGATCAATATCACCTGGCTACGCAACGGCCAAACTGT

CACTGAGGGAGTGGCCCAGACCAGCTTCTATTCCCAGCCTGACCATTTGTTCCGCAAGTT

CCACTACCTGCCCTTCGTGCCCTCAGCCGAGGACGTCTATGACTGCCAGGTGGAGCACTG

GGGCCTGGATGCGCCACTCCTCAGGCATTGGGGTACGGAGCCCCCTCCCCATGCACCCTC

CTGGCCCCAGGTTTCCTTTACTCTAGAATCCTTTCATATACCACCGACTCCTTCCTTTCT

CTCCTAGAGCTCCAGGTGCCTATTCCACCACCAGATGCCATGGAGACCCTGGTCTGTGCC

CTGGGTCTGGCCATCGGCCTGGTGGGCTTCCTCGTGGGCACCGTCCTCATCATCATGGGC

ACATATGTGTCCAGTGTCCCCAGGTGCAGAGGCCCCGGGAGTCTGGGGGGTGGGGGAGGA

AAGTGGATGACTCTGAACAGGACGTGGGTGGAGAATCAGAGATTCTGTTGTGGGGAAAGA

AGTCAGAAAAGAAATGGGCAGGGAGAAAAGAAGCAGAGGTGGGGTGAGAGAGTGAGGTTT

TGGGGGAGGTGGGCACTCAGAGATAGGATCCCAGCATATTGAAATTGAGCAACCTCGATC

GTATGTTTTCTGCTATTTTAGGTAATGATCCTTCTGAGAGAAATGACTTGTGGGAGACAC

CCTGCAGATCCTCATGGGTTTGTGACAGCCCCTGCGTGCTCAGTGCCCTTTAAGTGCATC

CCGCTGTGCTGACTTTGAGTGGGATCAACATCTGTCCTACGGGTCCCCTCTTTTTTGGCC

CCAGTATTCATGGCAGGGTTTGTTGGACACCTACTAGCTTCCCTTCCCATTCAACACAAA

CACACATTCTTGCTCTACCCAAAGCTCTGGCTGGCAGCACTAAATGCTTTGGTGGTGTTT

GCACTGTGTCCTTTCCAGGCCTTGGCCAGTTCTTCCAGGGGTGAGGCATGTGGTGCTGGG

GATTGGCAGCCGTCCTGGGGCCCACACAGGTGTGTCTTGCTCCATTTGGCCCATTGTGTG

TTACTTTGTGAATGAGCCATTTCACATGGACTTCATGAAATTTGCCTCCTGAGTTCAGGT

TTACCCTGAAAGGGATGCAGATTATCCTGTTCCTCACGACCCCCTCAGCTAACAACAGTT

CTGAAGGGTGCTGGGACAAGACAGGCTCATGGGGACTCCACTCCTGCCTGGGTTTACTCT

GTATGAAGAGGCCACTGGTATCCTGCCATGATGTTATCTCCTTTTTCTACTTT

>HLA:HLA00995 DOA*01:01:02:03

AGCTGCGCGGTGCTCTGACGGCCTTTTCTCTCTCTTCAGGGGGTCCCTTCTTGGAGGACG

TGGTAATAGTTGGTCCAAGCCCCTCCCATCCTCCAAAACTTCCTCTGGCCTCTCCTGGGG

GCCCAGTGAATCCTTCCACCTTCTCACCCCGGCTCTTTCCCTCTTTACCCAGCAACAGAT

ACATTCACTCAGAGAATTTCTGTGATTGGCTGAAGACAGCAGGGGTCGCCCCCATCCTCG

AATCTGTTTTCTTCTTCTTTACCTCCGCCTTGTTCCTGTCCTCACCACACGGACTGAGAC

TGATTTGATTAAAGCACCAGAGTGTAATGGCCCTCAGAGCAGGGCTGGTCCTGGGGTTCC

ACACCCTGATGACCCTCCTGAGCCCGCAGGAGGCAGGGGCCACCAAGGGTGAGTGCGAGG

GCGAGGAGGGTGCGGCGGGGAGCAGAGATTTACGGAGTTGGGTTACATGAGGAGGTGGCA

TGGAGGATGCTTGTTCCTCTCGCTCTCTGGTTTATGGGCAACTTCCTTCACCAAGAGACA

CCCAATCCCCCTCATCTCTGTCACATCCACTCTGGACCTAAATGAAGATGCAGCTCGGTC

AGCTGCGCAGGTGCCCCAGTCAGCCTTTGCTGACGTTCAGATTTCTCCTCATTCCTTCCT

CCTTCCTGAGACCCAAACCTCCACCCAACAGATGCCAGCAAGCACCCTGATTTCTCTACC

ACCCCTGGCCGGGAATGTGCCCGATCAAGTCCAGTTCTGTTGCAGTATTTATGCCCATGC

CGGTAGTTAACTATTTACCTGTCTTTGTTCTTCGGGAGACATGAGCTGGGGTGCGGGTCT

ACAGATGGTTCATCTTTTTTTTCTTTTATTTCCCTGGCCCACCATTGTGCTGGGTGCATG

CTAGTTCCTCAATAACTGTTGCTCAAACAACTTCATAGAGTTCTACAAGAATTAAAACTT

AATCCCTAACTTCCAGAAAACTAGACAACAGTTATGGAAGAGCCACACTCAGTCATAATG

CTCTGAGATGGAGGAATTGGGACATGAACCTTGACTTCTGACTCCTCGTCCAGTGCTCTT

TGTAATGCCTTGAGTTGCCTCTCCCTATCCCCTTGGTCTCTGGGTCACTAACCTTAATTC

TTACCCCTGCCAGCCGTGGCCTGTTTCCCCTCACACCCACCTGCACTGCATCTCTGTGCA

AAGCTCCATACTCTCCTGTCCTTAATCATTCCTCCTCATCCCACCCCCACAGTCCCACCA

GCTCACAACACAGGGCCTGACAACACAGCCAGGGCAGATGACCACAGCCAAGATTTAAAT

TCTGGAACCCCAAGCATGATTTTAGGCAGTCCCTCCTCTTCCCATCCTGATATGCCAGAC

TGCACTGTCTCTGTGCCGACTGCAGTGTGCTGGGATGAGCCTCTTTCTTCCTGTTCTCTC

TCCCTTTCTCTCTCCCAGGGCCAGCCCAGTGTCAGAACAGGACTCTGTCCCCACACAGAA

CCCAGGACGGGGCCCAGGCTCAGGGACTCAACAATCACATATTGTGGATGAGACAGACAC

ATTTTTTTCTCTCTCCTTGACCCTGAACTCGCCAAACACAGCTGACCACATGGGCTCCTA

CGGACCCGCCTTCTACCAGTCTTACGGCGCCTCGGGCCAGTTCACCCATGAATTTGATGA

GGAACAGCTGTTCTCTGTGGACCTGAAGAAAAGCGAGGCCGTGTGGCGTCTGCCTGAGTT

TGGTGACTTTGCCCGCTTTGACCCGCAGGGCGGGCTGGCCGGCATCGCCGCAATCAAAGC

CCATCTGGACATCCTGGTGGAGCGCTCCAACCGCAGCAGAGCCATCAACGGTACCGGCCC

TCCCTCTGCCCACCCAGTCAGGCGGGAAGGTCCAGAGAAACTTCCTCCCAGTTCCTAGGC

TCCCATCACTCTGGGGCGCGCTCTCAGCGCCCGCGCCTGTCATGCCCTGTTCCTTTCTTT

CCCAGGAGGCTCCAGGTCTTCCCAGACCCCTTTGGCACCCCTCTCCTTGAGGAATGACAC

CTCTCACCCGGACTCCCGCCCAGGGACCAGTCAAATATAGGAGCTCCTGGCGTCCCCACT

CCCTCCCCAGTCTCCTCTCCCTCTGTTTCCCTCCTCTCCTGCCCCAGTGGATACCCCAGA

GCATCCCCTGCCCACAGATGGCTACAAAGGGGGAACGTCCCTTAATCCCAGTCCTAGTAA

GGCCCTGGGGTGAGGGATGAGCCTGTGGACTCAGGGCCTGTTCCTCCTAGTGCCTCCACG

GGTGACCGTGCTCCCCAAGTCTCGGGTGGAGCTGGGCCAGCCCAACATCCTCATCTGCAT

CGTGGACAACATCTTCCCCCCTGTGATCAATATCACCTGGCTGCGCAACGGCCAAACTGT

CACTGAGGGAGTGGCCCAGACCAGCTTCTATTCCCAGCCTGACCATTTGTTCCGCAAGTT

CCACTACCTGCCCTTCGTGCCCTCAGCCGAGGACGTCTATGACTGCCAGGTGGAGCACTG

GGGCCTGGATGCGCCACTCCTCAGGCATTGGGGTACGGAGCCCCCTCCCCATGCACCCTC

CTGGCCCCAGGTTTCCTTTACTCTAGAATCCTTTCATATACCACCAACTCCTTCCTTTCT

CTCCTAGAGCTCCAGGTGCCTATTCCACCACCAGATGCCATGGAGACCCTGGTCTGTGCC

CTGGGCCTGGCCATCGGCCTGGTGGGCTTCCTCGTGGGCACCGTCCTCATCATCATGGGC

ACATATGTGTCCAGTGTCCCCAGGTGCAGAGGCCCCGGGAGTCTGGGGGGTGGGGGAGGA

AAGTGGATGACTCTGAACAGGACGTGGGTGGAGAATCAGAGATTCTGTTGTGGGGAAAGA

AGTCAGAAAAGAAATGGGCAGGGAGAAAAGAAGCAGAGGTAGGGTGAGAGAGTGAGGTTT

TGGGGGAGGTGGGCACTCAGAGATAGGATCCCAGCATATTGAAATTGAGCAACCTCGATC

GTATGTTTTCTGCTATTTTAGGTAATGATCCTTCTGAGAGAAATGACTTGTGGGAGACAC

CCTGCAGATCCTCATGGGTTTGTGACAGCCCCTGCGTGCTCAGTGCCCTTTAAGTGCATC

CCGCTGTGCTGACTTTGAGTGGGATCAACATCTGTCCTACGGGTCCCCTCTTTTTTGGCC

CCAGTATTCATGGCAGGGTTTGTTGGACACCTACTAGCTTCCCTTCCCATTCAACACAAA

CACACATTCTTGCTCTACCCAAAGCTCTGGCTGGCAGCACTAAATGCTTTGGTGGTGTTT

GCACTGTGTCCTTTCCAGGCCTTGGCCAGTTCTTCCAGGGGTGAGGCATGTGGTGCTGGG

GATTGGCAGCCATCCTGGGGCCCACACAGGTGTGTCTTGCTCCATTTGGCCCATTGTGTG

TTACTTTGTGAATGAGCCATTTCACATGGACTTCATGAAATTTGCCTCCTGAGTTCAGGT

TTACCCTGAAAGGGATGCAGATTATCCTGTTCCTCACGACCCCCTCAGCTAACAACAGTT

CTGAAGGGTGCTGGGACAGGACAGGCTCATGGGGACTCCACTCCTGCCTGGGTTTACTCT

GTATGAAGAGGCCACTGGTATCCTGCCATGATGTTATCTCCTTTTTCTACTTT

>HLA36099 DOA*01:01:02:45

AAAGTAGAAAAAGGAGATAACATCATGGCAGGATACCAGTGGCCTCTTCATACAGAGTAA

ACCCAGGCAGGAGTGGAGTCCCCATGAGCCTGTCCTGTCCCAGCACCCTTCAGAACTGTT

GTTAGCTGAGGGGGTCGTGAGGAACAGGATAATCTGCATCCCTTTCAGGGTAAACCTGAA

CTCAGGAGGCAAATTTCATGAAGTCCATGTGAAATGGCTCATTCACAAAGTAACACACAA

TGGGCCAAATGGAGCAAGACACACCTGTGTGGGCCCCAGGATGGCTGCCAATCCCCAGCA

CCACATGCCTCACCCCTGGAAGAACTGGCCAAGGCCTGGAAAGGACACAGTGCAAACACC

ACCAAAGCATTTAGTGCTGCCAGCCAGAGCTTTGGGTAGAGCAAGAATGTGTGTGTGTGT

TGAATGGGAAGGGAAGCTAGTAGGTGTCCAACAAACCCTGCCATGAATACTGGGGCCAAA

AAAGAGGGGACCCGTAGGACAGATGTTGATCCCACTCAAAGTCAGCACAGCGGGATGCAC

TTAAAGGGCACTGAGCACGCAGGGTCTGTCACAAACCCATGAGGATCTGCAGGGTGTCTC

CCACAAGTCATTTCTCTCAGAAGGATCATTACCTAAAATAGCAGAAAACATACGATCGAG

GTTGCTCAATTTCAATATGCTGGGATCCTATCTCTGAGTGCCCACCTCCCCCAAAACCTC

ACTCTCTCACCCCACCTCTGCTTCTTTTCTCCCTGCCCATTTCTTTTCTGACTTCTTTCC

CCACAACAGAATCTCTGATTCTCCACCCACGTCCTGTTCAGAGTCATCCACTTTCCTCCC

CCACCCCCCAGACTCCCGGGGCCTCTGCACCTGGGGACACTGGACACATATGTGCCCATG

ATGATGAGGACGGTGCCCACGAGGAAGCCCACCAGGCCGATGGCCAGGCCCAGGGCACAG

ACCAGGGTCTCCATGGCATCTGGTGGTGGAATAGGCACCTGGAGCTCTAGGAGAGAAAGG

AAGGAGTTGGTGGTATATGAAAGGATTCTAGAGTAAAGGAAACCTGGGGCCAGGAGGGTG

CATGGGGAGGGGGCTCCGTACCCCAATGCCTGAGGAGTGGCGCATCCAGGCCCCAGTGCT

CCACCTGGCAGTCATAGACGTCCTCGGCTGAGGGCACGAAGGGCAGGTAGTGGAACTTGC

GGAACAAATGGTCAGGCTGGGAATAGAAGCTGGTCTGGGCCACTCCCTCAGTGACAGTTT

GGCCGTTGCGCAGCCAGGTGATATTGATCACAGGGGGGAAGATGTTGTCCACGATGCAGA

TGAGGATGTTGGGCTGGCCCAGCTCCACCCGAGACTTGGGGAGCACGGTCACCCGTGGAG

GCACTAGGAGGAACAGGCCCTGAGTCCACAGGCTCATCCCTCACCCCAGGGCCTTACTAG

GACTGGGATTAAGGGACGTTCCCCCTTTGTAGCCATCTGTGGGCAGGGGATGCTCTGGGG

TATCCACTGGGGCAGGAGAGGAGGAAAACAGAGGGAGAGGAGACTGGGGAGGGAGTGGGG

ACGCCAGGAGCTCCTATATTTGACTGGTCCCTGGGCGGGAGTCCGGGTGAGAGGTGTCAT

TCCTCAAGGAGAGGGGTGCCAAAGGGGTCTGGGAAGACCTGGAGCCTCCTGGGAAAGAAA

GGAACAGGGCATGACAGGCGCGGGCGCTGAGAGCGCGCCCCAGAGTGATGGGAGCCTAGG

AACTGGGAGGAAGTTTCTCTGGACCTTCCCGCCTGACTGGGTGGGCAGAGGGAGGGCCGG

TACCGTTGATGGCTCTGCTGCGGTTGGAGCGCTCCACCAGGATGTCCAGATGGGCTTTGA

TTGCGGCGATGCCGGCCAGCCCGCCCTGCGGGTCAAAGCGGGCAAAGTCACCAAACTCAG

GCAGACGCCACACGGCCTCGCTTTTCTTCAGGTCCACAGAGAACAGCTGTTCCTCATCAA

ATTCATGGGTGAACTGGCCCGAGGCGCCGTAAGACTGGTAGAAGGCGGGTCCGTAGGAGC

CCATGTGGTCAGCTGTGTTTGGCGAGTTCAGGGTCAAGGAGAGAGAAAAAAATGTGTCTG

TCTCATCCACAATATGTGATTGTTGAGTCCCTGAGCCTGGGCCCCGTCCTGGGTTCTGTG

TGGGGACAGAGTCCTGTTCTGACACTGGGCTGGCCCTGGGAGAGAGAAAGGGAGAGAGAA

CAGGAAGAAAGAGGCTCATCCCAGCACACTGCAGTCGGCACAGAGACAGTGCAGTCTGGC

ATATCAGGATGGGAAGAGGAGGGACTGCCTAAAATCATGCTTGGGGTTCCAGAATTTAAA

TCTTGGCTGTGGTCATCTGCCCTGGCTGTGTTGTCAGGCCCTGTGTTGTGAGCTGGTGGG

ACTGTGGGGGTGGGATGAGGAGGAATGATTAAGGACAGGAGAGTATGGAGCTTTGCACAG

AGATGCAGTGCAGGTGGGTGTGAGGGGAAACAGGCCACGGCTGGCAGGGGTAAGAATTAA

GGTTAGTGACCCAGAGACCAAGGGGATAGGGAGAGGCAACTCAAGGCATTACAAAGAGCA

CTGGACGAGGAGTCAGAAGTCAAGGTTCATGTCCCAATTCCTCCATCTCAGAGCATTATG

ACTGAGTGTGGCTCTTCCATAACTGTTGTCTAGTTTTCTGGAAGTTAGGGATTAAGTTTT

AATTCTTGTAGAACTCTATGAAGTTGTTTGAGCAACAGTTATTGAGGAACTAGCATGCAC

CCAGCACAATGGTGGGCCAGGGAAATAAAAGAAAAAAAAGATGAACCATCTGTAGACCCG

CACCCCAGCTCATGTCTCCCGAAGAACAAAGACAGGTAAATAGTTAACTACCGGCATGGG

CATAAATACTGCAACAGAACTGGACTTGATCGGGCACATTCCAGGCCAGGGGTGGTAGAG

AAATCAGGGTGCTTGCTGGCATCTGTTGGGTGGAGGTTTGGGTCTCAGGAAGGAGGAAGG

AATGAGGAGAAATCTGAACGTCAGCAAAGGCTGACTGGGGCACCTGCGCAGCTGACCGAG

CTGCATCTTCATTTAGGTCCAGAGTGGATGTGACAGAGATGAGGGGGATTGGGTGTCTCT

TGGTGAAGGAAGTTGCCCATAAACCAGAGAGCGAGAGGAACAAGCATCCTCCATGCCACC

TCCTCATGTAACCCAACTCCGTAAATCTCTGCTCCCCGCCGCACCCTCCTCGCCCTCGCA

CTCACCCTTGGTGGCCCCTGCCTCCTGCGGGCTCAGGAGGGTCATCAGGGTGTGGAACCC

CAGGACCAGCCCTGCTCTGAGGGCCATTACACTCTGGTGCTTTAATCAAATCAGTCTCAG

TCCGTGTGGTGAGGACAGGAACAAGGCGGAGGTAAAGAAGAAGAAAACAGATTCGAGGAT

GGGGGCGACCCCTGCTGTCTTCAGCCAATCACAGAAATTCTCTGAGTGAATGTATCTGTT

GCTGGGTAAAGAGGGAAAGAGCCGGGGTGAGAAGGTGGAAGGATTCACTGGGCCCCCAGG

AGAGGCCAGAGGAAGTTTTGGAGGATGGGAGGGGCTTGGACCAACTATTACCACGTCCTC

CAAGAAGGGACCCCCTGAAGAGAGAGAAAAGGCCGTCAGAGCACCGCGCAGCT

>HLA:HLA00997 DOA*01:01:04:01

AGCTGCGCGGTGCTCTGACGGCCTTTTCTCTCTCTTCAGGGGGTCCCTTCTTGGAGGACG

TGGTAATAGTTGGTCCAAGCCCCTCCCATCCTCCAAAACTTCCTTTGGCCTCTCCTGGGG

GCCCAGTGAATCCTTCCACCTTCTCACCCCGGCTCTTTCCCTCTTTACCCAGCAACAGAT

ACATTCACTCAGAGAATTTCTGTGATTGGCTGAAGACAGCAGGGGTCGCCCCCATCCTCG

AATCTGTTTTCTTCTTCTTTACCTCCGCCTTGTTCCTGTCCTCACCACACGGACTGAGAC

TGATTTGATTAAAGCACCAGAGTGTAATGGCCCTCAGAGCAGGGCTGGTCCTGGGGTTCC

ACACCCTGATGACCCTCCTGAGCCCGCAGGAGGCAGGGGCCACCAAGGGTGAGTGCGAGG

GCGAGGAGGGTGCGGCGGGGAGCAGAGATTTACGGAGTTGGGTTACATGAGGAGGTGGCA

TGGAGGATGCTTGTTCCTCTCGCTCTCTGGTTTATGGGCAACTTCCTTCACCAAGAGACA

CCCAATCCCCCTCATCTCTGTCACATCCACTCTGGACCTAAATGAAGATGCAGCTCGGTC

AGCTGCGCAGGTGCCCCAGTCAGCCTTTGCTGACGTTCAGATTTCTCCTCATTCCTTCCT

CCTTCCTGAGACCCAAACCTCCACCCAACAGATGCCAGCAAGCACCCTGATTTCTCTACC

ACCCCTGGCCGGGAATGTGCCCGATCAAGTCCAGTTCTGTTGCAGTATTTATGCCCATGC

CGGTAGTTAACTATTTACCTGTCTTTGTTCTTCGGGAGACATGAGCTGGGGTGCGGGTCT

ACAGATGGTTCATCTTTTTTTTCTTTTATTTCCCTGGCCCACCATTGTGCTGGGTGCATG

CTAGTTCCTCAATAACTGTTGCTCAAACAACTTCATAGAGTTCTACAAGAATTAAAACTT

AATCCCTAACTTCCAGAAAACTAGACAACAGTTATGGAAGAGCCACACTCAGTCATAATG

CTCTGAGATGGAGGAATTGGGACATGAACCTTGACTTCTGACTCCTCGTCCAGTGCTCTT

TGTAATGCCTTGAGTTGCCTCTCCCTATCCCCTTGGTCTCTGGGTCACTAACCTTAATTC

TTACCCCTGCCAGCCGTGGCCTGTTTCCCCTCACACCCACCTGCACTGCATCTCTGTGCA

AAGCTCCATACTCTCCTGTCCTTAATCATTCCTCCTCATCCCACCCCCACAGTCCCACCA

GCTCACAACACAGGGCCTGACAACACAGCCAGGGCAGATGACCACAGCCAAGATTTAAAT

TCTGGAACCCCAAGCATGATTTTAGGCAGTCCCTCCTCTTCCCATCCTGATATGCCAGAC

TGCACTGTCTCTGTGCCGACTGCAGTGTGCTGGGATGAGCCTCTTTCTTCCTGTTCTCTC

TCCCTTTCTCTCTCCCAGGGCCAGCCCAGTGTCAGAACAGGACTCTGTCCCCACACAGAA

CCCAGGACGGGGCCCAGGCTCAGGGACTCAACAATCACATATTGTGGATGAGACAGACAC

ATTTTTTTCTCTCTCCTTGACCCTGAACTCGCCAAACACAGCTGACCACATGGGCTCCTA

CGGACCCGCCTTCTACCAGTCTTACGGCGCCTCGGGCCAGTTCACCCATGAATTTGATGA

GGAACAGCTGTTCTCTGTGGACCTGAAGAAAAGCGAGGCCGTGTGGCGTCTGCCTGAGTT

TGGTGACTTTGCCCGCTTTGACCCGCAGGGCGGGCTGGCCGGCATCGCCGCAATCAAAGC

CCATCTGGACATCCTGGTGGAGCGCTCCAACCGCAGCAGAGCCATCAACGGTACCGGCCC

TCCCTCTGCCCACCCAGTCAGGCGGGAAGGTCCAGAGAAACTTCCTCCCAGTTCCTAGGC

TCCCATCACTCTGGGGCGCGGTCTCAGCGCCCGCGCCTGTCATGCCCTGTTCCTTTCTTT

CCCAGGAGGCTCCAGGTCTTCCCAGACCCCTTTGGCACCCCTCTCCTTGAGGAATGACAC

CTCTCACCCGGACTCCCGCCCGGGGACCAGTCAAATATAGGAGCTCCTGGCGTCCCCACT

CCCTCCCCAGTCTCCTCTCCCTCTGTTTCCCTCCTCTCCTGCCCCAGTGGATACCCCAGA

GCATCCCCTGCCCACAGATGGCTACAAAGGGGGAACGTCCCTTAATCCCAGTCCTAGTAA

GGCCCTGGGGTGAGGGATGAGCCTGTGGACTCAGGGCCCGTTCCTCCTAGTGCCTCCACG

GGTGACCGTGCTCCCCAAGTCTCGGGTGGAGCTGGGCCAGCCCAACATCCTCATCTGCAT

CGTGGACAACATCTTCCCCCCTGTGATCAATATCACCTGGCTACGCAACGGCCAAACTGT

CACTGAGGGAGTGGCCCAGACCAGCTTCTATTCCCAGCCTGACCATTTGTTCCGCAAGTT

CCACTACCTGCCCTTCGTGCCCTCAGCCGAGGACGTCTATGACTGCCAGGTGGAGCACTG

GGGCCTGGATGCGCCACTCCTCAGGCATTGGGGTACGGAGCCCCCTCCCCATGCACCCTC

CTGGCCCCAGGTTTCCTTTACTCTAGAATCCTTTCATATACCACCGACTCCTTCCTTTCT

CTCCTAGAGCTCCAGGTGCCTATTCCACCACCAGATGCCATGGAGACCCTGGTCTGTGCC

CTGGGCCTGGCCATCGGCCTGGTGGGCTTCCTCGTGGGCACCGTCCTCATCATCATGGGC

ACATATGTGTCCAGTGTCCCCAGGTGCAGAGGCCCCAGGAGTCTGGGGGGTGGGGGAGGA

AAGTGGATGACTCTGAACAGGACGTGGGTGGAGAATCAGAGATTCTGTTGTGGGGAAAGA

AGTCAGAAAAGAAATGGGCAGGGAGAAAAGAAGCAGAGGTGGGGTGAGAGAGTGAGGTTT

TGGGGGAGGTGGGCACTCAGAGATAGGATCCCAGCATATTGAAATTGAGCAACCTCGATC

GTATGTTTTCTGCTATTTTAGGTAATGATCCTTCTGAGAGAAATGACTTGTGGGAGACAC

CCTGCAGATCCTCATGGGTTTGTGACAGCCCCTGCGTGCTCAGTGCCCTTTAAGTGCATC

CCGCTGTGCTGACTTTGAGTGGGATCAACATCTGTCCTACGGGTCCCCTCTTTTTTGGCC

CCAGTATTCATGGCAGGGTTTGTTGGACACCTACTAGCTTCCCTTCCCATTCAACACAAA

CACACATTCTTGCTCTACCCAAAGCTCTGGCTGGCAGCACTAAATGCTTTGGTGGTGTTT

GCACTGTGTCCTTTCCAGGCCTTGGCCAGTTCTTCCAGGGGTGAGGCATGTGGTGCTGGG

GATTGGCAGCCGTCCTGGGGCCCACACAGGTGTGTCTTGCTCCATTTGGCCCATTGTGTG

TTACTTTGTGAATGAGCCATTTCACATGGACTTCATGAAATTTGCCTCCTGAGTTCAGGT

TTACCCTGAAAGGGATGCAGATTATCCTGTTCCTCACGACCCCCTCAGCTAACAACAGTT

CTGAAGGGTGCTGGGACAAGACAGGCTCATGGGGACTCCACTCCTGCCTGGGTTTACTCT

GTATGAAGAGGCCACTGGTATCCTGCCATGATGTTATCTCCTTTTTCTACTTT

>HLA:HLA00999 DOA*01:01:05

AGCTGCGCGGTGCTCTGACGGCCTTTTCTCTCTCTTCAGGGGGTCCCTTCTTGGAGGACG

TGGTAATAGTTGGTCCAAGCCCCTCCCATCCTCCAAAACTTCCTCTGGCCTCTCCTGGGG

GCCCAGTGAATCCTTCCACCTTCTCACCCCGGCTCTTTCCCTCTTTACCCAGCAACAGAT

ACATTCACTCAGAGAATTTCTGTGATTGGCTGAAGACAGCAGGGGTCGCCCCCATCCTCG

AATCTGTTTTCTTCTTCTTTACCTCCGCCTTGTTCCTGTCCTCACCACACGGACTGAGAC

TGATTTGATTAAAGCACCAGAGTGCAATGGCCCTCAGAGCAGGGCTGGTCCTGGGGTTCC

ACACCCTGATGACCCTCCTGAGCCCGCAGGAGGCAGGGGCCACCAAGGGTGAGTGCGAGG

GCGAGGAGGGTGCGGCGGGGAGCAGAGATTTACGGAGTTGGGTTACATGAGGAGGTGGCA

TGGAGGATGCTTGTTCCTCTCGCTCTCTGGTTTATGGGCAACTTCCTTCACCAAGAGACA

CCCAATCCCCCTCATCTCTGTCACATCCACTCTGGACCTAAATGAAGATGCAGCTCGGTC

AGCTGCGCAGGTGCCCCAGTCAGCCTTTGCTGACGTTCAGATTTCTCCTCATTCCTTCCT

CCTTCCTGAGACCCAAACCTCCACCCAACAGATGCCAGCAAGCACCCTGATTCCTCTACC

ACCCCTGGCCTGGAATGTGCCCGATCAAGTCCAGTTCTGTTGCAGTATTTATGCCCATGC

CGGTAGTTAACTATTTACCTGTCTTTGTTCTTCGGGAGACATGAGCTGGGGTGCGGGTCT

ACAGATGGTTCATCTTTTTTTTCTTTTATTTCCCTGGCCCACCATTGTGCTGGGTGCATG

CTAGTTCCTCAATAACTGTTGCTCAAACAACTTCATAGAGTTCTACAAGAATTAAAACTT

AATCCCTAACTTCCAGAAAACTAGACAACAGTTATGGAAGAGCCACACTCAGTCATAATG

CTCTGAGATGGAGGAATTGGGACATGAACCTTGACTTCTGACTCCTCGTCCAGTGCTCTT

TGTAATGCCTTGAGTTGCCTCTCCCTATCCCCTTGGTCTCTGGGTCACTAACCTTAATTC

TTACCCCTGCCAGCCGTGGCCTGTTTCCCCTCACACCCACCTGCACTGCATCTCTGTGCA

AAGCTCCATACTCTCCTGTCCTTAATCATTCCTCCTCATCCCACCCCCACAGTCCCACCA

GCTCACAACACAGGGCCTGACAACACAGCCAGGGCAGATGACCACAGCCAAGATTTAAAT

TCTGGAACCCCAAGCATGATTTTAGGCAGTCCCTCCTCTTCCCATCCTGATATGCCAGAC

TGCACTGTCTCTGTGCCGACTGCAGTGTGCTGGGATGAGCCTCTTTCTTCCTGTTCTCTC

TCCCTTTCTCTCTCCCAGGGCCAGCCCAGTGTCAGAACAGGACTCTGTCCCCACACAGAA

CCCAGGACGGGGCCCAGGCTCAGGGACTCAACAATCACATATTGTGGATGAGACAGACAC

ATTTTTTTCTCTCTCCTTGACCCTGAACTCGCCAAACACAGCTGACCACATGGGCTCCTA

CGGACCCGCCTTCTACCAGTCTTACGGCGCCTCGGGCCAGTTCACCCATGAATTTGATGA

GGAACAGCTGTTCTCTGTGGACCTGAAGAAAAGCGAGGCCGTGTGGCGTCTGCCTGAGTT

TGGCGACTTTGCCCGCTTTGACCCGCAGGGCGGGCTGGCCGGCATCGCCGCAATCAAAGC

CCATCTGGACATCCTGGTGGAGCGCTCCAACCGCAGCAGAGCCATCAACGGTACCGGCCC

TCCCTCTGCCCACCCAGTCAGGCGGGAAGGTCCAGAGAAACTTCCTCCCAGTTCCTAGGC

TCCCATCACTCTGGGGCGCGCTCTCAGCGCCCGCGCCTGTCATGCCCTGTTCCTTTCTTT

CCCAGGAGGCTCCAGGTCTTCCCAGACCCCTTTGGCACCCCTCTCCTTGAGGAATGACAC

CTCTCACCCGGACTCCCGCCCAGGGACCAGTCAAATATAGGAGCTCCTGGCGTCCCCACT

CCCTCCCCAGTCTCCTCTCCCTCTGTTTCCCTCCTCTCCTGCCCCAGTGGATACCCCAGA

GCATCCCCTGCCCACAGATGGCTACAAAGGGGGAACGTCCCTTAATCCCAGTCCTAGTAA

GGCCCTGGGGTGAGGGATGAGCCTGTGGACTCAGGGCCTGTTCCTCCTAGTGCCTCCACG

GGTGACCGTGCTCCCCAAGTCTCGGGTGGAGCTGGGCCAGCCCAACATCCTCATCTGCAT

CGTGGACAACATCTTCCCCCCTGTGATCAATATCACCTGGCTGCGCAACGGCCAAACTGT

CACTGAGGGAGTGGCCCAGACCAGCTTCTATTCCCAGCCTGACCATTTGTTCCGCAAGTT

CCACTACCTGCCCTTCGTGCCCTCAGCCGAGGACGTCTATGACTGCCAGGTGGAGCACTG

GGGCCTGGATGCGCCACTCCTCAGGCATTGGGGTACGGAGCCCCCTCCCCATGCACCCTC

CTGGCCCCAGGTTTCCTTTACTCTAGAATCCTTTCATATACCACCAACTCCTTCCTTTCT

CTCCTAGAGCTCCAGGTGCCTATTCCACCACCAGATGCCATGGAGACCCTGGTCTGTGCC

CTGGGCCTGGCCATCGGCCTGGTGGGCTTCCTCGTGGGCACCGTCCTCATCATCATGGGC

ACATATGTGTCCAGTGTCCCCAGGTGCAGAGGCCCCGGGAGTCTGGGGGGTGGGGGAGGA

AAGTGGATGACTCTGAACAGGACGTGGGTGGAGAATCAGAGATTCTGTTGTGGGGAAAGA

AGTCAGAAAAGAAATGGGCAGGGAGAAAAGAAGCAGAGGTGGGGTGAGAGAGTGAGGTTT

TGGGGGAGGTGGGCACTCAGAGATAGGATCCCAGCATATTGAAATTGAGCAACCTCGATC

GTATGTTTTCTGCTATTTTAGGTAATGATCCTTCTGAGAGAAATGACTTGTGGGAGACAC

CCTGCAGATCCTCATGGGTTTGTGACAGCCCCTGCGTGCTCAGTGCCCTTTAAGTGCATC

CCGCTGTGCTGACTTTGAGTGGGATCAACATCTGTCCTACGGGTCCCCTCTTTTTTGGCC

CCAGTATTCATGGCAGGGTTTGTTGGACACCTACTAGCTTCCCTTCCCATTCAACACACA

CACACATTCTTGCTCTACCCAAAGCTCTGGCTGGCAGCACTAAATGCTTTGGTGGTATTT

GCACTGTGTCCTTTCCAGGCCTTGGCCAGTTCTTCCAGGGGTGAGGCATGTGGTGCTGGG

GATTGGCAGCCATCCTGGGGCCCACACAGGTGTGTCTTGCTCCATTTGGCCCATTGTGTG

TTACTTTGTGAATGAGCCATTTCACATGGACTTCATGAAATTTGCCTCCTGAGTTCAGGT

TTACCCTGAAAGGGATGCAGATTATCCTGTTCCTCACGACCCCCTCAGCTAACAACAGTT

CTGAAGGGTGCTGGGACAGGACAGGCTCATGGGGACTCCACTCCTGCCTGGGTTTACTCT

GTATGAAGAGGCCACTGGTATCCTGCCATGATGTTATCTCCTTTTTCTACTTT

>HLA:HLA06455 DOB*01:01:01:04

CATAATAAACAGTCTGCCTAGTTCTTCTAGGGCCCCATAATATGTCAAACATATATTTTT

ACTTCTTCTCCCAGCCCATTTTTAGTATACCTAAATTACTGTCAGTGATTCTGCAGGCAA

ACAATGGTTGAGTTGTATGACACAACTTTGTGAAAGTATCCTACCCAGTACCTGATGATG

CAAAACTCTTCTATCTTGATTGGTTGTCAATCTGAGGAGTTTCCAATCCTGGGGAAGCCA

GAAAAACAGCGATTTATACTCTTAATGGGTACTTTCTGACTGAATTTTATGAACTCATTC

TGAAGAGGCTGACGATTTTACTGTCTCATTTTTTTCCTTTCTCCAGAATGGGTTCTGGGT

GGGTCCCCTGGGTGGTGGCTCTGCTAGTGAATCTGACCCGACTGGATTCCTCCATGACTC

AAGGCACAGACTCTCCAGGTAAGAACAGAGCAATTATTTTTTTCCAGTGTGTATGCAAGA

ATTGGCATGGGGGAGTGATGCCTTTCTTTGTAAGTCCAGGCCACAGACCAGACTGGAAGT

GGCTTTTGGTTTCAAAGAACAGTGTTCTTCCCTTTGGCAGAAAGGTACGCCTTGCCTCTT

TACATGGGATGGACTTCATATACCAGAGCCACCTATTCAAGGGGTAGGGAGGCAGGAAGA

GGGAAACATTGTGTCTTGTTTAGGATCCTTATTGTGTGTATCAACCTCAGTCAGTGCCTG

GGCGTGTTGAAGGCCTTGGCTTGGGTTCGAGCCTGCTGGGAGAAACAACCTGCAGTAGGC

TGGGTCACAGAGGCAATCTGTGATTTTTTGGTCAGGACACGGAAACAAATCTCAGTTGGG

GTATATGTGGACAAATGAAACTGGAAACAAAGGTTGCTCCTTCTGTCATTTATTAAGCCA

CTATTATATTGTCAGAATTGTACTAAACAGTTTTGAGAAGTAAGAGAAGTTGAATAGAAT

ACATTGTCCTTGTCCTCCGGCTACCAGGTACAAGTTACTTGTAACTGTTATTTTTCTAGC

ACAGGTGACAGAATATGCAGCCATGAAGCAATGTGAGATGAAAGCACATATTAATGAGCA

GAAACAGGATGTAATGTGCTAAGAACAGAATCCCCTTTGCATGTTAGTTTCATTAAATAC

AAAAGAGGAACAAACCTGGCCAGGAGAGATCATTATTCTTAGAGAATAGAAACCGCCCTG

AGTTTATAATGTCCATTAAACAATACAACTGAAAAAAAAATCAGCACAGATGTTAAATGA

TGATGAAAAATTCAGATTTCCCCCCTGGTTTAGACTACTAGAGGAAATAGAGAAGAGTAT

ACATGCTGAGAAATTACAGGCTGGAACTTCATCTGAAATTAGCTACTGAGTGAGGGATAA

GTGGGGTTCACCCAGGAAGGTCATTCTTATGGCTCAGTTCAGAGTTGGAGGAGGCTTCTG

AACTTAGAAAGGAAAGTAAATTACAACCCAACATTAATAGCAATTATCTTTCAAGTCTTG

ACTTAGATGCAATGTCTTCAGGACGTCCTTCCTGACTTACCTACATTATTAACTCCATTT

GAATTTCCTTTTTATTGTAGTTGTTGTTCTTAAGTGCATAGGATTGGTTTAATTTTACCC

AATGAGTTCACAGCACATTGTAATTATTGGCAGTAGTGCAAGACTCTCTCGTCTCTTCTC

TTGCCTCCGTTCTCATTCTCTCCCCTCCCTAGAGAATCCATTCTAAACGTGTCTGGTATG

TCTCTAAGTATGAGAGTGGCTTTTAGAAATATGTAGCATTCTTGCTCTTTATGTGTTTTT

AAAATTTAATAAATGTCATTCTCTGTTGAATCCCATTCTGTTTCTTTTCTCACTTGACAC

TGTGCTTTTTGAGCATACTGAGGTCAAAGGCCTCCTCTTAGATCCATGTGGTCTGACGTA

ATTTACCAGGCATGGGTTTTCCCAGAGGAGGGGGCTGGTTCATGGTTTTGGTTTTGGTTT

TCCAGAAGATTTTGTGATTCAGGCAAAGGCTGACTGTTACTTCACCAACGGGACAGAAAA

GGTGCAGTTTGTGGTCAGATTCATCTTTAACTTGGAGGAGTATGTACGTTTCGACAGTGA

TGTGGGGATGTTTGTGGCATTGACCAAGCTGGGGCAGCCAGATGCTGAGCAGTGGAACAG

CCGGCTGGATCTCTTGGAGAGGAGCAGACAGGCCGTGGATGGGGTCTGTAGACACAACTA

CAGGCTGGGCGCACCCTTCACTGTGGGGAGAAAAGGTGAGCTGGAAGCTGAGGTCTGGCG

GGGCTCAGGAATGTCCCCCATGTGAACCTGGCCATGGCTCTTCTTTCTTACAAGCAATTT

TCTGCTTTAGGATAAATGGTTGTCTGTGTAGATGTTCTGGCCCCAGCTGTGATATATTAT

CCTCACAAGTCAGCCACTGTGATCTTGGTCTCAGACCCCCAAGGTTCTCAGGGACTTCGA

GGGCTATTGTACCCTCAAAGAGAAGCAGTAATTATGGGAGTACCTCAGAAAGTCTAAATC

CTCCTGACAGGCATTGACATACCCTGTTACTGATCTTGGGGGCTGAGACTTGCCTATACT

TTGTGTTCACTTGGGTGATCTGGGAAAGAGATTAGACATAGTGATAGTCCCTAAAGAATC

TCCTGTCCCAGCTTGGTGGTTTTCTTTCACGGTGTCTCATTTTTCCTCCCTTCCTAGTGC

AACCAGAGGTGACAGTGTACCCAGAGAGGACCCCACTCCTGCACCAGCATAATCTGCTGC

ACTGCTCTGTGACAGGCTTCTATCCAGGGGATATCAAGATCAAGTGGTTCCTGAATGGGC

AGGAGGAGAGAGCTGGGGTCATGTCCACTGGCCCTATCAGGAATGGAGACTGGACCTTTC

AGACTGTGGTGATGCTAGAAATGACTCCTGAACTTGGACATGTCTACACCTGCCTTGTCG

ATCACTCCAGCCTGCTGAGCCCTGTTTCTGTGGAGTGGAGTGAGAATTAGTTTCTAGTAC

TCTCTGGGCCTGACTCAGGACTATACTGACTCAATACAGAGCCTGTGTCACTTCTGCGTT

TATCTTGGTCACAACATGAATTATTCTTTCCCTTGATCTGGGACAGTCACAGAAACCAGA

GTCCTTGGGTTAGGGTGGGAGAAAACATGGCAGATATCTATCCTCATATCTTCCAAGAAA

TGAGGAGATCTAATCACCTCATTATGTGCTTCCAACCCTATGAACTGGTGTCCTCTAATT

CTTTGGTCTTAGTATTTAGGAGGCATTCTTATGGGCTGTGAGAATCTGTAACCGATGGGT

GGTAACTCCATGGGTGCCAACTTTGGTTTCGAAGAACCTTTTCTAAATTTATTTATTTTT

CTCTAGCTAGCATTGGATTTGGTGTCTAGTACAGATTCTGGGATTCCAAGAAAGTGCTTT

AAATATTGGGATATTTTTACTAATTTAAAGACCTGTTTCCCATAGGAGCTCAGTCTGAAT

ATTCTTGGAGAAAGATGCTGAGTGGCATTGCAGCCTTCCTACTTGGGCTAATCTTCCTTC

TGGTGGGAATCGTCATCCAGCTAAGGGCTCAGAAAGGTAATGAGCCTGTGAGGAGTGCCC

TGCCACCTGTCCCAGACCTTCCCCACTCCCACCTTCCCTAACGTCAATGATCTGAGGCAA

GGAAAGCTGATTGTGCCTCTCAGGGATCACCGGGATAATTTTTTTCTGAAGCTAGAAATG

GGATAAGCAGAGAGAGTGCTGACCTTGCCAGCCATTTGTTCTTCCCTCGGGATAATCATA

TTGGGTCCTAATTGGGGCAATCCATTCTTTTCTCGATTTCTTTCCAGGATATGTGAGGAC

GCAGATGTCTGGTAATGAGGTAATGTCTCTTTTTCCTTGTCTTTGAGTGGCAGATCATTC

TCCCGGTTCTTTGGCCAGAGGGAGATGACATGGGGGTAGGGAGGAGTAAGGTTGCTGCTG

TCTGGATGGGACTGTCCCCTGAGTCTCTGGAACGGCTGTGGGGGGTGGTGAGGCTGCCTC

CTGAGACCTTCATCACTGTGCCTCCAGGTCTCAAGAGCTGTTCTGCTCCCTCAGTCATGC

TAAGGTCCTCACTGAAGCTTCTCTCTCTGGAGCCTGAAGTAGTGATGAGTAGTCTGGGCC

CTGGGTGAGGTAAAGGACATTCATGAGGTCAATGTTCTGGGAATAACTCTCTTCCCTGAT

CCTTGGAGGAGCCCGAACTGATTCTGGAGCTCTGTGTTCTGAGATCATGCATCTCCCACC

CATCTGCCCTTCTCCCTTCTACGTGTACATCATTAATCCCCATTGCCAAGGGCATTGTCC

AGAAACTCCCCTGAGACCTTACTCCTTCCAGCCCCAAATCATTTACTTTTCTGTGGTCCA

GCCCTACTCCTATAAGTCATGATCTCCAAAGCTTTCTGTCTTCCAACTGCAGTCTCCACA

GTCTTCAGAAGACAAATGCTCAGGTAGTCACTGTTTCCTTTTCACTGTTTTTAAAAACCT

TTTATTGTCAAATAAAATGGAGATACAAAAAATGTACATTTTAGTGAATTATTTAAGAAA

AACCCCTGTAATCAAGTCAAGGAACAGGACTTTGCCAGCTCCAGCAGAAGTCTCTGTACG

TCAGGCCAATCAAAGCCTCTCCTTCCCCTCGAAAGTGACCATATCCTGATTTTATTGTAA

CCTCTTTCATGTCTTTGTAGTCTAGTCCCCCAGGTATGTGTTCCTGGACGCCACAGCTTA

GTTGTCTTTTACGTCTCTTATACTTCACTGGTTTCTCCCCCATCAG

>HLA01099 DOB*01:02:01:01

CTGATGGGGGAGAAACCAGTGAAGTATAAGAGACGTAAAAGACAACTAAGCTGTGGCGTC

CAGGAACACATACCTGGGGGACTAGACTACAAAGACATGAAAGAGGTTACAATAAAATCA

GGATATGGTCACTTTCGAGGGGAAGGAGAGGCTTTGATTGGCCTGACGTACAGAGACTTC

TGCTGGAGCTGGCAAAGTCCTGTTCCTTGACTTGATTACAGGGGTTTTTCTTAAATAATT

CACTAAAATGTACATTTTTTGTATCTCCATTTTATTTGACAATAAAAGGTTTTTAAAAAC

AGTGAAAAGGAAACAGTGACTACCTGAGCATTTGTCTTCTGAAGACTGTGGAGACTGCAG

TTGGAAGACAGAAAGCTTTGGAGATCATGACTTATAGGAGTAGGGCTGGACCACAGAAAA

GTAAATGATTTGGGACTGGAAGGAGTAAGGTCTCAGGGGAGTTTCTGGACAATGCCCTTG

GCAATGGGGATTAATGATGTACACGTAGAAGGGAGAAGGGCAGATGGGTGGGAGATGCAT

GATCTCAGAACACAGAGCTCCAGAATCAGTTCGGGCTCCTCCAAGGATCAGGGAAGAGAG

TTATTCCCAGAACATTGACCTCATGAATGTCCTTTACCTCACCCAGGGCCCAGACTACTC

ATCACTACTTCAGGCTCCAGAGAGAGAAGCTTCAGTGAGGACCTTAGCATGACTGAGGGA

GCAGAACAGCTCTTGAGACCTGGAGGCACAGTGATGAAGGTCTCAGGAGGCAGCCTCACC

ACCCCCCACAGCCGTTCCAGAGACTCAGGGGACAGTCCCATCTAGACAGCAGCAACCTTA

CTCCTCCCTACCCCCATGTCATCTCCCTCTGGCCAAAGAACCGGGAGAATGATCTGCCAC

TCAAAGACAAGGAAAAAGAGACATTACCTCATTACCAGACATCTGCGTCCTCACATATCC

TGGAAAGAAATCGAGAAAAGAATGGATTGCCCCAATTAGGACCCAATATGATTATCCCGA

GGGAAGAACAAATGGCTGGCAAGGTCAGCACTCTCTCTGCTTATCCCATTTCTAGCTTCA

GAAAAAAATTATCCCGGTGATCCCTGAGAGGCACAATCAGCTTTCCTTGCCTCAGATCAT

TGACGTTAGGGAAGGTGGGAGTGGGGAAGGTCTGGGACAGGTGGCAGGGCACTCCTCACA

GGCTCATTACCTTTCTGAGCCCTTAGCTGGATGACGATTCCCACCAGAAGGAAGATTAGC

CCAAGTAGGAAGGCTGCAATGCCACTCAGCATCTTTCTCCAAGAATATTCAGACTGAGCT

CCTATGGGAAACAGGTCTTTAAATTAGTAAAAATATCCCAATATTTAAAGCACTTTCTTG

GAATCCCAGAATCTGTACTAGACACCAAATCCAATGCTAGCTAGAGAAAAATAAATAAAT

TTAGAAAAGGTTCTTCGAAACCAAAGTGGCACCCATGGAGTTACCACCCATCGGTTACAG

ATTCTCACAGCCCATAAGAATGCCTCCTAAATACTAAGACCAAAGAATTAGAGGACACCA

GTTCATAGGGTTGGAAGCACATAATGAGGTGATTAGATCTCCTCATTTCTTGGAAGATAT

GAGGATAGATATCTGCCATGTTTTCTCCCACCCTAACCCAAGGACTCTGGTTTCTGTGAC

TGTCCCAGATCAAGGGAAAGAATAATTCATGTTGTGACCAAGATAAACGCAGAAGTGACA

CAGGCTCTGTATTGAGTCAGTATAGTCCTGAGTCAGGCCCAGAGAGTACTAGAAACTAAT

TCTCACTCCACTCCACAGAAACAGGGCTCAGCAGGCTGGAGTGATCGACAAGGCAGGTGT

AGACATGTCCAAGTTCAGGAGTCATTTCTAGCATCACCACAGTCTGAAAGGTCCAGTCTC

CATTCCTGATAGGGCCAGTGGACATGACCCCAGCTCTCTCCTCCTGCCCATTCAGGAACC

ACTTGATCTTGATATCCCCTGGATAGAAGCCTGTCACAGAGCAGTGCAGCAGATTATGCT

GGTGCAGGAGTGGGGTCCTCTCTGGGTACACTGTCACCTCTGGTTGCACTAGGAAGGGAG

GAAAAATGAGACACCGTGAAAGAAAACCACCAAGCTGGGACAGGAGATTCTTTAGGGACT

ATCACTATGTCTAATCTCTTTCCCAGATCACCCAAGTGAACACAAAGTATAGGCAAGTCT

CAGCCCCCAAGATCAGTAACAGGGTATGTCAATGCCTGTCAGGAGGATTTAGACTTTCTG

AGGTACTCCCACAATTACTGCTTCTCTTTGAGGGTACAATAGCCCTCGAAGTCCCTGAGA

ACCTTGGGGGTCTGAGACCAAGATCACAGTGGCTGACTTGTGAGGATAATATATCACAGC

TGGGGCCAGAACATCTACACAGACAACCATTTATCCTAAAGCAGAAAATTGCTTGTAAGA

AAGAAGAGCCATGGCCAGGTTCACATGGGGGACATTCCTGAGCCCCGCCAGACCTCAGCT

TCCAGCTCACCTTTTCTCCCCACAGTGAAGGGTGCGCCCAGCCTGTAGTTGTGTCTACAG

ACCCCATCCACGGCCTGTCTGCTCCTCTCCAAGAGATCCAGCCGGCTGTTCCACTGCTCA

GCATCTGGCTGCCCCAGCTTGGTCAATGCCACAAACATCCCCACATCACTGTCGAAACGT

ACATACTCCTCCAAGTTAAAGATGAATCTGACCACAAACTGCACCTTTTCTGTCCCGTTG

GTGAAGTAACAGTCAGCCTTTGCCTGAATCACAAAATCTTCTGGAAAACCAAAACCAAAA

CCATGAACCAGCCCCCTCCTCTGGGAAAACCCATGCCTGGTAAATTACGTCAGACCACAT

GGATCTAAGAGGAGGCCTTTGACCTCAGTATGCTCAAAAAGCACAGTGTCAAGTGAGAAA

AGAAACAGAATGGGATTCAACAGAGAATGACATTTATTAAATTTTAAAAACACATAAAGA

GCAATAATGCTACATATTTCTAAAAGCCACTCTCATACTTAGAGACATACCAGACACGTT

TAGAATGGATTCTCTAGGGAGGGGAGAGAATGAGAACGGAGGCAAGAGAAGAGATGAGAG

AGTCTTGCACTACTGCCAATAATTACAATGTGCTGTGAACTCATTGGGTAAAATTAAACC

AATCCTATGCACTTAAGAACAACAACTACAATAAAAAGGAAATTCAAATGGAGTTAATAA

TGTAGGTAAGTCAGGAAGGACGTCCTGAAGACATTGCATCTAAGTCAAGACTTGAAAGAT

AATTGCTATTAATGTTGGGTTGTAATTTACTTTCCTTTCTAAGTTCAGAAGCCTCCTCCA

ACTCTGAACTGAGCCATAAGAATGACCTTCCTGGGTGAACCCCACTTATCCCTCACTCAG

TAGCTAATTTCAGATGAAGTTCCAGCCTGTAATTTCTCAGCATGTATACTCTTCTCTATT

TCCTCTAGTAGTCTAAACCAGGGGGGAAATCTGAATTTTTCATCATCATTTAACATCTGT

GCTGATTTTTTTTTCAGTTGTATTGTTTAATGGACATTATAAACTCAGGGCGGTTTCTAT

TCTCTAAGAATAATGATCTCTCCTGGCCAGGTTTGTTCCTCTTTTGTATTTAATGAAACT

AACATGCAAAGGGGATTCTGTTCTTAGCACATTACATCCTGTTTCTGCTCATTAATATGT

GCTTTCATCTCACATTGCTTCATGGCTGCATATTCTGTCACCTGTGCTAGAAAAATAACA

GTGACAAGTAACTTGTACCTGGTAGCCGGAGGACAAGGACAATGTATTCTATTCAACTTC

TCTTACTTCTCAAAACTGTTTAGTACAATTCTGACAATATAATAGTGGCTTAATAAATGA

CAGAAGGAGCAACCTTTGTTTCCAGTTTCATTTGTCCACATATACCCCAACTGAGATTTG

TTTCCGTGTCCTGACCAAAAAATCACAGATTGCCTCTGTGACCCAGCCTACTGCAGGTTG

TTTCTCCCAGCAGGCTCGAACCCAAGCCAAGGCCTTCAACACGCCCAGGCACTGACTGAG

GTTGATACACACAATAAGGATCCTAAACAAGACACAATGTTTCCCTCTTCCTGCCTCCCT

ACCCCTTGAATAGGTGGCTCTGGTATATGAAGTCCATCCCATGTAAAGAGGCAAGGCGTA

CCTTTCTGCCAAAGGGAAGAACACTGTTCTTTGAAACCAAAAGCCACTTCCAGTCTGGTC

TGTGGCCTGGACTTACAAAGAAAGGCATCACTCCCCCATGCCAATTCTTGCATACACACT

GGAAAAAAACAATTGCTCTGTTCTTACCTGGAGAGTCTGTGCCTTGAGTCATGGAGGAAT

CCAGTTGGGTCAGATTCACTAGCAGAGCCACCACCCAGGGGACCCACCCAGAACCCATTC

TGGAGAAAGGAAAAAAATGAGATAGTAAAATCGTCAGCCTCTTCAGAATGAGCTCATAAA

ATTCAGTCAGAAAGTACCCATTAAGAGTATAAATCGCTGTTTTTCTGGCTTCCCCAGGAT

TGGAAACTCCTCAGATTGACAACCAATCAAGATAGAAGAGTTTTGCATCATCAGGTACTG

GGTAGGATACTTTCACAAAGTTGTGTCATACAACTCAACCATTGTTTGCCTGCAGAATCA

CTGACAGTAATTTAGGTATACTAAAAATGGGCTGGGAGAAGAAGTAAAAATATATGTTTG

ACATATTATGGGGCCCTAGAAGAACTAGGCAGACTGTTTATTATG

>HLA34861 DOB*01:03:01:02

CTGAAGGGGGAGAAACCAGTGAAGTATGAGAGACGTAAAAGACAACTAAGCTGTGGCGTC

CAGGAACACATACCTGGGGGACTAGACTACAAAGACATGAAAGAGGTTACAATAAAATCA

GGATATGGTCACTTTCGAGGGGAAGGAGAGGCTTTGATTGGCCTGACGTACAGAGACTTC

CGCTGGAGCTGGCAAAGTCCTGTTCCTTGACTTGATTACAGGGGTTTTTCTTAAATAATT

CACTAAAATGTACATTTTTTGTATCTCCATTTTATTTGACAATAAAAGGTTTTTAAAAAC

AGTGAAAAGGAAACAGTGACTACCTGAGCATTTGTCTTCTGAAGACTGTGGAGACTGCAG

TTGGAAGACAGAAAGCTTTGGAGATCATGACTTATAGGAGTAGGGCTGGACCACAGAAAA

GTAAATGATTTGGGGCTGGAAGGAGTAAGGTCTCAGGGGAGTTTCTGGACAATGCCCTTG

GCAATGGGGATTAATGATGTACACGTAGAAGGGAGAAGGGCAGATGGGTGGGAGATGCAT

GATCTCAGAACACAGAGCTCCAGAATCAGTTCGGGCTCCTCCAAGGATCAGGGAAGAGAG

TTATTCCCAGAACATTGACCTCATGAATGTCCTTTACCTCACCCAGGGCCCAGACTACTC

ATCACTACTTCAGGCTCCAGAGAGAGAAGCTTCAGTGAGGACCTTAGCATGACTGAGGGA

GCAGAACAGCTCTTGAGACCTGGAGGCACAGTGATGAAGGTCTCAGGAGGCAGCCTCACC

ACCCCCCACAGCCGTTCCAGAGACTCAGGGGACAGTCCCATCCAGACAGCAGCAACCTTA

CTCCTCCCTACCCCCATGTCATCTCCCTCTGGCCAAAGAACCGGGAGAATGATCTGCCAC

TCAAAGACAAGGAAAAAGAGACATTACCTCATTACCAGACATCTGCGTCCTCACATATCC

TGGAAAGAAATCGAGAAAAGAATGGATTGCCCCAATTAGGACCCAATATGATTATCCCGA

GGGAAGAACAAATGGCTGGCAAGGTCAGCACTCTCTCTGCTTATCCCATTTCTAGCTTCA

GAAAAAAATTATCCCGGTGATCCCTGAGAGGCACAATCAGCTTTCCTTGCCTCAGATCAT

TGACGTTAGGGAAGGTGGGAGTGGGGAAGGTCTGGGACAGGTGGCAGGGCACTCCTCACA

GGCTCATTACCTTTCTGAGCCCTTAGCTGGATGATGATTCCCACCAGAAGGAAGATTAGC

CCAAGTAGGAAGGCTGCAATGCCACTCAGCATCTTTCTCCAAGAATATTCAGACTGAGCT

CCTATGGGAAACAGGTCTTTAAATTAGTAAAAATATCCCAATATTTAAAGCACTTTCTTG

GAATCCCAGAATCTGTACTAGACACCAAATCCAATGCTAGCTAGAGAAAAATAAATAAAT

TTAGAAAAGGTTCTTCGAAACCAAAGTTGGCACCCATGGAGTTACCCCCCATCGGTTACA

GATTCTCACAGCCCATAAGAATGCCTCCTAAATACTAAGACCAAAGAATTAGAGGACACC

AGTTCATAGGGTTGGAAGCACATAATGAGGTGATTAGATCTCCTCATTTCTTGGAAGATA

TGAGGATAGATATCTGCCATGTTTTCTCCCACCCTAACCCAAGGACTCTGGTTTCTGTGA

CTGTCCCAGATCAAGGGAAAGAATAATTCATGTTGTGACCAAGATAAACGCAGAAGTGAC

ACAGGCTCTGTATTGAGTCAGTATAGTCCTGAGTCAGGCCCAGAGAGTACTAGAAACTAA

TTCTCACTCCACTCCACAGAAACAGGGCTCAGCAGGCTGGAGTGATCGACAAGGCAGGTG

TAGACATGTCCAAGTTCAGGAGTCATTTCTAGCATCACCACAGTCTGAAAGGTCCAGTCT

CCATTCCTGATAGGGCCAGTGGACATGACCCCAGCTCTCTCCTCCTGCCCATTCAGGAAC

CACTTGATCTTGATATCCCCTGGATAGAAGCCTGTCACAGAGCAGTGCAGCAGATTATGC

TGGTGCAGGAGTGGGGTCCTCTCTGGGTACACTGTCACCTCTGGTTGCACTAGGAAGGGA

GGAAAAATGAGACACCGTGAAAGAAAACCACCAAGCTGGGACAGGAGATTCTTTAGGGAC

TATCACTATGTCTAATCTCTTTCCCAGATCACCCAAGTGAACACAAAGTATAGGCAAGTC

TCAGCCCCCAAGATCAGTAACAGGGTATGTCAATGCCTGTCAGGAGGATTTAGACTTTCT

GAGGTACTCCCACAATTACTGCTTCTCTTTGAGGGCACAATAGCCCTCGAAGTCCCTGAG

AACCTTGGGGGTCTGAGACCAAGATCACAGTGGCTGACTTGTGAGGATAATATATCACAG

CTGGGGCCAGAACATCTACACAGACAACCATTTATCCTAAAGCAGAAAATTGCTTGTAAG

AAAGAAGAGCCATGGCCAGGTTCACATGGGGGACATTCCTGAGCCCCGCCAGACCTCAGC

TTCCAGCTCACCTTTTCTCCCCACAGTGAAGGGTGCGCCCAGCCTGTAGTTGTGTCTACA

GACCCCATCCACGGCCTGTCTGCTCCTCTCCAAGAGATCCAGCCGGCTGTTCCACTGCTC

AGCATCTGGCTGCCCCAGCTTGGTCAATGCCACAAACATCCCCACATCACTGTCGAAACG

TACATACTCCTCCAAGTTAAAGATGAATCTGACCACAAACTGCACCTTTTCTGTCCCGTT

GGTGAAGTAACAGTCAGCCTTTGCCTGAATCACAAAATCTTCTGGAAAACCAAAACCAAA

ACCATGAACCAGCCCCCTCCTCTGGGAAAACCCATGCCTGGTAAATTACGTCAGACCACA

TGGATCTAAGAGGAGGCCTTTGACCTCAGTATGCTCAAAAAGCACAGTGTCAAGTGAGAA

AAGAAACAGAATGGGATTCAACAGAGAATGACATTTATTAAATTTTAAAAACACATAAAG

AGCAATAATGCTACATATTTCTAAAAGCCACTCTCATACTTAGAGACTTACCAGACACGT

TTAGAATGGATTCTCTAGGGAGGGGAGAGAATGAGAACGGAGGCAAGAGAAGAGACGAGA

GAGTCTTGCACTACTGCCAATAATTACAATGTGCTGTGAACTCATTGGGTAAAATTAAAC

CAATCCTATGCACTTAAGAACAACAACTACAATAAAAAGGAAATTCAAATGGAGTTAATA

ATGTAGGTAAGTCAGGAAGGACGTCCTGAAGACATTGCATCTAAGTCAAGACTTGAAAGA

TAATTGCTATTAATGTTGGGTTGTAATTTACTTTCCTTTCTAAGTTCAGAAGCCTCCTCC

AACTCTGAACTGAGCCATAAGAATGACCTTCCTGGGTGAACCCCACTTATCCCTCACTCA

GTAGCTAATTTCAGATGAAGTTCCAGCCTGTAATTTCTCAGCATGTATACTCTTCTCTAT

TTCCTCTAGTAGTCTAAACCAGGGGGGAAATCTGAATTTTTCATCATCATTTAACATCTG

TGCTGATTTTTTTTTCAGTTGTATTGTTTAATGGACATTATAAACTCAGGGCGGTTTCTA

TTCTCTGAGAATAATGATCTCTCCTGGCCAGGTTTGTTCCTCTTTTGTATTTAATGAAAC

TAACATGCAAAGGGGATTCTGTTCTTAGCACATTACATCCTGTTTCTGCTCATTAATATG

TGCTTTCATCTCACATTGCTTCATGGCTGCATATTCTGTCACCTGTGCTAGAAAAATAAC

AGTGACAAGTAACTTGTACCTGGTAGCCGGAGGACAAGGACAATGTATTCTATTCAACTT

CTCTTACTTCTCAAAACTGTTTAGTACAATTCTGACAATATAATAGTGGCTTAATAAATG

ACAGAAGGAGCAACCTTTGTTTCCAGTTTCATTTGTCCACATATACCCCAACTGAGATTT

GTTTCCGTGTCCTGACCAAAAAATCACAGATTGCCTCTGTGACCCAGCCTACTGCAGGTT

GTTTCTCCCAGCAGGCTCGAACCCAAGCCAAGGCCTTCAACACGCCCAGGCACTGACTGA

GGTTGATACACACAATAAGGATCCTAAACAAGACACAATGTTTCCCTCTTCCTGCCTCCC

TACCCCTTGAATAGGTGGCTCTGGTATATGAAGTCCATCCCATGTAAAGAGGCAAGGCGT

ACCTTTCTGCCAAAGGGAAGAACACTGTTCTTTGAAACCAAAAGCCACTTCCAGTCTGGT

CTGTGGCCTGGACTTACAAAGAAAGGCATCACTCCCCCATGCCAATTCTTGCATACACAC

TGGAAAAAAACAATTGCTCTGTTCTTACCTGGAGAGTCTGTGCCTTGAGTCATGGAGGAA

TCCAGTCGGGTCAGATTCACTAGCAGAGCCACCACCCAGGGGACCCACCCAGAACCCATT

CTGGAGAAAGGAAAAAAATGAGATAGTAAAATCGTCAGCCTCTTCAGAATGAGCTCATAA

AATTCAGTCAGAAAGTACCCATTAAGAGTATAAATCGCTGTTTTTCTGGCTTCCCCAGGA

TTGGAAACTCCTCAGATTGACAACCAATCAAGATAGAAGAGTTTTGCATCATCAGGTACT

GGGTAGGATACTTTCACAAAGTTGTGTCATACAACTCAACCATTGTTTGCCTGCAGAATC

ACTGACAGTAATTTAGGTATACTAAAAATGGGCTGGGAGAAGAAGTAAAAATATATGTTT

GACATATTATGGGGCCCTAGAAGAACTAGGCAGACTGTTTATTATG

>HLA:HLA06460 DOB*01:05

CATAATAAACAGTCTGCCTAGTTCTTCTAGGGCCCCATAATATGTCAAACATATATTTTT

ACTTCTTCTCCCAGCCCATTTTTAGTATACCTAAATTACTGTCAGTGATTCTGCAGGCAA

ACAATGGTTGAGTTGTATGACACAACTTTGTGAAAGTATCCTACCCAGTACCTGATGATG

CAAAACTCTTCTATCTTGATTGGTTGTCAATCTGAGGAGTTTCCAATCCTGGGGAAGCCA

GAAAAACAGCGATTTATACTCTTAATGGGTACTTTCTGACTGAATTTTATGAACTCATTC

TGAAGAGGCTGACGATTTTACTGTCTCATTTTTTTCCTTTCTCCAGAATGGGTTCTGGGT

GGGTCCCCTGGGTGGTGGCTCTGCTAGTGAATCTGACCCGACTGGATTCCTCCATGACTC

AAGGCACAGACTCTCCAGGTAAGAACAGAGCAATTATTTTTTTCCAGTGTGTATGCAAGA

ATTGGCATGGGGGAGTGATGCCTTTCTTTGTAAGTCCAGGCCACAGACCAGACTGGAAGT

GGCTTTTGGTTTCAAAGAACAGTGTTCTTCCCTTTGGCAGAAAGGTACGCCTTGCCTCTT

TACATGGGATGGACTTCATATACCAGAGCCACCTATTCAAGGGGTAGGGAGGCAGGAAGA

GGGAAACATTGTGTCTTGTTTAGGATCCTTATTGTGTGTATCAACCTCAGTCAGTGCCTG

GGCGTGTTGAAGGCCTTGGCTTGGGTTCGAGCCTGCTGGGAGAAACAACCTGCAGTAGGC

TGGGTCACAGAGGCAATCTGTGATTTTTTGGTCAGGACACGGAAACAAATCTCAGTTGGG

GTATATGTGGACAAATGAAACTGGAAACAAAGGTTGCTCCTTCTGTCATTTATTAAGCCA

CTATTATATTGTCAGAATTGTACTAAACAGTTTTGAGAAGTAAGAGAAGTTGAATAGAAT

ACATTGTCCTTGTCCTCCGGCTACCAGGTACAAGTTACTTGTAACTGTTATTTTTCTAGC

ACAGGTGACAGAATATGCAGCCATGAAGCAATGTGAGATGAAAGCACATATTAATGAGCA

GAAACAGGATGTAATGTGCTAAGAACAGAATCCCCTTTGCATGTTAGTTTCATTAAATAC

AAAAGAGGAACAAACCTGGCCAGGAGAGATCATTATTCTTAGAGAATAGAAACCGCCCTG

AGTTTATAATGTCCATTAAACAATACAACTGAAAAAAAAATCAGCACAGATGTTAAATGA

TGATGAAAAATTCAGATTTCCCCCCTGGTTTAGACTACTAGAGGAAATAGAGAAGAGTAT

ACATGCTGAGAAATTACAGGCTGGAACTTCATCTGAAATTAGCTACTGAGTGAGGGATAA

GTGGGGTTCACCCAGGAAGGTCATTCTTATGGCTCAGTTCAGAGTTGGAGGAGGCTTCTG

AACTTAGAAAGGAAAGTAAATTACAACCCAACATTAATAGCAATTATCTTTCAAGTCTTG

ACTTAGATGCAATGTCTTCAGGACGTCCTTCCTGACTTACCTACATTATTAACTCCATTT

GAATTTCCTTTTTATTGTAGTTGTTGTTCTTAAGTGCATAGGATTGGTTTAATTTTACCC

AATGAGTTCACAGCACATTGTAATTATTGGCAGTAGTGCAAGACTCTCTCGTCTCTTCTC

TTGCCTCCGTTCTCATTCTCTCCCCTCCCTAGAGAATCCATTCTAAACGTGTCTGGTATG

TCTCTAAGTATGAGAGTGGCTTTTAGAAATATGTAGCATTATTGCTCTTTATGTGTTTTT

AAAATTTAATAAATGTCATTCTCTGTTGAATCCCATTCTGTTTCTTTTCTCACTTGACAC

TGTGCTTTTTGAGCATACTGAGGTCAAAGGCCTCCTCTTAGATCCATGTGGTCTGACGTA

ATTTACCAGGCATGGGTTTTCCCAGAGGAGGGGGCTGGTTCATGGTTTTGGTTTTGGTTT

TCCAGAAGATTTTGTGATTCAGGCAAAGGCTGACTGTTACTTCACCAACGGGACAGAAAA

GGTGCAGTTTGTGGTCAGATTCATCTTTAACTTGGAGGAGTATGTACGTTTCGACAGTGA

TGTGGGGATGTTTGTGGCATTGACCAAGCTGGGGCAGCCAGATGCTGAGCAGTGGAACAG

CCGGCTGGATCTCTTGGAGAGGAGCAGACAGGCCGTGGATGGGGTCTGTAGACACAACTA

CAGGCTGGGCGCACCCTTCACTGTGGGGAGAAAAGGTGAGCTGGAAGCTGAGGTCTGGCG

GGGCTCAGGAATGTCCCCCATGTGAACCTGGCCATGGCTCTTCTTTCTTACAAGCAATTT

TCTGCTTTAGGATAAATGGTTGTCTGTGTAGATGTTCTGGCCCCAGCTGTGATATATTAT

CCTCACAAGTCAGCCACTGTGATCTTGGTCTCAGACCCCCAAGGTTCTCAGGGACTTCGA

GGGCTATTGTACCCTCAAAGAGAAGCAGTAATTATGGGAGTACCTCAGAAAGTCTAAATC

CTCCTGACAGGCATTGACATACCCTGTTACTGATCTTGGGGGCTGAGACTTGCCTATACT

TTGTGTTCACTTGGGTGATCTGGGAAAGAGATTAGACATAGTGATAGTCCCTAAAGAATC

TCCTGTCCCAGCTTGGTGGTTTTCTTTCACGGTGTCTCATTTTTCCTCTCTTCCTAGTGC

AACCAGAGGTGACAGTGTACCCAGAGAGGACCCCACTCCTGCACCAGCATAATCTGCTGC

ACTGCTCTGTGACAGGCTTCTATCCAGGGGATATCAAGATCAAGTGGTTCCTGAATGGGC

AGGAGGAGAGAGCTGGGGTCATGTCCACTGGCCCTATCAGGAATGGAGACTGGACCTTTC

AGACTGTGGTGATGCTAGAAATGACTCCTGAACTTGGACATGTCTACACCTGCCTTGTCG

ATCACTCCAGCCTGCTGAGCCCTATTTCTGTGGAGTGGAGTGAGAATTAGTTTCTAGTAC

TCTCTGGGCCTGACTCAGGACTATACTGACTCAATACAGAGCCTGTGTCACTTCTGCGTT

TATCTTGGTCACAACATGAATTATTCTTTCCCTTGATCTGGGACAGTCACAGAAACCAGA

GTCCTTGGGTTAGGGTGGGAGAAAACATGGCAGATATCTATCCTCATATCTTCCAAGAAA

TGAGGAGATCTAATCACCTCATTATGTGCTTCCAACCCTATGAACTGGTGTCCTCTAATT

CTTTGGTCTTAGTATTTAGGAGGCATTCTTATGGGCTGTGAGAATCTGTAACCGATGGGT

GGTAACTCCATGGGTGCCAACTTTGGTTTCGAAGAACCTTTTCTAAATTTATTTATTTTT

CTCTAGCTAGCATTGGATTTGGTGTCTAGTACAGATTCTGGGATTCCAAGAAAGTGCTTT

AAATATTGGGATATTTTTACTAATTTAAAGACCTGTTTCCCATAGGAGCTCAGTCTGAAT

ATTCTTGGAGAAAGATGCTGAGTGGCATTGCAGCCTTCCTACTTGGGCTAATCTTCCTTC

TGGTGGGAATCGTCATCCAGCTAAGGGCTCAGAAAGGTAATGAGCCTGTGAGGAGTGCCC

TGCCACCTGTCCCAGACCTTCCCCACTCCCACCTTCCCTAACGTCAATGATCTGAGGCAA

GGAAAGCTGATTGTGCCTCTCAGGGATCACCGGGATAATTTTTTTCTGAAGCTAGAAATG

GGATAAGCAGAGAGAGTGCTGACCTTGCCAGCCATTTGTTCTTCCCTCGGGATAATCATA

TTGGGTCCTAATTGGGGCAATCCATTCTTTTCTCGATTTCTTTCCAGGATATGTGAGGAC

GCAGATGTCTGGTAATGAGGTAATGTCTCTTTTTCCTTGTCTTTGAGTGGCAGATCATTC

TCCCGGTTCTTTGGCCAGAGGGAGATGACATGGGGGTAGGGAGGAGTAAGGTTGCTGCTG

TCTGGATGGGACTGTCCCCTGAGTCTCTGGAACGGCTGTGGGGGGTGGTGAGGCTGCCTC

CTGAGACCTTCATCACTGTGCCTCCAGGTCTCAAGAGCTGTTCTGCTCCCTCAGTCATGC

TAAGGTCCTCACTGAAGCTTCTCTCTCTGGAGCCTGAAGTAGTGATGAGTAGTCTGGGCC

CTGGGTGAGGTAAAGGACATTCATGAGGTCAATGTTCTGGGAATAACTCTCTTCCCTGAT

CCTTGGAGGAGCCCGAACTGATTCTGGAGCTCTGTGTTCTGAGATCATGCATCTCCCACC

CATCTGCCCTTCTCCCTTCTACGTGTACATCATTAATCCCCATTGCCAAGGGCATTGTCC

AGAAACTCCCCTGAGACCTTACTCCTTCCAGCCCCAAATCATTTACTTTTCTGTGGTCCA

GCCCTACTCCTATAAGTCATGATCTCCAAAGCTTTCTGTCTTCCAACTGCAGTCTCCACA

GTCTTCAGAAGACAAATGCTCAGGTAGTCACTGTTTCCTTTTCACTGTTTTTAAAAACCT

TTTATTGTCAAATAAAATGGAGATACAAAAAATGTACATTTTAGTGAATTATTTAAGAAA

AACCCCTGTAATCAAGTCAAGGAACAGGACTTTGCCAGCTCCAGCAGAAGTCTCTGTACG

TCAGGCCAATCAAAGCCTCTCCTTCCCCTCGAAAGTGACCATATCCTGATTTTATTGTAA

CCTCTTTCATGTCTTTGTAGTCTAGTCCCCCAGGTATGTGTTCCTGGACGCCACAGCTTA

GTTGTCTTTTACGTCTCTTATACTTCACTGGTTTCTCCCCCATCAG

>HLA:HLA00499 DPA1*01:03:01:01

GGTGGACCTGAAAGAAAGATTAATTATATATTTTATTAGATAGCACATTCATTCTTAATT

ATCAGCAGAATATTTTTGTAAAATATTGACAACATAACATAAAACATGATTTAAAATATA

GTGACCAAAAAACATCGAAGATCATCTGACTGTCTGGGATGGGGGTGGGGCTCGGGAGAA

GGAACATAATGGAAAACATTTGCCGTCAGAAGAAGGTGTAACTTATCTTTTTACATCTCT

TTCTCTAACTCTGAAAATGAACTGTGAACTGGAGCTCTCTTGACCACGCTGGTACCTAAA

ATTCTCCCATCTCTTCCCCAGCACCTTCCAGCGTCCTCTTTACCCAGCAACAGAGAATGT

CAGCTCTATGATTTCTCTGATAGGTGAATCCCAGCCATGCTGATTCCTCTCCACCCATTT

CCAGTGCTAGAGGCCCACAGTTTCAGTCTCATCTGCCTCCACTCGGCCTCAGTTCCTCAT

CACTGTTCCTGTGCTCACAGTCATCAATTATAGACCCCACAACATGCGCCCTGAAGACAG

AATGTTCCATATCAGAGCTGTGATCTTGAGAGCCCTCTCCTTGGCTTTCCTGCTGAGTCT

CCGAGGAGCTGGGGCCATCAAGGGTGAGTGCTCAGGAGGACGCAGGAGCGTCGGGGTGAG

TGATGGGGTGGTTCACATCAATTGCTGCTTCAGGGATCACAGATTTTAGGGGCTCATTGA

TCTATCTGGTCCTCATAGTCTATGTTCCCTCTGGCCCTCATAATAATAACAGCAATAACA

GCCAGAATTTATGAGACTCCTGCATAGTTTCTTTCCCCATTTACATCTCACAGGAATCTT

CAATGAAGATAATATTCCATTCATTTAGAAATTATTCCTTTTATTTAGAAATTATTTTGA

AAAAACTGAAGCTCAAAAAGATGAATAAGTTTTCCAAGGTTACACAGCAGATCAACGAGC

CAAGTTTGAAGTCCAGACCCAGCTCTGAGGGTCATACACTGCCTTCCCCAGATTCCTGCA

CACAGTGACCTACTATCAGGGCCCTCCTATCTCTCTGGGATCCCCAGCCTCTATCTTTTG

TGGCTGCTTTACAGGAACTCCGAGCTATGGACTCTGCATTAGGAGACGAAGTGCAAAGAG

TGTTTCTGTATCCTCCCTCTCTTCTAGGACCCTAGGGCTCTTCCTGGGTCTTTGTGGGTG

GTCACAAGCTTTCCTCTCTCAAGACAGCAGGGTTGCATGGTCTTGATAGCCTTGTGATTC

GGGTTCTGAGAGATTCAGGACTGCAAGGGAGGCCTAGACTTTTGATAGCTGCAAGGACTC

AGCCAGAGATGGACCGTAGTGAATGCTCCTTTTTCCTGTAGCTGAAATCAGGGAGAATGA

CATCAAGCCTGTGCATGATGCTGTCATTCCAAAATCTAGTGATGGGGAAGGTTAGAATCC

ATAACGTACAAGATGCACACTGGCTTCAGACAGTTTTATTTAAGATGTGTAGAATAAAGA

GGAGGTCAGGCTGGGTAGAACCAGAAGTATCTATTGCCCTGTTCGCGGTCACCTGAGTTA

TTTCTAATGTTATGTTATAATAAACACCACAATAGGCTTCTCTTCATAGATGCAAATACT

TTTTAGTATTCTTGGTAGAAATTCCTAATGAGCTCAGCTGTCTCTTCAGGGCTTCCCTGC

CCAGTCTCTTAACATTTAAACATGTCATTTACCTTAAAAACATAAGTGCAAACCAACTGA

TAAAAAACAACCTTGCCTTCAGTCTGCATCCTGTCCCAGAGACACTTTCTTTGTGTCCTC

ACACGTGGAGCTAAGCTTCTGACTTGTCTCTGGTACATCCCTGAGGATCCTCTCATCTTG

GCCATCAGGAACCTCTACAGAAGGTCAAATTCAGTGGGTTCTTCTCAGTGCCTCTGACTT

GAGTTACTAATAACATTTGCACTATAATCCACTTCTTTCTGATGAACTACCCTGTCCTTA

TTTTTCTCCTGTTTACCTGGATCCTCCTTATCATCTTTTAAACCACCTCTTAACTATCAT

GTTCTCTCATTATACCCTGAGATCTCGGCAATTCTGATTTTTGGCACTCTTCCTGGAAAA

TCTTATTTAACCTGCACCTGCCACTAATGACTCTCAGTTCTATGGCCTAAATTCCTCTCC

TGAGACCACCCATAATCCACAAATATCTATGTATTATTTCTCCTTAGATGACTTTCAGGT

CTTCTAAGTGCAATAGCCCCACAGTGAACTCAGTATCTTCTCCCGGTCAGGCTGTCTTCC

CTGAGAGAAGTGGCTTTTGCCCTGTTTTCTGAATGCCTACATTGAAGCCATCTGTTCCCC

AGGAAGCCTTCCCTGATGTGCTGTTTGGTCGCATCTTGTGTATACCTACGTATCTGCACT

TATCCTTCTGAACCTGCTGTTGTCCTGTCACTTGTGTTTCCTTCTGTGACTTATACGCGT

CTGCAGAACAGGACGTATGTATTATTTTTATTTGGGTATTTAGCATCTAACAGTGTTTGA

CATATAGTAGTCTTTTAATACATATTTTTGTCTGAATGGAAATGATATTTTGAAGAAAAA

TAATCTGTTCCATAGCTGGCTGATCTTTGGACTGCAGAACTTGTGAAAGTGTTTTTTAAA

AAGCATTTTAAAAAGTACAAGGGACATTCATGTATTAAGAAGATGAGTTTCCAATAACTG

CTAGAGGACTTTGTGTCTTTTTATTTTACCCTCTTTTTCCTGATGAGTCCTTTGAGTCCT

TTAAACTGAGGAGCAAGCTAAGTTTCCTAGTGAAATACCTATAGGATTTGTTTTGTTTAG

TTTCAAATACCACTCTTTGCTTGGCCACTTACTGTGTCAGGGAGTCATTCTCAGTGAAAA

ATAAGACACAGGTCATACCCTCTAGACACTTACAATTACAGTGGCAAGGAGTCATTCTCC

TGTCACTGTAAGTGGCCAAGCACAGACTGGGTCCCCACATGTCAGGGCTGAAAACTCACA

GGGAAATCTGTGAGTTGGGAGGTGAGAGCAGAAGAGTCCCGTAGTTCCTTCTCACTCTGA

TGCATTTATCATTCTAAACCCAGACTTTCACATACACATTCATCGTTTTCTTTCATGATA

ATAGTTGCTTTTATCCTCTTATCTTTGCTAATTCTTACAAACTAATAAAGACTAAGAAAC

AAAATAAATTAAATCCTACAGGTGTTCCAAACTCAGCAATAATTTCTAGTTGGCCTCTAA

AACAAAAATCAAAATATAAATGTAAGAAAAGTTTAGAATGCTTAGTACCTGTGTGATGAA

ATAATCTGTACACTAAACCCCCAAGTCATGAGTTTACCTATACAACAAACCTGCACATGT

ACTCCTGAACATAAAATAAATGTTGAAATATTTTTAAAAAGGAAACAAAAGTTTGGAACA

AATGCCAAAATAACTGTACTGTACTTTTGAATTTATATGCCCCAAATGAAAAATATTATC

AACAAAGCTATACATTCTACAGTTTCATGTTCATAAACTAAGACAGAAACTTTAAAACTG

TCAAGAGCCCTAAAATTTGAAGGATATTTTCTTCTTCCTCTCAATTTTGTATTTTTTTCT

ACCTTTTCTATAATAAGAAAAAGAAAATGTCCATTCCCCCACCCCCATGACTCTAAAAAC

AATTTTACATCTGTGTCATAGAAAAATTAAGATCTTAATGGGAGAGAAAACCTCTCTACT

AGTTCCGCCAGTAGCCGTATGACCTTAGCAAGTTATTAATATGTAACTTCCCTGCATTTC

CTTACCTGTAAAATGTATGATATGTATTTGCTTCATAGGGTTATTGTGACAATTCAGCGA

CTGAAATATGTAAAGTATTTAGAAGGATGCCTGGCACAAGTAAGTGCTCAACAAATGTTA

GCTGTCATTGTTACTATTACTATTGTGTAGGGTCAGGATGCCCAGACTTTCAAAGACCAG

GAAGCAGCTTGACTTATCAGTGATAAACTTTTCATTTTGTTCTTTGCTCCTTTCTTTTTA

TAACTGCTCATCTGCTCTGTATTATTTCCTTTATGGTGTTGCTCCTTCTTCTTCCCCATA

TGTCCTTCCTTTGACCTCTTACCTTCTTCCTTTTTATATTCATAAGTCTTTATTCATTCT

CTAGCTTTGACCACTTGCATATTCAAACTGACATTTTGTCGTGTTTTTCTCTACTGTCTT

TATGCAGCGGACCATGTGTCAACTTATGCCGCGTTTGTACAGACGCATAGACCAACAGGG

GAGTTTATGTTTGAATTTGATGAAGATGAGATGTTCTATGTGGATCTGGACAAGAAGGAG

ACCGTCTGGCATCTGGAGGAGTTTGGCCAAGCCTTTTCCTTTGAGGCTCAGGGCGGGCTG

GCTAACATTGCTATATTGAACAACAACTTGAATACCTTGATCCAGCGTTCCAACCACACT

CAGGCCACCAACGGTACGCCCTATCTTTGCCTCTTCCTCTGTAGCCCAACTGGAAGGGAT

GAGAGGGCCTCTCTGCCACCCTCAGACTAGGAAGCCTAAGTGCCCCCTGCTGTGTGATCC

TCTTCCCCTAGTGGCCATGGGCTGATCCCACTACAGCAAGGGCTTGCATCCTCTCTTCTC

AGGAGAGAGAAAGGTGAGCAGAGTGAGGCTGGTCAGTGGTGTGATACCCCTGTCTGTGAT

TCAGAGCTGCCATAAAATCTAAGGCTGAGGTAGAGGACCACCCTCCCCTAAGAGGTGGAG

CCTTTGTGATTCATCCCAGAAGAGGGGCCTAACCTGGTGCTGTCTCCTTCCAGATCCCCC

TGAGGTGACCGTGTTTCCCAAGGAGCCTGTGGAGCTGGGCCAGCCCAACACCCTCATCTG

CCACATTGACAAGTTCTTCCCACCAGTGCTCAACGTCACGTGGCTGTGCAACGGGGAGCT

GGTCACTGAGGGTGTCGCTGAGAGCCTCTTCCTGCCCAGAACAGATTACAGCTTCCACAA

GTTCCATTACCTGACCTTTGTGCCCTCAGCAGAGGACTTCTATGACTGCAGGGTGGAGCA

CTGGGGCTTGGACCAGCCGCTCCTCAAGCACTGGGGTATGCAACTGCTTTTCTCTCCATA

ATCTCCTGGCATCCTCTATTCCAAAGACCTGGTGTCCTCTGCACCAGCTTTCCGCACTGG

CTGGGTCTCAGTCCTCTCCTCGTCCTAACATCCAATTAACTGGTCCATAACCTTCAATTC

CCACAACCATCCCAGGCCATCACCACCCTCACTGCACCTCCTGACCCTATCTCTTCATTC

TTCCCCCAGAGGCCCAAGAGCCAATCCAGATGCCTGAGACAACGGAGACTGTGCTCTGTG

CCCTGGGCCTGGTGCTGGGCCTAGTCGGCATCATCGTGGGCACCGTCCTCATCATAAAGT

CTCTGCGTTCTGGCCATGACCCCCGGGCCCAGGGGACCCTGTGAAATACTGTAAAGGTGG

GAATGTAAAGAGGAGGCCCTAGGATTTGTAGAATGTAAGGAAGGGAGGAAAAATTCAATC

TGATAAGTGTTCATTGATCTTCTAATGGGTTAAAAGCATTCAGCCACATAACAACAACAA

CACCGATAACTAACTGAGTAGTTAATATGGTCAGGCGCTATTCTGAGGATTTACATTTAT

TAACTCACTTTATTCTCACACATAGTCTTTGAGGTAGGTACTATTATTTTCACTATTTCA

CATGAGAGATACTTACATCTTTTTACATACACAGAGACTTTAAGCACTTTGATCAAGTTC

CCACAGCTATGAAGTAGTAGGGCTAGCTTCCAATCCAGAAAGTCTGGATCCAAGACTGTT

TATCCACTGTCCTATTCACCCTATTTTGTGAAGGAAAAGACCAAGTTCAAATTCTCCAGA

GTCCATTGCCAAATAATGGAGTCAGATCTATATTTCTATACATAATTACAACACAGTGTG

GTGGGTGCCTGTAACTACTTACTGTCTCTACTTGGACTCATTCCATGGCAATGTTCACAC

AAAAAATGCCCCTCCAGAGATCTTACAGGTTTCTATTTATCATAACACTCACCATGCTTT

ATATTTTTATATGTTTTGGGAATTCTCTTAGCATTAGACAGTGAACTTCCATGCAGATGA

CCACATCTAATTCATTATTATTATTGTTATTCATGCTGGACCTCAGGTACAAAAGGTTAA

GAACTTCTCAGTTCATTATATGATCATCATTGGTGCCTCCGAGCTCTCTCTCTCTCCCTT

GATTTATTTGGTCCCTTTTATCTCCAGTCCTTACTCCCATATCTAACCTCTTACCCCTAC

CTCATAGGTAAACATTTTAATGAATTTGATGTTTCCTTTTATTTGCATAGATCCTCTGTA

ATATGTAGTAGTGTCCAGTGTACATGTATTTTTAATTAACCAAAATGGCATTAAATTATA

GATCTAATTTTGTACATCCAGTTTGTTTCTTCCAAATCTTCCATAGTATTTTACTTTATA

TGTCCATGCATTAGTCCATTTTGCATTGCTATAAAGGAATATCTGAAGTTACCTAATTTA

CGAAGAAAAGAGCTTTAAATGGCTCACAGATCTGCAGGCTGTACGCGAAACATGGCACTA

GCATCTGCTTCTGTTGGGGGATTCTGGAAGCTTTTACTCATGGTGGAAGGCAAGTGGAGC

CAGTGCATCACATGGTCATAGAGGGAGAAAGAGACATAGAAAGAGGTGCCAGCCTCTTTT

TAACAACCAGGTTTCATGTGCACTAATAGAGTGAGAACTCACTCATTACCCGGAGAGGGG

ACAAAGCCATTCATGAGGGTCTCCTCCATGATTCAAATACCTCCCACCAGGCCCCACCTG

CAACACTGGGGATCAATTTTCAACATGAGACTTGGAAGTGACAAATATCCAAATCATATT

AATCCACATATCTACATTGCTCCTGGGATACCTGGATCATTCCTGGTTCTCTACTATTGC

AAGCAATGCTTGTATCTCACATGGAACTGCATATACATGTGGGCCTGACCTGCATCCCTG

GAATGTATGTATCCTAGAAAGGGGTTGCAGGGTTGCTGGAGATGCAGCTCCTTAATTTGA

CTAAACACTGCTCATCTTCTCATCAGAATGGCTGTACTCATCTGAACTTCCTTTGTCAGT

ACTCTAATTGTCCTGCAACTCCTAAATGGACTTCAACACTGGACATTATCCAGTTTTCTA

ACTTTTGCCAATTTCATGTGCATAAAGAAATATGCTGTTTTATTTTGCATTTCTTTAATT

ACTAATAATTGGGGTTATAATTAGGACTGATTAGCCACTTGGGGGTTCCTTTTCTATAAA

TTGCCTGTTCACATTCATTGTCCATTTTTGTACTATGTGCTTCCATCATTTTCTTATTGA

TTTGCAGGTGATCCTTATATAGTCCTGCTAGTAGTCCCTTGTCAGTTTTAGGCATTGCAA

ATGTTTTCCTCTAATCTGACTTCTGGCAACTGTCTCCTTGGTTTCCTTTATTGAAGAGAA

ATCCTTAATATTTTGTAATGAAGTCCATCAACTGTATTTTTGTTTGTGTGTCTTTTTTAA

AAGAAGTCTTCCCTATACTGAGATATCAAAGATACTCTTAAAACATCTCCTACAGTTTTA

AATTTCACATTTACTACTTTAATTCATCTGGGATTCATCTTTGTGTTTGATGGGGATCAT

GTTTTATTTTTCTTTATATAATGGGCCAGTGTGTTCCCACAACTACTAAATAGTTCACCT

TTTCCCCATAGGTTAGTAGTGTCTCCTTTGCTATACTGAAAGCTCCCATTATAGGTGGGC

CTGTGTCTGAGTTCCATCTTGTTCCACTGTTCTGTTTGTCTCTTCTTGTGCCAGTGTCCT

AGTATTTTGATTACTATGACATTGTAGTGTGTGTTAGTATCCAGTAGGACAAATTCTTGT

TTATTTTTCTTAGTTCACACACATTTATAATTATATCTATAATGATTTGTAACAGAGTGA

AGTGAATGTAGAATGTCAGATGTTAAGAGGAAGAATGGAAAAGAGGGCTGGGACTAGGGT

GATGTAGGGGATGCACTTGGCTTAGGTGCAAAATTTGGGGGATACCAAAAGAACTCAGTA

ATAAATCATATTTTAATGAAATATCTTGAAAAGGCAAAATTAATGCAAAGATACATGATT

AACAAAACATCCAAAGAGGAGTATTTAACAAAAATGGAGAAGCAGAGAAGCAGAAGAATT

AGGAGAATATGCTGTCACATGAGCCAAGGAATTAAAGAATTCAGGAAGGAGGAAGTACTG

CTGTCAGATGTTCAACAGAGGTCATTTTAGAAAATTTACCTTGGTTTTTGAAATCCTTTC

AAAGAGCAGTATACACAATGTGAGCAAGTATCCTTCGTTCATTGCCGTCATTGATATGGT

TTGGATATTTGTCCCTTCCAATTCTCATTCCAGGGTTAAGCTTCTTCTCTGCCCTCAGTA

ATGTGGCCCTTCCCCTTGTCTGTATATTTTGGAGACATGAAGCATGTGGGATGGCCTCAC

AGTCAGCTGGGGTTTGAGGGTGAAATTCAATGACTTTCGTGAACTCCTTGGCTCCTATGT

GCTCTTCACCTGGAGGGCCAGGGCATGTGCAGGGATGACCACCTTCTCCCTGGGACCTGA

ACAGGGCAGAGAAATGGGAAGCTCGGGTGCAAAGGGAGTGGGGAAGATGGGTCCGGGCTT

ACAGTACTGAACCCAGGAATGACAATAACTGTGTGTGTTGCTGCAGGTGACAAAATATCT

GAACAGAAGAGGACTTAGGAGAGATCTGAACTCCAGCTGCCCTACAAACTCCATCTCAGC

TTTTCTTCTCACTTCATGTGAAAACTACTCCAGTGGCTGACTGAATTGCTGACCCTTCAA

GCTCTGTCCTTATCCATTACCTCAAAGCAGTCATTCCTTAGTAAAGTTTCCAACAAATAG

AAATTAATGACACTTTGGTAGCACTAATATGGAGATTATCCTTTCATTGAGCCTTTTATC

CTCTGTTCTCCTTTGAAGAGCCCCTCACTGTCACCTTCCCGAGAATACCCTAAGACCAAT

AAATACTTCAGTATTTCAGAGCGGGGAGACTCTGAGTCATTCTTACTGGAAGTCTAGGAC

CAGGTCACATGTGAATACTATTTCTTGAAGGTGTGGTTTCAACCTCTGTTGCCGATGTGG

TTACTAAAGGTTCTGATCCCACTTGAACGGAAAGGTCTGAGGATATTGATTCAGTCCTGG

GTTTTTCCCTAACTACAGGATAGGGTGGGGTAGAGAAAGGATATTTGGGGGAAATTTTAC

TTGGATGAAGATTTTCTTGGATGTAGTTTGAAGACTGCAGTGTTTGAAGTCTCTGAGGGA

AGAGATTTGGTCTGTCTGGATCAAGATTTCAGGCAGATTAGGATTCCATTCACAGCCCCT

GAGCTTCCTTCCCAAGGCTGTATTGTAATTATAGCAATATTTCATGGAGGATTTTTCTAC

ATGATAAACTAAGAGCCAAGAAATAAATTTTTTAAAATGCCCTAATTCATTGCAATTTTT

ACCAGCCATAGTCACTCCATGTGGGAGAACTTAAATCATGATTACCAGAGCTTTCAAAGG

TTTGAGAATAGTGATGATTATGAAGAAAAATATCTTATTTGAGCAAGGATTTTGTTTCTT

TATGAGTGTTCATTAGATATTACGATGAAAAAAGCATGAAATGGTAAAAATTCAGATAAA

TATAAAAACATGTTCTCTAGTTTTTTTTAAGTTAAAAAAGGAATTGTTTAAAGTA

>HLA:HLA06604 DPA1*01:03:01:02

GGTGGACCTGAAAGAAAGATTAATTATATATTTTATTAGATAGCACATTCATTCTTAATT

ATCAGCAGAATATTTTTGTAAAATATTGACAACATAACATAAAACATGATTTAAAATATA

GTGACCAAAAAACATCAAAGATCATCTGACTGTCTGGGATGGGGGTGGGGCTCGGGAGAA

GGAACATAATGGAAAACATTTGCCGTCAGAAGAAGGTGTAACTTATCTTTTTACATCTCT

TTCTCTAACTCTGAAAATGAACTGTGAACTGGAGCTCTCTTGACCACGCTGGTACCTAAA

ATTCTCCCATCTCTTCCCCAGCACCTTCCAGCGTCCTCTTTACCCAGCAACAGAGAATGT

CAGCTCTATGATTTCTCTGATAGGTGAATCCCAGCCATGCTGATTCCTCTCCACCCATTT

CCAGTGCTAGAGGCCCACAGTTTCAGTCTCATCTGCCTCCACTCGGCCTCAGTTCCTCAT

CACTGTTCCTGTGCTCACAGTCATCAATTATAGACCCCACAACATGCGCCCTGAAGACAG

AATGTTCCATATCAGAGCTGTGATCTTGAGAGCCCTCTCCTTGGCTTTCCTGCTGAGTCT

CCGAGGAGCTGGGGCCATCAAGGGTGAGTGCTCAGGAGGACGCAGGAGCGTCGGGGTGAG

TGATGGGGTGGTTCACATCAATTGCTGCTTCAGGGATCACAGATTTTAGGGGCTCATTGA

TCTATCTGGTCCTCATAGTCTATGTTCCCTCTGGCCCTCATAATAATAACAGCAATAACA

GCCAGAATTTATGAGACTCCTGCATAGTTTCTTTCCCCATTTACATCTCACAGGAATCTT

CAATGAAGATAATATTCCATTCATTTAGAAATTATTCCTTTTATTTAGAAATTATTTTGA

AAAAACTGAAGCTCAAAAAGATGAATAAGTTTTCCAAGGTTACACAGCAGATCAACGAGC

CAAGTTTGAAGTCCAGACCCAGCTCTGAGGGTCATACACTGCCTTCCCCAGATTCCTGCA

CACAGTGACCTACTATCAGGGCCCTCCTATCTCTCTGGGATCCCCAGCCTCTATCTTTTG

TGGCTGCTTTACAGGAACTCCGAGCTATGGACTCTGCATTAGGAGACGAAGTGCAAAGAG

TGTTTCTGTATCCTCCCTCTCTTCTAGGACCCTAGGGCTCTTCCTGGGTCTTTGTGGGTG

GTCACAAGCTTTCCTCTCTCAAGACAGCAGGGTTGCATGGTCTTGATAGCCTTGTGATTC

GGGTTCTGAGAGATTCAGGACTGCAAGGGAGGCCTAGACTTTTGATAGCTGCAAGGACTC

AGCCAGAGATGGACCGTAGTGAATGCTCCTTTTTCCTGTAGCTGAAATCAGGGAGAATGA

CATCAAGCCTGTGCATGATGCTGTCATTCCAAAATCTAGTGATGGGGAAGGTTAGAATCC

ATAACGTACAAGATGCACACTGGCTTCAGACAGTTTTATTTAAGATGTGTAGAATAAAGA

GGAGGTCAGGCTGGGTAGAACCAGAAGTATCTATTGCCCTGTTCGCGGTCACCTGAGTTA

TTTCTAATGTTATGTTATAATAAACACCACAATAGGCTTCTCTTCATAGATGCAAATACT

TTTTAGTATTCTTGGTAGAAATTCCTAATGAGCTCAGCTGTCTCTTCAGGGCTTCCCTGC

CCAGTCTCTTAACATTTAAACATGTCATTTACCTTAAAAACATAAGTGCAAACCAACTGA

TAAAAAACAACCTTGCCTTCAGTCTGCATCCTGTCCCAGAGACACTTTCTTTGTGTCCTC

ACACGTGGAGCTAAGCTTCTGACTTGTCTCTGGTACATCCCTGAGGATCCTCTCATCTTG

GCCATCAGGAACCTCTACAGAAGGTCAAATTCAGTGGGTTCTTCTCAGTGCCTCTGACTT

GAGTTACTAATAACATTTGCACTATAATCCACTTCTTTCTGATGAACTACCCTGTCCTTA

TTTTTCTCCTGTTTACCTGGATCCTCCTTATCATCTTTTAAACCACCTCTTAACTATCAT

GTTCTCTCATTATACCCTGAGATCTCGGCAATTCTGATTTTTGGCACTCTTCCTGGAAAA

TCTTATTTAACCTGCACCTGCCACTAATGACTCTCAGTTCTATGGCCTAAATTCCTCTCC

TGAGACCACCCATAATCCACAAATATCTATGTATTATTTCTCCTTAGATGACTTTCAGGT

CTTCTAAGTGCAATAGCCCCACAGTAAACTCAGTATCTTCTCCCGGTCAGGCTGTCTTCC

CTGAGAGAAGTGGCTTTTGCCCTGTTTTCTGAATGCCTACATTGAAGCCATCTGTTCCCC

AGGAAGCCTTCCCTGATGTGCTGTTTGGTCGCATCTTGTGTATACCTACGTATCTGCACT

TATCCTTCTGAACCTGCTGTTGTCCTGTCACTTGTGTTTCCTTCTGTGACTTATACGCGT

CTGCAGAACAGGACGTATGTATTATTTTTATTTGGGTATTTAGCATCTAACAGTGTTTGA

CATATAGTAGTCTTTTAATACATATTTTTGTCTGAATGGAAATGATATTTTGAAGAAAAA

TAATCTGTTCCATAGCTGGCTGATCTTTGGACTGCAGAACTTGTGAAAGTGTTTTTTAAA

AAGCATTTTAAAAAGTACAAGGGACATTCATGTATTAAGAAGATGAGTTTCCAATAACTG

CTAGAGGACTTTGTGTCTTTTTATTTTACCCTCTTTTTCCTGATGAGTCCTTTGAGTCCT

TTAAACTGAGGAGCAAGCTAAGTTTCCTAGTGAAATACCTATAGGATTTGTTTTGTTTAG

TTTCAAATACCACTCTTTGCTTGGCCACTTACTGTGTCAGGGAGTCATTCTCAGTGAAAA

ATAAGACACAGGTCATACCCTCTAGACACTTACAATTACAGTGGCAAGGAGTCATTCTCC

TGTCACTGTAAGTGGCCAAGCACAGACTGGGTCCCCACATGTCAGGGCTGAAAACTCACA

GGGAAATCTGTGAGTTGGGAGGTGAGAGCAGAAGAGTCCCGTAGTTCCTTCTCACTCTGA

TGCATTTATCATTCTAAACCCAGACTTTCACATACACATTCATCGTTTTCTTTCATGATA

ATAGTTGCTTTTATCCTCTTATCTTTGCTAATTCTTACAAACTAATAAAGACTAAGAAAC

AAAATAAATTAAATCCTACAGGTGTTCCAAACTCAGCAATAATTTCTAGTTGGCCTCTAA

AACAAAAATCAAAATATAAATGTAAGAAAAGTTTAGAATGCTTAGTACCTGTGTGATGAA

ATAATCTGTACACTAAACCCCCAAGTCATGAGTTTACCTATACAACAAACCTGCACATGT

ACTCCTGAACATAAAATAAATGTTGAAATATTTTTAAAAAGGAAACAAAAGTTTGGAACA

AATGCCAAAATAACTGTACTGTACTTTTGAATTTATATGCCCCAAATGAAAAATATTATC

AACAAAGCTATACATTCTACAGTTTCATGTTCATAAACTAAGACAGAAACTTTAAAACTG

TCAAGAGCCCTAAAATTTGAAGGATATTTTCTTCTTCCTCTCAATTTTGTATTTTTTTCT

ACCTTTTCTATAATAAGAAAAAGAAAATGTCCATTCCCCCACCCCCATGACTCTAAAAAC

AATTTTACATCTGTGTCATAGAAAAATTAAGATCTTAATGGGAGAGAAAACCTCTCTACT

AGTTCCGCCAGTAGCCGTATGACCTTAGCAAGTTATTAATATGTAACTTCCCTGCATTTC

CTTACCTGTAAAATGTATGATATGTATTTGCTTCATAGGGTTATTGTGACAATTCAGCGA

GTGAAATATGTAAAGTATTTAGAAGGATGCCTGGCACAAGTAAGTGCTCAACAAATGTTA

GCTGTCATTGTTACTATTACTATTGTGTAGGGTCAGGATGCCCAGACTTTCAAAGACCAG

GAAGCAGCTTGACTTATCAGTGATAAACTTTTCATTTTGTTCTTTGCTCCTTTCTTTTTA

TAACTGCTCATCTGCTCTGTATTATTTCCTTTATGGTGTTGCTCCTTCTTCTTCCCCATA

TGTCCTTCCTTTGACCTCTTACCTTCTTCCTTTTTATATTCATAAGTCTTTATTCATTCT

CTAGCTTTGACCACTTGCATATTCAAACTGACATTTTGTCGTGTTTTTCTCTACTGTCTT

TATGCAGCGGACCATGTGTCAACTTATGCCGCGTTTGTACAGACGCATAGACCAACAGGG

GAGTTTATGTTTGAATTTGATGAAGATGAGATGTTCTATGTGGATCTGGACAAGAAGGAG

ACCGTCTGGCATCTGGAGGAGTTTGGCCAAGCCTTTTCCTTTGAGGCTCAGGGCGGGCTG

GCTAACATTGCTATATTGAACAACAACTTGAATACCTTGATCCAGCGTTCCAACCACACT

CAGGCCACCAACGGTACGCCCTATCTTTGCCTCTTCCTCTGTAGCCCAACTGGAAGGGAT

GAGAGGGCCTCTCTGCCACCCTCAGACTAGGAAGCCTAAGTGCCCCCTGCTGTGTGATCC

TCTTCCCCTAGTGGCCATGGGCTGATCCCACTACAGCAAGGGCTTGCATCCTCTCTTCTC

AGGAGAGAGAAAGGTGAGCAGAGTGAGGCTGGTCAGTGGTGTGATACCCCTCTCTGTGAT

TCAGAGCTGCCATAAAATCTAAGGCTGAGGTAGAGGACCACCCTCCCCTAAGAGGTGGAG

CCTTTGTGATTCATCCCAGAAGAGGGGCCTAACCTGGTGCTGTCTCCTTCCAGATCCCCC

TGAGGTGACCGTGTTTCCCAAGGAGCCTGTGGAGCTGGGCCAGCCCAACACCCTCATCTG

CCACATTGACAAGTTCTTCCCACCAGTGCTCAACGTCACGTGGCTGTGCAACGGGGAGCT

GGTCACTGAGGGTGTCGCTGAGAGCCTCTTCCTGCCCAGAACAGATTACAGCTTCCACAA

GTTCCATTACCTGACCTTTGTGCCCTCAGCAGAGGACTTCTATGACTGCAGGGTGGAGCA

CTGGGGCTTGGACCAGCCGCTCCTCAAGCACTGGGGTATGCAACTGCTTTTCTCTCCATA

ATCTCCTGGCATCCTCTATTCCAAAGACCTGGTGTCCTCTGCACCAGCTTTCCGCACTGG

CTGGGTCTCAGTCCTCTCCTCGTCCTAACATCCAATTAACTGGTCCATAACCTTCAATTC

CCACAACCATCCCAGGCCATCACCACCCTCACTGCACCTCCTGACCCTATCTCTTCATTC

TTCCCCCAGAGGCCCAAGAGCCAATCCAGATGCCTGAGACAACGGAGACTGTGCTCTGTG

CCCTGGGCCTGGTGCTGGGCCTAGTCGGCATCATCGTGGGCACCGTCCTCATCATAAAGT

CTCTGCGTTCTGGCCATGACCCCCGGGCCCAGGGGACCCTGTGAAATACTGTAAAGGTGG

GAATGTAAAGAGGAGGCCCTAGGATTTGTAGAATGTAAGGAAGGGAGGAAAAATTCAATC

TGATAAGTGTTCATTGATCTTCTAATGGGTTAAAAGCATTCAGCCACATAACAACAACAA

CACCGATAACTAACTGAGTAGTTAATATGGTCAGGCGCTATTCTGAGGATTTACATTTAT

TAACTCACTTTATTCTCACACATAGTCTTTGAGGTAGGTACTATTATTTTCACTATTTCA

CATGAGAGATACTTACATCTTTTTACATACACAGAGACTTTAAGCACTTTGATCAAGTTC

CCACAGCTATGAAGTAGTAGGGCTAGCTTCCAATCCAGAAAGTCTGGATCCAAGACTGTT

TATCCACTGTCCTATTCACCCTATTTTGTGAAGGAAAAGACCAAGTTCAAATTCTCCAGA

GTCCATTGCCAAATAATGGAGTCAGATCTATATTTCTATACATAATTACAACACAGTGTG

GTGGGTGCCTGTAACTACTTACTGTCTCTACTTGGACTCATTCCATGGCAATGTTCACAC

AAAAAATGCCCCTCCAGAGATCTTACAGGTTTCTATTTATCATAACACTCACCATGCTTT

ATATTTTTATATGTTTTGGGAATTCTCTTAGCATTAGACAGTGAACTTCCATGCAGATGA

CCACATCTAATTCATTATTATTATTGTTATTCATGCTGGACCTCAGGTACAAAAGGTTAA

GAACTTCTCAGTTCATTATATGATCATCATTGGTGCCTCCGAGCTCTCTCTCTCTCCCTT

GATTTATTTGGTCCCTTTTATCTCCAGTCCTTACTCCCATATCTAACCTCTTACCCCTAC

CTCATAGGTAAACATTTTAATGAATTTGATGTTTCCTTTTATTTGCATAGATCCTCTGTA

ATATGTAGTAGTGTCCAGTGTACATGTATTTTTAATTAACCAAAATGGCATTAAATTATA

GATCTAATTTTGTACATCCAGTTTGTTTCTTCCAAATCTTCCATAGTATTTTACTTTATA

TGTCCATGCATTAGTCCATTTTGCATTGCTATAAAGGAATATCTGAAGTTACCTAATTTA

CGAAGAAAAGAGCTTTAAATGGCTCACAGATCTGCAGGCTGTACGCGAAACATGGCACTA

GCATCTGCTTCTGTTGGGGGATTCTGGAAGCTTTTACTCATGGTGGAAGGCAAGTGGAGC

CAGTGCATCACATGGTCATAGAGGGAGAAAGAGACATAGAAAGAGGTGCCAGCCTCTTTT

TAACAACCAGGTTTCATGTGCACTAATAGAGTGAGAACTCACTCATTACCCGGAGAGGGG

ACAAAGCCATTCATGAGGGTCTCCTCCATGATTCAAATACCTCCCACCAGGCCCCACCTG

CAACACTGGGGATCAATTTTCAACATGAGACTTGGAAGTGACAAATATCCAAATCATATT

AATCCACATATCTACATTGCTCCTGGGATACCTGGATCATTCCTGGTTCTCTACTATTGC

AAGCAATGCTTGTATCTCACATGGAACTGCATATACATGTGGGCCTGACCTGCATCCCTG

GAATGTATGTATCCTAGAAAGGGGTTGCAGGGTTGCTGGAGATGCAGCTCCTTAATTTGA

CTAAACACTGCTCATCTTCTCATCAGAATGGCTGTACTCATCTGAACTTCCTTTGTCAGT

ACTCTAATTGTCCTGCAACTCCTAAATGGACTTCAACACTGGACATTATCCAGTTTTCTA

ACTTTTGCCAATTTCATGTGCATAAAGAAATATGCTGTTTTATTTTGCATTTCTTTAATT

ACTAATAATTGGGGCTATAATTAGGACTGATTAGCCACTTGGGGGTTCCTTTTCTATAAA

TTGCCTGTTCACATTCATTGTCCATTTTTGTACTATGTGCTTCCATCATTTTCTTATTGA

TTTGCAGGTGATCCTTATATAGTCCTGCTAGTAGTCCCTTGTCAGTTTTAGGCATTGCAA

ATGTTTTCCTCTAATCTGACTTCTGGCAACTGTCTCCTTGGTTTCCTTTATTGAAGAGAA

ATCCTTAATATTTTGTAATGAAGTCCATCAACTGTATTTTTGTTTGTGTGTCTTTTTTAA

AAGAAGTCTTCCCTATACTGAGATATCAAAGATACTCTTAAAACATCTCCTACAGTTTTA

AATTTCACATTTACTACTTTAATTCATCTGGGATTCATCTTTGTGTTTGATGGGGATCAT

GTTTTATTTTTCTTTATATAATGGGCCAGTGTGTTCCCACAACTACTAAATAGTTCACCT

TTTCCCCATAGGTTAGTAGTGTCTCCTTTGCTATACTGAAAGCTCCCATTATAGGTGGGC

CTGTGTCTGAGTTCCATCTTGTTCCACTGTTCTGTTTGTCTCTTCTTGTGCCAGTGTCCT

AGTATTTTGATTACTATGACATTGTAGTGTGTGTTAGTATCCAGTAGGACAAATTCTTGT

TTATTTTTCTTAGTTCACACACATTTATAATTATATCTATAATGATTTGTAACAGAGTGA

AGTGAATGTAGAATGTCAGATGTTAAGAGGAAGAATGGAAAAGAGGGCTGGGACTAGGGT

GATGTAGGGGATGCACCTGGCTTAGGTGCAAAATTTGGGGGATACCAAAAGAACTCAGTA

ATAAATCATATTTTAATGAAATATCTTGAAAAGGCAAAATTAATGCAAAGATACATGATT

AACAAAACATCCAAAGAGGAGTATTTAACAAAAATGGAGAAGCAGAGAAGCAGAAGAATT

AGGAGAATATGCTGTCACATGAGCCAAGGAATTAAAGAATTCAGGAAGGAGGAAGTACTG

CTGTCAGATGTTCAACAGAGGTCATTTTAGAAAATTTACCTTGGTTTTTGAAATCCTTTC

AAAGAGCAGTATACACAATGTGAGCAAGTATCCTTCGTTCATTGCCGTCATTGATATGGT

TTGGATATTTGTCCCTTCCAATTCTCATTCCAGGGTTAAGCTTCTTCTCTGCCCTCAGTA

ATGTGGCCCTTCCCCTTGTCTGTATATTTTGGAGACATGAAGCATGTGGGATGGCCTCAC

AGTCAGCTGGGGTTTGAGGGTGAAATTCAATGACTTTCGTGAACTCCTTGGCTCCTATGT

GCTCTTCACCTGGAGGACCAGGGCATGTGCAGGGATGACCACCTTCTCCCTGGGACCTGA

ACAGGGCAGAGAAATGGGAAGCTCGGGTGCAAAGGGAGTGGGGAAGATGGGTCCGGGCTT

ACAGTACTGAACCCAGGAATGACAATAACTGTGTGTGTTGCTGCAGGTGACAAAATATCT

GAACAGAAGAGGACTTAGGAGAGATCTGAACTCCAGCTGCCCTACAAACTCCATCTCAGC

TTTTCTTCTCACTTCATGTGAAAACTACTCCAGTGGCTGACTGAATTGCTGACCCTTCAA

GCTCTGTCCTTATCCATTACCTCAAAGCAGTCATTCCTTAGTAAAGTTTCCAACAAATAG

AAATTAATGACACTTTGGTAGCACTAATATGGAGATTATCCTTTCATTGAGCCTTTTATC

CTCTGTTCTCCTTTGAAGAACCCCTCACTGTCACCTTCCCGAGAATACCCTAAGACCAAT

AAATACTTCAGTATTTCAGAGCGGGGAGACTCTGAGTCATTCTTACTGGAAGTCTAGGAC

CAGGTCACATGTGAATACTATTTCTTGAAGGTGTGGTTTCAACCTCTGTTGCCGATGTGG

TTACTAAAGGTTCTGATCCCACTTGAACGGAAAGGTCTGAGGATATTGATTCAGTCCTGG

GTTTTTCCCTAACTACAGGATAGGGTGGGGTAGAGAAAGGATATTTGGGGGAAATTTTAC

TTGGATGAAGATTTTCTTGGATGTAGTTTGAAGACTGCAGTGTTTGAAGTCTCTGAGGGA

AGAGATTTGGTCTGTCTGGATCAAGATTTCAGGCAGATTAGGATTCCATTCACAGCCCCT

GAGCTTCCTTCCCAAGGCTGTATTGTAATTATAGCAATATTTCATGGAGGATTTTTCTAC

ATGATAAACTAAGAGCCAAGAAATAAAATTTTTAAAATGCCCTAATTCATTGCAATTTTT

ACCAGCCATAGTCACTCCATGTGGGAGAACTTAAATCATGATTACCAGAGCTTTCAAAGG

TTTGAGAATAGTGATGATTATGAAGAAAAATATCTTATTTGAGCAAGGATTTTGTTTCTT

TATGAGTGTTCATTAGATATTACGATGAAAAAAGCATGAAATGGTAAAAATTCAGATAAA

TATAAAAACATGTTCTCTAGTTTTTTTTAAGTTAAAAAAGGAATTGTTTAAAGTA

>HLA:HLA06607 DPA1*01:03:01:04

GGTGGACCTGAAAGAAAGATTAATTATATATTTTATTAGATAGCACATTCATTCTTAATT

ATCAGCAGAATATTTTTGTAAAATATTGACAACATAACATAAAACATGATTTAAAATATA

GTGACCAAAAAACATCAAAGATCATCTGACTGTCTGGGATGGGGGTGGGGCTCGGGAGAA

GGAACATAATGGAAAACATTTGCCGTCAGAAGAAGGTGTAACTTATCTTTTTACATCTCT

TTCTCTAACTCTGAAAATGAACTGTGAACTGGAGCTCTCTTGACCACGCTGGTACCTAAA

ATTCTCCCATCTCTTCCCCAGCACCTTCCAGCGTCCTCTTTACCCAGCAACAGAGAATGT

CAGCTCTATGATTTCTCTGATAGGTGAATCCCAGCCATGCTGATTCCTCTCCACCCATTT

CCAGTGCTAGAGGCCCACAGTTTCAGTCTCATCTGCCTCCACTCGGCCTCAGTTCCTCAT

CACTGTTCCTGTGCTCACAGTCATCAATTATAGACCCCACAACATGCGCCCTGAAGACAG

AATGTTCCATATCAGAGCTGTGATCTTGAGAGCCCTCTCCTTGGCTTTCCTGCTGAGTCT

CCGAGGAGCTGGGGCCATCAAGGGTGAGTGCTCAGGAGGACGCAGGAGCGTCGGGGTGAG

TGATGGGGTGGTTCACATCAATTGCTGCTTCAGGGATCACAGATTTTAGGGGCTCATTGA

TCTATCTGGTCCTCATAGTCTATGTTCCCTCTGGCCCTCATAATAATAACAGCAATAACA

GCCAGAATTTATGAGACTCCTGCATAGTTTCTTTCCCCATTTACATCTCACAGGAATCTT

CAATGAAGATAATATTCCATTCATTTAGAAATTATTCCTTTTATTTAGAAATTATTTTGA

AAAAACTGAAGCTCAAAAAGATGAATAAGTTTTCCAAGGTTACACAGCAGATCAACGAGC

CAAGTTTGAAGTCCAGACCCAGCTCTGAGGGTCATACACTGCCTTCCCCAGATTCCTGCA

CACAGTGACCTACTATCAGGGCCCTCCTATCTCTCTGGGATCCCCAGCCTCTATCTTTTG

TGGCTGCTTTACAGGAACTCCGAGCTATGGACTCTGCATTAGGAGACGAAGTGCAAAGAG

TGTTTCTGTATCCTCCCTCTCTTCTAGGACCCTAGGGCTCTTCCTGGGTCTTTGTGGGTG

GTCACAAGCTTTCCTCTCTCAAGACAGCAGGGTTGCATGGTCTTGATAGCCTTGTGATTC

GGGTTCTGAGAGATTCAGGACTGCAAGGGAGGCCTAGACTTTTGATAGCTGCAAGGACTC

AGCCAGAGATGGACCGTAGTGAATGCTCCTTTTTCCTGTAGCTGAAATCAGGGAGAATGA

CATCAAGCCTGTGCATGATGCTGTCATTCCAAAATCTAGTGATGGGGAAGGTTAGAATCC

ATAACGTACAAGATGCACACTGGCTTCAGACAGTTTTATTTAAGATGTGTAGAATAAAGA

GGAGGTCAGGCTGGGTAGAACCAGAAGTATCTATTGCCCTGTTCGCGGTCACCTGAGTTA

TTTCTAATGTTATGTTATAATAAACACCACAATAGGCTTCTCTTCATAGATGCAAATACT

TTTTAGTATTCTTGGTAGAAATTCCTAATGAGCTCAGCTGTCTCTTCAGGGCTTCCCTGC

CCAGTCTCTTAACATTTAAACATGTCATTTACCTTAAAAACATAAGTGCAAACCAACTGA

TAAAAAACAACCTTGCCTTCAGTCTGCATCCTGTCCCAGAGACACTTTCTTTGTGTCCTC

ACACGTGGAGCTAAGCTTCTGACTTGTCTCTGGTACATCCCTGAGGATCCTCTCATCTTG

GCCATCAGGAACCTCTACAGAAGGTCAAATTCAGTGGGTTCTTCTCAGTGCCTCTGACTT

GAGTTACTAATAACATTTGCACTATAATCCACTTCTTTCTGATGAACTACCCTGTCCTTA

TTTTTCTCCTGTTTACCTGGATCCTCCTTATCATCTTTTAAACCACCTCTTAACTATCAT

GTTCTCTCATTATACCCTGAGATCTCAGCAATTCTGATTTTTGGCACTCTTCCTGGAAAA

TCTTATTTAACCTGCACCTGCCACTAATGACTCTCAGTTCTATGGCCTAAATTCCTCTCC

TGAGACCACCCATAATCCACAAATATCTATGTATTATTTCTCCTTAGATGACTTTCAGGT

CTTCTAAGTGCAATAGCCCCACAGTAAACTCAGTATCTTCTCCCGGTCAGGCTGTCTTCC

CTGAGAGAAGTGGCTTTTGCCCTGTTTTCTGAATGCCTACATTGAAGCCATCTGTTCCCC

AGGAAGCCTTCCCTGATGTGCTGTTTGGTCGCATCTTGTGTATACCTACGTATCTGCACT

TATCCTTCTGAACCTGCTGTTGTCCTGTCACTTGTGTTTCCTTCTGTGACTTATACGCGT

CTGCAGAACAGGACGTATGTATTATTTTTATTTGGGTATTTAGCATCTAACAGTGTTTGA

CATATAGTAGTCTTTTAATACATATTTTTGTCTGAATGGAAATGATATTTTGAAGAAAAA

TAATCTGTTCCATAGCTGGCTGATCTTTGGACTGCAGAACTTGTGAAAGTGTTTTTTAAA

AAGCATTTTAAAAAGTACAAGGGACATTCATGTATTAAGAAGATGAGTTTCCAATAACTG

CTAGAGGACTTTGTGTCTTTTTATTTTACCCTCTTTTTCCTGATGAGTCCTTTGAGTCCT

TTAAACTGAGGAGCAAGCTAAGTTTCCTAGTGAAATACCTATAGGATTTGTTTTGTTTAG

TTTCAAATACCACTCTTTGCTTGGCCACTTACTGTGTCAGGGAGTCATTCTCAGTGAAAA

ATAAGACACAGGTCATACCCTCTAGACACTTACAATTACAGTGGCAAGGAGTCATTCTCC

TGTCACTGTAAGTGGCCAAGCACAGACTGGGTCCCCACATGTCAGGGCTGAAAACTCACA

GGGAAATCTGTGAGTTGGGAGGTGAGAGCAGAAGAGTCCCGTAGTTCCTTCTCACTCTGA

TGCATTTATCATTCTAAACCCAGACTTTCACATACACATTCATCGTTTTCTTTCATGATA

ATAGTTGCTTTTATCCTCTTATCTTTGCTAATTCTTACAAACTAATAAAGACTAAGAAAC

AAAATAAATTAAATCCTACAGGTGTTCCAAACTCAGCAATAATTTCTAGTTGGCCTCTAA

AACAAAAATCAAAATATAAATGTAAGAAAAGTTTAGAATGCTTAGTACCTGTGTGATGAA

ATAATCTGTACACTAAACCCCCAAGTCATGAGTTTACCTATACAACAAACCTGCACATGT

ACTCCTGAACATAAAATAAATGTTGAAATATTTTTAAAAAGGAAACAAAAGTTTGGAACA

AATGCCAAAATAACTGTACTGTACTTTTGAATTTATATGCCCCAAATGAAAAATATTATC

AACAAAGCTATACATTCTACAGTTTCATGTTCATAAACTAAGACAGAAACTTTAAAACTG

TCAAGAGCCCTAAAATTTGAAGGATATTTTCTTCTTCCTCTCAATTTTGTATTTTTTTCT

ACCTTTTCTATAATAAGAAAAAGAAAATGTCCATTCCCCCACCCCCATGACTCTAAAAAC

AATTTTACATCTGTGTCATAGAAAAATTAAGATCTTAATGGGAGAGAAAACCTCTCTACT

AGTTCCGCCAGTAGCCGTATGACCTTAGCAAGTTATTAATATGTAACTTCCCTGCATTTC

CTTACCTGTAAAATGTATGATATGTATTTGCTTCATAGGGTTATTGTGACAATTCAGCGA

GTGAAATATGTAAAGTATTTAGAAGGATGCCTGGCACAAGTAAGTGCTCAACAAATGTTA

GCTGTCATTGTTACTATTACTATTGTGTAGGGTCAGGATGCCCAGACTTTCAAAGACCAG

GAAGCAGCTTGACTTATCAGTGATAAACTTTTCATTTTGTTCTTTGCTCCTTTCTTTTTA

TAACTGCTCATCTGCTCTGTATTATTTCCTTTATGGTGTTGCTCCTTCTTCTTCCCCATA

TGTCCTTCCTTTGACCTCTTACCTTCTTCCTTTTTATATTCATAAGTCTTTATTCATTCT

CTAGCTTTGACCACTTGCATATTCAAACTGACATTTTGTCGTGTTTTTCTCTACTGTCTT

TATGCAGCGGACCATGTGTCAACTTATGCCGCGTTTGTACAGACGCATAGACCAACAGGG

GAGTTTATGTTTGAATTTGATGAAGATGAGATGTTCTATGTGGATCTGGACAAGAAGGAG

ACCGTCTGGCATCTGGAGGAGTTTGGCCAAGCCTTTTCCTTTGAGGCTCAGGGCGGGCTG

GCTAACATTGCTATATTGAACAACAACTTGAATACCTTGATCCAGCGTTCCAACCACACT

CAGGCCACCAACGGTACGCCCTATCTTTGCCTCTTCCTCTGTAGCCCAACTGGAAGGGAT

GAGAGGGCCTCTCTGCCACCCTCAGACTAGGAAGCCTAAGTGCCCCCTGCTGTGTGATCC

TCTTCCCCTAGTGGCCATGGGCTGATCCCACTACAGCAAGGGCTTGCATCCTCTCTTCTC

AGGAGAGAGAAAGGTGAGCAGAGTGAGGCTGGTCAGTGGTGTGATACCCCTCTCTGTGAT

TCAGAGCTGCCATAAAATCTAAGGCTGAGGTAGAGGACCACCCTCCCCTAAGAGGTGGAG

CCTTTGTGATTCATCCCAGAAGAGGGGCCTAACCTGGTGCTGTCTCCTTCCAGATCCCCC

TGAGGTGACCGTGTTTCCCAAGGAGCCTGTGGAGCTGGGCCAGCCCAACACCCTCATCTG

CCACATTGACAAGTTCTTCCCACCAGTGCTCAACGTCACGTGGCTGTGCAACGGGGAGCT

GGTCACTGAGGGTGTCGCTGAGAGCCTCTTCCTGCCCAGAACAGATTACAGCTTCCACAA

GTTCCATTACCTGACCTTTGTGCCCTCAGCAGAGGACTTCTATGACTGCAGGGTGGAGCA

CTGGGGCTTGGACCAGCCGCTCCTCAAGCACTGGGGTATGCAACTGCTTTTCTCTCCATA

ATCTCCTGGCATCCTCTATTCCAAAGACCTGGTGTCCTCTGCACCAGCTTTCCGCACTGG

CTGGGTCTCAGTCCTCTCCTCGTCCTAACATCCAATTAACTGGTCCATAACCTTCAATTC

CCACAACCATCCCAGGCCATCACCACCCTCACTGCACCTCCTGACCCTATCTCTTCATTC

TTCCCCCAGAGGCCCAAGAGCCAATCCAGATGCCTGAGACAACGGAGACTGTGCTCTGTG

CCCTGGGCCTGGTGCTGGGCCTAGTCGGCATCATCGTGGGCACCGTCCTCATCATAAAGT

CTCTGCGTTCTGGCCATGACCCCCGGGCCCAGGGGACCCTGTGAAATACTGTAAAGGTGG

GAATGTAAAGAGGAGGCCCTAGGATTTGTAGAATGTAAGGAAGGGAGGAAAAATTCAATC

TGATAAGTGTTCATTGATCTTCTAATGGGTTAAAAGCATTCAGCCACATAACAACAACAA

TACCGATAACTAACTGAGTAGTTAATATGGTCAGGCGCTATTCTGAGGATTTACATTTAT

TAACTCACTTTATTCTCACACATAGTCTTTGAGGTAGGTACTATTATTTTCACTATTTCA

CATGAGAGATACTTACATCTTTTTACATACACAGAGACTTTAAGCACTTTGATCAAGTTC

CCACAGCTATGAAGTAGTAGGGCTAGCTTCCAATCCAGAAAGTCTGGATCCAAGACTGTT

TATCCACTGTCCTATTCACCCTATTTTGTGAAGGAAAAGACCAAGTTCAAATTCTCCAGA

GTCCATTGCCAAATAATGGAGTCAGATCTATATTTCTATACATAATTACAACACAGTGTG

GTGGGTGCCTGTAACTACTTACTGTCTCTACTTGGACTCATTCCATGGCAATGTTCACAC

AAAAAATGCCCCTCCAGAGATCTTACAGGTTTCTATTTATCATAACACTCACCATGCTTT

ATATTTTTATATGTTTTGGGAATTCTCTTAGCATTAGACAGTGAACTTCCATGCAGATGA

CCACATCTAATTCATTATTATTATTGTTATTCATGCTGGACCTCAGGTACAAAAGGTTAA

GAACTTCTCAGTTCATTATATGATCATCATTGGTGCCTCCGAGCTCTCTCTCTCTCCCTT

GATTTATTTGGTCCCTTTTATCTCCAGTCCTTACTCCCATATCTAACCTCTTACCCCTAC

CTCATAGGTAAACATTTTAATGAATTTGATGTTTCCTTTTATTTGCATAGATCCTCTGTA

ATATGTAGTAGTGTCCAGTGTACATGTATTTTTAATTAACCAAAATGGCATTAAATTATA

GATCTAATTTTGTACATCCAGTTTGTTTCTTCCAAATCTTCCATAGTATTTTACTTTATA

TGTCCATGCATTAGTCCATTTTGCATTGCTATAAAGGAATATCTGAAGTTACCTAATTTA

CGAAGAAAAGAGCTTTAAATGGCTCACAGATCTGCAGGCTGTACGCGAAACATGGCACTA

GCATCTGCTTCTGTTGGGGGATTCTGGAAGCTTTTACTCATGGTGGAAGGCAAGTGGAGC

CAGTGCATCACATGGTCATAGAGGGAGAAAGAGACATAGAAAGAGGTGCCAGCCTCTTTT

TAACAACCAGGTTTCATGTGCACTAATAGAGTGAGAACTCACTCATTACCCGGAGAGGGG

ACAAAGCCATTCATGAGGGTCTCCTCCATGATTCAAATACCTCCCACCAGGCCCCACCTG

CAACACTGGGGATCAATTTTCAACATGAGACTTGGAAGTGACAAATATCCAAATCATATT

AATCCACATATCTACATTGCTCCTGGGATACCTGGATCATTCCTGGTTCTCTACTATTGC

AAGCAATGCTTGTATCTCACATGGAACTGCATATACATGTGGGCCTGACCTGCATCCCTG

GAATGTATGTATCCTAGAAAGGGGTTGCAGGGTTGCTGGAGATGCAGCTCCTTAATTTGA

CTAAACACTGCTCATCTTCTCATCAGAATGGCTGTACTCATCTGAACTTCCTTTGTCAGT

ACTCTAATTGTCCTGCAACTCCTAAATGGACTTCAACACTGGACATTATCCAGTTTTCTA

ACTTTTGCCAATTTCATGTGCATAAAGAAATATGCTGTTTTATTTTGCATTTCTTTAATT

ACTAATAATTGGGGCTATAATTAGGACTGATTAGCCACTTGGGGGTTCCTTTTCTATAAA

TTGCCTGTTCACATTCATTGTCCATTTTTGTACTATGTGCTTCCATCATTTTCTTATTGA

TTTGCAGGTGATCCTTATATAGTCCTGCTAGTAGTCCCTTGTCAGTTTTAGGCATTGCAA

ATGTTTTCCTCTAATCTGACTTCTGGCAACTGTCTCCTTGGTTTCCTTTATTGAAGAGAA

ATCCTTAATATTTTGTAATGAAGTCCATCAACTGTATTTTTGTTTGTGTGTCTTTTTTAA

AAGAAGTCTTCCCTATACTGAGATATCAAAGATACTCTTAAAACATCTCCTACAGTTTTA

AATTTCACATTTACTACTTTAATTCATCTGGGATTCATCTTTGTGTTTGATGGGGATCAT

GTTTTATTTTTCTTTATATAATGGGCCAGTGTGTTCCCACAACTACTAAATAGTTCACCT

TTTCCCCATAGGTTAGTAGTGTCTCCTTTGCTATACTGAAAGCTCCCATTATAGGTGGGC

CTGTGTCTGAGTTCCATCTTGTTCCACTGTTCTGTTTGTCTCTTCTTGTGCCAGTGTCCT

AGTATTTTGATTACTATGACATTGTAGTGTGTGTTAGTATCCAGTAGGACAAATTCTTGT

TTATTTTTCTTAGTTCACACACATTTATAATTATATCTATAATGATTTGTAACAGAGTGA

AGTGAATGTAGAATGTCAGATGTTAAGAGGAAGAATGGAAAAGAGGGCTGGGACTAGGGT

GATGTAGGGGATGCACCTGGCTTAGGTGCAAAATTTGGGGGATACCAAAAGAACTCAGTA

ATAAATCATATTTTAATGAAATATCTTGAAAAGGCAAAATTAATGCAAAGATACATGATT

AACAAAACATCCAAAGAGGAGTATTTAACAAAAATGGAGAAGCAGAGAAGCAGAAGAATT

AGGAGAATATGCTGTCACATGAGCCAAGGAATTAAAGAATTCAGGAAGGAGGAAGTACTG

CTGTCAGATGTTCAACAGAGGTCATTTTAGAAAATTTACCTTGGTTTTTGAAATCCTTTC

AAAGAGCAGTATACACAATGTGAGCAAGTATCCTTCGTTCATTGCCGTCATTGATATGGT

TTGGATATTTGTCCCTTCCAATTCTCATTCCAGGGTTAAGCTTCTTCTCTGCCCTCAGTA

ATGTGGCCCTTCCCCTTGTCTGTATATTTTGGAGACATGAAGCATGTGGGATGGCCTCAC

AGTCAGCTGGGGTTTGAGGGTGAAATTCAATGACTTTCGTGAACTCCTTGGCTCCTATGT

GCTCTTCACCTGGAGGACCAGGGCATGTGCAGGGATGACCACCTTCTCCCTGGGACCTGA

ACAGGGCAGAGAAATGGGAAGCTCGGGTGCAAAGGGAGTGGGGAAGATGGGTCCGGGCTT

ACAGTACTGAACCCAGGAATGACAATAACTGTGTGTGTTGCTGCAGGTGACAAAATATCT

GAACAGAAGAGGACTTAGGAGAGATCTGAACTCCAGCTGCCCTACAAACTCCATCTCAGC

TTTTCTTCTCACTTCATGTGAAAACTACTCCAGTGGCTGACTGAATTGCTGACCCTTCAA

GCTCTGTCCTTATCCATTACCTCAAAGCAGTCATTCCTTAGTAAAGTTTCCAACAAATAG

AAATTAATGACACTTTGGTAGCACTAATATGGAGATTATCCTTTCATTGAGCCTTTTATC

CTCTGTTCTCCTTTGAAGAACCCCTCACTGTCACCTTCCCGAGAATACCCTAAGACCAAT

AAATACTTCAGTATTTCAGAGCGGGGAGACTCTGAGTCATTCTTACTGGAAGTCTAGGAC

CAGGTCACATGTGAATACTATTTCTTGAAGGTGTGGTTTCAACCTCTGTTGCCGATGTGG

TTACTAAAGGTTCTGATCCCACTTGAACGGAAAGGTCTGAGGATATTGATTCAGTCCTGG

GTTTTTCCCTAACTACAGGATAGGGTGGGGTAGAGAAAGGATATTTGGGGGAAATTTTAC

TTGGATGAAGATTTTCTTGGATGTAGTTTGAAGACTGCAGTGTTTGAAGTCTCTGAGGGA

AGAGATTTGGTCTGTCTGGATCAAGATTTCAGGCAGATTAGGATTCCATTCACAGCCCCT

GAGCTTCCTTCCCAAGGCTGTATTGTAATTATAGCAATATTTCATGGAGGATTTTTCTAC

ATGATAAACTAAGAGCCAAGAAATAAAATTTTTAAAATGCCCTAATTCATTGCAATTTTT

ACCAGCCATAGTCACTCCATGTGGGAGAACTTAAATCATGATTACCAGAGCTTTCAAAGG

TTTGAGAATAGTGATGATTATGAAGAAAAATATCTTATTTGAGCAAGGATTTTGTTTCTT

TATGAGTGTTCATTAGATATTACGATGAAAAAAGCATGAAATGGTAAAAATTCAGATAAA

TATAAAAACATGTTCTCTAGTTTTTTTTAAGTTAAAAAAGGAATTGTTTAAAGTA

>HLA:HLA06606 DPA1*01:03:01:05

GGTGGACCTGAAAGAAAGATTAATTATATATTTTATTAGATAGCACATTCATTCTTAATT

ATCAGCAGAATATTTTTGTAAAATATTGACAACATAACATAAAACATGATTTAAAATATA

GTGACCAAAAAACATCAAAGATCATCTGACTGTCTGGGATGGGGGTGGGGCTCGGGAGAA

GGAACATAATGGAAAACATTTGCCGTCAGAAGAAGGTGTAACTTATCTTTTTACATCTCT

TTCTCTAACTCTGAAAATGAACTGTGAACTGGAGCTCTCTTGACCACGCTGGTACCTAAA

ATTCTCCCATCTCTTCCCCAGCACCTTCCAGCGTCCTCTTTACCCAGCAACAGAGAATGT

CAGCTCTATGATTTCTCTGATAGGTGAATCCCAGCCATGCTGATTCCTCTCCACCCATTT

CCAGTGCTAGAGGCCCACAGTTTCAGTCTCATCTGCCTCCACTCGGCCTCAGTTCCTCAT

CACTGTTCCTGTGCTCACAGTCATCAATTATAGACCCCACAACATGCGCCCTGAAGACAG

AATGTTCCATATCAGAGCTGTGATCTTGAGAGCCCTCTCCTTGGCTTTCCTGCTGAGTCT

CCGAGGAGCTGGGGCCATCAAGGGTGAGTGCTCAGGAGGACGCAGGAGCGTCGGGGTGAG

TGATTGCTGCTTCAGGGATCACAGATTTTAGGGGCTCATTGATCTATCTGGTCCTCATAG

TCTATGTTCCCTCTGGCCCTCATAATAATAACAGCAATAACAGCCAGAATTTATGAGACT

CCTGCATAGTTTCTTTCCCCATTTACATCTCACAGGAATCTTCAATGAAGATAATATTCC

ATTCATTTAGAAATTATTCCTTTTATTTAGAAATTATTTTGAAAAAACTGAAGCTCAAAA

AGATGAATAAGTTTTCCAAGGTTACACAGCAGATCAACGAGCCAAGTTTGAAGTCCAGAC

CCAGCTCTGAGGGTCATACACTGCCTTCCCCAGATTCCTGCACACAGTGACCTACTATCA

GGGCCCTCCTATCTCTCTGGGATCCCCAGCCTCTATCTTTTGTGGCTGCTTTACAGGAAC

TCCGAGCTATGGACTCTGCATTAGGAGACGAAGTGCAAAGAGTGTTTCTGTATCCTCCCT

CTCTTCTAGGACCCTAGGGCTCTTCCTGGGTCTTTGTGGGTGGTCACAAGCTTTCCTCTC

TCAAGACAGCAGGGTTGCATGGTCTTGATAGCCTTGTGATTCGGGTTCTGAGAGATTCAG

GACTGCAAGGGAGGCCTAGACTTTTGATAGCTGCAAGGACTCAGCCAGAGATGGACCGTA

GTGAATGCTCCTTTTTCCTGTAGCTGAAATCAGGGAGAATGACATCAAGCCTGTGCATGA

TGCTGTCATTCCAAAATCTAGTGATGGGGAAGGTTAGAATCCATAACGTACAAGATGCAC

ACTGGCTTCAGACAGTTTTATTTAAGATGTGTAGAATAAAGAGGAGGTCAGGCTGGGTAG

AACCAGAAGTATCTATTGCCCTGTTCGCGGTCACCTGAGTTATTTCTAATGTTATGTTAT

AATAAACACCACAATAGGCTTCTCTTCATAGATGCAAATACTTTTTAGTATTCTTGGTAG

AAATTCCTAATGAGCTCAGCTGTCTCTTCAGGGCTTCCCTGCCCAGTCTCTTAACATTTA

AACATGTCATTTACCTTAAAAACATAAGTGCAAACCAACTGATAAAAAACAACCTTGCCT

TCAGTCTGCATCCTGTCCCAGAGACACTTTCTTTGTGTCCTCACACGTGGAGCTAAGCTT

CTGACTTGTCTCTGGTACATCCCTGAGGATCCTCTCATCTTGGCCATCAGGAACCTCTAC

AGAAGGTCAAATTCAGTGGGTTCTTCTCAGTGCCTCTGACTTGAGTTACTAATAACATTT

GCACTATAATCCACTTCTTTCTGATGAACTACCCTGTCCTTATTTTTCTCCTGTTTACCT

GGATCCTCCTTATCATCTTTTAAACCACCTCTTAACTATCATGTTCTCTCATTATACCCT

GAGATCTCGGCAATTCTGATTTTTGGCACTCTTCCTGGAAAATCTTATTTAACCTGCACC

TGCCACTAATGACTCTCAGTTCTATGGCCTAAATTCCTCTCCTGAGACCACCCATAATCC

ACAAATATCTATGTATTATTTCTCCTTAGATGACTTTCAGGTCTTCTAAGTGCAATAGCC

CCACAGTAAACTCAGTATCTTCTCCCGGTCAGGCTGTCTTCCCTGAGAGAAGTGGCTTTT

GCCCTGTTTTCTGAATGCCTACATTGAAGCCATCTGTACCCCAGGAAGCCTTCCCTGATG

TGCTGTTTGGTCGCATCTTGTGTATACCTACGTATCTGCACTTATCCTTCTGAACCTGCT

GTTGTCCTGTCACTTGTGTTTCCTTCTGTGACTTATACGCGTCTGCAGAACAGGACGTAT

GTATTATTTTTATTTGGGTATTTAGCATCTAACAGTGTTTGACATATAGTAGTCTTTTAA

TACATATTTTTGTCTGAATGGAAATGATATTTTGAAGAAAAATAATCTGTTCCATAGCTG

GCTGATCTTTGGACTGCAGAACTTGTGAAAGTGTTTTTTAAAAAGCATTTTAAAAAGTAC

AAGGGACATTCATGTATTAAGAAGATGAGTTTCCAATAACTGCTAGAGGACTTTGTGTCT

TTTTATTTTACCCTCTTTTTCCTGATGAGTCCTTTGAGTCCTTTAAACTGAGGAGCAAGC

TAAGTTTCCTAGTGAAATACCTATAGGATTTGTTTTGTTTAGTTTCAAATACCACTCTTT

GCTTGGCCACTTACTGTGTCAGGGAGTCATTCTCAGTGAAAAATAAGACACAGGTCATAC

CCTCTAGACACTTACAATTACAGTGGCAAGGAGTCATTCTCCTGTCACTGTAAGTGGCCA

AGCACAGACTGGGTCCCCACATGTCAGGGCTGAAAACTCACAGGGAAATCTGTGAGTTGG

GAGGTGAGAGCAGAAGAGTCCCGTAGTTCCTTCTCACTCTGATGCATTTATCATTCTAAA

CCCAGACTTTCACATACACATTCATCGTTTTCTTTCATGATAATAGTTGCTTTTATCCTC

TTATCTTTGCTAATTCTTACAAACTAATAAAGACTAAGAAACAAAATAAATTAAATCCTA

CAGGTGTTCCAAACTCAGCAATAATTTCTAGTTGGCCTCTAAAACAAAAATCAAAATATA

AATGTAAGAAAAGTTTAGAATGCTTAGTACCTGTGTGATGAAATAATCTGTACACTAAAC

CCCCAAGTCATGAGTTTACCTATACAACAAACCTGCACATGTACTCCTGAACATAAAATA

AATGTTGAAATATTTTTAAAAAGGAAACAAAAGTTTGGAACAAATGCCAAAATAACTGTA

CTGTACTTTTGAATTTATATGCCCCAAATGAAAAATATTATCAACAAAGCTATACATTCT

ACAGTTTCATGTTCATAAACTAAGACAGAAACTTTAAAACTGTCAAGAGCCCTAAAATTT

GAAGGATATTTTCTTCTTCCTCTCAATTTTGTATTTTTTTCTACCTTTTCTATAATAAGA

AAAAGAAAATGTCCATTCCCCCACCCCCATGACTCTAAAAACAATTTTACATCTGTGTCA

TAGAAAAATTAAGATCTTAATGGGAGAGAAAACCTCTCTACTAGTTCCGCCAGTAGCCGT

ATGACCTTAGCAAGTTATTAATATGTAACTTCCCTGCATTTCCTTACCTGTAAAATGTAT

GATATGTATTTGCTTCATAGGGTTATTGTGACAATTCAGCGAGTGAAATATGTAAAGTAT

TTAGAAGGATGCCTGGCACAAGTAAGTGCTCAACAAATGTTAGCTGTCATTGTTACTATT

ACTATTGTGTAGGGTCAGGATGCCCAGACTTTCAAAGACCAGGAAGCAGCTTGACTTATC

AGTGATAAACTTTTCATTTTGTTCTTTGCTCCTTTCTTTTTATAACTGCTCATCTGCTCT

GTATTATTTCCTTTATGGTGTTGCTCCTTCTTCTTCCCCATATGTCCTTCCTTTGACCTC

TTACCTTCTTCCTTTTTATATTCATAAGTCTTTATTCATTCTCTAGCTTTGACCACTTGC

ATATTCAAACTGACATTTTGTCGTGTTTTTCTCTACTGTCTTTATGCAGCGGACCATGTG

TCAACTTATGCCGCGTTTGTACAGACGCATAGACCAACAGGGGAGTTTATGTTTGAATTT

GATGAAGATGAGATGTTCTATGTGGATCTGGACAAGAAGGAGACCGTCTGGCATCTGGAG

GAGTTTGGCCAAGCCTTTTCCTTTGAGGCTCAGGGCGGGCTGGCTAACATTGCTATATTG

AACAACAACTTGAATACCTTGATCCAGCGTTCCAACCACACTCAGGCCACCAACGGTACG

CCCTATCTTTGCCTCTTCCTCTGTAGCCCAACTGGAAGGGATGAGAGGGCCTCTCTGCCA

CCCTCAGACTAGGAAGCCTAAGTGCCCCCTGCTGTGTGATCCTCTTCCCCTAGTGGCCAT

GGGCTGATCCCACTACAGCAAGGGCTTGCATCCTCTCTTCTCAGGAGAGAGAAAGGTGAG

CAGAGTGAGGCTGGTCAGTGGTGTGATACCCCTCTCTGTGATTCAGAGCTGCCATAAAAT

CTAAGGCTGAGGTAGAGGACCACCCTCCCCTAAGAGGTGGAGCCTTTGTGATTCATCCCA

GAAGAGGGGCCTAACCTGGTGCTGTCTCCTTCCAGATCCCCCTGAGGTGACCGTGTTTCC

CAAGGAGCCTGTGGAGCTGGGCCAGCCCAACACCCTCATCTGCCACATTGACAAGTTCTT

CCCACCAGTGCTCAACGTCACGTGGCTGTGCAACGGGGAGCTGGTCACTGAGGGTGTCGC

TGAGAGCCTCTTCCTGCCCAGAACAGATTACAGCTTCCACAAGTTCCATTACCTGACCTT

TGTGCCCTCAGCAGAGGACTTCTATGACTGCAGGGTGGAGCACTGGGGCTTGGACCAGCC

GCTCCTCAAGCACTGGGGTATGCAACTGCTTTTCTCTCCATAATCTCCTGGCATCCTCTA

TTCCAAAGACCTGGTGTCCTCTGCACCAGCTTTCCGCACTGGCTGGGTCTCAGTCCTCTC

CTCGTCCTAACATCCAATTAACTGGTCCATAACCTTCAATTCCCACAACCATCCCAGGCC

ATCACCACCCTCACTGCACCTCCTGACCCTATCTCTTCATTCTTCCCCCAGAGGCCCAAG

AGCCAATCCAGATGCCTGAGACAACGGAGACTGTGCTCTGTGCCCTGGGCCTGGTGCTGG

GCCTAGTCGGCATCATCGTGGGCACCGTCCTCATCATAAAGTCTCTGCGTTCTGGCCATG

ACCCCCGGGCCCAGGGGACCCTGTGAAATACTGTAAAGGTGGGAATGTAAAGAGGAGGCC

CTAGGATTTGTAGAATGTAAGGAAGGGAGGAAAAATTCAATCTGATAAGTGTTCATTGAT

CTTCTAATGGGTTAAAAGCATTCAGCCACATAACAACAACAACACCGATAACTAACTGAG

TAGTTAATATGGTCAGGCGCTATTCTGAGGATTTACATTTATTAACTCACTTTATTCTCA

CACATAGTCTTTGAGGTAGGTACTATTATTTTCACTATTTCACATGAGAGATACTTACAT

CTTTTTACATACACAGAGACTTTAAGCACTTTGATCAAGTTCCCACAGCTATGAAGTAGT

AGGGCTAGCTTCCAATCCAGAAAGTCTGGATCCAAGACTGTTTATCCACTGTCCTATTCA

CCCTATTTTGTGAAGGAAAAGACCAAGTTCAAATTCTCCAGAGTCCATTGCCAAATAATG

GAGTCAGATCTATATTTCTATACATAATTACAACACAGTGTGGTGGGTGCCTGTAACTAC

TTACTGTCTCTACTTGGACTCATTCCATGGCAATGTTCACACAAAAAATGCCTCTCCAGA

GATCTTACAGGTTTCTATTTATCATAACACTCACCATGCTTTATATTTTTATATGTTTTG

GGAATTCTCTTAGCATTAGACAGTGAACTTCCATGCAGATGACCACATCTAATTCATTAT

TATTATTGTTATTCATGCTGGACCTCAGGTACAAAAGGTTAAGAACTTCTCAGTTCATTA

TATGATCATCATTGGTGCCTCCGAGCTCTCTCTCTCTCCCTTGATTTATTTGGTCCCTTT

TATCTCCAGTCCTTACTCCCATATCTAACCTCTTACCCCTACCTCATAGGTAAACATTTT

AATGAATTTGATGTTTCCTTTTATTTGCATAGATCCTCTGTAATATGTAGTAGTGTCCAG

TGTACATGTATTTTTAATTAACCAAAATGGCATTAAATTATAGATCTAATTTTGTACATC

CAGTTTGTTTCTTCCAAATCTTCCATAGTATTTTACTTTATATGTCCATGCATTAGTCCA

TTTTGCATTGCTATAAAGGAATATCTGAAGTTACCTAATTTACGAAGAAAAGAGCTTTAA

ATGGCTCACAGATCTGCAGGCTGTACGCGAAACATGGCACTAGCATCTGCTTCTGTTGGG

GGATTCTGGAAGCTTTTACTCATGGTGGAAGGCAAGTGGAGCCAGTGCATCACATGGTCA

TAGAGGGAGAAAGAGACATAGAAAGAGGTGCCAGCCTCTTTTTAACAACCAGGTTTCATG

TGCACTAATAGAGTGAGAACTCACTCATTACCCGGAGAGGGGACAAAGCCATTCATGAGG

GTCTCCTCCATGATTCAAATACCTCCCACCAGGCCCCACCTGCAACACTGGGGATCAATT

TTCAACATGAGACTTGGAAGTGACAAATATCCAAATCATATTAATCCACATATCTACATT

GCTCCTGGGATACCTGGATCATTCCTGGTTCTCTACTATTGCAAGCAATGCTTGTATCTC

ACATGGAACTGCATATACATGTGGGCCTGACCTGCATCCCTGGAATGTATGTATCCTAGA

AAGGGGTTGCAGGGTTGTTGGAGATGCAGCTCCTTAATTTGACTAAACACTGCTCATCTT

CTCATCAGAATGGCTGTACTCATCTGAACTTCCTTTGTCAGTACTCTAATTGTCCTGCAA

CTCCTAAATGGACTTCAACACTGGACATTATCCAGTTTTCTAACTTTTGCCAATTTCATG

TGCATAAAGAAATATGCTGTTTTATTTTGCATTTCTTTAATTACTAATAATTGGGGCTAT

AATTAGGACTGATTAGCCACTTGGGGGTTCCTTTTCTATAAATTGCCTGTTCACATTCAT

TGTCCATTTTTGTACTATGTGCTTCCATCATTTTCTTATTGATTTGCAGGTGATCCTTAT

ATAGTCCTGCTAGTAGTCCCTTGTCAGTTTTAGGCATTGCAAATGTTTTCCTCTAATCTG

ACTTCTGGCAACTGTCTCCTTGGTTTCCTTTATTGAAGAGAAATCCTTAATATTTTGTAA

TGAAGTCCATCAACTGTATTTTTGTTTGTGTGTCTTTTTTAAAAGAAGTCTTCCCTATAC

TGAGATATCAAAGATACTCTTAAAACATCTCCTACAGTTTTAAATTTCACATTTACTACT

TTAATTCATCTGGGATTCATCTTTGTGTTTGATGGGGATCATGTTTTATTTTTCTTTATA

TAATGGGCCAGTGTGTTCCCACAACTACTAAATAGTTCACCTTTTCCCCATAGGTTAGTA

GTGTCTCCTTTGCTATACTGAAAGCTCCCATTATAGGTGGGCCTGTGTCTGAGTTCCATC

TTGTTCCACTGTTCTGTTTGTCTCTTCTTGTGCCAGTGTCCTAGTATTTTGATTACTATG

ACATTGTAGTGTGTGTTAGTATCCAGTAGGACAAATTCTTGTTTATTTTTCTTAGTTCAC

ACACATTTATAATTATATCTATAATGATTTGTAACAGAGTGAAGTGAATGTAGAATGTCA

GATGTTAAGAGGAAGAATGGAAAAGAGGGCTGGGACTAGGGTGATGTAGGGGATGCACTT

GGCTTAGGTGCAAAATTTGGGGGATACCAAAAGAACTCAGTAATAAATCATATTTTAATG

AAATATCTTGAAAAGGCAAAATTAATGCAAAGATACATGATTAACAAAACATCCAAAGAG

GAGTATTTAACAAAAATGGAGAAGCAGAGAAGCAGAAGAATTAGGAGAATATGCTGTCAC

ATGAGCCAAGGAATTAAAGAATTCAGGAAGGAGGAAGTACTGCTGTCAGATGTTCAACAG

AGGTCATTTTAGAAAATTTACCTTGGTTTTTGAAATCCTTTCAAAGAGCAGTATACACAA

TGTGAGCAAGTATCCTTCGTTCATTGCCGTCATTGATATGGTTTGGATATTTGTCCCTTC

CAATTCTCATTCCAGGGTTAAGCTTCTTCTCTGCCCTCAGTAATGTGGCCCTTCCCCTTG

TCTGTATATTTTGGAGACATGAAGCATGTGGGATGGCCTCACAGTCAGCTGGGGTTTGAG

GGTGAAATTCAATGACTTTCGTGAACTCCTTGGCTCCTATGTGCTCTTCACCTGGAGGAC

CAGGGCATGTGCAGGGATGACCACCTTCTCCCTGGGACCTGAACAGGGCAGAGAAATGGG

AAGCTCGGGTGCAAAGGGAGTGGGGAAGATGGGTCCGGGCTTACAGTACTGAACCCAGGA

ATGACAATAACTGTGTGTGTTGCTGCAGGTGACAAAATATCTGAACAGAAGAGGACTTAG

GAGAGATCTGAACTCCAGCTGCCCTACAAACTCCATCTCAGCTTTTCTTCTCACTTCATG

TGAAAACTACTCCAGTGGCTGACTGAATTGCTGACCCTTCAAGCTCTGTCCTTATCCATT

ACCTCAAAGCAGTCATTCCTTAGTAAAGTTTCCAACAAATAGAAATTAATGACACTTTGG

TAGCACTAATATGGAGATTATCCTTTCATTGAGCCTTTTATCCTCTGTTCTCCTTTGAAG

AACCCCTCACTGTCACCTTCCCGAGAATACCCTAAGACCAATAAATACTTCAGTATTTCA

GAGCGGGGAGACTCTGAGTCATTCTTACTGGAAGTCTAGGACCAGGTCACATGTGAATAC

TATTTCTTGAAGGTGTGGTTTCAACCTCTGTTGCCGATGTGGTTACTAAAGGTTCTGATC

CCACTTGAACGGAAAGGTCTGAGGATATTGATTCAGTCCTGGGTTTTTCCCTAACTACAG

GATAGGGTGGGGTAGAGAAAGGATATTTGGGGGAAATTTTACTTGGATGAAGATTTTCTT

GGATGTAGTTTGAAGACTGCAGTGTTTGAAGTCTCTGAGGGAAGAGATTTGGTCTGTCTG

GATCAAGATTTCAGGCAGATTAGGATTCCATTCACAGCCCCTGAGCTTCCTTCCCAAGGC

TGTATTGTAATTATAGCAATATTTCATGGAGGATTTTTCTACATGATAAACTAAGAGCCA

AGAAATAAAATTTTTAAAATGCCCTAATTCATTGCAATTTTTACCAGCCATAGTCACTCC

ATGTGGGAGAACTTAAATCATGATTACCAGAGCTTTCAAAGGTTTGAGAATAGTGATGAT

TATGAAGAAAAATATCTTATTTGAGCAAGGATTTTGTTTCTTTATGAGTGTTCATTAGAT

ATTACGATGAAAAAAGCATGAAATGGTAAAAATTCAGATAAATATAAAAACATGTTCTCT

AGTTTTTTTTAAGTTAAAAAAGGAATTGTTTAAAGTA

>DPA1_in_KAS116

CACTTTAAACAATTCCTTTTTTAACTTAAAAATAAAAAGAAACCTAGAAAACATGTTTTT

ATATTTATCTGCATTTTTACCATTTCGTGCTTTTTTCATCATAATATCTAATGAACACCC

ATAAAGAAACAAAATCCTTGCTCAAATAAGATATTTTTCTTCATAATCATCACTATTCTC

AAACTTTTGGAAGCTCTGGTAATCATGATTTAAGTTCTCCCACATGGAGTGACTATGGCT

GGTAAAAATTGCAATGAATGAGGGCATTTTTAAAATTTTATTTCTTGGCTCTTAGTTTAT

CATGTAGAAAAAATCCTCCATGAAATATTGCTATAATTACAATCCAGCCTTGGGAAGGAA

GCTCAGGGGCTGTGAATGGAATCCTAATCTGCCTGAAATCTTGATCCAGACAGACCAAAT

CTCTTCCCTCAGAGACTTCAAACACTGCAGTCTTCAAACTACATCCAAGAAAATCTTCAT

CCAAGTAAAATTTCCCCCCAATATCCTTTCTCTGCCCCACCCTATCCTGTAGTTAGAGAA

GAACCCAGGGCTGAATCAATATCCTCAGACCTTTCCATTCAGGTGGGATCAGAACCTTTA

GTAACTACATCTGCAATAGAGGTTGAAACCACACCTTCAAGAAATAGTATTCACATGTGA

CCTGGTCCTAGACTTCCAGTAAGAATGACTCAGAGTCTCCCAGCTCTGAAATACTGAAGT

ATTTATTGGTCTTAGAGTATTCTCAGGAAGGTGACAGTGAGGGGTTTTTCAAAGAAGACC

AGAGGATAAAAGGCTCAATGAAAGGATAATCTCCATATCAGTGCTATCAAAGTGTCAACA

TTTATTCATTAATTTCTATTTGTTGGAAACTTTACTAAGGAATGACTGCTTTAAGGTAAC

GGATAAGGACAGAGCTTGAAGGGTCAGCAATTCAGTCAGCCACTGGGGTAGTTTTCACAT

GAAGTGAGAAGAAAAGCTGTGACGGAGTTTGTAGGGCAGCTGGAGTTCAGATCTCTCCTA

AGTCCTCTTCTGTTCAGATATTTTGTCACCTGCAGCAACACACACAGTTACTGTCATTCC

TGGGTTCAGTACTGCAAGCCCAAACCCATCTTTCCCACTCCCTTTGTACCCGAGCTTCCC

ATTTCTCTGCCCTGTTCAGCTCCCAGGGAGAAGGTGGTCATCCCTGCACAAACCCTGGTC

CTCCAGGTGAACAGCACATAGGAGCCAAGGAGTTCACTAAAGTCATTGAATTTCACCCTC

AAACCCCAGCTGACTTTGAGGGCATCCCACATGCTTCATGTCTCCAAAATATCCTGACAA

GGGGAAGGGCCACATTACTGAGGGCAGAGAAGAAGCTTAACCCTGGAATGAGAATTGGAA

GGGACAAATATCCAAACCATATCAATGATGGCAATGAAGGAAGGATACTTGTTCACATTG

TGTAAACTGCTCTTTGAAAGGATTTCAAAACCAAGGTAAATTTTCTAAAATGACCTCTGT

TGAACATCTGACAGCAGTACTTCCTCCTTCCTGGAATTCTTTAATTCCTTGGCTTATGTG

ACAACATATTCTCCTAATTATTCTGCCTCTCTGCTTCTTCTCCATTTTCGTTGAATAATC

CTCTTTTGATATTTTGTCAATCATGTATCTTTGCATTAATTTTGCTTTTTCAAGATATTT

CATTAAAATATGATTTATTACTGAGTTCTTTTGGCATCTCCCAAATTTTGCACCTAAGTC

AAGTGCATCCCTTACATCACCCTAATTCCAGCCCTCCTTTCCATTCTTCCTCTTAACATC

TGACATTCTACATTCACTTCATTCTGTTACAAATCATTATAGATATCATTATAAATGTGT

GTGAACAAAGAAAAATAAAGAAGAATTTTTCCTACTGGATACTAACACACACTACAATGT

CATAGTAATCAAAACACTAGGACACTGGCACAAGAAGAGACAAACAGAACAGTGGAACAA

GATGGAACTCAGACACAGGCCCATCTGTAATGGGAGCTTTCAGTATAGCAAAGGAGACAC

CACTAGCTTATGGGGAAAAGGTGAACTATTTAGTAGTTGTGGGAACACACTGGCCCATTA

TATAGAGAAAAATAAAACATGATCCCCATCAAACACAAAGATGAATCCCAAATGAATTAA

AGTAGTACATGTGAAATTTAAAACTGTAGGAGATGTTTTAAGAGTATCTTTGATATCTCA

GTACAGGGAAGATTTCTTTTAAAAAAGATGCATAAACAAAAATACAGTTGATGGACTTCA

ACACAAAATATTAAGGATTTCTCTTCAATAAAGGAAACCAAGGAGAAAGTTACCAGAAGT

CAGATTAGGGGAAAACATTTGCAATGCCTAAAACTGACAAGGGACTACTAGCAGGACTGT

GTAAGTATCACTTGCAAATCAATAAGAAAATGATAGAAGCACATAATACAAAAATGGGCA

ATGAATGTGAACAGGCAATTTATAGAAAAGGAACCCCCAAGTGGTTAATCAGTCCTAATT

ATAGCCCCAATTATTAGTAATTAAAGAAATGCAAAATAAAACAGCATATTTCTTTATGCA

AATGAAATTGGCAAAAGTTAGAAAACTGGATAATGTCCAGTGTTGATGTGCATTTAGGAG

TTGCAGGACATTTCAGGTACTGACAAAGGAAGCACAGATGAGTACAGCCATTCTGATGAG

AAGATGAGCAGTATTTAGTCAAATTAAGGAGCTGCATCTCCAGCAACCCTGCAACCCCTT

TCTAGGATACATACATTCCAGGGATGCAGGTCAGGCCCACATGCATATGCAGTTCCATGT

GAGATACGAGCATCGCTTGCAATAGTAGAGAACCAGGGGTGATCCAGGTATCCCAGGAGC

AATATAGATATGTGGATTAATATGATTTGGATATTTGTCCCTTCCAAATCTCATGTTGAA

AATTGATACCCAGTGTTGCAGGTGGGGCCTGGTGGGAGGTATTTGAATCATGGAGGAGAC

TCCCTCATGAATGGCTTGGTGCCCGCCCCAGGGTAATGAGTGAGTTCTCACTCTATTAGT

GCACGTGAAACCTCGTTGTTAAAAGAGCCTGGCACCTCTTTCTATGTCTCTTTCTCCCTC

TATGACCATGTGATGCACTGGCTCCACTTGCCTTCCACCATGAGTAAAAGCTTCCAGAAT

CCCCCAACAGAAGCAGATGCTGGTGCCATGTTTCATGGACAGCCTGCAGATCTGTAAGCC

ATTTAAAGCTCTTTTCTTCATAAATTAGGTAACTTCAGATATTCCTTTATTCCTTTATAG

GAATGCAAAATGGACTAATGCATGGACATATAGAGGAAAATACTATGGAAGATTTGGAAG

ATACAAACTGGATATACAAAATTAGATCTATAATTTAATGCCATTTTGGTTAATTAAAAA

TACATGTACACTGGACACTACTACATATTAGGGAGCATCTATGCAAATAAAAGGAAACAT

CAAATTCATTAAAATGTTTACCTATGAGGTAGGGGTAAGAGGTTAGATATGGGAGTAAGG

ACTGGAGATAAAAGGGACCAAATAAATCGAGAGAGAGAGAGAGTTCGGAGGCACCAGTGA

TGACAGTATAATGAACTGAGAAGTTCTTAACCTTTTGTACCTGAGGTCCAGCATGAATAA

CAATAATAATAATGAATTAGATGTGGTCATCTGCATGGAAGTTCACTGTCTAATGCTAAG

AGAATTCCCAAAACATATAAAAATATAAAGCATGGTGAGTGTTACGATAAATAGAAACCT

ATAAGATCTCAGGAGGGGCATTTTTTGTGTGAGCATTGCCATCGAATGAGTCCAAGTAGA

GACAGTAAGTAGTTACAGGCTCCCACCACACTGTGTTGTAATTATGTATAGAAATATAGA

TCTGGCTCCGTTATTAGACAACGGACTCTTGGAGAATTTCAACTTGGTCTTTTCCTTCAC

AAAATAGGGTGAATAGGACAGTGGATAAACAGTCTTGGATCCAGACTTTCTGGATTGGAA

GGTAGCCCTACTACTTCATAGCTGTGGGAACTGGATCAAAGTGCTTAAGATCTCTGTGTA

TGTAAAAAGATGTAAGTATCTCTCATGTGAAATGGTGAAAATAATAGTACCTACCTCAAA

GACTATTTGTGAGAATAAAGTGAGTTAATGAATGTAAACCCTCAGAATAGCGCCTGACCA

TATTAACTACTCAGTTAGTTATCAATGTTGTTGTTGTTATGTGGCTGAGTGCTTTTAACC

CATTAGAAGATCAATGAACACTTATCAGATTGAATTTTTCCTCCCTTCCTTACATTCTAC

AAATTCTAGGACCTCCTTTTTTACATTCCCACCTTTACAGTATTTCACAGGGGCCCCTGG

GCCCGGGGGTCATGGCCAGAACGCAGAGACTTTATGATGAGGACGGTGCCCACGATGATG

CCCACTAGGCCCAGCACCAGGCCCAGGGCACAGAGCACAGTCTCCGTTGTCTCAGGCATC

TGGATTGGCTCTTGGGCCTCTGGGGGAAGAATGAAGAGATAGGGTCAGGAGGTGTAGTGA

GGGTGGTTCTGGCCTGGGATGGTTGTGGGAATTGAAGGTTATGGACCAGTTAATTGGATG

TTAGGACGAGGAGAGGACTGAGACCTGGCCAGTGGGGAAAGCTGGTGCAGAGGACACCAG

GTCTTTGGAATAGAGGATGCCAGGAGATTATGGAGAGAAAAGCAGTTGCATACCCCAGTG

CTTGAGGAGCGGCTGGTCCAAGCCCCAGTGCTCCACCCTGCAGTCATAGACGTCCTCTGC

TGAGGGCACAAAGGTCAGGTAATGGAACTTGTGGAAGCTGTAATCTGTTCTGGGCAGGAA

GAGGCTCTCAGCGACACCCTCAGTGACTGGCTCCCCATTGCACAGCCATGTGACGTTGAG

CACTGGTGGGAAGAACCTGTCAATGTGGCAGATGAGGGTGTTGGGCTGGCCCAGCTCCAC

AGGCTCCTTGGGAAACACGGTCACCTCAGGGGGATCTAGGAGGAGACAGCACCAGGTTAG

GTCCCTCTTCTGGGATGAATCACAAAGGCTCCACCTCTTGGGGGAGGGTGGTCCTCTACC

TCAGCCTTAGATTTTATGGCAGCTGTGAATCACAGAGAGGGGTATCACACCACTGACCAG

CCTCACTCTGCTCACCTTTCTCTCTCCTGAGAAGAGAGGATGAAAGCCCTTGCTGTGGTG

GGATCAGCCCACGGCCACTAGTGGAAGAGGATCACACAGCAGGGGGCACTTAGGCTTCCT

AGTCTGAGGGTGGCAGAGAGGCCCTCTCATCCCTTCCAGTTGGGCTACAGAGGAAGAGGC

AAAGATAGGCAGTACCATTGGCGGCCTGAGTGTGGTTGGAACGCTGGATCAAGGTATTCA

AGTTGTTGTTCAATATAGCAATGTTAGCCAGCCCGCCCTGAGCCTCAAAGGAAAAGGCTC

GGCCAAACTCCTCCAGATGCCAGACGGTCTCCTTTTTATCCAGATCCACATAGAACTGCT

CATCTTCATCAAATTCAAACATAAACTCCCCTGTTGGTCTATGGGTCTGTACAAACGCGG

CATAAGTTGACACATGGTCCGCTGCATAAAGAAAGTAGAGAAAAACATGACAAAATGTCA

GTTTGAATATGCAAGTGGTCAAAGCTAGAGAATGAATAAAGACTTATGAATATAAAAAGG

AAGAAAGTAAGAGGTCAAAGGAAGGACATATGGGGAAGAAGAAGGAGCAACACCATAAAG

GAAATAATACAGAGCAGATGAGCAGTTATAAAAAGAAAGGAGTGAAGAACAAAATAAAAA

GTTTATCATTGATAAGTCAAGCTGCTTCCTGGTCTTTGAAAGTCTGGGCATCCTGACCCT

ACACAATAGTAAAAATAACAATGACAGCTAACATTTGTTGAGCACTTAGTTGTGCCAGGC

ATCCTTCTAAATACTTTACATATTCACTCATTGAATTGTCACAATAACTTTATGAAGCAA

ATACATATCATACATTTTACAGGTAAGGAAATGCAGGGAAGTTACATATTAATTACTTGC

TAAGGTCATATGGCTACTGGTGGAACTAGTAGAGAGGTTTTCTATCCCATTAAGTTCTTA

ATTCTTCTAGGACACAGATGTAAAATTGTTTTTAGAGTCGTGGGGTTGGGGGAGTGGACA

TTTTCTTTTTCTTATTATAGAAAAGGTAGAAAAAAATACAAAATTGAGAGGAAGAAGAAA

ATATCCTTCAAATTTTAGTGCTCTTGACAGTTTTAAAGTTTCTGTCTTAGTTTATGAACA

TGAAAATGTAGAATGTATAGCTTTGTTGATAATATTTTTCATTTGGGGCATATAAATTCA

AAAGTACAGTACAGTTATTTTGGCATTTGTTCCAAACTTTTGTTTCCTTTTTAAAAATAT

TTCAACATTTATTTTATGTTCAGGAGTACATGTGCAGGTTTGTTGTATAGGTAAACTCAT

GACTCGGGGGTTTAGTGTACAGATTATTTCATCACGCAGGTACTAAGCATTCTGAACTTT

TCTTACATTTATATTTTGATTTTTGTTTTAGAGGCCAACTAGAAATTATTGCTGAGTTTG

GAACACCTGTAGGATTTAATTTATTTTGTTTCTTAGTCTTTATTAGTTTGTAAGAATTAG

CAAAGATAAGAGGATAAAAGCAACTATTATCATGAAAGAAAATGATGAATGTGTATGTGA

AAGTCTGGGTTTAGAATGGTAAATGCATCAGAGTGAGAAGGAACTATGGAGCTCTTCTGC

TCTCACTTCCCAACTCACGGATTTCCCTGTGAGTTTTCAGCCCTGACATGTGGGGACAGG

AGAATGACTCCTTGTCACTGTAATTGTAAGTGTCTAGAGGGTATGATCTATGTCTTATTT

TTCACTGAGAATGACTCCCTGACACAGTAAGTGGCCAAGCAAAGAGTGGTATTTGAAACT

AAACAAAACAAATCCTATAGGTATTTCACTAGGAAACTTAGCTTGCTCCTCAGTTTAAAG

GACTCAAAGGACTCATCAGGAAAAAGAGGGTAAAATAAAAAGACACAAAGTCCTCTAGCA

GTTATTGAAAACTCATCTTCTTATTACATGAATGTCCCTTGTACTTTTTAAAATGCTTTT

TAAAAAACACTTTCACAAGTTCTGCAGTCCAAAGATCAGCCAGCTATGGAACAGATTATT

TTACTTCAAAATATCATTTCCATTCAGACAAAAATATGTATTAAAAGACTACTATATGTC

AAACACTGTTAGATGCTAAATACTCAAATAAAAATAATACATACGTCCTGTTGTGCAGAC

GCGTATAAGTCACAGAAGGAAACACAAGTGACAAGACAACAGCAGGTTCAGAAGGATAAG

TGCAGATACGTGGGTATACACAAGATGCGATCAAACAGCACATCAGGGAAGGCTTCCTGG

GGAACAGATGGCTTCAATGTAGGCATTCAGAAAACAGGGCAAAAGCCACTTCTCTCAGGG

GGAAGACAGCCTGACTGGGAGAAGATACTGAGTTCACTGTGGGGCTATTGCACTTAGAAG

ATCTGAAAGTCATCTAAGGAGAAATAATACGTAGACATTTGTGTATGATGGGTGGTCTCA

GGAGAGGAGTTTAGGCCAGAGAACTGAGAGTCATTAGTGGCAGGCGCAGGTTAAATGAGA

TTTTCCAGGAAGAGTGCCAAAAATCAGAATTGCCGAGATCTCAGGGTATAATGAGAGAAC

ATGATAATTAAGAGGTGATTTAAAGGATGATAAGGAGGATCCAGGTAAACAGGAGAAAAA

TAAGGACATGGTAGTTCATCAGAAAGAAGTGGATTACAGTGCAAATGTTATTAGTAACTC

AAGTCAGAGGCACTGAGAAGAACCCACTGGATTTGACCTTCTGTAGAGGTTCCTGATGGC

CAAGATGAGAGGATCCTCAGGGATGTACCAGAGGCAAGTCAGAAGCTTAGCTCCACGTGT

GAGGACACAAAGAAAGTGTCTCTGGGACAGGATGCAGACTGAAGGCAAGGTTGTTTTTTA

TCAGTTGGTTTGCACTTATGTTTTTAAGGTAAATGACATGTTTAAATGTTAAGAGACTGG

GCAGGGAAGCCCTGAAGAGACAGCTGAGCTCATTAGGAATTTCTACCAAGAATACTAAAA

AGTATTTGCATCTATGAAGAGAAGCCTATTGTGGTGTTTATTATAACATAACATTAGAAA

TAACTCAGGTGACCGCGAACAGGGCAATAGATACTTCTGGTTCTACCCAGCCTGACCTCC

TCTTTATTCTACACATCTTAAATAAAACTGTCTGAAGCCAGTGTGCATCTTGTACGTTAT

GGATTCTAACCTTCCCCATCACTAGATTTTGGAATGACAGCATCATGCACAGGCTTGATG

TCATTCTCCCTGATTTCAGCTACAGGAAAAAGGAGCATTCACTACGGTCCATCTCTGGCT

GAGTCCTTGCAGCTATCAAAAGTCTAGGCCTCCCTTGCAGTCCTGGATCTCTCAGAACCC

GAATCACAAGGCTATCAAGACCATGCAACCCTGCTGTCTTGAGGGAGGAAAGCTTGTGAC

CACCCACAAAGACCCAGGAAGAGCCCTAGGGTCCTAGAAGAGAGAGAGGATACAGAAACA

CTCTTCGCACTTCCTCTCCTAATGCAGAGTCCATAGCTCGGAGTTCCTGTGAAGCAGCCA

CAAAAGACAGAGGCTGGGGATCCCAGAGAGATAGGAGGGCCCTGGTAGTAGGTCACTGTG

TGCAGGAATCTGGGGAAGGCAGTGTACGACCCTCAGAGCTGGGTCTGGACTTCAAACTTG

GCTCGTTGATCTGCTGTGTAACCTTGGAAAACTTATTCATCTTTTTGAGCTTCAGTTTTT

TCAAAATAATTTCTAAATAAAAGGAATAATTTCTAAATGAATGGAATATTATCTTCATTG

AAGGTTCCTGTGAGATGTAAATGGGGAAAGAAACTATGCAGGCGTCTCATAAATTCTGGC

TGTTATTGCTGTTATTATTATGAGGGCCAGAGGGAACATAGACTATGAGGACCAGATAGA

TCAATGAGCCCCTAAAATCTGTGATTCCTGAAGCAACAATTGATGTGAACCACCCCATCA

CTCACCCCGACGCTCTTGCGTCCTCCTGAGCACTCACCCTTGATGGCCCCAGCTCCTCGG

AGACTCAGCAGGAAAGCCAAGGAGAGGGCTCTCAAGATCACAGCTCTGATATGGAACATT

CTGTCTTCAGGGCGCATGTTGTGGGGTCTATAATTGATGACTGTGAGCACAGGAACAGTG

ATGAGGAACTGAGGCCGAGTGGAGGCAGATGAGACTGAAACTGTGGGCCTCTAGCACTGG

AAATGGGTGGAGAGGAATCAGCATGGCTGGGATTCACCTATCAGAGAAATCATAGAGCTG

ACATTCTCTGTTGCTGGGTAAAGAGGACGCTGGAAGGTGCTGGGGAAGAGATGGGAGAAT

TTTAGGTACCAGCGTGGTCAAGAGAGCTCCAGTTCACAGTTCATTTTCAGAGTTAGAGAA

AGAGATGTAAAAAGATAAGTTACACCTTCTTCTGACGGCAAATGTTTTCCATTATGTTCC

TTCTCCCGAGCCCCACCCCCATCCCAGACAGTCAGATGATCTTTGATGTTTTTTGGTCAC

TATATTTTAAATCATGTTTTATGTTATGTTGTCAATATTTTACAAAAATATTCTGCTGAT

AATTAAGAATGAATGTGCTATCTAATAAAATATATAATTAATCTTTCTTTCAGGTCCACC

>HLA:HLA00517 DPB1*02:01:02:01

TAATCCCTGTAGATGGGCCAGCAGAATATTTGAGATCACCTTCAGAGCAAAGAAAACGCA

TAATCTCGCCAAACATCATGACTTATCTGACTGGTTAAAATGAGTATCACTGTCTTTCCT

CCGTCATCTTAAGTGCATCACAGGCTTTATATTTTCAGACCTTTCATACTAACTTTCTGC

CTAGTGAGCAATGACTCATACAAAGCTCAGTGTCCATTGGTTCTTTTCTCAGACTCTGTC

CAATCCCAGGGTCACAGAAGACTACTTGGGTTCATGGTCTCTAATATTTCAAACAGGAGC

TCCCTTTAGCGAGTCCTTCTTTTCCTGACTGCAGCTCTTTTCATTTTGCCATCCTTTTCC

AGCTCCATGATGGTTCTGCAGGTTTCTGCGGCCCCCCGGACAGTGGCTCTGACGGCGTTA

CTGATGGTGCTGCTCACATCTGTGGTCCAGGGCAGGGCCACTCCAGGTAAGAGCCGAACT

GCCATTCTTGGAGGGTCTGGCTCAGGGAACAATTCCTAGGGGACGTTATCTTTAAGGGAT

CAAATTCTGAGACAGGCTGCGGGGGCTCCTGCCCTAAGGCAGTGTCCTCTCTTCCCAGCT

AGAGAAAGAGGTTCATCCCCTATAGGATAGCTTGCTACCCTACTGGCCTATTCTCTCTCC

AAGGACATGGGTACAGTAAACAGAGAGAGGTGCCCAGTGGTCAGTATGCTTGTCTTTGGG

GAAAATGGGACCAAGAGGTCCTGGATAACCTTGGACAGACAAGGTTTGCAGAGAGAGAAG

TTGGCAAGTGCAGGCTCCTGGGCGTGTTCATGTCTGCATCCAGCCTGGAGGGGACTCAGG

CAGAGAGCCCTAAGCTGGAGTGTCCAGGCTCTGAGGATCACTGAGGATTCAGTGCTCACG

AAGAATGCCTCTTATTCCCCAGGGTGGAGCAGGAGCCCACATCCCTTGGACAATTAAGGA

GAGAAGGGAGGGAGGGGGATAGGTTTTAGCCCCTGAAGGCATTCTCATTAAAGGTACTTC

TCCCAGCCTCCCCAGAACTTGGTTAGGGTACTAGAGTGGGTTGCGACTTGTAGGAAGAAT

GAGATGAGGTTGTGTGGGTGCATGACAGGGATTGAGTGTAGGTTATCAGACAGCCAAGGA

AGCAGTAACCAAGTGAAAAATCTCTTCTTCCTGCTGCCTCCCTGTGGCTGGTGTAATATT

ATGGCATCTATGATCCATTGTTTTTCTCTCAGGATACTCTCAGGATATTTCTTTTTATAT

ATATATATACTTTAAGTTCTAGGGTACATGTGCACAACGTGCAGGTTTGTTACATATGTA

TACATGTGCCATGTTGGTGTGCTGCACCCATTAACTCGTCATTTACATTAGGTATATTTC

CTAATGCTATCCCTCCCCCCTCCCCCCACCCCACAACAGGCCCCGGTGTATGATGTTCCC

CTTCCTGTGTCCATGTGTTCTCATTGTTCAGTTCCCACCTATGAGTGAGAACATGTGGTC

TTTGGTTTTTTGTCCTTGCAATAGTTTGCTGTGAATGATGGTTTCCAGCTTCCTCCATGT

CCCTACAAAGGACATGAACTCATCCTTTTTTATGGCTGCACAGTATTCCATGGTGTATAT

GTGTGCATTTTCTTAATCCAGTCTATCACTGATGGACAGTTGGGTTGGTTCCAAGTCTTT

GCTATTGTGAATAGTGCCGCTATAAACATATGTGTGCATGTGTCTTTATAGCAGCATGAT

TTATAATCCTTTGGGTATATACCCAGTAATGGGATGGCTGGGTCAAATGGTATTTCTAGT

TCTAGATCCTTGAGGAATTGCCACACTGTCTTGAGATACCATCTCACACCAGTTAAAATG

GCGATCATTAAAAAGTCAGGAAACAACAGGTGCTGGAGAGGATGTGGAGAAATAGGAACA

CTTTTACTCTGTTGGTGGGACTGTAAACTAGTTCAACCATTGTACTCTCAGGACATTTCT

AGTCCAAATTTACACCAACACTCTGAGAGGAAGGACTGCAAAGTAGGTACCTTAGTTTTC

CACTGACTTCCACTTTTCCTGCTTACACCCTTCCTCCTAGACCTCTCCACACCCCTCCTA

GGACACACCTAAAAGGTACTGACATCATGTCACCTCCTCATCTTTCAGGGTAGCAAGGTT

GGAATCTCCTGAATACAGCCCCTCAAGCCCTAAAACCTCTTATCTATTACCTTGGGTTCA

TTGTCCAGGAAGGGGAGGAGAACTTGAACTTGTAGTCACAGAAGGGTGCTGAGAACTAAC

CAGCAGGACGGCTCAGCCCTGGGAACTGCAGAGGGGTGAGGCTGGGGAGAGAGGAGGCTG

GAGCAGCACTGGTGACACTGAACAGTGTCAGGAGGAAGTGACGGATGCAGCGCCCCCATC

CCATAGGCAGAGCTGTCATGTGGGATGAGGGACAGTGTTGGGAGCCACCAAGGAAACCCA

GAGGTGGGGGAGCAGAGAGCAGAAGGGAGCATGTGATGCTGGACAGTGAAAGGGAGGACA

GGCAAAGGCTGGGTTGAGGTTTGTAGGGGGAATGAGATGAGGCAGTGGAGCCATGTGACA

GGGACTGAGGGTAGATTACTGGAGCTCCCTGCGTAGAATGAATGTTCAATCAAAATTTGC

TGGAGGGAGAGCTGGAGCCATAGGGGAGTGGGTAAAGTGGGCAGGGCTGATTCCACAATT

CCCTGCATGCTCCCCCAACTCCACACACATCCCCAACCTCAAACAGGGCACAAGACCAAA

GGGCTGAGGAGCCAGGCTATAGCTTAAAGAGGCTGGGGGAGAAAAGCTTGGCTGAGACAA

CCCATAGGGAGCTAGAGGTTTTTAATATATCCTATTCTGAATAAGAGACGAATTCATTCA

GATCAGTGGTTTCAAACCGTGCTCTGGGCAACTCAATTGCTAAGGGTTCCACAAACAGGA

TAAAGTTTCTTATATACAAAAAAAAATGAAGGTTTCAAATTACACCATAAAACCCCTCAT

TGCTTATGTCTACTTGGCAGGTAAAATTCCATTTCAAAAGTTAAATGTACTTAAAAAATT

ACCTAAGACTGGGTAAATTAAAAAAATTAAATGTTGCAAAGAAAAAATTCAAAATTCTTA

TTCTTGAATGAAAAACGTTCTCTTACTGGTGATTGAGGAGGAGAAACAAAGACTAACAAA

TGAAAATGGGAGAATCCACACTCAGAGTGGGGCAACTGAACAGGCAGGGGCGGATGGATG

GCAGAGGAGGAGGAATCTGGACTCAAGGAGCTGGGGGGCTCTGGGCCTGGAATTTTAGGG

TCTGGGGCCCAAGGCACCAGGAGAAGAGGCAGGTCAGGATATCTGAGTCAAGACCTGGGA

TCTTGCCTTAGCAATGACACTGGAGACTAAAGGTGGACTCCATGGTGCCCTTGAGCCCAG

CCCTACCCCATCTCCACTATCCTCTGCCACCAGCTGTGCAACTTCTGCTAGGGGTGAGGT

TAATAAACTGGAGAAGTTAATTTGTGGAGCATGAAACAGATGAGCAGAACAATCACAGCA

CCTTAATTTCCCCAGTGTGCCCAAGAACAGAGCAGGCCTGAAGATACTCAAACAGAAACA

AACATGTGCCGTGTCACTGATAATTCTGTGTAGACACACACCTGCCAGACACTGCTCATG

GCACTCCCTAGGAAGAACAGCATGTGGGAAAGGCTGCCAAAATTGTTCATGTAAAAATTA

CATCAATGCTGTCTTCCTCGGTGCTGCCTATGCAGCTGGCAGCCATCTCTTCCTCCACAT

CATGGCCTCCCTCAGACTCCTCATGAAGGATAAGATCCTCAAAAAGAGGACCAACAAGTT

CATGAGGCACCAATCAGACTGAAATGTCAAAATTAAGCATAACTGGCGGAAACCCAGAGG

TCTTAACAGTAGGGTTCGTAGAAGGTCCAAGGGCCAGATCTTGATGCCCAACATTGCTTA

TGGGAGCAACAACAACAACAAAAAAAACATGCTGCCCAGTGGCTTCCAGAAGTTTCTGGT

CCACAGCCTCAAGGAGCTGAAAGTGCTGCTGATGTGCAACAAATCTTACTGTGCTGAGAT

CGCTCACAAAATTTCCTCCAGAACTGCAAAGTCATCATGGAAAGAGTCACCCAGCCGGCC

ATCAGAGTCACCAACCCCAGTACCAGGGTGCACAGCTAAGAAAATGAGTAGAAAGTTCAT

GTCCACGTTTTGTGTGTAAATAAAACCATAAAAACTGCCAAAAAAAATTACATCAATGCC

TCTAAACCCAAAGGACTCTACCCCCACAGGTCCCTGGTTGTTGTGGTGATTTTCATTGTG

TAAAATACTTTCCACATCTTTTGACACCAAGTCTTTCTGCAGCCATGTTTGAAAATTAAC

TTTCAGGCTACAGAGTCTTTCTTATACCAAAGTTGAAGAAAGTTTTAAGAAATATATTTC

TACATCTCCTACATGCAAAACAACAGGAGCAAGTTGAGGAATTCTCAAGAAACTGGTCGA

GAAGAGAGAGCGCTTAGCTATGGAAAAGAGAAAGAAGGAAGGGAGGGCTTCCTGGAGGAG

GTGGCATTTGAACCAGGACTGACATCAGGATGGAAATGTCAGTCAGGGAGTTAAGTAGGG

GGAGCAGCTCCGCCCTCCACGTCCCCAGCTCCTCCCGCCCCTGTTTTTTCTCCCAGTGAC

CCCACGTGAAACGTCTCCGCCTCCTCCAGCCACCAGCAGAAGGGACTGCCTTCCCCTCAG

TGCTCGCCCCTCCCTAGTGATCACTCAGTGCCCCTGAGCTCATTCTTTTCAGTAAATTCT

CTCTCTGCGTGGTGAGAAAACAGGCCTGGAGAGGCTCTGCGACCCGCTTAGGACCACAGA

ACTCGGTACTAGGAAAACTCCTATTTTAAAATCCAGCCCTGGGTGGGAAGATTTGGGAAG

AATCGTTAATATTGAGAGAGAGAGGGAGAAAGAGGATTAGATGAGAGTGGCGCCTCCGCT

CATGTCCGCCCCCTCCCCGCAGAGAATTACCTTTTCCAGGGACGGCAGGAATGCTACGCG

TTTAATGGGACACAGCGCTTCCTGGAGAGATACATCTACAACCGGGAGGAGTTCGTGCGC

TTCGACAGCGACGTGGGGGAGTTCCGGGCGGTGACGGAGCTGGGGCGGCCTGATGAGGAG

TACTGGAACAGCCAGAAGGACATCCTGGAGGAGGAGCGGGCAGTGCCGGACAGGATGTGC

AGACACAACTACGAGCTGGGCGGGCCCATGACCCTGCAGCGCCGAGGTGAGTGAGGGCTT

TGGGCCGGCGGTCCCAGGGCAGCCCCGCGGGCCCGTGCCCAGGGCGCAGGAGCAGCCGGG

TTGGCCTAAGGGACCTTAGTGCCGGGCGGAAAGGGGACTTTGGGTTGGGGATTCATGGGG

GGAGCCCATCTGGAGCTTGTCAGGGGAGCGAGCGCGGGGACCTGGACTGGGCTGAGCATG

GAGTGAGGAGGACGAGAGCAGAGAGACCCCCGGGACTTCATCAGGCCTGGCAGCTGACTG

CATGTGGGGTGAAAAAAGGAAGCCACAGGACAGCGCACAAGGGTATGGTGTGGAGATGGA

GGTGGAGATGGCACAGCAGGCCACACAGAGAAGAAACCTACAGGGAGGTAGCTGGGTTTG

AGGTGCTTGAGGGGCAGATGGGTGGTCTGATGGGCAGGTAGACAGAAGGGTCTGCAGCCG

GGGAGGAGACTGAGATACATGAGACCATCCAGGGAGAGGGGACCCAGGGGGAAGAGCAAA

GGACCGGATCCTGGGAACTGGACAGTTGTGATTTGGCCAAGACAGAAAAGCCTGTGAAAG

AGACCAAAAAAACCCAAGTGCAGTGTGAGGAGAGGCCCGCAGAGAAGAGTCTTGGAAGCT

GAGGGGAGGTGACCTCAGCAGCACAGTGGACAGCGGTGCCAGTGACTTGGGAAGGTCAGA

AAACAGAAGATGGAAAGTGGGTTTGGAAACCAGGGAGACCTGGGGAGAGCAGGTTGGCCG

CAGCGGCAGGAGCTGGAATGGGAGGGGGTGCATGAGGCTGAGTGTGGCGCATCCTCCTCG

GGGCTGAGATGGATTTTACTTGTCTTGGGTTCCCCACGGCTGTCACAGGGCAGTGTCTCA

GTTCATTCGTCTTTTTCCTTCAGGAAGTCTGGGTGTAAAGGGATGGAGAGAGGTGAGGTG

TGTGCAGTAAGAGGATTTCTCAAGGATGGGACAGGAAGGCCTTGGAGCTTTGGCTTCCTC

CTGTGAACTTGTGGGGTGGGGAGCCTGGTGCACCAACCTGAGGGACTTGAGGGAGTAGTA

TCAGGATGTGGGATTGAGCCCTGGACCTTTTTTCTAGAAAGAGGAAAAAAATGAAGGGAG

GAGGAGGAGGAAGCTGGGGAGATCACACCTTTGATTTTCTTGTTCCTGGAAAGTGAAAGG

AAGTTCACCTGCTATGAGTGAGAAGGTGGACACACTGGGTGGGGATGAGGTGAGTGACAT

GAGCTTAGGAAAGTTGCTGAGGTAATTGGTTGAGAGAGGTGTTCAAATAAAAATAACGCA

ATTGGCAAAAACTGTTACTAAGACTTTGTAGAGGCACCAATCAGTGACATGGCAGCATTT

TCTTTCACAGTAATCAACTGCCAGATTGCAGACAGCCCTGATGCCAGCCTAAGGAGTGTG

GGTTTCTCCTCCAGGCCCGCAGGTCCCCAACCTCACTCCTCTGAAGACTCTTCTGGAGAT

CCTCTGTGATGCACAGATCTCCAGACTCAGTGCCCCCAGACTCAGATTCCCTGGGTGGGG

AGGTCTGGGGATCTCTGCTTGTAATCAGCTCCCTAGAGGTTCCCATGTAGCCAGATAAGT

ATTGTCAGAACACTGAAGATTTTTGAAAAATGAAAAAGAGAAGGTTGGAGATGTGTCTTC

AGAAGACTACTAAGGGTGCTGGCTAGAGGAGGGACCAGAGGCAGGGAGATGAGGTAGGAA

ACTGCTATTATTTGTCAGGGAAATTGCAATCAAGGCATGAGTTAGAACAGGGAAAACACA

GAGGCAAGGGAGAGGTGGAAGGGGGAGGAAAGAAGTAGTGACAATTCCAGGGTGGATGTC

CACCCAAATCTAGAAGTAATTGAGCAAATGTTTTCTGGGCATTAGAGAAGGCAACTAGAA

CAAACAGGAATCCTTGCCTTGGTGAAATGTATTTGAACTGGGTCAGAAATGAGGCCATTG

GGTATCAGGCCTTAACTCCAGCGCACCCTGGAGGTCACTGATGTGGCTCCAGGCTGACCT

GCTCCTGTCAAAGAATATTGAGCAAGATGCCTCTCGTGGAATGTTCTGGGACCTTAAAAC

AGATACCCAAGTATTCCCCCTGATTTCATGGTTCCCAGAAGCTCTATGGGGAAGAAATTG

TAGGTAATTCACAACTGAGATTTAGACATAAGTTGAATAGTGTAATGGACATTGAGTTAA

CCGAGGTAATGAAGTAGTGAGACACAGGTGCCCCTGAAATAAACTCACATTGAGGGAAGA

GGCTGACAATGTGGATCAGTCTGAAAACAAGGCAAAAATACAATAGGGAGTAAGGGTTGT

GTGTCAGTTCAAGACTGTACTTTTACCTGGCCCAGCGCCATGTTAGGGTATTTGTGTTCT

CCAGGAAGTAGAAAGGAAAGAACTGAGTGATTAGGGACCTAGAAGACTAATTTGAGACAT

TCCTCTTGATGAGCTGTTCTCTAGGGTAGTCCTCTGAAAGAGCTGTTCTCTAGTGGATCT

CCCTGAATGAACTGTTCTCTAGGAGCACTTGACCCTTTTCTGTGTTTGTTTTTTGTTTTG

TGTTTGTGTTTGTTTTTGAGACAGGTTCTCACTTTGTCTCCCAGGCTGGAGTGCTGTGGC

ACCATCATGGCTCACTGCAGCCTCAACCTCCTGGGCTCAAGTGATCCTCCTGCCTCAGCC

TCCCATGTAGCTAGAACTACAGATACACGTACCACCATGTCTGGCTAATTTATTTTTCTT

TTTAGAGATGGGTTCTCACTATGTTGCCCAGGCCGGTCTCAAAACCCTGGGCTCAAGTGA

TCCTCATGCCTCAACCTCCCAAAGTGCTAAGATTATAGGCATGACCACCATGCCTGGCCT

TTTCTGCTTTCTGAGGAGGAAAAAGGTACTGGTGGCAGAGATCCAAAAGAAAAGTTGCCA

GTGGCAGTGTGGAAATTCACCTGAGAACAACAGGACAAGCTGGGGCACAAATGCAAAGAT

GCAGAGGGAGGCAACACCTGGTCATCTGTGAGACCTTCATGGGACCTGAAGACGCAGCAC

AGAGGAGGAACTTGAAAAAGGACGGGATTTCTACTACTCAAGCATGTAGGAGCTCAGGAT

ATTCTGTAAATATGAAGATTTTGAGTTTTTGTAGGTGAGGTAAAAAAATACATAGGTTTT

TTACAGAATAAGACATGTAAAGCTCTCTTCATTTTCTTTGTATTTTCATGAAGTTATTAG

ATTCACAGGCCACCATAATGCCATTGTCTGTATATCTTAATTTCAAGATATTATTTGAGT

AAATTTTGCTTCCTTTGTATCAAGATAGAACTTTGAAAAGGTAGGTAATTTCACAGTTGA

TCAAATATTCTTTGCCCAAATTACTTTTGGTTAAAATTTCTCCTAAATGTGCTACAGAGT

GCAAACTCTGTCTCCCTGCCATTCCGCTATATACTTACTAACTATTATTTTATTCAAGAT

CATGCATGCTCTACTTGAAGGTCTATTTCTATCTTTTCAATGCTACCCTTACCCACTAGC

CTAATCACATTATTCCTATTTTCAACATCTAGGAATCAATTACATAGTGAACATGCCTAA

GAAATAATAATCTGGGCAGATGCAGTGGCTCAGGCCCGTAATCCCAGCCCTTTGAGAGGC

CGAGCGGGTGGATCACTTGAGGTCAGGCGTTGGTCAAGTGCTCCTAGAGAACCAGGCTGA

CCAACATGGAGAAACCTTGTCTCTACTAATAATACAAAAATTAGCCAGGTGAAGTGGCAG

GCACCTATAATCCCAGCTATTCGGGAGGCTGAGGAAGGAGAATTGGTTGAAGCCCAGAGG

TGGAGGTTGCAGTGAGCCAATATTGCGCCACTGCATTCCAGACTTGGCAACAGAGTGACA

CTCCATCTCAACAAAAAGAAAGAATGAAAGAAAGAAAGAGCGAGATTATGTCTCAAAAAA

AAGGAAGGAAGGAAGGAAGGAAGGAAGGAAGGAAGGAAGGAAGGAAGGAAAGAAGGACAA

TCTCAAATTCTATTTCATTATTTTTCTTCCACGCTCCTAGTCCAGCCTAGGGTGAATGTT

TCCCCCTCCAAGAAGGGGCCCTTGCAGCACCACAACCTGCTTGTCTGCCACGTGACGGAT

TTCTACCCAGGCAGCATTCAAGTCCGATGGTTCCTGAATGGACAGGAGGAAACAGCTGGG

GTCGTGTCCACCAACCTGATCCGTAATGGAGACTGGACCTTCCAGATCCTGGTGATGCTG

GAAATGACCCCCCAGCAGGGAGATGTCTACACCTGCCAAGTGGAGCACACCAGCCTGGAT

AGTCCTGTCACCGTGGAGTGGAGTGAGTCTCTGATGACCCTCTAGACCCCACCTCTGAAG

AGCAGGGGACTCTCTGGCTCTGGGGTCCACTCATCTTATCTTCTGCATCTATACCCTGGG

GCCATGTCCAAACCCCATCTTTCTTCTATACCAGCTCCTGAGCATAGTTTGAAGCCAGGG

AAATGGAGACTTCCTGACCTTGGCTTAGGGGTTCCTGAAGATTCATAGTTCTCCCCCTTG

TCAGAGAATCTAGGGACACTGACTGGTCTCGAAACCCTCACACTTAGGAACTGACCTCAC

ACATAGGAACAGTTCTCTTCCTTCAGCATTTTAGCCTCTTCTCAGGCATTTTGAGAGGCA

ACTTCCAGAATCAGCATTTGCCACCTTGTTGAGGTCACACCCCTGTTCCAGATATGAGGG

TGGCTCTTTCTGAATTTCCTCTTAGCAAGCTTTTTCCGCTGCACTGTCCTCATCCCGATA

TGCTGCATCAGGCTCCAGAATCTCAGACAGGACATGAGTAGGGATGCAGCTGGTGGAGGT

GACACTAAACCTGGGTCTGTCCTTCCCAGAGGCACAGTCTGATTCTGCCCGGAGTAAGAC

ATTGACGGGAGCTGGGGGCTTCGTGCTGGGGCTCATCATCTGTGGAGTGGGCATCTTCAT

GCACAGGAGGAGCAAGAAAGGTGAGAAAGCCTGCAGGGTGAGCGGGACTTACCTTCCCCT

GGCATATTCACACTTATTCCACGATGAGGGGTTTGACAGAAAAGAAATGTCAGAAAGCTC

TAGAGGCCACTGATATCAGATAATCGGGGAACAAACATGACCTATAGCGAGAGAGGGATC

CCAGGCTGGGATCTTAATGCAGCCAGATGCATGAGGTCCCAAGTACTCAGGCTCCTGCGG

AGCGTCCATTGAGTGATGGGCAATGGAATTTGGTGGGATGGAAATGTTTCTCTAATTATC

TGAGGTGGTTTCAATGGCTGATTATATAACCTTTCGTCTTTCATTTCAGTTCAACGAGGA

TCTGCATAAACAGGTAATATTCCTGCTTTGATTTCCTTGTGGGGTGGGTTGCAGGAGGAT

ATGAGTCCTTTCTGTGCATTGTAACACTGAGGCTCCTCCAGGAAGGGAATCTCAGGCATG

AACCCCTCTTTCAATGTCAGCCTTCAGGCAAGTGGGGAAAGAGCATTGCTTGGCTCCATT

GCTGAAGGAAGCAGAGATCAACTCTGTTATTTATCAGCCTGAGACGCATCCTCTCACCAT

AATTTTTCTCTCCTGGACTTACAGGAAGGAGGCTGGCAACCTGGGATAACTTGTCTTTTA

CCCCCACAGGGTTCCTGAGCTCACTGAAAAGACTATTGTGCCTTAGGAAAAGCATTTGCT

GTGTTTCGTTAGCATCTGGCTCCAGGACAGACCTTCAACTTCCAAATTGGATACTGCTGC

CAAGAAGTTGCTCTGAAGTCAGTTTCTATCATTCTGCTCTTTGATTCAAAGCACTGTTTC

TCTCACTGGGCCTCCAACCATGTTCCCTTCTTCTTAGCACCACAAATAATCAAAACCCAA

CATGACTGTTTGTTTTCCTTTAAAAATATGCACCAAATCATCTCTCATCACTTTTCTCTG

AGGGTTTTAGTAGACAGTAGGAGTTAATAAAGAAGTTCATTTTGGTTTAAACATAGGAAA

GAAGAGAACCATGAAAATGGGGATATGTTAACTATTGTATAATGGGGCCTGTTACACATG

ACACTCTTCTGAATTGACTGTATTTCAGTGAGCTGCCCCCAAATCAAGTTTAGTGCCCTC

ATCCATTTATGTCTCAGACCACTATTCTTAACTATTCAATGGTGAGCAGACTGCAAATCT

GCCTGATAGGACCCATATTCCCACAGCACTAATTCAACATATACCTTACTGAGAGCATGT

TTTATCATTACCATTAAGAAGTTAAATGAACATCAGAATTTAAAATCATAAATATAATCT

AATACACTTTAA

>HLA:HLA17114 DPB1*02:02:01:04

TAATCCCTGTAGATGGGCCAGCAGAATATTTGAGATCACCTTCAGAGCAAAGAAAACGCA

TAATCTCGCCAAACATCATGACTTATCTGACTGGTTAAAATGAGTATCACTGTCTTTCCT

CCGTCATCTTAAGTGCATCACAGGCTTTATATTTTCAGACCTTTCATACTAACTTTCTGC

CTAGTGAGCAATGACTCATACAAAGCTCAGTGTCCATTGGTTCTTTTCTCAGACTCTGTC

CAATCCCAGGGTCACAGAAGACTACTTGGGTTCATGGTCTCTAATATTTCAAACAGGAGC

TCCCTTTAGCGAGTCCTTCTTTTCCTGACTGCAGCTCTTTTCATTTTGCCATCCTTTTCC

AGCTCCATGATGGTTCTGCAGGTTTCTGCGGCCCCCCGGACAGTGGCTCTGACGGCGTTA

CTGATGGTGCTGCTCACATCTGTGGTCCAGGGCAGGGCCACTCCAGGTAAGAGCCGAACT

GCCATTCTTGGAGGGTCTGGCTCAGGGAACAATTCCTAGGGGACGTTATCTTTAAGGGAT

CAAATTCTGAGACAGGCTGCGGGGGCTCCTGCCCTAAGGCAGTGTCCTCTCTTCCCAGCT

AGAGAAAGAGGTTCATCCCCTATAGGATAGCTTGCTACCCTACTGGCCTATTCTCTCTCC

AAGGACATGGGTACAGTAAACAGAGAGAGGTGCCCAGTGGTCAGTATGCTTGTCTTTGGG

GAAAATGGGACCAAGAGGTCCTGGATAACCTTGGACAGACAAGGTTTGCAGAGAGAGAAG

TTGGCAAGTGCAGGCTCCTGGGCGTGTTCATGTCTGCATCCAGCCTGGAGGGGACTCAGG

CAGAGAGCCCTAAGCTGGAGTGTCCAGGCTCTGAGGATCACTGAGGATTCAGTGCTCACG

AAGAATGCCTCTTATTCCCCAGGGTGGAGCAGGAGCCCACATCCCTTGGACAATTAAGGA

GAGAAGGGAGGGAGGGGGATAGGTTTTAGCCCCTGAAGGCATTCTCATTAAAGGTACTTC

TCCCAGCCTCCCCAGAACTTGGTTAGGGTACTAGAGTGGGTTGCGACTTGTAGGAAGAAT

GAGATGAGGTTGTGTGGGTGCATGACAGGGATTGAGTGTAGGTTATCAGACAGCCAAGGA

AGCAGTAACCAAGTGAAAAATCTCTTCTTCCTGCTGCCTCCCTGTGGCTGGTGTAATATT

ATGGCATCTATGATCCATTGTTTTTCTCTCAGGATACTCTCAGGATATTTCTTTTTATAT

ATATATATACTTTAAGTTCTAGGGTACATGTGCACAACGTGCAGGTTTGTTACATATGTA

TACATGTGCCATGTTGGTGTGCTGCACCCATTAACTCGTCATTTACATTAGGTATATTTC

CTAATGCTATCCCTCCCCCCTCCCCCCACCCCACAACAGGCCCCGGTGTATGATGTTCCC

CTTCCTGTGTCCATGTGTTCTCATTGTTCAGTTCCCACCTATGAGTGAGAACATGTGGTC

TTTGGTTTTTTGTCCTTGCAATAGTTTGCTGTGAATGATGGTTTCCAGCTTCCTCCATGT

CCCTACAAAGGACATGAACTCATCCTTTTTTATGGCTGCACAGTATTCCATGGTGTATAT

GTGTGCATTTTCTTAATCCAGTCTATCACTGATGGACAGTTGGGTTGGTTCCAAGTCTTT

GCTATTGTGAATAGTGCCGCTATAAACATATGTGTGCATGTGTCTTTATAGCAGCATGAT

TTATAATCCTTTGGGTATATACCCAGTAATGGGATGGCTGGGTCAAATGGTATTTCTAGT

TCTAGATCCTTGAGGAATTGCCACACTGTCTTGAGATACCATCTCACACCAGTTAAAATG

GCGATCATTAAAAAGTCAGGAAACAACAGGTGCTGGAGAGGATGTGGAGAAATAGGAACA

CTTTTACTCTGTTGGTGGGACTGTAAACTAGTTCAACCATTGTACTCTCAGGACATTTCT

AGTCCAAATTTACACCAACACTCTGAGAGGAAGGACTGCAAAGTAGGTACCTTAGTTTTC

CACTGACTTCCACTTTTCCTGCTTACACCCTTCCTCCTAGACCTCTCCACACCCCTCCTA

GGACACACCTAAAAGGTACTGACATCATGTCACCTCCTCATCTTTCAGGGTAGCAAGGTT

GGAATCTCCTGAATACAGCCCCTCAAGCCCTAAAACCTCTTATCTATTACCTTGGGTTCA

TTGTCCAGGAAGGGGAGGAGAACTTGAACTTGTAGTCACAGAAGGGTGCTGAGAACTAAC

CAGCAGGACGGCTCAGCCCTGGGAACTGCAGAGGGGTGAGGCTGGGGAGAGAGGAGGCTG

GAGCAGCACTGGTGACACTGAACAGTGTCAGGAGGAAGTGACGGATGCAGCGCCCCCATC

CCATAGGCAGAGCTGTCATGTGGGATGAGGGACAGTGTTGGGAGCCACCAAGGAAACCCA

GAGGTGGGGGAGCAGAGAGCAGAAGGGAGCATGTGATGCTGGACAGTGAAAGGGAGGACA

GGCAAAGGCTGGGTTGAGGTTTGTAGGGGGAATGAGATGAGGCAGTGGAGCCATGTGACA

GGGACTGAGGGTAGATTACTGGAGCTCCCTGCGTAGAATGAATGTTCAATCAAAATTTGC

TGGAGGGAGAGCTGGAGCCATAGGGGAGTGGGTAAAGTGGGCAGGGCTGATTCCACAATT

CCCTGCATGCTCCCCCAACTCCACACACATCCCCAACCTCAAACAGGGCACAAGACCAAA

GGGCTGAGGAGCCAGGCTATAGCTTAAAGAGGCTGGGGGAGAAAAGCTTGGCTGAGACAA

CCCATAGGGAGCTAGAGGTTTTTAATATATCCTATTCTGAATAAGAGACGAATTCATTCA

GATCAGTGGTTTCAAACCGTGCTCTGGGCAACTCAATTGCTAAGGGTTCCACAAACAGGA

TAAAGTTTCTTATATACAAAAAAAAATGAAGGTTTCAAATTACACCATAAAACCCCTCAT

TGCTTATGTCTACTTGGCAGGTAAAATTCCATTTCAAAAGTTAAATGTACTTAAAAAATT

ACCTAAGACTGGGTAAATTAAAAAAATTAAATGTTGCAAAGAAAAAATTCAAAATTCTTA

TTCTTGAATGAAAAACGTTCTCTTACTGGTGATTGAGGAGGAGAAACAAAGACTAACAAA

TGAAAATGGGAGAATCCACACTCAGAGTGGGGCAACTGAACAGGCAGGGGCGGATGGATG

GCAGAGGAGGAGGAATCTGGACTCAAGGAGCTGGGGGGCTCTGGGCCTGGAATTTTAGGG

TCTGGGGCCCAAGGCACCAGGAGAAGAGGCAGGTCAGGATATCTGAGTCAAGACCTGGGA

TCTTGCCTTAGCAATGACACTGGAGACTAAAGGTGGACTCCATGGTGCCCTTGAGCCCAG

CCCTACCCCATCTCCACTATCCTCTGCCACCAGCTGTGCAACTTCTGCTAGGGGTGAGGT

TAATAAACTGGAGAAGTTAATTTGTGGAGCATGAAACAGATGAGCAGAACAATCACAGCA

CCTTAATTTCCCCAGTGTGCCCAAGAACAGAGCAGGCCTGAAGATACTCAAACAGAAACA

AACATGTGCCGTGTCACTGATAATTCTGTGTAGACACACACCTGCCAGACACTGCTCATG

GCACTCCCTAGGAAGAACAGCATGTGGGAAAGGCTGCCAAAATTGTTCATGTAAAAATTA

CATCAATGCTGTCTTCCTCGGTGCTGCCTATGCAGCTGGCAGCCATCTCTTCCTCCACAT

CATGGCCTCCCTCAGACTCCTCATGAAGGATAAGATCCTCAAAAAGAGGACCAACAAGTT

CATGAGGCACCAATCAGACTGAAATGTCAAAATTAAGCATAACTGGCGGAAACCCAGAGG

TCTTAACAGTAGGGTTCGTAGAAGGTCCAAGGGCCAGATCTTGATGCCCAACATTGCTTA

TGGGAGCAACAACAACAACAAAAAAAACATGCTGCCCAGTGGCTTCCAGAAGTTTCTGGT

CCACAGCCTCAAGGAGCTGAAAGTGCTGCTGATGTGCAACAAATCTTACTGTGCTGAGAT

CGCTCACAAAATTTCCTCCAGAACTGCAAAGTCATCATGGAAAGAGTCACCCAGCCGGCC

ATCAGAGTCACCAACCCCAGTACCAGGGTGCACAGCTAAGAAAATGAGTAGAAAGTTCAT

GTCCACGTTTTGTGTGTAAATAAAACCATAAAAACTGCCAAAAAAAATTACATCAATGCC

TCTAAACCCAAAGGACTCTACCCCCACAGGTCCCTGGTTGTTGTGGTGATTTTCATTGTG

TAAAATACTTTCCACATCTTTTGACACCAAGTCTTTCTGCAGCCATGTTTGAAAATTAAC

TTTCAGGCTACAGAGTCTTTCTTATACCAAAGTTGAAGAAAGTTTTAAGAAATATATTTC

TACATCTCCTACATGCAAAACAACAGGAGCAAGTTGAGGAATTCTCAAGAAACTGGTCGA

GAAGAGAGAGCGCTTAGCTATGGAAAAGAGAAAGAAGGAAGGGAGGGCTTCCTGGAGGAG

GTGGCATTTGAACCAGGACTGACATCAGGATGGAAATGTCAGTCAGGGAGTTAAGTAGGG

GGAGCAGCTCCGCCCTCCACGTCCCCAGCTCCTCCCGCCCCTGTTTTTTCTCCCAGTGAC

CCCACGTGAAACGTCTCCGCCTCCTCCAGCCACCAGCAGAAGGGACTGCCTTCCCCTCAG

TGCTCGCCCCTCCCTAGTGATCACTCAGTGCCCCTGAGCTCATTCTTTTCAGTAAATTCT

CTCTCTGCGTGGTGAGAAAACAGGCCTGGAGAGGCTCTGCGACCCGCTTAGGACCACAGA

ACTCGGTACTAGGAAAACTCCTATTTTAAAATCCAGCCCTGGGTGGGAAGATTTGGGAAG

AATCGTTAATATTGAGAGAGAGAGGGAGAAAGAGGATTAGATGAGAGTGGCGCCTCCGCT

CATGTCCGCCCCCTCCCCGCAGAGAATTACCTTTTCCAGGGACGGCAGGAATGCTACGCG

TTTAATGGGACACAGCGCTTCCTGGAGAGATACATCTACAACCGGGAGGAGCTCGTGCGC

TTCGACAGCGACGTGGGGGAGTTCCGGGCGGTGACGGAGCTGGGGCGGCCTGAGGCGGAG

TACTGGAACAGCCAGAAGGACATCCTGGAGGAGGAGCGGGCAGTGCCGGACAGGATGTGC

AGACACAACTACGAGCTGGGCGGGCCCATGACCCTGCAGCGCCGAGGTGAGTGAGGGCTT

TGGGCCGGCGGTCCCAGGGCAGCCCCGCGGGCCCGTGCCCAGGGCGCAGGAGCAGCCGGG

TTGGCCTAAGGGACCTTAGTGCCGGGCGGAAAGGGGACTTTGGGTTGGGGATTCATGGGG

GGAGCCCATCTGGAGCTTGTCAGGGGAGCGAGCGCGGGGACCTGGACTGGGCTGAGCATG

GAGTGAGGAGGACGAGAGCAGAGAGACCCCCGGGACTTCATCAGGCCTGGCAGCTGACTG

CATGTGGGGTGAAAAAAGGAAGCCACAGGACAGCGCACAAGGGTATGGTGTGGAGATGGA

GGTGGAGATGGCACAGCAGGCCACACAGAGAAGAAACCTACAGGGAGGTAGCTGGGTTTG

AGGTGCTTGAGGGGCAGATGGGTGGTCTGATGGGCAGGTAGACAGAAGGGTCTGCAGCCG

GGGAGGAGACTGAGATACATGAGACCATCCAGGGAGAGGGGACCCAGGGGGAAGAGCAAA

GGACCGGATCCTGGGAACTGGACAGTTGTGATTTGGCCAAGACAGAAAAGCCTGTGAAAG

AGACCAAAAAAACCCAAGTGCAGTGTGAGGAGAGGCCCGCAGAGAAGAGTCTTGGAAGCT

GAGGGGAGGTGACCTCAGCAGCACAGTGGACAGCGGTGCCAGTGACTTGGGAAGGTCAGA

AAACAGAAGATGGAAAGTGGGTTTGGAAACCAGGGAGACCTGGGGAGAGCAGGTTGGCCG

CAGCGGCAGGAGCTGGAATGGGAGGGGGTGCATGAGGCTGAGTGTGGCGCATCCTCCTCG

GGGCTGAGATGGATTTTACTTGTCTTGGGTTCCCCACGGCTGTCACAGGGCAGTGTCTCA

GTTCATTCGTCTTTTTCCTTCAGGAAGTCTGGGTGTAAAGGGATGGAGAGAGGTGAGGTG

TGTGCAGTAAGAGGATTTCTCAAGGATGGGACAGGAAGGCCTTGGAGCTTTGGCTTCCTC

CTGTGAACTTGTGGGGTGGGGAGCCTGGTGCACCAACCTGAGGGACTTGAGGGAGTAGTA

TCAGGATGTGGGATTGAGCCCTGGACCTTTTTTCTAGAAAGAGGAAAAAAATGAAGGGAG

GAGGAGGAGGAAGCTGGGGAGATCACACCTTTGATTTTCTTGTTCCTGGAAAGTGAAAGG

AAGTTCACCTGCTATGAGTGAGAAGGTGGACACACTGGGTGGGGATGAGGTGAGTGACAT

GAGCTTAGGAAAGTTGCTGAGGTAATTGGTTGAGAGAGGTGTTCAAATAAAAATAACGCA

ATTGGCAAAAACTGTTACTAAGACTTTGTAGAGGCACCAATCAGTGACATGGCAGCATTT

TCTTTCACAGTAATCAACTGCCAGATTGCAGACAGCCCTGATGCCAGCCTAAGGAGTGTG

GGTTTCTCCTCCAGGCCCGCAGGTCCCCAACCTCACTCCTCTGAAGACTCTTCTGGAGAT

CCTCTGTGATGCACAGATCTCCAGACTCAGTGCCCCCAGACTCAGATTCCCTGGGTGGGG

AGGTCTGGGGATCTCTGCTTGTAATCAGCTCCCTAGAGGTTCCCATGTAGCCAGATAAGT

ATTGTCAGAACACTGAAGATTTTTGAAAAATGAAAAAGAGAAGGTTGGAGATGTGTCTTC

AGAAGACTACTAAGGGTGCTGGCTAGAGGAGGGACCAGAGGCAGGGAGATGAGGTAGGAA

ACTGCTATTATTTGTCAGGGAAATTGCAATCAAGGCATGAGTTAGAACAGGGAAAACACA

GAGGCAAGGGAGAGGTGGAAGGGGGAGGAAAGAAGTAGTGACAATTCCAGGGTGGATGTC

CACCCAAATCTAGAAGTAATTGAGCAAATGTTTTCTGGGCATTAGAGAAGGCAACTAGAA

CAAACAGGAATCCTTGCCTTGGTGAAATGTATTTGAACTGGGTCAGAAATGAGGCCATTG

GGTATCAGGCCTTAACTCCAGCGCACCCTGGAGGTCACTGATGTGGCTCCAGGCTGACCT

GCTCCTGTCAAAGAATATTGAGCAAGATGCCTCTCGTGGAATGTTCTGGGACCTTAAAAC

AGATACCCAAGTATTCCCCCTGATTTCATGGTTCCCAGAAGCTCTATGGGGAAGAAATTG

TAGGTAATTCACAACTGAGATTTAGACATAAGTTGAATAGTGTAATGGACATTGAGTTAA

CCGAGGTAATGAAGTAGTGAGACACAGGTGCCCCTGAAATAAACTCACATTGAGGGAAGA

GGCTGACAATGTGGATCAGTCTGAAAACAAGGCAAAAATACAATAGGGAGTAAGGGTTGT

GTGTCAGTTCAAGACTGTACTTTTACCTGGCCCAGCGCCATGTTAGGGTATTTGTGTTCT

CCAGGAAGTAGAAAGGAAAGAACTGAGTGATTAGGGACCTAGAAGACTAATTTGAGACAT

TCCTCTTGATGAGCTGTTCTCTAGGGTAGTCCTCTGAAAGAGCTGTTCTCTAGTGGATCT

CCCTGAATGAACTGTTCTCTAGGAGCACTTGACCCTTTTCTGTGTTTGTTTTTTGTTTTG

TGTTTGTGTTTGTTTTTGAGACAGGTTCTCACTTTGTCTCCCAGGCTGGAGTGCTGTGGC

ACCATCATGGCTCACTGCAGCCTCAACCTCCTGGGCTCAAGTGATCCTCCTGCCTCAGCC

TCCCATGTAGCTAGAACTACAGATACACGTACCACCATGTCTGGCTAATTTATTTTTCTT

TTTAGAGATGGGTTCTCACTATGTTGCCCAGGCCGGTCTCAAAACCCTGGGCTCAAGTGA

TCCTCATGCCTCAACCTCCCAAAGTGCTAAGATTATAGGCATGACCACCATGCCTGGCCT

TTTCTGCTTTCTGAGGAGGAAAAAGGTACTGGTGGCAGAGATCCAAAAGAAAAGTTGCCA

GTGGCAGTGTGGAAATTCACCTGAGAACAACAGGACAAGCTGGGGCACAAATGCAAAGAT

GCAGAGGGAGGCAACACCTGGTCATCTGTGAGACCTTCATGGGACCTGAAGACGCAGCAC

AGAGGAGGAACTTGAAAAAGGACGGGATTTCTACTACTCAAGCATGTAGGAGCTCAGGAT

ATTCTGTAAATATGAAGATTTTGAGTTTTTGTAGGTGAGGTAAAAAAATACATAGGTTTT

TTACAGAATAAGACATGTAAAGCTCTCTTCATTTTCTTTGTATTTTCATGAAGTTATTAG

ATTCACAGGCCACCATAATGCCATTGTCTGTATATCTTAATTTCAAGATATTATTTGAGT

AAATTTTGCTTCCTTTGTATCAAGATAGAACTTTGAAAAGGTAGGTAATTTCACAGTTGA

TCAAATATTCTTTGCCCAAATTACTTTTGGTTAAAATTTCTCCTAAATGTGCTACAGAGT

GCAAACTCTGTCTCCCTGCCATTCCGCTATATACTTACTAACTATTATTTTATTCAAGAT

CATGCATGCTCTACTTGAAGGTCTATTTCTATCTTTTCAATGCTACCCTTACCCACTAGC

CTAATCACATTATTCCTATTTTCAACATCTAGGAATCAATTACATAGTGAACATGCCTAA

GAAATAATAATCTGGGCAGATGCAGTGGCTCAGGCCCGTAATCCCAGCCCTTTGAGAGGC

CGAGCGGGTGGATCACTTGAGGTCAGGCGTTGGTCAAGTGCTCCTAGAGAACCAGGCTGA

CCAACATGGAGAAACCTTGTCTCTACTAATAATACAAAAATTAGCCAGGTGAAGTGGCAG

GCACCTATAATCCCAGCTATTCGGGAGGCTGAGGAAGGAGAATTGGTTGAAGCCCAGAGG

TGGAGGTTGCAGTGAGCCAATATTGCGCCACTGCATTCCAGACTTGGCAACAGAGTGACA

CTCCATCTCAACAAAAAGAAAGAATGAAAGAAAGAAAGAGCGAGATTATGTCTCAAAAAA

AAGGAAGGAAGGAAGGAAGGAAGGAAGGAAGGAAGGAAGGAAAGAAGGACAATCTCAAAT

TCTATTTCATTATTTTTCTTCCACGCTCCTAGTCCAGCCTAGGGTGAATGTTTCCCCCTC

CAAGAAGGGGCCCTTGCAGCACCACAACCTGCTTGTCTGCCACGTGACGGATTTCTACCC

AGGCAGCATTCAAGTCCGATGGTTCCTGAATGGACAGGAGGAAACAGCTGGGGTCGTGTC

CACCAACCTGATCCGTAATGGAGACTGGACCTTCCAGATCCTGGTGATGCTGGAAATGAC

CCCCCAGCAGGGAGATGTCTACACCTGCCAAGTGGAGCACACCAGCCTGGATAGTCCTGT

CACCGTGGAGTGGAGTGAGTCTCTGATGACCCTCTAGACCCCACCTCTGAAGAGCAGGGG

ACTCTCTGGCTCTGGGGTCCACTCATCTTATCTTCTGCATCTATACCCTGGGGCCATGTC

CAAACCCCATCTTTCTTCTATACCAGCTCCTGAGCATAGTTTGAAGCCAGGGAAATGGAG

ACTTCCTGACCTTGGCTTAGGGGTTCCTGAAGATTCATAGTTCTCCCCCTTGTCAGAGAA

TCTAGGGACACTGACTGGTCTCGAAACCCTCACACTTAGGAACTGACCTCACACATAGGA

ACAGTTCTCTTCCTTCAGCATTTTAGCCTCTTCTCAGGCATTTTGAGAGGCAACTTCCAG

AATCAGCATTTGCCACCTTGTTGAGGTCACACCCCTGTTCCAGATATGAGGGTGGCTCTT

TCTGAATTTCCTCTTAGCAAGCTTTTTCCGCTGCACTGTCCTCATCCCGATATGCTGCAT

CAGGCTCCAGAATCTCAGACAGGACATGAGTAGGGATGCAGCTGGTGGAGGTGACACTAA

ACCTGGGTCTGTCCTTCCCAGAGGCACAGTCTGATTCTGCCCGGAGTAAGACATTGACGG

GAGCTGGGGGCTTCGTGCTGGGGCTCATCATCTGTGGAGTGGGCATCTTCATGCACAGGA

GGAGCAAGAAAGGTGAGAAAGCCTGCAGGGTGAGCGGGACTTACCTTCCCCTGGCATATT

CACACTTATTCCACGATGAGGGGTTTGACAGAAAAGAAATGTCAGAAAGCTCTAGAGGCC

ACTGATATCAGATAATCGGGGAACAAACATGACCTATAGCGAGAGAGGGATCCCAGGCTG

GGATCTTAATGCAGCCAGATGCATGAGGTCCCAAGTACTCAGGCTCCTGCGGAGCGTCCA

TTGAGTGATGGGCAATGGAATTTGGTGGGATGGAAATGTTTCTCTAATTATCTGAGGTGG

TTTCAATGGCTGATTATATAACCTTTCGTCTTTCATTTCAGTTCAACGAGGATCTGCATA

AACAGGTAATATTCCTGCTTTGATTTCCTTGTGGGGTGGGTTGCAGGAGGATATGAGTCC

TTTCTGTGCATTGTAACACTGAGGCTCCTCCAGGAAGGGAATCTCAGGCATGAACCCCTC

TTTCAATGTCAGCCTTCAGGCAAGTGGGGAAAGAGCATTGCTTGGCTCCATTGCTGAAGG

AAGCAGAGATCAACTCTGTTATTTATCAGCCTGAGACGCATCCTCTCACCATAATTTTTC

TCTCCTGGACTTACAGGAAGGAGGCTGGCAACCTGGGATAACTTGTCTTTTACCCCCACA

GGGTTCCTGAGCTCACTGAAAAGACTATTGTGCCTTAGGAAAAGCATTTGCTGTGTTTCG

TTAGCATCTGGCTCCAGGACAGACCTTCAACTTCCAAATTGGATACTGCTGCCAAGAAGT

TGCTCTGAAGTCAGTTTCTATCATTCTGCTCTTTGATTCAAAGCACTGTTTCTCTCACTG

GGCCTCCAACCATGTTCCCTTCTTCTTAGCACCACAAATAATCAAAACCCAACATGACTG

TTTGTTTTCCTTTAAAAATATGCACCAAATCATCTCTCATCACTTTTCTCTGAGGGTTTT

AGTAGACAGTAGGAGTTAATAAAGAAGTTCATTTTGGTTTAAACATAGGAAAGAAGAGAA

CCATGAAAATGGGGATATGTTAACTATTGTATAATGGGGCCTGTTACACATGACACTCTT

CTGAATTGACTGTATTTCAGTGAGCTGCCCCCAAATCAAGTTTAGTGCCCTCATCCATTT

ATGTCTCAGACCACTATTCTTAACTATTCAATGGTGAGCAGACTGCAAATCTGCCTGATA

GGACCCATATTCCCACAGCACTAATTCAACATATACCTTACTGAGAGCATGTTTTATCAT

TACCATTAAGAAGTTAAATGAACATCAGAATTTAAAATCATAAATATAATCTAATACACT

TTAA

>HLA:HLA00521 DPB1*04:01:01:01

TAATCCCTGTAGATGGGCCAGCAGAATATTTGAGATCACCTTCAGAGCAAAGAAAACGCA

TAATCTCCCCAAACATCATGACTTATCTGACTGGTTAAAATGAGTATCACTGTCTTTCCT

CCGTCATCTTAAGTGCATCACAGGCTTTATATTTTCAGACCTTTCATACTAACTTTCTGC

CTAGTGAGCAATGACTCATACAAAGCTCAGTGTCCATTGGTTCTTTTCTCAGACTCTGTC

CAATCCCAGGGTCACAGAAGACTACTTGGGTTCATGGTCTCTAATATTTCAAACAGGAGC

TCCCTTTAGCGAGTCCTTCTTTTCCTGACTGCAGCTCTTTTCATTTTGCCATCCTTTTCC

AGCTCCATGATGGTTCTGCAGGTTTCTGCGGCCCCCCGGACAGTGGCTCTGACGGCGTTA

CTGATGGTGCTGCTCACATCTGTGGTCCAGGGCAGGGCCACTCCAGGTAAGAGCCGAACT

GCCATTCTTGGAGGGTCTGGCTCAGGGAACAATTCCTAGGGGACGTTATCTTTAAGGGAT

CAAATTCTGAGACAGGCTGCGGGGGCTCCTGCCCTAAGGCAGTGTCCTCTCTTCCCAGCT

AGAGAAAGAGGTTCATCCCCTATAGGATAGCTTGCTACCCTACTGGCCTATTCTCTCTCC

AAGGACATGGGTACAGTAAACAGAGAGAGGTGCCCAGTGGTCAGTGTCTTTGGGGAAAAT

GGGACCAAGAGGTCCTGGATAACCTTGGACAGACAAGGTTTGCAGAGAGAGAAGTTGGCA

AGTGCAGGCTCCTGGGCGTGTTCATGTCTGCATCCAGCCTGGAGGGGACTCAGGCAGAGA

GCCCTAAGCTGGAGTGTCCAGGCTCTGAGGATCACTGAGGATTCAGTGCTCACGAAGAAT

GCCTCTTATTCCCCAGGGTGGAGCAGGAGCCCACATCCCTTGGACAATTAAGGAGAGAAG

GGAGGGAGGGGGATAGGTTTTAGCCCCTGAAGGCATTCTCATTAAAGGTACTTCTCCCAG

CCTCCCCAGAACTTGGTTAGGGTACTAGAGTGGGTTGCGACTTGTAGGAAGAATGAGATG

AGGTTGTGTGGGTGCATGACAGGGATTGAGTGTAGGTTATCAGACAGCCAAGGAAGCAGT

AACCAAGTGAAAAATCTCTTCTTCCTGCTGCCTCCCTGTGGCTGGTGTAATATTATGGCA

TCTATGATCCATTGTTTTTCTCTCAGGATACTCTCAGGATATTTCTTTTTATATATATAT

ATACTTTAAGTTCTAGGGTACATGTGCACAACGTGCAGGTTTGTTACATATGTATACATG

TGCCATGTTGGTGTGCTGCACCCATTAACTCGTCATTTACATTAGGTATATTTCCTAATG

CTATCCCTCCCCCCTCCCCCCACCCCACAACAGGCCCCGGTGTATGATGTTCCCCTTCCT

GTGTCCATGTGTTCTCATTGTTCAGTTCCCACCTATGAGTGAGAACATGTGGTCTTTGGT

TTTTTGTCCTTGCAATAGTTTGCTGTGAATGATGATTTCCAGCTTCCTCCATGTCCCTAC

AAAGGACATGAACTCATCCTTTTTTATGGCTGCACAGTATTCCATGGTGTATATGTGTGC

ATTTTCTTAATCCAGTCTATCACTGATGGACAGTTGGGTTGGTTCCAAGTCTTTGCTATT

GTGAATAGTGCCGCAATAAACATATGTGTGCATGTGTCTTTATAGCAGCATGATTTATAA

TCCTTTGGGTATATATCCAGTAATGGGATGGCTGGGTCAAATGGTATTTCTAGTTCTAGA

TCTTTGAGGAATTGCCACACTGTCTTGAGATACCATCTCACACCAGTTAAAATGGCGATC

ATTAAAAAGTCAGGAAACAACAGGTGCTGGAGAGGATGTGGAGAAATAGGAACACTTTTA

CTCTGTTGGTGGGACTGTAAACTAGTTCAACCATTGTACTCTCAGGACATTTCTAGTCCA

AATTTACACCAACACTCTGAGAGGAAGGACTGCAAAGTAGGTACCTTAGTTTTCCACTGA

CTTCCACTTTTCCTGCTTACACCCTTCCTCCTAGACCTCTCCACACCCCTCCTAGGACAC

ACCTAGAAGGTACTGACATCATGTCACCTCCTCATCTTTCAGGGTAGCAAGGTTGGAATC

TCCTGAATACAGCCCCTCAAGCCCTAAAACCTCTTATCTATTACCTTGGGTTCATTGTCC

AGGAAGGGGAGGAGAACTTGAACTTGTAGTCACAGAAGGGTGCTGAGAACTAACCAGCAG

GACGGCTCAGCCCTGGGAACTGCAGAGGGGTGAGGCTGGGGAGAGAGGAGGCTGGAGCAG

CACTGGTGACACTGAACAGTGTCAGGAGGAAGTGACGGATGCAGCGCCCCCATCCCATAG

GCAGAGCTGTCATGTGGGATGAGGGACAGTGTTGGGAGCCACCAAGGAAACCCAGAGGTG

GGGGAGCAGAGAGCAGAAGGGGGCATGTGATGCTGGGCAGTGAAAGGGAGGACGGGCAAA

GGCTGGGTTGAGGTTTGTAGGGGGAATGAGATGAGGCAGTGGAGCCATGTGACAGGGACT

GAGGGTAGATTACTGGAGCTCCCTGCGTAGAATGAATGTTCAATCAAAACCTGCTGGAGG

GAGAGCTGGAGCCATAGGGGAGTGGGTAAAGTGGGCAGGGCTGATTCCACAATTCCCTGC

ATGCTCCCCCAACTCCACACACATCCCCAACCTCAAACAGGGCACAAGACCAAAGGGCTG

AGGAGCCAGGCTATAGCTTAAAGAGGCTGGGGGAGAAAAGCTTGGCTGAGACAACCCATA

GGGAGCTAGAGGTTTTTAATATATCCTATTCTGAATAAGAGACGAATTCATTCAGATCAG

TGGTTTCAAACCGTGCTCTGGGCAACTCAATTGCTAAGGGTTCCACAAACAGGATAAAGT

TTCTTATATACAAAAAAAAATGAAGGTTTCAAATTACACCATAAAACCCCTCATTGCTTA

TGTCTACTTGGCAGGTAAAATTCCATTTCAAAAGTTAAATGTACTTAAAAAATTACCTAA

GACTGGGTAAATTAAAAAAATTAAATGTTGCAAAGAAAAAATTCAAAATTCTTATTCTTG

AATGAAAAACGTTCTCTTACTGGTGATTGAGGAGGAGAAACAAAGACTAACAAATGAAAA

TGGGAGAATCCACACTCAGAGTGGGGCAATTGAACAGGCAGGGGCGGATGGATGGCAGAG

GAGGAGGAATCTGGACTCAAGGAGCTGGGGGGCTCTGGGCCTGGAATTTTAGGGTCTGGG

GCCCAAGGCACCAGGAGAAGAGGCAGGTCAGGATATCTGAGTCAAGACCTGGGATCTTGC

CTTAGCAATGACACTGGAGACTAAAGGTGGACTCCATGGTGCCCTTGAGCCCAGCCCTAC

CCCATCTCCACTATCCTCTGCCACCAGCTGTGCAACTTCTGCTAGGGGTGAGGTTAATAA

ACTGGAGAAGTTAATTTGTGGAGCATGAAACAGATGAGCAGAACAATCACAGCACCTTAA

TTTCCCCAGTGTGCCCAAGAACAGAGCAGGCCTGAAGATACTCAAACAGAAACAAACATG

TGCCGTGTCACTGATAATTCTGTGTAGACACACACCTGCCAGACACTGCTCATGGCACTC

CCTAGGAAGAACAGCATGTGGGAAAGGCTGCCAAAATTGTTCATGTAAAAATTACATCAA

TGCTGTCTTCCTCGGTGCTGCCTATGCAGCTGGCAGCCATCTCTTCCTCCACATCATGGC

CTCCCTCAGACTCCTCATGAAGGATAAGATCCTCAAAAAGAGGACCAACAAGTTCATGAG

GCACCAATCAGACTGAAATGTCAAAATTAAGCATAACTGGCGGAAACCCAGAGGTCTTAA

CAGTAGGGTTCGTAGAAGGTCCAAGGGCCAGATCTTGATGCCCAACATTGCTTATGGGAG

CAACAACAACAACAAAAAAAACATGCTGCCCAGTGGCTTCCAGAAGTTTCTGGTCCACAG

CCTCAAGGAGCTGAAAGTGCTGCTGATGTGCAACAAATCTTACTGTGCTGAGATCGCTCA

CAAAATTTCCTCCAGAACTGCAAAGTCATCATGGAAAGAGTCACCCAGCCGGCCATCAGA

GTCACCAACCCCAGTACCAGGGTGCACAGCTAAGAAAATGAGTAGAAAGTTCATGTCCAC

GTTTTGTGTGTAAATAAAACCATAAAAACTGCCAAAAAAAATTACATCAATGCCTCTAAA

CCCAAAGGACTCTACCCCCACAGGTCCCTGGTTGTTGTGGTGATTTTCATTGTGTAAAAT

ACTTTCCACATCTTTTGACACCAAGTCTTTCTGCAGCCATGTTTGAAAATTAACTTTCAG

GCTACAGAGTCTTTCTTATACCAAAGTTGAAGAAAGTTTTAAGAAATATATTTCTACATC

TCCTACATGCAAAACAACAGGAGCAAGTTGAGGAATTCTCAAGAAACTGGTCGAGAAGAG

AGAGCGCTTAGCTATGGAAAAGAGAAAGAAGGAAGGGAGGGCTTCCTGGAGGAGGTGGCA

TTTGAACCAGGACTGACATCAGGATGGAAATGTCAGTCAGGGAGTTAAGTAGGGGGAGCA

GCTCCGCCCTCCACGTCCCCAGCTCCTCCCGCCCCTGTTTTTTCTCCCAGTGACCCCACG

TGAAACGTCTCCGCCTCCTCCAGCCACCAGCAGAAGGGACTGCCTTCCCCTCAGTGCTCG

CCCCTCCCTAGTGATCACTCAGTGCCCCTGAGCTCATTCTTTTCAGTAAATTCTCTCTCT

GCGTGGTGAGAAAACAGGCCTGGAGAGGCTCTGCGACCCGCTTAGGACCACAGAACTCGG

TACTAGGAAAACTCCTATTTTAAAATCCAGCCCTGGGTGGGAAGATTTGGGAAGAATCGT

TAATATTGAGAGAGAGAGGGAGAAAGAGGATTAGATGAGAGTGGCGCCTCCGCTCATGTC

CGCCCCCTCCCCGCAGAGAATTACCTTTTCCAGGGACGGCAGGAATGCTACGCGTTTAAT

GGGACACAGCGCTTCCTGGAGAGATACATCTACAACCGGGAGGAGTTCGCGCGCTTCGAC

AGCGACGTGGGGGAGTTCCGGGCGGTGACGGAGCTGGGGCGGCCTGCTGCGGAGTACTGG

AACAGCCAGAAGGACATCCTGGAGGAGAAGCGGGCAGTGCCGGACAGGATGTGCAGACAC

AACTACGAGCTGGGCGGGCCCATGACCCTGCAGCGCCGAGGTGAGTGAGGGCTTTGGGCC

GGCGGTCCCAGGGCAGCCCCGCGGGCCCGTGCCCAGGGCGCAGGAGCAGCCGGGTTGGCC

TAAGGGACCTTAGTGCCGGGCGGAAAGGGGACTTTGGGTTGGGGATTCATGGGGGGAGCC

CATCTGGAGCTTGTCAGGGGAGCGAGCGCGGGGACCTGGACTGGGCTGAGCATGGAGTGA

GGAGGACGAGAGCAGAGAGACCCCCGGGACTTCATCAGGCCTGGCAGCTGACTGCATGTG

GGGTGAAAAAAGGAAGCCACAGGACAGCGCACAAGGGTATGGTGTGGAGATGGAGGTGGA

GATGGCACAGCAGGCCACACAGAGAAGAAACCTACAGGGAGGTAGCTGGGTTTGAGGTGC

TTGAGGGGCAGATGGGTGGTCTGATGGGCAGGTAGACAGAAGGGTCTGCAGCCGGGGAGG

AGACTGAGATACATGAGACCATCCAGGGAGAGGGGACCCAGGGGGAAGAGCAAAGGACCG

GATCCTGGGAACTGGACAGTTGTGATTTGGCCAAGACAGAAAAGCCTGTGAAAGAGACCA

AAAAAACCCAAGTGCAGTGTGAGGAGAGGCCCGCAGAGAAGAGTCTTGGAAGCTGAGGGG

AGGTGACCTCAGCAGCACAGTGGACAGCGGTGCCAGTGACTTGGGAAGGTCAGAAAACAG

AAGATGGAAAGTGGGTTTGGAAACCAGGGAGACCTGGGGAGAGCAGGTTGGCCGCAGCGG

CAGGAGCTGGAATGGGAGGGGGTGCATGAGGCTGAGTGTGGCGCATCCTCCTCGGGGCTG

AGATGGATTTTACTTGTCTTGGGTTCCCCACGGCTGTCACAGGGCAGTGTCTCAGTTCAT

TCGTCTTTTTCCTTCAGGAAGTCTGGGTGTAAAGGGATGGAGAGAGGTGAGGTGTGTGCA

GTAAGAGGATTTCTCAAGGATGGGACAGGAAGGCCTTGGAGCTTTGGCTTCCTCCTGTGA

ACTTGTGGGGTGGGGAGCCTGGTGCACCAACCTGAGGGACTTGAGGGAGTAGTATCAGGA

TGTGGGATTGAGCCCTGGACCTTTTTTCTAGAAAGAGGAAAAAAATGAAGGGAGGAGGAG

GAGGAAGCTGGGGAGATCACACCTTTGATTTTCTTGTTCCTGGAAAGTGAAAGGAAGTTC

ACCTGCTATGAGTGAGAAGGTGGACACACTGGGTGGGGATGAGGTGAGTGACATGAGCTT

AGGAAAGTTGCTGAGGTAATTGGTTGAGAGAGGTGTTCAAATAAAAATAACGCAATTGGC

AAAAACTGTTACTAAGACTTTGTAGAGGCACCAATCAGTGACATGGCAGCATTTTCTTTC

ACAGTAATCAACTGCCAGATTGCAGACAGCCCTGATGCCAGCCTAAGGAGTGTGGGTTTC

TCCTCCAGGCCCGCAGGTCCCCAACCTCACTCCTCTGAAGACTCTTCTGGAGATCCTCTG

TGATGCACAGATCTCCAGACTCAGTGCCCCCAGACTCAGATTCCCTGGGTGGGGAGGTCT

GGGGATCTCTGCTTGTAATCAGCTCCCTAGAGGTTCCCATGTAGCCAGATAAGTATTGTC

AGAACACTGAAGATTTTTGAAAAATGAAAAAGAGAAGGTTGGAGATGTGTCTTCAGAAGA

CTACTAAGGGTGCTGGCTAGAGGAGGGACCAGAGGCAGGGAGATGAGGTAGGAAACTGCT

ATTATTTGTCAGGGAAATTGCAATCAAGGCATGAGTTAGAACAGGGAAAACACAGAGGCA

AGGGAGAGGTGGAAGGGGGAGGAAAGAAGTAGTGACAATTCCAGGGTGGATGTCCACCCA

AATCTAGAAGTAATTGAGCAAATGTTTTCTGGGCATTAGAGAAGGCAACTAGAACAAACA

GGAATCCTTGCCTTGGTGAAATGTATTTGAACTGGGTCAGAAATGAGGCCATTGGGTATC

AGGCCTTAACTCCAGCGCACCCTGGAGGTCACTGATGTGGCTCCAGGCTGACCTGCTCCT

GTCAAAGAATATTGAGCAAGATGCCTCTCGTGGAATGTTCTGGGACCTTAAAACAGATAC

CCAAGTATTCCCCCTGATTTCATGGTTCCCAGAAGCTCTATGGGGAAGAAATTGTAGGTA

ATTCACAACTGAGATTTAGACATAAGTTGAATAGTGTAATGGACATTGAGTTAACCGAGG

TAATGAAGTAGTGAGACACAGGTGCCCCTGAAATAAACTCACATTGAGGGAAGAGGCTGA

CAATGTGGATCAGTCTGAAAACAAGGCAAAAATACAATAGGGAGTAAGGGTTGTGTGTCA

GTTCAAGACTGTACTTTTACCTGGCCCAGCGCCATGTTAGGGTATTTGTGTTCTCCAGGA

AGTAGAAAGGAAAGAACTGAGTGATTAGGGACCTAGAAGACTAATTTGAGACATTCCTCT

TGATGAGCTGTTCTCTAGGGTAGTCCTCTGAAAGAGCTGTTCTCTAGTGGATCTCCCTGA

ATGAACTGTTCTCTAGGAGCACTTGACCCTTTTCTGTGTTTGTTTTTTGTTTTGTGTTTG

TGTTTGTTTTTGAGACAGGTTCTCACTTTGTCTCCCAGGCTGGAGTGCTGTGGCACCATC

ATGGCTCACTGCAGCCTCAACCTCCTGGGCTCAAGTGATCCTCCTGCCTCAGCCTCCCAT

GTAGCTAGAACTACAGATACACGTACCACCATGTCTGGCTAATTTATTTTTCTTTTTAGA

GATGGGTTCTCACTATGTTGCCCAGGCCGGTCTCAAAACCCTGGGCTCAAGTGATCCTCA

TGCCTCAACCTCCCAAAGTGCTAAGATTATAGGCATGACCACCATGCCTGGCCTTTTCTG

CTTTCTGAGGAGGAAAAAGGTACTGGTGGCAGAGATCCAAAAGAAAAGTTGCCAGTGGCA

GTGTGGAAATTCACCTGAGAACAACAGGACAAGCTGGGGCACAAATGCAAAGATGCAGAG

GGAGGCAACACCTGGTCATCTGTGAGACCTTCATGGGACCTGAAGACGCAGCACAGAGGA

GGAACTTGAAAAAGGACGGGATTTCTACTACTCAAGCATGTAGGAGCTCAGGATATTCTG

TAAATATGAAGATTTTGAGTTTTTGTAGGTGAGGTAAAAAAATACATAGGTTTTTTACAG

AATAAGACATGTAAAGCTCTCTTCATTTTCTTTGTATTTTCATGAAGTTATTAGATTCAC

AGGCCACCATAATGCCATTGTCTGTATATCTTAATTTCAAGATATTATTTGAGTAAATTT

TGCTTCCTTTGTATCAAGATAGAACTTTGAAAAGGTAGGTAATTTCACAGTTGATCAAAT

ATTCTTTGCCCAAATTACTTTTGGTTAAAATTTCTCCTAAATGTGCTACAGAGTGCAAAC

TCTGTCTCCCTGCCATTCCGCTATATACTTACTAACTATTATTTTATTCAAGATCATGCA

TGCTCTACTTGAAGGTCTATTTCTATCTTTTCAATGCTACCCTTACCCACTAGCCTAATC

ACATTATTCCTATTTTCAACATCTAGGAATCAATTACATAGTGAACATGCCTAAGAAATA

ATAATCTGGGCAGATGCAGTGGCTCAGGCCCGTAATCCCAGCCCTTTGAGAGGCCGAGCG

GGTGGATCACTTGAGGTCAGGCGTTGGTCAAGTGCTCCTAGAGAACCAGGCTGACCAACA

TGGAGAAACCTTGTCTCTACTAATAATACAAAAATTAGCCAGGTGAAGTGGCAGGCACCT

ATAATCCCAGCTATTCGGGAGGCTGAGGAAGGAGAATTGGTTGAAGCCCGGAGGTGGAGG

TTGCAGTGAGCCAATATTGCGCCACTGCATTCCAGACTTGGCAACAGAGTGACACTCCAT

CTCAACAAAAAGAAAGAATGAAAGAAAGAAAGAGCGAGATTATGTCTCAAAAAAAAGGAA

GGAAGGAAGGAAGGAAGGAAGGAAGGAAGGAAGGAAGGAAGGAAAGAAGGACAATCTCAA

ATTCTATTTCATTATTTTTCTTCCACGCTCCTAGTCCAGCCTAGGGTGAATGTTTCCCCC

TCCAAGAAGGGGCCCTTGCAGCACCACAACCTGCTTGTCTGCCACGTGACGGATTTCTAC

CCAGGCAGCATTCAAGTCCGATGGTTCCTGAATGGACAGGAGGAAACAGCTGGGGTCGTG

TCCACCAACCTGATCCGTAATGGAGACTGGACCTTCCAGATCCTGGTGATGCTGGAAATG

ACCCCCCAGCAGGGAGATGTCTACACCTGCCAAGTGGAGCACACCAGCCTGGATAGTCCT

GTCACCGTGGAGTGGAGTGAGTCTCTGATGACCCTCTAGACCCCACCTCTGAAGAGCAGG

GGACTCTCTGGCTCTGGGGTCCACTCATCTTATCTTCTGCATCTATACCCTGGGGCCATG

TCCAAACCCCATCTTTCTTCTATACCAGCTCCTGAGCATAGTTTGAAGCCAGGGAAATGG

AGACTTCCTGACCTTGGCTTAGGGGTTCCTGAAGATTCATAGTTCTCCCCCTTGTCAGAG

AATCTAGGGACACTGACTGGTCTCGAAACCCTCACACTTAGGAACTGACCTCACACATAG

GAACAGTTCTCTTCCTTCAGCATTTTAGCCTCTTCTCAGGCATTTTGAGAGGCAACTTCC

AGAATCAGCATTTGCCACCTTGTTGAGGTCACACCCCTGTTCCAGATATGAGGGTGGCTC

TTTCTGAATTTCCTCTTAGCAAGCTTTTTCCGCTGCACTGTCCTCATCCCGATATGCTGC

ATCAGGCTCCAGAATCTCAGACAGGACATGAGTAGGGATGCAGCTGGTGGAGGTGACACT

AAACCTGGGTCTGTCCTTCCCAGAGGCACAGTCTGATTCTGCCCGGAGTAAGACATTGAC

GGGAGCTGGGGGCTTCGTGCTGGGGCTCATCATCTGTGGAGTGGGCATCTTCATGCACAG

GAGGAGCAAGAAAGGTGAGAAAGCCTGCAGGGTGAGCGGGACTTACCTTCCCCTGGCATA

TTCACACTTATTCCACGATGAGGGGTTTGACAGAAAAGAAATGTCAGAAAGCTCTAGAGG

CCACTGATATCAGATAATCGGGGAACAAACATGACCTATAGCGAGAGAGGGATCCCAGGC

TGGGATCTTAATGCAGCCAGATGCATGAGGTCCCAAGTACTCAGGCTCCTGCGGAGCGTC

CATTGAGTGATGGGCAATGGAATTTGGTGGGATGGAAATGTTTCTCTAATTATCTGAGGT

GGTTTCAATGGCTGATTATATAACCTTTCGTCTTTCATTTCAGTTCAACGAGGATCTGCA

TAAACAGGTAATATTCCTGCTTTGATTTCCTTGTGGGGTGGGTTGCAGGAGGATATGAGT

CCTTTCTGTGCATTGTAACACTGAGGCTCCTCCAGGAAGGGAATCTCAGGCATGAACCCC

TCTTTCAATGTCAGCCTTCAGGCAAGTGGGGAAAGAGCATTGCTTGGCTCCATTGCTGAA

GGAAGCAGAGATCAACTCTGTTATTTATCAGCCTGAGACGCATCCTCTCACCATAATTTT

TCTCTCCTGGACTTACAGGAAGGAGGCTGGCAACCTGGGATAACTTGTCTTTTACCCCCA

CAGGGTTCCTGAGCTCACTGAAAAGACTATTGTGCCTTAGGAAAAGCATTTGCTGTGTTT

CGTTAGCATCTGGCTCCAGGACAGACCTTCAACTTCCAAATTGGATACTGCTGCCAAGAA

GTTGCTCTGAAGTCAGTTTCTATCATTCTGCTCTTTGATTCAAAGCACTGTTTCTCTCAC

TGGGCCTCCAACCATGTTCCCTTCTTCTTAGCACCACAAATAATCAAAACCCAACATGAC

TGTTTGTTTTCCTTTAAAAATATGCACCAAATCATCTCTCATCACTTTTCTCTGAGGGTT

TTAGTAGACAGTAGGAGTTAATAAAGAAGTTCATTTTGGTTTAAACATAGGAAAGAAGAG

AACCATGAAAATGGGGATATGTTAACTATTGTATAATGGGGCCTGTTACACATGACACTC

TTCTGAATTGACTGTATTTCAGTGAGCTGCCCCCAAATCAAGTTTAGTGCCCTCATCCAT

TTATGTCTCAGACCACTATTCTTAACTATTCAATGGTGAGCAGACTGCAAATCTGCCTGA

TAGGACCCATATTCCCACAGCACTAATTCAACATATACCTTACTGAGAGCATGTTTTATC

ATTACCATTAAGAAGTTAAATGAACATCAGAATTTAAAATCATAAATATAATCTAATACA

CTTTAA

>HLA:HLA00522 DPB1*04:02:01:01

TAATCCCTGTAGATGGGCCAGCAGAATATTTGAGATCACCTTCAGAGCAAAGAAAACGCA

TAATCTCCCCAAACATCATGACTTATCTGACTGGTTAAAATGAGTATCACTGTCTTTCCT

CCGTCATCTTAAGTGCATCACAGGCTTTATATTTTCAGACCTTTCATACTAACTTTCTGC

CTAGTGAGCAATGACTCATACAAAGCTCAGTGTCCATTGGTTCTTTTCTCAGACTCTGTC

CAATCCCAGGGTCACAGAAGACTACTTGGGTTCATGGTCTCTAATATTTCAAACAGGAGC

TCCCTTTAGCGAGTCCTTCTTTTCCTGACTGCAGCTCTTTTCATTTTGCCATCCTTTTCC

AGCTCCATGATGGTTCTGCAGGTTTCTGCGGCCCCCCGGACAGTGGCTCTGACGGCGTTA

CTGATGGTGCTGCTCACATCTGTGGTCCAGGGCAGGGCCACTCCAGGTAAGAGCCGAACT

GCCATTCTTGGAGGGTCTGGCTCAGGGAACAATTCCTAGGGGACGTTATCTTTAAGGGAT

CAAATTCTGAGACAGGCTGCGGGGGCTCCTGCCCTAAGGCAGTGTCCTCTCTTCCCAGCT

AGAGAAAGAGGTTCATCCCCTATAGGATAGCTTGCTACCCTACTGGCCTATTCTCTCTCC

AAGGACATGGGTACAGTAAACAGAGAGAGGTGCCCAGTGGTCAGTATGCTTGTCTTTGGG

GAAAATGGGACCAAGAGGTCCTGGATAACCTTGGACAGACAAGGTTTGCAGAGAGAGAAG

TTGGCAAGTGCAGGCTCCTGGGCGTGTTCATGTCTGCATCCAGCCTGGAGGGGACTCAGG

CAGAGAGCCCTAAGCTGGAGTGTCCAGGCTCTGAGGATCACTGAGGATTCAGTGCTCACG

AAGAATGCCTCTTATTCCCCAGGGTGGAGCAGGAGCCCACATCCCTTGGACAATTAAGGA

GAGAAGGGAGGGAGGGGGATAGGTTTTAGCCCCTGAAGGCATTCTCATTAAAGGTACTTC

TCCCAGCCTCCCCAGAACTTGGTTAGGGTACTAGAGTGGGTTGCGACTTGTAGGAAGAAT

GAGATGAGGTTGTGTGGGTGCATGACAGGGATTGAGTGTAGGTTATCAGACAGCCAAGGA

AGCAGTAACCAAGTGAAAAATCTCTTCTTCCTGCTGCCTCCCTGTGGCTGGTGTAATATT

ATGGCATCTATGATCCATTGTTTTTCTCTCAGGATACTCTCAGGATATTTCTTTTTATAT

ATATATATACTTTAAGTTCTAGGGTACATGTGCACAACGTGCAGGTTTGTTACATATGTA

TACATGTGCCATGTTGGTGTGCTGCACCCATTAACTCGTCATTTACATTAGGTATATTTC

CTAATGCTATCCCTCCCCCCTCCCCCCACCCCACAACAGGCCCCGGTGTATGATGTTCCC

CTTCCTGTGTCCATGTGTTCTCATTGTTCAGTTCCCACCTATGAGTGAGAACATGTGGTC

TTTGGTTTTCTGTCCTTGCAATAGTTTGCTGTGAATGATGGTTTCCAGCTTCCTCCATGT

CCCTACAAAGGACATGAACTCATCCTTTTTTATGGCTGCACAGTATTCCATGGTGTATAT

GTGTGCATTTTCTTAATCCAGTCTATCACTGATGGACAGTTGGGTTGGTTCCAAGTCTTT

GCTATTGTGAATAGTGCCGCTATAAACATATGTGTGCATGTGTCTTTATAGCAGCATGAT

TTATAATCCTTTGGGTATATACCCAGTAATGGGATGGCTGGGTCAAATGGTATTTCTAGT

TCTAGATCCTTGAGGAATTGCCACAATGTCTTGAGATACCATCTCACACCAGTTAAAATG

GCGATCATTAAAAAGTCAGGAAACAACAGGTGCTGGAGAGGATGTGGAGAAATAGGAACA

CTTTTACTCTGTTGGTGGGACTGTAAACTAGTTCAACCATTGTACTCTCAGGACATTTCT

AGTCCAAATTTACACCAACACTCTGAGAGGAAGGACTGCAAAGTAGGTACCTTAGTTTTC

CACTGACTTCCACTTTTCCTGCTTACACCCTTCCTCCTAGACCTCTCCACACCCCTCCTA

GGACACACCTAAAAGGTACTGACATCATGTCACCTCCTCATCTTTCAGGGTAGCAAGGTT

GGAATCTCCTGAATACAGCCCCTCAAGCCCTAAAACCTCTTATCTATTACCTTGGGTTCA

TTGTCCAGGAAGGGGAGGAGAACTTGAACTTGTAGTCACAGAAGGGTGCTGAGAACTAAC

CAGCAGGACGGCTCAGCCCTGGGAACTGCAGAGGGGTGAGGCTGGGGAGAGAGGAGGCTG

GAGCAGCACTGGTGACACTGAACAGTGTCAGGAGGAAGTGACGGATGCAGCGCCCCCATC

CCATAGGCAGAGCTGTCATGTGGGATGAGGGACAGTGTTGGGAGCCACCAAGGAAACCCA

GAGGTGGGGGAGCAGAGAGCAGAAGGGAGCATGTGATGCTGGACAGTGAAAGGGAGGACA

GGCAAAGGCTGGGTTGAGGTTTGTAGGGGGAATGAGATGAGGCAGTGGAGCCATGTGACA

GGGACTGAGGGTAGATTACTGGAGCTCCCTGCGTAGAATGAATGTTCAATCAAAATTTGC

TGGAGGGAGAGCTGGAGCCATAGGGGAGTGGGTAAAGTGGGCAGGGCTGATTCCACAATT

CCCTGCATGCTCCCCCAACTCCACACACATCCCCAACCTCAAACAGGGCACAAGACCAAA

GGGCTGAGGAGCCAGGCTATAGCTTAAAGAGGCTGGGGGAGAAAAGCTTGGCTGAGACAA

CCCATAGGGAGCTAGAGGTTTTTAATATATCCTATTCTGAATAAGAGACGAATTCATTCA

GATCAGTGGTTTCAAACCGTGCTCTGGGCAACTCAATTGCTAAGGGTTCCACAAACAGGA

TAAAGTTTCTTATATACAAAAAAAAATGAAGGTTTCAAATTACACCATAAAACCCCTCAT

TGCTTATGTCTACTTGGCAGGTAAAATTCCATTTCAAAAGTTAAATGTACTTAAAAAATT

ACCTAAGACTGGGTAAATTAAAAAAATTAAATGTTGCAAAGAAAAAATTCAAAATTCTTA

TTCTTGAATGAAAAACGTTCTCTTACTGGTGATTGAGGAGGAGAAACAAAGACTAACAAA

TGAAAATGGGAGAATCCACACTCAGAGTGGGGCAACTGAACAGGCAGGGGCGGATGGATG

GCAGAGGAGGAGGAATCTGGACTCAAGGAGCTGGGGGGCTCTGGGCCTGGAATTTTAGGG

TCTGGGGCCCAAGGCACCAGGAGAAGAGGCAGGTCAGGATATCTGAGTCAAGACCTGGGA

TCTTGCCTTAGCAATGACACTGGAGACTAAAGGTGGACTCCATGGTGCCCTTGAGCCCAG

CCCTACCCCATCTCCACTATCCTCTGCCACCAGCTGTGCAACTTCTGCTGGGGGTGAGGT

TAATAAACTGGAGAAGTTAATTTGTGGAGCATGAAACAGATGAGCAGAACAATCACAGCA

CCTTAATTTCCCCAGTGTGCCCAAGAACAGAGCAGGCCTGAAGATACTCAAACAGAAACA

AACATGTGCCGTGTCACTGATAATTCTGTGTAGACACACACCTGCCAGACACTGCTCATG

GCACTCCCTAGGAAGAACAGCATGTGGGAAAGGCTGCCAAAATTGTTCATGTAAAAATTA

CATCAATGCTGTCTTCCTCGGTGCTGCCTATGCAGCTGGCAGCCATCTCTTCCTCCACAT

CATGGCCTCCCTCAGACTCCTCATGAAGGATAAGATCCTCAAAAAGAGGACCAACAAGTT

CATGAGGCACCAATCAGACTGAAATGTCAAAATTAAGCATAACTGGCGGAAACCCAGAGG

TCTTAACAGTAGGGTTCGTAGAAGGTCCAAGGGCCAGATCTTGATGCCCAACATTGCTTA

TGGGAGCAACAACAACAACAAAAAAAACATGCTGCCCAGTGGCTTCCAGAAGTTTCTGGT

CCACAGCCTCAAGGAGCTGAAAGTGCTGCTGATGTGCAACAAATCTTACTGTGCTGAGAT

CGCTCACAAAATTTCCTCCAGAACTGCAAAGTCATCATGGAAAGAGTCACCCAGCCGGCC

ATCAGAGTCACCAACCCCAGTACCAGGGTGCACAGCTAAGAAAATGAGTAGAAAGTTCAT

GTCCACGTTTTGTGTGTAAATAAAACCATAAAAACTGCCAAAAAAAATTACATCAATGCC

TCTAAACCCAAAGGACTCTACCCCCACAGGTCCCTGGTTGTTGTGGTGATTTTCATTGTG

TAAAATACTTTCCACATCTTTTGACACCAAGTCTTTCTGCAGCCATGTTTGAAAATTAAC

TTTCAGGCTACAGAGTCTTTCTTATACCAAAGTTGAAGAAAGTTTTAAGAAATATATTTC

TACATCTCCTACATGCAAAACAACAGGAGCAAGTTGAGGAATTCTCAAGAAACTGGTCGA

GAAGAGAGAGCGCTTAGCTATGGAAAAGAGAAAGAAGGAAGGGAGGGCTTCCTGGAGGAG

GTGGCATTTGAACCAGGACTGACATCAGGATGGAAATGTCAGTCAGGGAGTTAAGTAGGG

GGAGCAGCTCCGCCCTCCACGTCCCCAGCTCCTCCCGCCCCTGTTTTTTCTCCCAGTGAC

CCCACGTGAAACGTCTCCGCCTCCTCCAGCCACCAGCAGAAGGGACTGCCTTCCCCTCAG

TGCTCGCCCCTCCCTAGTGATCACTCAGTGCCCCTGAGCTCATTCTTTTCAGTAAATTCT

CTCTCTGCGTGGTGAGAAAACAGGCCTGGAGAGGCTCTGCGACCCGCTTAGGACCACAGA

ACTCGGTACTAGGAAAACTCCTATTTTAAAATCCAGCCCTGGGTGGGAAGATTTGGGAAG

AATCGTTAATATTGAGAGAGAGAGGGAGAAAGAGGATTAGATGAGAGTGGCGCCTCCGCT

CATGTCCGCCCCCTCCCCGCAGAGAATTACCTTTTCCAGGGACGGCAGGAATGCTACGCG

TTTAATGGGACACAGCGCTTCCTGGAGAGATACATCTACAACCGGGAGGAGTTCGTGCGC

TTCGACAGCGACGTGGGGGAGTTCCGGGCGGTGACGGAGCTGGGGCGGCCTGATGAGGAG

TACTGGAACAGCCAGAAGGACATCCTGGAGGAGAAGCGGGCAGTGCCGGACAGGATGTGC

AGACACAACTACGAGCTGGGCGGGCCCATGACCCTGCAGCGCCGAGGTGAGTGAGGGCTT

TGGGCCGGCGGTCCCAGGGCAGCCCCGCGGGCCCGTGCCCAGGGCGCAGGAGCAGCCGGG

TTGGCCTAAGGGACCTTAGTGCCGGGCGGAAAGGGGACTTTGGGTTGGGGATTCATGGGG

GGAGCCCATCTGGAGCTTGTCAGGGGAGCGAGCGCGGGGACCTGGACTGGGCTGAGCATG

GAGTGAGGAGGACGAGAGCAGAGAGACCCCCGGGACTTCATCAGGCCTGGCAGCTGACTG

CATGTGGGGTGAAAAAAGGAAGCCACAGGACAGCGCACAAGGGTATGGTGTGGAGATGGA

GGTGGAGATGGCACAGCAGGCCACACAGAGAAGAAACCTACAGGGAGGTAGCTGGGTTTG

AGGTGCTTGAGGGGCAGATGGGTGGTCTGATGGGCAGGTAGACAGAAGGGTCTGCAGCCG

GGGAGGAGACTGAGATACATGAGACCATCCAGGGAGAGGGGACCCAGGGGGAAGAGCAAA

GGACCGGATCCTGGGAACTGGACAGTTGTGATTTGGCCAAGACAGAAAAGCCTGTGAAAG

AGACCAAAAAAACCCAAGTGCAGTGTGAGGAGAGGCCCGCAGAGAAGAGTCTTGGAAGCT

GAGGGGAGGTGACCTCAGCAGCACAGTGGACAGCGGTGCCAGTGACTTGGGAAGGTCAGA

AAACAGAAGATGGAAAGTGGGTTTGGAAACCAGGGAGACCTGGGGAGAGCAGGTTGGCCG

CAGCGGCAGGAGCTGGAATGGGAGGGGGTGCATGAGGCTGAGTGTGGCGCATCCTCCTCG

GGGCTGAGATGGATTTTACTTGTCTTGGGTTCCCCACGGCTGTCACAGGGCAGTGTCTCA

GTTCATTCGTCTTTTTCCTTCAGGAAGTCTGGGTGTAAAGGGATGGAGAGAGGTGAGGTG

TGTGCAGTAAGAGGATTTCTCAAGGATGGGACAGGAAGGCCTTGGAGCTTTGGCTTCCTC

CTGTGAACTTGTGGGGTGGGGAGCCTGGTGCACCAACCTGAGGGACTTGAGGGAGTAGTA

TCAGGATGTGGGATTGAGCCCTGGACCTTTTTTCTAGAAAGAGGAAAAAAATGAAGGGAG

GAGGAGGAGGAAGCTGGGGAGATCACACCTTTGATTTTCTTGTTCCTGGAAAGTGAAAGG

AAGTTCACCTGCTATGAGTGAGAAGGTGGACACACTGGGTGGGGATGAGGTGAGTGACAT

GAGCTTAGGAAAGTTGCTGAGGTAATTGGTTGAGAGAGGTGTTCAAATAAAAATAACGCA

ATTGGCAAAAACTGTTACTAAGACTTTGTAGAGGCACCAATCAGTGACATGGCAGCATTT

TCTTTCACAGTAATCAACTGCCAGATTGCAGACAGCCCTGATGCCAGCCTAAGGAGTGTG

GGTTTCTCCTCCAGGCCCGCAGGTCCCCAACCTCACTCCTCTGAAGACTCTTCTGGAGAT

CCTCTGTGATGCACAGATCTCCAGACTCAGTGCCCCCAGACTCAGATTCCCTGGGTGGGG

AGGTCTGGGGATCTCTGCTTGTAATCAGCTCCCTAGAGGTTCCCATGTAGCCAGATAAGT

ATTGTCAGAACACTGAAGATTTTTGAAAAATGAAAAAGAGAAGGTTGGAGATGTGTCTTC

AGAAGATTACTAAGGGTGCTGGCTAGAGGAGGGACCAGAGGCAGGGAGATGAGGTAGGAA

ACTGCTATTATTTGTCAGGGAAATTGCAATCAAGGCATGAGTTAGAACAGGGAAAACACA

GAGGCAAGGGAGAGGTGGAAGGGGGAGGAAAGAAGTAGTGACAATTCCAGGGTGGATGTC

CACCCAAATCTAGAAGTAATTGAGCAAATGTTTTCTGGGCATTAGAGAAGGCAACTAGAA

CAAACAGGAATCCTTGCCTTGGTGAAATGTATTTGAACTGGGTCAGAAATGAGGCCATTG

GGTATCAGGCCTTAACTCCAGCGCACCCTGGAGGTCACTGATGTGGCTCCAGGCTGACCT

GCTCCTGTCAAAGAATATTGAGCAAGATGCCTCTCGTGGAATGTTCTGGGACCTTAAAAC

AGATACCCAAGTATTCCCCCTGATTTCATGGTTCCCAGAAGCTCTATGGGGAAGAAATTG

TAGGTAATTCACAACTGAGATTTAGACATAAGTTGAATAGTGTAATGGACATTGAGTTAA

CCGAGGTAATGAAGTAGTGAGACACAGGTGCCCCTGAAATAAACTCACATTGAGGGAAGA

GGCTGACAATGTGGATCAGTCTGAAAACAAGGCAAAAATACAATAGGGAGTAAGGGTTGT

GTGTCAGTTCAAGACTGTACTTTTACCTGGCCCAGCGCCATGTTAGGGTATTTGTGTTCT

CCAGGAAGTAGAAAGGAAAGAACTGAGTGATTAGGGACCTAGAAGACTAATTTGAGACAT

TCCTCTTGATGAGCTGTTCTCTAGGGTAGTCCTCTGAAAGAGCTGTTCTCTAGTGGATCT

CCCTGAATGAACTGTTCTCTAGGAGCACTTGACCCTTTTCTGTGTTTGTTTTTTGTTTTG

TGTTTGTGTTTGTTTTTGAGACAGGTTCTCACTTTGTCTCCCAGGCTGGAGTGCTGTGGC

ACCATCATGGCTCACTGCAGCCTCAACCTCCTGGGCTCAAGTGATCCTCCTGCCTCAGCC

TCCCATGTAGCTAGAACTACAGATACACGTACCACCATGTCTGGCTAATTTATTTTTCTT

TTTAGAGATGGGTTCTCACTATGTTGCCCAGGCCGGTCTCAAAACCCTGGGCTCAAGTGA

TCCTCATGCCTCAACCTCCCAAAGTGCTAAGATTATAGGCATGACCACCATGCCTGGCCT

TTTCTGCTTTCTGAGGAGGAAAAAGGTACTGGTGGCAGAGATCCAAAAGAAAAGTTGCCA

GTGGCAGTGTGGAAATTCACCTGAGAACAACAGGACAAGCTGGGGCACAAATGCAAAGAT

GCAGAGGGAGGCAACACCTGGTCATCTGTGAGACCTTCATGGGACCTGAAGACGCAGCAC

AGAGGAGGAACTTGAAAAAGGACGGGATTTCTACTACTCAAGCATGTAGGAGCTCAGGAT

ATTCTGTAAATATGAAGATTTTGAGTTTTTGTAGGTGAGGTAAAAAAATACATAGGTTTT

TTACAGAATAAGACATGTAAAGCTCTCTTCATTTTCTTTGTATTTTCATGAAGTTATTAG

ATTCACAGGCCACCATAATGCCATTGTCTGTATATCTTAATTTCAAGATATTATTTGAGT

AAATTTTGCTTCCTTTGTATCAAGATAGAACTTTGAAAAGGTAGGTAATTTCACAGTTGA

TCAAATATTCTTTGCCCAAATTACTTTTGGTTAAAATTTCTCCTAAATGTGCTACAGAGT

GCAAACTCTGTCTCCCTGCCATTCCGCTATATACTTACTAACTATTATTTTATTCAAGAT

CATGCATGCTCTACTTGAAGGTCTATTTCTATCTTTTCAATGCTACCCTTACCCACTAGC

CTAATCACATTATTCCTATTTTCAACATCTAGGAATCAATTACATAGTGAACATGCCTAA

GAAATAATAATCTGGGCAGATGCAGTGGCTCAGGCCCGTAATCCCAGCCCTTTGAGAGGC

CGAGCGGGTGGATCACTTGAGGTCAGGCGTTGGTCAAGTGCTCCTAGAGAACCAGGCTGA

CCAACATGGAGAAACCTTGTCTCTACTAATAATACAAAAATTAGCCAGGTGAAGTGGCAG

GCACCTATAATCCCAGCTATTCGGGAGGCTGAGGAAGGAGAATTGGTTGAAGCCCGGAGG

TGGAGGTTGCAGTGAGCCAATATTGCGCCACTGCATTCCAGACTTGGCAACAGAGTGACA

CTCCATCTCAACAAAAAGAAAGAATGAAAGAAAGAAAGAGCGAGATTATGTCTCAAAAAA

AAGGAAGGAAGGAAGGAAGGAAGGAAGGAAGGAAAGAAGGACAATCTCAAATTCTATTTC

ATTATTTTTCTTCCACGCTCCTAGTCCAGCCTAGGGTGAATGTTTCCCCCTCCAAGAAGG

GGCCCTTGCAGCACCACAACCTGCTTGTCTGCCACGTGACGGATTTCTACCCAGGCAGCA

TTCAAGTCCGATGGTTCCTGAATGGACAGGAGGAAACAGCTGGGGTCGTGTCCACCAACC

TGATCCGTAATGGAGACTGGACCTTCCAGATCCTGGTGATGCTGGAAATGACCCCCCAGC

AGGGAGATGTCTACACCTGCCAAGTGGAGCACACCAGCATGGATAGTCCTGTCACCGTGG

AGTGGAGTGAGTCTCTGATGACCCTCTAGACCCCACCTCTGAAGAGCAGGGGACTCTCTG

GCTCTGGGGTCCACTCATCTTATCTTCTGCATCTATACCCTGGGGCCATGTCCAAACCCC

ATCTTTCTTCTATACCAGCTCCTGAGCATAGTTTGAAGCCAGGGAAATGGAGACTTCCTG

ACCTTGGCTTAGGGGTTCCTGAAGATTCATAGTTCTCCCCCTTGTCAGAGAATCTAGGGA

CACTGACTGGTCTCGAAACCCTCACACTTAGGAACTGACCTCACACATAGGAACAGTTCT

CTTCCTTCAGCATTTTAGCCTCTTCTCAGGCATTTTGAGAGGCAACTTCCAGAATCAGCA

TTTGCCACCTTGTTGAGGTCACACCCCTGTTCCAGATATGAGGGTGGCTCTTTCTGAATT

TCCTCTTAGCAAGCTTTTTCCGCTGCACTGTCCTCATCCCGATATGCTGCATCAGGCTCC

AGAATCTCAGACAGGACATGAGTAGGGATGCAGCTGGTGGAGGTGACACTAAACCTGGGT

CTGTCCTTCCCAGAGGCACAGTCTGATTCTGCCCGGAGTAAGACATTGACGGGAGCTGGG

GGCTTCGTGCTGGGGCTCATCATCTGTGGAGTGGGCATCTTCATGCACAGGAGGAGCAAG

AAAGGTGAGAAAGCCTGCAGGGTGAGCGGGACTTACCTTCCCCTGGCATATTCACACTTA

TTCCACGATGAGGGGTTTGACAGAAAAGAAATGTCAGAAAGCTCTAGAGGCCACTGATAT

CAGATAATCGGGGAACAAACATGACCTATAGCGAGAGAGGGATCCCAGGCTGGGATCTTA

ATGCAGCCAGATGCATGAGGTCCCAAGTACTCAGGCTCCTGCGGAGCGTCCATTGAGTGA

TGGGCAATGGAATTTGGTGGGATGGAAATGTTTCTCTAATTATCTGAGGTGGTTTCAATG

GCTGATTATATAACCTTTCGTCTTTCATTTCAGTTCAACGAGGATCTGCATAAACAGGTA

ATATTCCTGCTTTGATTTCCTTGTGGGGTGGGTTGCAGGAGGATATGAGTCCTTTCTGTG

CATTGTAACACTGAGGCTCCTCCAGGAAGGGAATCTCAGGCATGAACCCCTCTTTCAATG

TCAGCCTTCAGGCAAGTGGGGAAAGAGCATTGCTTGGCTCCATTGCTGAAGGAAGCAGAG

ATCAACTCTGTTATTTATCAGCCTGAGACGCATCCTCTCACCATAATTTTTCTCTCCTGG

ACTTACAGGAAGGAGGCTGGCAACCTGGGATAACTTGTCTTTTACCCCCACAGGGTTCCT

GAGCTCACTGAAAAGACTATTGTGCCTTAGGAAAAGCATTTGCTGTGTTTCGTTAGCATC

TGGCTCCAGGACAGACCTTCAACTTCCAAATTGGATACTGCTGCCAAGAAGTTGCTCTGA

AGTCAGTTTCTATCATTCTGCTCTTTGATTCAAAGCACTGTTTCTCTCACTGGGCCTCCA

ACCATGTTCCCTTCTTCTTAGCACCACAAATAATCAAAACCCAACATGACTGTTTGTTTT

CCTTTAAAAATATGCACCAAATCATCTCTCATCACTTTTCTCTGAGGGTTTTAGTAGACA

GTAGGAGTTAATAAAGAAGTTCATTTTGGTTTAAACATAGGAAAGAAGAGAACCATGAAA

ATGGGGATATGTTAACTATTGTATAATGGGGCCTGTTACACATGACACTCTTCTGAATTG

ACTGTATTTCAGTGAGCTGCCCCCAAATCAAGTTTAGTGCCCTCATCCATTTATGTCTCA

GACCACTATTCTTAACTATTCAATGGTGAGCAGACTGCAAATCTGCCTGATAGGACCCAT

ATTCCCACAGCACTAATTCAACATATACCTTACTGAGAGCATGTTTTATCATTACCATTA

AGAAGTTAAATGAACATCAGAATTTAAAATCATAAATATAATCTAATACACTTTAA

>HLA:HLA00530 DPB1*13:01:01:01

TAATCCCTGTAGATGGGCCAGCAGAATATTTGAGATCACCTTCAGAGCAAAGAAAACGCA

TAATCTCCCCAAACATCATGACTTATCTGACTGGTTAAAATGAGTATCACTGTCTTTCCT

CCGTCATCTTAAGTGCATCACAGGCTTTATATTTTCAGACCTTTCATACTAACTTTCTGC

CTAGTGAGCAATGACTCATACAAAGCTCAGTGTCCATTGGTTCTTTTCTCAGACTCTGTC

CAATCCCAGGGTCACAGAAGACTACTTGGGTTCATGGTCTCTAATATTTCAAACAGGAGC

TCCCTTTAGCGAGTCCTTCTTTTCCTGACTGCAGCTCTTTTCATTTTGCCATCCTTTTCC

AGCTCCATGATGGTTCTGCAGGTTTCTGCGGCCCCCCGGACAGTGGCTCTGACGGCGTTA

CTGATGGTGCTGCTCACATCTGTGGTCCAGGGCAGGGCCACTCCAGGTAAGAGCCGAGCT

GCCATTCTTGGAGGGTCTGGCTCAGGGAACAATTCCTAGGGGACGTTATCTTTAAGGGAT

CAAATTCTGAGACAGGCTGCGGGGGCTCCTGCCCTAAGGCAGTGTCCTCTCTTCCCAGCT

AGAGAAAGAGGTTCATCCCCTATAGGATAGCTTGCTACCCTACTGGCCTATTCTCTCTCC

AAGGACATGGGTACAGTAAACAGAGAGAGGTGCCCAGTGGTCAGTATGCTTGTCTTTGGG

GAAAATGGGACCAAGAGGTCCAGGATAACCTTGGACAGACAAGGTTTGCAGAGAGAGAAG

TTGGCAAGTGCAGGCTCCTGGGCGTGTTCACATCTGCATCCAGCCTGGAGGGGACTCAGG

CAGAGAGCCCTAAGCTGGAGTGTCCAGGCTCTGAGGATCACTGAGGATTCAGTGCTCACG

AAGAATGCCTCTTATTCCCCAGGGTGGAGCAGGAGCCCACACCCCTTGGACAATTAAGGA

GAGAAGGGAGGGAGGGGGATAGGTTTTAGCCCCTGAAGGCATTCTCATTAAAGGTACTTC

TCCCAGCCTCCCCAGAACTTGGTTAGGGTACTAGAGTGGGTTGCGACTTGTAGGAAGAAT

GAGATGAGGTTGTGTGGGTGCATGACAGGGATTGAGTGTAGGTTATCAGACAGCCAAGGA

AGCAGTAACCAAGTGAAAAATCTCTTCTTCCTGCTGCCTCCCTGTGGCTGGTGTAATATT

ATGGCATCTATGATCCATTGTTTTTCTCTCAGGATACTCTCAGGATATTTCTTTTTATAT

ATATATATACTTTAAGTTCTAGGGTACATGTGCACAACGTGCAGGTTTGTTACATATGTA

TACATGTGCCATGTTGGTGTGCTGCACCCATTAACTCGTCATTTACATTAGGTATATTTC

CTAATGCTATCCCTCCCCCCTCCCCCCACCCCACAACAGGCCCCGGTGTATGATGTTCCC

CTTCCTGTGTCCATGTGTTCTCATTGTTCAGTTCCCACCTATGAGTGAGAACATGTGGTC

TTTGGTTTTTTGTCCTTGCAATAGTTTGCTGTGAATGATGGTTTCCAGCTTCCTCCATGT

CCCTACAAAGGACATGAACTCATCCTTTTTTATGGCTGCACAGTATTCCATGGTGTATAT

GTGTGCATTTTCTTAATCCAGTCTATCACTGATGGACAGTTGGGTTGGTTCCAAGTCTTT

GCTATTGTGAATAGTGCCGCTATAAACATATGTGTGCATGTGTCTTTATAGCAGCATGAT

TTATAATCCTTTGGGTATATACCCAGTAATGGGATGGCTGGGTCAAATGGTATTTCTAGT

TCTAGATCCTTGAGGAATTGCCACACTGTCTTGAGATACCATCTCACACCAGTTAAAATG

GCGATCATTAAAAAGTCAGGAAACAACAGGTGCTGGAGAGGATGTGGAGAAATAGGAACA

CTTTTACTCTGTTGGTGGGACTGTAAACTAGTTCAACCATTGTACTCTCAGGACATTTCT

AGTCCAAATTTACACCAACACTCTGAGAGGAAGGACTGCAAAGTAGGTACCTTAGTTTTC

CACTGACTTCCACTTTTCCTGCTTACACCCTTCCTCCTAGACCTCTCCACACCCCTCCTA

GGACACACCTAAAAGGTACTGACATCATGTCACCTCCTCATCTTTCAGGGTAGCAAGGTT

GGAATCTCCTGAATACAGCCCCTCAAGCCCTAAAACCTCTTATCTATTACCTTGGGTTCA

TTGTCCAGGAAGGGGAGGAGAACTTGAACTTGTAGTCACAGAAGGGTGCTGAGAACTAAC

CAGCAGGACGGCTCAGCCCTGGGAACTGCAGAGGGGTGAGGCTGGGGAGAGAGGAGGCTG

GAGCAGCACTGGTGACACTGAACAGTGTCAGGAGGAAGTGACGGATGCAGCGCCCCCATC

CCATAGGCAGAGCTGTCATGTGGGATGAGGGACAGTGTTGGGAGCCACCAAGGAAACCCA

GAGGTGGGGGAGCAGAGAGCAGAAGGGGGCATGTGATGCTGGGCAGTGAAAGGGAGGACA

GGCAAAGGCTGGGTTGAGGTTTGTAGGGGGAATGAGATGAGGCAGTGGAGCCATGTGACA

GGGACTGAGGGTAGATTACTGGAGCTCCCTGCGTAGAATGAATGTTCAATCAAAACCTGC

TGGAGGGAGAGCTGGAGCCATAGGGGAGTGGGTAAAGTGGGCAGGGTTGATTCCACAATT

CCCTGCATGCTCCCCCAACTCCACACACATCCCCAACCTCAAACAGGGCACAAGACCAAA

GGGCTGAGGAGCCAGGCTATAGCTTAAAGAGGCTGGGGGAGAAAAGCTTGGCTGAGACAA

CCCATAGGGAGCTAGAGGTTTTTAATATATCCTATTCTGAATAAGAGACGAATTCATTCA

GATCAGTGGTTTCAAACCGTGCTCTGGGCAACTCAATTGCTAAGGGTTCCACAAACAGGA

TAAAGTTTCTTATATACAAAAAAAATGAAGGTTTCAAATTACACCATAAAACCCCTCATT

GCTTATGTCTACTTGGCAGGTAAAATTCCATTTCAAAAGTTAAATGTACTTAAAAAATTA

CCTAAGACTGGGTAAATTAAAAAAATTAAATGTTGCAAAGAAAAAATTCAAAATTCTTAT

TCTTGAATGAAAAACGTTCTCTTACTGGTGATTGAGGAGGAGAAACAAAGACTAACAAAT

GAAAATGGGAGAATCCACACTCAGAGTGGGGCAATTGAACAGGCAGGGGCGGATGGATGG

CAGAGGAGGAGGAATCTGGACTCAAGGAGCTGGGGGGCTCTGGGCCTGGAATTTCAGGGT

CTGGGGCCCAAGGCACCAGGAGAAGAGGCAGGTCAGGATATCTGAGTCAAGACCTGGGAT

CTTGCCTTAGCAATGACACTGGAGACTAAAGGTGGACTCCATGGTGCCCTTGAGCCCAGC

CCTACCCCATCTCCACTATCCTCTGCCACCAGCTGTGCAACTTCTGCTAGGGGTGAGGTT

AATAAACTGGAGAAGTTAATTTGTGGAGCATGAAACAGATGAGCAGAACAATCACAGCAC

CTTAATTTCCCCAGTGTGCCCAAGAACAGAGCAGGCCTGAAGACACTCAAACAGAAACAA

ACATGTGCCGTGTCACTGATAATTCTGTGTAGACACACACCTGCCAGACACTGCTCATGG

CACTCCCTAGGAAGAACAGCATGTGGGAAAGGCTGCCAAAATTGTTCATGTAAAAATTAC

ATCAATGCTGTCTTCCTCGGTGCTGCCTATGCAGCTGGCAGCCATCTCTTCCTCCACATC

ATGGCCTCCCTCAGACTCCTCATGAAGGATAAGATCCTCAAAAAGAGGACCAACAAGTTC

ATGAGGCACCAATCAGACTGAAATGTCAAAATTAAGCATAACTGGCGGAAACCCAGAGGT

CTTAACAGTAGGGTTCGTAGAAGGTCCAAGGGCCAGATCTTGATGCCCAACATTGCTTAT

GGGAGCAACAACAACAACAAAAAACACATGCTGCCCAGTGGCTTCCAGAAGTTTCTGGCC

CACAGCCTCAAGGAGCTGAAAGTGCTGCTGATGTGCAACAAATCTTACTGTGCTGAGATC

GCTCACAAAATTTCCTCCAGAACTGCAAAGTCATCATGGAAAGAGTCACCCAGCCGGCCA

TCAGAGTCACCAACCCCAGTACCAGGGTGCACAGCTAAGAAAATGAGTAGAAAGTTCATG

TCCACGTTTTGTGTGTAAATAAAACCATAAAAACTGCCAAAAAAAATTACATGAATGCCT

CTAAACCCAAAGGACTCTACCCCCACAGGTCCCTGGTTGTTGTGGTGATTTTCATTGTGT

AAAATACTTTCCACATCTTTTGACACCAAGTCTTTCTGCAGCCATGTTTGAAAATTAACT

TTCAGGCTACAGAGTCTTTCTTATACCAAAGTTGAAGAAAGTTTTAAGAAATATATTTCT

ACATCTCCTACATGCAAAACAACAGGAGCAAGTTGAGGAATTCTCAAGAAACTGGTCGAG

AAGAGAGAGCGCTTAGCTATGGAAAAGAGAAAGAAGGAAGGGAGGGCTTCCTGGAGGAGG

TGGCATTTGAACCAGGACTGACATCAGGATGGAAATGTCAGTCAGGGAGTTAAGTAGGGG

GAGCAGCTCCGCCCTCCACGTCCCCAGCTCCTCCCGCCCCTGTTTTTTCTCCCAGTGACC

CCACGTGAAACGTCTCCGCCTCCTCCAGCCACCAGCAGAAGGGACTGCCTTCCCCTCAGT

GCTCGCCCCTCCCTAGTGATCACTCAGTGCCCCTGAGCTCATTCTTTTCAGTAAATTCTC

TCTCTGCGTGGTGAGAAAACAGGCCTGGAGAGGCTCTGCGACCCGCTTAGGACCACAGAA

CTCGGTACTAGGAAAACTCCTATTTTAAAATCCAGCCCTGAGTGGGAAGATTTGGGAAGA

ATCGTTAATATTGAGAGAGAGAGGGAGAAAGAGGATTAGATGAGAGTGGCGCCTCCGCTC

ATGTCCGCCCCCTCCCCGCAGAGAATTACGTGTACCAGTTACGGCAGGAATGCTACGCGT

TTAATGGGACACAGCGCTTCCTGGAGAGATACATCTACAACCGGGAGGAGTACGCGCGCT

TCGACAGCGACGTGGGGGAGTTCCGGGCGGTGACGGAGCTGGGGCGGCCTGCTGCGGAGT

ACTGGAACAGCCAGAAGGACATCCTGGAGGAGGAGCGGGCAGTGCCGGACAGGATATGCA

GACACAACTACGAGCTGGACGAGGCCGTGACCCTGCAGCGCCGAGGTGAGTGAGGGCTTT

GGGCCGGCGGTCCCAGGGCAGCCCCGCGGGCCCGTGCCCAGGGCGCAGGAGCAGCCGGGT

TGGCCTAAGGGACCTTAGTGCCGGGCGGAAAGGGGACTTTGGGTTGGGGATTCATGGGGG

GAGCCCATCTGGAGCTTGTCAGGGGAGCGAGCGCGGGGACCTGGACTGGGCTGAGCATGG

AGTGAGGAGGACGGGAGCAGAGAGACCCCGGGGACTTCATCAGGCCTGGCAGCTGACTGC

ATGTGGGGTGAAAAAAAGGAAGCCACAGGACAGCGCACAAGGGTATGGTGTGGAGATGGA

GGTGGAGATGGCACAGCAGGCCACACAGAGAAGAAACCTACAGGGAGTTAGCTGGGTTTG

AGGTGCTTGAGGGGCAGATGGGTGGTCTGATGGGCAGGTAGACAGAAGGGTCTGCAGCCG

GGGAGGAGACTGAGATACATGAGACCATCCAGGGAGAGGGGACCCAGGGGGAAGAGCAAA

GGACTGGATCCTGGGAACTGGACAGTTGTGATTTGGCCAAGACAGAAAAGCCTGTGAAAG

AGACCAATAAAACCCAAGTGCAGTGTGAGGAGAGGCCCGCAGAGAAGAGTCTTGGAAGCT

GAGGGGAGGTGACCTCAGCAGCACAGTGGACAGCGGTGCCAGTGACTTGGGAAGGTCAGA

AAACAGAAGATGGAAAGTGGGTTTGGAAACCAGGGAGACCTGGGGAGAGCAGGTTGGCCG

CAGCGGCAGGAGCTGGAATGGGAGGGGGTGCATGAGGCTGAGTGTGGCGCATCCTCCTCG

GGGCTGAGATGGATTTTACTTGTCTTGGGTTCCCCACGGCTGTCACAGGGCAGTGTCTGA

GTTCATTCGTCTTTTTCCTTCAGGAAGTCTGGGTGTAAAGGGATGGAGAGAGGTGAGGTG

TGTGCAGTAAGAGGATTTCTCAAGGATGGGACAGGAAGGCCTTGGAGCTTTGGCTTCCTC

CTGTGAACTTGTGGGGTGGGGAGCCTGGTGCACCAACCTTTGGGACTTGAGGGAGTAGTA

TCAGGATGTGGGATTGAGCCCTGGACCTTTTTTCTAGAAAGAGGAAAAAAATGAAGGGAG

GAGGAGGAGGAAGCTGGGGAGATCACACCTTTGATTTTCTTGTTCCTGGAAAGTGAAAGG

AAGTTCACCTGCTATGAGTGAGAAGGTGGACACACTGGGTGGGGATGAGGTGAGTGACAT

GAGCTTAGGAAAGTTGCTGAGGTAATTGGTTGAGAGAGGTGTTCAAATAAAAGTAATGCA

ATTGGCAAAAACTGTTACTAAGACTTTGTAGAGGCACAAATCAGTGACATGGCAGCATCT

TCTTTCACAGTAATCAACTGCCAGATTGCAGGGAGCCCTGATACCAGCCTAAGGAGTGTG

AGTTTCTCCTCTAGGCCCACAGGTCCCCAACCTCACTCCTCCGAAGACTCTCCTGGAGAT

CATCTGTGATGCGCAGATACCCAGACTCACTGCCCCGAGACTCAGATTCCCTGGGTGGGG

AGGTCTGGGGATCTCTGCTTGTAATCAGCTCCCTAGAGGTTCCCATGTAGCCGGATAAGT

ATTGTCAGAACACTGAAGATTTTTGAAAAATGAAAAAGAGAAGGTTGGAGATGTGTCTTC

AGAAGACTACTAAGGGTGCTGGGTAGAGGAGGGACCAGAGGCAGGGAGATGAGGTAGGAA

ACTGCTATTATTTGTCAGGGAAATTGCAATCAAGAAATGAGTTAGAACAGGGAAAACACA

GAGGCAGGGGAGAGGTGGAAGGGGGAGGAAAGGAGTAGTGACAATTCCAGGGTGTATGCT

CACCCAAATCTAGAAGTAATTGAGCAAATGTTTTCTGGGCATTAGAGAAGGCAACTAGAA

CAAACAGGAATCCTTGCCTTGGTGAAATGTATTTGAACTGGGTCAGAAATGAGGCCATTG

GGTATCAAGCCTTAACTCCAGCGCCCCCTGGAGGTCGCTGATGTGGCTCCAGGCTGACCT

GCTCCTGTCAAAGAATATTGAGCAAGATGCCTCTCATGGAATGTTCTGGGACCTTAAAAC

AGATACCCAAGTATTCCCCCTGATTTCATGGTTCCCAGAAGCTCTATGGGGAAGAAATTG

TACGTAATTCACAACTGAGATTTAGACATAAGTTGAATAGTGTAATGGACGTTGAGTTAA

CCGAGGTAATGAAGTAGTGAGACACAGGTGCCCCTGAAATAAACTCACATTGAGGGAAGA

GGCTGACAATGTGGATCAGTCTGTAAACAAGGCAAAAATACAATAGAGAGTAAGGGTTGT

GTGTCAGTTCAAGACTGTACTTTTACCTGGCCCAGCGCCATGTTGGGGTATTTGTGTTCT

CCAGGAAGTAGAAAGGAAAGAACTGAGTGATTAGGGACCTAGAAGACTAATTTGAGACAT

TCCTCTTGATGAACTGTTCTCTAGGGTAGTCCTCTGAAAGAGCTGTTCTCTAGTGGATCT

CCCTGAATGAACTGTTCTCTAGGAGCACTTGACCCTTTTCTGTGTTTGTTTTTTGTTTTG

TGTTTGTGTTTGTTTTTGAGACAGGTTCTCGCTCTGTCTCCCAGGCTGGAGTGCTGTGGC

GCCATCATGACTCACTGCAGCCTCAACCTCCTGGGCTCAAGTGATCCTCCTGCCTCAGCC

TCCCATGTAGCTAGAACTACAGATACATGTACCACCATGTCTGGCTAATTTATTTTTCTT

TTTAGAGATGGGTTCTCACTATGTTGCCCAGGCTGGTCTCGAAACCCTGGGCTCAAGTGA

TCCTCACGCCTCAGCCTCCCAAAGTGCTAAGATTATAGGCATGACCACCATGCCTGGCCT

TTTCTGCCTTCCGAGAAGGAAAAAGGTACTGGTGGCAGAGATCCAAAAGAAAAGTTGCCA

GTGGCAGTGTGGAAATTGACCTGAGAACAACAGAACAAGCTGGGGCACAAATGCAAAGAT

GCAGAGGGAGGCAACATCTGGTTATCTGTGAGACCTTCATGGGACCTGAAGACACATCCT

TTTGGAAAAGGATGGGATTTCTACTACTCAAGCATGTAGGGGCTCAGGATATTATGTAAA

TATGAAGATTTTGAGTTTTTGTAAGTGAGGTAAAAAAAATACCCAGGTTATTTACAGAAT

AAGACATGTCAAGCTCTCTTCATTTTCTTTGTATTTTCATGAAATAAAGGTATTAGATTA

ACAGGCCACCATAATGCCATTGTCTGTATATCTTAATTTCAAGATATTATTTGAGTAATT

TTTGCATCCTTTGTATCAAGATAGAACTTTGAAAAGACAGGTAATTTCACAGTTGATTAA

ATATTCTTTGCCCAAATTACTTTTGGTTAAAATTTCTCCTATATGTGCTACAGAGTGCAA

ACTCTGTCTCCCTGCCATTCCGCTATATACTTACTAATTATTATTTTATTCAAGATCATG

CATGCTCTACTTGAAGGTCTATCTTTTCAGTGCTACCCTTACCCACTAGCCTAATCACAT

TATATCTATTTTCAACATCTAGGAATCAATTACGTAGTGAACATGCCTAAGAAATAATAA

TCTGGGCAGATGCAGTGGCTCAGGCCTGTAATCCCAGCACTTTGAGAGGCTGAGCGGGTG

GATCACTTGAGGTCAGGAGTTGGTCAAGTGCTCCTAGAGAACCAGCCTGACCAACATGGA

GAAACCTTGTCTCTACTGATAATACAAAAATTAGCCAGGTGAGGTGGCAGGCACCTATAA

TCCCAGCTATTCGGGAGGCTGAGGAAGGAGAATTGCTTGAACCCCTGAGGTGGAGGTTGC

AGTGAGCCAATATTGCGCCACTGCATTCCAGACTTGGCAACAGAGCGACACTCCACCTCA

ACAAAAAGAAAGAAAGAGCGAGATTACGTCTCAAAAAAGGAAGGAAGGAAGGACAATCTC

AAATGCTATTTCATTATTTTCCTTCCCCACTCGTAGTCCAGCCTAAGGTGAACGTTTCCC

CCTCCAAGAAGGGGCCCCTGCAGCACCACAACCTGCTTGTCTGCCACGTGACAGATTTCT

ACCCAGGCAGCATTCAAGTCCGATGGTTCCTGAATGGACAGGAGGAAACAGCTGGGGTCG

TGTCCACCAACCTGATCCGTAATGGAGACTGGACCTTCCAGATCCTGGTGATGCTGGAAA

TGACCCCCCAGCAGGGAGACGTCTACATCTGCCAAGTGGAGCACACCAGCCTGGACAGTC

CTGTCACCGTGGAGTGGAGTGAGTCTCTGATGACCGTCTAGACCCCACCTCTGAAGAGCA

GGGGACTCTCTGGCTCTGGGGTCCACTCATCTTATCTTCTGCATCTATACCCTGGGGCCA

TGTCCAAACCCCATCTTTCTTCTATACCAGCTCCTGAGCATAGTTTGAAGCCAGGGCAAT

GGAGACTTCCTGACCTTGGCTTAGGGGTTCCTGAAGATTCATAGTTCTCCCCCTTGTCAG

AGAATCTAGGGACACTGGCTGATCTCGAAACCCTCACACACAGGAACTGACCTCACACAT

AGGAACAGTTCTCTTCTTTCAGCATTTTAGCCTCTTCTCAGGCATTTTGAGAGGCAACTT

CTAGAATCAGCATTTGCCACCTTGTTGAGGTCACACCCCTGTTCCGGACATGAGGGTGGC

TCTTTCTGAATTTCCTCTTAGCAAGCTTTTTCCCCTGCACTGTCTTCATCCCGATATTCT

GCATCACGCTCCAGAATCTCAGACAGGACATGAGTAGGGATGCAGCTGGTGGAGGTGACA

CTAAACCTGGGTCTGTCCTTCCCAGAGGCACAGTCTGATTCTGCCCGGAGTAAGACATTG

ACGGGAGCTGGGGGCTTCGTGCTGGGGCTCATCATCTGTGGAGTGGGCATCTTCATGCAC

AGGAGGAGCAAGAAAGGTGAGAAAGCCTGCAAGGTGAGCGGGACCTACCTTCCCCTGGCA

TATTCACACTTACTCCATGATGAGGGTTCAGACAGAAAAGAAATGTCAGAAAGCTCTAGA

GACCACTGAAATCAGATAGTCGGGGAACAAACATGACCTATGGCGAGAGGGGGATCCCAG

GCTGGGATCTTAATGCAGCCAGATGCATGAGGTCCCAGGTGCTCAGACTCCTGCGGGGCA

TCCATTGAGTGGTGGTCAATGGAATTTGGTGGGATGGAAATGTTTCTCTAACTATCTGAG

GTGGTTTCAATGGCTGAATACATAACCTTTCCTCTTTCATTTCAGTTCAACGAGGATCTG

CATAACAGGTAATATTCCTGCTTTGATTTCCTTGGGGGGTGGGTTACAGGAGGATATGAC

TCCTTTCTGTGCATTGTAATACTGAGGCTCCTCCAGGAAGGGAATCTCAGGCATGAACCC

CTCTTTCAACCTCAGCTCTCGGGTGAGTAGGGAAAAAGCATTGCACGGCTCCATTGCTGA

AGGAAGCAGAGATCAGTTCTGTTCTTTATCAGCCTAAGATGCAGCCTCTCACCATAATTT

TTCTCTCCTGGACTTAAAGGAAGGAGGCCAGCAACCTGGGATAACTTGTCCTTTACCCCC

ACAGGGTTCCTGACCTCACCGAAAAGACTAATGTGCCTTAGAACAAGCATTTGCTGTGTT

TTGTTAGCACCTGGTTCCAGGACAGACCCTCAGCTTCCCAAGAGGATACTGCTGCCAAGA

ACTTGCTCTGAAGTCAGTTTCTATCGTTCTGCTCTTTGATTCAAAGCACTGTTTCTCTCA

CTGGGCCTCCAACCATGTTCCCTTCTTCTTAGCACCACAAATAATCAAAACCCAACATAA

GTGTTTGTTTTCCTTTAAAAATATGCATCAAATCGTCTCTCATTACTTTGAGGGTTTTAG

TAAACAGTAGGAGTTAATAAAGAAGTTCATTTTGGTTTACACATAGGAAAGAAGAGAAGC

ATGAAAGTGGAGATATGTTAACTATTGTATAATGTGGCCTGTTATACATGACACTCTTCT

GAATTGACTGTATTTCAGTGAGCTGCCCCCAAATCAAGTTTAGTGCCCTCATCCATTTAT

GTCTCAGACCGCTATTCTTAACTATTCAATGGTGAGCAGACTGCAAATCTGCCTGATAGG

ACCCGTATTCCCACAGCACTAATTCAACATATATCTTACTGAGAGCATGTTTTATCATTA

CCATTAAGAAGTTAAATGAACATCAGAATTTAAAATCATAAATATAATCTAATACACTTT

AA

>HLA:HLA06601 DQA1*01:03:01:02

AAAAATTCCTGGAGGTTGTAAGCCAGAACATCCTGAAGGATGCCGTATAACTGATGACCT

CATCTATCCACGAGGCTGCTCAGAAATGCCCACCCCTGGCCAGGCGCGGTGGCTCATGCC

TGTAATCTGAGCACTTTGGGAGGCTGAGACGGGCAGATCATGAGGTTAGGAGTTCAAGAC

CAGCGTGGCCAACATAGTGAAACCCCGTCTCTACTAAAAATACAAAAATTAGCTGGGCGT

GGTGGCAGGTGCCTGTAGTCCCAGCTACTTGGGAGGGTGAGGGAGGAGAATCGCTTGAAT

CCGGGAGGCAAAGGTTGCAGTGAGCCGAGACCTTGCCATTGTACCTCAGCCTGGGTGACA

GAGTGAGACTATGTCTCAAAAAAAAAGAAAAAGAAAAGAAAAAGAAAAAAAAAGAAAAAC

ACCCATCCCTCTTGCGACTGGCAGACATGCACACACCAGAGAAGATTCCAATTTAGTGTC

CTCCCTTTATTCATAGAACAATTCCTCAAGTCCACTCTGAGTAGAGGCTGCATCACAAGG

GGATTGCCCCGTCTCCTTCCAGGGCTCTTAATACAAACTCTTCAACTAGTAACTGAGATG

TCACCATAGGGGATTTTTCTAATTGGCCAAAACCTGACTTGGCAGGGTTTGGTTTGGGTG

TCTTCAGATTTCCTTGTCTTGAGGTCCTCACAATTACTCTACAGCTCAGAACACCAACTG

CTGAGGCTGCCTTGGGAAGAGGATGATCCTAAACAAAGCTCTGCTGCTGGGGGCCCTCGC

TCTGACCACCGTGATGAGCCCCTGTGGAGGTGAAGACATTGTGGGTGAGTGCATGAGTGA

GGGATGTTCTCTGGAGCTGAAAAACAGTAAATTGAAGGAAAAGAGATAAAGCGATTTGCA

GAGAAACTGTAGAGATTTCCTAAGGGCCCTTTCAGTATTAAGACAATTAAAAATTATAGC

TGTTCCTCCTTCAGGAAACCAGAGCCCCAACCTACTCTTTTTGTTATCTATGCTGTTGTG

TTCACTAAGGACGCTATTCTGTTTATATTATATTCAGTGACTACAGCCTGGAGGTCTCTA

TGTCGTTCCATCATGATTGCCTCAAAAATTAGTGAGGTTTCCATCAGTGGATAATTTTTT

ATTATTAAAAATTTATGAAGTGTCATTCTCAAATTTCCCTGAACAACTTTTGAAGCTTTT

CGGATGTCTCCGGTGGTAGATCTTGGGGTCATTCCATCAATTATGTACTCTATAGATATT

TAAAATGTTGCCCGTTTCTTTCTCTCAGACTTACTCACATTTCCACATGGGAACTGGCAC

AGGTGGGGAGTGGGTAAAGGAGTCCAGCAGGCTGAATGCCTTCAACAGTCATTTTACCAC

ATGGTCCTCACTTACTCTCAGCTGCCTCATATGTGTCACCTCACAAGTAATCAAATAAAA

TGGGCATGTGGCTAAGTTTTGTAAATAGTGAAAACATGAATGTCAATTTTTTTTACATAT

TTCTATTACAGATATAGCTTCACATTTCTTTTCTTTAGCAAAATAAGGGATCATTTTATT

TTAAAATTGAGAAGTAGAAAAAATTGGTAAAATAAATCATTTTATTCTCAAATTATCAAC

CCAAATTACCTGTTCTTCACCTCATCTAATAAAGTCCTATAAAAAGAAAAGTGGGCCAGA

CAAGGTGGCTCATGCCTGTAATCCCAGCACTTTGGGAGGCCGAGGCAGGAGGATCATTTG

AGCCTGGGAGCTTGAGACCAGCCTGGGCAACATAGCAAGACCTCATCTCTACAAAAAAAG

AAAAATAAAAATTAGCCAGGCATGGTGGTGTGTGCCTGTGGTGCCAGCTACTCAGAAGGC

TGCCGTGGGAGGACCACTTGAGTCCATGAGGCGGAAGCTGCAGTGAGCCATGATGGCACC

ACTACACTCCAGCCAGGGCAACAGAGAGAGACTCTGTCTCAAAAAGAAAGAGGAAAGAAA

GCAAGGAACCAAGGAAGGAAGGAAGGAAGGAGAAGGAAGGAAGGAAAAGAAAAGAAAAGG

GAAGGGAGGAAGGAAGGAAAAAAGAAAGACGGAAAGAAAAAGGAAGGAAGGAAGAAAGGA

AGCACAGATTAATTATTTGGTCTCTTACTCTCCTCTGCCTTTGTCGTCCATCTCTTTCCA

CCTCTCTTGATGCATTCCTTTCTCCCCCTTCCCTTTCAGGATCCACCTCTGGCTCCCTGC

TCCTTTATAGATATGGACAGTGGGTTTGTAAAGCAAAAGTTGAAAAGTCAGATAGTTAAA

AGGGGAAGTGAACTGGAAGGTACTCTAAACTTTCACAACTTTATTAACCGTGGCTGCTCC

CATTATGATTTTGTTCGGCAGTGGAAGTTTCACCTGCTTCTCCAGAGCACTTGGCATCTT

TGTTCCAAATTTCCTTTCTTCAGCCTCACACCAGAGTGCCCTGGTCAGGCTCGGCTCATC

CATTAGGCACTATGTGGGCAGTGCAGGGGACCCTCCATACTGTAAAGCCACATGAGAAGG

TTTTAACACCTTTTAAAATTAGAAAAATAATGGAATATTAGAGCCTAAGAAAATGTTTTA

ACTTTTAATTCAGCCTAGATTATATTGTCTTTATACCAATTCAGTCATAAAATATAATTT

TCCATATTTTTATGGAGGAAGGGGCCCATACAAGCAAGAATGCTCGGGGCTCACATGTCA

GAATGCAGCCTTGGTCATGACTGATCCTGGCGTTTGTATGGTTCTGCTACCTGTGTGCCT

GTCCGTCTTCCCCAAAATCTGTGTGGTCCTCGAATATAGCAACTGTCATTCAATACACAT

GTTTGAGCACACAGTGAGCTAAGTTTTAAGGATTCAAAGATGAAAAGTCATGCTGTCTTC

CCTGCAGAGGGTGCTCAGATTAGTGATGGAAACAGTGTGGGGTGCAGGAAAGCAGAAGGC

CATTGCTGAGCAGGGCAGTGGACCCAGCAGAGGCTGAAACAATACAAGTGACTTGGTTCC

AGGTGGGCCAGCAGGGTGACGTCCTCTAGCAAAATTTGGCACCCAAGACAAGTACCAGAA

GAAAAGAAAGACTGCATGTATTCCACATATATTCATGTTTGAACAAGGAGTCAAGATTTA

TGGCAAGGATAAGGAGGCTTTGTTGGTGACCTGTTAAGACCAACCAGGGCAGTCATGCTG

GATAGGGAAGAAGGTGAGCTGGAAGAGGAACAGACAAACTTGGACAGCCAGATGTTGAGA

TGGAGGGGTTGGAGGTCATAATGTGGTCAAAACATGTTGATGAGAGGACTCAGCTACAAA

GTTGTTAACTTAAGCAGAAACCTCAAGGATGGATTTTAGAATTTCTCCAGGAAGTCCTAA

AAGATAATTCCTTTTCAGGGAGAAAAACAACAGACCACTGCAAAGACCAGGGAACATGAA

AGGATAATGTAGTTTGGCTTGCTTGGCAGATACTTTTGAAGGATGTTGGACTGTAAGGCT

GTCGATATCCTCCTCACAGAACTTACTACAGTACATTGTATCTGTTCCCTTACCTACCTG

ACTCTTCCACTATTCAGTTTGTTCCTTAATGGTAGACCATGCCTGATTGGTGTTTTACAC

ATCTCCGGCTATGTCTGACACTTGTGGATGCTCAGAAAGTGGGGAAGGAAGGAAAGATAC

GATGGTAAAAGGCTTACACATGTCTTGACAAGAAAGTCCAGTTCGGCTCATTTGGCTGGA

GTCGTACTGCATGGCTGCCATTCTGCTCTGGCATCCTCAGACAAGCACACTGCCCATTAG

AGGAAAAAGTGTGAATATAAGTGTTGAGTCAGAATGCTGTAGACATTTAGTAACCTCCTT

CACAGGAAAAAAAAAAGATGGGGGGAATGACAGAAATCCAAAAACTAGTAGAGCTTCCAC

TTTTCATTTCAGAAGAAATCAGTTGCTCTCCTATAAGGACCATTACTATTAACAAAACAG

AGACCTTAGAAGGAGGCATTGTTTATTTATTATATATTTTGTAATGTTATTACCAATCTT

GTTATACTCTTTCTTATACCCTACAATTGTTAGCAGAAATTATTTTAAATTAATAAGATC

CTGCATGCTTTTCCTTAAAAAAAAAAAAAAGAAAGAAAGAAAGATCTCTGTGTAGAGTGT

CCTATTCTGAGCCAGTCCTGAGAGGAAAGGAAGTATAATCAATTTGTTATTAACCGATGA

AAGAATTAAGTGAAAGATAAATCTCAGGAAGCAGAGGGAAGTAAACCTAATCTCTGACTA

AGAAAGCTAAATACTATGATAACTCATTCATTCCTTCTTTTGTTCAATTACATTATTTAA

TCATAAGTCCATGACGTGCCAGGCACTCAGGAAATAGTAAAAATTGGACGTGCGATATTC

TGCCCTTGTGTAGCGCACACTAGAGTGGGAAAGAAAGTGCACTTTTAACTGGACAACTAC

CAACATGATGAGGGGAGGAAACAGGGGCTGGAAATGTCCACGGACTGTGCCGAAAAATGA

AGCCCATAATATTTGAAAGTCAGTTTCTTCCATCATTTTGTGTATTAAGGTTCTTTCTTC

CCCTGTTCTCCGCCTTCCTGCTTGTCATCTTCACTCATCAGCTGACCATGTTGCCTCTTG

TGGTGTAAACTTGTACCAGTTTTACGGTCCCTCTGGCCAGTTCACCCATGAATTTGATGG

AGATGAGCAGTTCTACGTGGACCTGGAGAAGAAGGAGACTGCCTGGCGGTGGCCTGAGTT

CAGCAAATTTGGAGGTTTTGACCCGCAGGGTGCACTGAGAAACATGGCTGTGGCAAAACA

CAACTTGAACATCATGATTAAACGCTACAACTCTACCGCTGCTACCAATGGTATGCGTCC

ACCATTCTGCCTCTCTTTACTTAAGCTATCCCTTCATACCAGGGTTCATTATTTTCTTCC

CAAGAGGTCCCCAGATCTTCTTATGGCAATTGCTGAAATTTTATCATCTCCCATCTCTAA

AATCACATATTCCCATGTAATACAAGGGTCTTTCCATTATGCATTCAGCAAATCCTTCTA

GGAGAGGTCTCATCAACCTCCTACTTTATTAAACATGCCCACAGAGAGAAGGGCACAGGA

ATAAAGCAGAGGCAATGTGTCATTGCTCCCAAGCAGAAGGTAAATAAGACCTCTTTGACT

ATCAGGTGGTGAAATGCTGGTAGGAGGGCTCTTCCAGGATGTAATGCAGAACTTCAGGGC

AGAGCTATTCACACTTCACACCAGTGCTGTTTCCTCACCACAGAGGTTCCTGAGGTCACA

GTGTTTTCCAAGTCTCCCGTGACACTGGGTCAGCCCAACACCCTCATCTGTCTTGTGGAC

AACATCTTTCCTCCTGTGGTCAACATCACATGGCTGAGCAATGGGCACGCAGTCACAGAA

GGTGTTTCTGAGACCAGCTTCCTCTCCAAGAGTGATCATTCCTTCTTCAAGATCAGTTAC

CTCACCTTCCTCCCTTCTGCTGATGAGATTTATGACTGCAAGGTGGAGCACTGGGGCCTG

GACCAGCCTCTTCTGAAACACTGGGGTAAGGATGAGTTTCATCATTTTTTGATTCTTTCT

TGTCTGTCAGGTTCAGAACTTCCTGCCTTTTACTCCTATATCCAAAAACTTGTTTTCCAC

ACTTCATGGGTTTCTTTTCTGTCTCACTTTTTTTTTTGAAAGAATAAAGCAAAAAAAGCA

GAGATTTATTGAAAATGAAAGTACACTCTACAGGATGGGAGTGGGCCTGCCACTTCATGG

TTTTCTAATGATAGACTTCACTCTCCTCCCTAAGCTGGGGGCCTTGAGTCTTTGTAGAGC

CAACCCTGTACCCCATCCCATCCCACACACATGCACATGAGCAAACTCTGCATTCTGACC

TCAACAACTTCACTTCCACAGAGCCTGAGATTCCAGCCCCTATGTCAGAGCTCACAGAGA

CTGTGGTCTGTGCCCTGGGGTTGTCTGTGGGCCTCGTGGGCATTGTGGTGGGCACTGTCT

TCATCATCCAAGGCCTGCGTTCAGTTGGTGCTTCCAGACACCAAGGGCCCTTGTGAATCC

CATCCTGGAAGGGAAGGTAAGATTGAGACTGGTTACAGTTGAAGCGGCAGTATGAAAGGA

AGGAAAGTGGGAGGGCGTTGTGGACATGAATGTGGTTTAAAGTTGTAGGGGAATTGGGAA

GTGGCATGATGATGACACAGGACCCCCCTCAGACCCATTGATCTCATGTCTGCCCTGTTG

CAGGTGCATCGCCATCTACAGGAACAGAAGAATGGACTTGCTAAATGACCTAGCACTATT

CTCTGGCCCGATTTATCATATCCCTTTTCTCCTCCAAATGTTTCTCCTCTCACCTTTTCT

CTGGGACTTAAGCTGCTATATCCCCTCAGAGCTCACAAATGCCTTTACATTCTTTCCCTG

ACCTCCTGATTTTTTTTTTCTTTTCTCAAATGTTACCTACAAAGACATGCCTGGGGTAAG

CCACCCGGCTCC

>HLA:HLA00607 DQA1*02:01:01:01

AAAAATTCCTGGAGGTTGTAACCCAGAAAATCCTGAAGGATGCCATATAATTGATGACCT

CATCTATCCATGAGGCTGCTCAGAAATGCCCACCCCTGGCCAGGCGCGGTGGCTCATGCC

TGTAATCCAAGCACTTTGGGAGGCTGAGGCAGGCAGATCACGAGGTCAGGAGTTCAAGAC

CAGCGTGGCCAACATAGTGAAACTCTGTCTCTACTAAAAATACAGAAATTAGCCGGGCAT

GGTGGCAGGCACCTGCAGTCCCAGCTACTTGGGAGGGTGAGGCAGGAGAATCGCTTGAAC

CCGGGAGGCAAAGGTTGCAGTGAGCCGAGACCATGCCATTGTACCTCAGCCTGGGTGACA

GAGTGAGACTACGTCTCGAAAAATAAAGAAAAAGAAAAGAAAAAGAAAAAAAAAGAAAAA

TTCCCATCCCTTTTGCGAATGGCAGACATGCACACACCAGAGAAGATTCCAATTTAGTGT

CTTCCCTCTCTTCATAGAACAATTCCTCAAGTCCACTCTGAGTAGAGGCTGCATCACAAC

AAGGGGATTGCCCTGTCTCCTTCCAGGGCTCTTAATAGAAACTCTTCAACTAGTAACTGA

GATGTCACCATGGGGGATTTTTCTAATTGGCCAAAACCTGACTTGGCAGGGTTTGGTTTG

GGTGTCTTCAGATTTCCTTGTCTTGAGGTCCTCACAATTACTCTACAGCTCAGAACAGCA

ACTGCTGAGGCTGCCTTGGGAAGAAGATGATCCTAAACAAAGCTCTGATGCTGGGGGCCC

TCGCCCTGACCACCGTGATGAGCCCTTGTGGAGGTGAAGACATTGTGGGTGAGTGCGTGA

GTGAGGAATGTTCTCTGGAGCTGAAAAACAGTAAATTGAAGGAAAAGAGAGAAAGCGATT

TGCAGAGAAATTGTAGAGATTTCCTAAGACCCCTTTCAGTATTAAGAGAATTAAAAATTA

TAGCTGTTCCTCCTTCAGGAAACCAGAGCCCCAACCTACTCTTTTTGTTATGTATGCTTT

TGTGTTCACTAAGGATGCTATTCTGTTTATATTATATTCAGTGACTACAGCCTGGAGGTC

TCTATGTCATTCCATCATGATTGCCTCAAAAATTAGTGAGGTTTCCATCAGTGGATAATT

TTTTATTATTAAAAATGTATGAAGTGTCATTCTCAAATTTCCCTGAACAACTTTTGAAGA

TTTTCGGATGTCTCCTGTAGTAGATCTTGGGGTCGTTCCATCAATTATATACTCTATAGA

TATTAAAAAAGTTGCCCGTTTCTTTCTCTCAGACTTACTCACATTTCCACATGGGAACTG

GCACAGGTGGGGAGTAGGTAAAGGAGTCCAGCAGGCTGAATGCCTTCAACAATCATTTTA

CCACATGGTCCTCACTTACTCTCAGCTGCCTCATATGTGTCACCTCACAAATAATCAAAT

AAAATGGGCATGTAGCTAAGCTTTGTAAATAGTGAAAACATGGATGTCAATTGTTTTTAC

ATATTTCTATTACAGGTATAGCTTCACATTTCTTTTCTTTAGCAAAATAAGGGATCCTTT

TAGTTTAAAATTGAGAAGTAGAAAAAATTGGTAAATTAAATCATTTTATTCTCAAATTAT

CAACCCAAATTACCTGTTCTTCACCTCATCTAATAAAGTCCTATAAAAAGAAAAGTGGGC

CAGACATGGTGGCTCATGCCTGTAATCCCAGCACTTTGGGAGGCCGAAGCAGGAGGATCA

TTTGAGCCTGGGAGTTTGAGACCAGCCTGGGCAACACAGCAAGACCTCATCTCTACCAAA

AAATAAAATAAAAATTAGCCAGGCATGGTAGTGCATGCCTGTGGTGCCAGCTACTCAGAA

GGCTGCAGTGGGAGGAGCACTTGAGTCCAGGAGGTGGAAGCTGCAGTGAGCCATGATGGC

ACCACTACACTCCAGCCAGGGCAACAGAGAGAGACCCTGTCTCAAAAAGAAAGCGGAAAG

AAAGAGAGAAAGGAAGGAAAGAAGGAAAGAAGGAAGCAAGGAAGGAGAAAGGGAAGGGAA

GAAAGAAGAAAGAAAGAAAGAAAACAGAAGGAAGGAAGCACAGATTAATTATTTGGTCTC

TTAGTCTCCTCTGCCTTTGTCGTCCATCTCTTCCCACCTCTCTTCATGCATTCCTTTCTC

CCTCTTCCCTTTCAGGATCCATCTCTGACTCCCTGCTCCTTTATAGAGATGGACATGAGT

TTGTAAAACAAAAGTTGAAAAGTCAGATAGTTAAAAGGGGAAGTAAACTGGAAGGTACTC

TAAACTTTCACAACCTTATTAACCGTGGCAGCTCCCATTCTGATTTTGTTCAGCAGTGGA

AGTTTCACCCTCTCCTCCAGAGCGCTTGGCTTCTTTGTTCCAAATTTCCTTTCTTCAGCC

TCACACCAGAGTGCCCTGGTCAGGCTCAGCTCATCCATTAGGCACAATGTGGGCAGTGCA

GGGGAACCTCCATACTGTAAAGCCACATGAGAATGTTTTAACTCCTTTTAAAATTATAAA

AAAATGAAATTGTAGAGCCTAAGAAAATGTTTTAACTTTTAATTCAGCCTATATTATATT

GTCTTTATACCAATTCAGTCATAAAATATAATTTTCCATATTTTTATGGAGGAAGGCGTC

CACACAAGCAAGAGTGCTTGGGGCTCACATGTCAGAACGCATCCCTGATCATGGCTGATC

CTGACCTTCGTGTGGTTCTGCTAACTATGTGCCTGTCAGTCTTCCCCAAAATCTATGTGG

TCCTCAAATATAACAACTGTCATTCAATACACATGTTTGAGCACCCAGTGAGCTAAGTTT

TAAGGATTCAAAGATGAAAAGTCATGCTGTCTCCCCTGCAGAGGGTGCTCAGACTAGTGA

TGGAAACAGTATGGGATGAAAGAAAGCAGAAGGCCATTGCTGAGCAGGCAGTGGACTCAG

CAGAGGCTGAAACTATACAAGTGACTTGGTTCCAGCTGGGCCAGCAGGATAACCAGACGA

AAAGAAGGATTGCATATATTCCATATATATTTATGTTTGAACAAAGAGTCAAGGTTTATT

GCAAGGATAAGGAGGCTTTGTTGGTGGCCTGTTAAGACCATCCAGCGTGGTCATACTGGA

TAGGGAAGAAGGTGAGCTGGAAGAGGGATAGACAAACTTGGATGGCCAGATGTTGAGATG

GAGGAGCTGGAGGTCATAACGTGGTCAAAAACATGTTGATGAGAGGACTTAGCTACAAAG

TTGTTAACTTAAGCAGAAACCTCAAGGATTGATTTTATGATTTCTCCAGGAAGTCCTAAA

AGATAATTTCATTTCAGGGAGGAAAACAACAGACCACTGCAAAGACCAGGAACATGAAAG

GATAATGTAGTTTGGTTTGCTTGGCAGATACTTGTGAAAGATGTTGGACTGTAAGGCTGT

CAATATCCTCCTCGCAGAACTTACTACAGTACATTGTATCTGCTCCCTTACCTACCTGAC

TCTCCCACTATTCAGTTTGTTCCTTAATGGTAGACCATGCCTGATTGGTGTTTTACACTT

CCCCTGCTATGTCTGATACTTGTGGATGCTCAGAAAGTGGGGAAGGAAGGAAAGATACGA

TGGTAAAAGGCTTACACATGTCTTGACCAGAATGTTCAGTTTGGCTCATTTGGCTGGAGT

CATACTGCATGGCTGCCATTCTGCTCTGGCATCCTCAGAGAAGCACACTGCCCATTAGAG

GAAAAAGGGTGAATATAAATGTTGAGTCAGAACACTGCAGACATTTAGTAACCTCCTTCA

GAGGAAAAAAAGGGTGGGGGGAATGACAGAAATCCAAAAACTAGTAGAGCTTCCACTTTT

TCATTTCAGAAGAAATCAGTTACTCTCCTCTAAGGACCATTACTATTAACAAAACAGAGA

CCTTAGAAGGAAGCATTATTTATTTATCATATATTTTGTAATGTTATTACCGTTCTTGTT

ATACTCTTTCTTATACCCTACCATTGTTAGCAGAAATTATTTTAAATTAATAAGATCCTG

CATGCTTTTCCTTTTTCTAAAAAAAGAAAGATCTCTGTGTAGAATGTCCTGTTCTGAGCC

AGTCCTGAGAGGAAAGGAAGTATAATCAATTTGTTATTAACTGATGAAAGAATTAAGTGA

AAGATAAACCTTAGGAAGCAGAGGGAAGTTAATCTATGACTAAGAAAGTTAAGTACTCTG

ATAACTCATTCATTCCTTCTTCTGTTCATTTACATTATTTAATCACAAGTCCATGATGTG

CCAGGCACTCAGGAAATAGTGAAAATCGGACACGCGATATTCTGCCCTTGTGTAGCACAC

ACTGTAGTGGGAAAGAAAGTGCACTTTTAACTGGACAACTATCAACACGAAGAGGGGAGG

AAGCAGGGGCTGGAAATGTCCACAGACTTTGCCAAAGACAAAGCCCATAATATTTGAAAG

TCAGTTTCTTCCATCATTTTGTGTATTAAGGTTTTTTATTCTCCTGTTCTCTGCCTTCCT

GCTTGTCATCTTCACTCATCAGCTGACCACGTTGCCTCTTACGGTGTAAACTTGTACCAG

TCTTACGGTCCCTCTGGCCAGTTCACCCATGAATTTGATGGAGACGAGGAGTTCTATGTG

GACCTGGAGAGGAAGGAGACTGTCTGGAAGTTGCCTCTGTTCCACAGACTTAGATTTGAC

CCGCAATTTGCACTGACAAACATCGCTGTGCTAAAACATAACTTGAACATCCTGATTAAA

CGCTCCAACTCTACCGCTGCTACCAATGGTATGTGTCCACCATTCTGCCTTTCTTTACTG

ATCTATCCCTTTATACCAAGTTTCATTATTTTCTTTCCAAGAGGTCCCCAGATCTTCTCA

TGGCAATTGCTGAAATTTTATCATTTCTCATCTCTAAAATCACATATCCCCATGTAATAC

AAGGGTCTTTCCATTATGCATTCATTAAATCATTCTAGGAGAGGTCTCATCAACCTCCTA

CTTTATTAAACATGCCCACAGAGAGAAGGGCACAGGAGTAAAGCAGAGGCAATGTGTCAT

TGCTCCCAAGTAGAAGGTAAATAAGGCCTCTTTGACCAGCAGGAGAGGAAATGCTGGTAG

GAAGACTCTTCCAGGATGTAATGCAGAAGCTCAGGGCAGAGCTATTCACACTTCACACCA

GTGCTGTTTCCTCACCATAGAGGTTCCTGAGGTCACAGTGTTTTCCAAGTCTCCCGTGAC

ACTGGGTCAGCCCAACACCCTCATCTGTCTTGTGGACAACATCTTTCCTCCTGTGGTCAA

CATCACCTGGCTGAGCAATGGGCACTCAGTCACAGAAGGTGTTTCTGAGACCAGCTTCCT

CTCCAAGAGTGATCATTCCTTCTTCAAGATCAGTTACCTCACCTTCCTCCCTTCTGCTGA

TGAGATTTATGACTGCAAGGTGGAGCACTGGGGCCTGGATGAGCCTCTTCTGAAACACTG

GGGTAAGGATGAGTTTCACCATTTTTTGATGCTTTCTTGTCTGTCAAGTTCAGAACTTCC

TGCCTTTTACTCTATGTCCCAAAACTTGTTTTCCACACTTCATGAGTTTCTTTTATCTTT

TTTTTTTTTTGAAAGAATTAAGCAACAAAAGCACAGATTTATTAAAAAAGAAAGTACACT

CCACAGGGTGGGAGCAGGCCTGCCACTTCATGGGTTTCTAATAACAGACTTCACTCTCCT

CCCTGAGCCAGGGGCCTTGAGTCTTTGCAGAGCCAACCCTCCACCCCATCCCATCCCTCA

CACATGCACATGAGCACACTCTGCATTCTGACCTCAACAACTTCACTTCCACAGAGCCTG

AGATTCCAGCACCTATGTCAGAGCTCACAGAGACTGTGGTCTGTGCCCTGGGGTTGTCTG

TGGGCCTCGTGGGCATTGTGGTGGGGACCGTCTTGATCATCCGAGGCCTGCGTTCAGTTG

GTGCTTCCAGACACCAAGGGCCCTTGTGAATCCCATCCTGAAAAAGAAGGTAAGTTTGAG

ATTTGTTAGAGCTGAAGCTGCAGGAAGGAAAGTGGGAGGAGGCTGTGGACATGAATGTGG

TTGAAAGTTGTAGGGGAATTGGGAAGTGGCATGATGATGACACAGGAGCCCCCTTGGACC

CATCGATCTCATGTCTGTCCTGTTGCAGGTGCATCACCATCTACAGCAGCGGAAGAGTGG

ACTTGCTACATGACCTAGCACTATTCTCTGGCCCGATTTATCATACCCTTTTTCTCCTGC

AAATGTTTCTCCTCTTACCTTTTCTCTGCTTTTTTTTCCTTAAGCTTCTGTATCCCCTCA

GAGCTCACAAATGCCTTTGAATTCTTTCCCTGACCTCCTGATTTTTTTTTCTTTTCTCAG

GTGTTACCTACTAAGAGATGCCTGGGGTAAGCCGCCCAGCTAC

>HLA:HLA00608 DQA1*03:01:01:01

AAAAATTCCTGGAGGTTGTAACCCAGAAAATCCTGAAGGATGCCATATAACTGATGACCT

CATCTATCCATGAGGCTGCTCAGAAATGCCCTCCCCTGGCCAGGCACGGTGGCTCATGCC

TGTAATCCAAGCACTTTGGGAGGCTGAGGCAGGCAGATCATGAGGTCAGGAGTTCAAGAC

CAGCGTGGCCAACATAGTGAAACTCTGTCTCTACTAAAAATACAGAAATTAGCCGGGCAT

GGTGGCAGCCGCCTGCAGTCCCAGCTACTCGGGAGGGTGAGGCAGGAGAATCGCTTGAAC

CTGGGAGGCAAAGGTTGCAGTGAGCCGAGACCATGCCATTGTACCTCAGCCTGGGTGACA

GAGTGAGACTACGTCTCGAAAAGAAAAGAAAAAGAAAAGAAAAAGAAAAAAAAAGAAAAA

TGCCCATCCCTCTTGCGAATGGCAGACATGCACACACCAGAGAAGATTCCAATTTAGTGT

CCTCCCTCTGTTCATAGAACAATTCCTCAAGTCCACTCTGAGTAGAGGCTGCATCACAAC

AAGGGGATTGCCCTGTCTCCTTCCAGGGCTCTTAATACAAACTCTTCAGCTAGTAACTGA

GATGTCACCATGGGGGATTTTTCTAATTGGCCAAAACCTGACCTGGCAGGGTTTGGTTTG

GGTGTCTTCAGATTTCCTTGTCTTGAGGCCCTCACAATTGCTCTACAGCTCAGAACAGCA

ACTGCTGAGGCTGCCTTGGGAAGAGGATGATCCTAAACAAAGCTCTGATGCTGGGGGCCC

TCGCCCTGACCACCGTGATGAGCCCTTGTGGAGGTGAAGACATTGTGGGTGAGTGCATGA

GTGAGGAATGTTCTCTGGAGCTGAAAAACAGTAAATTGAAGGAAAAGAGAGAAAGCGATT

TGCAGAGAAATTGTAGAGATTTCCTAAGACCCCTTTCAGTATTAAGAGAATTAAAAATTA

TAGCTGTTCCTCCTTCAGGAAACCAGAGCCCCAACCTACTCTTTTTGTTATGTATGCTTT

TGTGTTCACTAAGGATGCTATTCTGTTTATATTATATTCAGTGACAACAGCCTGGAGGTC

TCTATGTCGTTCCGTCATGATTGCCTCAAAAATTAGTGAAGTTTCCATCAGTGGATAATT

TTTTATTATTAAAAATGTATGAAGTGTCATTCTCAAATTTCCCTGAACAACTTTTGAAGC

TTTTCGTATGTCTCCTGTAGTAGATCTTGGGGTCGTTCCATCAATTATATACTCTATAGA

TATTAAAAAAGTTGCCCGTTTCTTTCTCTCAGACTTACTCACATTTCCACATGGGAACTG

GCACAGGTGGGGAGTGGGTAAAGGAGTCCAGCAGGCTGAATGCCTTCAACAATCATTTTA

CCACATGGTCCTCACTTACTCTCAGCTGCCTCATATGTGTCACCTCACAAATAATCAAAT

AAAATGGGCATGTAGCTAAGCTTTGTAAATAGTGAAAACATGGATGTCAATTGTTTTTAC

ATATTTCTATTACAGGTATAGCTTCACATTTTTCTTTAGCAAAATAAGGGATCCTTTTAG

TTTAAAATTGAGAAGTAGAAAAAATTGGTAAATTAAATCATTTTATTCTCAAATTATCAA

CCCAAATTACCTGTTCTTCACCTCATCTAATAAAGTCCTATAAAAAGAAAAGTGGGCCAG

ACATGGTGGCTCATGCCTGTAATCCCAGCACTTTGGGAGGCCGAAGCAGGAGGATCATTT

GAGCCTGGGAGTTTGAGACCAGCCTGGGCAACATAGCAAGACCTCATCTCTACCAAAAAA

TAAAATAAAAATTAGCCAGGTGTGGTGGTGCATGCCTGTGGTGCCAGCTACTCAGAAGGC

TGCAGTGGGAGGAGCACTTGAGTCCAGGAGGTGGAAGCTGCAGTGAGCCATGATGGCACC

ACTACACTCCAGCCAGGGCAACAGAGAGAGACTCTGTCTCAAAAAGAAAGAGGAAAGAAA

GAGAGAAAGGAAGGAAAGAAGGAAAGAAGGAAGGAAGGAAGGAGAAAGGGAAGGGAGGAA

GGAAAAAAGAAAGAAAGAAAGAAAACGGAAGGAAGGAAGCACAGATTAATTATTTGGTCT

CTTTGTCTCCTCTGCCTTTGTCGTCCATCTCTTCCCACCTCTCTTCATGCATTCCTTTCT

CCCTCTTCCCTTTCAGGATCCATCTCTGACTCCCTGCTCCTTTATAGAGATGGACAGTGA

GTTTGTAAAACAAAAGTTGAAAAGTCAGATAGTTAAAAGGGGAAGTGAACTGGAAGGTAC

TCTAAACTTTCACAACCTTATTAACCATGGCTGCTCCCATTCTGATTTTGTTCAGCAGTG

GAAGTTTCACCCGCTCCTCCAGAGCGCTTGGCTTCTTTGTTCCAAATTTCCTTTCTTCAA

CCTCACACCAGAGTGCCCTGGTCAGGCTCAGCTCATCCATTAGGCACAATGTGGGCAGTG

CAGGGGACCCTCCAGACTGTAAAGCCACATGAGAATGTTTTAACTCCTTTTAAAATTATA

AAAAAATGAAATTGTAGAGCCTAAGAAAATGTTTTAACTTTTAATTCAGCCTAGATTATA

TTGTCTTTATACCAATTCAGTCATAAAATATAGTTTTCCATATTTTTATGGAGGAAGGCG

TCCACACAAGCAAGAGTGCTTGGGGCTCACATGTCAGAACGCAACCCTGATCATGGCTGA

TCCTGGCCTTCGTGTGGTTCTGCTAACTATGTGCCTGTCAGTCTTCCCCAAAATCTATGT

GGTCCTCAAATATAACAACTGTCATTCAATACACATGTTTGAGCACCCAGTGAGCTAAGT

TTTAAGGATTCAAAGATGAAAAGTCATGCTGTCTCCTCTGCAAAGGGTGCTCAGACTAGT

GATGGAAACAGTATGGGATGAAAGAAAGCAGAAGGCCATTGCTGAGCAGGCAGTGGACTC

AGCAGAGGCTGAAACTATACAAGTGACTTGGTTCCAGCTGGGCCAGCAGGATAACCAGAT

GAAAAGAAGGATTGCATATATTCCATATATATTTATGTTTGAACAAAGAGTCAAGGTTTA

TTGCAAGGATAAGGAGGTTTTGTTGGTGGCCTGTTAAGACCATCCAGGGTGGTCATACTG

GATAGGGAAGAAGGTGAGCTGGAAGAGGAACAGACAAACTTGGATGGCCAGATGTTGAGA

TGGAGGAGATGGAGGTCATAACGTGGTCAAAAACATGTTGATGAGAGGACTTAGCTACAA

AGTTGTTAACTTAAGCAGAAACCTCAAGGATTGATTTTATGATTTCTCCAGGAAGTCCTA

AAAGTTAATTTCATTTCAGGGAGAAAAACAACAGACCACTGCAAAGACCAGGAACATGAA

AGGATAATGTAGTTTGGTTTGCTTGGCAGATACTTGTGAAAGATGTTGGACTGTAAGGCT

GTCAATATCCTCCTCGCAGAACTTACTACAGTACATTGTATCTGCTCCCTTACCTACCTG

ACTCTCCCACTATTCAGTTTGTTCCTTAATGGTAGACCATGCCTGATCGGTGTTTTACAC

ATCCCCTGCTATGTCTGATACTTGTGGATGCTCAGAAAGTGGGGAAGGAAGGAAAGATAC

GATGGTAAAAGGCTTACACATGTCTTGAGCAGAATGTTCAGTTTGGCTCATTTGGCTGGA

GTCATACTGCATGGCTGCCATTCTGCTCTGGCATCCTCAGAGAAGCACACTGCCCATTAA

AGGAAAAAGGGTGAATACAAATGTTGAGTCAGAACACTGCAGACATTTAGTAACCTCCTT

CAGAGGAAAAAAAAAGGTGGGGGGAATGACAGAAATCCAAAAACTAGTAGAGCTTCCACT

TTTTCATTTCAGAAGAAATCAGTTACTCTCCTCTAAGGACCATTACTATTAACAAAACAG

AGACCTTAGAAGGAAGCATTATTTACTTATCATATATTTTGTAATGTTATTACCCTTCTT

GTTATACTCTTTCTTATACCCTACCATTGTTAGCAGAAATTATTTTAAATTAATAAGATC

CTGCATGCTTTTCCTTTTTCTAAAAAAAGAAAGATCTCTGTGTAGAATGTCCTGTTCTGA

GCCAGTCCTGAGAGGAAAGGAAGTATAATCAATTTGTTATTAACTGATGAAAGAATTAAG

TGAAAGATAAACCTTAGGAAGCAGAGGGAAGTTAATCTATGACTAAGAAAGTTAAGTACT

CTGATAACTCATTCATTCCTTCTTTTGTTCATTTACATTATTTAATCACAAGTCTATGAT

GTGCCAGGCACTCAGGAAATAGTGAAAATTGGACACGCGATATTCTGCCCTTGTGTAGCA

CACACCGTAGTGGGAAAGAAAGTGCACTTTTAACCGGACAACTATCAACACGAAGAGGGG

AGGAAGCAGGGGCTGGAAATGTCCACAGACTTTGCCAAAGACAAAGCCCATAATATCTGA

AAGTCAGTTTCTTCCATCATTTTGTGTATTAAGGTTCTTTATTCCCCTGTTCTCTGCCTT

CCTGCTTGTCATCTTCACTCATCAGCTGACCATGTTGCCTCTTACGGTGTAAACTTGTAC

CAGTCTTATGGTCCCTCTGGGCAGTACAGCCATGAATTTGATGGAGACGAGGAGTTCTAT

GTGGACCTGGAGAGGAAGGAGACTGTCTGGCAGTTGCCTCTGTTCCGCAGATTTAGAAGA

TTTGACCCGCAATTTGCACTGACAAACATCGCTGTGCTAAAACATAACTTGAACATCGTG

ATTAAACGCTCCAACTCTACCGCTGCTACCAATGGTATGTGTCCACCATTCTGCCTTTCT

TTACTGATTTATCCCTTTATACCAAGTTTCATTATTTTCTTTCCAAGAGGTCCCCAGATC

TTCTCATGGCAATTGCTGAAATTTTATCATTTCTCATCTCTAAAATCACATATTCCAATG

TAATACAAGGGTCTTTCCATTATGCATTCATTAAATCCTTCTAGGAGAGGTCTCATCAAC

CTTCTACTTTATTAAACATGCCCACAGAGAGAAGGGCACAGGAGTAAAGCAGAGGCAATG

TGTCGTTGCTCCCAAATGTGTCGTTACAATGTGTCGTTGCTTACCCAAAGAGGTAAATAA

GGCCTCTTTGACCAGCAGGAGAGGAAATGCTGGTAGGAAGACTCTTCCAGGATGTAATGC

AGAAGAAGCTCAGGGCAGAGCTATTCACACTTTACACCAGTGCTGTTTCCTCACCATAGA

GGTTCCTGAGGTCACAGTGTTTTCCAAGTCTCCCGTGACACTGGGTCAGCCCAACACCCT

CATCTGTCTTGTGGACAACATCTTTCCTCCTGTGGTCAACATCACCTGGCTGAGCAATGG

GCACTCAGTCACAGAAGGTGTTTCTGAGACCAGCTTCCTCTCCAAGAGTGATCATTCCTT

CTTCAAGATCAGTTACCTCACCTTCCTCCCTTCTGCTGATGAGATTTATGACTGCAAGGT

GGAGCACTGGGGCCTGGATGAGCCTCTTCTGAAACACTGGGGTAAGGATGAGTTTCACCA

TTTTTTGATGCTTTCTTGTCTGTCAAGTTCAGAACTTCCTGCCTTTTACTCTATATCCCA

AAACTTGTTTTCCACACTTCATGAGTTTCTTTTGTCTTTTTTTTGAAAGAATTAAGCAAC

AAAAGCACAGATTTATTAAAAAAGAAAGTACACTCCACAGGGTGGGAGCAGGCCTGCCAC

TTCATGGGTTTCTAATAACAGACTTCACTCTCCTCCCTAAGCTGGGGGCCTTGAGTCTTT

GCAGAGCCAACCCTCTACCCCATCCCATCCCACACACATGCACATGAGCAAACTCTGCAT

TCTGACCTCAACAACTTCACTTCCACAGAGCCTGAGATTCCAACACCTATGTCAGAGCTC

ACAGAGACTGTGGTCTGCGCCCTGGGGTTGTCTGTGGGCCTCGTGGGCATTGTGGTGGGG

ACCGTCTTGATCATCCGAGGCCTGCGTTCAGTTGGTGCTTCCAGACACCAAGGGCCCTTG

TGAATCCCATCCTGAAAAGGAAGGTAAGATTGAGATTTGTTAGAGCTGAAGCTGCAGGAA

GGAAAGTGGGAGGAGGCTGTGGACATGAATGTGGTTGAAAGTTGTAGGGGAATTGGGAAG

TGGCATGATGATGACACAGGAGCCCCCTTGGACCCATCGATCTCATGTCTGTCCTGTTGC

AGGTGCATCACCATCTACAGCAGCGGAAGAGTGGACTTGCTACATGACCTAGCACTATTC

TCTGGCCCGATTTATCATACCCTTTTTCTCCTGCAAATGTTTCTCCTCTTACCTTTTCTC

TGCTTTTTTTTTCTTAAGCTTCTGTATCCCCTCAGAGCTCACAAATGCCTTTGAATTCTT

TCCCTGACCTCCTGATTTTTTTTTCTTTTCTCAGGTGTTACCTACTAAGAGATGCCTGGG

GTAAGCCGCCCAGCTAC

>HLA:HLA00613 DQA1*05:01:01:01

AAAAATTCCTGGAGGTTGTAACTCAGAAAATCCTGAAGGATGCCGTATAATTGATGATGT

CATCTATCCACGAGGCTGCTCAGAAATGCCCACCCCTGGCCAGGGCGGTGGCTCATGCCT

GTAATCTGAGCACTTTGGGAGGCTGAGATGGGCAGATCACGAATTCAGGAGTTCGAGACC

AGCCTGGCCAACATAGTGAAACTCTGTCTCTACTAAAAATACAAAAATTAGCCGGGCGTG

GTGGCAGGTGCCTGTAATCCCAGCTACTTGGGAGGGTGAGACAGGAGAATCACTTGAACC

CGGGAGTCAAAGTTTGCAGTGAGCCGAGACCATGCCATTGCACCTCAGCCTGGGTGACAG

AGTGAGACTACATCTCAAAAAAAAAAAAAAAGAAAGAAAGAAAAAAAGAAAAGAAATGCC

CACCCCTCTTGCCACTGGCAGACATGCACACACCAGAGAAGATTCCGATTTCGTGTCCTC

CCTCTATTCACAGAACATTTCCTCAAGTCCACTCTGAGTGGAGGCTGCATCACAACAAGG

GGATTGCCCTGTCTCCTTCCAGGGCTCTTAATACAAACTCTTCAACTAGTAACTGAGGTG

TCATCATAGGGGATTTTTCTAATTAGCCAAAACCTGACTTGGCAGGGTTTGGTTTGGGTG

TCTTCAGATTGCCTTGTCTCGAGGTCCTCACAATTGCTCTACAACTCAGAACAGCAACTG

CTAAGGCTGCCTTGGGAAGAGGATGATCCTAAACAAAGCTCTGATGCTGGGGGCCCTTGC

CCTGACCACCGTGATGAGCCCCTGTGGAGGTGAAGACATTGTGGGTGAGTGTATGAGTGA

GGGATGTTCTCTGGAGCTGGAAGAGAGGAAATTGAAGCAAAAGAGAGAAAGCGATTTGCA

GAGAAATTGTAGAGATTTCCTAAGGGTCCCTTCAGTATTAAGAGATTTAAAAATTATGGC

TGTTCCTCCTTCAGGAAACCAGAGTCCCAACCTACTCTTTTTGTTATCTATGCTGTTGGC

GTTCACTAAGGATGCTATTCTGTTTATATTGTATTCAGTGACTATAGCCTGGAGGTCTCT

ATGTCATTCCATCATGATTGCCTCAAAAATTAGTGAGGTTTCCATCAATGGATAATTTTT

TATTATTAAAAATTTGTGAAGTATCATTCTCAAATTTCCCTGAACAACTTTTGAAGCTTT

TCGGATGTCTCCTGTAGTAGCGCTTGGGGTAAATGATTCCATCAATTATATACTCTATAG

ATATTAAGAAAGATGCCCTTTTCTTTCTCTCAGACTTACTAACATTTCCACGTGGGAACT

GGCACAGGTGGGGAGTGGGTAAAGGAGTCCAGCAGGCTGAATGCCTTCAACAATCATTTT

ACCACATGGTCCTCATTTACTCTCAGCTGCCTCATATGTGTCACCTCACAAATAATCAAA

TAAAATTGGCATGTAGCTAAGCTTTGTAAATAGTGAAAACATGAATGTCAATTTTATTTT

TACATATTTCTATTATAGGTATAGCTTCACATTTCTTTTCTTTTGCAAAATAAGGTATCC

TTTTATTTTAAAATTGAGAATTTATAGTAGAAAAACTTGGTAAATTAAATCATTTTATTC

TCAAATTATCAACCCAAATTACCTGTTCTTCAACTCATCTAATGAAGTCTTATAAAAAGA

AAAGTGGGCCAGACATGGTGGCTCATGCCTGTAATCCCAGCACTTTGGGAGGCCGAGGCA

GGAGGATCCTTTGAGCCCAGGAGTTGAGACCAGCCTGGGCAACATAGCAAGACCTCATCT

CTACAAAAAATAAAAATTAGCCAGGTGTGGTGGTGCATGCCTGTGGTGCCAGCTACTCAG

AAGGCTGCAGTGGGAGGACTGCTTGAGTCCAGGAGGCGGAAGCTGCAGTGAGCCATGATG

GCACCACTACACTCCAGCCAGGGCAACAGAGAGAAACTCTGTCTCAAAAAGAAAAAGGAA

AGAAAGAGAGAAAGGAAGGAAAGAAAGAAAGAAGGAAGGAAGGAAGGAAGGAAGAAGGGG

AAGGGAAGGGAAGGAAGGGAAGGAAAGGGGAGGAAAGAAGGAAGGAAAAAAGAAAAAGAA

AGAAAAAAGAAAGAAAAAGAAAGAAAGAAAGAAAAGAGGGAAGGAAAGAAAGAAAAAGGA

AGGAAGGAAGCACAGATTAATTATTAATCATTTGGTCTCTCTTAGTCTTCTCTGTCTTTG

TCATCCATCTATTTCCACCTCTCTTCATGCATTCCTTTCTCCCTCTTCCCTTTCAGGATC

CATCTCTGACTCCCTGCTTCTTTATATGGACAGTGGGGTTTGTAAAACAAAAGTTGAAAA

ATCAGATAGTTAAAAGGTGAAGTGAACTGGAAGGTCTAAACTTCCACAACCTTATTAACC

ATGGCTGCTCCCATTCTGATTTTGTTCGACGGTGGAAGTTTCACCTCCTTCTCCAGAGCA

CTTGGCTTCTTTGTTCCAAATTTCCTTTCTTCAACCTCACACCAGAGTGCCCTGGTCAGG

CTCGGCTCATCCATTAGGCACAACATGGGCAATGCAGAGAACCCTCCATACTGTAAAGCC

ACATGAGAATGTTTTAACTCCTTTTAAGATTAGAAAAAAATGAAATTTCAGAGCCTAAGA

AAATGTTTTAATTCAGCCTAGATTGTATTGTCTTTATACCAATTCAGTCATAAAATACAA

TTTTCCATATTTTCATGGAGGAAGGGGCCCACAAAAGCAAGAGTGCTCAGGGCTCACATG

TCAGAATGCAGCCCTGGTCATGGCTGATCCTGGCCTTCTTATGATTCTGCTAGTGTGCCT

GTCCATCTTCCCCAAAATCTATGTCGTCCTCAAATATAGCAACTGTAATTCAAAACACGT

TTGAGCACACAGTAAGCTAAGTTTTAAGGATTCAAAGATGAAAAGTCATGCTGTCTTCCC

TGCAGAGGGTGCTCAGACTAGTGACGGAAACGGTATGGGATGCAGGAAAGCAGAAGGCCA

TTGCTGACCAGGGCAGTGGACTCAGCAGAGGCTGAAACTATACAAATGACTTGGTTCCAG

CTGGGCCAGCAGGGTGACATCCTCCAGCAAAACTCGGCACCCAAGACAAGTCCCAGATGA

AAAGAAGGATTGCTTTGTGTTTGATAAGGAGTCAAGGTTTATTGCAAGGATAAGGCTTTG

TTGGTGGCCTGTTAAGACAATCCAGGGCAGTCATACTGGATAAGGAAGAAGGTGAGCTGG

AAGAGGAACAGACAAACAGCCAGATATTGAGGTGGAGGAGCTGGAGGTCATAACGTGGTC

AAAAACATGTCGATGAGAGGACTTAGCTACAAAGTTGTTAACTTAAGCAGAAACCTCAAG

GATGAATTTTAGGATTTCTCCAGGAAGTCCTAAAAGATAATTTCATTTCAGGGAGAAAAA

CAACAAACCACTGGAAAGACCAGGGAACATGAAAGGATAATGTAGTTTGGTTTGCTTGGC

AGATACTTATGAAGGATGTTGGACTGTAAGGCTGTTGATAGCCTCCTCACAGAACATGCT

ACAGTACATTGTATCTGCTCCCTTACCTACCTGACTCTTCCACTCTTCAGTTTGTTCCTT

AATGGTAGACCATGCCTGATTGGTGTTTTACACATCCCCTGCTATGTCTGACACTTGTGG

ATGCTCAGAAAGTGGGGAAGGAAGGAAAGATACAACGGTAAAAGGCTTACACGTCTTGAC

AGGAATGTCCAGTTCGGCTCATTTGGCTGGAGCCACATTGCACGGCTGCCATTCTGCTCT

GGCATCCTCAGACAAGCACACTGCCCATTAGAGGAAAAAGGGTTTAATTTACCTGAGTCC

TCAAGTGAATATAAGTGTTAAGTCAGAACACTGTAGACATTTAGTAACCTCCTTCAGAGG

AAAAAAAAAGGTGGGGGGAAATGACAGAAATCCAAAAACTAGTAGAGCTTCCACTCTTCA

TTTCAGAAGAAATCAGTTGCTCTCCTCTAAGGACCATTACTATTAACAAAACAGAGACCT

TGAAGGAGGCATTGTTTATTTATTATATATTTTGTAATGTTATTACCATTCTTGTTATAC

TCTTCCTTATACCCTACAATTGTTAGCAGACATTATTTTAAATTAATAAGATCCTGCATG

CTTTTCTTTTTTTTAAAAAAAGAAAGACCTCTGTGTAGAGTGTCCTGTTCTGAGCCAGTC

CTGAGAGGAAAGAAAATACAATCAGTTTGTTATTAACTGATGAAAGAATTAAGTGAAAGA

TGAATCTTAGGAAGCAGAAGGAAGTAAACCTAATCTCTGACTAAGAAAGCTAAATACCAT

AATAACTCATTCATTCCTTCTTTTGTTCAATTACATTATTTAATCATAAGTCCGTGATGT

GCCAGGCACTCAGGAAATAGTAAAAACTGGACATGTGATATTCTGCCCTTGTGTAGCGCA

CATTATAGTGGGAAAGAAAGCGCAATTTTAACCGGACAACTACCAACAATAAGAGTGGAG

GAAGCAGGGGTTGGAAATGTCCACAGGCTGTGCCAAAGATGAAGCCCGTAATATTTGAAA

GTCAGTTTCTTTCATCATTTTGTGTATTAAGGTTCTTTCTTCCCCTGTTCTCCACCTTCC

TGCTTGTCATCTTCACTCATCAGCTGACCACGTCGCCTCTTATGGTGTAAACTTGTACCA

GTCTTACGGTCCCTCTGGCCAGTACACCCATGAATTTGATGGAGATGAGCAGTTCTACGT

GGACCTGGGGAGGAAGGAGACTGTCTGGTGTTTGCCTGTTCTCAGACAATTTAGATTTGA

CCCGCAATTTGCACTGACAAACATCGCTGTCCTAAAACATAACTTGAACAGTCTGATTAA

ACGCTCCAACTCTACCGCTGCTACCAATGGTATGTGTCAACAATTCTGCCCCTCTTTACT

GATTTATCCCTTCATACCAATTTTCATTATTTTATTTCCAAGAGGTCCCCAGATCTTCTC

ATGGCAATTGCTGAAATTTTATCATCTCCCATCTCTAAAATCACATATTCCCATGTAATA

CAAGGGTCTTTCCATTATCCATTCATTAAATCCTTCTCGGAGAGGTCTCATCAACCTCCT

ACTTTATTAAACATGCCCACAGAGAGAAGGGCACAGGAATAAAGCGAAGGCAATGTGTCG

TTGCTCCCAAGCAGAAGGTAAATAAGACCTCTTTGACTATCAGGTGGTGAAATGCTGGTA

GGAGGGCTCTTCCAGGATGTAATGCAGAAGCTCATGGCAGAGCTATTCACACTTCACATC

AGTGCTGTTTCCTCACCACAGAGGTTCCTGAGGTCACAGTGTTTTCCAAGTCTCCCGTGA

CACTGGGTCAGCCCAACATCCTCATCTGTCTTGTGGACAACATCTTTCCTCCTGTGGTCA

ACATCACATGGCTGAGCAATGGGCACTCAGTCACAGAAGGTGTTTCTGAGACCAGCTTCC

TCTCCAAGAGTGATCATTCCTTCTTCAAGATCAGTTACCTCACCCTCCTCCCTTCTGCTG

AGGAGAGTTATGACTGCAAGGTGGAGCACTGGGGCCTGGACAAGCCTCTTCTGAAACACT

GGGGTAAGGATGAGTTCCACCACTTTTTGATGCTTTCTTGTCTGTCAAGTTCAGAACTTC

CTGCCTTTTACTCCTATATCCCAAAACTTGTTTTCCACACTTCATGGGTTTCTTTCTTTC

TTTTGAAAGAATAAAGCAACAAAAGCAAAGATTTATTGAAAATGAAAGTACACTTCACAG

GGTGGGAACGGGCCTGAGCATAGGGGCTCAAGAGCCACTTCATGGTTTTCTAATGATAGA

CTTCACTCTCCTCCCTAAGCTGGGGACCTTGAGTCTTTGCAGAGCCAACCCTCCACCCCA

TCCCATCCCACACACATGCACATGAGCACACTCTGCTTTCTGACCTCAACGACTTCATAT

CCACAGAGCCTGAGATTCCAGCCCCTATGTCAGAGCTCACAGAGACTGTGGTCTGCGCCC

TGGGATTGTCTGTGGGCCTCGTGGGCATTGTGGTGGGCACTGTCTTCATCATCCGAGGCC

TGCGTTCAGTTGGTGCTTCCAGACACCAAGGGCCCTTGTGAATCCCATCCTGGAATGGAA

GGTAAGATTGAGATTTGTTAGAGCTGAATCCGCAGTATGAGAGGAAGGAAAGTGGAGGAG

GCTGTGGACATGAATGGTTGAAAGTTGTAGGGGAATTGGGAAGTGGCATGATGATGACAT

AGGAGCGGCCTAGGACCCATCCATCTCATGTCTGTCCTGTTGCAGGTGCATCGCCATCTA

CAGGAGCAGAAGAGTGGACTTGCTACATGACCTAGCATTATTTTCTGGCCCCATTTATCA

TATCCCTTTTCTCCTCCAAATGTTTCTCCTCTCACCTCTTCTGTGGGACTTAAATTGCTA

TATCTGCTCAGAGCTCACAAATGCCTTTGAATTATTTCCCTGACTTCCTGATTTTTTTCT

TCTTAAGTGTTACCTACTAAGAGTTGCCTGGAGTAAGCCACCCAGCTAC

>DQA1_in_KAS116

AAAAATTCCTGGAGGTTGTAAGCCAGAACATCCTGAAGGATGCCGTATAACTGATGACCT

CATCTATCCACGAGGCTGCTCAGAAATGCCCACCCCTGGCCAGGCACGGTGGCTCATGCC

TGTAATCTGAGCACTTTGGGAGGCTGAGACGGGCAGATCATGAGGTCAGGAGTTCAAGAC

CAGCGTGGCCAACATAGTGAAACCCCGTCTCTACTAAAAATACAAAAATTAGCTGGGCGT

GGTGGCAGGTGCCTGTAGTCCCAGCTACTTGGGAGGGTGAGGGAGGAGAATCGCTTGAAT

CCGGGAGGCAAAGGTTGCAGTGAGCCGAGACCTTGCCATTGTACCTCAGCCTGGGTGACA

GAGTAAGACTACGTCTCAAAAAGAAAGAAAAAGAAAAGAAAAAGAAAAAAAAAGAAAAAC

ACCCATCCCTCTTGCGACTGGCAGACATGCACACACCAGAGAAGATTCCAATTGAGTGTC

CTCCCTTTATTCATAGAACAATTCCTCAAGTCCACTCTGAGTAGAGGCTGCATCACAAGG

GGATTGCCCCGTCTCCTTCCAGGGCTCTTAATACAAACTCTTCAACTAGTAACTGAGATG

TCACCATAGGGGATTTTTCTAATTGGCCAAAACCTGACTTGGCAGGGTTTGGTTTGGGTG

TCTTCAGATTTCCTTGTCTTGAGGTCCTCACAATTACTCTACAGCTCAGAACACCAACTG

CTGAGGCTGCCTTGGGAAGAGGATGATCCTAAACAAAGCTCTGCTGCTGGGGGCCCTCGC

TCTGACCACCGTGATGAGCCCCTGTGGAGGTGAAGACATTGTGGGTGAGTGCATGAGTGA

GGGATGTTCTCTGGAGCTGAAAAACAGTAAATTGAAGGAAAAGAGATAAAGCGATTTGCA

GAGAAACTGTAGAGATTTCCTAAGGGCCCTTTCAGTATTAAGACAATTAAAAATTATAGC

TGTTCCTCCTTCAGGAAACCAGAGCCCCAACCTACTCTTTTTGTTATCTATGCTGTTGTG

TTCACTAAGGACGCTATTCTGTTTATATTATATTCAGTGACTACAGCCTGGAGGTCTCTA

TGTCGTTCCGTCATGATTGCCTCAAAAATTAGTGAGGTTTCCATCAGTGGATAATTTTTT

ATTATTAAAAATTTATGAAGTGTCATTCTCAAATTTCCCTGAACAACTTTTGAAGCTTTT

CGGATATCTCCGGTGGTAGATCTTGGGGTCATTCCATCAATTATGTACTCTATAGATATT

TAAAATGTTGCCCATTTCTTTCTCTCAGACTTACTTACATTTCCACATGGGAACTGGCAC

AGGTGGGGAGTGGGTAAAGGAGTCCAGCAGGCTGAATGCCTTCAACAATCATTTTACCAC

ATGGTCCTCACTTACTCTCAGCTGCCTCATATGTGTCACCTCACAAGTAATCAAATAAAA

TGGGCATGTGGCTAAGCTTTGTAAATAGTGAAAACATGAATGTCAATTTTTTTTACATAT

TTCTATTACAGATATAGCTTCACATTTCTTTTCTTTAGCAAAATAAGGGATCATTTTATT

TTAAAATTGAGAAGTAGAAAAAATTGGTAAAATAAATCATTTTATTCTCAAATTATCAAC

CCAAATTACCTGTTCTTCACCTCATCTAATAAAGTCCTATAAAAAGAAAAGTGGGCCAGA

CAAGGTGGCTCATGCCTGTAATCCCAGCACTTTGGGAGGCCGAGGCAGGAGGATCATTTG

AGCCTGGGAGCTTGAGACCAGCCTGGGCAACATAGCAAGACCTCATCTCTACAAAAAAAG

AAAAATAAAAATTAGCCAGGCATGGTGGTGTGTGCCTGTGGTGCCAGCTACTCAGAAGGC

TGCCGTGGGAGGACCACTTGAGTCCATGAGGCGGAAGCTGCAGTGAGCCATGGTGGCACC

ACTACACTCCAGCCAGGGCAACAGAGAGAGACGCTGTCTCAAAAAGAAAGAGGAAAGAAA

GCGAGGAAGGAAGGAAGGAAGGAAGGAGAAGGAAGGAAGGAAGGAAAAGAAAAGAAAAGG

GAAGGGAGGAAGGAAGGAAAAAAGAAAGACAGAAAGAAAGAGGAAGGAAAGAAGAAAGGA

AGCACAGATAATTATTTGGTCTCTTACTCTCCTCTGCCTTTGTCGTCCATCTCTTTCCAC

CTCTCTTCATGCATCCCTTTCTCCCTCTTCCCTTTCAGGATCCATCTCTGACTCCCTGCT

CCTTTATAGAGATGGACAGTGGGTTTGTAAAACAAAAGTTGAAAAGTCAGATAGTTAAAA

GGGGAAGTGAACTGGAAGGTACCTTAAACTTTCACAACTTTATTAACCGTGGCTGCTCCC

ATTATGATTTTGTTCGGCAGTGGAAGTTTCACCTGCTTCTCCAGAGCACTTGGCGTCTTT

GTTCCAAATTTCCTTTCTTCAACCTCACACCAGAGTGCCCTGGTCAGGCTTGGCTCATTC

ATTAGGCACAATGTGGGCAGTGCAGGGGACCCTCCATACTGTAAAGCCACATGAGAAGGT

TTTAACTCGTTTTAAAATTAGAAAAATAATGGAATATTAGAGCCTAAGAAAATGTTTTAA

CTTTTAATTCAGCCTAGATTATATTGTCTTTATACCAATTCAGTCATAAAATATAATTTT

CCATATTTTTGTGGAGGAAGAGGCCCATACAAGCAAGAATGCTCGGGGCTCACATGTCAG

AATGCAGCCTTGGTCATGACTGATCCTGGCCTTCGTATGGTTCTGCTACCTGTGTGCCTG

CCCGTCTTCCCCAAAATCTATGTGGTCCTCGAATATAGCAACTGTCATTCAACACACATG

TTTGAGCACACAGTGAGCTAAGTTTTAAGGATTCAAAGATGAAAAATCATGCTGTCTTCC

CTGCAGAGGGTGCTCAGATTAGTGATGGAAACAGTATGGGGTGCAGGAAAGCAGAAGGCC

ATTGCTGAGCAGGGCAGTGGACCCAGCAGAGGCTGAAACAATACAAGTGACTTGGTTCCA

GGTGGGCCAGCAGGGTGACGTCCTCTAGCAAAATTTGGCACCCAAGACAAGTACCAGAAG

AAAAGAAAGACTGCATGTATTCCACATATATTCATGTTTGAACAAGGAGTCAAGATTTAT

GGCAAGGATAAGGAGGCTTTGTTGGTGACCTGTTAAGACCAACCAGGGCAGTCATGCTGG

ATAGGGAAGAAGGTGAGCTGGAAGAGGAACAGACAAACTTGGACAGCCAGATGTTGAGAC

GGAGGGGTTGGAGGTCATAATGTGGTCAAAAACATGTTGATGAGAGGACTCAGCTACAAA

GTTGTTAACTTAAGCAGAAACCTCAAGGATGGATTTTAGAATTTCTCCAGGAAGTCCTAA

AAGATAATTCCATTTCAGGGAGAAAAACAACAGACCACTGCAAAGACCAGGGAACATGAA

AGGATAATGTAGTTTGTCTTGCTTGGCAGATACTTTTGAAGGATGTTGGACTGTAAGGCT

GTCGATATCCTCCTCAGAGAACTTACTACAGTACATTGTATCTGTTCCCTTACCTACCTG

ACTCTTCCACTATTCAGTTTGTTCCTTAATGGTAGACCATGCCTGATTGGTGTTTTACAC

ATCTCCGGCTATGTCTGACACTTGTGGATGCTCAGAAAGTGGGGAAGGAAGGAAAGATAC

GATGGTAAAAGGCTTACACATGTCTTGACAAAAAAGTCCAGTTTGGCTCATTTGGCTGGA

GTCGTACTGCATGGCTGCCATTCTGCTCTGGCATCCTCAGACAAGCACACTGCCCATTAG

AGGAAAAAGTGTGAATATAAGTGTTGAGTCAGAATGCTGTAGACATTTAGTAACCTCCTT

CACAGGAAAAAAAAAAAGGTGGGGGGAATGACAGAAATCCAAAAACTAGTAGAGCTTCCA

CTTTTCATTTCAGAAGAAATCAGTTGCTCTCCTCTAAGGACCATTACTATTAACAAAACA

GAGACCTTAGAAGGAGGCATTGTTTATTTATTATATATTTTGTAATGTTATTACCAATCT

TGTTATACTCTTTCTTATACCCTACAATTGTTAGCAGAAATTATTTTAAATTAATAAGAT

CCTGCATGCTTTTCCTTTAAAAAAAAAAAAAAAAAGAAAGATCTCTGTGTAGAGTGTCCT

ATTCTGAGCCAGTCCTGAGAGGAAAGGAAGTATAATCAATTTGTTATTAACCAATGAAAG

AATTAAGTGAAAGATAAATCTCAGGAAGCAGAGGGAAGTAAACCTAATTTCTGACTAAGA

AAGCTAAATACTATGATAACTTATTCATTCCTTCTTTTGTTCAATTACATTATTTAATCA

TAAGTCCATGACGTGCCAGGCACTCAGGAAATAGTAAAAATTGGACATGTGATATTCTGC

CCTTGTGTAGCGCACACTAGAGTGGGAAAGAAAGTGCACTTTTAACTGGACAACTACCAA

CATGAAGAGGGGAGGAAGCAGGGGCTGGAAATGTCCACAGACTGTGCCAAAAAATGAAGC

CCATAATATTTGAAAGTCAGTTTCTTCCATCATTTTGTGTATTAAGGTTCTTTCTTCCCC

TGTTCTCCGCCTACCTGCTTGTCATCTTCACTCATCAGCTGACCACGTTGCCTCTTGTGG

TGTAAACTTGTACCAGTTTTACGGTCCCTCTGGCCAGTACACCCATGAATTTGATGGAGA

TGAGGAGTTCTACGTGGACCTGGAGAGGAAGGAGACTGCCTGGCGGTGGCCTGAGTTCAG

CAAATTTGGAGGTTTTGACCCGCAGGGTGCACTGAGAAACATGGCTGTGGCAAAACACAA

CTTGAACATCATGATTAAACGCTACAACTCTACCGCTGCTACCAATGGTATGCGTCCACC

ATTCTGCCTCTCTTTACTTAAGCTATCCCTCCATACCAGGGTTCATTATTTTCTTCCCAA

GAGGTCCCCAGATCTTCTTATGGCAATTGCTGAAATTTTATCATCTCCCATCTCTAAAAT

CACATATTCCCATGTAATACAAGGGTCTTTCCATTATGCATTCAGCAAATCCTTCTAGGA

GAGGTCTCATCAACCTCCTACTTTATTAAACATGCCCACAGAGAGAAGGGCACAGGAATA

AAGCAGAGGCAATGTGTCGTTGCTCCCAAGCAGAAGGTAAATAAGACCTCTTTGACTATC

AGGTGGTGAAATGCTGGTAGGAGGGCTCTTCCAGGATGTAATGCAGAACTTCAGGGCAGA

GCTATTCACACTTCACACCAGTGCTGTTTCCTCACCACAGAGGTTCCTGAGGTCACAGTG

TTTTCCAAGTCTCCCGTGACACTGGGTCAGCCCAACACCCTCATTTGTCTTGTGGACAAC

ATCTTTCCTCCTGTGGTCAACATCACATGGCTGAGCAATGGGCAGTCAGTCACAGAAGGT

GTTTCTGAGACCAGCTTCCTCTCCAAGAGTGATCATTCCTTCTTCAAGATCAGTTACCTC

ACCTTCCTCCCTTCTGCTGATGAGATTTATGACTGCAAGGTGGAGCACTGGGGCCTGGAC

CAGCCTCTTCTGAAACACTGGGGTAAGGATGAGTTTCATCATTTTTTGATTCTTTCTTGT

CTGTCAAGTTCAGAACTTCCTGCCTTTTACTCCTATATCCAAAAACTTGTTTTCCACACT

TCATGGGTTTCTTTTCTGTCTCTCTTTTTTTTTTTTGAAAGAATAAAGCAAAAAAAGCAG

AGATTTATTGAAAATGAAAGTACACTCTACAGGATGGGAGTGGGCCTGCCACTTCATGGT

TTTCTAATGATAGACTTCACTCTTCTCCCTAAGCTGGGGGCCTTGAGTCTTTGCAGAGCC

AACCCTCTACCCCATCCCATCCCACACACATGCACATGAGCAAACTCTGCATTCTGACCT

CAACAACTTCACTTCCACAGAGCCTGAGATTCCAGCCCCTATGTCAGAGCTCACAGAGAC

TGTGGTCTGCGCCCTGGGGTTGTCTGTGGGCCTCGTGGGCATTGTGGTGGGCACTGTCTT

CATCATCCAAGGCCTGCGTTCAGTTGGTGCTTCCAGACACCAAGGGCCATTGTGAATCCC

ATCCTGGAAGGGAAGGTAAGATTGAGACTGGTTACAGTTGAAGCGGCAGTATGAAAGGAA

GGAAAGTGGGAGGGCGTTGTGGACATGAATGTGGTTGAAAGTTGTAGGGGAATTGGGAAG

TGGCATGATGATGACACAGGAGGCCCCTCGGACCCATCGATCTCATGTCTGCCCTGTTGC

AGGTGCATCGCCATCTACAGGAGCAGAAGAGTGGACTTGCTACATGACCTAGCACTATTC

TCTGGCCCGATTTATCATATCCCTTTTCTCCTCCAAATATTTCTCCTCTCACCTTTTCTC

TGGGACTTAAGCTGCTATATCCCCTCAGAGCTCACAAATGTCTTTACATTCTTTCCCTGA

CCTCCTGATTTTTTTTTTCTTTTCTCAAATGTTACCTACAAAGACATGCCTGGGGTAAGC

CACCCGGCTAC

>HLA:HLA00622 DQB1*02:01:01:01

TTCTAAGAACTTTGCTCTTTTCACCAAAACTTAAGGCTCCTCAGGGTGTGTCTAAGACAA

CAGCAGTAAAAATGTCTATGACAGCAATTTTCTCTCCCCTGAAATATGATCCCCACTTAA

TTTGCCCTATTGAAAGAATCCCAAGTATAAGAACAACTGGTTTTTAATCAATATTACAAA

GATGTTTACTGTTGAATCGCATTTTTCTTTGGCTTCTTAAAATCCCTTAGGCATTCAATC

TTCAGCTCTTCCATAATTGAGAGGAAATTTTCACCTCAAATGTTCATCCAGTGCAATTGA

AAGACGTCACAGTGCCAGGCACTGGATTCAGAACCTTCACAAAAAAAAAATCTGCCCAGA

GACAGATGAGGTCCTTCAGCTCCAGTGCTGATTGGTTCCTTTCCAAGGGACCATCCAATC

CTACCACGCATGGAAACATCCACAGATTTTTATTCTTTCTGCCAGGTACATCAGATCCAT

CAGGTCCGAGCTGTGTTGACTACCACTTTTCCCTTCGTCTCAATTATGTCTTGGAAAAAG

GCTTTGCGGATCCCCGGAGGCCTTCGGGCAGCAACTGTGACCTTGATGCTGTCGATGCTG

AGCACCCCAGTGGCTGAGGGCAGAGACTCTCCCGGTAAGTGCAGGGCAGCTGCTCTCCAG

AGCCGCTACTCTGGGAACAGGCTCTCCTTGGGCTGGGGTACGGGGATGGTGATCTCCATA

ATCTCGGACACAATCTTTTATCAACATTTCCTCTGTTTTGGGAAAGAGAGCTATGTTGCA

TTTCCATTTATCTTTTAATGATGAAGTGAGGACAATCCAATCCCATCCTACAGGCTTAAG

CCTGGAAGAGGAGGAGAGAGGAGAGAAAAGAGGAGACAAAGTGTTCATTTACTACCAGTG

ATAGGACAAAGTGAGCATGGGGTTATTTTTGAAGATATGAATTTCTCCAAAGACACAGCA

GGATTTGCCATTTAGGCGTGTCCCAAGACTTGCCTGGACTAAATATTATGATTTCCTGCA

TTGGGAAATGCAAGGCAGCAATGGTGTCTGTAGTCTCCGTATTTGGGGAAAAGTTGTCTG

TATTCCTGACCCAGTGGAGCGTTTGTGGAGGCAAAATCTTGGTACTGAGGGAAGCTGACT

GGCTGACCACAGAAAGAGAGCCTTCAGGTTTCACTGATTTATGGGCAAATGGTGACCTGA

GTGGGATTCAGATACCCGAGTTGATGATGGACTAAATTTAGTAGAAAGGAGGATGTAAAG

AAGGGAAATAACACATACTGTGAAACCACTCATTTCAGACACAGAACAATACTTTACATA

AATTCTCTCTCACTCCTTCTAACATCCTGTGTGTAGATATCATGATTTTCTTTTACACAA

TTATACTTGTGATATGGATATTCTGTTACATAAACTGCCCGGGCTGGTGACTGCCACAGT

TTAATGGGAATCTAGTTTATCAAATTCAAAAGCTTGTGCTCTTTCGGTGAATAAATGTTT

CTTTCTAGGACTCAGAGATCTAGGACTCCCTTCTTTCTAACACAGACGTGAGTGAACCTC

ACAGGGCACTTGGGAGGGTAAATCCAGGCATGGGAAGGAAGGTATTTTACCCAGGGACCA

AGAGAATAGGCGTATCGGAAGAGGACAGGTTTAATTCCTGGACCTGTCTCGTCATTCCCT

TGAACTGTCAGGTTTATGTGGATAACTTTATCTCTGAGGTACCCAGGAGCTCCATGGAAA

ATGAGATTTCATGCGAGAACGCCCTGATCCCTCTAAGTGCAGAGGTCCATGTAAAATCAG

CCCGACTGCCTCTTCACTTGGTTCACAGGCCGAGACAGGGACAGGGCTTTCCTCCCTTTC

CTGCCTGTAGGAAGGCGGATTCCCGAAGACCCCCGAGAGGGCGGGCAGGGCTGGGCAGAG

CCGCCGGGAGGATCCCAGGTCTGCAGCGCGAGGCACGGGCCGGCGGGAACTTGTGGTCGC

GCGGGCTGTTCCACAGCTCCGGGCCGGGTCAGGGTGGCGGCTGCGGGGGCGGACGGGCTG

GGCCGCACTGACTGGCCGGTGATTCCTCGCAGAGGATTTCGTGTACCAGTTTAAGGGCAT

GTGCTACTTCACCAACGGGACAGAGCGCGTGCGTCTTGTGAGCAGAAGCATCTATAACCG

AGAAGAGATCGTGCGCTTCGACAGCGACGTGGGGGAGTTCCGGGCGGTGACGCTGCTGGG

GCTGCCTGCCGCCGAGTACTGGAACAGCCAGAAGGACATCCTGGAGAGGAAACGGGCGGC

GGTGGACAGGGTGTGCAGACACAACTACCAGTTGGAGCTCCGCACGACCTTGCAGCGGCG

AGGTGAGCGGCGTCGCCCCTCTGCGAGGCCCACCCTTGGCCCCAAGTCTCTGCGCCAGGA

GGGGCGAAGGGTCGTGGCCTCTGGAACCTGAGCCCCGTTTGTTCCACCCCAGAGGACAGG

AGGCAGCGGCGAGAGTGGTGGGGGCAGGTGCATCGGAGGTGCGGGGACCTAGGGCAGAGC

AGGGGGACAGGCAGAGTTGGCCAGGCTGCCTAGTGTCGCCCCAGCCTACCCGTTCGTCGG

CCTTGTCCTCTGCTCTGCATGTTCTTGCCTCGTGCCTTATGCATTTGCCTCCTTTTGCCT

TACCTTTGCTAAGCAGCTCTCTCTGCTCAGAATGCCCGCCCTCTTCCCCTGCCCGCCCGC

CCGCCCCACTAGCACTGCCCCACCCAGCAAGGCCCACGTGCACAGCTCTTGCAGCAGGAA

GCTTCAGGCTTAGCCTGGTGGAGTTAGGGCTGTTCCACAACTGCGCGCAGGACATTCAGC

AATTACAGTTGTGAAATAAGATATTTTAACTTTTGGCTTCAAATCATTATTCATCGTAAT

TCTGTTTTCTTAAATGGCTCTCATTCATGGCAGAGATCTTTGAGGTGAGGGTGTTTTAAT

CATTGCATGCCTAGTACCTGACACATTGACTGGTATGTGGTGTGAGCTCAATGATCTTCT

GTTAAATTAATGAATAAATGTACTCAGCTGCCCATCCACTTAGGCTCAAGAAAAAAAAAG

AGGTAAACAGAGCCTTAAAAATGGACTTTATTAATTATTTTCTATAATTTTGCTTAATGC

TTTAAAGTAAACTCTTATTGACTTGGATCTTAATAGAGTTTGTGAATACAAAATCTGAGG

AAAAAAGTTTTTGCTAAAAATAAAAACAACGCTTGAAAGATATTGTAAGGCAGTTTAAAT

TTCTTTTCTTTTCTTTTTTTTTTTTTTTTGAGACGGATTCTCACTCTGTCGCCCAGGCCG

GAGTGCAGTGGCGCGATCTCGGCTCACTGCAAGCTCCGCCTCCCGGGTTCACGCCATTCT

CCTGCCTCAGCCTCCTGAGTAGGTGGGATTACAGGCGCGTGCCACCACGCCCGGCTAATT

TTTTTGTATTTTTAGTAGAGGCGGGGTTTCACCGTGTTAGCCAGGATGGTCTGGATCTCC

TGACCTCATGATCCGCCCGCCTCGGCCTCCCAAAGTGCTGGGATTACAGGTGTGAGCCAC

AGTGCCCGGCCGGCACTTTTAATTTCTTAGAAAAGCTGAACAAATGGCACAATGCAAAGA

GCAAAAGTTTTGGAATAAATAGATTGAAGCCATTAAATTATTGGATAAAAATAGTTTCGG

GTTGCTTTTGGCCTAGGTTCTCCCCTCCCCCCATGACTATCCACTTCAGGAATAAACATT

CTGAAAGTCAATTTTACCCATTTAGTGAGCATTTATTTCTAGACAGTTGCCTTATCAAAT

ACCATCTATGTTACGTCATTTAATCTCACAGTTACTTGTGCATCAGAGATTAGCATCACC

ACTTTATATATTGGTACATGATAAACACTTTATTGGTCATGGATGGGGAGATGGTCACTG

TAGGCTAATATTGGTACATGATAAACACTTTAAGTAATCAGCCCATAATTGCTCACCAAG

ACCTTAAGCCTCCCAAAGTACACAACATTCTTTGTGTTCTTCACTACACATCCATAGAGT

CTAAGGGACGTAAAGCCTCGTTAAAGCCAGTTTTGACCAGAAGCAGCAATGAGTCTATTC

CTGTGTGTTTTCCATGTTAATGGGACAAAATGATACTTTCAAGGCATTGAAAATTCATGA

TTAATCAATCCCTAGTCTGACCCCAGTGTTATCTATGCAGGTTTGCAAAACCTTTAGTTT

ACTTAATACTCCCTTGCCTTCTTTTGATTCACATCCTAATGCCAGCAAATACTTATGTTT

TTGCTATTTCAGTTCCATTTCCATAAAATTTATTTTATCATCTTTTCTCATAAATTTATG

CCCTCTATTTTTACTCCCAATCTGTTTAAGATGAACAAATCTTATAAGGCCACATAGCTG

ACTGTTATTTCTGTTGGACTCCAGGAAGGAGAACCTAAAGAAAAGTTCAAGTCCAAGCAG

AAACCGTGATTTCTTCCAGATGATGGCTCATGAGTGCCATTTAATTGGGGTGCCACCTGG

TGACCTCAGCAAATCCCAGCTATATTTATGTGTTCACATTACAGGATCATTAACCCAGAC

CGACCACTGCACAGATCTCAGAATATTTTCTATGGAGAACATACATAATAATGCCTGATT

TCAGAAGAAGAAAGTAATTCTCAATAGCAAGGGGATGGAGTAGGGTAGACAGCTGTAATT

AAACTCACTTGTGTGATAAAAAGAAATTAAGGAAAAAAGAAAATGAGAGAACATATTACT

AAATAAAGAAAGCATACATTAAATATTTACTATAGTTTCACACTAAGAGAATAAAGGAAA

TGCAATAAAGTGGCCTGAAAGGTAAAGGATGAGATGTGTAAAGGGGTGTAGTATTTTTAC

TATGAGCAGCAATCTGAGAAGATAAAGGAATCGAGTTACGGGCAAACATGATGTTTGATC

AGTGTTATTTGTTTTCAAGGCCTGCCTAAATTTTTTTCAAATATTACAAACTTTTGAAAT

AACATTCTTTTTGTTTTTTGCTGTCTGTTACTAGGTTGCACATTTTATAAAGGCAGGGAC

CATGGTATGTTGTTTGTCTTTGGATTCTCAGTGATTGTTATATTTATATTTGTTGAAGGA

ACCTTAATCCAAGACTTGGACTCCAAGTATCTTTCCACTCTGGTTCCAAGGAGGGACCTT

CCTCACAGCAGGCATGCTGTGTGGTCTCACATCTCACTCCTATATCTTTCCCTGTCTGTT

ACTGCCCTCAGTGGAGCCCACAGTGACCATCTCCCCATCCAGGACAGAGGCCCTCAACCA

CCACAACCTGCTGGTCTGCTCGGTGACAGATTTCTATCCAGCCCAGATCAAAGTCCGGTG

GTTTCGGAATGACCAGGAGGAGACAGCTGGCGTTGTGTCCACCCCCCTTATTAGGAATGG

TGACTGGACCTTCCAGATCCTGGTGATGCTGGAAATGACTCCCCAGCGTGGAGACGTCTA

CACCTGCCACGTGGAGCACCCCAGCCTCCAGAGCCCCATCACCGTGGAGTGGCGTAAGGG

GATATTGAGTTTCTGTTACTGTGGGCCCCACAAGACAAAGGACAGAGCTCCTTCTGACCC

ATCCCTTCCCATCTCTTATCCCTGATGTCACTGCTGAGCTGGGAATCACAGGAGACTAGA

GCACCTCTAGTTCCATGGCGAGTGCATCAGAAGAATCCTGATCTCATCACCTTTCCAGAT

GCTAGGGAAATTACTCTACATACTGTTGCTCTGGATCCCAGTCCTGATTGCTCTGAGGAA

CTGATTATTAGGGCTGGTGACTGGGATCTTAGGGTCTAAGTTTATGGATGAGTTCCTGAG

GAGTGGAGATCTGCTTCCCCACTCTGTCACCTACTCACTGTATCCAAGGACCTATTGGCT

GGCCTTTCCCTCCCTTAGGGGTGGTCTGAATGGAGAACTAGGTTCCTTTGATGCCTTCAC

CTCCTGCATCTCAGACTGGACTTCAGCTCCTCATCAGGGAAACTATGGGGTATGGGGACA

AACACTGACACTCAGGCTCTGCTTCTCAGGGGCTCAATCTGAATCTGCCCAGAGCAAGAT

GCTGAGTGGCATTGGAGGCTTCGTGCTGGGGCTGATCTTCCTCGGGCTGGGCCTTATCAT

CCATCACAGGAGTCAGAAAGGTGAGGAACCCCAGGGGAAAAGGGGAAGATGGCCTGTGAC

CCAGACCCTCTGTTCAGAGAGGTCCTGTCTCTAGATGTAGCTCTTTCCTCCTGACCCTGA

GAGGAAGAAAGCTGAGCTGGAAGTGGAAGGAGACAGGACAAGGTTGGAGGAGGCATTGGA

ATCTGATTTTACTAGCTGAAGGGTAGCCCTGTCACAGAGCTGACTGATAGAGCTTATTCC

AGGGCATCCTTACCATTCATCATTGTCTCACTGGCTCCTTTCCAAAAACTTCCTCCATTA

AGAGGGTCAGAGCCTCGGCCTCCTTGCCTTCTAGTGACAATTTCCTTTATTTTAGGGGAT

TTTAAATTAGGGTGCTTAAGGACTTGAAGAACATGAATGGGAAGAGAATATAACTCTAAT

TAAGTCACATGTGTCATTTTCCTTTGGGGTGAGAGAGTGGCTGTTCATGTAATGAGACCT

TTCTCTGCATAACTTCCTTTTGTAAGACCTCAAGGGCCTCCACCAGCAGGTAATATTTCA

GCCGTGATCCAGTGTGGGGAGGGCACAGGTGTAAGAGGGAAGAGCATGAGCTGAGTGTAC

CTGACAGTAGTGGTCTCTGTTCATGGTATATTTGCTGCTATGAGGATCAAGACTTAGGGG

TGAAGTTTGCCAGTTTCTAGGAATCTCCAGAGGTTGTTCCCCAGAACCAAGCCTTAACTT

TGGTGGTATCTTCCTGTTAAATGTGGAGCCAGAACCACGGCTTAAATGTTAGACACTAGG

ATGATGCCCACTTTGTGCCACATGATGGTGGCTACTGCCTGTAGGCATTTTCCAGTGACT

GAAAGAGGCTGCTAGTGGTAGGGATGAGGTATCATCCAATTTCCTAAAAAGATTGAACCC

TTCATATTCACCAGAAGAGTAACAGCTGTTCCCCCACCTCCCACACATCTGCATCAAGCT

GAAGTTCTGTGTCTTCATGAGCTGATTTCACCTTTGCACAGATCTTGGGGGAGGTGATGA

CAATACACTCTGGACCTCAGCTTTGTCTGTCTGAAGCTGCAGGAGGCCCCTGAGGGGTGG

GGAAGATGGCAGGCCCACCAGCGTACCCTGTGCTGATCATCCCTCTTCTCTCTTCTTCAG

GGCTCCTGCACTGACTCCTGAGACTATTTTAACTGGGATTGGTTATCACTTTTCTGTAAC

GCCTGCTTGTCCCTGCCCAGAATTCCCAGCTGTCTGTGTCAGCCTGTCCCCCTGAGATCA

GAGTCCTACAGTGGCTGTCACGCAGCCACCAGGTCATCTCCTTTCATCCCCACCTTGAGG

CGGATGGCTGTGACCCTACTTCCTGCACTGACCCACAGCC

>HLA:HLA00629 DQB1*03:03:02:01

TTCTAAGAACTTTGCTCTTCTCACCAAAACTTAAGGCTCCTCAGGGTGTGTCTAAGACAA

CAGCAGTAAAAATGTCTATGACAGCAATTTTCTCTCCCCTGAAATATGATCCCCACTTAA

TTTGCCCTACTGAAAGAATCCCAAGTATAAAAACAACCAGTTTTTAATCAATATTACAAA

GATGTTTACTGTTGAATCGCATTTTTCTTTGGCTTCTTAAAATCCCTTAGGCATTCAATC

TTCAGCTCTTCTATAATTGAGAGGAAGTTTTCACCTCAAATGTTCATCCAGTGCAATTTG

AAGACGTCACAGTGCCAGGGACTGGATTGAGAACCTTCACAAAAAAAAAATGTCTGCCTA

GAGACAGATTAGGTCCTTCAGCTCCAGTGCTGATTGGTTCCTTTCCAAAGGACCATCCAA

TCCTGCCACGCAGGGAAACATCCACAGGTTTTTATTCTTTCTGCCAGGTACATCAGATCC

ATCAGGTCCAAGCTGTGTTGACTACCACTACTTTTCCCTTCGTCTCAATTATGTCTTGGA

AGAAGGCTTTGCGGATCCCTGGAGGCCTTCGGGTAGCAACTGTGACCTTGATGCTGGCGA

TGCTGAGCACCCCGGTGGCTGAGGGCAGAGACTCTCCCGGTAAGTGCAGGGCCACTGCTC

TCCAGAGCCGCCACTCTGGGAACAGGCTCTCCTTGGGCTGGGGTAGGGGGATGGTGATCT

CCATGATCTCGGACACAATCTTTCATCAACATTTCCTCTCTTTGGGGAAAGAGAACGATG

TTGCATTCCCATTTATCTTTTAGTGATGAGGTGAGCACAGTCGGATCCCCATCCTACAGG

CTTAAGCCTGGAAGAGAAGGAGAGAGGAGAGGAAAGAGGAGACAAAGTGTACATTTACTA

CCAGTGACAGGACAAAGTGAGCATGGGGTTATTTTTGAAGATACGAATTTCTCCAGAGAC

ACAGCAGGATTTGTCATTTAGGCGTGCCCCAAGACTTTGCCTGGACTAAATATTATGGGA

TCCTGCATTGGGAAATGTAAGGCAGCAATGGTGTCTGTAGTCTCCGTATTTGAGGAAAAG

TTGTCTGTATTCCTGACTGACTGGAACGTTTGTGGAGGCAAAATCTTGGTACTGAAGGAA

GCTGACTGGATGACCACAGACAGGGAGTCTTCAGGTTTCACTGATTTATGGGCAAATGGT

GACTTGAGTGGGATTCAGAGACCCGAGTTGGTGGACTGAATTTAGCAGAAAGGAGGATGT

AAAGAAGGGAAATAACATATATTGTGAAACCACTCATTTCAGACACAGCACAATACTTTA

CATAAATTCTCTCTCACTCCTTCTAACATCCTGTGTGTAGATATCATGATTTTCTTTTAC

ACAATTATACTTGTGATATGGATATTCTGTTACATACCTGCCCTAGCTGGTGACTGCCAC

AGCTTAATTGGAATCTAGTTTATCAAATTCAAAAGCTTGTGCTCTTTCCATGAATAAATG

TTTCTTTCTAGGACTCGGAGGTGTAGGTCCTTTCTAACATAAAAGTGAGTGAACCTCACA

TGGCATTCGGAAGGGTAAATCCAGGCATGGGAAGGGAGGTATTTTACCGAGGGACCAAGA

GAATACGCATATCAGAACGAGGACAGGCTTAATTTCTGGACCCGTCTCATCATTCCCTTG

AACTCACAGGTTTATGTGGATAATTTTATCTCTGAGGTTTCCAGGAGCTCAATGGAAAAT

GGGATTTCATGCGAGAGCGCCCTGATTCCCTCTAAGTGCAGAGGTCTATGTAAAATCAGC

CCGACTGCCTCTTCCCTCGGTTCACAGGCTCCGGCAGGGACAGGGCTTTCCGCCCTTTCC

TGCCTGCAGGAAGGCGGATTCCCGAAGCCCCCAGAGAGGGCGGGCAGGGCTGGGCAGAGC

CGCCGGGCGGATCACAAGTCTGGAGCGCCAGGCACGGGCGGGCGGGAACTGGAGGTCGCG

CGGGCGGTTCCACAGCTCCGGGCCGGGTCAGGGCGGCGGCTGCGGGGGCGGCCGGGCTGG

GGCCGGGCCGGGGCCTGACTGACCGGCCGGTGATTCCCCGCAGAGGATTTCGTGTACCAG

TTTAAGGGCATGTGCTACTTCACCAACGGGACGGAGCGCGTGCGTCTTGTGACCAGATAC

ATCTATAACCGAGAGGAGTACGCACGCTTCGACAGCGACGTGGGGGTGTATCGGGCGGTG

ACGCCGCTGGGGCCGCCTGACGCCGAGTACTGGAACAGCCAGAAGGAAGTCCTGGAGAGG

ACCCGGGCGGAGTTGGACACGGTGTGCAGACACAACTACCAGTTGGAGCTCCGCACGACC

TTGCAGCGGCGAGGTGAGCGTCGTCGCCCGTCTGCGAGGCCCATCCTTGGCAGGGGCCCA

GAGTCTCTGCGCCAGGAGGGGCGAAGGGGGCGCGGCCTCTGGAACCTGAGCCTTGTTCGT

TCCACCCCGGCTGACAGGAGGAGGCGGTGGGGGTGCTGGGGGCTGGTGCATCGGAGGGGC

AGGGACCTAGGGCAGAGCAGGGGGACAGGCAGAGTTGGTCAAGCTGCCTAGTTTCGCCCC

ATCCTCCCCGTCCGTCGGCCTCGCCCTCTGCTCTGCACGTTCTTGCCTCGTGCCTTATGC

GTTTGCCTCCTCGTGCCTTACCTTTACTAAACAGTTCTCTCTGCCCCCAATTTCCGCCCT

CTTCCCCTGCCCGCCCGCCCGGCTAGCACTGCCGCACCCGGCAAGGTCCACCTACACAGC

TCATGCAGTGGGAAGCTTCAGACTTGGCCTGATGGAGTTAGGGCTGCCCCACAACTGCGC

GCAGGGCATCCAGCAATTACAGTTGTCAAATAAGATATTTTGACTTTTGACTTCAAATTA

TTATTCATCGTAATTCTGTTTTCTTAAATGGCTCTCATTCATGGCGGAGCTCTTTGAGAT

GAGAGTGTTTTAATCATTGCATGCCTGGTATCTGACACATTGACTGGCATGTGGTATGAG

CTCAATGATCTTCTGTTAAATTAATGAATAAATGTGCTCAGCTGCCAATCCACTTAGGCT

CAAGGGAAAGCAGAGGATAAATAGAGCCTTAAAAATGGACTTTTATTAATTATTTTCTGT

CATTTTGCTTAATTCTTTAAAGTAAACTCTTATTGACTTGGATCTTAATAGAGTTTGTGA

ATACAAAGTCTGAGGAAAAAAGTGTTTGCTAAAAATAAAAACAACACTTGAATGATGTTT

GTAAGGCAGTTTTAATTTCTTAGAAAAGCTGAACAAATGGCACAATGCAAAGAGCAGAAG

TTTTGGAATAAATAGATTGAAGCAATTAAATTATTGGATAAAAATAGTTTCAGGTTGCTT

TTGGCTTAGGTTCTCCCCTCCCCCCATCACTATCCACTTCAGGAATAAACATTCTGAAAG

TCAATTTTACCCATTTAGTGAGCACTTATTTCTAGACAGTTACCTTATCAAATACCGTCT

ATGTTACGTCATTTAATCTCACAGTTGCCTGTGCATTAGAGATTAGCATCACCACTTTAT

ATATCCTAATATTAGTACATGACAAACACTTTAAGTAATCAGCCCACAAGTACTCACCAA

GACCTTAAGCCTCCCAAAGTACACAATATTCTTTATGTTCTTCACTACACATCTATAGAG

TCAAAGGGACATAAAGCCTTGTTAAAGCCAGTTTTGACTAGAAGCAGCAATGAGTCTCTT

CCTGTTTGATCTCCATGTTAATGGGACAAAATGATACTTTCAAGGCATTGAAAATTTATG

ATTAATCAATCCCTAGTCTGACTCCAGTGTTACCTATGCAGATTCACAAAACTTTTAGTT

TACTTTAAACTCCCTTGCCTTCTTTTGACTCACATCGTAGTGCCAGCAAGTACTTACATT

TTTGCTATTTTGGTTCTATTTCCATAAAATTTATTTTATCATCTTTTCTCATAAGTTTGT

GCCCTCTATTTTACTCCCAGTCTGTGTAAGATGAACAAATCTTATAAAGCCACATAGCTG

ACTGTGATTTCAGGTGGACTCCAGGAAGGAGAACCAAAGAAAAGTTCAAGTCCAAGCAGA

AACCGTGATTCCTTCCGGATGATGGCTCAAGAGTGCCTTTTAATTGGGGTGCAACCTGCT

GACCTCAGCAAATCTCAGCTATATTTATATGTTCACATTACAGGCTCATTAACCTAGGCT

GATCTCTGCAAGGATCTCAGAATATTTTCTACAGATAACATACATGATAATATCTGATTT

CAGGACAAAAAAGTAATTCTCAATAGCAAGGGAATGGAGTAGGGTAGACAGCTAGTAATT

AAACTCACTTGTATGTTAAAAATAAATTAAGGAAAAAAAGAAAATGAGAGAACATATTAC

TAAATAAAGAAAGCATACATTAAATATTTACTATAGTTTCACACTAAGAGAATAAAGGAA

ATGCAATAAAGTGGCCTGAAAGATAAAGGATGAGATGTGTAAAGAGACAGGGAAAGATGT

GTCATTTTTTTACTATGAGCAGCAGTCTGAGAAGATAAAGGAATCGAGTTATGGGCAAAC

ATGATGTTTGATCAGTGTTATTTGTTTTGAAGGCCTGCCTACTTTTGTTTCAAATATTAC

AGACTTTTGAAATCACATTCTTTTTGTTTTTTGCTGTCTGTTACTAGATCGCACATTCTG

TAAAGGCAGGGACCATGGTATGTTGTTTATCTTTGGATTCTCAGTGATTGTCATATTTAT

ATTTGTTGAATGAATCTTAATCCAAGACTTGGACTCCAGGTATCTTTCCACTCTGGTTCC

AAGGAGGGACCATTCCCAATGGTAGACGTGCTGTGTGGTCTCACATCTCACTCCTGTCTT

TTCCTGTCTGTTACTGCCCTCAGTGGAGCCCACAGTGACCATCTCCCCATCCAGGACAGA

GGCCCTCAACCACCACAACCTGCTGGTCTGCTCAGTGACAGATTTCTATCCAGCCCAGAT

CAAAGTCCGGTGGTTTCGGAATGACCAGGAGGAGACAACTGGCGTTGTGTCCACCCCCCT

TATTAGGAACGGTGACTGGACCTTCCAGATCCTGGTGATGCTGGAAATGACTCCCCAGCG

TGGAGACGTCTACACCTGCCACGTGGAGCACCCCAGCCTCCAGAACCCCATCATCGTGGA

GTGGCGTAAGGGGATATTGAGTTTCTGTTACTATGGGCCCCACAAGACAAAGGGCAGAGC

TCCTTCTGACCCATTCCTTCCCATCTCTTATCCCTGATGTCACTACTGAGCTGGGGATCA

CAGGAGACTAGAGCACCTCTTGCTCCATGGCAAGTGCATCAGAAGAATCCTGATCTCATC

ACCTTTCCAGATGCTAGGGAAATTATTCTACGTACTGTTTCTCCAGATCCCAGTCCTGAT

AGCTCGGAGGGACTTATTATTAGGGCTGGTGACTGGGATCTTAGGGTTTAAGGTATGGAT

GAGTTCCTGAGGAGTGGAGATCTGCTTCCCCGCTCTCTCACCTACTCACTGTACCGAAGG

ACCTATTGGCTGGCTTTCCCCTCCCTTAGGGGTGGTCTGAATGGAGGACTAGGTTCCTTT

GACACTTTCACCTCCTGCATCTCAGACTGGACTTCAGCTCCTCAGCAGGGATGCTATGGG

GTGTAGGGACAAACACTGACACTCAGGCTCTGCTTCTTAGGGGCTCAGTCTGAATCTGCC

CAGAGCAAGATGCTGAGTGGCATTGGAGGCTTCGTGCTGGGGCTGATCTTCCTCGGGCTG

GGCCTTATTATCCATCACAGGAGTCAGAAAGGTGAGGAACCCCAAGGGAAAAGGGGAAGA

TGGGCTGTGACCCAGACCCTCTGTTCAGGGAGGTCCTGTCTCTAGATGTGGCTCTTTCCT

CCTGACCCTGAGAGGAAGAAAACTGAGCTGGAGGTGGGAGGAGACAGGACAAGATTGGAG

GAGGCACTGGAATCTGATTTTACTAGTTGAAAGGTAGCCCTGTCACACAGGTGACTGATA

GAGCTTATTCCAGGATGTACTTACCATTCATCGTCTCATTGGCTCCTTTCCAAAAGCTTC

CTCCATTAAGAGGGTCAGAGCCTTGGCCTCCTTGCCTTCTAGTGACAATTTTCTTTGTTT

TAGGGGATTTTAAATTAGGGTACTTAAGGCCTTGAAGAACATGAGTGGTAAGAGAATATA

ACTCTAATTAAGTCACATGTGTCATTTTCCTTTGGGGTGAGAGAGTGGCTGTTTGTGTAA

TGAGACCTTTCTCTGCATAACTTCCTTTTGTAAGACCTCAAGGGCCTCCACCAGCAGGTG

ATATTTCAGCCATGAGCCAGTGTGGGGGGGCACAGGTGTAAGAGGGAAGAGCATGAGCTG

AATGCACCTGACCACAATGGTCTCTGTTCATGGTATATTTGCTGCTATGAGGATCAAGAC

TTAGGGTCGAAGTTTGCCAGTTTCTAGGAATCTCCAGAGGTTGTTCCCCAGAACCAAGCC

TTAACTTTGGTGGTATCTTCTTGTGAAATGTGAAGCCAGAACCACAGCTTAAATGTTAGA

CAAGAGGATGATGCCCACTTTGTGCCACATGTTGGTGGCTACTGCCTGTAGGCATTTTCC

AGTGACTGAAAGAGGCTGCTAGTGGTAGGGATGAGGTATCATCCAATTTTCTAAAAAGAT

TGAACCCTTCATATTCCCCAGAAGAGTAACAGCTGTTCCACCACTTCCCACACATCTGCA

TCAAGCTGAAGTTCTGTGTCCTCATGAGCTGATTTCACCTTTGCACAGATCTTGCGGGAG

GTGACGATAATACATTCTGGACCTCAGCTTTCTCTGTCTGAAGCTGCAGGGGGCCCCTGA

GGGGTGGGGGAGATTGCAGGCCCACCAGCGTACCCTGTGCTGATCATCCCTCTTCTCTCT

TCTTCAGGGCTCCTGCACTGACTCCTGAGACTATTTTAACTGGGATTGGTTATCACTTTT

CTGTAACGCCTGCTTGTCCCTGCCCAGAATTCCCAGCTGCCTGTGTCAGCCTGTCCCCCG

AGATCAGAGTCCTACAGTGGCTGTCACGCAGCCACCAGGTCATCTCCTTTCATCCCCACC

TCAAGGCTGATGGCTGTGACCCTGCTTCCTGCACTGACCCAGAGCC

>HLA:HLA00631 DQB1*03:05:01

TTCTAAGAACTTTGCTCTTCTCACCAAAACTTAAGGCTCCTCAGGGTGTGTCTAAGACAA

CAGCAGTAAAAATGTCTATGACAGCAATTTTCTCTCCCCTGAAATATGATCCCCACTTAA

TTTGCCCTACTGAAAGAATCCCAAGTATAAAAACAACCAGTTTTTAATCAATATTACAAA

GATGTTTACTGTTGAATCGCATTTTTCTTTGGCTTCTTAAAATCCCTTAGGCATTCAATC

TTCAGCTCTTCTATAATTGAGAGGAAGTTTTCACCTCAAATGTTCATCCAGTGCAATTTG

AAGACGTCACAGTGCCAGGGACTGGATTGAGAACCTTCACAAAAAAAAATGTCTGCCTAG

AGACAGATTAGGTCCTTCAGCTCCAGTGCTGATTGGTTCCTTTCCAAAGGACCATCCAAT

CCTGCCACGCAGGGAAACATCCACAGGTTTTTATTCTTTCTGCCAGGTACATCAGATCCA

TCAGGTCCAAGCTGTGTTGACTACCACTACTTTTCCCTTCGTCTCAATTATGTCTTGGAA

GAAGGCTTTGCGGATCCCTGGAGGCCTTCGGGTAGCAACTGTGACCTTGATGCTGGCGAT

GCTGAGCACCCCGGTGGCTGAGGGCAGAGACTCTCCCGGTAAGTGCAGGGCCACTGCTCT

CCAGAGCCGCCACTCTGGGAACAGGCTCTCCTTGGGCTGGGGTAGGGGGATGGTGATCTC

CATGATCTCGGACACAATCTTTCATCAACATTTCCTCTCTTTGGGGAAAGAGAACGATGT

TGCATTCCCATTTATCTTTTAGTGATGAGGTGAGCACAGTCGGATCCCCATCCTACAGGC

TTAAGCCTGGAAGAGAAGGAGAGAGGAGAGGAAAGAGGAGACAAAGTGTACATTTACTAC

CAGTGACAGGACAAAGTGAGCATGGGGTTATTTTTGAAGATACGAATTTCTCCAGAGACA

CAGCAGGATTTGTCATTTAGGCGTGCCCCAAGACTTTGCCTGGACTAAATATTATGGGAT

CCTGCATTGGGAAATGTAAGGCAGCAATGGTGTCTGTAGTCTCCGTATTTGAGGAAAAGT

TGTCTGTATTCCTGACTGACTGGAGCGTTTGTGGAGGCAAAATCTTGGTACTGAAGGAAG

CTGACTGGATGACCACAGACAGGGAGTCTTCAGGTTTCACTGATTTATGGGCAAATGGTG

ACTTGAGTGGGATTCAGAGACCCGAGTTGGTGGACTGAATTTAGCAGAAAGGAGGATGTA

AAGAAGGGAAATAACATATATTGTGAAACCACTCATTTCAGACACAGCACAATACTTTAC

ATAAATTCTCTCTCACTCCTTCTAACATCCTGTGTGTAGATATCATGATTTTCTTTTACA

CAATTATACTTGTGATATGGATATTCTGTTACATACCTGCCCTAGCTGGTGACTGCCACA

GCTTAATTGGAATCTAGTTTATCAAATTCAAAAGCTTGTGCTCTTTCCATGAATAAATGT

TTCTTTCTAGGACTCGGAGGTGTAGGTCCTTTCTAACATAAAAGTGAGTGAACCTCACAT

GGCATTCGGAAGGGTAAATCCAGGCATGGGAAGGGAGGTATTTTACCGAGGGACCAAGAG

AATACGCATATCAGAACGAGGACAGGCTTAATTTCTGGACCCGTCTCATCATTCCCTTGA

ACTCACAGGTTTATGTGGATAATTTTATCTCTGAGGTTTCCAGGAGCTCAATGGAAAATG

GGATTTCATGCGAGAGCGCCCTGATTCCCTCTAAGTGCAGAGGTCTATGTAAAATCAGCC

CGACTGCCTCTTCCCTCGGTTCACAGGCTCCGGCAGGGACAGGGCTTTCCGCCCTTTCCT

GCCTGCAGGAAGGCGGATTCCCGAAGCCCCCAGAGAGGGCGGGCAGGGCTGGGCAGAGCC

GCCGGGCGGATCACAAGTCTGGAGCGCCAGGCACGGGCGGGCGGGAACTGGAGGTCGCGC

GGGCGGTTCCACAGCTCCGGGCCGGGTCAGGGCGGCGGCTGCGGGGGCGGCCGGGCTGGG

GCCGGGCCGGGGCCTGACTGACCGGCCGGTGATTCCCCGCAGAGGATTTCGTGTACCAGT

TTAAGGGCATGTGCTACTTCACCAACGGGACCGAGCGCGTGCGGGGTGTGACCAGATACA

TCTATAACCGAGAGGAGTACGCGCGCTTCGACAGCGACGTGGGGGTGTATCGGGCGGTGA

CGCCGCTGGGGCCGCCTGCCGCCGAGTACTGGAACAGCCAGAAGGAAGTCCTGGAGAGGA

CCCGGGCGGAGTTGGACACGGTGTGCAGACACAACTACCAGTTGGAGCTCCGCACGACCT

TGCAGCGGCGAGGTGAGCGTCGTCGCCCGTCTGCGAGGCCCATCCTTGGCAGGGGCCCAG

AGTCTCTGCGCCAGGAGGGGCGAAGGGGGCGCGGCCTCTGGAACCTGAGCCTTGTTCGTT

CCACCCCGGCTGACAGGAGGAGGCGGTGGGGGTGCTGGGGGCTGGTGCATCGGAGGGGCA

GGGACCTAGGGCAGAGCAGGGGGACAGGCAGAGTTGGTCAAGCTGCCTAGTTTCGCCCCA

TCCTCCCCGTCCGTCGGCCTCGCCCTCTGCTCTGCACGTTCTTGCCTCGTGCCTTATGCG

TTTGCCTCCTCGTGCCTTACCTTTACTAAGCAGTTCTCTCTGCCCCCAATTTCCGCCCTC

TTCCCCTGCCCGCCCGCCCGGCTAGCACTGCCGCACCCGGCAAGGTCCACCTACACAGCT

CATGCAGTGGGAAGCTTCAGACTTGGCCTGATGGAGTTAGGGCTGCCCCACAACTGCGCG

CAGGGCATCCAGCAATTACAGTTGTCAAATAAGATATTTTGACTTTTGACTTCAAATTAT

TATTCATCGTAATTCTGTTTTCTTAAATGGCTCTCATTCATGGCGGAGCTCTTTGAGATG

AGAGTGTTTTAATCATTGCATGCCTGGTATCTGACACATTGACTGGCATGTGGTATGAGC

TCAATGATCTTCTGTTAAATTAATGAATAAATGTGCTCAGCTGCCAATCCACTTAGGCTC

AAGGGAAAGCAGAGGATAAATAGAGCCTTAAAAATGGACTTTTATTAATTATTTTCTGTC

ATTTTGCTTAATTCTTTAAAGTAAACTCTTATTGACTTGGATCTTAATAGAGTTTGTGAA

TACAAAGTCTGAGGAAAAAAGTGTTTGCTAAAAATAAAAACAACACTTGAATGATGTTTG

TAAGGCAGTTTTAATTTCTTAGAAAAGCTGAACAAATGGCACAATGCAAAGAGCAGAAGT

TTTGGAATAAATAGATTGAAGCAATTAAATTATTGGATAAAAATAGTTTCAGGTTGCTTT

TGGCTTAGGTTCTCCCCTCCCCCCATCACTATCCACTTCAGGAATAAACATTCTGAAAGT

CAATTTTACCCATTTAGTGAGCACTTATTTCTAGACAGTTACCTTATCAAATACCGTCTA

TGTTACGTCATTTAATCTCACAGTTGCCTGTGCATTAGAGATTAGCATCACCACTTTATA

TATCCTAATATTAGTACATGACAAACACTTTAAGTAATCAGCCCACAAGTACTCACCAAG

ACCTTAAGCCTCCCAAAGTACACAATATTCTTTATGTTCTTCACTACACATCTATAGAGT

CAAAGGGACATAAAGCCTTGTTAAAGCCAGTTTTGACTAGAAGCAGCAATGAGTCTCTTC

CTGTTTGATCTCCATGTTAATGGGACAAAATGATACTTTCAAGGCATTGAAAATTTATGA

TTAATCAATCCCTAGTCTGACTCCAGTGTTACCTATGCAGATTCACAAAACTTTTAGTTT

ACTTTAAACTCCCTTGCCTTCTTTTGACTCACATCGTAGTGCCAGCAAGTACTTACATTT

TTGCTATTTTGGTTCTATTTCCATAAAATTTATTTTATCATCTTTTCTCATAAGTTTGTG

CCCTCTATTTTACTCCCAGTCTGTGTAAGATGAACAAATCTTATAAGGCCACATAGCTGA

CTGTGATTTCAGGTGGACTCCAGGAAGGAGAACCAAAGAAAAGTTCAAGTCCAAGCAGAA

ACCGTGATTCCTTCCGGATGATGGCTCAAGAGTGCCTTTTAATTGGGGTGCAACCTGCTG

ACCTCAGCAAATCTCAGCTATATTTATATGTTCACATTACAGGCTCATTAACCTAGGCTG

ATCTCTGCAAGGATCTCAGAATATTTTCTACAGATAACATACATGATAATATCTGATTTC

AGGACAAAAAAGTAATTCTCAATAGCAAGGGAATGGAGTAGGGTAGACAGCTAGTAATTA

AACTCACTTGTATGTTAAAAATAAATTAAGGAAAAAAAGAAAATGAGAGAACATATTACT

AAATAAAGAAAGCATACATTAAATATTTACTATAGTTTCACACTAAGAGAATAAAGGAAA

TGCAATAAAGTGGCCTGAAAGATAAAGGATGAGACGTGTAAAGAGACAGGGAAAGATGTG

TCATTTTTTTACTATGAGCAGCAGTCTGAGAAGATAAAGGAATCGAGTTATGGGCAAACA

TGATGTTTGATCAGTGTTATTTGTTTTGAAGGCCTGCCTACTTTTGTTTCAAATATTACA

GACTTTTGAAATCACATTCTTTTTGTTTTTTGCTGTCTGTTACTAGATCGCACATTCTGT

AAAGGCAGGGACCATGGTATGTTGTTTATCTTTGGATTCTCAGTGATTGTCATATTTATA

TTTGTTGAATGAATCTTAATCCAAGACTTGGACTCCAGGTATCTTTCCACTCTGGTTCCA

AGGAGGGACCATTCCCAATGGTAGACGTGCTGTGTGGTCTCACATCTCACTCCTGTCTTT

TCCTGTCTGTTACTGCCCTCAGTGGAGCCCACAGTGACCATCTCCCCATCCAGGACAGAG

GCCCTCAACCACCACAACCTGCTGGTCTGCTCAGTGACAGATTTCTATCCAGCCCAGATC

AAAGTCCGGTGGTTTCGGAATGACCAGGAGGAGACAACTGGCGTTGTGTCCACCCCCCTT

ATTAGGAACGGTGACTGGACCTTCCAGATCCTGGTGATGCTGGAAATGACTCCCCAGCGT

GGAGACGTCTACACCTGCCACGTGGAGCACCCCAGCCTCCAGAACCCCATCATCGTGGAG

TGGCGTAAGGGGATATTGAGTTTCTGTTACTATGGGCCCCACAAGACAAAGGGCAGAGCT

CCTTCTGACCCATTCCTTCCCATCTCTTATCCCTGATGTCACTACTGAGCTGGGGATCAC

AGGAGACTAGAGCACCTCTTGCTCCATGGCAAGTGCATCAGAAGAATCCTGATCTCATCA

CCTTTCCAGATGCTAGGGAAATTATTCTACGTACTGTTTCTCCAGATCCCAGTCCTGATA

GCTCGGAGGGACTTATTATTAGGGCTGGTGACTGGGATCTTAGGGTTTAAGGTATGGATG

AGTTCCTGAGGAGTGGAGATCTGCTTCCCCGCTCTCTCACCTACTCACTATACCGAAGGA

CCTATTGGCTGGCTTTCCCCTCCCTTAGGGGTGGTCTGAATGGAGGACTAGGTTCCTTTG

ACACTTTCACCTCCTGCATCTCAGACTGGACTTCAGCTCCTCAGCAGGGATGCTATGGGG

TGTAGGGACAAACGCTGACACTCAGGCTCTGCTTCTTAGGGGCTCAGTCTGAATCTGCCC

AGAGCAAGATGCTGAGTGGCATTGGAGGCTTCGTGCTGGGGCTGATCTTCCTCGGGCTGG

GCCTTATTATCCATCACAGGAGTCAGAAAGGTGAGGAACCCCAAGGGAAAAGGGGAAGAT

GGGCTGTGACCCAGACCCTCTGTTCAGGGAGGTCCTGTCTCTAGATGTGGCTCTTTCCTC

CTGACCCTGAGAGGAAGAAAACTGAGCTGGAGGTGGGAGGAGACAGGACAAGATTGGAGG

AGGCATTGGAATCTGATTTTACTAGTTGAAAGGTAGCCCTGTCACACAGGTGACTGATAG

AGCTTATTCCAGGATATACTTACCATTCATCATCTCATTGGCTCCTTTCCAAAAGCTTCC

TCCATTAAGAGGGTCAGAGCCTTGGCCTCCTTGCCTTCTAGTGACAATTTTCTTTGTTTT

AGGGGATTTTAAATTAGGGTACTTAAGGCCTTGAAGAACATGAGTGGTAAGAGAATATAA

CTCTAATTAAGTCACATGTGTCATTTTCCTTTGGGGTGAAAGAGTGGCTGTTTGTGTAAT

GAGACCTTTCTCTGCATAACTTCCTTTTGTAAGACCTCAAGGGCCTCCACCAGCAGGTGA

TATTTCAGCCATGAGCCAGTGTGGGGGGGCACAGGTGTAAGAGGGAAGAGCATGAGCTGA

ATGCACCTGACCACAATGGTCTCTGTTCATGGTATATTTGCTGCTATGAGGATCAAGACT

TAGGGTCGAAGTTTGCCAGTTTCTAGGAATCTCCAGAGGTTGTTCCCCAGAACCAAGCCT

TAACTTTGGTGGTATCTTCTTGTGAAATGTGAAGCCAGAACCACAGCTTAAATGTTAGAC

AAGAGGATGATGCCCACTTTGTGCCACATGTTGGTGGCTACTGCCTGTAGGCATTTTCCA

GTGACTGAAAGAGGCTGCTAGTGGTAGGGATGAGGTATCATCCAATTTTCTAAAAAGATT

GAACCCTTCATATTCCCCAGAAGAGTAACAGCTGTTCCGCCACTTCCCACATATCTGCAT

CAAGCTGAAGTTCTGTGTCCTCACGAGCTGATTTCACCTTTGCACAGATCTTGCGGGAGG

TGACAATAATACATTCTGGACCTCAGCTTTCTCTGTCTGAAGCTGCAGGGGGCCCCTGAG

GGGTGGGGGAGATTGCAGGCCCACCAGCGTACCCTGTGCTGATCATCCCTCTTCTCTCTT

CTTCAGGGCTCCTGCACTGACTCCTGAGACTATTTTAACTGGGATTGGTTATCACTTTTC

TGTAACGCCTGCTTGTCCCTGCCCAGAATTCCCAGCTGCCTGTGTCAGCCTGTCCCCCTG

AGATCAGAGTCCTACAGTGGCTGTCACGCAGCCACCAGGTCATCTCCTTTCATCCCCACC

TCGAGGCTGATGGCTGTGACCCTGCTTCCTGCACTTACCCAGAGCC

>HLA:HLA12879 DQB1*05:01:01:03

TTCTAAGACCTTTGCTCTTCTCCCCAGGACTTAAGGCTCTTCAGCGTGTCTAAGACAACA

GCAGTAAAAATTTCTGTGACAGCAATTTTCTCTCCCCTGAAATATGATCCCCACCTAATT

TGCTTTATTAAAAATCCCAAGTATAATAACAACTGGTTTTTAACAATATTACAGAGATGT

TTACTGTTGAATTGCATTTTTCTTTTGCCTCTCAAAATCCCTGAGGAATTTGTTCTTCAG

CTCTTCTATAATCGAGAGGAAATTTTCACCTCAGATGTTCATCCAGTGCAATTGGAAGAC

GTCACAGTGCCAGGCACTGGATTGAGAACCTTCACAAAAAAAAATGTCTGCCCAGAGACA

GATGAGGTCCTTCAGCTCCAGTGCTGATTGGTTCTTTTCCAAGCGACCATCCAATCCTGC

CACGCACGGAAACATCCACAGGTTTTTATTCTTTCTGCCAGGTACATCAGATCCATCAGG

TCTGAGCTGTGTTGACTACCACTACTTTTCCCTTCGTCTCAATTATGTCTTGGAAGAAGT

CTTTGCGGATCCCCGGAGACCTTCGGGTAGCAACTGTCACCTTGATGCTGGCGATCCTGA

GCTCCTCACTGGCTGAGGGCAGAGACTCTCCCGGTAAGTGCAGGAAAGCTGCTCTCCAGA

GCCGCCACTCTGGGAACAGGCTTTCCTTGGGCTGGGGTATGGGGGATGGTGATCTCCATG

GTCTTGGACACAATCTTTCATCAACATTTCCTCTTTTTTGGGAAAGAAAGCTATGTTGCA

TTCCCATTTACCTTTTAGTGATGAAATGAGGACAATCCAATCCCCATCCTACAGGTTTAA

GCCTGGAAGAGGAGGAGAGAGGAGAGGAAAGAGGAGATAAAGTGTACATTTACTACCAGT

GATAGGACAAAGTGAGCATGGGGTTATTTTTGAAGATATTAATTTCTCCAAAGACACAGC

AGGATTTGTCATTTCAGCGTGCCCCAAGACTTTGCCTGGACTAAATATTATGAGATTCTG

CAGTGGGAAATGTAAGGCAGCGATGGTGTCTGTAGTCTCCGTATTTGAGGAAAAGTAGTC

TGTATTCCTGACTGACTGGAGCGTTTGTGGAGGCAAAATCTTGGTACTGAGGGAAGCTGA

CTGGCTGACCACAGACAGGGAGTCTTCAGGTTTCACTGATTTATGGGCAAATGGTGACTT

GAGTGGGATTCAGAGACCCGAGTTGTTGGTGGACTAAATTTAGTAGAAAGGAGGATGTAA

AGAAGGGAAATAACACATATTATGAAACCACTCACTCAGACACAGAACAATACTTTACAT

AAATTCTCTCTCACTCCTTCTAACATCCTGTGTGCAGATATCATGATTTTCTTTTACACA

ATTATACTTGTGATACGGATATTCTGCCCAATATGGATATACACAACCTGGCCAAGCTGG

TAACTGCCACAGTTTAATTGGAATCTAGTTTATCAAATTCAAAAGCTTATGCTCTTTCCA

TGAATAAATATTTCTATCTAGGACTCAGAGTTGTAGGTCCTTTCCAACATAGAAGGGAGT

GAACCTCAACGGGTCTTGGGAGGGTAAATCCAGGCATGGGAAGGAAGGTATTTTACCCAG

GGACCAAGAGAATACGCGTATCAGAACGAGGACAAGATTAATTCCTGGACCTGTCGCATC

ATTCCCTTGAACTCACAGGTTTATGTGGATAAGTTTATCTCTGAGGTTTCCAGGAGCTGC

ATGAAAAATGGGATTTCATGCGAGAACGCCCCGATCCCTCTAAGTGCAGAGGTCCATGTA

AAATCAGCCCGACTGCCTCTTCGCTGGGTTCACTGGCTCAGGCAGGGACAGGGCTTTCCG

CCCTTTCCTGCCTGTAGGAAGGCGGATTCCCGAAGCCCCCAGAGAGGGCGGGCAGGGCTG

GGCAGAGCCGCCGGGAGGATCCCAGGTCTGGGGCGCCAGGCACGGGCTGGCGGGAACTGG

AGGTCGCGCGGGCGGTTCCACAGCTCCGGTCCGGGTCAGGGCGGCGGCTGGGGGCGCAGC

CGGGCTAGGGCCAGGCTGGGGCCTGACTGACTGGCCCGTGATTCCCCGCAGAGGATTTCG

TGTACCAGTTTAAGGGCCTGTGCTACTTCACCAACGGGACGGAGCGCGTGCGGGGTGTGA

CCAGACACATCTATAACCGAGAGGAGTACGTGCGCTTCGACAGCGACGTGGGGGTGTACC

GGGCAGTGACGCCGCAGGGGCGGCCTGTTGCCGAGTACTGGAACAGCCAGAAGGAAGTCC

TGGAGGGGGCCCGGGCGTCGGTGGACAGGGTGTGCAGACACAACTACGAGGTGGCGTACC

GCGGGATCCTGCAGAGGAGAGGTGAGCGTCGTCGCCCCTCCGTGAGGCCCACCGTTGGCC

GGGACCTCTAGTCTCTGTGCTGGGAGGGGCGATGGGGTTGCGGCCTCTGAAACCTGAGCC

CCGTTTATTCCACCCCAGGGGACAGGAGTTGGCGGCGTGAGTGGTGGGGCAGGTGCATCA

GATGGGCGGGGACCTAGGGCAGAGCAGGGAGACAAGCAGAGTTGGCCAGGCTGCCTAGTG

TCCCCCCAGGGTCCTCGTCTGTTGGCCTCGTCCTCCGCTCTGCACGTTTCTCGCCTCGTG

CCTTATGTGTTTGCCTCCTCGTGCCTTACCTTTGCTAAGCAGTTCTCTCTGCCCCCAATG

CCCACCCTCTTCCCCTGCCCGTCCGCCCCACTAGCACTGCCCCATTCAGCAAGGCCCACG

TGCGCAGCTCGCGCCGCAGGAAGCTTCAGGCTTGGCCTGGTGGAGTTAGGGCTGCCCCAC

AACTGCGCCCAGGGCATCCAGCAATTACAGTTGCAAAATAAGATATTTTGACTTTTTGGC

TTCAAATCATTATTCATCGTAATTCTGTTTTCCTAAATGGCTATCACTAATGGCGGAGAT

TTTTGAGGTGGGAGACTGTTTAAATTATTGCATGCTTGGTACCTGACACTTTGACTGGTA

TGTGGTATGAGCTCAATGATCTTCTGTTAAATTCATGAATAAATGTACTCAGCTGCCCAT

CCACTTAGGCTCAAGAAAAAAACAGAGGCTTACAAATGGACTTTGTTAATTATTTTCTAT

CATTTTGCCTAATGCTTTAAAGTAAACTCTTATTGACTAGGATCTTAATAGAATTTGTGA

ATACAAAGTCTGAGAAAAAAAGTGTTTGCTAAAAATAAAAACAATGCTTGAATGACGTTG

TAAGGCAGAGTTTTAATTTCTTAGACAAGCTGAACAAATGGCACAATGCAAAGGGCAGAA

GTTTGGGAATAAATAGGTTGAAGCCATTAAATTATTAGATAAAAATAGTTTCAGGTTGCT

TTTGGCCTGGGTTCTCCCCTCCCTCCATCACTATCCACTTCAGGAATAAACATTCTGAAA

GTCAATTTTACCCATTCAGGTAGCACTTATTTCTAGACAGTTGCCTTATCAAATACCATA

TATGTTGTGCCATTTAATCTCACAGTTACCTGTGCATTAGAGATTAGCATCACCACTTGA

TATATCCTAATATTGGTACAGGATAAACACTTTAAGTAATCAGCCCACAATTACTCACCA

AGACCTTAAGCCTCCCAAAGTATAAACCATTCTTATGTTCCTCAGTGTACATCCATAGAG

TCTAAGGGATGTAAGGCCTTGTTGAAGCCAGTTTTGACCAGAAGCAGCAATGAGCCTATT

CCTGTTTGTTCTCCATGTTAATGGGGCATTGAAAATTCACTGATTAATCAATCCCTAGTC

TGACCCCAGTGTTATCTATGCAGGTTCACAAAACTTTTAGATTACTTTACACCCCCTTGC

CTTCTTTTGACTCACATTCTAATGCCAGCAAGTACTTATATTTTTGCTATTTCAGTTCTA

TTTCCATAAAATTTATTTTATCATCTTTTCTCATAAAATTGTGCCCTCTATTTTACTCCC

AGTCTGTTTAAGATGAACAAATCTTACAAGGTCACATAGCTGACTGTGATATCAGTTGGA

CTCCAGGAAGGAGAACCTAAAGAAAAGTTCAAGTCCAAGCAGAAACCGTGATTCCTTCCG

GATGATGGCTCAAGAGTGATGTTTAACTGGGATGCAACCTGCTGACCTCAGCAAATCCTA

GTTATATGTATGTGTTCACATTACAGGCTCATTAGCCCAGGCCGACCTCTGCATGGATCT

CAGAATATTTTCTATGGAGAACATACATGATAATGTCTGATTTCAGAACAAGAAAGTAAT

TCTCAATAGCAAGGAAATGGAGTAGGGTAGACAGCTAGTAACTAAACTCACTTGTGCGTT

AAGAAGAAATTAAGGAAAAAAGAAAATAAGAGAACATATTACTAAATAAAGAAACACACA

TTAAATATTTGCTATAGTTTCACACTAAGAGAATAAAGGAAATGCAATAAAGTGGCCTGA

AAGGTAAAGGATGAGATGTGTAAAAGAGGCAGGGAAAGATGTATAATAATTTTTTACTAT

GAGCAGCAATCTGAGAAGATAAAGGAATTGAGCTGTGGGCAAACATGATGTTTGATCAGT

GTTATTTGTTTTCAAGGCCTGCCTACTTTTTTTTTCAAATATTACAAACTTTTGAAATAA

CATTCTTTTTGTTTTTTACTGTCTGTTACTAGATTGCATATTCTATAAATGAAGGGACCA

TGGTATGTTGTTTATCTTTGGATTCTCAGTGATTGTCAAATTTATATTTGTTGAAGGAAC

CTTAATCCAAGACTTGGACTCCAGGTATCTTTCCACTCTGGTTCCAAGGAGGGACCCTTC

CTCATGGTGGACGTGCTGTGTGGTCTCACGTCTCACTCCTGTGTCTTTTCCTGTCTGTTA

CTGCCCTTAGTGGAGCCCACAGTGACCATCTCCCCATCCAGGACAGAGGCCCTCAACCAC

CACAACCTGCTGATCTGCTCGGTGACAGATTTCTATCCAAGCCAGATCAAAGTCCGGTGG

TTTCGGAATGATCAGGAGGAGACAGCCGGCGTTGTGTCCACCCCCCTCATTAGGAACGGT

GACTGGACCTTCCAGATCCTGGTGATGCTGGAAATGACTCCCCAGCGTGGAGATGTCTAC

ACCTGCCACGTGGAGCACCCCAGCCTCCAGAGCCCCATCACCGTGGAGTGGCGTAAGGAG

ATATTGAGTTTCTGTTATTATGGGCCCCACAAGACAAAGAGCTCCTTCTGACCCATTCCT

TCCCATCTCTTATCCCTGATGTCACTACTGAGCTGGGAATCACAGGAGACTAGAGCACCT

CTAGTTCCATGGCGAGTGCATCAGAAGAATCCTGATCTCATCACCTTTCCAGATGCTAGG

GAAATTACTCTACATACTGTTGCTCTGGATCCCAGTCCTGATAGCTCTGACGGACTGATT

CTTAGGGCTGGTGATTGGGATCTTAGGGTCTAAGGTTATGGATGAGTTCCTGAGGAGCAG

AGATTTGCTTCCCCACTCTCTCACCTACCCACTGTATCCAAGGACCTATTGTCTGGCCTT

TCCCCTCCTTAGGGGTGGTCTGAATGGAGAGCTAGGTTCCTTTGATGCCTTCACCTCCTG

CACCTCAGACTGGACTTCAACTCCTCAGCAGGGATGCTATGGGGTGTGGGGACAAACACT

GACACTCAGGTTCTGCTTTTTAGGGGCTCAGTCTGAATCTGCCCAGAGCAAGATGCTGAG

TGGCGTTGGAGGCTTCGTGCTGGGGCTGATCTTCCTTGGGCTTGGCCTTATCATCCGTCA

AAGGAGTCGGAAAGGTGAGGAACCCCAGGGGAAAAGGGGAAGATGGGCTGTGACCCAGAC

CCTCTGTTCAGAGTGGTCCTGTCTGTAGATTAGCTCTTTCCTCCTGACCCTGAGAGGAAG

TGCGAGGAGACAGGACAAGATGGGAGGAGGCATTGGAATCTGATTTTACTGGTTGAAAGG

TAGCGCTGTCACAGAGCTGACTGATAGAGCTTATTCCAGGGCATCCTTACCATTCATCAT

TGTCTCACTGGCTCCTTTCCAAAAGCTTCCTCCATTAAGAGGGTCAGAGCCTCAGCCTCC

TTTCTTTCTGGTGACAATTTCCTTTGTTTTAGGGGATTTTAAATTAGGGTGCTGAAGGCC

TGGAAGAACATGGGTGGGAAGAGAATGTAACTCTAAGTCATGTGTGTCATTTTCCTTTGG

GGTGAGAGAGTGGCTGTTTGTGTAATGAGACCTTTCTCTGCATAACTTCCTTTTGTAAGA

CCTCAAGGGCCTCCACCAGCAGGTAATATTTCAGCCATGATCCAGTGTGGGGAGGCACAG

GTATAAGAGGGAAGAGCATGAGCTGAGTGTACCTGACCACAGTGGTCCATGTTCGTGGCC

TATTTGCTGCTATGAGGATCAAGACTTAGGGGAGAAGTTTGCCAGTTTCTAGGAATCTCC

AGACATTGTTCCCCAGAACCAAGCCTTAACTTTGGTGGCATCTTCTTGTGAAATGTGGAG

CCAGAACCACAGCTTAAATGTTAGACACTAGGATGATGCCCACTTTGTGCCACATGATGG

TGGCTACTGCCTGTAGGCATTTTCCAGTGACTGAAAGAGGCTGCTAGTGGTCGGGATGAG

ATATCATCCAATTTCCTAAAAAGACTGAACCCTTCATATTCCCCAGAAGAATAACAGCTG

TTCCCCACCTCCCACACATCTGCATCAAGCTGAAGTTCTGTGTCCTCGTGAGCTGATTTC

ACCTTTGCACAGATCTTGGGGGAGGTGATGACAATACACCCTGGACCTCAACTTTCTCTG

TCTGAAGCTGCAGGGGGCCCCTGAAGGGTGGGGGAGATGGCAGGCCCACCAGGATACCCT

GTGCTGATCAATCCTCTTCTCTCTTCTCCAGGGCTTCTGCACTGACTCCTGAGACTGTTT

TAACTAAGACTGGTTATCACTCTTCTGTGATGCCTGCTTGTCCCTGCCCAGAATTCCCAG

CTGCCTGTGTCAGCTTGTCCCCCTGAGATCAAAGTCCTACAGTGGCTGTCACGCAACCAC

CAGGTCATCTCCTTTCATCCCCACCCCAAGGCGCTGGCTGTGACTCTGCTTCCTGCACTG

ACCCAGAGCC

>HLA:HLA00647 DQB1*06:03:01:01

TTCTAAGAACTTTGCTCTTCTCCCCAGGACTTAAGGCTCTTCAGCGTGTCTAAGACAACA

GCAGTAAAAATTTCTGTGACAGCAATTTTCTCTCCCCTGAAATATGATCCCCACCTAATT

TGCTTTATTAAAAATCCCAAGTATAATAACAACTGGTTTTTAACAATATTACAGAGATGT

TTACTGTTGAATCGCATTTTTTTCTTTTGCCTCTCAAAATCCCCGAGGCATTCGTTCTTC

AGCTCTTCTATAATCGAGAGGAAATTTTCACCTCAGATGTTCATCCAGTACAATTTGAAG

ACGTCACAGTGCCAGGCACTGGATTGAGAACCTTCACAAAAAAAATGTCTGCCCAGAGAC

AGATGAGGTCCTTCAGCTCCAGTGCTGATTGGTTCTTTTCCGAGGGACCATCCAATCCTG

CCACTCACGGAAACATCCACAGGTTTTTATTCTTTCTGCCAGGTACATCAGATCCATCAG

GTCCGAGCTGTGTTGACTACCACTGCTTTTCCCTTCGTCTCAGTTATGTCTTGGAAGAAG

GCTTTGCGGATCCCCGGAGACCTTCGGGTAGCAACTGTCACCTTGATGCTGGCGATGCTG

AGCTCCCTACTGGCTGAGGGCAGAGACTCTCCCGGTAAGTGCAGGGCAGCTGCTCTCCAG

AGCCGCCACTCTGGGAACAGGCTCTCCTTGGGCTGGGGTATGGGGGATGGTGATCTCTAT

GATCTTGGACACAATCTTTCATCAACATTTCCTCTCTCTTGGGAAAGAAAGCTATGTTGC

ATTCCCATTTATCTTTTAGTGATGAGATGAGGACAATCCAATCCCCATCCTACAGGCTTA

AGCCTGGAAGAGGAGGAGAGAGGAGAGAAAGAGGAGATAAAGTGTACATTTACTACCAGC

GATAGGACAAAGTGAGCATGGGGTTATTTTTGAAGATATGAATTTCTCCAAAGACACAGC

AGGATTTGTCATTTAGGCGTGCCCCAAGACTGCCTGGACTTAGGATTATGAGATCCTGCA

TTGGAAAATGTGAGGCAGCAATGGTGTCTGTAGTCTCTGTATTTGAGGAAAATTTGTCTC

TATTCCTGACCGAGTGGAGCATTTGTGGAGGCAAAATCTTGGTGCTGAGGGAAGCTGACT

GGCTGACCACAGAGAGGGAGTCTTCGGGTTTCACTGATTTATGGGCAAATGGTGACTTGA

GTGGGATTCAGAGACCCGAGTTAATGGTGGACTGAATTTAGTAGGAAGGAGGATGTAAAG

AAGGGAAATAACACATATTGTGAAACCACTCACTTCAGACAGAACAATACTTTATATAAC

TTCTCTCTCACTCCTTCTAACATCCTGTGTGTAGATATCATGATTTTCTTTTACACAATT

ATACTTGTGATATGGATATTCTGTTACATAACCTGCCCAAACTGGTGACTGCCACAGTTT

AACTGGAATCTAGTTTATCAAATTCAAAAGCTTGTGCTCTTTCCATGAATAAATGTCTCT

ATCTAGGACTCAGAGGTGTAGGTCCTTTCCAACATAGAAGGGAGTGAACCTCAACGGGAC

TTGGGAGGTTAAATCTAGGCATGGGAAGGAAGGTATTTTACCCAGGGACCAAGAGAATAC

GCGTGTCAGAACGAGGCCAGGCTTAATTCCTGGACCTATCTCGTCATTCCGTTGAACTCT

CAGATTTATGTGGATAACTTTATCTCTGAGGTATCCAGGAGCTTCATGAAAAATGGGATT

TCATGCGAGAACGCCCTGATCCCTCTAAGTGCAGAGGTGCATGTAAAATCAGCCCGACTG

CCTCTTCGCTGGGTTCACAGGCTCAGGCAGGGACAGGGCTTTCCTCCCTTTCCTGGATGT

AGGAAGGCAGATTCCAGAAGCCCGCAAAGAAGGCGGGCAGAGCTGGGCAGAGCCGCCGGG

AGGATCCCAGGTCTGGAGCGCCAGGCACGGGCGGGCGGGAACTGGAGGTCGCGCGGGCGG

TTCCACAGCTCCAGGCCGGGTCAGGGCGGCGGCTGCGGGGGCGGCCGGGCTGGGGCCTGA

CTGACCGGCCGGTGATTCCCCGCAGAGGATTTCGTGTACCAGTTTAAGGGCATGTGCTAC

TTCACCAACGGGACGGAGCGCGTGCGTCTTGTAACCAGACACATCTATAACCGAGAGGAG

TACGCGCGCTTCGACAGCGACGTGGGGGTGTACCGCGCGGTGACGCCGCAGGGGCGGCCT

GATGCCGAGTACTGGAACAGCCAGAAGGAAGTCCTGGAGGGGACCCGGGCGGAGTTGGAC

ACGGTGTGCAGACACAACTACGAGGTGGCGTTCCGCGGGATCTTGCAGAGGAGAGGTGAG

CGTCGTCGCCCCTCCGTGAGGCCCACCCTTGGCCGAGACCCCGAGTTTCTGTGCCGGGAG

GGGCGATGGGGGCGAGGTCTCTGAAATCTGAGCCCAGTTCATTCCACCCCAGGGGAAAGG

AGGCGGCGGCGGGGTGGTGGGGGCAGGTGCATCGGAGGGGCGGGGACCTAGGGCAGAGCA

GGGGGACAAGCAGAGTTGGCCAGGCTGCCTAGTGTCCCCCCCAGCCTCCCCGTCCGTCGG

CCTCGTCCTCCGCTCTGCACGTTTCTCGCCTCGTGCCTTATGTGTTTGCCTCCTCGTACC

TTACCTTCGCTAAGCAGTTCTCTCTGCCCCCAGTGCCCACCCTCTTCCCCTGCCCGCCGG

CCTCGCTAGCACTGCCCCACCCAGCAAGGCCCACGTGCGCAGCTTGCGCCGCAGGAAGCT

TCAGGCTTGGCCTGGTGGAGTTAGGGCTGCCCCACAGCTGCGCGCAGGGCATCCAGCAAT

TACAGTTGCGAAATAAGATATTTTGACTTTTTGGCTTCAAATCATTATTCATCGTAATTC

TGTTTTCTTAAATGGCTACCACTAATGGCGGAGATCTTTGAGGCGAGAGACTGTTTAATT

ATTGCATGCCTGGTACCTGACACATTGACTGGTATGTGGTGTGAGCTCAATGATCTTCTG

TTAAATTCATGAATAAATGTACTCAGCTGCCAATCCAGTTAGGTTCAAGAAAAAAACAGA

GGCTTACAAATGAACTTTATCAATTATTTTCTAACATTTTGCTTAATGCTTTCAAGTAAA

CTCTTACTGACTTGGATCTTAATAGAATTTGTGAATACAAAGTCTGAGGAAAATGTGTTT

GCTAAAAATAAAAACAATGCTTGAATGATGTTGTAAGGCAGAGTTTTAATTTCTTAGAAA

AGCTGAACAAATAGCACTATGCAAAGATCAGAAGTTTGGGAATAAATAGGTTGAAGCTAT

TAAATTATTGGATAAAAATAGTTTCAGGTTGCTTTTGGCCTAGGTTATCCCCTCCCCCCC

ATCACTATCCACTTCAGGAATAAACATTCTGAAAGTCAATTTTACCCATTCAGGAAGCAC

TTATTTCTAGACAGTTGCCTTATCAAATACCATCTATGTTATGTCATTTAATCTCACAGC

TACCTGTGCATTAGAGATTAGCATCACCACTTTATATATCCTAATATTGGTACATGATAA

ACACTTTAAGTAATCAGCCCACAATTACTCACCAAGATCTTAAGCCTCCCAAAGTACACA

ACATTCTTATGTTCCTCACTACACATATGTAGAGTCTAAGGGATGTAAGGCCTTGTTAAA

GCCAGTTTTGACCAGAAGCAGCAATGAGTCTATTCCTGTTTGTTCTGTGTGTTAATGCGG

CATTGAAAATTCATGATTAATCAATCCCTAGTCTGACCCCGGTGTTATCTATGCAGGTTC

ACAAAACTTTTAGTTTGCTTTACACTCCCTTGCCTTCTTTTGACTCACATCCTAATGCCA

GCAAGTACTTATATTTTTGCTATTTCAGTTTTATTTCCATAAAATTTATTTTATCATCTT

TTCTCATAAATTTGTGCCCTCTATTTTTACTCCCAGTCTGTTGAAGATGAACAAATCTTA

TAAGGCCACATAGCTGACTCTGATTTCAGTTGGCCTCCAGGAAGGAGAACCTAAAGAAAA

GTTCAAGTCCAAGCAGAAACCATGATTCCTTCCGGATGATGGCTCAAGAGTGCCATTTAA

TTGGGGTGCCACCTGCTGACCTCAGCAAATCCCAGTTATATGTATATGTTCACATTACAG

GCTCATTAGCCCAGGCCGACCTCTGCACGGATCTCAGAATATTTTCTGTGGAGAACATAC

ACGATAATGTCTGATTTCAGAACAAGAAAGTAATTGTCAATAGCAAGGAAATGGAGTAGG

GTAGACAGCTAGTAATTAAACTCACTTGTGCGTTAAAAAGAAATTAAGGAAAAAAAGAAA

ATGAGAGAACATATTACTAAATAAAGAAAGCACACATTAAATATTTGCTATAGTTTCACA

CTAAGAGAATAAAGGAAATGTGATAAAGTGGCCTAAAAGGAAAGGATGAGATGTGTAAAA

GAGGCGGGGAAAGATGTGTCATTTTTTTTTACTATGAGCAGCAATCTGAGAAGATAAAGG

AATTGAGTTGTGGACAAACATGATGTTTGATCAAGGGTTATTTGTTTTGAAGGCCTGCCT

ACTTTTTTTTCAAATATTACAAACTTTTGAAATAACATTCTACTTTTTTTTCAAATATTA

CAAACTTTTGAAATAACATTCTTTTTGTTTTTTGCTGTCTGTTACTAGATTGCACATTCT

ATAAATGCAGGGACCATAGTATGTTATTTATCTTTGAATTCTCAGTGATTGTCAAATTTA

TATTTGTTGAATGAACCTTAATCCAAGACTTCGACTCCAGGTATCTTTCCACTCTGGTCC

AAGGAGGGATCCTTCCTCATGGTAGACGTGGTGTGTGTTCTCACATCTCACTCCTATGTC

TTTTCCTGTCTGTTACTGCCCTCAGTGGAGCCCACAGTGACCATCTCCCCATCCAGGACA

GAGGCCCTCAACCACCACAACCTGCTGGTCTGCTCGGTGACAGATTTCTATCCAGGCCAG

ATCAAAGTCCGGTGGTTTCGGAATGATCAGGAGGAGACAGCCGGCGTTGTGTCCACCCCC

CTTATTAGGAATGGTGACTGGACTTTCCAGATCCTGGTGATGCTGGAAATGACTCCCCAG

CGTGGAGATGTCTACACCTGCCACGTGGAGCACCCCAGCCTCCAGAGCCCCATCACCGTG

GAGTGGCGTAAGGGGATATTGAGTTTCTGTTACTATGGGCCCACAAGACAAAGAGCTCCT

TCTGACCCATTCCTTCCCATCTCTTATCCCTGATGTCACTACTGAGCTGGGAATCACAGG

AGACTAGAGCACCTCTTGCTCCATGGCAAGTGCATCAGAAGAATCCTGGTCTCATCACCT

TTCCAGATGCTAGGGAAATTACTCTACATACTGTTGCTCTGAATCCCAGTCCTGATAGCT

CTGAGGGACTGATTCTTAGGGCTGGTGACTGGGATCTTAGGGTCTAAGGTTATGGATGAG

TTCTTGAAGAGCAGAGATTTGCTTCCCCACTCTCTCACCTATTCACTGTATCCAAGGACC

TATTGGCTGGTCTTTCCCCTCCTTAGGGGTGGTCTGAATGGAGAACTAGGTTCCTTTGAT

GCCTTCACCTTCTGCACCTCAGACTGGACTTCAACTCCTCAGCAGGGATGCTATGGGGTG

TGGGGACAAACACAGACACTCAGTTCTGCTCTTTAGGGGCTCAGTCTGAATCTGCCCAGA

GCAAGATGCTGAGTGGCGTTGGAGGCTTCGTGCTGGGGCTGATCTTCCTTGGGCTGGGCC

TTATCATCCGTCAAAGGAGTCAGAAAGGTGAGGAACCCCAGGGGAAAAGGGGAAGATGGG

CTGTGACCCAGACCCTCTGTTCAGAGTGGTCCTGTCTGTAGATTAGCTCTTTCCTCCTGA

CCCTGAGAAGAAGTGCGAGGAGACAGGACAAGATGGGAGGAGGCATTGGAATCTGATTTT

ACTGGTTGAAAGGTAGCGCTGTCACAGAGCTGACTGATAGAGCTTATTCCAGGGCATCCT

TACCATTCATCATTGTCTCACTGGCTCCTTTCCAAAAGCTTCCTCCATTAAGAGGGTCAG

AGCCTCAGCTTCCTTTCTTTCTAGTGACAATTTCCTTTGTTTTAGGGGATTTTAAATTAG

GGTGCTGAAGGCCTGGAAGAACATGGGTGGGAAGAGAATGTAACTCTAAGTCATGTGTGT

CATTTTCCTTTGGGGTGAGAGAGTGGCTGTTTGTGTAATGAGACCTTTCTCTGCATAACT

TCCTTTTGTAAGACCTCAAGGGCCTCCACCAGCAGGTAATATTTCAGCCATGATCCAGTG

TGGGGAGGCACAGGTATAAGAGGGAAGAGCATGAGCTGAGTGTACCTGACCACAGTGGTC

CATGTTCATGGCCTATTTGCTGCTATGAGGATCAAGACTTAGGGGAGAAGTTTGCCAGTT

TCTAGGAATCTCCAGACATTGTTCCCCAGAACCAAGCCTTAACTTTGGTGGCATCTTCTT

GTGAAATGTGGAGCCAGAACCACAGCTTAAATGTTAGACACTAGGATGATGCCCACTTTG

TGCCACATGATGGTGGCTACTGCCTGTAAGCATTTTCCAGTGACTGAAAGAGGCTGCTAG

TGGTCGGGAAGAGATATCATCCAATTTCCTAAAAAGACTGAACCCTTCATATTCCCCAGA

AGAATAACAGCTGTTCCCCACCTCCCACACATCTGCATCAAGCTGAAGTTCTGTGTCCTC

ATGAGCTGATTTCACCTTTGCACAGATCTTGGGGGAGGTGATGACAATACACCCTGGACC

TCAACTTTCTCTGTCTGAAGCTGCAGGGGGCCCCTGAAGGGTGGGGGAGATGGCAGGCCC

ACCAGGATACCCTGTGCTGATCAATGCTCTTCTCTCTTCTCCAGGGCTTCTGCACTGACT

CCTGAGACTATTTTAACTAGGATTGGTTATCACTCTTCTGTGATGCCTGCTTGTGCCTGC

CCAGAATTCCCAGCTGCCTGTGTCAGCTTGTCCCCCTGAGATCAAAGTCCTACAGTGGCT

GTCACGCAGCCACCAGGTCATCTCCTTTCATCCCCACCCCAAGGCGCTGGCTGTGACTCT

GCTTCCTGCACTGACCCAGAGCC

>DQB1_in_MANN

GGCTGTGGGTCAGTGCAGGAAGTAGGGTCACAGCCATCCGCCTCAAGGTGGGGATGAAAG

GAGATGACCTGGTGGCTGCGTGACAGCCACTGTAGGACTCTGATCTCAGGGGGACAGGCT

GACACAGACAGCTGGGAATTCTGGGCAGGGACAAGCAGGCGTTACAGAAAAGTGATAACC

AATCCCAGTTAAAATAGTCTCAGGAGTCAGTGCAGGAGCCCTGAAGAAGAGAGAAGAGGG

ATGATCAGCACAGGGTACGCTGGTGGGCCTGCCATCTCCCCCACCCCTCAGGGGCCTCCT

GCAGCTTCAGACAGACAAAGCTGAGGTCCAGAGTGTATTATCATCACCTCCCCCAAGATC

TGTGCAAAGGTGAAATCAGCTCATGAAGACACAGAACTTCAGCTTGATGCAGATGTGTGG

GAGGTGGGGGAACAGCTGTTACTCTTCTGGTGAATATGAAGGGTTCAATCTTTTTAGGAA

ATTGGATGATACCTCATCCCTACCACTAGCAGCCTCTTTCAGTCACTGGAAAATGCCTAC

AGGCAGTAGCCACCATCATGTGGCACAAAGTGGGCATCATCCTAGTGTCTAACATTTAAG

CCGTGGTTCTGGCTCCACATTTCACAGGAAGATACCACCAAAGTTAAGGCTTGGTTCTGG

GGAACAACCTCTGGAGATTCCTAGAAACTGGCAAACTTCACCCCTAAGTCTTGATCCTCA

TAGCAGCAAATATACCATGAACAGAGACCACTACTGTCAGGTACACTCAGCTCATGCTCT

TCCCTCTTACACCTGTGCCCTCCCCACACTGGATCACGGCTGAAATATTACCTGCTGGTG

GAGGCCCTTGAGGTCTTACAAAAGGAAGTTATGCAGAGAAAGGTCTCATTACATGAACAG

CCACTCTCTCACCCCAAAGGAAAATGACACATGTGACTTAATTAGAGTTATATTCTCTTC

CCATTCATGTTCTTCAAGTCCTTAAGCACCCTAATTTAAAATCCCCTAAAATAAAGGAAA

TTGTCACTAGAAGGCAAGGAGGCCGAGGCTCTGACCCTCTTAATGGAGGAAGTTTTTGGA

AAGGAGCCAGTGAGACAATGATGAATGGTAAGGATGCCCTGGAATAAGCTCTATCAGTCA

GCTCTGTGACAGGGCTACCCTTCAGCTAGTAAAATCAGATTCCAATGCCTCCTCCAACCT

TGTCCTGTCTCCTTCCACTTCCAGCTCAGCTTTCTTCCTCTCAGGGTCAGGAGGAAAGAG

CTACATCTAGAGACAGGACCTCTCTGAACAGAGGGTCTGGGTCACAGGCCATCTTCCCCT

TTTCCCCTGGGGTTCCTCACCTTTCTGACTCCTGTGATGGATGATAAGGCCCAGCCCGAG

GAAGATCAGCCCCAGCACGAAGCCTCCAATGCCACTCAGCATCTTGCTCTGGGCAGATTC

AGATTGAGCCCCTGAGAAGCAGAGCCTGAGTGTCAGTGTTTGTCCCCATACCCCATAGTT

TCCCTGATGAGGAGCTGAAGTCCAGTCTGAGATGCAGGAGGTGAAGGCATCAAAGGAACC

TAGTTCTCCATTCAGACCACCCCTAAGGGAGGGAAAGGCCAGCCAATAGGTACTTGGATA

CAGTGAGTAGGTGACAGAGTGGGGAAGCAGATCTCCACTCCTCAGGAACTCATCCATAAA

CTTAGACCCTAAGATCCCAGTCACCAGCCCTAATAATCAGTTCCTCAGAGCAATCAGGAC

TGGGATCCAGAGCAACAGTATGTAGAGTAATTTCCCTAGCATCTGGAAAGGTGATGAGAT

CAGGATTCTTCTGATGCACTCGCCATGGAACTAGAGGTGCTCTAGTCTCCTGTGATTCCC

AGCTCAGCAGTGACATCAGGGATAAGAGATGGGAAGGGATGGGTCAGAAGGAGCTCTGTC

CTTTGTCTTGTGGGGCCCACAGTAACAGAAACTCAATATCCCCTTACGCCACTCCACGGT

GATGGGGCTCTGGAGGCTGGGGTGCTCCACGTGGCAGGTGTAGACGTCTCCACGCTGGGG

AGTCATTTCCAGCATCACCAGGATCTGGAAGGTCCAGTCACCATTCCTAATAAGGGGGGT

GGACACAACGCCAGCTGTCTCCTCCTGGCCATTCCGAAACCACCGGACTTTGATCTGGGC

TGGATAGAAATCTGTCACCGAGCAGACCAGCAGGTTGTGGTGGTTGAGGGCCTCTGTCCT

GGATGGGGAGATGGTCACTGTGGGCTCCACTGAGGGCAGTAACAGACAGGGAAAGATATA

GGAGTGAGATGTGAGACCACACAGCATGCCTGCTGTGAGGAAGGTCCCTCCTTGGAACCA

GAGTGGAAAGATACTTGGAGTCCAAGTCTTGGATTAAGGTTCCTTCAACAAATATAAATA

TAACAATCACTGAGAATCCAAAGACAAACAACATACCATGGTCCCTGCCTTTATAAAATG

TGCAACCTAGTAACAGACAGCAAAAAACAAAAAGAATGTTATTTCAAAAGTTTGTAATAT

TTGAAAAAAATTTAGGCAGGCCTTGAAAACAAATAACACTGATCAAACATCATGTTTGCC

CGTAACTCGATTCCTTTATCTTCTCAGATTGCTGCTCATAGTAAAAATACTACACCCCTT

TACACATCTCATCCTTTACCTTTCAGGCCACTTTATTGCATTTCCTTTATTCTCTTAGTG

TGAAACTATAGTAAATATTTAATGTATGCTTTCTTTATTTAGTAATATGTTCTCTCATTT

TCTTTTTTCCTTAATTTCTTTTTATCACACAAGTGAGTTTAATTACAGCTGTCTACCCTA

CTCCATCCCCTTGCTATTGAGAATTACTTTCTTCTTCTGAAATCAGGCATTATTATGTAT

GTTCTCCATAGAAAATATTCTGAGATCTGTGCAGTGGTCGGTCTGGGTTAATGATCCTGT

AATGTGAACACATAAATATAGCTGGGATTTGCTGAGGTCACCAGGTGGCACCCCAATTAA

ATGGCACTCATGAGCCATCATCTGGAAGAAATCACGGTTTCTGCTTGGACTTGAACTTTT

CTTTAGGTTCTCCTTCCTGGAGTCCAACAGAAATAACAGTCAGCTATGTGGTCTTATAAG

ATTTGTTCATCTTAAACAGATTGGGAGTAAAAATAGAGGGCATAAATTTATGAGAAAAGA

TGATAAAATAAATTTTATGGAAATGGAACTGAAATAGCAAAAACATAAGTATTTGCTGGC

ATTAGGATGTGAATCAAAAGAAGGCAAGGGAGTATTAAGTAAACTAAAGGTTTTGCAAAC

CTGCATAGATAACACTGGGGTCAGACTAGCGATTGATTAATCATGAATTTTCAATGCCTT

GAAAGTATCATTTTGTCCCATTAACATGGAAAACACACAGGAATAGACTCATTGCTGCTT

CTGGTCAAAACTGGCTTTAACGAGGCTTTACGTCCCTTAGACTCTATGGATGTGTAGTGA

AGAACACAAAGAATGTTGTGTACTTTGGGAGGCTTAAGGTCTTGGTGAGCAATTATGGGC

TGATTACTTAAAGTGTTTATCATGTACCAATATTAGCCTACAGTGACCATCTCCCCATCC

ATGACCAATAAAGTGTTTATCATGTACCAATATATAAAGTGGTGATGCTAATCTCTGATG

CACAAGTAACTGTGAGATTAAATGACGTAACATAGATGGTATTTGATAAGGCAACTGTCT

AGAAATAAATGCTCACTAAATGGGTAAAATTGACTTTCAGAATGTTTATTCCTGAAGTGG

ATAGTCATGGGGGGAGGGGAGAACCTAGGCCAAAAGCAACCCGAAACTATTTTTATCCAA

TAATTTAATGGCTTCAATCTATTTATTCCAAAACTTTTGCTCTTTGCATTGTGCCATTTG

TTCAGCTTTTCTAAGAAATTAAAAGTGCCGGCCGGGCACTGTGGCTCACACCTGTAATCC

CAGCACTTTGGGAGGCCGAGGCGGGCGGATCATGAGGTCAGGAGATCCAGACCATCCTGG

CTAACACGGTGAAACCCCGCCTCTACTAAAAATACAAAAAAATTAGCCGGGCGTGGTGGC

ACGCGCCTGTAATCCCACCTACTCAGGAGGCTGAGGCAGGAGAATGGCGTGAACCCGGGA

GGCGGAGCTTGCAGTGAGCCGAGATCGCGCCACTGCACTCCGGCCTGGGCGACAGAGTGA

GAATCCGTCTCAAAAAAAAAAAAAAAAAAGAAAAGAAATTTAAACTGCATTACAATATCT

TTCAAGCGTTGTTTTTATTTTTAGCAAAAACTTTTTTCCTCAGATTTTGTATTCACAAAC

TCTATTAAGATCCAAGTCAATAAGAGTTTACTTTAAAGCATTAAGCAAAATTATAGAAAA

TAATTAATAAAGTCCATTTTTAAGGCTCTGTTTACCTCTTTTTTTTTCTTGAGCCTAAGT

GGATGGGCAGCTGAGTACATTTATTCATTAATTTAACAGAAGATCATTGAGCTCACACCA

CATACCAGTCAATGTGTCAGGTACTAGGCATGCAATGATTAAAACACCCTCACCTCAAAG

ATCTCTGCCATGAATGAGAGCCATTTAAGAAAACAGAATTACGATGAATAATGATTTGAA

GCCAAAAGTTAAAATATCTTATTTCACAACTGTAATTGCTGGATGTCCTGCGCGCAGTTG

TGGAACAGCCCTAACTCCACCAGGCTAAGCCTGAAGCTTCCTGCTGCAAGAGCTGTGCAC

GTGGGCCTTGCTGGGTGGGGCAGTGCTAGCGGGGCGGGCGGGCGGGCAGGGGAAGAGGGC

GGGCATTCTGAGCAGAGAGAGCTGCTTAGCAAAGGTAAGGCAAAAGGAGGCAAATGCATA

AGGCACGAGGCAAGAACATGCAGAGCAGAGGACAAGGCCGACGAACGGGTAGGCTGGGGC

GACACTAGGCAGCCTGGCCAACTCTGCCTGTCCCCCTGCTCTGCCCTAGGTCCCCGCACC

TCCGATGCACCTGCCCCCACCACTCTCGCCGCTGCCTCCTGTCCTCTGGGGTGGAACAAA

CGGGGCTCAGGTTCCAGAGGCAACGACCCTTCGCCCCTCCTGGCGCAGAGACTTGGGGCC

AAGGGTGGGCCTCGCAGAGGGGCGACGCCGCTCACCTCGCCGCTGCAAGGTCGTGCGGAG

CTCCAACTGGTAGTTGTGTCTGCACACCCTGTCCACCGCCGCCCGTTTCCTCTCCAGGAT

GTCCTTCTGGCTGTTCCAGTACTCGGCGGCAGGCAGCCCCAGCAGCGTCACCGCCCGGAA

CTCCCCCACGTCGCTGTCGAAGCGCACGATCTCTTCTCGGTTATAGATGCTTCTGCTCAC

AAGACGCACGCGCTCTGTCCCGTTGGTGAAGTAGCACATGCCCTTAAACTGGTACACGAA

ATCCTCTGCGAGGAATCACCGGCCAGTCAGTGCGGCCCAGCCCGTCCGCCCCCGCAGCCG

CCACCCTGACCCGGCCCGGAGCTGTGGAACAGCCCGCGCGACCACAAGTTCCCGCCGGCC

CGTGCCTCGCGCTGCAGACCTGGGATCCTCCCGGCGGCTCTGCCCAGCCCTGCCCGCCCT

CTCGGGGGTCTTCGGGAATCCGCCTTCCTAAAGGCAGGAAAGGGAGGAAAGCCCTGTCCC

TGTCTCGGCCTGTGAACCAAGTGAAGAGGCAGTCGGGCTGATTTTACATGGACCTCTGCA

CTTAGAGGGATCAGGGCGTTCTCGCATGAAATCTCATTTTCCATGGAGCTCCTGGGTACC

TCAGAGATAAAGTTATCCACATAAACCTGACAGTTCAAGGGAATGACGAGACAGGTCCAG

GAATTAAACCTGTCCTCTTCCGATACGCCTATTCTCTTGGTCCCTGGGTAAAATACCTTC

CTTCCCATGCCTGGATTTACCCTCCCAAGTGCCCTGTGAGGTTCACTCACTTCTGTGTTA

GAAAGAAGGGAGTCCTAGATCTCTGAGTCCTAGAAAGAAACATTTATTCACCGAAAGAGC

ACAAGCTTTTGAATTTGATAAACTAGATTCCCATTAAACTGTGGCAGTCACCAGCCCGGG

CAGGTTATGTAACAGAATATCCATATCACAAGTATAATTGTGTAAAAGAAAATCATGATA

TCTACACACAGGATGTTAGAAGGAGTGAGAGAGAATTTATGTAAAGTATTGTTCTGTGTC

TGAAATGAGTGGTTTCACAGTATGTGTTATTTCCCTTCTTTACATCCTCCTTTCTACTAA

ATTTAGTCCATCATCAACTCGGGTATCTGAATCCCACTCAGGTCACCATTTGCCCATAAA

TCAGTGAAACCTGAAGGCTCTCTTTCTGTGGTCAGCCAGTCAGCTTCCCTCAGTACCAAG

ATTTTGCCTCCACAAACGCTCCACTGGGTCAGGAATACAGACAACTTTTCCCCAAATACG

GAGACTACAGACACCATTGCTGCCTTGCATTTCCCAATGCAGGAAATCATAATATTTAGT

CCAGGCAAGTCTTGGGACACGCCTAAATGGCAAATCCTGCTGTGTCTTTGGAGAAATTCA

TATCTTCAAAAATAACCCCATGCTCACTTTGTCCTATCACTGGTAGTAAATGAACACTTT

GTCTCCTCTTTTCTCTCCTCTCTCCTCCTCTTCCAGGCTTAAGCCTGTAGGATGGGATTG

GATTGTCCTCACTTCATCATTAAAAGATAAATGGAAATGCAACATAGCTCTCTTTCCCAA

AACAGAGGAAATGTTGATAAAAGATTGTGTCCGAGATTATGGAGATCACCATCCCCGTAC

CCCAGCCCAAGGAGAGCCTGTTCCCAGAGTAGCGGCTCTGGAGAGCAGCTGCCCTGCACT

TACCGGGAGAGTCTCTGCCCTCAGCCACTGGGGTGCTCAGCATCGACAGCATCAAGGTCA

CAGTTGCTGCCCGAAGGCCTCCGGGGATCCGCAAAGCCTTTTTCCAAGACATAATTGAGA

CGAAGGGAAAAGTGGTAGTCAACACAGCTCGGACCTGATGGATCTGATGTACCTGGCAGA

AAGAATAAAAATCTGTGGATGTTTCCATGCGTGGTAGGATTGGATGGTCCCTTGGAAAGG

AACCAATCAGCACTGGAGCTGAAGGACCTCATCTGTCTCTGGGCAGATTTTTTTTGTGTG

AAGGTTCTGAATCCAGTGCCTGGCACTGTGACGTCTTTCAATTGCACTGGATGAACATTT

GAGGTGAAAATTTCCTCTCAATTATGGAAGAGCTGAAGATTGAATGCCTAAGGGATTTTA

AGAAGCCAAAGAAAAATGCGATTCAACAGTAAACATCTTTGTAATATTGATTAAAAACCA

GTTGTTCTTATACTTGGGATTCTTTCAATAGGGCAAATTAAGTGGGGATCATATTTCAGG

GGAGAGAAAATTGCTGTCATAGACATTTTTACTGCTGTTGTCTTAGACACACCCTGAGGA

GCCTTAAGTTTTGGTGAAAAGAGCAAAGTTCTTAGAA

>HLA:HLA00662 DRA*01:01:01:01

TCACTAATGTGCTTCAGGTATATCCCTGTCTAGAAGTCAGATTGGGGTTAAAGAGTCTGT

CCGTGATTGACTAACAGTCTTAAATACTTGATTTGTTGTTGTTGTTGTCCTGTTTGTTTA

AGAACTTTACTTCTTTATCCAATGAACGGAGTATCTTGTGTCCTGGACCCTTTGCAAGAA

CCCTTCCCCTAGCAACAGATGCGTCATCTCAAAATATTTTTCTGATTGGCCAAAGAGTAA

TTGATTTGCATTTTAATGGTCAGACTCTATTACACCCCACATTCTCTTTTCTTTTATTCT

TGTCTGTTCTGCCTCACTCCCGAGCTCTACTGACTCCCAAAAGAGCGCCCAAGAAGAAAA

TGGCCATAAGTGGAGTCCCTGTGCTAGGATTTTTCATCATAGCTGTGCTGATGAGCGCTC

AGGAATCATGGGCTATCAAAGGTAGGTGCTGAGGGAATGAAATCTGGGACGATAGACTAC

GAAGCATTGGAGAAAAGACCTATGGACATTTGGAAGATAATGTGTGGAGTGAAAGAATAG

TGTGACAGGTATTATGTGGTCTCGACAGAAAGTATAACAAATTGTGGTTTGGTGGAGTTC

TTCCCTCACCACAAACTGAAGTAAGTCAAATTTGGTTTAGAGGGTCAAAACTGAGTTGTG

TATTGATGAATAGCACGGTCCTGCTACAAGCCAAACTGGGGGTGGGGGTGGGGGTGGGGG

AGGAAGAATATTTTCTGGCAAGCATTAACAAGTTATATTTCTGGGCTTTAATTATTCTTT

CTGGAAAATTAGTAAAATTAAAAACTAAAAACCACACATAGTTTTGCTAGAATTAAATGA

AAAAAAAAGTTATTAGCCCTGTTCTTATCTGAATACATGATACAGTAGTTATTTTTTGGA

GTGTAAATCCTGTCGGTATATATTGAGCACATATATTGTGTTGAAGATTACTAGAAGGAA

AAGTCATCAAAAAGCAACAATTTACCCCAGGAAAAGGGGAGGGAAGGCATGCTGATATGA

GTTGCCTCATGGGACAGTGATAGCCATTCCCTGCCTTCCCATCTCCATGGTACAGCAGAT

CTTATATCATGTTAACTTAGTAATATTTCCAAGAGAGTAGAAAAATAAGTAAGGAAATGG

GGAATCTGATATTATTGTCTCTCATCTCCAGAGCAACATTGGTGCTGTTGTAAAGATGTA

CTGTAGAAAAGTATTCTTCACCCAGCGTGACCCCCACAGAAGGTGTCAGGTAGACTTGAA

ATAAGCAAAGTAATAACCCAGCTCCCATACCCATAGTGGCAATTGTAGATTTCTATTGCC

CCAAAAGAGCCATACATAGGGATACTTACCTAGAAAGACAGAGGATCTTCCCTTGGTTTG

TGAAGAGGCAGCTAGTATATTTGTGTGTGTTTGCATAGATGCAAACGGTAAATAAATTCC

TAGGTTTATCAATACACAGTCAAACATTAAAATCTCTCATCTTGGCTGGGCACGGTGGCT

CACGCCTGTAATCCCAGCACTTTGGGAGGCCGAGGCGGGCGGATCACGAGGTCAAGAGAT

CGAGACCGTCCTGGGCAACATGGTGAAACCCCGTCTCTACTAAAAATACAAAAAATTAGC

TGGGTATGGTGGCACACGCCTGTAGTCCCAGCTACTCGGGAGGCTGAGGCAGGAGAATTG

CTTGAGCCCGGGAGGCGGAGGTTGCAGTGAGCTGAGATGGTGCCACTGCACTCCAGCCTG

GCGATAGAGCAAGACTCCGTCTCAAACAACCAAACCAAAACAAAACAAAATATCTCACCT

TATCTTTGAAGACTAAGGAAAAAAAAAATCTCCCACTCATCGATACACTCCACAGAGGCA

GCATACTCTCCCAGTGTAGCTTTCTCTTTTCATGTTCATTATTCCCTTGGTGTTGGTTAT

TCTCAATGTCAATCGTAACAGAACATCTTCCATAATAACAGTCCCAATTTAAGGAGCATT

AAGATAAAAGGTGGAATTGCCAAGGTCAATCCAGACGAGAACCTTCTCATAGAGGTAACC

ACCGTGTGGGTTTGGATGCTGGGAAGCAGGGGGACTATGACGCTACAAGGTCTCAGTCTT

AATTTTTGGAGTACTTCAGTCCCCAGGTATATTTTCCATAGATTTGGCCCTTAAATAAAG

AGAAGCTTCTGACTCTAAAATGTAAACAGTGCTTGTTACAGTCTTGTTGATATATTAAGA

AATTACTCACCTTATCTCATTTAATCTTAAAAACAAACCCCTGACAGGATCAAAACCACA

GCAGGACTACATAATAGGAAAACTATACATAAATAGGTAGAATAATCTGCTCAGGATCAC

TAGGTAAGTTGCTGAATAAGAATTCAAGATGTTTTTGATCCCAGAGTTTAAAACCCAACC

TTTCAAACAGTGTTTCCTTCTTCTTAGAGTACAATGTTCTGAGAAAGAGATCCTCTGGAA

TTCTGGCCTAAGTGTATTTAATGCCCGGGTAAAGAAAGTGAGAGAACATTTCTCTTTAGG

GGCTGCTGCTGGATTTCTAAAAAGAAAATAATTTCTCAGCTAGTAACATGGAGCCAAACA

ACAGCTTCACAAGACTCTGGGTTCTTTAGCCCTCATCTCCTTCAATCCACCCTCTTTATA

ACCAGTCCTTCTTGTTTTTCCCCTCCCAGCTTTGTTCAGCAGCATGCCCTTCACCCAGAC

CTTGTCTTGTCACTCATCCCTACTCGCCATCATTCTTTCATTCCTCTTGGCCCAATCTCT

CTCCACCACTTCCTGCCTACATGTATGTAGGTTATTCATTTCCCTCTCTTGATTCCCCCC

ACCCAACTCTCTTTCTCCATTTCTTGCCTTTCAGAAGAACATGTGATCATCCAGGCCGAG

TTCTATCTGAATCCTGACCAATCAGGCGAGTTTATGTTTGACTTTGATGGTGATGAGATT

TTCCATGTGGATATGGCAAAGAAGGAGACGGTCTGGCGGCTTGAAGAATTTGGACGATTT

GCCAGCTTTGAGGCTCAAGGTGCATTGGCCAACATAGCTGTGGACAAAGCCAACCTGGAA

ATCATGACAAAGCGCTCCAACTATACTCCGATCACCAATGGTACCTCCCTCTCTGCTGCA

CTCCTGGACATGGGAATCCATAGTTTGAAAGTAGTTGCTTCAGCTCTTTGTGTTAGATTA

TTGTAACTGATTTTCCCTCCAAGGGCCTAACCTTGCCATTAACAAGCCCCAAATTCTCAT

GCCAGAGGTCTGAGAACTTTATGGGTTTGATCCTATCTTGTTGTGCTCAAGTCTTGTCTC

TGTCATCCATGGTCTCCTACGAAGTCATTGCCCTAAGTTCATGCTAGGGGAGCCAGAAGG

GAAGTCCTTGGATATCTTATACCTCAATATTGGCTCAATTTCTTGGGGAGGGGGTGCTGT

CAGAGATTGTTATCTGAGGATGTGACATAGATTTCTCAGGGCACAATTTCAACTACTTTT

TCAGCTTTAGGGTTTTTAGATACGTTTGTACCACAATTGAGCATGGGAGGGAGAGGGGTG

AGCCTAAGCAGTGATGGCTGATTTCTGTCACGTCTGTCATGTGTCCCCCAGTACCTCCAG

AGGTAACTGTGCTCACGAACAGCCCTGTGGAACTGAGAGAGCCCAACGTCCTCATCTGTT

TCATCGACAAGTTCACCCCACCAGTGGTCAATGTCACGTGGCTTCGAAATGGAAAACCTG

TCACCACAGGAGTGTCAGAGACAGTCTTCCTGCCCAGGGAAGACCACCTTTTCCGCAAGT

TCCACTATCTCCCCTTCCTGCCCTCAACTGAGGACGTTTACGACTGCAGGGTGGAGCACT

GGGGCTTGGATGAGCCTCTTCTCAAGCACTGGGGTATGGACCAACACTCAATCTCCTTTA

TTTCAAGGTTTCCTCCTATGATGCTTGTGTGAAACTCGGTGTTCTAACTGTTTCATAATA

TCTGCTACAATTAATATAACTGTCTTCTCCTACTATCCAGCTTCCTCCTTTTTTTAATCT

GTAATTCTCTCAATACATCATTCTGTCTTCCTCTTCTTTAATCTATGAATAACTTTTCTC

TTTATTAAGAACCCTACATTTGATTCTGAGTGTTACTTCTTCCCACACTCATTACCATGT

ACTCTGCCTTATCTCCCCCCAGAGTTTGATGCTCCAAGCCCTCTCCCAGAGACTACAGAG

AACGTGGTGTGTGCCCTGGGCCTGACTGTGGGTCTGGTGGGCATCATTATTGGGACCATC

TTCATCATCAAGGGAGTGCGCAAAAGCAATGCAGCAGAACGCAGGGGGCCTCTGTAAGGC

ACATGGAGGTGAGTTAGGTGTGGTCAGAGGAAGACATATATGGAGATATCTGAGGGAGGA

AAACAGGGTGGGGAAAGGAAATGTAATGCATTTAAGAGACAAGGTAGGAACAGATGTGGC

TCTTGATTTCTCTTTGCTAGAATGAATCAGACATTGGTATCATCTGGTATCCCAAAGCTT

CAGGGTCTGTCATCCCTTTCTATAGACGGGCACCTTGATCACGGCTCCAGTCTTAGAAAT

CATCTCCAGTACCTAAAACCATTGTTTCACATTAGAATACTGAGTCTAGGGATCTAGAAA

ATACATTAGAATATGGAGTCTAGGGATCTAGAAAATACTGAGTCTAGGGATCTAGAAAAA

TAAGCCTCAAGATTTGGGCACATCCTAGCTTGTATTTCCTGGGGCAGGTCATCAGTTCAG

AAGCATTTCCAGATCCTGGCTCCTTTCAGGTTAGGGTCAATTCATTGCATGAAATGGGAA

TCTCTTAGAGGCCAATGCCTGCTTTTGCTTCTTTAGTCTCAAATGTAGTATGAGAAACTC

TAAAAAAAGGTAAAGCATGGTTGCTTATTATGTTCAGTTGGAGAGTAGGAACTAACTGTA

TACAGTTAGTTCATGTTGGAAAGGTTAGATGAACATTGAAAGAATTTTGCAAAGTCAAAG

GATTAAGAGAGAAGAGGAAGGAATCTGAAGCAAGGAGCTCAAAACGGATCTTAAATTCCT

TGGTAACTATGTGTGTCTTGCTATAGGTGATGGTGTTTCTTAGAGAGAAGATCACTGAAG

AAACTTCTGCTTTAATGACTTTACAAAGCTGGCAATATTACAATCCTTGACCTCAGTGAA

AGCAGTCATCTTCAGCGTTTTCCAGCCCTATAGCCACCCCAAGTGTGGTTATGCCTCCTC

GATTGCTCCGTACTCTAACATCTAGCTGGCTTCCCTGTCTATTGCCTTTTCCTGTATCTA

TTTTCCTCTATTTCCTATCATTTTATTATCACCATGCAATGCCTCTGGAATAAAACATAC

AGGAGTCTGTCTCTGCTATGGAATGCCCCATGGGGCATCTCTTGTGTACTTATTGTTTAA

GGTTTCCTCAAACTGTGATTTTTCTGAACACAATAAACTATTTTGATGATCTTGGGTGGA

ATTTTTGGTGTTTAAGCCAGTTCTTTGGGTGGCGGTGGGGGGTGGGGAGTCGGTCCTGGG

GAATATATGTGATCCTTTCCCGGTAAAATATCTGAATGTTGAATTTATCTTATAAATTCT

AGAATTCATCAGACATATCCCGGTTCATTTGGGCTTGGTCTCATTTTGTGCATCTGCAGG

CAACCCTCTTGTTGTGGTCTAGTCCTCATCAGGAAAACCTAAAGTGGGGTTGGTTTGTTG

GGAGATCTCTA

>HLA:HLA06469 DRA*01:01:01:02

TCACTAATGTGCTTCAGGTATATCCCTGTCTAGAAGTCAGATTGGGGTTAAAGAGTCTGT

CCGTGATTGACTAACAGTCTTAAATACTTGATTTGTTGTTGTTGTTGTCCTGTTTGTTTA

AGAACTTTACTTCTTTATCCAATGAACGGAGTATCTTGTGTCCTGGACCCTTTGCAAGAA

CCCTTCCCCTAGCAACAGATGCGTCATCTCAAAATATTTTTCTGATTGGCCAAAGAGTAA

TTGATTTGCATTTTAATGGTCAGACTCTATTACACCCCACATTCTCTTTTCTTTTATTCT

TGTCTGTTCTGCCTCACTCCCGAGCTCTACTGACTCCCAACAGAGCGCCCAAGAAGAAAA

TGGCCATAAGTGGAGTCCCTGTGCTAGGATTTTTCATCATAGCTGTGCTGATGAGCGCTC

AGGAATCATGGGCTATCAAAGGTAGGTGCTGAGGGAATGAAATCTGGGACGATAGACTAC

GAAGCATTGGAGAAAAGACCTATGGACATTTGGAAGATAATGTGTGGAGTGAAAGAATAG

TGTGACAGGTATTATGTGGTCTCGACAGAAAGTATAACAAATTGTGGTTTGGTGGAGTTC

TTCCCTCACCACAAACTGAAGTAAGTCAAATTTGGTTTAGAGGGTCAAAACTGAGTTGTG

TATTGATGAATAGCACGGTCCTGCTACAAGCCAAACTGGGGGTGGGGGTGGGGGTGGGGG

AGGAAGAATATTTTCTGGCAAGCATTAACAAGTTATATTTCTGGGCTTTAATTATTCTTT

CTGGAAAATTAGTAAAATTAAAAACTAAAAACCACACATAGTTTTGCTAGAATTAAATGA

AAAAAAAAAGTTATTAGCCCTGTTCTTATCTGAATACATGATACAGTAGTTATTTTTTGG

AGTGTAAATCCTGTCGGTATATATTGAGCACATATATTGTGTTGAAGATTACTAGAAGGA

AAAGTCATCAAAAAGCAACAATTTACCCCAGGAAAAGGGGAGGGAAGGCATGCTGATATG

AGTTGCCTCATGGGACAGTGATAGCCATTCCCTGCCTTCCCATCTCCATGGTACAGCAGA

TCTTATATCATGTTAACTTAGTAATATTTCCAAGAGAGTAGAAAAATAAGTAAGGAAATG

GGGAATCTGATATTATTCTCTCTCATCTCCAGAGCAACATTGGTGCTGTTGTAAAGATGT

ACTGTAGAAAAGTATTCTTCACCCAGCGTGACCCCCACAGAAGGTGTCAGGTAGACTTGA

AATAAGCAAAGTAATAACCCAGCTCCCATACCCATAGTGGCAATTGTAGATTTCTATTGC

CCCAAAAGAGCCATACATAGGGATACTTACCTAGAAAGACAGAGGATCTTCCCTTGGTTT

GTGAAGAGGCAGCTAGTATATTTGTGTGTGTTTGCATAGATGCAAACGGTAAATAAATTC

CTAGGTTTATCAATACACAGTCAAACATTAAAATCTCTCATCTTGGCTGGGCACGGTGGC

TCACGCCTGTAATCCCAGCACTTTGGGAGGCCGAGGCGGGCGGATCACGAGGTCAAGAGA

TCGAGACCGTCCTGGGCAACATGGTGAAACCCCGTCTCTACTAAAAATACAAAAAATTAG

CTGGGTATGGTGGCACACGCCTGTAGTCCCAGCTACTCGGGAGGCTGAGGCAGGAGGATT

GCTTGAGCCCGGGAGGCGGAGGTTGCAGTGAGCTGAGATGGTGCCACTGCACTCCAGCCT

GGCGATAGAGCAAGACTCCGTCTCAAACAACCAAACCAAAACAAAACAAAATATCTCACC

TTATCTTTGAAGACTAAGGAAAAAAAAAATCTCCCACTCATCGATACACTCCACAGAGGC

AGCATACTCTCCCAGTGTAGCTTTCTCTTTTCATGTTCATTATTCCCTTGGTGTTGGTTA

TTCTCAATGTCAATCGTAACAGAACATCTTCCATAATAACAGTCCCAATTTAAGGAGCAT

TAAGATAAAAGGTGGAATTGCCAAGGTCAATCCAGACGAGAACCTTCTCATAGAGGTAAC

CACCGTGTGGGTTTGGATGCTGGGAAGCAGGGGGACTATGACGCTACAAGGTCTCAGTCT

TAATTTTTGGAGTACTTCAGTCCCCAGGTATATTTTCCATAGATTTGGCCCTTAAATAAA

AAGAAGCTTCTGACTCTAAAATGTAAACAGTGCTTGTTACAGTCTTGTTGATATATTAAG

AAATTACTCACCTTATCTCATTTAATCTTAAAAACAAACCCCTGACAGGATCAAAACCAC

AGCAGGGCTACATAATAGGAAAACTATACATAAATAGGTAGAATAATCTGCTCAGGATCA

CTAGGTAAGTTGCTGAATAAGAATTCAAGATGTTTTTGATCCCAGAGTTTAAAACCCAAC

CTTTCAAACAGTGTTTCCTTCTTCTTAGAGTACAATGTTCTGAGAAAGAGATCCTCTGGA

ATTCTGGCCTAAGTGTATTTAATGCCCGGGTAAAGAAAGTGAGAGAACATTTCTCTTTAG

GGGCTGCTGCTGGATTTCTAAAAAGAAAATAATTTCTCAGCTAGTAACATGGAGCCAAAC

AACAGCTTCACAAGACTCTGGGTTCTTTAGCCCTCATCTCCTTCAACCCACCCTCTTTAT

AACCAGTCCTTCTTGTTTTTCCCCTCCCAGCTTTGTTCAGCAGCATGCCCTTCACCCAGA

CCTTGTCTTGTCACTCACCCCTACTCGCCATCATTCTTTCATTCCTCTTGGCCCAATCTC

TCTCCACCACTTCCTGCCTACATGTATGTAGGTTATTCATTTCCCTCTCTTGATTCCCCC

CACCCAACTCTCTTTCTCCATTTCTTGCCTTTCAGAAGAACATGTGATCATCCAGGCCGA

GTTCTATCTGAATCCTGACCAATCAGGCGAGTTTATGTTTGACTTTGATGGTGATGAGAT

TTTCCATGTGGATATGGCAAAGAAGGAGACGGTCTGGCGGCTTGAAGAATTTGGACGATT

TGCCAGCTTTGAGGCTCAAGGTGCATTGGCCAACATAGCTGTGGACAAAGCCAACCTGGA

AATCATGACAAAGCGCTCCAACTATACTCCGATCACCAATGGTACCTCCCTCTCTGCTGC

ACTCCTGGACATGGGAATCCATAGTTTGAAAGTAGTTGCTTCAGCTCTTTGTGTTAGATT

ATTGTAACTGATTTTCCCTCCAAGGGCCTAACCTTGCCATTAACAAGCCCCAAATTCTCA

TGCCAGAGGTCTGAGAACTTTATGGGTTTGATCCTATCTTGTTGTGCTCAAGTCTTGTCT

CTGTCATCCATGGTCTCCTACGAAGTCATTGCCCTAAGTTCATGCTAGGGGAGCCAGAAG

GGAAGTCCTTGGATATCTTATACCTCAATATTGGCTCAATTTCTTGGGGAGGGGGTGCTG

TCAGAGATTGTTATCTGAGGATGTGACATAGATTTCTCAGGGCACAATTTCAACTACTTT

TTCAGCTTTAGGGTTTTTAGATACGTTTGTACCACAATTGAGCATGGGAGGGAGAGGGGT

GAGCCTAAGCAGTGATGGCTGATTTCTGTCACGTCTGTCATGTGTCCCCCAGTACCTCCA

GAGGTAACTGTGCTCACGAACAGCCCTGTGGAACTGAGAGAGCCCAACGTCCTCATCTGT

TTCATCGACAAGTTCACCCCACCAGTGGTCAATGTCACGTGGCTTCGAAATGGAAAACCT

GTCACCACAGGAGTGTCAGAGACAGTCTTCCTGCCCAGGGAAGACCACCTTTTCCGCAAG

TTCCACTATCTCCCCTTCCTGCCCTCAACTGAGGACGTTTACGACTGCAGGGTGGAGCAC

TGGGGCTTGGATGAGCCTCTTCTCAAGCACTGGGGTATGGACCAACACTCAATCTCCTTT

ATTTCAAGGTTTCCTCCTATGATGCTTGTGTGAAACTCGGTGTTCTAACTGTTTCATAAT

ATCTGCTACAATTAATATAACTGTCTTCTCCTACTATCCAGCTTCCTCCTTTTTTTAATC

TGTAATTCTCTCAATACATCATTCTGTCTTCCTCTTCTTTAATCTATGAATAACTTTTCT

CTTTATTAAGAACCCTACATTTGATTCTGAGTGTTACTTCTTCCCACACTCATTACCATG

TACTCTGCCTTATCTCCCCCCAGAGTTTGATGCTCCAAGCCCTCTCCCAGAGACTACAGA

GAACGTGGTGTGTGCCCTGGGCCTGACTGTGGGTCTGGTGGGCATCATTATTGGGACCAT

CTTCATCATCAAGGGAGTGCGCAAAAGCAATGCAGCAGAACGCAGGGGGCCTCTGTAAGG

CACATGGAGGTGAGTTAGGTGTGGTCAGAGGAAGACATATATGGAGATATCTGAGGGAGG

AAAACAGGGTGGGGAAAGGAAATGTAATGCATTTAAGAGACAAGGTAGGAACAGATGTGG

CTCTTGATTTCTCTTTGCTAGAATGAATCAGACATTGGTATCATCTGGTATCCCAAAGCT

TCAGGGTCTGTCATCCCTTTCTATAGACGGGCACCTTGATCACGGCTCCAGTCTTAGAAA

TCATCTCCAGTACCTAAAACCATTGTTTCACATTAGAATACTGAGTCTAGGGATCTAGAA

AATACATTAGAATATGGAGTCTAGGGATCTAGAAAATACTGAGTCTAGGGATCTAGAAAA

ATAAGCCTCAAGATTTGGGCACATCCTAGCTTGTATTTCCTGGGGCAGGTCATCAGTTCA

GAAGCATTTCCAGATCCTGGCTCCTTTCAGGTTAGGGTCAATTCATTGCATGAAATGGGA

ATCTCTTAGAGGCCAATGCCTGCTTTTGCTTCTTTAGTCTCAAATGTAGTATGAGAAACT

CTAAAAAAAGGTAAAGCATGGTTGCTTATTATGTTCAGTTGGAGAGTAGGAACTAACTGT

ATACAGTTAGTTCATGTTGGAAAGGTTAGATGAACATTGAAAGAATTTTGCAAAGTCAAA

GGATTAAGAGAGAAGAGGAAGGAATCTGAAGCAAGGAGCTCAAAACGGATCTTAAATTCC

TTGGTAACTATGTGTGTCTTGCTATAGGTGATGGTGTTTCTTAGAGAGAAGATCACTGAA

GAAACTTCTGCTTTAATGACTTTACAAAGCTGGCAATATTACAATCCTTGACCTCAGTGA

AAGCAGTCATCTTCAGCGTTTTCCAGCCCTATAGCCACCCCAAGTGTGGTTATGCCTCCT

CGATTGCTCCGTACTCTAACATCTAGCTGGCTTCCCTGTCTATTGCCTTTTCCTGTATCT

ATTTTCCTCTATTTCCTATCATTTTATTATCACCATGCAATGCCTCTGGAATAAAACATA

CAGGAGTCTGTCTCTGCTATGGAATGCCCCATGGGGCATCTCTTGTGTACTTATTGTTTA

AGGTTTCCTCAAACTGTGATTTTTCTGAACACAATAAACTATTTTGATGATCTTGGGTGG

AATTTTTGGTGTTTAAGCCAGTTCTTTGGGTGGCGGTGGGGGGTGGGGAGTCGGTCCTGG

GGAATATATGTGATCCTTTCCCGGTAAAATATCTGAATGTTGAATTTATCTTATAAATTC

TAGAATTCATCAGACATATCCCGGTTCATTTGGGCTTGGTCTCATTTTGTGCATCTGCAG

GCAACCCTCTTGTTGTGGTCTAGTCCTCATCAGGAAAACCTAAAGTGGGGTTGGTTTGTT

GGGAGATCTCTA

>HLA:HLA06470 DRA*01:01:01:03

TCACTAATGTGCTTCAGGTATATCCCTGTCTAGAAGTCAGATTGGGGTTAAAGAGTCTGT

CCGTGATTGACTAACAGTCTTAAATACTTGATTTGTTGTTGTTGTTGTCCTGTTTGTTTA

AGAACTTTACTTCTTTATCCAATGAACGGAGTATCTTGTGTCCTGGACCCTTTGCAAGAA

CCCTTCCCCTAGCAACAGATGCGTCATCTCAAAATATTTTTCTGATTGGCCAAAGAGTAA

TTGATTTGCATTTTAATGGTCAGACTCTATTACACCCCACATTCTCTTTTCTTTTATTCT

TGTCTGTTCTGCCTCACTCCCGAGCTCTACTGACTCCCAAAAGAGCGCCCAAGAAGAAAA

TGGCCATAAGTGGAGTCCCTGTGCTAGGATTTTTCATCATAGCTGTGCTGATGAGCGCTC

AGGAATCATGGGCTATCAAAGGTAGGTGCTGAGGGAATGAAATCTGGGACGATAGACTAC

GAAGCATTGGAGAAAAGACCTATGGACATTTGGAAGATAATGTGTGGAGTGAAAGAATAG

TGTGACAGGTATTATGTGGTCTCGACAGAAAGTATAACAAATTGTGGTTTGGTGGAGTTC

TTCCCTCACCACAAACTGAAGTAAGTCAAATTTGGTTTAGAGGGTCAAAACTGAGTTGTG

TATTGATGAATAGCACGGTCCTGCTACAAGCCAAACTGGGGGTGGGGGTGGGGGTGGGGG

AGGAAGAATATTTTCTGGCAAGCATTAACAAGTTATATTTCTGGGCTTTAATTATTCTTT

CTGGAAAATTAGTAAAATTAAAAACTAAAAACCACACATAGTTTTGCTAGAATTAAATGA

AAAAAAAAGTTATTAGCCCTGTTCTTATCTGAATACATGATACAGTAGTTATTTTTTGGA

GTGTAAATCCTGTCGGTATATATTGAGCACATATATTGTGTTGAAGATTACTAGAAGGAA

AAGTCATCAAAAAGCAACAATTTACCCCAGGAAAAGGGGAGGGAAGGCATGCTGATATGA

GTTGCCTCATGGGACAGTGATAGCCATTCCCTGCCTTCCCATCTCCATGGTACAGCAGAT

CTTATATCATGTTAACTTAGTAATATTTCCAAGAGAGTAGAAAAATAAGTAAGGAAATGG

GGAATCTGATATTATTGTCTCTCATCTCCAGAGCAACATTGGTGCTGTTGTAAAGATGTA

CTGTAGAAAAGTATTCTTCACCCAGCGTGACCCCCACAGAAGGTGTCAGGTAGACTTGAA

ATAAGCAAAGTAATAACCCAGCTCCCATACCCATAGTGGCAATTGTAGATTTCTATTGCC

CCAAAAGAGCCATACATAGGGATACTTACCTAGAAAGACAGAGGATCTTCCCTTGGTTTG

TGAAGAGGCAGCTAGTATATTTGTGTGTGTTTGCATAGATGCAAACGGTAAATAAATTCC

TAGGTTTATCAATACACAGTCAAACATTAAAATCTCTCATCTTGGCTGGGCACGGTGGCT

CACGCCTGTAATCCCAGCACTTTGGGAGGCCGAGGCGGGCGGATCACGAGGTCAAGAGAT

CGAGACCGTCCTGGGCAACATGGTGAAACCCCGTCTCTACTAAAAATACAAAAAATTAGC

TGGGTATGGTGGCACACGCCTGTAGTCCCAGCTACTCGGGAGGCTGAGGCAGGAGGATTG

CTTGAGCCCGGGAGGCGGAGGTTGCAGTGAGCTGAGATGGTGCCACTGCACTCCAGCCTG

GCGATAGAGCAAGACTCCGTCTCAAACAACCAAACCAAAACAAAACAAAATATCTCACCT

TATCTTTGAAGACTAAGGAAAAAAAAAATCTCCCACTCATCGATACACTCCACAGAGGCA

GCATACTCTCCCAGTGTAGCTTTCTCTTTTCATGTTCATTATTCCCTTGGTGTTGGTTAT

TCTCAATGTCAATCGTAACAGAACATCTTCCATAATAACAGTCCCAATTTAAGGAGCATT

AAGATAAAAGGTGGAATTGCCAAGGTCAATCCAGACGAGAACCTTCTCATAGAGGTAACC

ACCGTGTGGGTTTGGATGCTGGGAAGCAGGGGGACTATGACGCTACAAGGTCTCAGTCTT

AATTTTTGGAGTACTTCAGTCCCCAGGTATATTTTCCATAGATTTGGCCCTTAAATAAAA

AGAAGCTTCTGACTCTAAAATGTAAACAGTGCTTGTTACAGTCTTGTTGATATATTAAGA

AATTACTCACCTTATCTCATTTAATCTTAAAAACAAACCCCTGACAGGATCAAAACCACA

GCAGGACTACATAATAGGAAAACTATACATAAATAGGTAGAATAATCTGCTCAGGATCAC

TAGGTAAGTTGCTGAATAAGAATTCAAGATGTTTTTGATCCCAGAGTTTAAAACCCAACC

TTTCAAACAGTGTTTCCTTCTTCTTAGAGTACAATGTTCTGAGAAAGAGATCCTCTGGAA

TTCTGGCCTAAGTGTATTTAATGCCCGGGTAAAGAAAGTGAGAGAACATTTCTCTTTAGG

GGCTGCTGCTGGATTTCTAAAAAGAAAATAATTTCTCAGCTAGTAACATGGAGCCAAACA

ACAGCTTCACAAGACTCTGGGTTCTTTAGCCCTCATCTCCTTCAATCCACCCTCTTTATA

ACCAGTCCTTCTTGTTTTTCCCCTCCCAGCTTTGTTCAGCAGCATGCCCTTCACCCAGAC

CTTGTCTTGTCACTCATCCCTACTCGCCATCATTCTTTCATTCCTCTTGGCCCAATCTCT

CTCCACCACTTCCTGCCTACATGTATGTAGGTTATTCATTTCCCTCTCTTGATTCCCCCC

ACCCAACTCTCTTTCTCCATTTCTTGCCTTTCAGAAGAACATGTGATCATCCAGGCCGAG

TTCTATCTGAATCCTGACCAATCAGGCGAGTTTATGTTTGACTTTGATGGTGATGAGATT

TTCCATGTGGATATGGCAAAGAAGGAGACGGTCTGGCGGCTTGAAGAATTTGGACGATTT

GCCAGCTTTGAGGCTCAAGGTGCATTGGCCAACATAGCTGTGGACAAAGCCAACCTGGAA

ATCATGACAAAGCGCTCCAACTATACTCCGATCACCAATGGTACCTCCCTCTCTGCTGCA

CTCCTGGACATGGGAATCCATAGTTTGAAAGTAGTTGCTTCAGCTCTTTGTGTTAGATTA

TTGTAACTGATTTTCCCTCCAAGGGCCTAACCTTGCCATTAACAAGCCCCAAATTCTCAT

GCCAGAGGTCTGAGAACTTTATGGGTTTGATCCTATCTTGTTGTGCTCAAGTCTTGTCTC

TGTCATCCATGGTCTCCTACGAAGTCATTGCCCTAAGTTCATGCTAGGGGAGCCAGAAGG

GAAGTCCTTGGATATCTTATACCTCAATATTGGCTCAATTTCTTGGGGAGGGGGTGCTGT

CAGAGATTGTTATCTGAGGATGTGACATAGATTTCTCAGGGCACAATTTCAACTACTTTT

TCAGCTTTAGGGTTTTTAGATACGTTTGTACCACAATTGAGCATGGGAGGGAGAGGGGTG

AGCCTAAGCAGTGATGGCTGATTTCTGTCACGTCTGTCATGTGTCCCCCAGTACCTCCAG

AGGTAACTGTGCTCACGAACAGCCCTGTGGAACTGAGAGAGCCCAACGTCCTCATCTGTT

TCATCGACAAGTTCACCCCACCAGTGGTCAATGTCACGTGGCTTCGAAATGGAAAACCTG

TCACCACAGGAGTGTCAGAGACAGTCTTCCTGCCCAGGGAAGACCACCTTTTCCGCAAGT

TCCACTATCTCCCCTTCCTGCCCTCAACTGAGGACGTTTACGACTGCAGGGTGGAGCACT

GGGGCTTGGATGAGCCTCTTCTCAAGCACTGGGGTATGGACCAACACTCAATCTCCTTTA

TTTCAAGGTTTCCTCCTATGATGCTTGTGTGAAACTCGGTGTTCTAACTGTTTCATAATA

TCTGCTACAATTAATATAACTGTCTTCTCCTACTATCCAGCTTCCTCCTTTTTTTAATCT

GTAATTCTCTCAATACATCATTCTGTCTTCCTCTTCTTTAATCTATGAATAACTTTTCTC

TTTATTAAGAACCCTACATTTGATTCTGAGTGTTACTTCTTCCCACACTCATTACCATGT

ACTCTGCCTTATCTCCCCCCAGAGTTTGATGCTCCAAGCCCTCTCCCAGAGACTACAGAG

AACGTGGTGTGTGCCCTGGGCCTGACTGTGGGTCTGGTGGGCATCATTATTGGGACCATC

TTCATCATCAAGGGAGTGCGCAAAAGCAATGCAGCAGAACGCAGGGGGCCTCTGTAAGGC

ACATGGAGGTGAGTTAGGTGTGGTCAGAGGAAGACATATATGGAGATATCTGAGGGAGGA

AAACAGGGTGGGGAAAGGAAATGTAATGCATTTAAGAGACAAGGTAGGAACAGATGTGGC

TCTTGATTTCTCTTTGCTAGAATGAATCAGACATTGGTATCATCTGGTATCCCAAAGCTT

CAGGGTCTGTCATCCCTTTCTATAGACGGGCACCTTGATCACGGCTCCAGTCTTAGAAAT

CATCTCCAGTACCTAAAACCATTGTTTCACATTAGAATACTGAGTCTAGGGATCTAGAAA

ATACATTAGAATATGGAGTCTAGGGATCTAGAAAATACTGAGTCTAGGGATCTAGAAAAA

TAAGCCTCAAGATTTGGGCACATCCTAGCTTGTATTTCCTGGGGCAGGTCATCAGTTCAG

AAGCATTTCCAGATCCTGGCTCCTTTCAGGTTAGGGTCAATTCATTGCATGAAATGGGAA

TCTCTTAGAGGCCAATGCCTGCTTTTGCTTCTTTAGTCTCAAATGTAGTATGAGAAACTC

TAAAAAAAGGTAAAGCATGGTTGCTTATTATGTTCAGTTGGAGAGTAGGAACTAACTGTA

TACAGTTAGTTCATGTTGGAAAGGTTAGATGAACATTGAAAGAATTTTGCAAAGTCAAAG

GATTAAGAGAGAAGAGGAAGGAATCTGAAGCAAGGAGCTCAAAACGGATCTTAAATTCCT

TGGTAACTATGTGTGTCTTGCTATAGGTGATGGTGTTTCTTAGAGAGAAGATCACTGAAG

AAACTTCTGCTTTAATGACTTTACAAAGCTGGCAATATTACAATCCTTGACCTCAGTGAA

AGCAGTCATCTTCAGCGTTTTCCAGCCCTATAGCCACCCCAAGTGTGGTTATGCCTCCTC

GATTGCTCCGTACTCTAACATCTAGCTGGCTTCCCTGTCTATTGCCTTTTCCTGTATCTA

TTTTCCTCTATTTCCTATCATTTTATTATCACCATGCAATGCCTCTGGAATAAAACATAC

AGGAGTCTGTCTCTGCTATGGAATGCCCCATGGGGCATCTCTTGTGTACTTATTGTTTAA

GGTTTCCTCAAACTGTGATTTTTCTGAACACAATAAACTATTTTGATGATCTTGGGTGGA

ATTTTTGGTGTTTAAGCCAGTTCTTTGGGTGGCGGTGGGGGGTGGGGAGTCGGTCCTGGG

GAATATATGTGATCCTTTCCCGGTAAAATATCTGAATGTTGAATTTATCTTATAAATTCT

AGAATTCATCAGACATATCCCGGTTCATTTGGGCTTGGTCTCATTTTGTGCATCTGCAGG

CAACCCTCTTGTTGTGGTCTAGTCCTCATCAGGAAAACCTAAAGTGGGGTTGGTTTGTTG

GGAGATCTCTA

>HLA:HLA25564 DRA*01:01:01:05

TCACTAATGTGCTTCAGGTATATCCCTGTCTAGAACTCAGATTGGGGTTAAAGAGTCTGT

CCGTCATTGACCAACAGTCTTAAATACTTGATTTGTTGTCGTTGTTGTCCTGTTTGTTTA

AGAACTTTACTTCTTTATCCAATGAACGGAGTATCTTGTGTCCTGGACCCTTTGCAAGAA

CCCTTCCCCTAGCAACAGATGCGTCATCTCAAAATATTTTTCTGATTGGCCAAAGAGTAA

TTGATTTGCATTTTAATGGTCAGACTCTATTACACCCCACATTCTCTTTTCTTTTATTCT

TGTCTGTTCTGCCTCACTCCCGAGCTCTACTGACTCCCAACAGAGCGCCCAAGAAGAAAA

TGGCCATAAGTGGAGTCCCTGTGCTAGGATTTTTCATCATAGCTGTGCTGATGAGCGCTC

AGGAATCATGGGCTATCAAAGGTAGGTGCTGAGGGAATGAAATCTGGGACGATAGACTAC

GAAGCATTGGAGAAAAGACCTATGGACATTTGGAAGATAATGTGTGGAGTGAAAGAATAG

TGTGACAGGTATTATGTGGTCTCGACAGAAAGTATAACAAATTGTGGTTTGGTGGAGTTC

TTCCCTCACCACAAACTGAAGTAAGTCAAATTTGGTTTAGAGGATCAAAACTGAGTTGTG

TATTGATGAATAGCAAGGTCCTGCTACAAGCCAAACTGGGGGTGGGGGTGGGGGTGGGGG

AGGAAGAATATTTTCTGGCAAGCATTAACAAGTTATATTTCTGGGCTTTAATTATTCTTT

CTGGAAAATTAGTAAAATTAAAAACTAAAAACCACACATAGTTTTGCTAGAATTAAATGA

AAAAAAAAGTTATTAGCCCTGTTCTTATCTGAATACATGATACAGTAGTTATTTTTTGGA

GTGTAAATCCTGTCGGTATATATTGAGCACATATATTGTGTTGAAGATTACTAGAAGGAA

AAGTCATCAAAAAGCAACAATTTACCCCAGGAAAAGGGGAGGGAAGGCATGCTGATATGA

GTTGCCTCATGGGACAGTGATAGCCATTCCCTGCCTTCCCATCTCCATGGTACAGCAGAT

CTTATATCATGTTAACTTAGTAATATTTCCAAGAGAGTAGAAAAATAAGTAAGGAAATGG

GGAATCTGATATTATTCTCTCTCATCTCCAGAGCAACATTGGTGCTGTTGTAAAGATGTA

CTGTAGAAAAGTATTCTTCACCCAGCATGACCCCCACAGAAGGTGTCAGGTAGACTTGAA

ATAAGCAAAGTAATAACCCAGCTCCCATACCCATAGTGGCAATTGTAGATTTCTATTGCC

CCAAAAGAGCCATACATAGGGATACTTACCTAGAAAGACAGAGGCTCTTCCCTTGGTTTG

TGAAGAGGCAGCTAGTATATTTGTGTGTGTTTGCATAGATGCAAACGGTAAATAAATTCC

TAGGTTTATCAATACACAGTCAAACATTAAAATCTCTCATCTTGGCTGGGCACGGTGGCT

CACGCCTGTAATCCCAGCACTTTGGGAGGCCGAGGCAGGCGGATCACGAGGTCAAGAGAT

CGAGACCGTCCTGGGCAACATGGTGAAACCCCGTCTCTACTAAAAATACAAAAAATTAGC

TGGGTATGGTGGCACACGCCTGTAGTCCCAGCTACTCGGGAGGCTGAGGCAGGAGGATTG

CTTGAGCCCGGGAGGCGGAGGTTGCAGTGAGCTGAGATGGTGCCACTGCACTCCAGCCTG

GCGATAGAGCAAGACTCCGTCTCAAACAACCAAACCAAAACAAAACAAAATATCTCACCT

TATCTTTGAAGACTAAGGAAAAAAAAATCTCCCACTCATCGATACACTCCACAGAGGCAG

CATACTCTCCAAGTGTAGCTTTCTCTTTTCATGTTCATTATTCCCTTGGTGTTGGTTATT

CTCAATGTCAATCATAACAGAACATCTTCCATAATAACAGTCCCAATTTAAGGAGCATTA

AGATAAAAGGTGGAATTGCCAAGGTCAATCCAGACGAGAACCTTCTCATAGAGGTAACCA

CCGTGTGGGTTTGGATGCTGGGAAGCAGGGGGACTATGACGCTACAAGGTCTCAGTCTTA

ATTTTTGGAGTATTTCAGTCCCCAGGTATATTTTCCATAGATTTGGCCCTTAAATAAAAA

GAAGCTTCTGACTCTAAAATGTAAACAGTGCTTGTTACAGTCTTGTTGATATATTAAGAA

ATTACTCACCTTATCTCATTTAATCTTAAAAACAAACCCCTGACAGGATCAAAACCACAG

CAGGGCTACATAATAGGAAAACTATACATAAATAGGTAGAATAATCTGCTCAGGATTACT

AGGTAAGTTGCTGAATAAGAATTCAAGATGTTTTTGATCCCAGAGTTTAAAACCCAACCT

TTCAAACAGCGTTTCTTTCTTCTTAGAGTACAATGTTCTGAGAAAGAGATCCTCTGGAAT

TCTGGCCTAAGTGTATTTAATGCCCGGGTAAAGAAAGTGAGAGAACATTTCTCTTTAGGG

GCTGCTGCTGGATTTCTAAAAAGAAAATAATTTCTCAGCTAGTAACATGGAGCCAAACAA

CAGCTTCACAAGACTCTGGGTTCTTTAGCCCTCATCTCCTTCAATCCACCCTCTTTATAA

CCAGTCCTTCTTGTTTTTCCCCTCCCAGCTTTGTTCAGCAGCATGCCCTTCACCCAGACC

TTGTCTTGTCAATCATCCCTACTCGCCATCATTCTTTCATTCCTCTTGGCCCAATCTCTC

TCCACCACTTCCTGCCTACATGTATGTAGTTATTCATTTCCCTCTCTTGATTCCCCCCAC

CCAACTCTCTTTCTCCATTTCTTGCCTTTCAGAAGAACATGTGATCATCCAGGCCGAGTT

CTATCTGAATCCTGACCAATCAGGCGAGTTTATGTTTGACTTTGATGGTGATGAGATTTT

CCATGTGGATATGGCAAAGAAGGAGACGGTCTGGCGGCTTGAAGAATTTGGACGATTTGC

CAGCTTTGAGGCTCAAGGTGCATTGGCCAACATAGCTGTGGACAAAGCCAACCTGGAAAT

CATGACAAAGCGCTCCAACTATACTCCGATCACCAATGGTACCTCCCTCTCTGCTGCACT

CCTGGACATGGGAATCCATAGTTTGAAAGTAGTTGCTTCAGCTCTTTGTGTTAGATTATT

GTAACTGATTTTCCCTCCAAGGGCCTAACCTTGCCATTAACAAGCCCCAAATTCTCATGC

CAGAGGTCTGAGAACTTTATGGGTTTGATCCTATCTTGTTGTGCTCAAGTCTTGTCTCTG

TCATCCATGGTCTCCTACGAAGTCATTGCCCTAAGTTCATGCTGGGGGAGCCAGAAGGGA

AGTCCTTGGATATCTTATACCTCAATATTGGCTCAATTTCTTGGGGAGGGGGTGCTGTCA

GAGATTGTTATCTGAGGATGTGACATAGATTTCTCAGGGCACAATTTCAACTACTTTTTC

AGCTTTAGGGTTTTTAGATACGTTTGTACCACAATTGAGCATGGGAGGGAGAGGGGTGAG

CCTAAGCAGTGATGGCTGATTTCTGTCACGTCTGTCATGTGTCCCCCAGTACCTCCAGAG

GTAACTGTGCTCACGAACAGCCCTGTGGAACTGAGAGAGCCCAACGTCCTCATCTGTTTC

ATCGACAAGTTCACCCCACCAGTGGTCAATGTCACGTGGCTTCGAAATGGAAAACCTGTC

ACCACAGGAGTGTCAGAGACAGTCTTCCTGCCCAGGGAAGACCACCTTTTCCGCAAGTTC

CACTATCTCCCCTTCCTGCCCTCAACTGAGGACGTTTACGACTGCAGGGTGGAGCACTGG

GGCTTGGATGAGCCTCTTCTCAAGCACTGGGGTATGGACCAACACTCAATCTCCTTTATT

TCAAGGTTTCCTCCTATGATGCTTGTGTGAAACTCGGTGTTCTAACTGTTTCATAATATC

TGCTACAATTAATATAACTGTCTTCTCCTCCTATCCAGCTTCCTCCTTTTTTTAATCTGT

AATTCTCTCAATACATCATTCTGTCTTCCTCTTCTTTAATCTATGAATAACTTTTCTCTT

TATTAAGAACCCTACATTTGATTCTGAGTGTTACTTCTTCCCACACTCATTACCATGTAC

TCTGCCTTATTTCCCCCCAGAGTTTGATGCTCCAAGCCCTCTCCCAGAGACTACAGAGAA

CGTGGTGTGTGCCCTGGGCCTGACTGTGGGTCTGGTGGGCATCATTATTGGGACCATCTT

CATCATCAAGGGAGTGCGCAAAAGCAATGCAGCAGAACGCAGGGGGCCTCTGTAAGGCAC

ATGGAGGTGAGTTAGGTGTGGTCAGAGGAAGACATATATGGAGATATCTGAGGGAGGAAA

ACAGGGTGGGGAAAGGAAATGTAATGCATTTAAGAGACAAGGTAGGAACAGATGTGGCTC

TTGATTTCTCTTTGCTAGAACGAATCAGACATTGGTATCATCTGGTATCCCAAAGCTTCA

GGGTCTGTCATCCCTTTCTATAGACGGGCACCTTGATCACGGCTCCAGTCTTAGAAATCA

TCTCCAGTACCTAAAACCATTGTTTCACATTAGAATACTGAGTCTAGGGATCTAGAAAAT

ACATTAGAATATGGAGTCTAGGGATCTAGAAAATACTGAGTCTAGGGATCTAGAAAAATA

AGCCTCAAGATTTGGGCACATCCTAGCTTGTATTTCCTGGGGCAGGTCATCAGTTCAGAA

GCATTTCCAGATCCTGGCTCCTTTCAGGTTAGGGTCAATTCATTGCATGAAATGGGAATC

TCTTAGAGGCCAATGCCTGCTTTTGCTTCTTTAGTCTCAAATGTAGTATGAGAAACTCTA

AAAAAAGGTAAAGCATGGTTGCTTATTATGTTCAGTTGGAGAGTAGGAACTAACTGTATA

CAGTTAGTTCATGTTGGAAAGGTTAGATGAACATTGAAAGAATTTTGCAAAGTCAAAGGA

TTAAGAGAGAAGAGGAAGGAATCTGAAGCAAGGAGCTCAAAACAGATCTTAAATTCCTTG

GTAACTATGTGTGTCTTGCTATAGGTGATGATGTTTCTTAGAGAGAAGATCACTGAAGAA

ACTTCTGCTTTAATGACTTTACAAAGCTGGCAATATTACAATCCTTGACCTCAGTGAAAG

CAGTCATCTTCAGCGTTTTCCAGCCCTATAGCCACCCCAAGTGTGGTTATGCCTCCTCGA

TTGCTCCGTACTCTAACATCTAGCTGGCTTCCCTGTCTATTGCCTTTTCCTGTATCTATT

TTCCTCTATTTCCTATCATTTTATTATCACCATGCAATGCCTCTGGAATAAAACATACAG

GAGTCTGTCTCTGCTATGGAATGCCCCATGGGGCATCTCTTGTGTACTTATTGTTTAAGG

TTTCCTCAAACTGTGATTTTTCTGAACACAATAAACTATTTTGATGATCTTGGGTGGAAT

TTTTGGTGTTTAAGCCAGTTCTTTGGGTGGCGGTGGGGGGTGGGGAGTCGGTCCTGGGGA

ATATATGTGATCCTTTCCCGGTAAAATATCTGAATGTTGAATTTATCTTATAAATTCTAG

AATTCATCAGACATATCCCGGTTCATTTGGGCTTGGTCTCATTTTGTGCATCTGCAGGCA

ACCCTCTTGTTGTGGTCTAGTCCTCATCAGGAAAACCTAAAGTGGGGTTGGTTTGTTGGG

AGATCTCTA

>HLA:HLA25847 DRA*01:01:01:07

TCACTAATGTGCTTCAGGTATATCCCTGTCTAGAAGTCAGATTGGGGTTAAAGAGTCTGT

CCGTGATTGACTAACAGTCTTAAATACTTGATTTGTTGTTGTTGTTGTCCTGTTTGTTTA

AGAACTTTACTTCTTTATCCAATGAACGGAGTATCTTGTGTCCTGGACCCTTTGCAAGAA

CCCTTCCCCTAGCAACAGATGCGTCATCTCAAAATATTTTTCTGATTGGCCAAAGAGTAA

TTGATTTGCATTTTAATGGTCAGACTCTATTACACCCCACATTCTCTTTTCTTTTATTCT

TGTCTGTTCTGCCTCACTCCCGAGCTCTACTGACTCCCAAAAGAGCGCCCAAGAAGAAAA

TGGCCATAAGTGGAGTCCCTGTGCTAGGATTTTTCATCATAGCTGTGCTGATGAGCGCTC

AGGAATCATGGGCTATCAAAGGTAGGTGCTGAGGGAATGAAATCTGGGACGATAGACTAC

GAAGCATTGGAGAAAAGACCTATGGACATTTGGAAGATAATGTGTGGAGTGAAAGAACAG

TGTGACAGGTATTATGTGGTCTCGACAGAAAGTATAACAAATTGTGGTTTGGTGGAGTTC

TTCCCTCACCACAAACTGAAGTAAGTCAAATTTGGTTTAGAGGGTCAAAACTGAGTTGTG

TATTGATGAATAGCACGGTCCTGCTACAAGCCAAACTGGGGGTGGGGGTGGGGGTGGGGG

AGGAAGAATATTTTCTGGCAAGCATTAACAAGTTATATTTCTGGGCTTTAATTATTCTTT

CTGGAAAATTAGTAAAATTAAAAACTAAAAACCACACATAGTTTTGCTAGAATTAAATGA

AAAAAAAAGTTATTAGCCCTGTTCTTATCTGAATACATGATACAGTAGTTATTTTTTGGA

GTGTAAATCCTGTCGGTATATATTGAGCACATATATTGTGTTGAAGATTACTAGAAGGAA

AAGTCATCAAAAAGCAACAATTTACCCCAGGAAAAGGGGAGGGAAGGCATGCTGATATGA

GTTGCCTCATGGGACAGTGATAGCCATTCCCTGCCTTCCCATCTCCATGGTACAGCAGAT

CTTATATCATGTTAACTTAGTAATATTTCCAAGAGAGTAGAAAAATAAGTAAGGAAATGG

GGAATCTGATATTATTGTCTCTCATCTCCAGAGCAACATTGGTGCTGTTGTAAAGATGTA

CTGTAGAAAAGTATTCTTCACCCAGCGTGACCCCCACAGAAGGTGTCAGGTAGACTTGAA

ATAAGCAAAGTAATAACCCAGCTCCCATACCCATAGTGGCAATTGTAGATTTCTATTGCC

CCAAAAGAGCCATACATAGGGATACTTACCTAGAAAGACAGAGGATCTTCCCTTGGTTTG

TGAAGAGGCAGCTAGTATATTTGTGTGTGTTTGCATAGATGCAAACGGTAAATAAATTCC

TAGGTTTATCAATACACAGTCAAACATTAAAATCTCTCATCTTGGCTGGGCACGGTGGCT

CACGCCTGTAATCCCAGCACTTTGGGAGGCCGAGGCGGGCGGATCACGAGGTCAAGAGAT

CGAGACCGTCCTGGGCAACATGGTGAAACCCCGTCTCTACTAAAAATACAAAAAATTAGC

TGGGTATGGTGGCACACGCCTGTAGTCCCAGCTACTCGGGAGGCTGAGGCAGGAGGATTG

CTTGAGCCCGGGAGGCGGAGGTTGCAGTGAGCTGAGATGGTGCCACTGCACTCCAGCCTG

GCGATAGAGCAAGACTCCGTCTCAAACAACCAAACCAAAACAAAACAAAATATCTCACCT

TATCTTTGAAGACTAAGGAAAAAAAAAATCTCCCACTCATCGATACACTCCACAGAGGCA

GCATACTCTCCCAGTGTAGCTTTCTCTTTTCATGTTCATTATTCCCTTGGTGTTGGTTAT

TCTCAATGTCAATCGTAACAGAACATCTTCCATAATAACAGTCCCAATTTAAGGAGCATT

AAGATAAAAGGTGGAATTGCCAAGGTCAATCCAGACGAGAACCTTCTCATAGAGGTAACC

ACCGTGTGGGTTTGGATGCTGGGAAGCAGGGGGACTATGACGCTACAAGGTCTCAGTCTT

AATTTTTGGAGTACTTCAGTCCCCAGGTATATTTTCCATAGATTTGGCCCTTAAATAAAG

AGAAGCTTCTGACTCTAAAATGTAAACAGTGCTTGTTACAGTCTTGTTGATATATTAAGA

AATTACTCACCTTATCTCATTTAATCTTAAAAACAAACCCCTGACAGGATCAAAACCACA

GCAGGACTACATAATAGGAAAACTATACATAAATAGGTAGAATAATCTGCTCAGGATCAC

TAGGTAAGTTGCTGAATAAGAATTCAAGATGTTTTTGATCCCAGAGTTTAAAACCCAACC

TTTCAAACAGTGTTTCCTTCTTCTTAGAGTACAATGTTCTGAGAAAGAGATCCTCTGGAA

TTCTGGCCTAAGTGTATTTAATGCCCGGGTAAAGAAAGTGAGAGAACATTTCTCTTTAGG

GGCTGCTGCTGGATTTCTAAAAAGAAAATAATTTCTCAGCTAGTAACATGGAGCCAAACA

ACAGCTTCACAAGACTCTGGGTTCTTTAGCCCTCATCTCCTTCAATCCACCCTCTTTATA

ACCAGTCCTTCTTGTTTTTCCCCTCCCAGCTTTGTTCAGCAGCATGCCCTTCACCCAGAC

CTTGTCTTGTCACTCATCCCTACTCGCCATCATTCTTTCATTCCTCTTGGCCCAATCTCT

CTCCACCACTTCCTGCCTACATGTATGTAGGTTATTCATTTCCCTCTCTTGATTCCCCCC

ACCCAACTCTCTTTCTCCATTTCTTGCCTTTCAGAAGAACATGTGATCATCCAGGCCGAG

TTCTATCTGAATCCTGACCAATCAGGCGAGTTTATGTTTGACTTTGATGGTGATGAGATT

TTCCATGTGGATATGGCAAAGAAGGAGACGGTCTGGCGGCTTGAAGAATTTGGACGATTT

GCCAGCTTTGAGGCTCAAGGTGCATTGGCCAACATAGCTGTGGACAAAGCCAACCTGGAA

ATCATGACAAAGCGCTCCAACTATACTCCGATCACCAATGGTACCTCCCTCTCTGCTGCA

CTCCTGGACATGGGAATCCATAGTTTGAAAGTAGTTGCTTCAGCTCTTTGTGTTAGATTA

TTGTAACTGATTTTCCCTCCAAGGGCCTAACCTTGCCATTAACAAGCCCCAAATTCTCAT

GCCAGAGGTCTGAGAACTTTATGGGTTTGATCCTATCTTGTTGTGCTCAAGTCTTGTCTC

TGTCATCCATGGTCTCCTACGAAGTCATTGCCCTAAGTTCATGCTAGGGGAGCCAGAAGG

GAAGTCCTTGGATATCTTATACCTCAATATTGGCTCAATTTCTTGGGGAGGGGGTGCTGT

CAGAGATTGTTATCTGAGGATGTGACATAGATTTCTCAGGGCACAATTTCAACTACTTTT

TCAGCTTTAGGGTTTTTAGATACGTTTGTACCACAATTGAGCATGGGAGGGAGAGGGGTG

AGCCTAAGCAGTGATGGCTGATTTCTGTCACGTCTGTCATGTGTCCCCCAGTACCTCCAG

AGGTAACTGTGCTCACGAACAGCCCTGTGGAACTGAGAGAGCCCAACGTCCTCATCTGTT

TCATCGACAAGTTCACCCCACCAGTGGTCAATGTCACGTGGCTTCGAAATGGAAAACCTG

TCACCACAGGAGTGTCAGAGACAGTCTTCCTGCCCAGGGAAGACCACCTTTTCCGCAAGT

TCCACTATCTCCCCTTCCTGCCCTCAACTGAGGACGTTTACGACTGCAGGGTGGAGCACT

GGGGCTTGGATGAGCCTCTTCTCAAGCACTGGGGTATGGACCAACACTCAATCTCCTTTA

TTTCAAGGTTTCCTCCTATGATGCTTGTGTGAAACTCGGTGTTCTAACTGTTTCATAATA

TCTGCTACAATTAATATAACTGTCTTCTCCTACTATCCAGCTTCCTCCTTTTTTTAATCT

GTAATTCTCTCAATACATCATTCTGTCTTCCTCTTCTTTAATCTATGAATAACTTTTCTC

TTTATTAAGAACCCTACATTTGATTCTGAGTGTTACTTCTTCCCACACTCATTACCATGT

ACTCTGCCTTATCTCCCCCCAGAGTTTGATGCTCCAAGCCCTCTCCCAGAGACTACAGAG

AACGTGGTGTGTGCCCTGGGCCTGACTGTGGGTCTGGTGGGCATCATTATTGGGACCATC

TTCATCATCAAGGGAGTGCGCAAAAGCAATGCAGCAGAACGCAGGGGGCCTCTGTAAGGC

ACATGGAGGTGAGTTAGGTGTGGTCAGAGGAAGACATATATGGAGATATCTGAGGGAGGA

AAACAGGGTGGGGAAAGGAAATGTAATGCATTTAAGAGACAAGGTAGGAACAGATGTGGC

TCTTGATTTCTCTTTGCTAGAATGAATCAGACATTGGTATCATCTGGTATCCCAAAGCTT

CAGGGTCTGTCATCCCTTTCTATAGACGGGCACCTTGATCACGGCTCCAGTCTTAGAAAT

CATCTCCAGTACCTAAAACCATTGTTTCACATTAGAATACTGAGTCTAGGGATCTAGAAA

ATACATTAGAATATGGAGTCTAGGGATCTAGAAAATACTGAGTCTAGGGATCTAGAAAAA

TAAGCCTCAAGATTTGGGCACATCCTAGCTTGTATTTCCTGGGGCAGGTCATCAGTTCAG

AAGCATTTCCAGATCCTGGCTCCTTTCAGGTTAGGGTCAATTCATTGCATGAAATGGGAA

TCTCTTAGAGGCCAATGCCTGCTTTTGCTTCTTTAGTCTCAAATGTAGTATGAGAAACTC

TAAAAAAAGGTAAAGCATGGTTGCTTATTATGTTCAGTTGGAGAGTAGGAACTAACTGTA

TACAGTTAGTTCATGTTGGAAAGGTTAGATGAACATTGAAAGAATTTTGCAAAGTCAAAG

GATTAAGAGAGAAGAGGAAGGAATCTGAAGCAAGGAGCTCAAAACGGATCTTAAATTCCT

TGGTAACTATGTGTGTCTTGCTATAGGTGATGGTGTTTCTTAGAGAGAAGATCACTGAAG

AAACTTCTGCTTTAATGACTTTACAAAGCTGGCAATATTACAATCCTTGACCTCAGTGAA

AGCAGTCATCTTCAGCGTTTTCCAGCCCTATAGCCACCCCAAGTGTGGTTATGCCTCCTC

GATTGCTCCGTACTCTAACATCTAGCTGGCTTCCCTGTCTATTGCCTTTTCCTGTATCTA

TTTTCCTCTATTTCCTATCATTTTATTATCACCATGCAATGCCTCTGGAATAAAACATAC

AGGAGTCTGTCTCTGCTATGGAATGCCCCATGGGGCATCTCTTGTGTACTTATTGTTTAA

GGTTTCCTCAAACTGTGATTTTTCTGAACACAATAAACTATTTTGATGATCTTGGGTGGA

ATTTTTGGTGTTTAAGCCAGTTCTTTGGGTGGCGGTGGGGGGTGGGGAGTCGGTCCTGGG

GAATATATGTGATCCTTTCCCGGTAAAATATCTGAATGTTGAATTTATCTTATAAATTCT

AGAATTCATCAGACATATCCCGGTTCATTTGGGCTTGGTCTCATTTTGTGCATCTGCAGG

CAACCCTCTTGTTGTGGTCTAGTCCTCATCAGGAAAACCTAAAGTGGGGTTGGTTTGTTG

GGAGATCTCTA

>HLA:HLA06468 DRA*01:01:02

TCACTAATGTGCTTCAGGTATATCCCTGTCTAGAAGTCAGATTGGGGTTAAAGAGTCTGT

CCGTGATTGACTAACAGTCTTAAATACTTGATTTGTTGTTGTTGTTGTCCTGTTTGTTTA

AGAACTTTACTTCTTTATCCAATGAACGGAGTATCTTGTGTCCTGGACCCTTTGCAAGAA

CCCTTCCCCTAGCAACAGATGCGTCATCTCAAAATATTTTTCTGATTGGCCAAAGAGTAA

TTGATTTGCATTTTAATGGTCAGACTCTATTACACCCCACATTCTCTTTTCTTTTATTCT

TGTCTGTTCTGCCTCACTCCCGAGCTCTACTGACTCCCAACAGAGCGCCCAAGAAGAAAA

TGGCCATAAGTGGAGTCCCTGTGCTAGGATTTTTCATCATAGCTGTGCTGATGAGCGCTC

AGGAATCATGGGCTATCAAAGGTAGGTGCTGAGGGAATGAAATCTGGGACGATAGACTAC

GAAGCATTGGAGAAAAGACCTATGGACATTTGGAAGATAATGTGTGGAGTGAAAGAATAG

TGTGACAGGTATTATGTGGTCTCGACAGAAAGTATAACAAATTGTGGTTTGGTGGAGTTC

TTCCCTCACCACAAACTGAAGTAAGTCAAATTTGGTTTAGAGGGTCAAAACTGAGTTGTG

TATTGATGAATAGCACGGTCCTGCTACAAGCCAAACTGGGGGTGGGGGTGGGGGTGGGGG

AGGAAGAATATTTTCTGGCAAGCATTAACAAGTTATATTTCTGGGCTTTAATTATTCTTT

CTGGAAAATTAGTAAAATTAAAAACTAAAAACCACACATAGTTTTGCTAGAATTAAATGA

AAAAAAAAGTTATTAGCCCTGTTCTTATCTGAATACATGATACAGTAGTTATTTTTTGGA

GTGTAAATCCTGTCGGTATATATTGAGCACATATATTGTGTTGAAGATTACTAGAAGGAA

AAGTCATCAAAAAGCAACAATTTACCCCAGGAAAAGGGGAGGGAAGGCATGCTGATATGA

GTTGCCTCATGGGACAGTGATAGCCATTCCCTGCCTTCCCATCTCCATGGTACAGCAGAT

CTTATATCATGTTAACTTAGTAATATTTCCAAGAGAGTAGAAAAATAAGTAAGGAAATGG

GGAATCTGATATTATTCTCTCTCATCTCCAGAGCAACATTGGTGCTGTTGTAAAGATGTA

CTGTAGAAAAGTATTCTTCACCCAGCGTGACCCCCACAGAAGGTGTCAGGTAGACTTGAA

ATAAGCAAAGTAATAACCCAGCTCCCATACCCATAGTGGCAATTGTAGATTTCTATTGCC

CCAAAAGAGCCATACATAGGGATACTTACCTAGAAAGACAGAGGATCTTCCCTTGGTTTG

TGAAGAGGCAGCTAGTATATTTGTGTGTGTTTGCATAGATGCAAACGGTAAATAAATTCC

TAGGTTTATCAATACACAGTCAAACATTAAAATCTCTCATCTTGGCTGGGCACGGTGGCT

CACGCCTGTAATCCCAGCACTTTGGGAGGCCGAGGCGGGCGGATCATGAGGTCAAGAGAT

CAAGACCGTCCTGGGCAACATGGTGAAACCCCGTCTCTACTAAAAATACAAAAAATTAGC

TGGGTATGGTGGCACACGCCTGTAGTCCCAGCTACTCGGGAGGCTGAGGCAGGAGGATTG

CTTGAACCCGGGAGGCGGAGGTTGCAGTGAGCTGAGATGGTGCCACTGCACTCCAGCCTG

GCGATAGAGCAAGACTCCGTCTCAAACAACCAAACCAAAACAAAACAAAATATCTCACCT

TATCTTTGAAGACTAAGGAAAAAAAAAATCTCCCACTCATCGATACACTCCACAGAGGCA

GCATACTCTCCAAGTGTAGCTTTCTCTTTTCATGTTCATTATTCCCTTGGTGTTGGTTAT

TCTCAATGTCAATCATAACAGAACATCTTCCATAATAACAGTCCCAATTTAAGGAGCATT

AAGATAAAAGGTGGAATTGCCAAGGTCAATCCAGACGAGAACCTTCTCATAGAGGTAACC

ACCGTGTGGGTTTGGATGCTGGGAAGCAGGGGGACTATGACGCTACAAGGTCTCAGTCTT

AATTTTTGGAGTACTTCAGTCCCCAGGTATATTTTCCATAGATTTGGCCCTTAAATAAAG

AGAAGCTTCTGACTCTAAAATGTAAACAGTGCTTGTTACAGTCTTGTTGATATATTAAGA

AATTACTCACCTTATCTCATTTAATCTTAAAAACAAACCCCTGACAGGATCAAAACCACA

GCAGGGCTACATAATAGGAAAACTATACATAAATAGGTAGAATAATCTGCTCAGGATCAC

TAGGTAAGTTGCTGAATAAGAATTCAAGATGTTTTTGATCCCAGAGTTTAAAACCCAACC

TTTCAAACAGCGTTTCTTTCTTCTTAGAGTACAATGTTCTGAGAAAGAGATCCTCTGGAA

TTCTGGCCTAAGTGTATTTAATGCCCGGGTAAAGAAAGTGAGAGAACATTTCTCTTTAGG

GGCTGCTGCTGGATTTCTAAAAAGAAAATAATTTCTCAGCTAGTAACATGGAGCCAAACA

ACAGCTTCACAAGACTCTGGGTTCTTTAGCCCTCATCTCCTTCAATCCACCCTCTTTATA

ACCAGTCCTTCTTGTTTTTCCCCTCCCAGCTTTGTTCAGCAGCATGCCCTTCACCCAGAC

CTTGTCTTGTCACTCATCCCTACTCGCCATCATTCTTTCATTCCTCTTGGCCCAATCTCT

CTCCACTACTTCCTGCCTACATGTATGTAGGTTATTCATTTCCCTCTCTTGATTCCCCCC

ACCCAACTCTCTTTCTCCATTTCTTGCCTTTCAGAAGAACATGTGATCATCCAGGCCGAG

TTCTATCTGAATCCTGACCAATCAGGCGAGTTTATGTTTGACTTTGATGGTGATGAGATT

TTCCATGTGGATATGGCAAAGAAGGAGACGGTCTGGCGGCTTGAAGAATTTGGACGATTT

GCCAGCTTTGAGGCTCAAGGTGCATTGGCCAACATAGCTGTGGACAAAGCCAACCTGGAA

ATCATGACAAAGCGCTCCAACTATACTCCGATCACCAATGGTACCTCCCTCTCTGCTGCA

CTCCTGGACATGGGAATCCATAGTTTGAAAGTAGTTGCTTCAGCTCTTTGTGTTAGATTA

TTGTAACTGATTTTCCCTCCAAGGGTCTAACCTTGCCATTAACAAGCCCCAAATTCTCAT

GCCAGAGGTCTGAGAACTTTATGGGTTTGATCCTATCTTGTTGTGCTCAAGTCTTGTCTC

TGTCATCCATGGTCTCCTACGAAGTCATTGCCCTAAGTTCATGCTGGGGGAGCCAGAAGG

GAAGTCCTTGGATATCTTATACCTCAATATTGGCTCAATTTCTTGGGGAGGGGGTGCTGT

CAGAGATTGTTATCTGAGGATGTGACATAGATTTCTCAGGGCACAATTTCAACTACTTTT

TCAGCTTTAGGGTTTTTAGATACGTTTGTACCACAATTGAGCATGGGAGGGAGAGGGGTG

AGCCTAAGCAGTGATGGCTGATTTCTGTCATGTCTGTCATGTGTCCCCCAGTACCTCCAG

AGGTAACTGTGCTCACGAACAGCCCTGTGGAACTGAGAGAGCCCAACGTCCTCATCTGTT

TCATAGACAAGTTCACCCCACCAGTGGTCAATGTCACGTGGCTTCGAAATGGAAAACCTG

TCACCACAGGAGTGTCAGAGACAGTCTTCCTGCCCAGGGAAGACCACCTTTTCCGCAAGT

TCCACTATCTCCCCTTCCTGCCCTCAACTGAGGACGTTTACGACTGCAGGGTGGAGCACT

GGGGCTTGGATGAGCCTCTTCTCAAGCACTGGGGTATGGACCAACACTCAATCTCCTTTA

TTTCAAGGTTTCCTCCTATGATGCTTGTGTGAAACTCGGTGTTCTAACTGTTTCATAATA

TCTGCTACAATTAATATAACTGTCTTCTCCTCCTATCCAGCTTCCTCCTTTTTTTAATCT

GTAATTCTCTCAATACATCATTCTGTCTTCCTCTTCTTTAATCTATGAATAACTTTTCTC

TTTATTAAGAACCCTACATTTGATTCTGAGTGTTACTTCTTCCCACACTCATTACCATGT

ACTCTGCCTTATTTCCCCCCAGAGTTTGATGCTCCAAGCCCTCTCCCAGAGACTACAGAG

AATGTGGTGTGTGCCCTGGGCCTGACTGTGGGTCTGGTGGGCATCATTATTGGGACCATC

TTCATCATCAAGGGAGTGCGCAAAAGCAATGCAGCAGAACGCAGGGGGCCTCTGTAAGGC

ACATGGAGGTGAGTTAGGTGTGGTCAGAGGAAGACATATATGGAGATATCTGAGGGAGGA

AAACAGGGTGGGGAAAGGAAATGTAATGCATTTAAGAGACAAGGTAGGAACAGATGTGGC

TCTTGATTTCTCTTTGCTAGAACGAATCAGACATTGGTATCATCTGGTATCCCAAAGCTT

CAGGGTCTGTCATCCCTTTCTATAGACGGGCACCTTGATCACGGCTCCAGTCTTAGAAAT

CATCTCCAGTACCTAAAACCATTGTTTCACATTAGAATACTGAGTCTAGGGATCTAGAAA

ATACATTAGAATATGGAGTCTAGGGATCTAGAAAATACTGAGTCTAGGGATCTAGAAAAA

TAAGCCTCAAGATTTGGGCACATCCTAGCTTGTATTTCCTGGGGCAGGTCATCAGTTCAG

AAGCATTTCCAGATCCTGGCTCCTTTCAGGTTAGGGTCAATTCATTGCATGAAATGGGAA

TCTCTTAGAGGCCAATGCCTGCTTTTGCTTCTTTAGTCTCAAATGTAGTATGAGAAACTC

TAAAAAAAGGTAAAGCATGGTTGCTTATTATGTTCAGTTGGAGAGTAGGAACTAACTGTA

TACAGTTAGTTCATGTTGGAAAGGTTAGATGAACATTGAAAGAATTTTGCAAAGTCAAAG

GATTAAGAGAGAAGAGGAAGGAATCTGAAGCAAGGAGCTCAAAACAGATCTTAAATTCCT

TGGTAACTATGTGTGTCTTGCTATAGGTGATGATGTTTCTTAGAGAGAAGATCACTGAAG

AAACTTCTGCTTTAATGACTTTACAAAGCTGGCAATATTACAATCCTTGACCTCAGTGAA

AGCAGTCATCTTCAGCGTTTTCCAGCCCTATAGCCACCCCAAGTGTGGTTATGCCTCCTC

GATTGCTCCGTACTCTAACATCTAGCTGGCTTCCCTGTCTATTGCCTTTTCCTGTATCTA

TTTTCCTCTATTTCCTATCATTTTATTATCACCATGCAATGCCTCTGGAATAAAACATAC

AGGAGTCTGTCTCTGCTATGGAATGCCCCATGGGGCATCTCTTGTGTACTTATTGTTTAA

GGTTTCCTCAAACTGTGATTTTTCTGAACACAATAAACTATTTTGATGATCTTGGGTGGA

ATTTTTGGTGTTTAAGCCAGTTCTTTGGGTGGCGGTGGGGGGTGGGGAGTCGGTCCTGGG

GAATATATGTGATCCTTTCCCGGTAAAATATCTGAATGTTGAATTTATCTTATAAATTCT

AGAATTCATCAGACATATCCCGGTTCATTTGGGCTTGGTCTCATTTTGTGCATCTGCAGG

CAACCCTCTTGTTGTGGTCTAGTCCTCATCAGGAAAACCTAAAGTGGGGTTGGTTTGTTG

GGAGATCTCTA

>HLA00664 DRB1*01:01:01:01

TTTAACTCCATCTTTGAGAAACATTTAATAATGTAATGTGTTTGTCGTACAGGGTGAGTA

CAGATGCACGGGAGGCCATACGGTTTAGGCAAAGGGGAGCACAAAAGTTGAAGATGAGGC

GCTGTCATCAATGCTGGGACTTCAGGCCAAGGGCAGGAGCTGAGGAAGCCACAAGGGAGG

ACATTTTCTGCAGTTGCCGAACCAGTAGCAACCAGGTCCTGAGAAAGCCCTCTCTTGTGG

AAGAATAACTGCCAAGCAGGAAAGCTTTTCATTCTGCAAAGCTGGGACAGAAGGTTCTTC

CTTGAATGTGGTCATCTGCATTTCAGCTCAGGAATCCTGCAAAAGACAGAGGAGAGTGTT

GTTTTCAACCTGGCTCTACTAACAGTTTCTTTTCTCCTCTTTCAAGGGCTCAGATGAGAG

CACTGCAGGAAGCAGAAAAACAAGTTCCTGAGTCTCCCCTGAGCCAATAGTCCCGCAGAG

CACAGCCCTTTTCTAAGTGGAGAGGAGGAATTTTGGTGTAAATTGCCTGATCAGAAATTT

GGATCCAAAGTCTTTCCTATTATTTCTGTCTCATGCCTTATCACCTCTACCATCATTCTA

GTGTGTCCGCAGTTTGTTCCTTCCAGTGGGTTTGTGGTCTCGCTGACTTCAAGCAGACCT

TCACAGTGAGTGTTACAGCTCTTAAAGGCGGCAGGGACCCAAAGAGTAGGCAGCAGCAAG

ATTTATTGTGAAGATCAAAAGAACAAAACTTCCACAGCAGGGAAGAGGACCTGAGTGGGT

TGCCACTGCTGGTTGGGGTGGCCAGCTTTTATTCCTGTATTTGTCCCTGCCCATGTCCTG

CAGATTGGTCCATTTTAAAGAGTGCTGATTGGTCCATTTTAAAGAGTGCTGATTGGCCCA

TTTTAAAAGTGCTGATTGGTCCATTTTAAAGAATGCTGATTGGTCCATTTTACAGAGTGC

AAATTGGTCCATTTTACAAACATCCAGCTAGCCACAGAGAACCTATTGGTGTGTTTTTAC

AAAGCACTGATTGGGGCATTTAGAAACCTCTTGTAAGAAAAGTTCTCCAAGTCCCCACCC

CACCCAGAAGTCCAGCTGGCTTCACCTCTCACTAGGGAAACTGTGTCTGTTTCTAAAAGA

GGATTAAAAGGTATTACCTGTTGGCTGAAGTCCAGAGTGTCCTGGGAAAAAGAGGAAAAG

AAATACACTTAAAAGTTATTGAAGCAAATCTGTCCTCCAACACAATGTCCCAGCCGCAGA

TCTCCCACCTGAGATTTCTCTAACACCACAACCCGCACCAACCAGGGCAGAAAGGGGCAG

AAAGGAGCAGAGACAGACCATGTGACCCATGAAGCCTGAAGTGTCTGTCACAGGATCCAG

TGTAATTCCATTAGCCTTAGTGGCTCTTCCTTAATTTGCTCCAGGATCTCAAACAAAAGG

ACCCCTACTTGTTAACCTTCCTCTTGTCTCTGCAGGCCACAAGCTATTATGCTTTGACAT

AGTAACCATGCACTGATGATTTCTGGATTAGCAGGACATTAGAGCCGTTTGGGGAAAGAA

AGGCTTTATTCAGGGCCACTCGTATACTGAGAACTAACCTCTCAGCAAAACCATGGTTCC

TCCTCCAGAAAAGCCTATGGAGAGAGTCAGCTCCCAAAGGCTCCTCACCTTTCTGATTCC

TGAAGTAGATGAACAGCCCGGCCCCAAGGAAGAGCAGGCCCAGCACGAAGCCCCCGACTC

CACTCAGCATCTTGCTCTGTGCAGATTCAGACCGTGCTCCTGAGAGAGGAATCCAGGTTT

AGTGATGCTTATTCCAAATTGAACCTCTTTAATTGAGACTCTAAGATTCAGAGCTTTGAA

AATGGGGCAGAAGCCTGCCCCACAAGAATTAAAATAACTAGCCATTTCAGGAGAAAAAAA

GGATTTCAAATCACGCTGAACAGTTACGATGTTCAGACATCAAACTCATTCAAATATTAC

AGCCTTGATGTAAGGCAAGCGTTCAACATCTGATCAACAGAAAGCCTGAGACTCAATGAA

GCTAAGTAGTTTGTCTAGAGTGACAGAGCTAATAAAAGGCAGGGCTGAGATTGGATTCCC

CTCATGTCAGGTAGGCCCCTACACTTCTCCTCTTCCCAGATCACAAAAAATAACTCAGAG

CAACAGCACCAGAAACTCAGTCTCAGACCCAGAAGCAGGGCCTGGAGCCTGGGGAGAGTG

GGTGACCCTGACCTGCGAAATCATGGGGAGGTTCAAAAGAGGGACAGTCTCTCCTGCCTG

GCAGGTGAGACTGCTTCTCCAGGAGGTACAGGTGTTTCTAGAAACACCTACAGGGCTACC

CCCAGTGACCTGTGCTGATGGAGATGAGAACATGGAGCAAATGAAAATAGGGTGTGGGAG

AGGAGAAACCTGACACTCAGGGATTAGCACAGTCCCCTTCTTGGTGGGTGAGAAATTTAT

GAAGTCAGAAAGCTGCTCACTCCATTCCACTGTGAGAGGGCTCGTCACACTTGGGTGCTC

CACTTGGCAGGTGTAAACCTCTCCACTCCGAGGAACTGTTTCCAGCATCACCAGGGTCTG

GAAGGTCCAATCTCCATTCTGGATCAGGCCTGTGGACACCACCCCAGCCTTCTCTTCCTG

GCCGTTCCGGAACCACCTGACTTCAATGCTGCCTGGATAGAAACCACTCACAGAGCAGAC

CAGGAGGTTGTGGTGCTGCAGGGGCTGGGTCTTTGAAGGATACACAGTCACCTTAGGCTC

AACTAGGAGAAAACAAATGTAGAGAGAATGAGTCAGGAAGACAGAGTAAGTCTCCTGGTT

TGGCTGTGTGTCTGCTTCTCTGCAAACCCAGGCTCTGACCTTGACCAGGCCTATAGCACA

GCTGGCCATGTGACCTTACAGTGTCATCAGCCTGGAATTTAATCTTGACAGTGAGGACCC

ATTAGATTTGAGAGGTGTTGTGAAAAATTGTGTTTGTTTCTTCATAGCTTTAAATTGGCA

TGCATTGTCAAACTGTTTACAAATCTTGGAAAGTATAGAGTGTAGTAATTAAAACTGATA

TTTGAGCCAGGTTGCTTGGTTCGAATCCAAGGTCTGCCTTTTACTGGTTGATCCTGGAAG

AGTTTTTTGATTCTTCTGTGTCTCAACTTTGTCACCTACAATGAAGGATAATTATACTAA

TTTACCTCTTGGGGTTATATGAGGATTAATGCATGTAAAATATATAAAACAATGACTGAA

GATAGCCTTCAGTTTATAAGGTCAGAAAGCTTCTCACTCCATTCCACTGTGAGGGCGCTC

ATCACACTTGGGTGCTCCACTTGGCACCTATTTATCATCCTTGTACCCCATGACAGAAAA

ATATGATTTAAAGAAATATGCATAGATAAAGGGACAGAATTGGGTACATGAGGAAACCGA

GTATGAATTTTAGGAATACTACTGCCATGCACTCACACCTTAGAACAAAACAGAAATTGT

TCTGCCCCTGGGAATGTGGGACAGACAGAAATGATTCTCCAAATTTTTACGTTCCTAGAA

AAGCATGAGTCCCGAAGCAGAGAAAAGGATTAGGGAATGTCATTTTAGTTTTGAAAGTTC

TTATATTTACATTTAGCTGATCAATGCATCTCCCGTGTAACACAAGCATAATTATTATTA

GGCCTATCATTGTAAAATGATTTTTCTTTCCAGAGTCACATTTGGATTAAGGCAGTGTCT
[truncated: 201,050 more chars]
