# Supplementary material for: Complete sequences of six major histocompatibility complex haplotypes, including all the major MHC class II structures
Source: HLA. 2023 Mar 18;102(1):28–43. doi: 10.1111/tan.15020 (PMC10986641; doi:10.1111/tan.15020)
Supplement: Supplementary file 5 — Supplementary Table S1. Data and assembly generation summary. Illumina and Nanopore sequencing data summary for the sequenced cell lines, number of polishing rounds performed during the assembly process (see Methods), and BioSample and GenBank IDs. Supplementary Table S2. Data generation details. Illumina and Nanopore sequencing runs performed on the different cell lines, including details on the utilized DNA extraction, library preparation and sequencing protocols. Supplementary Table S3. Comparison to reference HLA types. Comparison on a per‐locus basis between the assembled haplotype sequences and reference HLA types from IPD‐IMGT/HLA. “Edit distance assembly ↔ IMGT/HLA ref.” specifies the edit distance between the assembled haplotype and the reference HLA type (“IMGT/HLA reference”); “IMGT/HLA min. edit distance” specifies the IPD‐IMGT/HLA reference allele that most closely matches the assembled haplotype, if not identical to the reference HLA type. For incompletely defined alleles, “Added bases” specifies the number of bases that could be completed based on the assembled haplotype. Supplementary Table S4. Assembly comparison to GRCh38 and Norman et al. (2017). Comparison between the haplotype assemblies generated here and earlier assemblies of the same haplotypes from GRCh38 and the scaffold dataset released by. 9 “Undetermined nucleotides” were quantified by counting the number of ‘N' characters in the corresponding assembly version; “missing bases” were quantified by mapping the earlier assemblies of the considered haplotypes against the versions presented here, retaining only unique alignments, and counting the number of bases with 0 coverage from the earlier‐assembly alignments. Supplementary Table S5. Interspersed repeats content. Proportion of assembled MHC sequences marked as interspersed repeats by the RepeatMasker 56 algorithm, reported separately for the complete MHC and the MHC class II subregion. [file TAN-102-28-s005.docx]

| **PacBio Hifi** | | | | | | | **Biosample IDs** | **GenBank** | **BioSample ID for Norman et al. 2017 data** |
| --- | --- | --- | --- | --- | --- | --- | --- | --- | --- |
| Total reads, number | Total reads, bases | Read N50 | Est. whole-genome coverage | MHC reads, number | MHC reads, bases | MHC coverage |  |  |  |
| 1,96E+06 | 3,15E+10 | 1,65E+04 | 10,17 | 3,38E+03 | 5,50E+07 | 11,16 | SAMN21531220 | OK649231 | ERS509501 |
| 2,49E+06 | 3,62E+10 | 1,48E+04 | 11,67 | 4,06E+03 | 5,87E+07 | 11,63 | SAMN21531221 | OK649232 | ERS509521 |
| 2,17E+06 | 3,17E+10 | 1,49E+04 | 10,22 | 3,86E+03 | 5,65E+07 | 11,25 | SAMN21531222 | OK649234 | ERS509560 |
| 2,22E+06 | 3,18E+10 | 1,44E+04 | 10,25 | 3,48E+03 | 4,91E+07 | 9,73 | SAMN21531223 | OK649236 | ERS509576 |
| 1,95E+06 | 3,25E+10 | 1,74E+04 | 10,48 | 3,28E+03 | 5,50E+07 | 11,20 | SAMN21531225 | OK649233 | ERS509543 |
| 1,52E+06 | 2,84E+10 | 1,90E+04 | 9,15 | 2,39E+03 | 4,44E+07 | 9,04 | SAMN21531224 | OK649235 | ERS509569 |

**Supplementary Table 1: Data and assembly generation summary.** Illumina and Nanopore sequencing data summary for the sequenced cell lines, number of polishing rounds performed during the assembly process (see Methods), and BioSample and GenBank IDs.

**Supplementary Table 2: Data generation details.** Illumina and Nanopore sequencing runs performed on the different cell lines, including details on the utilized DNA extraction, library preparation and sequencing protocols.

| **Technology** | **Instrument** | **Cell line** | **Run ID** | **Extraction method, protocol / kit** | **Number of reads (ONT, PacBio), Number of fragments (Illumina)** | **Bases** | **Read N50** | **Accession** |
| --- | --- | --- | --- | --- | --- | --- | --- | --- |
| Oxford Nanopore | GridION | QBL | qbl1 | HMW (Jain et al. 2018), LSK 108 | 3,07E+05 | 3,95E+09 | 26599 | SRX13286785 |
| Oxford Nanopore | GridION | QBL | qbl2 | HMW (Jain et al. 2018), LSK 108 | 3,07E+05 | 3,88E+09 | 26205 | SRX13286785 |
| Oxford Nanopore | GridION | DBB | dbb1 | HMW (Jain et al. 2018), LSK 108 | 3,52E+05 | 4,39E+09 | 30517 | SRX13286782 |
| Oxford Nanopore | GridION | DBB | dbb2 | HMW (Jain et al. 2018), LSK 108 | 3,59E+05 | 4,45E+09 | 33528 | SRX13286782 |
| Oxford Nanopore | GridION | MANN | mann1 | HMW (Jain et al. 2018), LSK 108 | 3,31E+05 | 3,07E+09 | 21204 | SRX13286783 |
| Oxford Nanopore | GridION | MANN | mann2 | HMW (Jain et al. 2018), LSK 108 | 5,58E+05 | 4,49E+09 | 17031 | SRX13286783 |
| Oxford Nanopore | GridION | MANN | mann3 | HMW (Jain et al. 2018), LSK 108 | 1,21E+06 | 6,35E+09 | 15374 | SRX13286783 |
| Oxford Nanopore | GridION | SSTO | ssto1 | HMW (Jain et al. 2018), LSK 108 | 1,42E+05 | 1,40E+09 | 28559 | SRX13286786 |
| Oxford Nanopore | GridION | SSTO | ssto2 | HMW (Jain et al. 2018), LSK 108 | 8,80E+04 | 8,43E+08 | 29607 | SRX13286786 |
| Oxford Nanopore | GridION | SSTO | ssto3 | HMW (Jain et al. 2018), LSK 108 | 1,44E+06 | 6,55E+09 | 13036 | SRX13286786 |
| Oxford Nanopore | GridION | SSTO | ssto4 | HMW (Jain et al. 2018), LSK 108 | 1,45E+06 | 6,55E+09 | 12586 | SRX13286786 |
| Oxford Nanopore | GridION | APD | apd1 | HMW (Jain et al. 2018), LSK 108 | 6,47E+05 | 7,39E+09 | 28398 | SRX13286781 |
| Oxford Nanopore | GridION | APD | apd2 | HMW (Jain et al. 2018), Ultralong | 2,52E+05 | 4,10E+09 | 89709 | SRX13286781 |
| Oxford Nanopore | GridION | QBL | qbl3 | HMW (Jain et al. 2018), LSK 108 | 2,48E+05 | 3,41E+09 | 34003 | SRX13286785 |
| Oxford Nanopore | GridION | APD | apd3 | HMW (Jain et al. 2018), Ultralong | 8,82E+04 | 1,78E+08 | 12244 | SRX13286781 |
| Oxford Nanopore | GridION | SSTO | ssto5 | HMW (Jain et al. 2018), Ultralong | 4,65E+04 | 9,71E+08 | 110246 | SRX13286786 |
| Oxford Nanopore | GridION | APD | apd4 | HMW (Jain et al. 2018), LSK 109 | 1,74E+06 | 1,28E+10 | 16783 | SRX13286781 |
| Oxford Nanopore | GridION | SSTO | ssto6 | HMW (Jain et al. 2018), LSK 109 | 4,32E+06 | 1,30E+10 | 5555 | SRX13286786 |
| Oxford Nanopore | GridION | DBB | dbb3 | HMW (Jain et al. 2018), LSK 109 | 1,39E+06 | 1,27E+10 | 31714 | SRX13286782 |
| Oxford Nanopore | GridION | APD | apd5 | HMW (Jain et al. 2018), LSK 109 | 1,85E+06 | 1,46E+10 | 17192 | SRX13286781 |
| Oxford Nanopore | GridION | DBB | dbb4 | HMW (Jain et al. 2018), LSK 109 | 1,18E+06 | 1,52E+10 | 37161 | SRX13286782 |
| Oxford Nanopore | GridION | MANN | mann4 | HMW (Jain et al. 2018), LSK 109 | 1,89E+06 | 1,05E+10 | 15762 | SRX13286783 |
| Oxford Nanopore | GridION | MANN | mann5 | HMW (Jain et al. 2018), LSK 109 | 1,98E+06 | 1,20E+10 | 17764 | SRX13286783 |
| Oxford Nanopore | GridION | QBL | qbl4 | HMW (Jain et al. 2018), LSK 109 | 1,74E+06 | 1,56E+10 | 24572 | SRX13286785 |
| Oxford Nanopore | GridION | QBL | qbl5 | HMW (Jain et al. 2018), LSK 109 | 2,11E+06 | 1,70E+10 | 22470 | SRX13286785 |
| Illumina | HiSeq 4000 | QBL | qbl_il1 | Qiagen Blood&Tissue | 2,10E+05 | 6,30E+07 | 150 | SRX13450050 |
| Illumina | HiSeq 4000 | DBB | dbb_il1 | Qiagen Blood&Tissue | 2,18E+05 | 6,53E+07 | 150 | SRX13450048 |
| Illumina | HiSeq 4000 | MANN | mann_il1 | Qiagen Blood&Tissue | 3,04E+05 | 9,13E+07 | 150 | SRX13450049 |
| Illumina | HiSeq 4000 | SSTO | ssto_il1 | Qiagen Blood&Tissue | 2,53E+05 | 7,58E+07 | 150 | SRX13450051 |
| Illumina | HiSeq 4000 | APD | apd_il1 | Qiagen Blood&Tissue | 2,67E+05 | 8,01E+07 | 150 | SRX13450047 |
| Oxford Nanopore | GridION | APD | apd6 | Qiagen Blood&Tissue, LSK 109 | 9,33E+05 | 8,37E+09 | 18979 | SRX13286781 |
| Oxford Nanopore | GridION | DBB | dbb5 | Qiagen Blood&Tissue, LSK 109 | 1,29E+06 | 1,60E+10 | 21342 | SRX13286782 |
| Oxford Nanopore | GridION | MANN | mann6 | Qiagen Blood&Tissue, LSK 109 | 1,09E+06 | 7,39E+09 | 31549 | SRX13286783 |
| Oxford Nanopore | GridION | SSTO | ssto7 | Qiagen Blood&Tissue, LSK 109 | 2,41E+06 | 1,29E+10 | 18184 | SRX13286786 |
| Oxford Nanopore | GridION | APD | apd7 | Qiagen Blood&Tissue, LSK 109 | 1,53E+06 | 1,08E+10 | 15618 | SRX13286781 |
| Oxford Nanopore | GridION | MANN | mann7 | Qiagen Blood&Tissue, LSK 109 | 1,72E+06 | 1,04E+10 | 20468 | SRX13286783 |
| Oxford Nanopore | GridION | SSTO | ssto8 | Qiagen Blood&Tissue, LSK 109 | 4,33E+06 | 1,53E+10 | 7311 | SRX13286786 |
| Oxford Nanopore | GridION | MANN | mann8 | Qiagen Blood&Tissue, LSK 109 | 7,13E+05 | 1,61E+10 | 31305 | SRX13286783 |
| Oxford Nanopore | GridION | APD | apd8 | Qiagen Blood&Tissue, LSK 109 | 6,20E+05 | 1,41E+10 | 32943 | SRX13286781 |
| Oxford Nanopore | GridION | APD | apd9 | Qiagen Blood&Tissue, LSK 109 | 5,75E+05 | 1,37E+10 | 34781 | SRX13286781 |
| Oxford Nanopore | GridION | KAS116 | kas116_1 | HMW (Jain et al. 2018), LSK 109 | 1,02E+07 | 1,92E+10 | 3459 | SRX13286784 |
| Oxford Nanopore | GridION | KAS116 | kas116_2 | HMW (Jain et al. 2018), LSK 109 | 1,42E+06 | 5,40E+09 | 22008 | SRX13286784 |
| Oxford Nanopore | PromethION | KAS116 | kas116_3 | HMW (Jain et al. 2018), LSK 109 | 9,89E+06 | 7,09E+10 | 22594 | SRX13286784 |
| Oxford Nanopore | PromethION | KAS116 | kas116_4 | HMW (Jain et al. 2018), LSK 109 | 9,00E+06 | 6,30E+10 | 20251 | SRX13286784 |
| PacBio Hifi | Sequel II/e | APD | 735-4 | Nanobind CBB | 1,96E+06 | 3,15E+10 | 16491 | SRR23270455 |
| PacBio Hifi | Sequel II/e | DBB | 735-16 | Nanobind CBB | 2,49E+06 | 3,62E+10 | 14836 | SRR23270454 |
| PacBio Hifi | Sequel II/e | KAS116 | 735-5 | Nanobind CBB | 1,95E+06 | 3,25E+10 | 17358 | SRR23270453 |
| PacBio Hifi | Sequel II/e | MANN | 735-2 | Nanobind CBB | 2,17E+06 | 3,17E+10 | 14932 | SRR23270452 |
| PacBio Hifi | Sequel II/e | QBL | 735-18 | Nanobind CBB | 1,52E+06 | 2,84E+10 | 19045 | SRR23270451 |
| PacBio Hifi | Sequel II/e | SSTO | 735-15 | Nanobind CBB | 2,22E+06 | 3,18E+10 | 14382 | SRR23270450 |

**Supplementary Table 3: Comparison to reference HLA types.** Comparison on a per-locus basis between the assembled haplotype sequences and reference HLA types from IPD-IMGT/HLA. “Edit distance assembly ↔ IMGT/HLA ref.“ specifies the edit distance between the assembled haplotype and the reference HLA type (“IMGT/HLA reference”); “IMGT/HLA min. edit distance” specifies the IPD-IMGT/HLA reference allele that most closely matches the assembled haplotype, if not identical to the reference HLA type. For incompletely defined alleles, “Added bases” specifies the number of bases that could be completed based on the assembled haplotype.

| Cell line | **HLA-A** | | | | | | |
| --- | --- | --- | --- | --- | --- | --- | --- |
|  | IMGT/HLA reference | Edit distance assembly ↔ IMGT/HLA ref. | IMGT/HLA min. edit distance | Edit distance assembly ↔ IMGT/HLA min. | IMGT/HLA complete | Added bases | Manual inspection |
| APD | A*01:01:01:01 | 0 |  |  | ✓ |  |  |
| DBB | A*02:01:01:01 | 0 |  |  | ✓ |  |  |
| MANN | A*29:02:01:01 | 0 |  |  | ✓ |  |  |
| SSTO | A*32:01:01:01 | 0 |  |  | ✓ |  |  |
| KAS116 | A*24:02:01:01 | 0 |  |  | ✓ |  |  |
| QBL | A*26:01:01:01 | 0 |  |  | ✓ |  |  |
|  |  |  |  |  |  |  |  |
|  | **HLA-B** | | | | | | |
|  | IMGT/HLA reference | Edit distance assembly ↔ IMGT/HLA ref. | IMGT/HLA min. edit distance | Edit distance assembly ↔ IMGT/HLA min. | IMGT/HLA complete | Added bases | Manual inspection |
| APD | B*40:01:02:01 | 0 |  |  | ✓ |  |  |
| DBB | B*57:01:01:01 | 0 |  |  | ✓ |  |  |
| MANN | B*44:03:01:01 | 0 |  |  | ✓ |  |  |
| SSTO | B*44:02:01:01 | 0 |  |  | ✓ |  |  |
| KAS116 | B*51:01:01:03 | 0 |  |  | ✓ |  |  |
| QBL | B*18:01:01:01 | 0 |  |  | ✓ |  |  |
|  |  |  |  |  |  |  |  |
|  | **HLA-C** |  |  |  |  |  |  |
|  | IMGT/HLA reference | Edit distance assembly ↔ IMGT/HLA ref. | IMGT/HLA min. edit distance | Edit distance assembly ↔ IMGT/HLA min. | IMGT/HLA complete | Added bases | Manual inspection |
| APD | C*06:02:01:01 | 0 |  |  | ✓ |  |  |
| DBB | C*06:02:01:01 | 0 |  |  | ✓ |  |  |
| MANN | C*16:01:01:01 | 0 |  |  | ✓ |  |  |
| SSTO | C*05:01:01:02 | 0 |  |  | ✓ |  |  |
| KAS116 | C*12:03:01:01 | 0 |  |  | ✓ |  |  |
| QBL | C*05:01:01:01 | 0 |  |  | ✓ |  |  |
|  |  |  |  |  |  |  |  |
|  | **HLA-DMA** | | | | | | |
|  | IMGT/HLA reference | Edit distance assembly ↔ IMGT/HLA ref. | IMGT/HLA min. edit distance | Edit distance assembly ↔ IMGT/HLA min. | IMGT/HLA complete | Added bases | Manual inspection |
| APD | DMA*01:01:01:04 | 0 |  |  | ✓ |  |  |
| DBB | DMA*01:02:01:01 | 0 |  |  | ✓ |  |  |
| MANN | DMA*01:01:01:01 | 0 |  |  | ✓ |  |  |
| SSTO | DMA*01:01:01:02 | 0 |  |  | ✓ |  |  |
| KAS116 | DMA*01:01:01:01 | 0 |  |  | ✓ |  |  |
| QBL | DMA*01:01:01:02 | 0 |  |  | ✓ |  |  |
|  |  |  |  |  |  |  |  |
|  | **HLA-DMB** | | | | | | |
|  | IMGT/HLA reference | Edit distance assembly ↔ IMGT/HLA ref. | IMGT/HLA min. edit distance | Edit distance assembly ↔ IMGT/HLA min. | IMGT/HLA complete | Added bases | Manual inspection |
| APD | DMB*01:01:01:03 | 0 |  |  | ✓ |  |  |
| DBB | DMB*01:01:01:02 | 0 |  |  | ✓ |  |  |
| MANN | DMB*01:01:01:01 | 0 |  |  | ✓ |  |  |
| SSTO | DMB*01:03:01:01 | 0 |  |  | ✓ |  |  |
| KAS116 | DMB*01:02:01:01 | 0 |  |  | ✕ | 847 |  |
| QBL | DMB*01:01:01:04 | 0 |  |  | ✓ |  |  |
|  |  |  |  |  |  |  |  |
|  | **HLA-DOA** | | | | | | |
|  | IMGT/HLA reference | Edit distance assembly ↔ IMGT/HLA ref. | IMGT/HLA min. edit distance | Edit distance assembly ↔ IMGT/HLA min. | IMGT/HLA complete | Added bases | Manual inspection |
| APD | DOA*01:01:02:01 | 1 | DOA*01:01:02:45 | 0 | ✕ | 72 | DOA*01:01:02:45 supported by read data |
| DBB | DOA*01:01:01:01 | 0 |  |  | ✓ |  |  |
| MANN | DOA*01:01:01:01 | 0 |  |  | ✓ |  |  |
| SSTO | DOA*01:01:04:01 | 0 |  |  | ✓ |  |  |
| KAS116 | DOA*01:01:05 | 0 |  |  | ✓ |  |  |
| QBL | DOA*01:01:02:03 | 0 |  |  | ✓ |  |  |
|  |  |  |  |  |  |  |  |
|  | **HLA-DOB** | | | | | | |
|  | IMGT/HLA reference | Edit distance assembly ↔ IMGT/HLA ref. | IMGT/HLA min. edit distance | Edit distance assembly ↔ IMGT/HLA min. | IMGT/HLA complete | Added bases | Manual inspection |
| APD | DOB*01:01:01:04 | 0 |  |  | ✓ |  |  |
| DBB | DOB*01:05 | 0 |  |  | ✓ |  |  |
| MANN | DOB*01:03:01:01 | 1 | DOB*01:03:01:02 | 0 | ✕ | 937 | DOB*01:03:01:02 supported by read data |
| SSTO | DOB*01:01:01:04 | 0 |  |  | ✓ |  |  |
| KAS116 | DOB*01:02:01:01 | 0 |  |  | ✕ | 937 |  |
| QBL | DOB*01:03:01:01 | 1 | DOB*01:03:01:02 | 0 | ✕ | 937 | DOB*01:03:01:02 supported by read data |
|  |  |  |  |  |  |  |  |
|  | **HLA-DPA1** | | | | | | |
|  | IMGT/HLA reference | Edit distance assembly ↔ IMGT/HLA ref. | IMGT/HLA min. edit distance | Edit distance assembly ↔ IMGT/HLA min. | IMGT/HLA complete | Added bases | Manual inspection |
| APD | DPA1*01:03:01:05 | 0 |  |  | ✓ |  |  |
| DBB | DPA1*01:03:01:02 | 0 |  |  | ✓ |  |  |
| MANN | DPA1*01:03:01:01 | 0 |  |  | ✓ |  |  |
| SSTO | DPA1*01:03:01:04 | 0 |  |  | ✓ |  |  |
| KAS116 | DPA1*02:01:01:01 | 1 |  |  | ✕ | 227 | Novel allele supported by read data |
| QBL | DPA1*01:03:01:01 | 0 |  |  | ✓ |  |  |
|  |  |  |  |  |  |  |  |
|  | **HLA-DPB1** | | | | | | |
|  | IMGT/HLA reference | Edit distance assembly ↔ IMGT/HLA ref. | IMGT/HLA min. edit distance | Edit distance assembly ↔ IMGT/HLA min. | IMGT/HLA complete | Added bases | Manual inspection |
| APD | DPB1*04:02:01:01 | 0 |  |  | ✓ |  |  |
| DBB | DPB1*04:01:01:02 | 8 | DPB1*04:01:01:01 | 0 | ✓ |  | Inconclusive, here 12 instead of ten AAGGs according to IMGT |
| MANN | DPB1*02:01:02:01 | 0 |  |  | ✓ |  |  |
| SSTO | DPB1*04:01:01:01 | 0 |  |  | ✓ |  |  |
| KAS116 | DPB1*13:01:01:01 | 0 |  |  | ✓ |  |  |
| QBL | DPB1*02:02:01:01 | 4 | DPB1*02:02:01:04 | 0 | ✓ |  | Inconclusive, here 10 instead of eleven AAGGs according to IMGT |
|  |  |  |  |  |  |  |  |
|  | **HLA-DQA1** | | | | | | |
|  | IMGT/HLA reference | Edit distance assembly ↔ IMGT/HLA ref. | IMGT/HLA min. edit distance | Edit distance assembly ↔ IMGT/HLA min. | IMGT/HLA complete | Added bases | Manual inspection |
| APD | DQA1*01:03:01:02 | 0 |  |  | ✓ |  |  |
| DBB | DQA1*02:01:01:01 | 0 |  |  | ✓ |  |  |
| MANN | DQA1*02:01:01:01 | 0 |  |  | ✓ |  |  |
| SSTO | DQA1*03:01:01:01 | 0 |  |  | ✓ |  |  |
| KAS116 | DQA1*01:01:01:01 | 1 |  |  | ✓ |  | Inconclusive, here 17 instead of 18 As in IMGT |
| QBL | DQA1*05:01:01:01 | 0 |  |  | ✓ |  |  |
|  |  |  |  |  |  |  |  |
|  | **HLA-DQB1** | | | | | | |
|  | IMGT/HLA reference | Edit distance assembly ↔ IMGT/HLA ref. | IMGT/HLA min. edit distance | Edit distance assembly ↔ IMGT/HLA min. | IMGT/HLA complete | Added bases | Manual inspection |
| APD | DQB1*06:03:01:01 | 0 |  |  | ✓ |  |  |
| DBB | DQB1*03:03:02:01 | 0 |  |  | ✓ |  |  |
| MANN | DQB1*02:02:01:01 | 1 |  |  | ✓ |  | Presence of novel allele confidently supported, but heterozygous |
| SSTO | DQB1*03:05:01 | 0 |  |  | ✓ |  |  |
| KAS116 | DQB1*05:01:01:03 | 0 |  |  | ✓ |  |  |
| QBL | DQB1*02:01:01:01 | 0 |  |  | ✓ |  |  |
|  |  |  |  |  |  |  |  |
|  | **HLA-DRA** | | | | | | |
|  | IMGT/HLA reference | Edit distance assembly ↔ IMGT/HLA ref. | IMGT/HLA min. edit distance | Edit distance assembly ↔ IMGT/HLA min. | IMGT/HLA complete | Added bases | Manual inspection |
| APD | DRA*01:01:01:01 | 2 | DRA*01:01:01:07 | 0 | ✓ |  | DRA*01:01:01:07 supported by read data |
| DBB | DRA*01:01:02 | 0 |  |  | ✓ |  |  |
| MANN | DRA*01:01:01:02 | 0 |  |  | ✓ |  |  |
| SSTO | DRA*01:01:01:03 | 0 |  |  | ✓ |  |  |
| KAS116 | DRA*01:01:01:01 | 29 | DRA*01:01:01:05 | 0 | ✓ |  | DRA*01:01:01:05 supported by read data |
| QBL | DRA*01:01:01:01 | 0 |  |  | ✓ |  |  |
|  |  |  |  |  |  |  |  |
|  | **HLA-DRB1** | | | | | | |
|  | IMGT/HLA reference | Edit distance assembly ↔ IMGT/HLA ref. | IMGT/HLA min. edit distance | Edit distance assembly ↔ IMGT/HLA min. | IMGT/HLA complete | Added bases | Manual inspection |
| APD | DRB1*13:01:01:02 | 0 |  |  | ✕ | 153 |  |
| DBB | DRB1*07:01:01:02 | 0 |  |  | ✓ |  |  |
| MANN | DRB1*07:01:01:01 | 0 |  |  | ✓ |  |  |
| SSTO | DRB1*04:03:01:01 | 2 |  |  | ✓ |  | Presence of novel allele supported by read data |
| KAS116 | DRB1*01:01:01:01 | 0 |  |  | ✕ | 134 |  |
| QBL | DRB1*03:01:01:02 | 0 |  |  | ✕ | 52 |  |
|  |  |  |  |  |  |  |  |
|  | **HLA-DRB3** | | | | | | |
|  | IMGT/HLA reference | Edit distance assembly ↔ IMGT/HLA ref. | IMGT/HLA min. edit distance | Edit distance assembly ↔ IMGT/HLA min. | IMGT/HLA complete | Added bases | Manual inspection |
| APD | DRB3*02:02:01:02 | 0 |  |  | ✓ |  |  |
| DBB |  |  |  |  |  |  |  |
| MANN |  |  |  |  |  |  |  |
| SSTO |  |  |  |  |  |  |  |
| KAS116 |  |  |  |  |  |  |  |
| QBL | DRB3*02:02:01:01 | 0 |  |  | ✕ | 29 |  |
|  |  |  |  |  |  |  |  |
|  | **HLA-DRB4** | | | | | | |
|  | IMGT/HLA reference | Edit distance assembly ↔ IMGT/HLA ref. | IMGT/HLA min. edit distance | Edit distance assembly ↔ IMGT/HLA min. | IMGT/HLA complete | Added bases | Manual inspection |
| APD |  |  |  |  |  |  |  |
| DBB | DRB4*01:03:01:02N | 0 |  |  | ✓ |  |  |
| MANN | DRB4*01:01:01:01 | 0 |  |  | ✕ | 12 |  |
| SSTO | DRB4*01:03:01:03 | 0 |  |  | ✓ |  |  |
| KAS116 |  |  |  |  |  |  |  |
| QBL |  |  |  |  |  |  |  |
|  |  |  |  |  |  |  |  |
|  | **HLA-E** | | | | | | |
|  | IMGT/HLA reference | Edit distance assembly ↔ IMGT/HLA ref. | IMGT/HLA min. edit distance | Edit distance assembly ↔ IMGT/HLA min. | IMGT/HLA complete | Added bases | Manual inspection |
| APD | E*01:01:01:01 | 0 |  |  | ✓ |  |  |
| DBB | E*01:01:01:01 | 0 |  |  | ✓ |  |  |
| MANN | E*01:03:02:01 | 0 |  |  | ✓ |  |  |
| SSTO | E*01:01:01:01 | 0 |  |  | ✓ |  |  |
| KAS116 | E*01:03:02:01 | 0 |  |  | ✓ |  |  |
| QBL | E*01:01:01:01 | 0 |  |  | ✓ |  |  |
|  |  |  |  |  |  |  |  |
|  | **HLA-F** | | | | | | |
|  | IMGT/HLA reference | Edit distance assembly ↔ IMGT/HLA ref. | IMGT/HLA min. edit distance | Edit distance assembly ↔ IMGT/HLA min. | IMGT/HLA complete | Added bases | Manual inspection |
| APD | F*01:01:01:09 | 0 |  |  | ✓ |  |  |
| DBB | F*01:01:01:08 | 0 |  |  | ✓ |  |  |
| MANN | F*01:01:01:08 | 0 |  |  | ✓ |  |  |
| SSTO | F*01:01:02:06 | 0 |  |  | ✓ |  |  |
| KAS116 | F*01:01:01:01 | 5 | F*01:01:01:19 | 0 | ✓ |  | F*01:01:01:19 supported by read data |
| QBL | F*01:01:01:08 | 0 |  |  | ✓ |  |  |
|  |  |  |  |  |  |  |  |
|  | **HLA-G** | | | | | | |
|  | IMGT/HLA reference | Edit distance assembly ↔ IMGT/HLA ref. | IMGT/HLA min. edit distance | Edit distance assembly ↔ IMGT/HLA min. | IMGT/HLA complete | Added bases | Manual inspection |
| APD | G*01:06:01:01 | 0 |  |  | ✓ |  |  |
| DBB | G*01:01:01:01 | 0 |  |  | ✓ |  |  |
| MANN | G*01:01:01:01 | 3 | G*01:01:01:04 | 0 | ✓ |  | G*01:01:01:04 supported by read data |
| SSTO | G*01:01:12 | 0 |  |  | ✓ |  |  |
| KAS116 | G*01:01:02:01 | 0 |  |  | ✓ |  |  |
| QBL | G*01:01:02:01 | 0 |  |  | ✓ |  |  |

**Supplementary Table 4: Assembly comparison to GRCh38 and Norman et al. (2017).** Comparison between the haplotype assemblies generated here and earlier assemblies of the same haplotypes from GRCh38 and the scaffold dataset released by ^9^. “Undetermined nucleotides” were quantified by counting the number of ‘N’ characters in the corresponding assembly version; “missing bases” were quantified by mapping the earlier assemblies of the considered haplotypes against the versions presented here, retaining only unique alignments, and counting the number of bases with 0 coverage from the earlier-assembly alignments.

| **Cell line** | **GRCh38** | | | | **Norman et al. 2017 (scaffold)** | | |  | **This study** | |
| --- | --- | --- | --- | --- | --- | --- | --- | --- | --- | --- |
|  | Accession | Length | Missing bases (compared to new assembly) | Undetermined nucleotides (N) | Accession | Length | Missing bases (compared to new assembly) | Undetermined nucleotides (N) | Length | Undetermined nucleotides (N) |
| APD | GL000520.2 | 4.672.374 | 2.557.139 | 2.301.543 | CZSY02000001 | 4.952.522 | 81.505 | 2.377 | 4.928.029 | 0 |
| DBB | GL000522.2 | 4.604.811 | 849.430 | 406.094 | CZSY02000001 | 4.981.139 | 185.701 | 2.666 | 5.048.108 | 0 |
| MANN | GL000523.2 | 4.677.643 | 930.605 | 582.522 | CZTY02000001 | 4.944.856 | 154.680 | 2.682 | 5.025.203 | 0 |
| QBL | GL000525.2 | 4.606.388 | 614.949 | 316.659 | CZUB02000001 | 4.907.551 | 72.137 | 3.542 | 4.904.614 | 0 |
| SSTO | GL000526.2 | 4.929.269 | 870.054 | 755.016 | CZUK02000001 | 4.947.421 | 167.593 | 3.501 | 5.045.615 | 0 |
| KAS116 | - | - | - | - | CZSR02000001 | 4.721.327 | 244.556 | 9.114 | 4.907.004 | 0 |

**Supplementary Table 5: Interspersed repeats content.** Proportion of assembled *MHC* sequences marked as interspersed repeats by the RepeatMasker ^56^ algorithm, reported separately for the complete *MHC* and the *MHC class II* subregion.

| **Cell line** | **Repeat element content** | |
| --- | --- | --- |
|  | Whole MHC | MHC II |
| APD | 51,27% | 56,83% |
| COX | 51,44% | 56,86% |
| DBB | 51,69% | 63,30% |
| KAS116 | 51,42% | 61,49% |
| MANN | 51,95% | 63,16% |
| PGF | 51,66% | 62,55% |
| QBL | 51,33% | 57,01% |
| SSTO | 51,67% | 63,02% |
